# Automated design of genomic Southern blot probes

**DOI:** 10.1186/1471-2164-11-74

**Published:** 2010-01-29

**Authors:** Mike DR Croning, David G Fricker, Noboru H Komiyama, Seth GN Grant

**Affiliations:** 1 Genes to Cognition Programme, The Wellcome Trust Sanger Institute, Hinxton, Cambridge, CB10 1SA, UK

## Abstract

**Background:**

Sothern blotting is a DNA analysis technique that has found widespread application in molecular biology. It has been used for gene discovery and mapping and has diagnostic and forensic applications, including mutation detection in patient samples and DNA fingerprinting in criminal investigations. Southern blotting has been employed as the definitive method for detecting transgene integration, and successful homologous recombination in gene targeting experiments.

The technique employs a labeled DNA probe to detect a specific DNA sequence in a complex DNA sample that has been separated by restriction-digest and gel electrophoresis. Critically for the technique to succeed the probe must be unique to the target locus so as not to cross-hybridize to other endogenous DNA within the sample.

Investigators routinely employ a manual approach to probe design. A genome browser is used to extract DNA sequence from the locus of interest, which is searched against the target genome using a BLAST-like tool. Ideally a single perfect match is obtained to the target, with little cross-reactivity caused by homologous DNA sequence present in the genome and/or repetitive and low-complexity elements in the candidate probe. This is a labor intensive process often requiring several attempts to find a suitable probe for laboratory testing.

**Results:**

We have written an informatic pipeline to automatically design genomic Sothern blot probes that specifically attempts to optimize the resultant probe, employing a brute-force strategy of generating many candidate probes of acceptable length in the user-specified design window, searching all against the target genome, then scoring and ranking the candidates by uniqueness and repetitive DNA element content. Using these *in silico *measures we can automatically design probes that we predict to perform as well, or better, than our previous manual designs, while considerably reducing design time.

We went on to experimentally validate a number of these automated designs by Southern blotting. The majority of probes we tested performed well confirming our *in silico *prediction methodology and the general usefulness of the software for automated genomic Southern probe design.

**Conclusions:**

Software and supplementary information are freely available at: http://www.genes2cognition.org/software/southern_blot

## Background

Southern blotting is a DNA analysis technique that allows one to detect a specific DNA sequence in a complex DNA sample [1]. Gel electrophoresis is used to size separate restriction-digested DNA, which is then transferred (or blotted) to a solid support such as a filter for probing and detection by radioactive or luminescent labelling.

The method has found widespread application throughout molecular biology. It has been used for gene discovery and mapping. It also has diagnostic and forensic applications, such as mutation detection in patient samples and DNA fingerprinting in criminal investigations. It has been employed as the definitive method for detecting transgene integration [2], and successful homologous recombination in gene targeting experiments that ablate or modify a gene's function *in vivo *[3].

For the technique to succeed one needs to identify a probe sequence that is unique within the genome for the gene or locus of interest so that it does not cross-hybridize with other endogenous DNA sequences present in the sample. Like others we have routinely used a manual approach to design and test our probes, which is labor intensive and usually requires trial of different probes before the desired result is obtained. It is therefore highly desirable to have bioinformatic tools that aid in the design process and can also optimize the probes.

The typical manual approach is to choose a probe of at least 300 bp in length, to ensure efficient labeling in the random priming reaction [4], and in practice probes of 500-1000 bp are generally employed. Following identification of the genomic locus one wishes to probe, the DNA sequence from a genome browser (such as Ensembl [5]) is examined for repetitive sequence elements as these can result in an intense background smear on hybridization that obscures single copy gene hybridization signals. The test probe is then searched against the genome using BLAST [6] or other means and the results inspected. One hopes to obtain a single perfect match to the target locus on the genome, with little or no cross-reactivity to other loci. If this is not the case, one has to return to the genome browser and move and/or shorten the sequence before repeating the BLAST search. With each genome search taking several minutes this is a time consuming exercise and is unlikely to yield the best possible probe.

Clearly this method is amenable to bioinformatic automation. Many programs already exist for oligonucleotide probe discovery, principally in the area of microarray design [7]. These programs are generally designed to find probes less than 100 bp rendering them inapplicable for the considerably longer Southern blot probes. To address this need we have written a system to find (near) unique probes in a specified region of a genome, which contain little or no repetitive DNA sequence, and also to design PCR primers to facilitate the recovery of the probes from cellular DNA for subsequent Southern blotting. We went on to experimentally validate a number of these designs by Southern blotting in the mouse genome.

## Implementation

**Figure 1:**
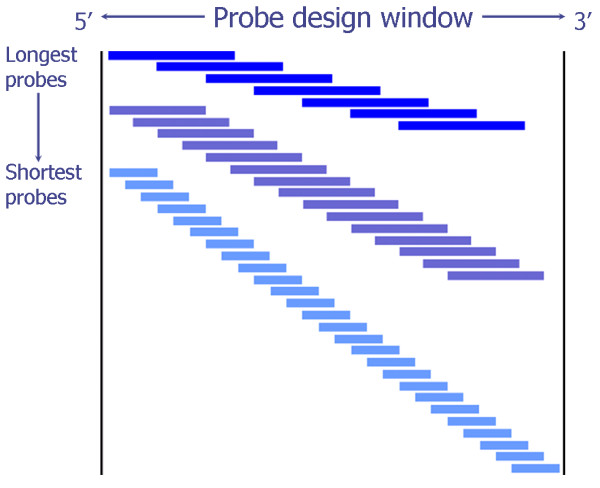
**Tiling approach to Southern blot probe design**. Many potential probes are generated and tested in the user-specified genomic window, starting from the maximum allowable probe length (default 1300), tiling the window by moving by a small percentage of the probe length each time (default 5%). Once this is completed the probe length is reduced by 50 bases (configurable) and the window re-tiled generating more candidate probes. The process is repeated until the minimum probe length is reached (default 500).

Given user-supplied chromosomal coordinates, and a desirable size range for the southern blot probe (default 500-1300 bp), we used a tiling approach to generate many possible probes in the specified design window. The program starts from the maximum allowable probe length, tiling the window by moving by a small percentage of the probe length each time (default 5%). Once this is completed the probe length is reduced by 50 bases (configurable) and the window re-tiled, generating more candidate probes. The process is repeated until the minimum probe length is reached (see Figure 1). The candidate probes are searched against the target genome using the Exonerate pairwise sequence alignment program [8].

We calibrated the method using a set of manually-designed genomic probes that we have previously successfully employed for Southern blotting (see Table 1). These had an average length of ~800 bp and when searched with Exonerate (with parameters --model affine:local --score 150) all of these produced a perfect match to their genomic locus (as would be expected) and a number of additional lower-scoring alignments to other loci. On average these second best matches spanned 17.5 ± 5.8% (mean ± standard error) of the probe length, with 74.6 ± 3.2% DNA sequence identity (n = 8). From the scores of the on-target or 'self-hit' and the highest scoring off-target locus alignments we calculated a score ratio as measure of uniqueness of the candidate probe. Our calibration probes averaged 19.5 ± 3.6 (n = 8). This score ratio is proportional to both the length and sequence identity of the two matches.

**Table 1:** Calibration of automated design pipeline with 8 manually-designed and experimentally-validated Southern blot probes.

Probe Name	Mouse Genomic Target	Length (bases)	Score ratio (self/second hit)	Second hit identity (%)	Second hit query coverage (%)	Repetitive & low-complexity DNA (%)
Dusp6_5prime_probe	*Dusp6 *(5')	946	30.1	71	8	3.2

SAP102_5prime_PDZ3_probe	*Dlg3 *(5')	969	27.2	72	8	2.7

Dusp6_3prime_probe	*Dusp6 *(3')	1004	29.4	61	13	4.5

Actb_probe	*Actb*	881	22.8	91	6	6.7

SAP102_3prime_probe	*Dlg3 *(3')	886	22.2	77	8	19.4

NR2B_probe	*Grin2b*	567	11.1	81	14	9.5

SAP102_5prime_probe	*Dlg3*	784	9.89	68	29	81.7

PSD-95_exon_9_probe	*Dlg4 *(exon 9)	296	3.3	76	54	nd

**Average ± standard error**		**791.6 ± 85.9**	**19.5 ± 3.6**	**74.6 ± 3.2**	**17.5 ± 5.8**	**18.2 ± 10.8**

Shown are the mouse genomic target and length of each of the probes, together with uniqueness and repetitive DNA content measures produced by evaluating the probes in the automated design pipeline. These are the score ratio, which is calculated as the ratio of the score of the on-target hit of the probe to the genomic locus of interest, and the highest-scoring off-target second best hit. The percentage repetitive and low-complexity DNA content (as estimated by RepeatMasker and DUST) is shown for each of the probes. Finally, the percentage identity and query coverage are given for the second hit used to calculate the score ratio.

Comparing the probe sequences to a version of the genomic assembly that has been screened for repeats and low-complexity regions by RepeatMasker [9] and DUST [10] allows us to estimate the repetitive DNA content of individual probes. Our calibration probes contained 18.2 ± 10.8% such DNA.

Considering these results we chose a minimum score ratio of 10 and a maximum combined repetitive and low-complexity base content of 5% as the minimum requirements for probe acceptance (configurable). Candidate probes reaching these criteria that were completely overlapped by a longer and better-scoring probe are considered redundant and removed from the passing set.

With the number of genome searches to be carried out potentially taking several hours for each Southern blot probe design, we thought that employing a single program and computer to complete the whole task was unlikely to achieve a reliable and timely solution. Instead we decided to use a database to store and retrieve the design information for each probe, and subsequently to hold the results of the many genome searches carried out for candidate probes. Multiple processors and cores as available from a compute cluster are employed to perform the genome searching, reducing the real time taken to test the probe designs *in silico*. When all the searches are complete the whole set of genome-search results are analyzed to find the best probe candidates.

A MySQL database (12 tables) was designed for this purpose together with a set of Perl data objects and SQL adaptor classes to allow programs to write and retrieve from the database. These follow the Ensembl API and schema design where one creates a set of classes representing the core objects in the system, in this case probe designs, candidate probe sequences belonging to a design to be tested, and their matches to the target genome, partnered by a set of complementary adaptor classes that hold the cognate SQL necessary for storing and retrieving these from the database. Changes to the database schema can the then be made without impact on the object classes [11].

We then decomposed the task into three principal steps:

1) *create_probe_search *takes the user-specified chromosomal coordinates for the design and generates many candidate probes at the granularity governed by the window tiling parameters, storing the design specification and the candidate probe sequences in the database. Use is made of the Ensembl API to extract the DNA sequence (or Slice) from the genome assembly covering the probe design window, then subsequently to extract sub-sequences to generate each candidate probe sequence. These sequences are grouped into batches (or jobs) for efficient searching with Exonerate in step 2.

2) *run_probe_search *takes the set of sequences specified by a particular job, searches them against the target genome, and parses the Exonerate output results, storing the hits, including their scores, location, and masked sequence content (as ascertained from the soft-masking) in the database. *run_probe_search *is not launched interactively but is initiated by *submit_probe_search *that utilizes the LSF job scheduling system to run many separate instances of *run_probe_search *in parallel to complete the genome searches required for a probe design.

3) *analyse_probe_search *is the final step in the probe design process. It checks that all the genome-searching jobs for the probe design have been completed successfully, then fetches the alignment results from the database, applying the specified cut-off criteria for score-ratio and repetitive/low-complexity DNA content. These are used to separate and rank the sequences into unique, passed and failed groups. Redundant (but passed) sequences are filtered into a fourth bin.

If none of the sequences in the probe design pass at the specified criteria, the cut-offs are automatically relaxed to find the best (but poorly-scoring) probes in what is likely to be a difficult portion of the genome to design Southern blot probes. Primer3 [12] is then used to generate primers for recovery of the passed candidate probes, run using the BioPerl-Run wrapper [13]. Chosen primers can be manually-checked for potentially confounding polymorphisms if required, by search of dbSNP.

**Figure 2:**
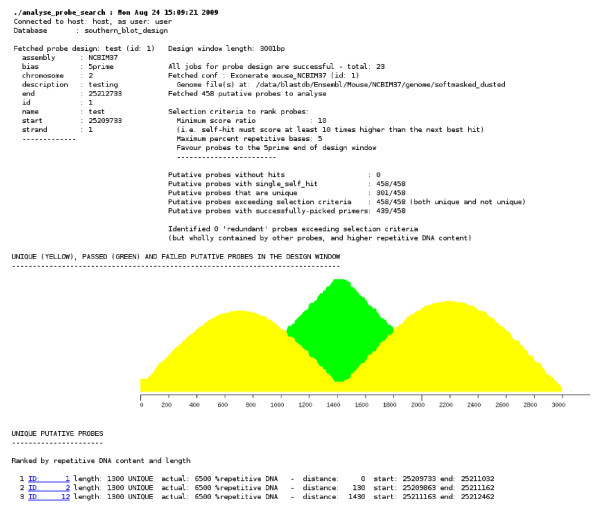
**Screenshot of final web output following a completed Southern blot probe design, search and analysis run**. In this example a probe is designed against a 3 kb window on chromosome 2 of the NCBI37 mouse genomic assembly. 458 candidate probes were tested and many unique probes were found, 301/458 (shown in yellow). The remainder (157/458) passed the empirical cut-off criteria (shown in green) suggesting this region of the chromosome is likely composed of quite unique sequence, almost free from repetitive and low-complexity DNA elements.

Static web output is generated for user inspection (see Figure 2). This includes the probe design window coordinates, counts of the candidate probes generated and subsequently placed in each bin and results of the quality assurance checks that each genome search generates an 'on target' hit to the correct position on the genome for the candidate probe sequence. A graphic plot is rendered showing the frequency of occurrence of each base position in the set of sequences found in the unique, passed or failed probe groups across the design window.

Four accessory scripts are also provided. *create_probe_search_db_tables *creates the MySQL database tables for the design pipeline. *delete_probe_design *removes a probe design from the database should it have been wrongly specified, or is no longer needed. *delete_job_results *removes the results of a particular batch of Exonerate genome searches from the database should an error have occurred, allowing the job to be resubmitted, and finally, *get_probe_search_cpu_time *calculates the total time to execute the searches for a given probe design.

Each of these programs read their customizable parameters from a .ini type configuration file.

## Results and Discussion

**Figure 3:**
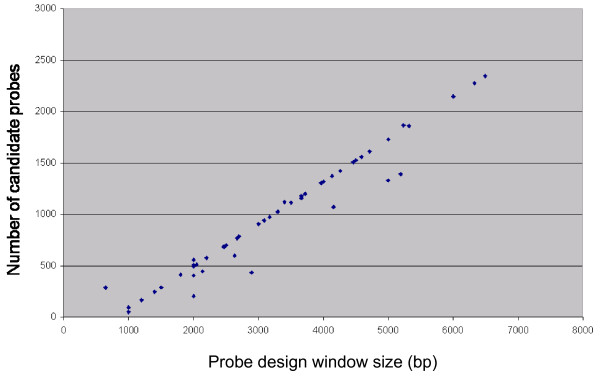
**Relationship between genomic design window size and the number of candidate probes generated by tiling**. The relationship is found to be linear. Using a 3 kb input window and acceptable probe length in the range 500-1300 bp, approximately 900 candidate probes are generated. Points lying off the main line arise from probe design searches where the tiling granularity has been reduced to try to find a suitable probe in difficult to design loci.

To date we have designed 124 probes using flanking regions in about 60 genes that we chose to perturb by gene targeting in mouse embryonic stem cells. Given a ~3 kb window in which to search for a Southern blot probe and a desirable length range for the final probe of 500-1300 bp, the tiling strategy outlined produces on average ~900 candidate probes (when used with the default granularity) to search against the genome (see Figures 1 and 3).

In total 103/124 (83%) of these designs passed by the criteria above of score ratio ≥ 10 and repetitive/low-complexity DNA content ≤ 5%. On average the best candidate probes for each design were 818.1 ± 25.0 bp long and contained only 4.1 ± 1.0% repetitive and low complexity DNA, the latter being significantly lower than our manually-designed calibration set (p < 0.05, Student's t-test, Table 2).

**Table 2:** Comparison of manually-designed (calibration) and automatically-designed Southern blot probes.

Probe set	Length (bases)	Average score ratio (self/second hit)	Average repetitive & low-complexity DNA (%)	Unique hit to genome	Passed empirical selection criteria
**Calibration** **(n = 8)**	791.6 ± 85.9	19.5 ± 10.8	18.2 ± 10.8	0/8	3/8

**Automated** **(n = 124)**	818.1 ± 25.0	23.7 ± 1.3	4.1 ± 1.0*	62/86	103/134

Shown are the average length (± standard error) of the probes in the calibration and automatically designed sets, together with their average score ratio, which is calculated as the ratio of the score of the on-target hit of the probe to the genomic locus of interest, and the highest-scoring off-target second best hit. The average combined percentage repetitive and low-complexity DNA content (as estimated by RepeatMasker and DUST) is shown for each of the probes. Also shown are the number of probes in each set that had a unique hit to their target genomic locus, with no off-target hits i.e. no apparent cross-reactivity. * p < 0.05 Student's t-test.

Additionally by such brute-force searching and scoring of genomic probes, in exactly half the design cases (62) it was possible to find one or more unique probes amongst those tested. These had a single (i.e. ideal) hit to their target genomic locus with no cross-reactivity to other loci. It is worth noting that none of the calibration probes gave this 'ideal' result, when evaluated using the same exonerate search parameters.

The remaining probes (97/124) that passed our empirical cut-off criteria, had an average score-ratio of 23.7 ± 1.3, which was not significantly different to the experimental calibration set (p > 0.05; Student's t-test).

**Figure 4:**
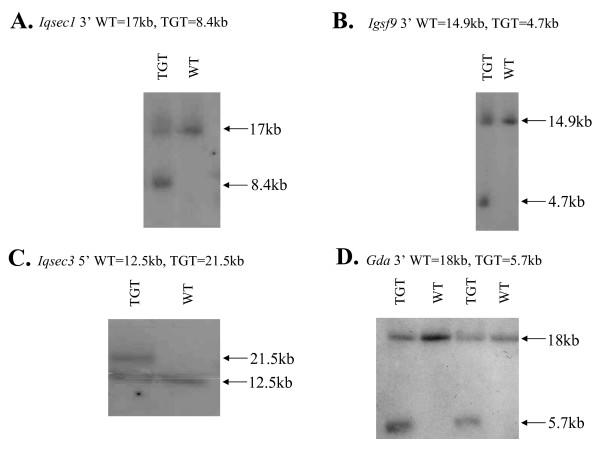
**Experimental validation of Southern blot probes**. Each lane on the four blots A-D contains mouse genomic DNA extracted from a separate embryonic stem (ES) cell line, that was restriction-digested and size-separated on an 0.6% by gel electrophoresis before blotting. The gel was run for 16-18 hr at 20-22 V, and a molecular weight standard of HindIII-digested Lambda DNA was employed. Panels A-D were hybridized with probes designed against four distinct mouse loci situated near the *Iqsec1*, *Igsf9*, *Iqsec3 *and *Gda *genes in order to confirm whether homologous recombination had taken place, ablating or modifying the gene's function. In each case examples of wild-type (WT) and gene-targeted (TGT) DNA are revealed by the presence of the extra band in the TGT ES clones, at a differing molecular weight.

In order to confirm the system does design effective Southern blot probes we experimentally tested 16 of the *in silico *designs. Blots were performed on mouse genomic DNA extracted from embryonic stem cell lines in order to confirm homologous recombination had occurred thus correctly targeting the gene to be ablated as part our of our high-throughput mouse knockout and molecular neurobiological phenotyping programme [14]. 13/16 of the probes tested gave a usable signal upon blotting, the remainder gave a smear likely indicative of non-specific probe binding, or no resolvable signal. Representative Sothern blots are shown in Figure 4.

## Conclusions

We have developed an automated system for the effective design of Southern blot probes. Many candidate probes that lie in a given genomic window are searched against the target genome in a brute-force approach to finding the best probe in the locus, as assessed by uniqueness and repetitive DNA sequence content. Using these *in silico *measures we can automatically design probes that would be predicted to perform as well, or better, than previous manual designs, while reducing the time taken by the molecular biologist to yield a successful probe. The majority of the probes we tested experimentally in Southern blotting performed well confirming our *in silico *prediction methodology, and the usefulness of the software for automated genomic Southern blot probe design.

## Availability and requirements

• **Project name: **southern_blot

• **Project home page: **http://www.genes2cognition.org/software/southern_blot and Additional file 1.

• **Operating system(s) **UNIX and Linux variants

• **Programming language: Perl and SQL**

• **Other requirements: **BioPerl core 1.5.0 or higher, BioPerl run 1.4 or higher, Ensembl core 32 or higher, Config::IniFiles 2.38 or higher, DBI 1.32 or higher, GD 2.17 or higher, Exonerate 1.0.0, Primer3 1.0.0, LSF 5.1 or higher, MySQL 5.045 or higher

• **License: **Artistic License 2.0

• **Any restrictions to use by non-academics: **none

## Abbreviations

API: application programming interface; BLAST: basic local alignment search tool; bp: base pairs; DNA: deoxyribonucleic acid; kb: kilobase; LSF: load sharing facility; PCR: polymerase chain reaction; SQL: structured query language.

## Authors' contributions

MDRC conceived and implemented the method. DGF experimentally validated the resulting probes. NHK and SGNG directed the investigation. All authors read and approved the final manuscript.

## Supplementary Material

Additional file 1 **Software package for automated design of genomic Southern blot probes**. Archive of all the components of the pipeline packaged using "tar", and subsequently compressed with "gzip". Includes source code, example configuration files, example output, and a user's guide for installation. Click here for file
