# Supplementary material for: Automated design of genomic Southern blot probes
Source: BMC Genomics. 2010 Jan 29;11:74. doi: 10.1186/1471-2164-11-74 (PMC2830989; doi:10.1186/1471-2164-11-74)
Supplement: Additional file 1 — Software package for automated design of genomic Southern blot probes. Archive of all the components of the pipeline packaged using "tar", and subsequently compressed with "gzip". Includes source code, example configuration files, example output, and a user's guide for installation. [file 1471-2164-11-74-S1.GZ › southern_blot_design/docs/example_run_output/test_probe_search_primers.html]

test - Primers for putative probes

```
./analyse_probe_search : Mon Aug 24 15:09:25 2009
Connected to host: ens-research, as user: ensadmin
Database       : g2c_southern_blot_design

Primers for recovering probe design: test (id: 1)

Primer3 parameters
  PRIMER_GC_CLAMP     : 1
  PRIMER_MAX_GC       : 50
  PRIMER_MAX_SIZE     : 22
  PRIMER_MAX_TM       : 60
  PRIMER_MIN_GC       : 40
  PRIMER_MIN_SIZE     : 20
  PRIMER_MIN_TM       : 55
  PRIMER_NUM_RETURN   : 5
  PRIMER_OPT_SIZE     : 21
  PRIMER_SALT_CONC    : 50
  -------------------

NOTE Tm QUOTED IN RESULTS BELOW ARE ACCORDING TO THE FORMULA:   Tm = (length * 2) + (GC * 2) - 5

UNIQUE PUTATIVE PROBES
----------------------

Primers for ID: 1
Primer pair 1, product size : 1063
  Primer left                            Primer right
  start: 25209909                        start: 25210951
  end  : 25209929                        end  : 25210971
  tm   : 57                              tm   : 57
  seq  : GATGGCATGAAAGCAGATAGC           seq  : TATACAAGGAGGGCCAAAGAG

  >1_primer_pair_1_product
  GATGGCATGAAAGCAGATAGCTACAAGGCTCTTGGACCAGGCTAGCACTGGGTCCTGCAC
  CCAGGGAGAGCCACCTCACCTTGACAGGGTTGGCAGGAAGGGGCCCTAGAAAGTCAGTAG
  GATACGGGTAGTCCATCATGGCGAGCACAGTAAATGCATTTCGGGCAAACCCAAAGAGCT
  GAGTCAGGTCCTTTGGGCTGGAAAGTGATTGACAGGTACCAAAGTTCTGGCTGATGGTGT
  CATAGGCTGGGAAGAGAGAGGCCAGGAGAAAAGGCTGAGGAAACTGCTGGCAAATGTGAA
  GGGCAAGAATGAATGCCCAAGGTGGGCAGCAGGTGAGGAAAGAGTCCCTCACCTCCCTGG
  AGGAACAAGTCTTTGATTTGCTGAAAGGCATCCCGCACAGCCTGGGCGCACTTGGGACTC
  TGGCCATAAAAGTCCTGGAGAAGAGACCAAGGTTGCTGCTGCCATTCTTGCACTGGCCTG
  GGGTACCCAAGTCCCCTCACTCACCGCTGTGACATCTCGGAAGAATTGGTAGGAGTCCCC
  AAGGCCTGCAACAGCTACAACAGGAGCGCTGGCTGCCAGTGCCCCAGCCACCAGGTGGGG
  GTACTTCATCCTCATGTAGGCACTCAGCATCCCCCCATAACTGGGAGTACAGAGCACAGA
  TCATGGTTGTGGGAAGCTGCCCACAACTCAGGCGAGCAGCCTCACTGTCCTCCAGGCTGA
  GGTGCTAGGCTGCTCTTTCCCTGCTCAGAACGCCCAAGGGTGGGAAAGAAGGACCTGAAA
  CTGTCAGGCCCACACACCCTGATCCCAGGGCCAAGGCAGATACAGCCTTCACTGGGAGAA
  GGCACCTGTGGGTGCCCTGCCCTGACCCAGCAATGAAGACATTGCAGAGACAAAGTCAGA
  AGGAATTGTCCCACTAGTGGGAACAACATAGCATACACTGCCTATGAGGTCCACTCAAGG
  AGGGCTTCCAGAAGGAGGTAAAGCTAGACCCCGCCCTTCCACATGTGGGGTAGGCATAGG
  ATGTTGAGACTGTAAGAGACATCTCTTTGGCCCTCCTTGTATA
  --------------------

Primer pair 2, product size : 1064
  Primer left                            Primer right
  start: 25209909                        start: 25210951
  end  : 25209929                        end  : 25210972
  tm   : 57                              tm   : 61
  seq  : GATGGCATGAAAGCAGATAGC           seq  : CTATACAAGGAGGGCCAAAGAG

  >1_primer_pair_2_product
  GATGGCATGAAAGCAGATAGCTACAAGGCTCTTGGACCAGGCTAGCACTGGGTCCTGCAC
  CCAGGGAGAGCCACCTCACCTTGACAGGGTTGGCAGGAAGGGGCCCTAGAAAGTCAGTAG
  GATACGGGTAGTCCATCATGGCGAGCACAGTAAATGCATTTCGGGCAAACCCAAAGAGCT
  GAGTCAGGTCCTTTGGGCTGGAAAGTGATTGACAGGTACCAAAGTTCTGGCTGATGGTGT
  CATAGGCTGGGAAGAGAGAGGCCAGGAGAAAAGGCTGAGGAAACTGCTGGCAAATGTGAA
  GGGCAAGAATGAATGCCCAAGGTGGGCAGCAGGTGAGGAAAGAGTCCCTCACCTCCCTGG
  AGGAACAAGTCTTTGATTTGCTGAAAGGCATCCCGCACAGCCTGGGCGCACTTGGGACTC
  TGGCCATAAAAGTCCTGGAGAAGAGACCAAGGTTGCTGCTGCCATTCTTGCACTGGCCTG
  GGGTACCCAAGTCCCCTCACTCACCGCTGTGACATCTCGGAAGAATTGGTAGGAGTCCCC
  AAGGCCTGCAACAGCTACAACAGGAGCGCTGGCTGCCAGTGCCCCAGCCACCAGGTGGGG
  GTACTTCATCCTCATGTAGGCACTCAGCATCCCCCCATAACTGGGAGTACAGAGCACAGA
  TCATGGTTGTGGGAAGCTGCCCACAACTCAGGCGAGCAGCCTCACTGTCCTCCAGGCTGA
  GGTGCTAGGCTGCTCTTTCCCTGCTCAGAACGCCCAAGGGTGGGAAAGAAGGACCTGAAA
  CTGTCAGGCCCACACACCCTGATCCCAGGGCCAAGGCAGATACAGCCTTCACTGGGAGAA
  GGCACCTGTGGGTGCCCTGCCCTGACCCAGCAATGAAGACATTGCAGAGACAAAGTCAGA
  AGGAATTGTCCCACTAGTGGGAACAACATAGCATACACTGCCTATGAGGTCCACTCAAGG
  AGGGCTTCCAGAAGGAGGTAAAGCTAGACCCCGCCCTTCCACATGTGGGGTAGGCATAGG
  ATGTTGAGACTGTAAGAGACATCTCTTTGGCCCTCCTTGTATAG
  ============================================================

Primers for ID: 2
Primer pair 1, product size : 1143
  Primer left                            Primer right
  start: 25209909                        start: 25211030
  end  : 25209929                        end  : 25211051
  tm   : 57                              tm   : 61
  seq  : GATGGCATGAAAGCAGATAGC           seq  : AGATCTGGACCCATGAGAGAAC

  >2_primer_pair_1_product
  GATGGCATGAAAGCAGATAGCTACAAGGCTCTTGGACCAGGCTAGCACTGGGTCCTGCAC
  CCAGGGAGAGCCACCTCACCTTGACAGGGTTGGCAGGAAGGGGCCCTAGAAAGTCAGTAG
  GATACGGGTAGTCCATCATGGCGAGCACAGTAAATGCATTTCGGGCAAACCCAAAGAGCT
  GAGTCAGGTCCTTTGGGCTGGAAAGTGATTGACAGGTACCAAAGTTCTGGCTGATGGTGT
  CATAGGCTGGGAAGAGAGAGGCCAGGAGAAAAGGCTGAGGAAACTGCTGGCAAATGTGAA
  GGGCAAGAATGAATGCCCAAGGTGGGCAGCAGGTGAGGAAAGAGTCCCTCACCTCCCTGG
  AGGAACAAGTCTTTGATTTGCTGAAAGGCATCCCGCACAGCCTGGGCGCACTTGGGACTC
  TGGCCATAAAAGTCCTGGAGAAGAGACCAAGGTTGCTGCTGCCATTCTTGCACTGGCCTG
  GGGTACCCAAGTCCCCTCACTCACCGCTGTGACATCTCGGAAGAATTGGTAGGAGTCCCC
  AAGGCCTGCAACAGCTACAACAGGAGCGCTGGCTGCCAGTGCCCCAGCCACCAGGTGGGG
  GTACTTCATCCTCATGTAGGCACTCAGCATCCCCCCATAACTGGGAGTACAGAGCACAGA
  TCATGGTTGTGGGAAGCTGCCCACAACTCAGGCGAGCAGCCTCACTGTCCTCCAGGCTGA
  GGTGCTAGGCTGCTCTTTCCCTGCTCAGAACGCCCAAGGGTGGGAAAGAAGGACCTGAAA
  CTGTCAGGCCCACACACCCTGATCCCAGGGCCAAGGCAGATACAGCCTTCACTGGGAGAA
  GGCACCTGTGGGTGCCCTGCCCTGACCCAGCAATGAAGACATTGCAGAGACAAAGTCAGA
  AGGAATTGTCCCACTAGTGGGAACAACATAGCATACACTGCCTATGAGGTCCACTCAAGG
  AGGGCTTCCAGAAGGAGGTAAAGCTAGACCCCGCCCTTCCACATGTGGGGTAGGCATAGG
  ATGTTGAGACTGTAAGAGACATCTCTTTGGCCCTCCTTGTATAGGGTGTCAATCGGCACA
  ACAGGGTGGAGCCTTAGAGTAGGGTAAGATTAGGACTCTAGGTTCTCTCATGGGTCCAGA
  TCT
  --------------------

Primer pair 2, product size : 1063
  Primer left                            Primer right
  start: 25209909                        start: 25210951
  end  : 25209929                        end  : 25210971
  tm   : 57                              tm   : 57
  seq  : GATGGCATGAAAGCAGATAGC           seq  : TATACAAGGAGGGCCAAAGAG

  >2_primer_pair_2_product
  GATGGCATGAAAGCAGATAGCTACAAGGCTCTTGGACCAGGCTAGCACTGGGTCCTGCAC
  CCAGGGAGAGCCACCTCACCTTGACAGGGTTGGCAGGAAGGGGCCCTAGAAAGTCAGTAG
  GATACGGGTAGTCCATCATGGCGAGCACAGTAAATGCATTTCGGGCAAACCCAAAGAGCT
  GAGTCAGGTCCTTTGGGCTGGAAAGTGATTGACAGGTACCAAAGTTCTGGCTGATGGTGT
  CATAGGCTGGGAAGAGAGAGGCCAGGAGAAAAGGCTGAGGAAACTGCTGGCAAATGTGAA
  GGGCAAGAATGAATGCCCAAGGTGGGCAGCAGGTGAGGAAAGAGTCCCTCACCTCCCTGG
  AGGAACAAGTCTTTGATTTGCTGAAAGGCATCCCGCACAGCCTGGGCGCACTTGGGACTC
  TGGCCATAAAAGTCCTGGAGAAGAGACCAAGGTTGCTGCTGCCATTCTTGCACTGGCCTG
  GGGTACCCAAGTCCCCTCACTCACCGCTGTGACATCTCGGAAGAATTGGTAGGAGTCCCC
  AAGGCCTGCAACAGCTACAACAGGAGCGCTGGCTGCCAGTGCCCCAGCCACCAGGTGGGG
  GTACTTCATCCTCATGTAGGCACTCAGCATCCCCCCATAACTGGGAGTACAGAGCACAGA
  TCATGGTTGTGGGAAGCTGCCCACAACTCAGGCGAGCAGCCTCACTGTCCTCCAGGCTGA
  GGTGCTAGGCTGCTCTTTCCCTGCTCAGAACGCCCAAGGGTGGGAAAGAAGGACCTGAAA
  CTGTCAGGCCCACACACCCTGATCCCAGGGCCAAGGCAGATACAGCCTTCACTGGGAGAA
  GGCACCTGTGGGTGCCCTGCCCTGACCCAGCAATGAAGACATTGCAGAGACAAAGTCAGA
  AGGAATTGTCCCACTAGTGGGAACAACATAGCATACACTGCCTATGAGGTCCACTCAAGG
  AGGGCTTCCAGAAGGAGGTAAAGCTAGACCCCGCCCTTCCACATGTGGGGTAGGCATAGG
  ATGTTGAGACTGTAAGAGACATCTCTTTGGCCCTCCTTGTATA
  ============================================================

Primers for ID: 12
Primer pair 1, product size : 1055
  Primer left                            Primer right
  start: 25211383                        start: 25212417
  end  : 25211403                        end  : 25212437
  tm   : 57                              tm   : 57
  seq  : ATGAAGCCAGAGTTGTTAGCG           seq  : AGATTCCCAGAAAGTCCAAGG

  >12_primer_pair_1_product
  ATGAAGCCAGAGTTGTTAGCGAAGGACCAGATATCCCCCTCATTCCCTGTGTAGAAAAAG
  ATGGGCCCTTCGCCCATCTTCCAGAACTTATCTGTTGGAAGTAAATGAGTTTCCATAAGG
  CCAGGGAAACGCAGGTAGGAACCCATGCGGTCGAGCCAGCACTCACCTGACACTAGGAAC
  CGCTGGCCAAAGGTTTTGTTGCCGAAACTCTCAAAGTTGAAATGGTCCATGTATTGCTCA
  AAATAATTCTCATGAAAGTCAGGGTCTAGAACTCTGTCGGCTGAGGGCAGGTGCAGAGAC
  TCAGGAGCTGGTTGGGATCATCAGGGATCTAGGCGGGTCAGGAGGAAGGGCAGCCAGTCT
  GTACTCACCTCTGGCCTGGAGGTTGCACAGTCCCAGTGACAGCAGCAGGACCAGGATCCA
  GGAGGGGACACCATGGTCCACAGGGTAACAAGGATGGAAGTTCATGCTTGATTCTGAGCC
  GGGCGCTGACTGTCATGTGATTTGGTCACATGACCGACACAACGGGCGGGGCAGCATCAC
  GTGATAGTCTGGCGGGGGCTGTCCTACTGTGGCTGGATTCTAGTTGGAGGATCAGCCTAC
  TCTTCTTCAGTTTCCCGGTTCCTCCAAATTTCTGGGCTCCTACTTGTTTCCACAGAGATG
  GATACTGTGGAGGTCCAGGAAGCAGAGAGATGGCTAAGGCTCATCAGGACCGTATGATCT
  CCCAAGTGTCCAGCTACTGAGTACCACAAGGTGATGGGTGGGAGGGTCCTCCCACGGAAG
  GATACCGCAGTCCCTAGGGGTTGCAAGCCCCACATGTTCCACTGGCTGCTAGAGCTACCT
  ACTCAATCAGCCCTGGGCATCACCATCAGGTACTCGGCCAAAATGACCTCTCTGCTTCCA
  GTCCTCAGTTCTGGTCAGCACCAGACAGGCCCATAATTACAGAGCCAGGGAAACTGGAAC
  ATTTGTCTCCCCTTAGACAGTGGCAGCAGGAAGGTGGGGGGTTGTTGCAGAGGAACAGTG
  TCTCTGAGAGAGGACCTTGGACTTTCTGGGAATCT
  --------------------

Primer pair 2, product size : 1053
  Primer left                            Primer right
  start: 25211383                        start: 25212415
  end  : 25211403                        end  : 25212435
  tm   : 57                              tm   : 57
  seq  : ATGAAGCCAGAGTTGTTAGCG           seq  : ATTCCCAGAAAGTCCAAGGTC

  >12_primer_pair_2_product
  ATGAAGCCAGAGTTGTTAGCGAAGGACCAGATATCCCCCTCATTCCCTGTGTAGAAAAAG
  ATGGGCCCTTCGCCCATCTTCCAGAACTTATCTGTTGGAAGTAAATGAGTTTCCATAAGG
  CCAGGGAAACGCAGGTAGGAACCCATGCGGTCGAGCCAGCACTCACCTGACACTAGGAAC
  CGCTGGCCAAAGGTTTTGTTGCCGAAACTCTCAAAGTTGAAATGGTCCATGTATTGCTCA
  AAATAATTCTCATGAAAGTCAGGGTCTAGAACTCTGTCGGCTGAGGGCAGGTGCAGAGAC
  TCAGGAGCTGGTTGGGATCATCAGGGATCTAGGCGGGTCAGGAGGAAGGGCAGCCAGTCT
  GTACTCACCTCTGGCCTGGAGGTTGCACAGTCCCAGTGACAGCAGCAGGACCAGGATCCA
  GGAGGGGACACCATGGTCCACAGGGTAACAAGGATGGAAGTTCATGCTTGATTCTGAGCC
  GGGCGCTGACTGTCATGTGATTTGGTCACATGACCGACACAACGGGCGGGGCAGCATCAC
  GTGATAGTCTGGCGGGGGCTGTCCTACTGTGGCTGGATTCTAGTTGGAGGATCAGCCTAC
  TCTTCTTCAGTTTCCCGGTTCCTCCAAATTTCTGGGCTCCTACTTGTTTCCACAGAGATG
  GATACTGTGGAGGTCCAGGAAGCAGAGAGATGGCTAAGGCTCATCAGGACCGTATGATCT
  CCCAAGTGTCCAGCTACTGAGTACCACAAGGTGATGGGTGGGAGGGTCCTCCCACGGAAG
  GATACCGCAGTCCCTAGGGGTTGCAAGCCCCACATGTTCCACTGGCTGCTAGAGCTACCT
  ACTCAATCAGCCCTGGGCATCACCATCAGGTACTCGGCCAAAATGACCTCTCTGCTTCCA
  GTCCTCAGTTCTGGTCAGCACCAGACAGGCCCATAATTACAGAGCCAGGGAAACTGGAAC
  ATTTGTCTCCCCTTAGACAGTGGCAGCAGGAAGGTGGGGGGTTGTTGCAGAGGAACAGTG
  TCTCTGAGAGAGGACCTTGGACTTTCTGGGAAT
  ============================================================

Primers for ID: 13
Primer pair 1, product size : 1055
  Primer left                            Primer right
  start: 25211383                        start: 25212417
  end  : 25211403                        end  : 25212437
  tm   : 57                              tm   : 57
  seq  : ATGAAGCCAGAGTTGTTAGCG           seq  : AGATTCCCAGAAAGTCCAAGG

  >13_primer_pair_1_product
  ATGAAGCCAGAGTTGTTAGCGAAGGACCAGATATCCCCCTCATTCCCTGTGTAGAAAAAG
  ATGGGCCCTTCGCCCATCTTCCAGAACTTATCTGTTGGAAGTAAATGAGTTTCCATAAGG
  CCAGGGAAACGCAGGTAGGAACCCATGCGGTCGAGCCAGCACTCACCTGACACTAGGAAC
  CGCTGGCCAAAGGTTTTGTTGCCGAAACTCTCAAAGTTGAAATGGTCCATGTATTGCTCA
  AAATAATTCTCATGAAAGTCAGGGTCTAGAACTCTGTCGGCTGAGGGCAGGTGCAGAGAC
  TCAGGAGCTGGTTGGGATCATCAGGGATCTAGGCGGGTCAGGAGGAAGGGCAGCCAGTCT
  GTACTCACCTCTGGCCTGGAGGTTGCACAGTCCCAGTGACAGCAGCAGGACCAGGATCCA
  GGAGGGGACACCATGGTCCACAGGGTAACAAGGATGGAAGTTCATGCTTGATTCTGAGCC
  GGGCGCTGACTGTCATGTGATTTGGTCACATGACCGACACAACGGGCGGGGCAGCATCAC
  GTGATAGTCTGGCGGGGGCTGTCCTACTGTGGCTGGATTCTAGTTGGAGGATCAGCCTAC
  TCTTCTTCAGTTTCCCGGTTCCTCCAAATTTCTGGGCTCCTACTTGTTTCCACAGAGATG
  GATACTGTGGAGGTCCAGGAAGCAGAGAGATGGCTAAGGCTCATCAGGACCGTATGATCT
  CCCAAGTGTCCAGCTACTGAGTACCACAAGGTGATGGGTGGGAGGGTCCTCCCACGGAAG
  GATACCGCAGTCCCTAGGGGTTGCAAGCCCCACATGTTCCACTGGCTGCTAGAGCTACCT
  ACTCAATCAGCCCTGGGCATCACCATCAGGTACTCGGCCAAAATGACCTCTCTGCTTCCA
  GTCCTCAGTTCTGGTCAGCACCAGACAGGCCCATAATTACAGAGCCAGGGAAACTGGAAC
  ATTTGTCTCCCCTTAGACAGTGGCAGCAGGAAGGTGGGGGGTTGTTGCAGAGGAACAGTG
  TCTCTGAGAGAGGACCTTGGACTTTCTGGGAATCT
  --------------------

Primer pair 2, product size : 1053
  Primer left                            Primer right
  start: 25211383                        start: 25212415
  end  : 25211403                        end  : 25212435
  tm   : 57                              tm   : 57
  seq  : ATGAAGCCAGAGTTGTTAGCG           seq  : ATTCCCAGAAAGTCCAAGGTC

  >13_primer_pair_2_product
  ATGAAGCCAGAGTTGTTAGCGAAGGACCAGATATCCCCCTCATTCCCTGTGTAGAAAAAG
  ATGGGCCCTTCGCCCATCTTCCAGAACTTATCTGTTGGAAGTAAATGAGTTTCCATAAGG
  CCAGGGAAACGCAGGTAGGAACCCATGCGGTCGAGCCAGCACTCACCTGACACTAGGAAC
  CGCTGGCCAAAGGTTTTGTTGCCGAAACTCTCAAAGTTGAAATGGTCCATGTATTGCTCA
  AAATAATTCTCATGAAAGTCAGGGTCTAGAACTCTGTCGGCTGAGGGCAGGTGCAGAGAC
  TCAGGAGCTGGTTGGGATCATCAGGGATCTAGGCGGGTCAGGAGGAAGGGCAGCCAGTCT
  GTACTCACCTCTGGCCTGGAGGTTGCACAGTCCCAGTGACAGCAGCAGGACCAGGATCCA
  GGAGGGGACACCATGGTCCACAGGGTAACAAGGATGGAAGTTCATGCTTGATTCTGAGCC
  GGGCGCTGACTGTCATGTGATTTGGTCACATGACCGACACAACGGGCGGGGCAGCATCAC
  GTGATAGTCTGGCGGGGGCTGTCCTACTGTGGCTGGATTCTAGTTGGAGGATCAGCCTAC
  TCTTCTTCAGTTTCCCGGTTCCTCCAAATTTCTGGGCTCCTACTTGTTTCCACAGAGATG
  GATACTGTGGAGGTCCAGGAAGCAGAGAGATGGCTAAGGCTCATCAGGACCGTATGATCT
  CCCAAGTGTCCAGCTACTGAGTACCACAAGGTGATGGGTGGGAGGGTCCTCCCACGGAAG
  GATACCGCAGTCCCTAGGGGTTGCAAGCCCCACATGTTCCACTGGCTGCTAGAGCTACCT
  ACTCAATCAGCCCTGGGCATCACCATCAGGTACTCGGCCAAAATGACCTCTCTGCTTCCA
  GTCCTCAGTTCTGGTCAGCACCAGACAGGCCCATAATTACAGAGCCAGGGAAACTGGAAC
  ATTTGTCTCCCCTTAGACAGTGGCAGCAGGAAGGTGGGGGGTTGTTGCAGAGGAACAGTG
  TCTCTGAGAGAGGACCTTGGACTTTCTGGGAAT
  ============================================================

Primers for ID: 14
Primer pair 1, product size : 1010
  Primer left                            Primer right
  start: 25211428                        start: 25212417
  end  : 25211448                        end  : 25212437
  tm   : 57                              tm   : 57
  seq  : CCTGTGTAGAAAAAGATGGGC           seq  : AGATTCCCAGAAAGTCCAAGG

  >14_primer_pair_1_product
  CCTGTGTAGAAAAAGATGGGCCCTTCGCCCATCTTCCAGAACTTATCTGTTGGAAGTAAA
  TGAGTTTCCATAAGGCCAGGGAAACGCAGGTAGGAACCCATGCGGTCGAGCCAGCACTCA
  CCTGACACTAGGAACCGCTGGCCAAAGGTTTTGTTGCCGAAACTCTCAAAGTTGAAATGG
  TCCATGTATTGCTCAAAATAATTCTCATGAAAGTCAGGGTCTAGAACTCTGTCGGCTGAG
  GGCAGGTGCAGAGACTCAGGAGCTGGTTGGGATCATCAGGGATCTAGGCGGGTCAGGAGG
  AAGGGCAGCCAGTCTGTACTCACCTCTGGCCTGGAGGTTGCACAGTCCCAGTGACAGCAG
  CAGGACCAGGATCCAGGAGGGGACACCATGGTCCACAGGGTAACAAGGATGGAAGTTCAT
  GCTTGATTCTGAGCCGGGCGCTGACTGTCATGTGATTTGGTCACATGACCGACACAACGG
  GCGGGGCAGCATCACGTGATAGTCTGGCGGGGGCTGTCCTACTGTGGCTGGATTCTAGTT
  GGAGGATCAGCCTACTCTTCTTCAGTTTCCCGGTTCCTCCAAATTTCTGGGCTCCTACTT
  GTTTCCACAGAGATGGATACTGTGGAGGTCCAGGAAGCAGAGAGATGGCTAAGGCTCATC
  AGGACCGTATGATCTCCCAAGTGTCCAGCTACTGAGTACCACAAGGTGATGGGTGGGAGG
  GTCCTCCCACGGAAGGATACCGCAGTCCCTAGGGGTTGCAAGCCCCACATGTTCCACTGG
  CTGCTAGAGCTACCTACTCAATCAGCCCTGGGCATCACCATCAGGTACTCGGCCAAAATG
  ACCTCTCTGCTTCCAGTCCTCAGTTCTGGTCAGCACCAGACAGGCCCATAATTACAGAGC
  CAGGGAAACTGGAACATTTGTCTCCCCTTAGACAGTGGCAGCAGGAAGGTGGGGGGTTGT
  TGCAGAGGAACAGTGTCTCTGAGAGAGGACCTTGGACTTTCTGGGAATCT
  --------------------

Primer pair 2, product size : 1008
  Primer left                            Primer right
  start: 25211428                        start: 25212415
  end  : 25211448                        end  : 25212435
  tm   : 57                              tm   : 57
  seq  : CCTGTGTAGAAAAAGATGGGC           seq  : ATTCCCAGAAAGTCCAAGGTC

  >14_primer_pair_2_product
  CCTGTGTAGAAAAAGATGGGCCCTTCGCCCATCTTCCAGAACTTATCTGTTGGAAGTAAA
  TGAGTTTCCATAAGGCCAGGGAAACGCAGGTAGGAACCCATGCGGTCGAGCCAGCACTCA
  CCTGACACTAGGAACCGCTGGCCAAAGGTTTTGTTGCCGAAACTCTCAAAGTTGAAATGG
  TCCATGTATTGCTCAAAATAATTCTCATGAAAGTCAGGGTCTAGAACTCTGTCGGCTGAG
  GGCAGGTGCAGAGACTCAGGAGCTGGTTGGGATCATCAGGGATCTAGGCGGGTCAGGAGG
  AAGGGCAGCCAGTCTGTACTCACCTCTGGCCTGGAGGTTGCACAGTCCCAGTGACAGCAG
  CAGGACCAGGATCCAGGAGGGGACACCATGGTCCACAGGGTAACAAGGATGGAAGTTCAT
  GCTTGATTCTGAGCCGGGCGCTGACTGTCATGTGATTTGGTCACATGACCGACACAACGG
  GCGGGGCAGCATCACGTGATAGTCTGGCGGGGGCTGTCCTACTGTGGCTGGATTCTAGTT
  GGAGGATCAGCCTACTCTTCTTCAGTTTCCCGGTTCCTCCAAATTTCTGGGCTCCTACTT
  GTTTCCACAGAGATGGATACTGTGGAGGTCCAGGAAGCAGAGAGATGGCTAAGGCTCATC
  AGGACCGTATGATCTCCCAAGTGTCCAGCTACTGAGTACCACAAGGTGATGGGTGGGAGG
  GTCCTCCCACGGAAGGATACCGCAGTCCCTAGGGGTTGCAAGCCCCACATGTTCCACTGG
  CTGCTAGAGCTACCTACTCAATCAGCCCTGGGCATCACCATCAGGTACTCGGCCAAAATG
  ACCTCTCTGCTTCCAGTCCTCAGTTCTGGTCAGCACCAGACAGGCCCATAATTACAGAGC
  CAGGGAAACTGGAACATTTGTCTCCCCTTAGACAGTGGCAGCAGGAAGGTGGGGGGTTGT
  TGCAGAGGAACAGTGTCTCTGAGAGAGGACCTTGGACTTTCTGGGAAT
  ============================================================

Primers for ID: 15
Primer pair 1, product size : 1063
  Primer left                            Primer right
  start: 25209909                        start: 25210951
  end  : 25209929                        end  : 25210971
  tm   : 57                              tm   : 57
  seq  : GATGGCATGAAAGCAGATAGC           seq  : TATACAAGGAGGGCCAAAGAG

  >15_primer_pair_1_product
  GATGGCATGAAAGCAGATAGCTACAAGGCTCTTGGACCAGGCTAGCACTGGGTCCTGCAC
  CCAGGGAGAGCCACCTCACCTTGACAGGGTTGGCAGGAAGGGGCCCTAGAAAGTCAGTAG
  GATACGGGTAGTCCATCATGGCGAGCACAGTAAATGCATTTCGGGCAAACCCAAAGAGCT
  GAGTCAGGTCCTTTGGGCTGGAAAGTGATTGACAGGTACCAAAGTTCTGGCTGATGGTGT
  CATAGGCTGGGAAGAGAGAGGCCAGGAGAAAAGGCTGAGGAAACTGCTGGCAAATGTGAA
  GGGCAAGAATGAATGCCCAAGGTGGGCAGCAGGTGAGGAAAGAGTCCCTCACCTCCCTGG
  AGGAACAAGTCTTTGATTTGCTGAAAGGCATCCCGCACAGCCTGGGCGCACTTGGGACTC
  TGGCCATAAAAGTCCTGGAGAAGAGACCAAGGTTGCTGCTGCCATTCTTGCACTGGCCTG
  GGGTACCCAAGTCCCCTCACTCACCGCTGTGACATCTCGGAAGAATTGGTAGGAGTCCCC
  AAGGCCTGCAACAGCTACAACAGGAGCGCTGGCTGCCAGTGCCCCAGCCACCAGGTGGGG
  GTACTTCATCCTCATGTAGGCACTCAGCATCCCCCCATAACTGGGAGTACAGAGCACAGA
  TCATGGTTGTGGGAAGCTGCCCACAACTCAGGCGAGCAGCCTCACTGTCCTCCAGGCTGA
  GGTGCTAGGCTGCTCTTTCCCTGCTCAGAACGCCCAAGGGTGGGAAAGAAGGACCTGAAA
  CTGTCAGGCCCACACACCCTGATCCCAGGGCCAAGGCAGATACAGCCTTCACTGGGAGAA
  GGCACCTGTGGGTGCCCTGCCCTGACCCAGCAATGAAGACATTGCAGAGACAAAGTCAGA
  AGGAATTGTCCCACTAGTGGGAACAACATAGCATACACTGCCTATGAGGTCCACTCAAGG
  AGGGCTTCCAGAAGGAGGTAAAGCTAGACCCCGCCCTTCCACATGTGGGGTAGGCATAGG
  ATGTTGAGACTGTAAGAGACATCTCTTTGGCCCTCCTTGTATA
  --------------------

Primer pair 2, product size : 1064
  Primer left                            Primer right
  start: 25209909                        start: 25210951
  end  : 25209929                        end  : 25210972
  tm   : 57                              tm   : 61
  seq  : GATGGCATGAAAGCAGATAGC           seq  : CTATACAAGGAGGGCCAAAGAG

  >15_primer_pair_2_product
  GATGGCATGAAAGCAGATAGCTACAAGGCTCTTGGACCAGGCTAGCACTGGGTCCTGCAC
  CCAGGGAGAGCCACCTCACCTTGACAGGGTTGGCAGGAAGGGGCCCTAGAAAGTCAGTAG
  GATACGGGTAGTCCATCATGGCGAGCACAGTAAATGCATTTCGGGCAAACCCAAAGAGCT
  GAGTCAGGTCCTTTGGGCTGGAAAGTGATTGACAGGTACCAAAGTTCTGGCTGATGGTGT
  CATAGGCTGGGAAGAGAGAGGCCAGGAGAAAAGGCTGAGGAAACTGCTGGCAAATGTGAA
  GGGCAAGAATGAATGCCCAAGGTGGGCAGCAGGTGAGGAAAGAGTCCCTCACCTCCCTGG
  AGGAACAAGTCTTTGATTTGCTGAAAGGCATCCCGCACAGCCTGGGCGCACTTGGGACTC
  TGGCCATAAAAGTCCTGGAGAAGAGACCAAGGTTGCTGCTGCCATTCTTGCACTGGCCTG
  GGGTACCCAAGTCCCCTCACTCACCGCTGTGACATCTCGGAAGAATTGGTAGGAGTCCCC
  AAGGCCTGCAACAGCTACAACAGGAGCGCTGGCTGCCAGTGCCCCAGCCACCAGGTGGGG
  GTACTTCATCCTCATGTAGGCACTCAGCATCCCCCCATAACTGGGAGTACAGAGCACAGA
  TCATGGTTGTGGGAAGCTGCCCACAACTCAGGCGAGCAGCCTCACTGTCCTCCAGGCTGA
  GGTGCTAGGCTGCTCTTTCCCTGCTCAGAACGCCCAAGGGTGGGAAAGAAGGACCTGAAA
  CTGTCAGGCCCACACACCCTGATCCCAGGGCCAAGGCAGATACAGCCTTCACTGGGAGAA
  GGCACCTGTGGGTGCCCTGCCCTGACCCAGCAATGAAGACATTGCAGAGACAAAGTCAGA
  AGGAATTGTCCCACTAGTGGGAACAACATAGCATACACTGCCTATGAGGTCCACTCAAGG
  AGGGCTTCCAGAAGGAGGTAAAGCTAGACCCCGCCCTTCCACATGTGGGGTAGGCATAGG
  ATGTTGAGACTGTAAGAGACATCTCTTTGGCCCTCCTTGTATAG
  ============================================================

Primers for ID: 16
Primer pair 1, product size : 1143
  Primer left                            Primer right
  start: 25209909                        start: 25211030
  end  : 25209929                        end  : 25211051
  tm   : 57                              tm   : 61
  seq  : GATGGCATGAAAGCAGATAGC           seq  : AGATCTGGACCCATGAGAGAAC

  >16_primer_pair_1_product
  GATGGCATGAAAGCAGATAGCTACAAGGCTCTTGGACCAGGCTAGCACTGGGTCCTGCAC
  CCAGGGAGAGCCACCTCACCTTGACAGGGTTGGCAGGAAGGGGCCCTAGAAAGTCAGTAG
  GATACGGGTAGTCCATCATGGCGAGCACAGTAAATGCATTTCGGGCAAACCCAAAGAGCT
  GAGTCAGGTCCTTTGGGCTGGAAAGTGATTGACAGGTACCAAAGTTCTGGCTGATGGTGT
  CATAGGCTGGGAAGAGAGAGGCCAGGAGAAAAGGCTGAGGAAACTGCTGGCAAATGTGAA
  GGGCAAGAATGAATGCCCAAGGTGGGCAGCAGGTGAGGAAAGAGTCCCTCACCTCCCTGG
  AGGAACAAGTCTTTGATTTGCTGAAAGGCATCCCGCACAGCCTGGGCGCACTTGGGACTC
  TGGCCATAAAAGTCCTGGAGAAGAGACCAAGGTTGCTGCTGCCATTCTTGCACTGGCCTG
  GGGTACCCAAGTCCCCTCACTCACCGCTGTGACATCTCGGAAGAATTGGTAGGAGTCCCC
  AAGGCCTGCAACAGCTACAACAGGAGCGCTGGCTGCCAGTGCCCCAGCCACCAGGTGGGG
  GTACTTCATCCTCATGTAGGCACTCAGCATCCCCCCATAACTGGGAGTACAGAGCACAGA
  TCATGGTTGTGGGAAGCTGCCCACAACTCAGGCGAGCAGCCTCACTGTCCTCCAGGCTGA
  GGTGCTAGGCTGCTCTTTCCCTGCTCAGAACGCCCAAGGGTGGGAAAGAAGGACCTGAAA
  CTGTCAGGCCCACACACCCTGATCCCAGGGCCAAGGCAGATACAGCCTTCACTGGGAGAA
  GGCACCTGTGGGTGCCCTGCCCTGACCCAGCAATGAAGACATTGCAGAGACAAAGTCAGA
  AGGAATTGTCCCACTAGTGGGAACAACATAGCATACACTGCCTATGAGGTCCACTCAAGG
  AGGGCTTCCAGAAGGAGGTAAAGCTAGACCCCGCCCTTCCACATGTGGGGTAGGCATAGG
  ATGTTGAGACTGTAAGAGACATCTCTTTGGCCCTCCTTGTATAGGGTGTCAATCGGCACA
  ACAGGGTGGAGCCTTAGAGTAGGGTAAGATTAGGACTCTAGGTTCTCTCATGGGTCCAGA
  TCT
  --------------------

Primer pair 2, product size : 1063
  Primer left                            Primer right
  start: 25209909                        start: 25210951
  end  : 25209929                        end  : 25210971
  tm   : 57                              tm   : 57
  seq  : GATGGCATGAAAGCAGATAGC           seq  : TATACAAGGAGGGCCAAAGAG

  >16_primer_pair_2_product
  GATGGCATGAAAGCAGATAGCTACAAGGCTCTTGGACCAGGCTAGCACTGGGTCCTGCAC
  CCAGGGAGAGCCACCTCACCTTGACAGGGTTGGCAGGAAGGGGCCCTAGAAAGTCAGTAG
  GATACGGGTAGTCCATCATGGCGAGCACAGTAAATGCATTTCGGGCAAACCCAAAGAGCT
  GAGTCAGGTCCTTTGGGCTGGAAAGTGATTGACAGGTACCAAAGTTCTGGCTGATGGTGT
  CATAGGCTGGGAAGAGAGAGGCCAGGAGAAAAGGCTGAGGAAACTGCTGGCAAATGTGAA
  GGGCAAGAATGAATGCCCAAGGTGGGCAGCAGGTGAGGAAAGAGTCCCTCACCTCCCTGG
  AGGAACAAGTCTTTGATTTGCTGAAAGGCATCCCGCACAGCCTGGGCGCACTTGGGACTC
  TGGCCATAAAAGTCCTGGAGAAGAGACCAAGGTTGCTGCTGCCATTCTTGCACTGGCCTG
  GGGTACCCAAGTCCCCTCACTCACCGCTGTGACATCTCGGAAGAATTGGTAGGAGTCCCC
  AAGGCCTGCAACAGCTACAACAGGAGCGCTGGCTGCCAGTGCCCCAGCCACCAGGTGGGG
  GTACTTCATCCTCATGTAGGCACTCAGCATCCCCCCATAACTGGGAGTACAGAGCACAGA
  TCATGGTTGTGGGAAGCTGCCCACAACTCAGGCGAGCAGCCTCACTGTCCTCCAGGCTGA
  GGTGCTAGGCTGCTCTTTCCCTGCTCAGAACGCCCAAGGGTGGGAAAGAAGGACCTGAAA
  CTGTCAGGCCCACACACCCTGATCCCAGGGCCAAGGCAGATACAGCCTTCACTGGGAGAA
  GGCACCTGTGGGTGCCCTGCCCTGACCCAGCAATGAAGACATTGCAGAGACAAAGTCAGA
  AGGAATTGTCCCACTAGTGGGAACAACATAGCATACACTGCCTATGAGGTCCACTCAAGG
  AGGGCTTCCAGAAGGAGGTAAAGCTAGACCCCGCCCTTCCACATGTGGGGTAGGCATAGG
  ATGTTGAGACTGTAAGAGACATCTCTTTGGCCCTCCTTGTATA
  ============================================================

Primers for ID: 27
Primer pair 1, product size : 1055
  Primer left                            Primer right
  start: 25211383                        start: 25212417
  end  : 25211403                        end  : 25212437
  tm   : 57                              tm   : 57
  seq  : ATGAAGCCAGAGTTGTTAGCG           seq  : AGATTCCCAGAAAGTCCAAGG

  >27_primer_pair_1_product
  ATGAAGCCAGAGTTGTTAGCGAAGGACCAGATATCCCCCTCATTCCCTGTGTAGAAAAAG
  ATGGGCCCTTCGCCCATCTTCCAGAACTTATCTGTTGGAAGTAAATGAGTTTCCATAAGG
  CCAGGGAAACGCAGGTAGGAACCCATGCGGTCGAGCCAGCACTCACCTGACACTAGGAAC
  CGCTGGCCAAAGGTTTTGTTGCCGAAACTCTCAAAGTTGAAATGGTCCATGTATTGCTCA
  AAATAATTCTCATGAAAGTCAGGGTCTAGAACTCTGTCGGCTGAGGGCAGGTGCAGAGAC
  TCAGGAGCTGGTTGGGATCATCAGGGATCTAGGCGGGTCAGGAGGAAGGGCAGCCAGTCT
  GTACTCACCTCTGGCCTGGAGGTTGCACAGTCCCAGTGACAGCAGCAGGACCAGGATCCA
  GGAGGGGACACCATGGTCCACAGGGTAACAAGGATGGAAGTTCATGCTTGATTCTGAGCC
  GGGCGCTGACTGTCATGTGATTTGGTCACATGACCGACACAACGGGCGGGGCAGCATCAC
  GTGATAGTCTGGCGGGGGCTGTCCTACTGTGGCTGGATTCTAGTTGGAGGATCAGCCTAC
  TCTTCTTCAGTTTCCCGGTTCCTCCAAATTTCTGGGCTCCTACTTGTTTCCACAGAGATG
  GATACTGTGGAGGTCCAGGAAGCAGAGAGATGGCTAAGGCTCATCAGGACCGTATGATCT
  CCCAAGTGTCCAGCTACTGAGTACCACAAGGTGATGGGTGGGAGGGTCCTCCCACGGAAG
  GATACCGCAGTCCCTAGGGGTTGCAAGCCCCACATGTTCCACTGGCTGCTAGAGCTACCT
  ACTCAATCAGCCCTGGGCATCACCATCAGGTACTCGGCCAAAATGACCTCTCTGCTTCCA
  GTCCTCAGTTCTGGTCAGCACCAGACAGGCCCATAATTACAGAGCCAGGGAAACTGGAAC
  ATTTGTCTCCCCTTAGACAGTGGCAGCAGGAAGGTGGGGGGTTGTTGCAGAGGAACAGTG
  TCTCTGAGAGAGGACCTTGGACTTTCTGGGAATCT
  --------------------

Primer pair 2, product size : 1053
  Primer left                            Primer right
  start: 25211383                        start: 25212415
  end  : 25211403                        end  : 25212435
  tm   : 57                              tm   : 57
  seq  : ATGAAGCCAGAGTTGTTAGCG           seq  : ATTCCCAGAAAGTCCAAGGTC

  >27_primer_pair_2_product
  ATGAAGCCAGAGTTGTTAGCGAAGGACCAGATATCCCCCTCATTCCCTGTGTAGAAAAAG
  ATGGGCCCTTCGCCCATCTTCCAGAACTTATCTGTTGGAAGTAAATGAGTTTCCATAAGG
  CCAGGGAAACGCAGGTAGGAACCCATGCGGTCGAGCCAGCACTCACCTGACACTAGGAAC
  CGCTGGCCAAAGGTTTTGTTGCCGAAACTCTCAAAGTTGAAATGGTCCATGTATTGCTCA
  AAATAATTCTCATGAAAGTCAGGGTCTAGAACTCTGTCGGCTGAGGGCAGGTGCAGAGAC
  TCAGGAGCTGGTTGGGATCATCAGGGATCTAGGCGGGTCAGGAGGAAGGGCAGCCAGTCT
  GTACTCACCTCTGGCCTGGAGGTTGCACAGTCCCAGTGACAGCAGCAGGACCAGGATCCA
  GGAGGGGACACCATGGTCCACAGGGTAACAAGGATGGAAGTTCATGCTTGATTCTGAGCC
  GGGCGCTGACTGTCATGTGATTTGGTCACATGACCGACACAACGGGCGGGGCAGCATCAC
  GTGATAGTCTGGCGGGGGCTGTCCTACTGTGGCTGGATTCTAGTTGGAGGATCAGCCTAC
  TCTTCTTCAGTTTCCCGGTTCCTCCAAATTTCTGGGCTCCTACTTGTTTCCACAGAGATG
  GATACTGTGGAGGTCCAGGAAGCAGAGAGATGGCTAAGGCTCATCAGGACCGTATGATCT
  CCCAAGTGTCCAGCTACTGAGTACCACAAGGTGATGGGTGGGAGGGTCCTCCCACGGAAG
  GATACCGCAGTCCCTAGGGGTTGCAAGCCCCACATGTTCCACTGGCTGCTAGAGCTACCT
  ACTCAATCAGCCCTGGGCATCACCATCAGGTACTCGGCCAAAATGACCTCTCTGCTTCCA
  GTCCTCAGTTCTGGTCAGCACCAGACAGGCCCATAATTACAGAGCCAGGGAAACTGGAAC
  ATTTGTCTCCCCTTAGACAGTGGCAGCAGGAAGGTGGGGGGTTGTTGCAGAGGAACAGTG
  TCTCTGAGAGAGGACCTTGGACTTTCTGGGAAT
  ============================================================

Primers for ID: 28
Primer pair 1, product size : 1055
  Primer left                            Primer right
  start: 25211383                        start: 25212417
  end  : 25211403                        end  : 25212437
  tm   : 57                              tm   : 57
  seq  : ATGAAGCCAGAGTTGTTAGCG           seq  : AGATTCCCAGAAAGTCCAAGG

  >28_primer_pair_1_product
  ATGAAGCCAGAGTTGTTAGCGAAGGACCAGATATCCCCCTCATTCCCTGTGTAGAAAAAG
  ATGGGCCCTTCGCCCATCTTCCAGAACTTATCTGTTGGAAGTAAATGAGTTTCCATAAGG
  CCAGGGAAACGCAGGTAGGAACCCATGCGGTCGAGCCAGCACTCACCTGACACTAGGAAC
  CGCTGGCCAAAGGTTTTGTTGCCGAAACTCTCAAAGTTGAAATGGTCCATGTATTGCTCA
  AAATAATTCTCATGAAAGTCAGGGTCTAGAACTCTGTCGGCTGAGGGCAGGTGCAGAGAC
  TCAGGAGCTGGTTGGGATCATCAGGGATCTAGGCGGGTCAGGAGGAAGGGCAGCCAGTCT
  GTACTCACCTCTGGCCTGGAGGTTGCACAGTCCCAGTGACAGCAGCAGGACCAGGATCCA
  GGAGGGGACACCATGGTCCACAGGGTAACAAGGATGGAAGTTCATGCTTGATTCTGAGCC
  GGGCGCTGACTGTCATGTGATTTGGTCACATGACCGACACAACGGGCGGGGCAGCATCAC
  GTGATAGTCTGGCGGGGGCTGTCCTACTGTGGCTGGATTCTAGTTGGAGGATCAGCCTAC
  TCTTCTTCAGTTTCCCGGTTCCTCCAAATTTCTGGGCTCCTACTTGTTTCCACAGAGATG
  GATACTGTGGAGGTCCAGGAAGCAGAGAGATGGCTAAGGCTCATCAGGACCGTATGATCT
  CCCAAGTGTCCAGCTACTGAGTACCACAAGGTGATGGGTGGGAGGGTCCTCCCACGGAAG
  GATACCGCAGTCCCTAGGGGTTGCAAGCCCCACATGTTCCACTGGCTGCTAGAGCTACCT
  ACTCAATCAGCCCTGGGCATCACCATCAGGTACTCGGCCAAAATGACCTCTCTGCTTCCA
  GTCCTCAGTTCTGGTCAGCACCAGACAGGCCCATAATTACAGAGCCAGGGAAACTGGAAC
  ATTTGTCTCCCCTTAGACAGTGGCAGCAGGAAGGTGGGGGGTTGTTGCAGAGGAACAGTG
  TCTCTGAGAGAGGACCTTGGACTTTCTGGGAATCT
  --------------------

Primer pair 2, product size : 1053
  Primer left                            Primer right
  start: 25211383                        start: 25212415
  end  : 25211403                        end  : 25212435
  tm   : 57                              tm   : 57
  seq  : ATGAAGCCAGAGTTGTTAGCG           seq  : ATTCCCAGAAAGTCCAAGGTC

  >28_primer_pair_2_product
  ATGAAGCCAGAGTTGTTAGCGAAGGACCAGATATCCCCCTCATTCCCTGTGTAGAAAAAG
  ATGGGCCCTTCGCCCATCTTCCAGAACTTATCTGTTGGAAGTAAATGAGTTTCCATAAGG
  CCAGGGAAACGCAGGTAGGAACCCATGCGGTCGAGCCAGCACTCACCTGACACTAGGAAC
  CGCTGGCCAAAGGTTTTGTTGCCGAAACTCTCAAAGTTGAAATGGTCCATGTATTGCTCA
  AAATAATTCTCATGAAAGTCAGGGTCTAGAACTCTGTCGGCTGAGGGCAGGTGCAGAGAC
  TCAGGAGCTGGTTGGGATCATCAGGGATCTAGGCGGGTCAGGAGGAAGGGCAGCCAGTCT
  GTACTCACCTCTGGCCTGGAGGTTGCACAGTCCCAGTGACAGCAGCAGGACCAGGATCCA
  GGAGGGGACACCATGGTCCACAGGGTAACAAGGATGGAAGTTCATGCTTGATTCTGAGCC
  GGGCGCTGACTGTCATGTGATTTGGTCACATGACCGACACAACGGGCGGGGCAGCATCAC
  GTGATAGTCTGGCGGGGGCTGTCCTACTGTGGCTGGATTCTAGTTGGAGGATCAGCCTAC
  TCTTCTTCAGTTTCCCGGTTCCTCCAAATTTCTGGGCTCCTACTTGTTTCCACAGAGATG
  GATACTGTGGAGGTCCAGGAAGCAGAGAGATGGCTAAGGCTCATCAGGACCGTATGATCT
  CCCAAGTGTCCAGCTACTGAGTACCACAAGGTGATGGGTGGGAGGGTCCTCCCACGGAAG
  GATACCGCAGTCCCTAGGGGTTGCAAGCCCCACATGTTCCACTGGCTGCTAGAGCTACCT
  ACTCAATCAGCCCTGGGCATCACCATCAGGTACTCGGCCAAAATGACCTCTCTGCTTCCA
  GTCCTCAGTTCTGGTCAGCACCAGACAGGCCCATAATTACAGAGCCAGGGAAACTGGAAC
  ATTTGTCTCCCCTTAGACAGTGGCAGCAGGAAGGTGGGGGGTTGTTGCAGAGGAACAGTG
  TCTCTGAGAGAGGACCTTGGACTTTCTGGGAAT
  ============================================================

Primers for ID: 29
Primer pair 1, product size : 953
  Primer left                            Primer right
  start: 25211485                        start: 25212417
  end  : 25211506                        end  : 25212437
  tm   : 57                              tm   : 57
  seq  : AAATGAGTTTCCATAAGGCCAG          seq  : AGATTCCCAGAAAGTCCAAGG

  >29_primer_pair_1_product
  AAATGAGTTTCCATAAGGCCAGGGAAACGCAGGTAGGAACCCATGCGGTCGAGCCAGCAC
  TCACCTGACACTAGGAACCGCTGGCCAAAGGTTTTGTTGCCGAAACTCTCAAAGTTGAAA
  TGGTCCATGTATTGCTCAAAATAATTCTCATGAAAGTCAGGGTCTAGAACTCTGTCGGCT
  GAGGGCAGGTGCAGAGACTCAGGAGCTGGTTGGGATCATCAGGGATCTAGGCGGGTCAGG
  AGGAAGGGCAGCCAGTCTGTACTCACCTCTGGCCTGGAGGTTGCACAGTCCCAGTGACAG
  CAGCAGGACCAGGATCCAGGAGGGGACACCATGGTCCACAGGGTAACAAGGATGGAAGTT
  CATGCTTGATTCTGAGCCGGGCGCTGACTGTCATGTGATTTGGTCACATGACCGACACAA
  CGGGCGGGGCAGCATCACGTGATAGTCTGGCGGGGGCTGTCCTACTGTGGCTGGATTCTA
  GTTGGAGGATCAGCCTACTCTTCTTCAGTTTCCCGGTTCCTCCAAATTTCTGGGCTCCTA
  CTTGTTTCCACAGAGATGGATACTGTGGAGGTCCAGGAAGCAGAGAGATGGCTAAGGCTC
  ATCAGGACCGTATGATCTCCCAAGTGTCCAGCTACTGAGTACCACAAGGTGATGGGTGGG
  AGGGTCCTCCCACGGAAGGATACCGCAGTCCCTAGGGGTTGCAAGCCCCACATGTTCCAC
  TGGCTGCTAGAGCTACCTACTCAATCAGCCCTGGGCATCACCATCAGGTACTCGGCCAAA
  ATGACCTCTCTGCTTCCAGTCCTCAGTTCTGGTCAGCACCAGACAGGCCCATAATTACAG
  AGCCAGGGAAACTGGAACATTTGTCTCCCCTTAGACAGTGGCAGCAGGAAGGTGGGGGGT
  TGTTGCAGAGGAACAGTGTCTCTGAGAGAGGACCTTGGACTTTCTGGGAATCT
  --------------------

Primer pair 2, product size : 951
  Primer left                            Primer right
  start: 25211485                        start: 25212415
  end  : 25211506                        end  : 25212435
  tm   : 57                              tm   : 57
  seq  : AAATGAGTTTCCATAAGGCCAG          seq  : ATTCCCAGAAAGTCCAAGGTC

  >29_primer_pair_2_product
  AAATGAGTTTCCATAAGGCCAGGGAAACGCAGGTAGGAACCCATGCGGTCGAGCCAGCAC
  TCACCTGACACTAGGAACCGCTGGCCAAAGGTTTTGTTGCCGAAACTCTCAAAGTTGAAA
  TGGTCCATGTATTGCTCAAAATAATTCTCATGAAAGTCAGGGTCTAGAACTCTGTCGGCT
  GAGGGCAGGTGCAGAGACTCAGGAGCTGGTTGGGATCATCAGGGATCTAGGCGGGTCAGG
  AGGAAGGGCAGCCAGTCTGTACTCACCTCTGGCCTGGAGGTTGCACAGTCCCAGTGACAG
  CAGCAGGACCAGGATCCAGGAGGGGACACCATGGTCCACAGGGTAACAAGGATGGAAGTT
  CATGCTTGATTCTGAGCCGGGCGCTGACTGTCATGTGATTTGGTCACATGACCGACACAA
  CGGGCGGGGCAGCATCACGTGATAGTCTGGCGGGGGCTGTCCTACTGTGGCTGGATTCTA
  GTTGGAGGATCAGCCTACTCTTCTTCAGTTTCCCGGTTCCTCCAAATTTCTGGGCTCCTA
  CTTGTTTCCACAGAGATGGATACTGTGGAGGTCCAGGAAGCAGAGAGATGGCTAAGGCTC
  ATCAGGACCGTATGATCTCCCAAGTGTCCAGCTACTGAGTACCACAAGGTGATGGGTGGG
  AGGGTCCTCCCACGGAAGGATACCGCAGTCCCTAGGGGTTGCAAGCCCCACATGTTCCAC
  TGGCTGCTAGAGCTACCTACTCAATCAGCCCTGGGCATCACCATCAGGTACTCGGCCAAA
  ATGACCTCTCTGCTTCCAGTCCTCAGTTCTGGTCAGCACCAGACAGGCCCATAATTACAG
  AGCCAGGGAAACTGGAACATTTGTCTCCCCTTAGACAGTGGCAGCAGGAAGGTGGGGGGT
  TGTTGCAGAGGAACAGTGTCTCTGAGAGAGGACCTTGGACTTTCTGGGAAT
  ============================================================

Primers for ID: 30
Primer pair 1, product size : 915
  Primer left                            Primer right
  start: 25209909                        start: 25210803
  end  : 25209929                        end  : 25210823
  tm   : 57                              tm   : 57
  seq  : GATGGCATGAAAGCAGATAGC           seq  : AGTGGGACAATTCCTTCTGAC

  >30_primer_pair_1_product
  GATGGCATGAAAGCAGATAGCTACAAGGCTCTTGGACCAGGCTAGCACTGGGTCCTGCAC
  CCAGGGAGAGCCACCTCACCTTGACAGGGTTGGCAGGAAGGGGCCCTAGAAAGTCAGTAG
  GATACGGGTAGTCCATCATGGCGAGCACAGTAAATGCATTTCGGGCAAACCCAAAGAGCT
  GAGTCAGGTCCTTTGGGCTGGAAAGTGATTGACAGGTACCAAAGTTCTGGCTGATGGTGT
  CATAGGCTGGGAAGAGAGAGGCCAGGAGAAAAGGCTGAGGAAACTGCTGGCAAATGTGAA
  GGGCAAGAATGAATGCCCAAGGTGGGCAGCAGGTGAGGAAAGAGTCCCTCACCTCCCTGG
  AGGAACAAGTCTTTGATTTGCTGAAAGGCATCCCGCACAGCCTGGGCGCACTTGGGACTC
  TGGCCATAAAAGTCCTGGAGAAGAGACCAAGGTTGCTGCTGCCATTCTTGCACTGGCCTG
  GGGTACCCAAGTCCCCTCACTCACCGCTGTGACATCTCGGAAGAATTGGTAGGAGTCCCC
  AAGGCCTGCAACAGCTACAACAGGAGCGCTGGCTGCCAGTGCCCCAGCCACCAGGTGGGG
  GTACTTCATCCTCATGTAGGCACTCAGCATCCCCCCATAACTGGGAGTACAGAGCACAGA
  TCATGGTTGTGGGAAGCTGCCCACAACTCAGGCGAGCAGCCTCACTGTCCTCCAGGCTGA
  GGTGCTAGGCTGCTCTTTCCCTGCTCAGAACGCCCAAGGGTGGGAAAGAAGGACCTGAAA
  CTGTCAGGCCCACACACCCTGATCCCAGGGCCAAGGCAGATACAGCCTTCACTGGGAGAA
  GGCACCTGTGGGTGCCCTGCCCTGACCCAGCAATGAAGACATTGCAGAGACAAAGTCAGA
  AGGAATTGTCCCACT
  --------------------

Primer pair 2, product size : 995
  Primer left                            Primer right
  start: 25209826                        start: 25210799
  end  : 25209847                        end  : 25210820
  tm   : 61                              tm   : 59
  seq  : AGCAGAATATCTCAGTGGAGGC          seq  : GGGACAATTCCTTCTGACTTTG

  >30_primer_pair_2_product
  AGCAGAATATCTCAGTGGAGGCCCCTTTCACAGAGCGTGGGTCAGGGCTGCTAGCTTCCA
  GGACACAACAGCAGATAGTGTCTGATGGCATGAAAGCAGATAGCTACAAGGCTCTTGGAC
  CAGGCTAGCACTGGGTCCTGCACCCAGGGAGAGCCACCTCACCTTGACAGGGTTGGCAGG
  AAGGGGCCCTAGAAAGTCAGTAGGATACGGGTAGTCCATCATGGCGAGCACAGTAAATGC
  ATTTCGGGCAAACCCAAAGAGCTGAGTCAGGTCCTTTGGGCTGGAAAGTGATTGACAGGT
  ACCAAAGTTCTGGCTGATGGTGTCATAGGCTGGGAAGAGAGAGGCCAGGAGAAAAGGCTG
  AGGAAACTGCTGGCAAATGTGAAGGGCAAGAATGAATGCCCAAGGTGGGCAGCAGGTGAG
  GAAAGAGTCCCTCACCTCCCTGGAGGAACAAGTCTTTGATTTGCTGAAAGGCATCCCGCA
  CAGCCTGGGCGCACTTGGGACTCTGGCCATAAAAGTCCTGGAGAAGAGACCAAGGTTGCT
  GCTGCCATTCTTGCACTGGCCTGGGGTACCCAAGTCCCCTCACTCACCGCTGTGACATCT
  CGGAAGAATTGGTAGGAGTCCCCAAGGCCTGCAACAGCTACAACAGGAGCGCTGGCTGCC
  AGTGCCCCAGCCACCAGGTGGGGGTACTTCATCCTCATGTAGGCACTCAGCATCCCCCCA
  TAACTGGGAGTACAGAGCACAGATCATGGTTGTGGGAAGCTGCCCACAACTCAGGCGAGC
  AGCCTCACTGTCCTCCAGGCTGAGGTGCTAGGCTGCTCTTTCCCTGCTCAGAACGCCCAA
  GGGTGGGAAAGAAGGACCTGAAACTGTCAGGCCCACACACCCTGATCCCAGGGCCAAGGC
  AGATACAGCCTTCACTGGGAGAAGGCACCTGTGGGTGCCCTGCCCTGACCCAGCAATGAA
  GACATTGCAGAGACAAAGTCAGAAGGAATTGTCCC
  ============================================================

Primers for ID: 31
Primer pair 1, product size : 1143
  Primer left                            Primer right
  start: 25209909                        start: 25211030
  end  : 25209929                        end  : 25211051
  tm   : 57                              tm   : 61
  seq  : GATGGCATGAAAGCAGATAGC           seq  : AGATCTGGACCCATGAGAGAAC

  >31_primer_pair_1_product
  GATGGCATGAAAGCAGATAGCTACAAGGCTCTTGGACCAGGCTAGCACTGGGTCCTGCAC
  CCAGGGAGAGCCACCTCACCTTGACAGGGTTGGCAGGAAGGGGCCCTAGAAAGTCAGTAG
  GATACGGGTAGTCCATCATGGCGAGCACAGTAAATGCATTTCGGGCAAACCCAAAGAGCT
  GAGTCAGGTCCTTTGGGCTGGAAAGTGATTGACAGGTACCAAAGTTCTGGCTGATGGTGT
  CATAGGCTGGGAAGAGAGAGGCCAGGAGAAAAGGCTGAGGAAACTGCTGGCAAATGTGAA
  GGGCAAGAATGAATGCCCAAGGTGGGCAGCAGGTGAGGAAAGAGTCCCTCACCTCCCTGG
  AGGAACAAGTCTTTGATTTGCTGAAAGGCATCCCGCACAGCCTGGGCGCACTTGGGACTC
  TGGCCATAAAAGTCCTGGAGAAGAGACCAAGGTTGCTGCTGCCATTCTTGCACTGGCCTG
  GGGTACCCAAGTCCCCTCACTCACCGCTGTGACATCTCGGAAGAATTGGTAGGAGTCCCC
  AAGGCCTGCAACAGCTACAACAGGAGCGCTGGCTGCCAGTGCCCCAGCCACCAGGTGGGG
  GTACTTCATCCTCATGTAGGCACTCAGCATCCCCCCATAACTGGGAGTACAGAGCACAGA
  TCATGGTTGTGGGAAGCTGCCCACAACTCAGGCGAGCAGCCTCACTGTCCTCCAGGCTGA
  GGTGCTAGGCTGCTCTTTCCCTGCTCAGAACGCCCAAGGGTGGGAAAGAAGGACCTGAAA
  CTGTCAGGCCCACACACCCTGATCCCAGGGCCAAGGCAGATACAGCCTTCACTGGGAGAA
  GGCACCTGTGGGTGCCCTGCCCTGACCCAGCAATGAAGACATTGCAGAGACAAAGTCAGA
  AGGAATTGTCCCACTAGTGGGAACAACATAGCATACACTGCCTATGAGGTCCACTCAAGG
  AGGGCTTCCAGAAGGAGGTAAAGCTAGACCCCGCCCTTCCACATGTGGGGTAGGCATAGG
  ATGTTGAGACTGTAAGAGACATCTCTTTGGCCCTCCTTGTATAGGGTGTCAATCGGCACA
  ACAGGGTGGAGCCTTAGAGTAGGGTAAGATTAGGACTCTAGGTTCTCTCATGGGTCCAGA
  TCT
  --------------------

Primer pair 2, product size : 1063
  Primer left                            Primer right
  start: 25209909                        start: 25210951
  end  : 25209929                        end  : 25210971
  tm   : 57                              tm   : 57
  seq  : GATGGCATGAAAGCAGATAGC           seq  : TATACAAGGAGGGCCAAAGAG

  >31_primer_pair_2_product
  GATGGCATGAAAGCAGATAGCTACAAGGCTCTTGGACCAGGCTAGCACTGGGTCCTGCAC
  CCAGGGAGAGCCACCTCACCTTGACAGGGTTGGCAGGAAGGGGCCCTAGAAAGTCAGTAG
  GATACGGGTAGTCCATCATGGCGAGCACAGTAAATGCATTTCGGGCAAACCCAAAGAGCT
  GAGTCAGGTCCTTTGGGCTGGAAAGTGATTGACAGGTACCAAAGTTCTGGCTGATGGTGT
  CATAGGCTGGGAAGAGAGAGGCCAGGAGAAAAGGCTGAGGAAACTGCTGGCAAATGTGAA
  GGGCAAGAATGAATGCCCAAGGTGGGCAGCAGGTGAGGAAAGAGTCCCTCACCTCCCTGG
  AGGAACAAGTCTTTGATTTGCTGAAAGGCATCCCGCACAGCCTGGGCGCACTTGGGACTC
  TGGCCATAAAAGTCCTGGAGAAGAGACCAAGGTTGCTGCTGCCATTCTTGCACTGGCCTG
  GGGTACCCAAGTCCCCTCACTCACCGCTGTGACATCTCGGAAGAATTGGTAGGAGTCCCC
  AAGGCCTGCAACAGCTACAACAGGAGCGCTGGCTGCCAGTGCCCCAGCCACCAGGTGGGG
  GTACTTCATCCTCATGTAGGCACTCAGCATCCCCCCATAACTGGGAGTACAGAGCACAGA
  TCATGGTTGTGGGAAGCTGCCCACAACTCAGGCGAGCAGCCTCACTGTCCTCCAGGCTGA
  GGTGCTAGGCTGCTCTTTCCCTGCTCAGAACGCCCAAGGGTGGGAAAGAAGGACCTGAAA
  CTGTCAGGCCCACACACCCTGATCCCAGGGCCAAGGCAGATACAGCCTTCACTGGGAGAA
  GGCACCTGTGGGTGCCCTGCCCTGACCCAGCAATGAAGACATTGCAGAGACAAAGTCAGA
  AGGAATTGTCCCACTAGTGGGAACAACATAGCATACACTGCCTATGAGGTCCACTCAAGG
  AGGGCTTCCAGAAGGAGGTAAAGCTAGACCCCGCCCTTCCACATGTGGGGTAGGCATAGG
  ATGTTGAGACTGTAAGAGACATCTCTTTGGCCCTCCTTGTATA
  ============================================================

Primers for ID: 32
Primer pair 1, product size : 919
  Primer left                            Primer right
  start: 25210133                        start: 25211030
  end  : 25210153                        end  : 25211051
  tm   : 57                              tm   : 61
  seq  : TTCTGGCTGATGGTGTCATAG           seq  : AGATCTGGACCCATGAGAGAAC

  >32_primer_pair_1_product
  TTCTGGCTGATGGTGTCATAGGCTGGGAAGAGAGAGGCCAGGAGAAAAGGCTGAGGAAAC
  TGCTGGCAAATGTGAAGGGCAAGAATGAATGCCCAAGGTGGGCAGCAGGTGAGGAAAGAG
  TCCCTCACCTCCCTGGAGGAACAAGTCTTTGATTTGCTGAAAGGCATCCCGCACAGCCTG
  GGCGCACTTGGGACTCTGGCCATAAAAGTCCTGGAGAAGAGACCAAGGTTGCTGCTGCCA
  TTCTTGCACTGGCCTGGGGTACCCAAGTCCCCTCACTCACCGCTGTGACATCTCGGAAGA
  ATTGGTAGGAGTCCCCAAGGCCTGCAACAGCTACAACAGGAGCGCTGGCTGCCAGTGCCC
  CAGCCACCAGGTGGGGGTACTTCATCCTCATGTAGGCACTCAGCATCCCCCCATAACTGG
  GAGTACAGAGCACAGATCATGGTTGTGGGAAGCTGCCCACAACTCAGGCGAGCAGCCTCA
  CTGTCCTCCAGGCTGAGGTGCTAGGCTGCTCTTTCCCTGCTCAGAACGCCCAAGGGTGGG
  AAAGAAGGACCTGAAACTGTCAGGCCCACACACCCTGATCCCAGGGCCAAGGCAGATACA
  GCCTTCACTGGGAGAAGGCACCTGTGGGTGCCCTGCCCTGACCCAGCAATGAAGACATTG
  CAGAGACAAAGTCAGAAGGAATTGTCCCACTAGTGGGAACAACATAGCATACACTGCCTA
  TGAGGTCCACTCAAGGAGGGCTTCCAGAAGGAGGTAAAGCTAGACCCCGCCCTTCCACAT
  GTGGGGTAGGCATAGGATGTTGAGACTGTAAGAGACATCTCTTTGGCCCTCCTTGTATAG
  GGTGTCAATCGGCACAACAGGGTGGAGCCTTAGAGTAGGGTAAGATTAGGACTCTAGGTT
  CTCTCATGGGTCCAGATCT
  --------------------

Primer pair 2, product size : 924
  Primer left                            Primer right
  start: 25210128                        start: 25211030
  end  : 25210147                        end  : 25211051
  tm   : 55                              tm   : 61
  seq  : CAAAGTTCTGGCTGATGGTG            seq  : AGATCTGGACCCATGAGAGAAC

  >32_primer_pair_2_product
  CAAAGTTCTGGCTGATGGTGTCATAGGCTGGGAAGAGAGAGGCCAGGAGAAAAGGCTGAG
  GAAACTGCTGGCAAATGTGAAGGGCAAGAATGAATGCCCAAGGTGGGCAGCAGGTGAGGA
  AAGAGTCCCTCACCTCCCTGGAGGAACAAGTCTTTGATTTGCTGAAAGGCATCCCGCACA
  GCCTGGGCGCACTTGGGACTCTGGCCATAAAAGTCCTGGAGAAGAGACCAAGGTTGCTGC
  TGCCATTCTTGCACTGGCCTGGGGTACCCAAGTCCCCTCACTCACCGCTGTGACATCTCG
  GAAGAATTGGTAGGAGTCCCCAAGGCCTGCAACAGCTACAACAGGAGCGCTGGCTGCCAG
  TGCCCCAGCCACCAGGTGGGGGTACTTCATCCTCATGTAGGCACTCAGCATCCCCCCATA
  ACTGGGAGTACAGAGCACAGATCATGGTTGTGGGAAGCTGCCCACAACTCAGGCGAGCAG
  CCTCACTGTCCTCCAGGCTGAGGTGCTAGGCTGCTCTTTCCCTGCTCAGAACGCCCAAGG
  GTGGGAAAGAAGGACCTGAAACTGTCAGGCCCACACACCCTGATCCCAGGGCCAAGGCAG
  ATACAGCCTTCACTGGGAGAAGGCACCTGTGGGTGCCCTGCCCTGACCCAGCAATGAAGA
  CATTGCAGAGACAAAGTCAGAAGGAATTGTCCCACTAGTGGGAACAACATAGCATACACT
  GCCTATGAGGTCCACTCAAGGAGGGCTTCCAGAAGGAGGTAAAGCTAGACCCCGCCCTTC
  CACATGTGGGGTAGGCATAGGATGTTGAGACTGTAAGAGACATCTCTTTGGCCCTCCTTG
  TATAGGGTGTCAATCGGCACAACAGGGTGGAGCCTTAGAGTAGGGTAAGATTAGGACTCT
  AGGTTCTCTCATGGGTCCAGATCT
  ============================================================

Primers for ID: 42
Primer pair 1, product size : 939
  Primer left                            Primer right
  start: 25211383                        start: 25212301
  end  : 25211403                        end  : 25212321
  tm   : 57                              tm   : 57
  seq  : ATGAAGCCAGAGTTGTTAGCG           seq  : TAATTATGGGCCTGTCTGGTG

  >42_primer_pair_1_product
  ATGAAGCCAGAGTTGTTAGCGAAGGACCAGATATCCCCCTCATTCCCTGTGTAGAAAAAG
  ATGGGCCCTTCGCCCATCTTCCAGAACTTATCTGTTGGAAGTAAATGAGTTTCCATAAGG
  CCAGGGAAACGCAGGTAGGAACCCATGCGGTCGAGCCAGCACTCACCTGACACTAGGAAC
  CGCTGGCCAAAGGTTTTGTTGCCGAAACTCTCAAAGTTGAAATGGTCCATGTATTGCTCA
  AAATAATTCTCATGAAAGTCAGGGTCTAGAACTCTGTCGGCTGAGGGCAGGTGCAGAGAC
  TCAGGAGCTGGTTGGGATCATCAGGGATCTAGGCGGGTCAGGAGGAAGGGCAGCCAGTCT
  GTACTCACCTCTGGCCTGGAGGTTGCACAGTCCCAGTGACAGCAGCAGGACCAGGATCCA
  GGAGGGGACACCATGGTCCACAGGGTAACAAGGATGGAAGTTCATGCTTGATTCTGAGCC
  GGGCGCTGACTGTCATGTGATTTGGTCACATGACCGACACAACGGGCGGGGCAGCATCAC
  GTGATAGTCTGGCGGGGGCTGTCCTACTGTGGCTGGATTCTAGTTGGAGGATCAGCCTAC
  TCTTCTTCAGTTTCCCGGTTCCTCCAAATTTCTGGGCTCCTACTTGTTTCCACAGAGATG
  GATACTGTGGAGGTCCAGGAAGCAGAGAGATGGCTAAGGCTCATCAGGACCGTATGATCT
  CCCAAGTGTCCAGCTACTGAGTACCACAAGGTGATGGGTGGGAGGGTCCTCCCACGGAAG
  GATACCGCAGTCCCTAGGGGTTGCAAGCCCCACATGTTCCACTGGCTGCTAGAGCTACCT
  ACTCAATCAGCCCTGGGCATCACCATCAGGTACTCGGCCAAAATGACCTCTCTGCTTCCA
  GTCCTCAGTTCTGGTCAGCACCAGACAGGCCCATAATTA
  --------------------

Primer pair 2, product size : 941
  Primer left                            Primer right
  start: 25211383                        start: 25212303
  end  : 25211403                        end  : 25212323
  tm   : 57                              tm   : 57
  seq  : ATGAAGCCAGAGTTGTTAGCG           seq  : TGTAATTATGGGCCTGTCTGG

  >42_primer_pair_2_product
  ATGAAGCCAGAGTTGTTAGCGAAGGACCAGATATCCCCCTCATTCCCTGTGTAGAAAAAG
  ATGGGCCCTTCGCCCATCTTCCAGAACTTATCTGTTGGAAGTAAATGAGTTTCCATAAGG
  CCAGGGAAACGCAGGTAGGAACCCATGCGGTCGAGCCAGCACTCACCTGACACTAGGAAC
  CGCTGGCCAAAGGTTTTGTTGCCGAAACTCTCAAAGTTGAAATGGTCCATGTATTGCTCA
  AAATAATTCTCATGAAAGTCAGGGTCTAGAACTCTGTCGGCTGAGGGCAGGTGCAGAGAC
  TCAGGAGCTGGTTGGGATCATCAGGGATCTAGGCGGGTCAGGAGGAAGGGCAGCCAGTCT
  GTACTCACCTCTGGCCTGGAGGTTGCACAGTCCCAGTGACAGCAGCAGGACCAGGATCCA
  GGAGGGGACACCATGGTCCACAGGGTAACAAGGATGGAAGTTCATGCTTGATTCTGAGCC
  GGGCGCTGACTGTCATGTGATTTGGTCACATGACCGACACAACGGGCGGGGCAGCATCAC
  GTGATAGTCTGGCGGGGGCTGTCCTACTGTGGCTGGATTCTAGTTGGAGGATCAGCCTAC
  TCTTCTTCAGTTTCCCGGTTCCTCCAAATTTCTGGGCTCCTACTTGTTTCCACAGAGATG
  GATACTGTGGAGGTCCAGGAAGCAGAGAGATGGCTAAGGCTCATCAGGACCGTATGATCT
  CCCAAGTGTCCAGCTACTGAGTACCACAAGGTGATGGGTGGGAGGGTCCTCCCACGGAAG
  GATACCGCAGTCCCTAGGGGTTGCAAGCCCCACATGTTCCACTGGCTGCTAGAGCTACCT
  ACTCAATCAGCCCTGGGCATCACCATCAGGTACTCGGCCAAAATGACCTCTCTGCTTCCA
  GTCCTCAGTTCTGGTCAGCACCAGACAGGCCCATAATTACA
  ============================================================

Primers for ID: 43
Primer pair 1, product size : 939
  Primer left                            Primer right
  start: 25211383                        start: 25212301
  end  : 25211403                        end  : 25212321
  tm   : 57                              tm   : 57
  seq  : ATGAAGCCAGAGTTGTTAGCG           seq  : TAATTATGGGCCTGTCTGGTG

  >43_primer_pair_1_product
  ATGAAGCCAGAGTTGTTAGCGAAGGACCAGATATCCCCCTCATTCCCTGTGTAGAAAAAG
  ATGGGCCCTTCGCCCATCTTCCAGAACTTATCTGTTGGAAGTAAATGAGTTTCCATAAGG
  CCAGGGAAACGCAGGTAGGAACCCATGCGGTCGAGCCAGCACTCACCTGACACTAGGAAC
  CGCTGGCCAAAGGTTTTGTTGCCGAAACTCTCAAAGTTGAAATGGTCCATGTATTGCTCA
  AAATAATTCTCATGAAAGTCAGGGTCTAGAACTCTGTCGGCTGAGGGCAGGTGCAGAGAC
  TCAGGAGCTGGTTGGGATCATCAGGGATCTAGGCGGGTCAGGAGGAAGGGCAGCCAGTCT
  GTACTCACCTCTGGCCTGGAGGTTGCACAGTCCCAGTGACAGCAGCAGGACCAGGATCCA
  GGAGGGGACACCATGGTCCACAGGGTAACAAGGATGGAAGTTCATGCTTGATTCTGAGCC
  GGGCGCTGACTGTCATGTGATTTGGTCACATGACCGACACAACGGGCGGGGCAGCATCAC
  GTGATAGTCTGGCGGGGGCTGTCCTACTGTGGCTGGATTCTAGTTGGAGGATCAGCCTAC
  TCTTCTTCAGTTTCCCGGTTCCTCCAAATTTCTGGGCTCCTACTTGTTTCCACAGAGATG
  GATACTGTGGAGGTCCAGGAAGCAGAGAGATGGCTAAGGCTCATCAGGACCGTATGATCT
  CCCAAGTGTCCAGCTACTGAGTACCACAAGGTGATGGGTGGGAGGGTCCTCCCACGGAAG
  GATACCGCAGTCCCTAGGGGTTGCAAGCCCCACATGTTCCACTGGCTGCTAGAGCTACCT
  ACTCAATCAGCCCTGGGCATCACCATCAGGTACTCGGCCAAAATGACCTCTCTGCTTCCA
  GTCCTCAGTTCTGGTCAGCACCAGACAGGCCCATAATTA
  --------------------

Primer pair 2, product size : 941
  Primer left                            Primer right
  start: 25211383                        start: 25212303
  end  : 25211403                        end  : 25212323
  tm   : 57                              tm   : 57
  seq  : ATGAAGCCAGAGTTGTTAGCG           seq  : TGTAATTATGGGCCTGTCTGG

  >43_primer_pair_2_product
  ATGAAGCCAGAGTTGTTAGCGAAGGACCAGATATCCCCCTCATTCCCTGTGTAGAAAAAG
  ATGGGCCCTTCGCCCATCTTCCAGAACTTATCTGTTGGAAGTAAATGAGTTTCCATAAGG
  CCAGGGAAACGCAGGTAGGAACCCATGCGGTCGAGCCAGCACTCACCTGACACTAGGAAC
  CGCTGGCCAAAGGTTTTGTTGCCGAAACTCTCAAAGTTGAAATGGTCCATGTATTGCTCA
  AAATAATTCTCATGAAAGTCAGGGTCTAGAACTCTGTCGGCTGAGGGCAGGTGCAGAGAC
  TCAGGAGCTGGTTGGGATCATCAGGGATCTAGGCGGGTCAGGAGGAAGGGCAGCCAGTCT
  GTACTCACCTCTGGCCTGGAGGTTGCACAGTCCCAGTGACAGCAGCAGGACCAGGATCCA
  GGAGGGGACACCATGGTCCACAGGGTAACAAGGATGGAAGTTCATGCTTGATTCTGAGCC
  GGGCGCTGACTGTCATGTGATTTGGTCACATGACCGACACAACGGGCGGGGCAGCATCAC
  GTGATAGTCTGGCGGGGGCTGTCCTACTGTGGCTGGATTCTAGTTGGAGGATCAGCCTAC
  TCTTCTTCAGTTTCCCGGTTCCTCCAAATTTCTGGGCTCCTACTTGTTTCCACAGAGATG
  GATACTGTGGAGGTCCAGGAAGCAGAGAGATGGCTAAGGCTCATCAGGACCGTATGATCT
  CCCAAGTGTCCAGCTACTGAGTACCACAAGGTGATGGGTGGGAGGGTCCTCCCACGGAAG
  GATACCGCAGTCCCTAGGGGTTGCAAGCCCCACATGTTCCACTGGCTGCTAGAGCTACCT
  ACTCAATCAGCCCTGGGCATCACCATCAGGTACTCGGCCAAAATGACCTCTCTGCTTCCA
  GTCCTCAGTTCTGGTCAGCACCAGACAGGCCCATAATTACA
  ============================================================

Primers for ID: 44
Primer pair 1, product size : 1010
  Primer left                            Primer right
  start: 25211428                        start: 25212417
  end  : 25211448                        end  : 25212437
  tm   : 57                              tm   : 57
  seq  : CCTGTGTAGAAAAAGATGGGC           seq  : AGATTCCCAGAAAGTCCAAGG

  >44_primer_pair_1_product
  CCTGTGTAGAAAAAGATGGGCCCTTCGCCCATCTTCCAGAACTTATCTGTTGGAAGTAAA
  TGAGTTTCCATAAGGCCAGGGAAACGCAGGTAGGAACCCATGCGGTCGAGCCAGCACTCA
  CCTGACACTAGGAACCGCTGGCCAAAGGTTTTGTTGCCGAAACTCTCAAAGTTGAAATGG
  TCCATGTATTGCTCAAAATAATTCTCATGAAAGTCAGGGTCTAGAACTCTGTCGGCTGAG
  GGCAGGTGCAGAGACTCAGGAGCTGGTTGGGATCATCAGGGATCTAGGCGGGTCAGGAGG
  AAGGGCAGCCAGTCTGTACTCACCTCTGGCCTGGAGGTTGCACAGTCCCAGTGACAGCAG
  CAGGACCAGGATCCAGGAGGGGACACCATGGTCCACAGGGTAACAAGGATGGAAGTTCAT
  GCTTGATTCTGAGCCGGGCGCTGACTGTCATGTGATTTGGTCACATGACCGACACAACGG
  GCGGGGCAGCATCACGTGATAGTCTGGCGGGGGCTGTCCTACTGTGGCTGGATTCTAGTT
  GGAGGATCAGCCTACTCTTCTTCAGTTTCCCGGTTCCTCCAAATTTCTGGGCTCCTACTT
  GTTTCCACAGAGATGGATACTGTGGAGGTCCAGGAAGCAGAGAGATGGCTAAGGCTCATC
  AGGACCGTATGATCTCCCAAGTGTCCAGCTACTGAGTACCACAAGGTGATGGGTGGGAGG
  GTCCTCCCACGGAAGGATACCGCAGTCCCTAGGGGTTGCAAGCCCCACATGTTCCACTGG
  CTGCTAGAGCTACCTACTCAATCAGCCCTGGGCATCACCATCAGGTACTCGGCCAAAATG
  ACCTCTCTGCTTCCAGTCCTCAGTTCTGGTCAGCACCAGACAGGCCCATAATTACAGAGC
  CAGGGAAACTGGAACATTTGTCTCCCCTTAGACAGTGGCAGCAGGAAGGTGGGGGGTTGT
  TGCAGAGGAACAGTGTCTCTGAGAGAGGACCTTGGACTTTCTGGGAATCT
  --------------------

Primer pair 2, product size : 1008
  Primer left                            Primer right
  start: 25211428                        start: 25212415
  end  : 25211448                        end  : 25212435
  tm   : 57                              tm   : 57
  seq  : CCTGTGTAGAAAAAGATGGGC           seq  : ATTCCCAGAAAGTCCAAGGTC

  >44_primer_pair_2_product
  CCTGTGTAGAAAAAGATGGGCCCTTCGCCCATCTTCCAGAACTTATCTGTTGGAAGTAAA
  TGAGTTTCCATAAGGCCAGGGAAACGCAGGTAGGAACCCATGCGGTCGAGCCAGCACTCA
  CCTGACACTAGGAACCGCTGGCCAAAGGTTTTGTTGCCGAAACTCTCAAAGTTGAAATGG
  TCCATGTATTGCTCAAAATAATTCTCATGAAAGTCAGGGTCTAGAACTCTGTCGGCTGAG
  GGCAGGTGCAGAGACTCAGGAGCTGGTTGGGATCATCAGGGATCTAGGCGGGTCAGGAGG
  AAGGGCAGCCAGTCTGTACTCACCTCTGGCCTGGAGGTTGCACAGTCCCAGTGACAGCAG
  CAGGACCAGGATCCAGGAGGGGACACCATGGTCCACAGGGTAACAAGGATGGAAGTTCAT
  GCTTGATTCTGAGCCGGGCGCTGACTGTCATGTGATTTGGTCACATGACCGACACAACGG
  GCGGGGCAGCATCACGTGATAGTCTGGCGGGGGCTGTCCTACTGTGGCTGGATTCTAGTT
  GGAGGATCAGCCTACTCTTCTTCAGTTTCCCGGTTCCTCCAAATTTCTGGGCTCCTACTT
  GTTTCCACAGAGATGGATACTGTGGAGGTCCAGGAAGCAGAGAGATGGCTAAGGCTCATC
  AGGACCGTATGATCTCCCAAGTGTCCAGCTACTGAGTACCACAAGGTGATGGGTGGGAGG
  GTCCTCCCACGGAAGGATACCGCAGTCCCTAGGGGTTGCAAGCCCCACATGTTCCACTGG
  CTGCTAGAGCTACCTACTCAATCAGCCCTGGGCATCACCATCAGGTACTCGGCCAAAATG
  ACCTCTCTGCTTCCAGTCCTCAGTTCTGGTCAGCACCAGACAGGCCCATAATTACAGAGC
  CAGGGAAACTGGAACATTTGTCTCCCCTTAGACAGTGGCAGCAGGAAGGTGGGGGGTTGT
  TGCAGAGGAACAGTGTCTCTGAGAGAGGACCTTGGACTTTCTGGGAAT
  ============================================================

Primers for ID: 45
Primer pair 1, product size : 961
  Primer left                            Primer right
  start: 25211600                        start: 25212540
  end  : 25211621                        end  : 25212560
  tm   : 57                              tm   : 57
  seq  : TGAAATGGTCCATGTATTGCTC          seq  : ATGAGACACTCTGTGCCAATG

  >45_primer_pair_1_product
  TGAAATGGTCCATGTATTGCTCAAAATAATTCTCATGAAAGTCAGGGTCTAGAACTCTGT
  CGGCTGAGGGCAGGTGCAGAGACTCAGGAGCTGGTTGGGATCATCAGGGATCTAGGCGGG
  TCAGGAGGAAGGGCAGCCAGTCTGTACTCACCTCTGGCCTGGAGGTTGCACAGTCCCAGT
  GACAGCAGCAGGACCAGGATCCAGGAGGGGACACCATGGTCCACAGGGTAACAAGGATGG
  AAGTTCATGCTTGATTCTGAGCCGGGCGCTGACTGTCATGTGATTTGGTCACATGACCGA
  CACAACGGGCGGGGCAGCATCACGTGATAGTCTGGCGGGGGCTGTCCTACTGTGGCTGGA
  TTCTAGTTGGAGGATCAGCCTACTCTTCTTCAGTTTCCCGGTTCCTCCAAATTTCTGGGC
  TCCTACTTGTTTCCACAGAGATGGATACTGTGGAGGTCCAGGAAGCAGAGAGATGGCTAA
  GGCTCATCAGGACCGTATGATCTCCCAAGTGTCCAGCTACTGAGTACCACAAGGTGATGG
  GTGGGAGGGTCCTCCCACGGAAGGATACCGCAGTCCCTAGGGGTTGCAAGCCCCACATGT
  TCCACTGGCTGCTAGAGCTACCTACTCAATCAGCCCTGGGCATCACCATCAGGTACTCGG
  CCAAAATGACCTCTCTGCTTCCAGTCCTCAGTTCTGGTCAGCACCAGACAGGCCCATAAT
  TACAGAGCCAGGGAAACTGGAACATTTGTCTCCCCTTAGACAGTGGCAGCAGGAAGGTGG
  GGGGTTGTTGCAGAGGAACAGTGTCTCTGAGAGAGGACCTTGGACTTTCTGGGAATCTCT
  GAGCTGCCCGGTTCTCCCCACTGCTGGCACTGTGCCCACAGCCCAAACAGAATGGGGGAG
  ATGGAGGGGCAGGGCTTCTGTGGGAAGCTGCCCTCCACCTCATTGGCACAGAGTGTCTCA
  T
  --------------------

Primer pair 2, product size : 1073
  Primer left                            Primer right
  start: 25211600                        start: 25212653
  end  : 25211621                        end  : 25212672
  tm   : 57                              tm   : 55
  seq  : TGAAATGGTCCATGTATTGCTC          seq  : ATGGACATGTTCCACTCACG

  >45_primer_pair_2_product
  TGAAATGGTCCATGTATTGCTCAAAATAATTCTCATGAAAGTCAGGGTCTAGAACTCTGT
  CGGCTGAGGGCAGGTGCAGAGACTCAGGAGCTGGTTGGGATCATCAGGGATCTAGGCGGG
  TCAGGAGGAAGGGCAGCCAGTCTGTACTCACCTCTGGCCTGGAGGTTGCACAGTCCCAGT
  GACAGCAGCAGGACCAGGATCCAGGAGGGGACACCATGGTCCACAGGGTAACAAGGATGG
  AAGTTCATGCTTGATTCTGAGCCGGGCGCTGACTGTCATGTGATTTGGTCACATGACCGA
  CACAACGGGCGGGGCAGCATCACGTGATAGTCTGGCGGGGGCTGTCCTACTGTGGCTGGA
  TTCTAGTTGGAGGATCAGCCTACTCTTCTTCAGTTTCCCGGTTCCTCCAAATTTCTGGGC
  TCCTACTTGTTTCCACAGAGATGGATACTGTGGAGGTCCAGGAAGCAGAGAGATGGCTAA
  GGCTCATCAGGACCGTATGATCTCCCAAGTGTCCAGCTACTGAGTACCACAAGGTGATGG
  GTGGGAGGGTCCTCCCACGGAAGGATACCGCAGTCCCTAGGGGTTGCAAGCCCCACATGT
  TCCACTGGCTGCTAGAGCTACCTACTCAATCAGCCCTGGGCATCACCATCAGGTACTCGG
  CCAAAATGACCTCTCTGCTTCCAGTCCTCAGTTCTGGTCAGCACCAGACAGGCCCATAAT
  TACAGAGCCAGGGAAACTGGAACATTTGTCTCCCCTTAGACAGTGGCAGCAGGAAGGTGG
  GGGGTTGTTGCAGAGGAACAGTGTCTCTGAGAGAGGACCTTGGACTTTCTGGGAATCTCT
  GAGCTGCCCGGTTCTCCCCACTGCTGGCACTGTGCCCACAGCCCAAACAGAATGGGGGAG
  ATGGAGGGGCAGGGCTTCTGTGGGAAGCTGCCCTCCACCTCATTGGCACAGAGTGTCTCA
  TTGCAGAGAGAAAAAAGGACCAGTTTTCTCTCTGGCACCCAGGTCTGGAAGAGGAGTGAC
  ATCCACGGAAGTTGGTGACTTGGACTGGCTGGCCGTGAGTGGAACATGTCCAT
  ============================================================

Primers for ID: 46
Primer pair 1, product size : 881
  Primer left                            Primer right
  start: 25209909                        start: 25210770
  end  : 25209929                        end  : 25210789
  tm   : 57                              tm   : 55
  seq  : GATGGCATGAAAGCAGATAGC           seq  : TGTCTTCATTGCTGGGTCAG

  >46_primer_pair_1_product
  GATGGCATGAAAGCAGATAGCTACAAGGCTCTTGGACCAGGCTAGCACTGGGTCCTGCAC
  CCAGGGAGAGCCACCTCACCTTGACAGGGTTGGCAGGAAGGGGCCCTAGAAAGTCAGTAG
  GATACGGGTAGTCCATCATGGCGAGCACAGTAAATGCATTTCGGGCAAACCCAAAGAGCT
  GAGTCAGGTCCTTTGGGCTGGAAAGTGATTGACAGGTACCAAAGTTCTGGCTGATGGTGT
  CATAGGCTGGGAAGAGAGAGGCCAGGAGAAAAGGCTGAGGAAACTGCTGGCAAATGTGAA
  GGGCAAGAATGAATGCCCAAGGTGGGCAGCAGGTGAGGAAAGAGTCCCTCACCTCCCTGG
  AGGAACAAGTCTTTGATTTGCTGAAAGGCATCCCGCACAGCCTGGGCGCACTTGGGACTC
  TGGCCATAAAAGTCCTGGAGAAGAGACCAAGGTTGCTGCTGCCATTCTTGCACTGGCCTG
  GGGTACCCAAGTCCCCTCACTCACCGCTGTGACATCTCGGAAGAATTGGTAGGAGTCCCC
  AAGGCCTGCAACAGCTACAACAGGAGCGCTGGCTGCCAGTGCCCCAGCCACCAGGTGGGG
  GTACTTCATCCTCATGTAGGCACTCAGCATCCCCCCATAACTGGGAGTACAGAGCACAGA
  TCATGGTTGTGGGAAGCTGCCCACAACTCAGGCGAGCAGCCTCACTGTCCTCCAGGCTGA
  GGTGCTAGGCTGCTCTTTCCCTGCTCAGAACGCCCAAGGGTGGGAAAGAAGGACCTGAAA
  CTGTCAGGCCCACACACCCTGATCCCAGGGCCAAGGCAGATACAGCCTTCACTGGGAGAA
  GGCACCTGTGGGTGCCCTGCCCTGACCCAGCAATGAAGACA
  --------------------

Primer pair 2, product size : 915
  Primer left                            Primer right
  start: 25209909                        start: 25210803
  end  : 25209929                        end  : 25210823
  tm   : 57                              tm   : 57
  seq  : GATGGCATGAAAGCAGATAGC           seq  : AGTGGGACAATTCCTTCTGAC

  >46_primer_pair_2_product
  GATGGCATGAAAGCAGATAGCTACAAGGCTCTTGGACCAGGCTAGCACTGGGTCCTGCAC
  CCAGGGAGAGCCACCTCACCTTGACAGGGTTGGCAGGAAGGGGCCCTAGAAAGTCAGTAG
  GATACGGGTAGTCCATCATGGCGAGCACAGTAAATGCATTTCGGGCAAACCCAAAGAGCT
  GAGTCAGGTCCTTTGGGCTGGAAAGTGATTGACAGGTACCAAAGTTCTGGCTGATGGTGT
  CATAGGCTGGGAAGAGAGAGGCCAGGAGAAAAGGCTGAGGAAACTGCTGGCAAATGTGAA
  GGGCAAGAATGAATGCCCAAGGTGGGCAGCAGGTGAGGAAAGAGTCCCTCACCTCCCTGG
  AGGAACAAGTCTTTGATTTGCTGAAAGGCATCCCGCACAGCCTGGGCGCACTTGGGACTC
  TGGCCATAAAAGTCCTGGAGAAGAGACCAAGGTTGCTGCTGCCATTCTTGCACTGGCCTG
  GGGTACCCAAGTCCCCTCACTCACCGCTGTGACATCTCGGAAGAATTGGTAGGAGTCCCC
  AAGGCCTGCAACAGCTACAACAGGAGCGCTGGCTGCCAGTGCCCCAGCCACCAGGTGGGG
  GTACTTCATCCTCATGTAGGCACTCAGCATCCCCCCATAACTGGGAGTACAGAGCACAGA
  TCATGGTTGTGGGAAGCTGCCCACAACTCAGGCGAGCAGCCTCACTGTCCTCCAGGCTGA
  GGTGCTAGGCTGCTCTTTCCCTGCTCAGAACGCCCAAGGGTGGGAAAGAAGGACCTGAAA
  CTGTCAGGCCCACACACCCTGATCCCAGGGCCAAGGCAGATACAGCCTTCACTGGGAGAA
  GGCACCTGTGGGTGCCCTGCCCTGACCCAGCAATGAAGACATTGCAGAGACAAAGTCAGA
  AGGAATTGTCCCACT
  ============================================================

Primers for ID: 47
Primer pair 1, product size : 881
  Primer left                            Primer right
  start: 25209909                        start: 25210770
  end  : 25209929                        end  : 25210789
  tm   : 57                              tm   : 55
  seq  : GATGGCATGAAAGCAGATAGC           seq  : TGTCTTCATTGCTGGGTCAG

  >47_primer_pair_1_product
  GATGGCATGAAAGCAGATAGCTACAAGGCTCTTGGACCAGGCTAGCACTGGGTCCTGCAC
  CCAGGGAGAGCCACCTCACCTTGACAGGGTTGGCAGGAAGGGGCCCTAGAAAGTCAGTAG
  GATACGGGTAGTCCATCATGGCGAGCACAGTAAATGCATTTCGGGCAAACCCAAAGAGCT
  GAGTCAGGTCCTTTGGGCTGGAAAGTGATTGACAGGTACCAAAGTTCTGGCTGATGGTGT
  CATAGGCTGGGAAGAGAGAGGCCAGGAGAAAAGGCTGAGGAAACTGCTGGCAAATGTGAA
  GGGCAAGAATGAATGCCCAAGGTGGGCAGCAGGTGAGGAAAGAGTCCCTCACCTCCCTGG
  AGGAACAAGTCTTTGATTTGCTGAAAGGCATCCCGCACAGCCTGGGCGCACTTGGGACTC
  TGGCCATAAAAGTCCTGGAGAAGAGACCAAGGTTGCTGCTGCCATTCTTGCACTGGCCTG
  GGGTACCCAAGTCCCCTCACTCACCGCTGTGACATCTCGGAAGAATTGGTAGGAGTCCCC
  AAGGCCTGCAACAGCTACAACAGGAGCGCTGGCTGCCAGTGCCCCAGCCACCAGGTGGGG
  GTACTTCATCCTCATGTAGGCACTCAGCATCCCCCCATAACTGGGAGTACAGAGCACAGA
  TCATGGTTGTGGGAAGCTGCCCACAACTCAGGCGAGCAGCCTCACTGTCCTCCAGGCTGA
  GGTGCTAGGCTGCTCTTTCCCTGCTCAGAACGCCCAAGGGTGGGAAAGAAGGACCTGAAA
  CTGTCAGGCCCACACACCCTGATCCCAGGGCCAAGGCAGATACAGCCTTCACTGGGAGAA
  GGCACCTGTGGGTGCCCTGCCCTGACCCAGCAATGAAGACA
  --------------------

Primer pair 2, product size : 1063
  Primer left                            Primer right
  start: 25209909                        start: 25210951
  end  : 25209929                        end  : 25210971
  tm   : 57                              tm   : 57
  seq  : GATGGCATGAAAGCAGATAGC           seq  : TATACAAGGAGGGCCAAAGAG

  >47_primer_pair_2_product
  GATGGCATGAAAGCAGATAGCTACAAGGCTCTTGGACCAGGCTAGCACTGGGTCCTGCAC
  CCAGGGAGAGCCACCTCACCTTGACAGGGTTGGCAGGAAGGGGCCCTAGAAAGTCAGTAG
  GATACGGGTAGTCCATCATGGCGAGCACAGTAAATGCATTTCGGGCAAACCCAAAGAGCT
  GAGTCAGGTCCTTTGGGCTGGAAAGTGATTGACAGGTACCAAAGTTCTGGCTGATGGTGT
  CATAGGCTGGGAAGAGAGAGGCCAGGAGAAAAGGCTGAGGAAACTGCTGGCAAATGTGAA
  GGGCAAGAATGAATGCCCAAGGTGGGCAGCAGGTGAGGAAAGAGTCCCTCACCTCCCTGG
  AGGAACAAGTCTTTGATTTGCTGAAAGGCATCCCGCACAGCCTGGGCGCACTTGGGACTC
  TGGCCATAAAAGTCCTGGAGAAGAGACCAAGGTTGCTGCTGCCATTCTTGCACTGGCCTG
  GGGTACCCAAGTCCCCTCACTCACCGCTGTGACATCTCGGAAGAATTGGTAGGAGTCCCC
  AAGGCCTGCAACAGCTACAACAGGAGCGCTGGCTGCCAGTGCCCCAGCCACCAGGTGGGG
  GTACTTCATCCTCATGTAGGCACTCAGCATCCCCCCATAACTGGGAGTACAGAGCACAGA
  TCATGGTTGTGGGAAGCTGCCCACAACTCAGGCGAGCAGCCTCACTGTCCTCCAGGCTGA
  GGTGCTAGGCTGCTCTTTCCCTGCTCAGAACGCCCAAGGGTGGGAAAGAAGGACCTGAAA
  CTGTCAGGCCCACACACCCTGATCCCAGGGCCAAGGCAGATACAGCCTTCACTGGGAGAA
  GGCACCTGTGGGTGCCCTGCCCTGACCCAGCAATGAAGACATTGCAGAGACAAAGTCAGA
  AGGAATTGTCCCACTAGTGGGAACAACATAGCATACACTGCCTATGAGGTCCACTCAAGG
  AGGGCTTCCAGAAGGAGGTAAAGCTAGACCCCGCCCTTCCACATGTGGGGTAGGCATAGG
  ATGTTGAGACTGTAAGAGACATCTCTTTGGCCCTCCTTGTATA
  ============================================================

Primers for ID: 48
Primer pair 1, product size : 880
  Primer left                            Primer right
  start: 25210172                        start: 25211030
  end  : 25210192                        end  : 25211051
  tm   : 57                              tm   : 61
  seq  : AGGAGAAAAGGCTGAGGAAAC           seq  : AGATCTGGACCCATGAGAGAAC

  >48_primer_pair_1_product
  AGGAGAAAAGGCTGAGGAAACTGCTGGCAAATGTGAAGGGCAAGAATGAATGCCCAAGGT
  GGGCAGCAGGTGAGGAAAGAGTCCCTCACCTCCCTGGAGGAACAAGTCTTTGATTTGCTG
  AAAGGCATCCCGCACAGCCTGGGCGCACTTGGGACTCTGGCCATAAAAGTCCTGGAGAAG
  AGACCAAGGTTGCTGCTGCCATTCTTGCACTGGCCTGGGGTACCCAAGTCCCCTCACTCA
  CCGCTGTGACATCTCGGAAGAATTGGTAGGAGTCCCCAAGGCCTGCAACAGCTACAACAG
  GAGCGCTGGCTGCCAGTGCCCCAGCCACCAGGTGGGGGTACTTCATCCTCATGTAGGCAC
  TCAGCATCCCCCCATAACTGGGAGTACAGAGCACAGATCATGGTTGTGGGAAGCTGCCCA
  CAACTCAGGCGAGCAGCCTCACTGTCCTCCAGGCTGAGGTGCTAGGCTGCTCTTTCCCTG
  CTCAGAACGCCCAAGGGTGGGAAAGAAGGACCTGAAACTGTCAGGCCCACACACCCTGAT
  CCCAGGGCCAAGGCAGATACAGCCTTCACTGGGAGAAGGCACCTGTGGGTGCCCTGCCCT
  GACCCAGCAATGAAGACATTGCAGAGACAAAGTCAGAAGGAATTGTCCCACTAGTGGGAA
  CAACATAGCATACACTGCCTATGAGGTCCACTCAAGGAGGGCTTCCAGAAGGAGGTAAAG
  CTAGACCCCGCCCTTCCACATGTGGGGTAGGCATAGGATGTTGAGACTGTAAGAGACATC
  TCTTTGGCCCTCCTTGTATAGGGTGTCAATCGGCACAACAGGGTGGAGCCTTAGAGTAGG
  GTAAGATTAGGACTCTAGGTTCTCTCATGGGTCCAGATCT
  --------------------

Primer pair 2, product size : 919
  Primer left                            Primer right
  start: 25210133                        start: 25211030
  end  : 25210153                        end  : 25211051
  tm   : 57                              tm   : 61
  seq  : TTCTGGCTGATGGTGTCATAG           seq  : AGATCTGGACCCATGAGAGAAC

  >48_primer_pair_2_product
  TTCTGGCTGATGGTGTCATAGGCTGGGAAGAGAGAGGCCAGGAGAAAAGGCTGAGGAAAC
  TGCTGGCAAATGTGAAGGGCAAGAATGAATGCCCAAGGTGGGCAGCAGGTGAGGAAAGAG
  TCCCTCACCTCCCTGGAGGAACAAGTCTTTGATTTGCTGAAAGGCATCCCGCACAGCCTG
  GGCGCACTTGGGACTCTGGCCATAAAAGTCCTGGAGAAGAGACCAAGGTTGCTGCTGCCA
  TTCTTGCACTGGCCTGGGGTACCCAAGTCCCCTCACTCACCGCTGTGACATCTCGGAAGA
  ATTGGTAGGAGTCCCCAAGGCCTGCAACAGCTACAACAGGAGCGCTGGCTGCCAGTGCCC
  CAGCCACCAGGTGGGGGTACTTCATCCTCATGTAGGCACTCAGCATCCCCCCATAACTGG
  GAGTACAGAGCACAGATCATGGTTGTGGGAAGCTGCCCACAACTCAGGCGAGCAGCCTCA
  CTGTCCTCCAGGCTGAGGTGCTAGGCTGCTCTTTCCCTGCTCAGAACGCCCAAGGGTGGG
  AAAGAAGGACCTGAAACTGTCAGGCCCACACACCCTGATCCCAGGGCCAAGGCAGATACA
  GCCTTCACTGGGAGAAGGCACCTGTGGGTGCCCTGCCCTGACCCAGCAATGAAGACATTG
  CAGAGACAAAGTCAGAAGGAATTGTCCCACTAGTGGGAACAACATAGCATACACTGCCTA
  TGAGGTCCACTCAAGGAGGGCTTCCAGAAGGAGGTAAAGCTAGACCCCGCCCTTCCACAT
  GTGGGGTAGGCATAGGATGTTGAGACTGTAAGAGACATCTCTTTGGCCCTCCTTGTATAG
  GGTGTCAATCGGCACAACAGGGTGGAGCCTTAGAGTAGGGTAAGATTAGGACTCTAGGTT
  CTCTCATGGGTCCAGATCT
  ============================================================

Primers for ID: 59
Primer pair 1, product size : 939
  Primer left                            Primer right
  start: 25211383                        start: 25212301
  end  : 25211403                        end  : 25212321
  tm   : 57                              tm   : 57
  seq  : ATGAAGCCAGAGTTGTTAGCG           seq  : TAATTATGGGCCTGTCTGGTG

  >59_primer_pair_1_product
  ATGAAGCCAGAGTTGTTAGCGAAGGACCAGATATCCCCCTCATTCCCTGTGTAGAAAAAG
  ATGGGCCCTTCGCCCATCTTCCAGAACTTATCTGTTGGAAGTAAATGAGTTTCCATAAGG
  CCAGGGAAACGCAGGTAGGAACCCATGCGGTCGAGCCAGCACTCACCTGACACTAGGAAC
  CGCTGGCCAAAGGTTTTGTTGCCGAAACTCTCAAAGTTGAAATGGTCCATGTATTGCTCA
  AAATAATTCTCATGAAAGTCAGGGTCTAGAACTCTGTCGGCTGAGGGCAGGTGCAGAGAC
  TCAGGAGCTGGTTGGGATCATCAGGGATCTAGGCGGGTCAGGAGGAAGGGCAGCCAGTCT
  GTACTCACCTCTGGCCTGGAGGTTGCACAGTCCCAGTGACAGCAGCAGGACCAGGATCCA
  GGAGGGGACACCATGGTCCACAGGGTAACAAGGATGGAAGTTCATGCTTGATTCTGAGCC
  GGGCGCTGACTGTCATGTGATTTGGTCACATGACCGACACAACGGGCGGGGCAGCATCAC
  GTGATAGTCTGGCGGGGGCTGTCCTACTGTGGCTGGATTCTAGTTGGAGGATCAGCCTAC
  TCTTCTTCAGTTTCCCGGTTCCTCCAAATTTCTGGGCTCCTACTTGTTTCCACAGAGATG
  GATACTGTGGAGGTCCAGGAAGCAGAGAGATGGCTAAGGCTCATCAGGACCGTATGATCT
  CCCAAGTGTCCAGCTACTGAGTACCACAAGGTGATGGGTGGGAGGGTCCTCCCACGGAAG
  GATACCGCAGTCCCTAGGGGTTGCAAGCCCCACATGTTCCACTGGCTGCTAGAGCTACCT
  ACTCAATCAGCCCTGGGCATCACCATCAGGTACTCGGCCAAAATGACCTCTCTGCTTCCA
  GTCCTCAGTTCTGGTCAGCACCAGACAGGCCCATAATTA
  --------------------

Primer pair 2, product size : 941
  Primer left                            Primer right
  start: 25211383                        start: 25212303
  end  : 25211403                        end  : 25212323
  tm   : 57                              tm   : 57
  seq  : ATGAAGCCAGAGTTGTTAGCG           seq  : TGTAATTATGGGCCTGTCTGG

  >59_primer_pair_2_product
  ATGAAGCCAGAGTTGTTAGCGAAGGACCAGATATCCCCCTCATTCCCTGTGTAGAAAAAG
  ATGGGCCCTTCGCCCATCTTCCAGAACTTATCTGTTGGAAGTAAATGAGTTTCCATAAGG
  CCAGGGAAACGCAGGTAGGAACCCATGCGGTCGAGCCAGCACTCACCTGACACTAGGAAC
  CGCTGGCCAAAGGTTTTGTTGCCGAAACTCTCAAAGTTGAAATGGTCCATGTATTGCTCA
  AAATAATTCTCATGAAAGTCAGGGTCTAGAACTCTGTCGGCTGAGGGCAGGTGCAGAGAC
  TCAGGAGCTGGTTGGGATCATCAGGGATCTAGGCGGGTCAGGAGGAAGGGCAGCCAGTCT
  GTACTCACCTCTGGCCTGGAGGTTGCACAGTCCCAGTGACAGCAGCAGGACCAGGATCCA
  GGAGGGGACACCATGGTCCACAGGGTAACAAGGATGGAAGTTCATGCTTGATTCTGAGCC
  GGGCGCTGACTGTCATGTGATTTGGTCACATGACCGACACAACGGGCGGGGCAGCATCAC
  GTGATAGTCTGGCGGGGGCTGTCCTACTGTGGCTGGATTCTAGTTGGAGGATCAGCCTAC
  TCTTCTTCAGTTTCCCGGTTCCTCCAAATTTCTGGGCTCCTACTTGTTTCCACAGAGATG
  GATACTGTGGAGGTCCAGGAAGCAGAGAGATGGCTAAGGCTCATCAGGACCGTATGATCT
  CCCAAGTGTCCAGCTACTGAGTACCACAAGGTGATGGGTGGGAGGGTCCTCCCACGGAAG
  GATACCGCAGTCCCTAGGGGTTGCAAGCCCCACATGTTCCACTGGCTGCTAGAGCTACCT
  ACTCAATCAGCCCTGGGCATCACCATCAGGTACTCGGCCAAAATGACCTCTCTGCTTCCA
  GTCCTCAGTTCTGGTCAGCACCAGACAGGCCCATAATTACA
  ============================================================

Primers for ID: 60
Primer pair 1, product size : 939
  Primer left                            Primer right
  start: 25211383                        start: 25212301
  end  : 25211403                        end  : 25212321
  tm   : 57                              tm   : 57
  seq  : ATGAAGCCAGAGTTGTTAGCG           seq  : TAATTATGGGCCTGTCTGGTG

  >60_primer_pair_1_product
  ATGAAGCCAGAGTTGTTAGCGAAGGACCAGATATCCCCCTCATTCCCTGTGTAGAAAAAG
  ATGGGCCCTTCGCCCATCTTCCAGAACTTATCTGTTGGAAGTAAATGAGTTTCCATAAGG
  CCAGGGAAACGCAGGTAGGAACCCATGCGGTCGAGCCAGCACTCACCTGACACTAGGAAC
  CGCTGGCCAAAGGTTTTGTTGCCGAAACTCTCAAAGTTGAAATGGTCCATGTATTGCTCA
  AAATAATTCTCATGAAAGTCAGGGTCTAGAACTCTGTCGGCTGAGGGCAGGTGCAGAGAC
  TCAGGAGCTGGTTGGGATCATCAGGGATCTAGGCGGGTCAGGAGGAAGGGCAGCCAGTCT
  GTACTCACCTCTGGCCTGGAGGTTGCACAGTCCCAGTGACAGCAGCAGGACCAGGATCCA
  GGAGGGGACACCATGGTCCACAGGGTAACAAGGATGGAAGTTCATGCTTGATTCTGAGCC
  GGGCGCTGACTGTCATGTGATTTGGTCACATGACCGACACAACGGGCGGGGCAGCATCAC
  GTGATAGTCTGGCGGGGGCTGTCCTACTGTGGCTGGATTCTAGTTGGAGGATCAGCCTAC
  TCTTCTTCAGTTTCCCGGTTCCTCCAAATTTCTGGGCTCCTACTTGTTTCCACAGAGATG
  GATACTGTGGAGGTCCAGGAAGCAGAGAGATGGCTAAGGCTCATCAGGACCGTATGATCT
  CCCAAGTGTCCAGCTACTGAGTACCACAAGGTGATGGGTGGGAGGGTCCTCCCACGGAAG
  GATACCGCAGTCCCTAGGGGTTGCAAGCCCCACATGTTCCACTGGCTGCTAGAGCTACCT
  ACTCAATCAGCCCTGGGCATCACCATCAGGTACTCGGCCAAAATGACCTCTCTGCTTCCA
  GTCCTCAGTTCTGGTCAGCACCAGACAGGCCCATAATTA
  --------------------

Primer pair 2, product size : 941
  Primer left                            Primer right
  start: 25211383                        start: 25212303
  end  : 25211403                        end  : 25212323
  tm   : 57                              tm   : 57
  seq  : ATGAAGCCAGAGTTGTTAGCG           seq  : TGTAATTATGGGCCTGTCTGG

  >60_primer_pair_2_product
  ATGAAGCCAGAGTTGTTAGCGAAGGACCAGATATCCCCCTCATTCCCTGTGTAGAAAAAG
  ATGGGCCCTTCGCCCATCTTCCAGAACTTATCTGTTGGAAGTAAATGAGTTTCCATAAGG
  CCAGGGAAACGCAGGTAGGAACCCATGCGGTCGAGCCAGCACTCACCTGACACTAGGAAC
  CGCTGGCCAAAGGTTTTGTTGCCGAAACTCTCAAAGTTGAAATGGTCCATGTATTGCTCA
  AAATAATTCTCATGAAAGTCAGGGTCTAGAACTCTGTCGGCTGAGGGCAGGTGCAGAGAC
  TCAGGAGCTGGTTGGGATCATCAGGGATCTAGGCGGGTCAGGAGGAAGGGCAGCCAGTCT
  GTACTCACCTCTGGCCTGGAGGTTGCACAGTCCCAGTGACAGCAGCAGGACCAGGATCCA
  GGAGGGGACACCATGGTCCACAGGGTAACAAGGATGGAAGTTCATGCTTGATTCTGAGCC
  GGGCGCTGACTGTCATGTGATTTGGTCACATGACCGACACAACGGGCGGGGCAGCATCAC
  GTGATAGTCTGGCGGGGGCTGTCCTACTGTGGCTGGATTCTAGTTGGAGGATCAGCCTAC
  TCTTCTTCAGTTTCCCGGTTCCTCCAAATTTCTGGGCTCCTACTTGTTTCCACAGAGATG
  GATACTGTGGAGGTCCAGGAAGCAGAGAGATGGCTAAGGCTCATCAGGACCGTATGATCT
  CCCAAGTGTCCAGCTACTGAGTACCACAAGGTGATGGGTGGGAGGGTCCTCCCACGGAAG
  GATACCGCAGTCCCTAGGGGTTGCAAGCCCCACATGTTCCACTGGCTGCTAGAGCTACCT
  ACTCAATCAGCCCTGGGCATCACCATCAGGTACTCGGCCAAAATGACCTCTCTGCTTCCA
  GTCCTCAGTTCTGGTCAGCACCAGACAGGCCCATAATTACA
  ============================================================

Primers for ID: 61
Primer pair 1, product size : 953
  Primer left                            Primer right
  start: 25211485                        start: 25212417
  end  : 25211506                        end  : 25212437
  tm   : 57                              tm   : 57
  seq  : AAATGAGTTTCCATAAGGCCAG          seq  : AGATTCCCAGAAAGTCCAAGG

  >61_primer_pair_1_product
  AAATGAGTTTCCATAAGGCCAGGGAAACGCAGGTAGGAACCCATGCGGTCGAGCCAGCAC
  TCACCTGACACTAGGAACCGCTGGCCAAAGGTTTTGTTGCCGAAACTCTCAAAGTTGAAA
  TGGTCCATGTATTGCTCAAAATAATTCTCATGAAAGTCAGGGTCTAGAACTCTGTCGGCT
  GAGGGCAGGTGCAGAGACTCAGGAGCTGGTTGGGATCATCAGGGATCTAGGCGGGTCAGG
  AGGAAGGGCAGCCAGTCTGTACTCACCTCTGGCCTGGAGGTTGCACAGTCCCAGTGACAG
  CAGCAGGACCAGGATCCAGGAGGGGACACCATGGTCCACAGGGTAACAAGGATGGAAGTT
  CATGCTTGATTCTGAGCCGGGCGCTGACTGTCATGTGATTTGGTCACATGACCGACACAA
  CGGGCGGGGCAGCATCACGTGATAGTCTGGCGGGGGCTGTCCTACTGTGGCTGGATTCTA
  GTTGGAGGATCAGCCTACTCTTCTTCAGTTTCCCGGTTCCTCCAAATTTCTGGGCTCCTA
  CTTGTTTCCACAGAGATGGATACTGTGGAGGTCCAGGAAGCAGAGAGATGGCTAAGGCTC
  ATCAGGACCGTATGATCTCCCAAGTGTCCAGCTACTGAGTACCACAAGGTGATGGGTGGG
  AGGGTCCTCCCACGGAAGGATACCGCAGTCCCTAGGGGTTGCAAGCCCCACATGTTCCAC
  TGGCTGCTAGAGCTACCTACTCAATCAGCCCTGGGCATCACCATCAGGTACTCGGCCAAA
  ATGACCTCTCTGCTTCCAGTCCTCAGTTCTGGTCAGCACCAGACAGGCCCATAATTACAG
  AGCCAGGGAAACTGGAACATTTGTCTCCCCTTAGACAGTGGCAGCAGGAAGGTGGGGGGT
  TGTTGCAGAGGAACAGTGTCTCTGAGAGAGGACCTTGGACTTTCTGGGAATCT
  --------------------

Primer pair 2, product size : 951
  Primer left                            Primer right
  start: 25211485                        start: 25212415
  end  : 25211506                        end  : 25212435
  tm   : 57                              tm   : 57
  seq  : AAATGAGTTTCCATAAGGCCAG          seq  : ATTCCCAGAAAGTCCAAGGTC

  >61_primer_pair_2_product
  AAATGAGTTTCCATAAGGCCAGGGAAACGCAGGTAGGAACCCATGCGGTCGAGCCAGCAC
  TCACCTGACACTAGGAACCGCTGGCCAAAGGTTTTGTTGCCGAAACTCTCAAAGTTGAAA
  TGGTCCATGTATTGCTCAAAATAATTCTCATGAAAGTCAGGGTCTAGAACTCTGTCGGCT
  GAGGGCAGGTGCAGAGACTCAGGAGCTGGTTGGGATCATCAGGGATCTAGGCGGGTCAGG
  AGGAAGGGCAGCCAGTCTGTACTCACCTCTGGCCTGGAGGTTGCACAGTCCCAGTGACAG
  CAGCAGGACCAGGATCCAGGAGGGGACACCATGGTCCACAGGGTAACAAGGATGGAAGTT
  CATGCTTGATTCTGAGCCGGGCGCTGACTGTCATGTGATTTGGTCACATGACCGACACAA
  CGGGCGGGGCAGCATCACGTGATAGTCTGGCGGGGGCTGTCCTACTGTGGCTGGATTCTA
  GTTGGAGGATCAGCCTACTCTTCTTCAGTTTCCCGGTTCCTCCAAATTTCTGGGCTCCTA
  CTTGTTTCCACAGAGATGGATACTGTGGAGGTCCAGGAAGCAGAGAGATGGCTAAGGCTC
  ATCAGGACCGTATGATCTCCCAAGTGTCCAGCTACTGAGTACCACAAGGTGATGGGTGGG
  AGGGTCCTCCCACGGAAGGATACCGCAGTCCCTAGGGGTTGCAAGCCCCACATGTTCCAC
  TGGCTGCTAGAGCTACCTACTCAATCAGCCCTGGGCATCACCATCAGGTACTCGGCCAAA
  ATGACCTCTCTGCTTCCAGTCCTCAGTTCTGGTCAGCACCAGACAGGCCCATAATTACAG
  AGCCAGGGAAACTGGAACATTTGTCTCCCCTTAGACAGTGGCAGCAGGAAGGTGGGGGGT
  TGTTGCAGAGGAACAGTGTCTCTGAGAGAGGACCTTGGACTTTCTGGGAAT
  ============================================================

Primers for ID: 62
Primer pair 1, product size : 865
  Primer left                            Primer right
  start: 25211573                        start: 25212417
  end  : 25211592                        end  : 25212437
  tm   : 53                              tm   : 57
  seq  : AGGTTTTGTTGCCGAAACTC            seq  : AGATTCCCAGAAAGTCCAAGG

  >62_primer_pair_1_product
  AGGTTTTGTTGCCGAAACTCTCAAAGTTGAAATGGTCCATGTATTGCTCAAAATAATTCT
  CATGAAAGTCAGGGTCTAGAACTCTGTCGGCTGAGGGCAGGTGCAGAGACTCAGGAGCTG
  GTTGGGATCATCAGGGATCTAGGCGGGTCAGGAGGAAGGGCAGCCAGTCTGTACTCACCT
  CTGGCCTGGAGGTTGCACAGTCCCAGTGACAGCAGCAGGACCAGGATCCAGGAGGGGACA
  CCATGGTCCACAGGGTAACAAGGATGGAAGTTCATGCTTGATTCTGAGCCGGGCGCTGAC
  TGTCATGTGATTTGGTCACATGACCGACACAACGGGCGGGGCAGCATCACGTGATAGTCT
  GGCGGGGGCTGTCCTACTGTGGCTGGATTCTAGTTGGAGGATCAGCCTACTCTTCTTCAG
  TTTCCCGGTTCCTCCAAATTTCTGGGCTCCTACTTGTTTCCACAGAGATGGATACTGTGG
  AGGTCCAGGAAGCAGAGAGATGGCTAAGGCTCATCAGGACCGTATGATCTCCCAAGTGTC
  CAGCTACTGAGTACCACAAGGTGATGGGTGGGAGGGTCCTCCCACGGAAGGATACCGCAG
  TCCCTAGGGGTTGCAAGCCCCACATGTTCCACTGGCTGCTAGAGCTACCTACTCAATCAG
  CCCTGGGCATCACCATCAGGTACTCGGCCAAAATGACCTCTCTGCTTCCAGTCCTCAGTT
  CTGGTCAGCACCAGACAGGCCCATAATTACAGAGCCAGGGAAACTGGAACATTTGTCTCC
  CCTTAGACAGTGGCAGCAGGAAGGTGGGGGGTTGTTGCAGAGGAACAGTGTCTCTGAGAG
  AGGACCTTGGACTTTCTGGGAATCT
  --------------------

Primer pair 2, product size : 863
  Primer left                            Primer right
  start: 25211573                        start: 25212415
  end  : 25211592                        end  : 25212435
  tm   : 53                              tm   : 57
  seq  : AGGTTTTGTTGCCGAAACTC            seq  : ATTCCCAGAAAGTCCAAGGTC

  >62_primer_pair_2_product
  AGGTTTTGTTGCCGAAACTCTCAAAGTTGAAATGGTCCATGTATTGCTCAAAATAATTCT
  CATGAAAGTCAGGGTCTAGAACTCTGTCGGCTGAGGGCAGGTGCAGAGACTCAGGAGCTG
  GTTGGGATCATCAGGGATCTAGGCGGGTCAGGAGGAAGGGCAGCCAGTCTGTACTCACCT
  CTGGCCTGGAGGTTGCACAGTCCCAGTGACAGCAGCAGGACCAGGATCCAGGAGGGGACA
  CCATGGTCCACAGGGTAACAAGGATGGAAGTTCATGCTTGATTCTGAGCCGGGCGCTGAC
  TGTCATGTGATTTGGTCACATGACCGACACAACGGGCGGGGCAGCATCACGTGATAGTCT
  GGCGGGGGCTGTCCTACTGTGGCTGGATTCTAGTTGGAGGATCAGCCTACTCTTCTTCAG
  TTTCCCGGTTCCTCCAAATTTCTGGGCTCCTACTTGTTTCCACAGAGATGGATACTGTGG
  AGGTCCAGGAAGCAGAGAGATGGCTAAGGCTCATCAGGACCGTATGATCTCCCAAGTGTC
  CAGCTACTGAGTACCACAAGGTGATGGGTGGGAGGGTCCTCCCACGGAAGGATACCGCAG
  TCCCTAGGGGTTGCAAGCCCCACATGTTCCACTGGCTGCTAGAGCTACCTACTCAATCAG
  CCCTGGGCATCACCATCAGGTACTCGGCCAAAATGACCTCTCTGCTTCCAGTCCTCAGTT
  CTGGTCAGCACCAGACAGGCCCATAATTACAGAGCCAGGGAAACTGGAACATTTGTCTCC
  CCTTAGACAGTGGCAGCAGGAAGGTGGGGGGTTGTTGCAGAGGAACAGTGTCTCTGAGAG
  AGGACCTTGGACTTTCTGGGAAT
  ============================================================

Primers for ID: 63
Primer pair 1, product size : 881
  Primer left                            Primer right
  start: 25209909                        start: 25210770
  end  : 25209929                        end  : 25210789
  tm   : 57                              tm   : 55
  seq  : GATGGCATGAAAGCAGATAGC           seq  : TGTCTTCATTGCTGGGTCAG

  >63_primer_pair_1_product
  GATGGCATGAAAGCAGATAGCTACAAGGCTCTTGGACCAGGCTAGCACTGGGTCCTGCAC
  CCAGGGAGAGCCACCTCACCTTGACAGGGTTGGCAGGAAGGGGCCCTAGAAAGTCAGTAG
  GATACGGGTAGTCCATCATGGCGAGCACAGTAAATGCATTTCGGGCAAACCCAAAGAGCT
  GAGTCAGGTCCTTTGGGCTGGAAAGTGATTGACAGGTACCAAAGTTCTGGCTGATGGTGT
  CATAGGCTGGGAAGAGAGAGGCCAGGAGAAAAGGCTGAGGAAACTGCTGGCAAATGTGAA
  GGGCAAGAATGAATGCCCAAGGTGGGCAGCAGGTGAGGAAAGAGTCCCTCACCTCCCTGG
  AGGAACAAGTCTTTGATTTGCTGAAAGGCATCCCGCACAGCCTGGGCGCACTTGGGACTC
  TGGCCATAAAAGTCCTGGAGAAGAGACCAAGGTTGCTGCTGCCATTCTTGCACTGGCCTG
  GGGTACCCAAGTCCCCTCACTCACCGCTGTGACATCTCGGAAGAATTGGTAGGAGTCCCC
  AAGGCCTGCAACAGCTACAACAGGAGCGCTGGCTGCCAGTGCCCCAGCCACCAGGTGGGG
  GTACTTCATCCTCATGTAGGCACTCAGCATCCCCCCATAACTGGGAGTACAGAGCACAGA
  TCATGGTTGTGGGAAGCTGCCCACAACTCAGGCGAGCAGCCTCACTGTCCTCCAGGCTGA
  GGTGCTAGGCTGCTCTTTCCCTGCTCAGAACGCCCAAGGGTGGGAAAGAAGGACCTGAAA
  CTGTCAGGCCCACACACCCTGATCCCAGGGCCAAGGCAGATACAGCCTTCACTGGGAGAA
  GGCACCTGTGGGTGCCCTGCCCTGACCCAGCAATGAAGACA
  --------------------

Primer pair 2, product size : 915
  Primer left                            Primer right
  start: 25209909                        start: 25210803
  end  : 25209929                        end  : 25210823
  tm   : 57                              tm   : 57
  seq  : GATGGCATGAAAGCAGATAGC           seq  : AGTGGGACAATTCCTTCTGAC

  >63_primer_pair_2_product
  GATGGCATGAAAGCAGATAGCTACAAGGCTCTTGGACCAGGCTAGCACTGGGTCCTGCAC
  CCAGGGAGAGCCACCTCACCTTGACAGGGTTGGCAGGAAGGGGCCCTAGAAAGTCAGTAG
  GATACGGGTAGTCCATCATGGCGAGCACAGTAAATGCATTTCGGGCAAACCCAAAGAGCT
  GAGTCAGGTCCTTTGGGCTGGAAAGTGATTGACAGGTACCAAAGTTCTGGCTGATGGTGT
  CATAGGCTGGGAAGAGAGAGGCCAGGAGAAAAGGCTGAGGAAACTGCTGGCAAATGTGAA
  GGGCAAGAATGAATGCCCAAGGTGGGCAGCAGGTGAGGAAAGAGTCCCTCACCTCCCTGG
  AGGAACAAGTCTTTGATTTGCTGAAAGGCATCCCGCACAGCCTGGGCGCACTTGGGACTC
  TGGCCATAAAAGTCCTGGAGAAGAGACCAAGGTTGCTGCTGCCATTCTTGCACTGGCCTG
  GGGTACCCAAGTCCCCTCACTCACCGCTGTGACATCTCGGAAGAATTGGTAGGAGTCCCC
  AAGGCCTGCAACAGCTACAACAGGAGCGCTGGCTGCCAGTGCCCCAGCCACCAGGTGGGG
  GTACTTCATCCTCATGTAGGCACTCAGCATCCCCCCATAACTGGGAGTACAGAGCACAGA
  TCATGGTTGTGGGAAGCTGCCCACAACTCAGGCGAGCAGCCTCACTGTCCTCCAGGCTGA
  GGTGCTAGGCTGCTCTTTCCCTGCTCAGAACGCCCAAGGGTGGGAAAGAAGGACCTGAAA
  CTGTCAGGCCCACACACCCTGATCCCAGGGCCAAGGCAGATACAGCCTTCACTGGGAGAA
  GGCACCTGTGGGTGCCCTGCCCTGACCCAGCAATGAAGACATTGCAGAGACAAAGTCAGA
  AGGAATTGTCCCACT
  ============================================================

Primers for ID: 64
Primer pair 1, product size : 881
  Primer left                            Primer right
  start: 25209909                        start: 25210770
  end  : 25209929                        end  : 25210789
  tm   : 57                              tm   : 55
  seq  : GATGGCATGAAAGCAGATAGC           seq  : TGTCTTCATTGCTGGGTCAG

  >64_primer_pair_1_product
  GATGGCATGAAAGCAGATAGCTACAAGGCTCTTGGACCAGGCTAGCACTGGGTCCTGCAC
  CCAGGGAGAGCCACCTCACCTTGACAGGGTTGGCAGGAAGGGGCCCTAGAAAGTCAGTAG
  GATACGGGTAGTCCATCATGGCGAGCACAGTAAATGCATTTCGGGCAAACCCAAAGAGCT
  GAGTCAGGTCCTTTGGGCTGGAAAGTGATTGACAGGTACCAAAGTTCTGGCTGATGGTGT
  CATAGGCTGGGAAGAGAGAGGCCAGGAGAAAAGGCTGAGGAAACTGCTGGCAAATGTGAA
  GGGCAAGAATGAATGCCCAAGGTGGGCAGCAGGTGAGGAAAGAGTCCCTCACCTCCCTGG
  AGGAACAAGTCTTTGATTTGCTGAAAGGCATCCCGCACAGCCTGGGCGCACTTGGGACTC
  TGGCCATAAAAGTCCTGGAGAAGAGACCAAGGTTGCTGCTGCCATTCTTGCACTGGCCTG
  GGGTACCCAAGTCCCCTCACTCACCGCTGTGACATCTCGGAAGAATTGGTAGGAGTCCCC
  AAGGCCTGCAACAGCTACAACAGGAGCGCTGGCTGCCAGTGCCCCAGCCACCAGGTGGGG
  GTACTTCATCCTCATGTAGGCACTCAGCATCCCCCCATAACTGGGAGTACAGAGCACAGA
  TCATGGTTGTGGGAAGCTGCCCACAACTCAGGCGAGCAGCCTCACTGTCCTCCAGGCTGA
  GGTGCTAGGCTGCTCTTTCCCTGCTCAGAACGCCCAAGGGTGGGAAAGAAGGACCTGAAA
  CTGTCAGGCCCACACACCCTGATCCCAGGGCCAAGGCAGATACAGCCTTCACTGGGAGAA
  GGCACCTGTGGGTGCCCTGCCCTGACCCAGCAATGAAGACA
  --------------------

Primer pair 2, product size : 915
  Primer left                            Primer right
  start: 25209909                        start: 25210803
  end  : 25209929                        end  : 25210823
  tm   : 57                              tm   : 57
  seq  : GATGGCATGAAAGCAGATAGC           seq  : AGTGGGACAATTCCTTCTGAC

  >64_primer_pair_2_product
  GATGGCATGAAAGCAGATAGCTACAAGGCTCTTGGACCAGGCTAGCACTGGGTCCTGCAC
  CCAGGGAGAGCCACCTCACCTTGACAGGGTTGGCAGGAAGGGGCCCTAGAAAGTCAGTAG
  GATACGGGTAGTCCATCATGGCGAGCACAGTAAATGCATTTCGGGCAAACCCAAAGAGCT
  GAGTCAGGTCCTTTGGGCTGGAAAGTGATTGACAGGTACCAAAGTTCTGGCTGATGGTGT
  CATAGGCTGGGAAGAGAGAGGCCAGGAGAAAAGGCTGAGGAAACTGCTGGCAAATGTGAA
  GGGCAAGAATGAATGCCCAAGGTGGGCAGCAGGTGAGGAAAGAGTCCCTCACCTCCCTGG
  AGGAACAAGTCTTTGATTTGCTGAAAGGCATCCCGCACAGCCTGGGCGCACTTGGGACTC
  TGGCCATAAAAGTCCTGGAGAAGAGACCAAGGTTGCTGCTGCCATTCTTGCACTGGCCTG
  GGGTACCCAAGTCCCCTCACTCACCGCTGTGACATCTCGGAAGAATTGGTAGGAGTCCCC
  AAGGCCTGCAACAGCTACAACAGGAGCGCTGGCTGCCAGTGCCCCAGCCACCAGGTGGGG
  GTACTTCATCCTCATGTAGGCACTCAGCATCCCCCCATAACTGGGAGTACAGAGCACAGA
  TCATGGTTGTGGGAAGCTGCCCACAACTCAGGCGAGCAGCCTCACTGTCCTCCAGGCTGA
  GGTGCTAGGCTGCTCTTTCCCTGCTCAGAACGCCCAAGGGTGGGAAAGAAGGACCTGAAA
  CTGTCAGGCCCACACACCCTGATCCCAGGGCCAAGGCAGATACAGCCTTCACTGGGAGAA
  GGCACCTGTGGGTGCCCTGCCCTGACCCAGCAATGAAGACATTGCAGAGACAAAGTCAGA
  AGGAATTGTCCCACT
  ============================================================

Primers for ID: 65
Primer pair 1, product size : 843
  Primer left                            Primer right
  start: 25210129                        start: 25210951
  end  : 25210149                        end  : 25210971
  tm   : 57                              tm   : 57
  seq  : AAAGTTCTGGCTGATGGTGTC           seq  : TATACAAGGAGGGCCAAAGAG

  >65_primer_pair_1_product
  AAAGTTCTGGCTGATGGTGTCATAGGCTGGGAAGAGAGAGGCCAGGAGAAAAGGCTGAGG
  AAACTGCTGGCAAATGTGAAGGGCAAGAATGAATGCCCAAGGTGGGCAGCAGGTGAGGAA
  AGAGTCCCTCACCTCCCTGGAGGAACAAGTCTTTGATTTGCTGAAAGGCATCCCGCACAG
  CCTGGGCGCACTTGGGACTCTGGCCATAAAAGTCCTGGAGAAGAGACCAAGGTTGCTGCT
  GCCATTCTTGCACTGGCCTGGGGTACCCAAGTCCCCTCACTCACCGCTGTGACATCTCGG
  AAGAATTGGTAGGAGTCCCCAAGGCCTGCAACAGCTACAACAGGAGCGCTGGCTGCCAGT
  GCCCCAGCCACCAGGTGGGGGTACTTCATCCTCATGTAGGCACTCAGCATCCCCCCATAA
  CTGGGAGTACAGAGCACAGATCATGGTTGTGGGAAGCTGCCCACAACTCAGGCGAGCAGC
  CTCACTGTCCTCCAGGCTGAGGTGCTAGGCTGCTCTTTCCCTGCTCAGAACGCCCAAGGG
  TGGGAAAGAAGGACCTGAAACTGTCAGGCCCACACACCCTGATCCCAGGGCCAAGGCAGA
  TACAGCCTTCACTGGGAGAAGGCACCTGTGGGTGCCCTGCCCTGACCCAGCAATGAAGAC
  ATTGCAGAGACAAAGTCAGAAGGAATTGTCCCACTAGTGGGAACAACATAGCATACACTG
  CCTATGAGGTCCACTCAAGGAGGGCTTCCAGAAGGAGGTAAAGCTAGACCCCGCCCTTCC
  ACATGTGGGGTAGGCATAGGATGTTGAGACTGTAAGAGACATCTCTTTGGCCCTCCTTGT
  ATA
  --------------------

Primer pair 2, product size : 880
  Primer left                            Primer right
  start: 25210172                        start: 25211030
  end  : 25210192                        end  : 25211051
  tm   : 57                              tm   : 61
  seq  : AGGAGAAAAGGCTGAGGAAAC           seq  : AGATCTGGACCCATGAGAGAAC

  >65_primer_pair_2_product
  AGGAGAAAAGGCTGAGGAAACTGCTGGCAAATGTGAAGGGCAAGAATGAATGCCCAAGGT
  GGGCAGCAGGTGAGGAAAGAGTCCCTCACCTCCCTGGAGGAACAAGTCTTTGATTTGCTG
  AAAGGCATCCCGCACAGCCTGGGCGCACTTGGGACTCTGGCCATAAAAGTCCTGGAGAAG
  AGACCAAGGTTGCTGCTGCCATTCTTGCACTGGCCTGGGGTACCCAAGTCCCCTCACTCA
  CCGCTGTGACATCTCGGAAGAATTGGTAGGAGTCCCCAAGGCCTGCAACAGCTACAACAG
  GAGCGCTGGCTGCCAGTGCCCCAGCCACCAGGTGGGGGTACTTCATCCTCATGTAGGCAC
  TCAGCATCCCCCCATAACTGGGAGTACAGAGCACAGATCATGGTTGTGGGAAGCTGCCCA
  CAACTCAGGCGAGCAGCCTCACTGTCCTCCAGGCTGAGGTGCTAGGCTGCTCTTTCCCTG
  CTCAGAACGCCCAAGGGTGGGAAAGAAGGACCTGAAACTGTCAGGCCCACACACCCTGAT
  CCCAGGGCCAAGGCAGATACAGCCTTCACTGGGAGAAGGCACCTGTGGGTGCCCTGCCCT
  GACCCAGCAATGAAGACATTGCAGAGACAAAGTCAGAAGGAATTGTCCCACTAGTGGGAA
  CAACATAGCATACACTGCCTATGAGGTCCACTCAAGGAGGGCTTCCAGAAGGAGGTAAAG
  CTAGACCCCGCCCTTCCACATGTGGGGTAGGCATAGGATGTTGAGACTGTAAGAGACATC
  TCTTTGGCCCTCCTTGTATAGGGTGTCAATCGGCACAACAGGGTGGAGCCTTAGAGTAGG
  GTAAGATTAGGACTCTAGGTTCTCTCATGGGTCCAGATCT
  ============================================================

Primers for ID: 66
Primer pair 1, product size : 843
  Primer left                            Primer right
  start: 25210129                        start: 25210951
  end  : 25210149                        end  : 25210971
  tm   : 57                              tm   : 57
  seq  : AAAGTTCTGGCTGATGGTGTC           seq  : TATACAAGGAGGGCCAAAGAG

  >66_primer_pair_1_product
  AAAGTTCTGGCTGATGGTGTCATAGGCTGGGAAGAGAGAGGCCAGGAGAAAAGGCTGAGG
  AAACTGCTGGCAAATGTGAAGGGCAAGAATGAATGCCCAAGGTGGGCAGCAGGTGAGGAA
  AGAGTCCCTCACCTCCCTGGAGGAACAAGTCTTTGATTTGCTGAAAGGCATCCCGCACAG
  CCTGGGCGCACTTGGGACTCTGGCCATAAAAGTCCTGGAGAAGAGACCAAGGTTGCTGCT
  GCCATTCTTGCACTGGCCTGGGGTACCCAAGTCCCCTCACTCACCGCTGTGACATCTCGG
  AAGAATTGGTAGGAGTCCCCAAGGCCTGCAACAGCTACAACAGGAGCGCTGGCTGCCAGT
  GCCCCAGCCACCAGGTGGGGGTACTTCATCCTCATGTAGGCACTCAGCATCCCCCCATAA
  CTGGGAGTACAGAGCACAGATCATGGTTGTGGGAAGCTGCCCACAACTCAGGCGAGCAGC
  CTCACTGTCCTCCAGGCTGAGGTGCTAGGCTGCTCTTTCCCTGCTCAGAACGCCCAAGGG
  TGGGAAAGAAGGACCTGAAACTGTCAGGCCCACACACCCTGATCCCAGGGCCAAGGCAGA
  TACAGCCTTCACTGGGAGAAGGCACCTGTGGGTGCCCTGCCCTGACCCAGCAATGAAGAC
  ATTGCAGAGACAAAGTCAGAAGGAATTGTCCCACTAGTGGGAACAACATAGCATACACTG
  CCTATGAGGTCCACTCAAGGAGGGCTTCCAGAAGGAGGTAAAGCTAGACCCCGCCCTTCC
  ACATGTGGGGTAGGCATAGGATGTTGAGACTGTAAGAGACATCTCTTTGGCCCTCCTTGT
  ATA
  --------------------

Primer pair 2, product size : 880
  Primer left                            Primer right
  start: 25210172                        start: 25211030
  end  : 25210192                        end  : 25211051
  tm   : 57                              tm   : 61
  seq  : AGGAGAAAAGGCTGAGGAAAC           seq  : AGATCTGGACCCATGAGAGAAC

  >66_primer_pair_2_product
  AGGAGAAAAGGCTGAGGAAACTGCTGGCAAATGTGAAGGGCAAGAATGAATGCCCAAGGT
  GGGCAGCAGGTGAGGAAAGAGTCCCTCACCTCCCTGGAGGAACAAGTCTTTGATTTGCTG
  AAAGGCATCCCGCACAGCCTGGGCGCACTTGGGACTCTGGCCATAAAAGTCCTGGAGAAG
  AGACCAAGGTTGCTGCTGCCATTCTTGCACTGGCCTGGGGTACCCAAGTCCCCTCACTCA
  CCGCTGTGACATCTCGGAAGAATTGGTAGGAGTCCCCAAGGCCTGCAACAGCTACAACAG
  GAGCGCTGGCTGCCAGTGCCCCAGCCACCAGGTGGGGGTACTTCATCCTCATGTAGGCAC
  TCAGCATCCCCCCATAACTGGGAGTACAGAGCACAGATCATGGTTGTGGGAAGCTGCCCA
  CAACTCAGGCGAGCAGCCTCACTGTCCTCCAGGCTGAGGTGCTAGGCTGCTCTTTCCCTG
  CTCAGAACGCCCAAGGGTGGGAAAGAAGGACCTGAAACTGTCAGGCCCACACACCCTGAT
  CCCAGGGCCAAGGCAGATACAGCCTTCACTGGGAGAAGGCACCTGTGGGTGCCCTGCCCT
  GACCCAGCAATGAAGACATTGCAGAGACAAAGTCAGAAGGAATTGTCCCACTAGTGGGAA
  CAACATAGCATACACTGCCTATGAGGTCCACTCAAGGAGGGCTTCCAGAAGGAGGTAAAG
  CTAGACCCCGCCCTTCCACATGTGGGGTAGGCATAGGATGTTGAGACTGTAAGAGACATC
  TCTTTGGCCCTCCTTGTATAGGGTGTCAATCGGCACAACAGGGTGGAGCCTTAGAGTAGG
  GTAAGATTAGGACTCTAGGTTCTCTCATGGGTCCAGATCT
  ============================================================

Primers for ID: 76
Primer pair 1, product size : 830
  Primer left                            Primer right
  start: 25211173                        start: 25211982
  end  : 25211194                        end  : 25212002
  tm   : 61                              tm   : 57
  seq  : AGTCAGCAGCTGTGTATATCCC          seq  : AACCGGGAAACTGAAGAAGAG

  >76_primer_pair_1_product
  AGTCAGCAGCTGTGTATATCCCCGCTGTGTGGACTGGACACCGAACGGAAGCGATTTCCC
  ATAGTACCGCTGCAGAAAGCAGGAAGGGATGGCTAATCCACTCCTCGGTGCTCCCCACCT
  CCTTCAACTCAGGGACTGCCAGGAACTGTACAGGTACCCACGTGCTCAGCAAAGACAAGC
  AGGGCCTCCTGCTGGGCTGCCAGTTCCACCATGAAGCCAGAGTTGTTAGCGAAGGACCAG
  ATATCCCCCTCATTCCCTGTGTAGAAAAAGATGGGCCCTTCGCCCATCTTCCAGAACTTA
  TCTGTTGGAAGTAAATGAGTTTCCATAAGGCCAGGGAAACGCAGGTAGGAACCCATGCGG
  TCGAGCCAGCACTCACCTGACACTAGGAACCGCTGGCCAAAGGTTTTGTTGCCGAAACTC
  TCAAAGTTGAAATGGTCCATGTATTGCTCAAAATAATTCTCATGAAAGTCAGGGTCTAGA
  ACTCTGTCGGCTGAGGGCAGGTGCAGAGACTCAGGAGCTGGTTGGGATCATCAGGGATCT
  AGGCGGGTCAGGAGGAAGGGCAGCCAGTCTGTACTCACCTCTGGCCTGGAGGTTGCACAG
  TCCCAGTGACAGCAGCAGGACCAGGATCCAGGAGGGGACACCATGGTCCACAGGGTAACA
  AGGATGGAAGTTCATGCTTGATTCTGAGCCGGGCGCTGACTGTCATGTGATTTGGTCACA
  TGACCGACACAACGGGCGGGGCAGCATCACGTGATAGTCTGGCGGGGGCTGTCCTACTGT
  GGCTGGATTCTAGTTGGAGGATCAGCCTACTCTTCTTCAGTTTCCCGGTT
  --------------------

Primer pair 2, product size : 872
  Primer left                            Primer right
  start: 25211226                        start: 25212076
  end  : 25211245                        end  : 25212097
  tm   : 55                              tm   : 61
  seq  : ATTTCCCATAGTACCGCTGC            seq  : ATACGGTCCTGATGAGCCTTAG

  >76_primer_pair_2_product
  ATTTCCCATAGTACCGCTGCAGAAAGCAGGAAGGGATGGCTAATCCACTCCTCGGTGCTC
  CCCACCTCCTTCAACTCAGGGACTGCCAGGAACTGTACAGGTACCCACGTGCTCAGCAAA
  GACAAGCAGGGCCTCCTGCTGGGCTGCCAGTTCCACCATGAAGCCAGAGTTGTTAGCGAA
  GGACCAGATATCCCCCTCATTCCCTGTGTAGAAAAAGATGGGCCCTTCGCCCATCTTCCA
  GAACTTATCTGTTGGAAGTAAATGAGTTTCCATAAGGCCAGGGAAACGCAGGTAGGAACC
  CATGCGGTCGAGCCAGCACTCACCTGACACTAGGAACCGCTGGCCAAAGGTTTTGTTGCC
  GAAACTCTCAAAGTTGAAATGGTCCATGTATTGCTCAAAATAATTCTCATGAAAGTCAGG
  GTCTAGAACTCTGTCGGCTGAGGGCAGGTGCAGAGACTCAGGAGCTGGTTGGGATCATCA
  GGGATCTAGGCGGGTCAGGAGGAAGGGCAGCCAGTCTGTACTCACCTCTGGCCTGGAGGT
  TGCACAGTCCCAGTGACAGCAGCAGGACCAGGATCCAGGAGGGGACACCATGGTCCACAG
  GGTAACAAGGATGGAAGTTCATGCTTGATTCTGAGCCGGGCGCTGACTGTCATGTGATTT
  GGTCACATGACCGACACAACGGGCGGGGCAGCATCACGTGATAGTCTGGCGGGGGCTGTC
  CTACTGTGGCTGGATTCTAGTTGGAGGATCAGCCTACTCTTCTTCAGTTTCCCGGTTCCT
  CCAAATTTCTGGGCTCCTACTTGTTTCCACAGAGATGGATACTGTGGAGGTCCAGGAAGC
  AGAGAGATGGCTAAGGCTCATCAGGACCGTAT
  ============================================================

Primers for ID: 77
Primer pair 1, product size : 939
  Primer left                            Primer right
  start: 25211383                        start: 25212301
  end  : 25211403                        end  : 25212321
  tm   : 57                              tm   : 57
  seq  : ATGAAGCCAGAGTTGTTAGCG           seq  : TAATTATGGGCCTGTCTGGTG

  >77_primer_pair_1_product
  ATGAAGCCAGAGTTGTTAGCGAAGGACCAGATATCCCCCTCATTCCCTGTGTAGAAAAAG
  ATGGGCCCTTCGCCCATCTTCCAGAACTTATCTGTTGGAAGTAAATGAGTTTCCATAAGG
  CCAGGGAAACGCAGGTAGGAACCCATGCGGTCGAGCCAGCACTCACCTGACACTAGGAAC
  CGCTGGCCAAAGGTTTTGTTGCCGAAACTCTCAAAGTTGAAATGGTCCATGTATTGCTCA
  AAATAATTCTCATGAAAGTCAGGGTCTAGAACTCTGTCGGCTGAGGGCAGGTGCAGAGAC
  TCAGGAGCTGGTTGGGATCATCAGGGATCTAGGCGGGTCAGGAGGAAGGGCAGCCAGTCT
  GTACTCACCTCTGGCCTGGAGGTTGCACAGTCCCAGTGACAGCAGCAGGACCAGGATCCA
  GGAGGGGACACCATGGTCCACAGGGTAACAAGGATGGAAGTTCATGCTTGATTCTGAGCC
  GGGCGCTGACTGTCATGTGATTTGGTCACATGACCGACACAACGGGCGGGGCAGCATCAC
  GTGATAGTCTGGCGGGGGCTGTCCTACTGTGGCTGGATTCTAGTTGGAGGATCAGCCTAC
  TCTTCTTCAGTTTCCCGGTTCCTCCAAATTTCTGGGCTCCTACTTGTTTCCACAGAGATG
  GATACTGTGGAGGTCCAGGAAGCAGAGAGATGGCTAAGGCTCATCAGGACCGTATGATCT
  CCCAAGTGTCCAGCTACTGAGTACCACAAGGTGATGGGTGGGAGGGTCCTCCCACGGAAG
  GATACCGCAGTCCCTAGGGGTTGCAAGCCCCACATGTTCCACTGGCTGCTAGAGCTACCT
  ACTCAATCAGCCCTGGGCATCACCATCAGGTACTCGGCCAAAATGACCTCTCTGCTTCCA
  GTCCTCAGTTCTGGTCAGCACCAGACAGGCCCATAATTA
  --------------------

Primer pair 2, product size : 941
  Primer left                            Primer right
  start: 25211383                        start: 25212303
  end  : 25211403                        end  : 25212323
  tm   : 57                              tm   : 57
  seq  : ATGAAGCCAGAGTTGTTAGCG           seq  : TGTAATTATGGGCCTGTCTGG

  >77_primer_pair_2_product
  ATGAAGCCAGAGTTGTTAGCGAAGGACCAGATATCCCCCTCATTCCCTGTGTAGAAAAAG
  ATGGGCCCTTCGCCCATCTTCCAGAACTTATCTGTTGGAAGTAAATGAGTTTCCATAAGG
  CCAGGGAAACGCAGGTAGGAACCCATGCGGTCGAGCCAGCACTCACCTGACACTAGGAAC
  CGCTGGCCAAAGGTTTTGTTGCCGAAACTCTCAAAGTTGAAATGGTCCATGTATTGCTCA
  AAATAATTCTCATGAAAGTCAGGGTCTAGAACTCTGTCGGCTGAGGGCAGGTGCAGAGAC
  TCAGGAGCTGGTTGGGATCATCAGGGATCTAGGCGGGTCAGGAGGAAGGGCAGCCAGTCT
  GTACTCACCTCTGGCCTGGAGGTTGCACAGTCCCAGTGACAGCAGCAGGACCAGGATCCA
  GGAGGGGACACCATGGTCCACAGGGTAACAAGGATGGAAGTTCATGCTTGATTCTGAGCC
  GGGCGCTGACTGTCATGTGATTTGGTCACATGACCGACACAACGGGCGGGGCAGCATCAC
  GTGATAGTCTGGCGGGGGCTGTCCTACTGTGGCTGGATTCTAGTTGGAGGATCAGCCTAC
  TCTTCTTCAGTTTCCCGGTTCCTCCAAATTTCTGGGCTCCTACTTGTTTCCACAGAGATG
  GATACTGTGGAGGTCCAGGAAGCAGAGAGATGGCTAAGGCTCATCAGGACCGTATGATCT
  CCCAAGTGTCCAGCTACTGAGTACCACAAGGTGATGGGTGGGAGGGTCCTCCCACGGAAG
  GATACCGCAGTCCCTAGGGGTTGCAAGCCCCACATGTTCCACTGGCTGCTAGAGCTACCT
  ACTCAATCAGCCCTGGGCATCACCATCAGGTACTCGGCCAAAATGACCTCTCTGCTTCCA
  GTCCTCAGTTCTGGTCAGCACCAGACAGGCCCATAATTACA
  ============================================================

Primers for ID: 78
Primer pair 1, product size : 939
  Primer left                            Primer right
  start: 25211383                        start: 25212301
  end  : 25211403                        end  : 25212321
  tm   : 57                              tm   : 57
  seq  : ATGAAGCCAGAGTTGTTAGCG           seq  : TAATTATGGGCCTGTCTGGTG

  >78_primer_pair_1_product
  ATGAAGCCAGAGTTGTTAGCGAAGGACCAGATATCCCCCTCATTCCCTGTGTAGAAAAAG
  ATGGGCCCTTCGCCCATCTTCCAGAACTTATCTGTTGGAAGTAAATGAGTTTCCATAAGG
  CCAGGGAAACGCAGGTAGGAACCCATGCGGTCGAGCCAGCACTCACCTGACACTAGGAAC
  CGCTGGCCAAAGGTTTTGTTGCCGAAACTCTCAAAGTTGAAATGGTCCATGTATTGCTCA
  AAATAATTCTCATGAAAGTCAGGGTCTAGAACTCTGTCGGCTGAGGGCAGGTGCAGAGAC
  TCAGGAGCTGGTTGGGATCATCAGGGATCTAGGCGGGTCAGGAGGAAGGGCAGCCAGTCT
  GTACTCACCTCTGGCCTGGAGGTTGCACAGTCCCAGTGACAGCAGCAGGACCAGGATCCA
  GGAGGGGACACCATGGTCCACAGGGTAACAAGGATGGAAGTTCATGCTTGATTCTGAGCC
  GGGCGCTGACTGTCATGTGATTTGGTCACATGACCGACACAACGGGCGGGGCAGCATCAC
  GTGATAGTCTGGCGGGGGCTGTCCTACTGTGGCTGGATTCTAGTTGGAGGATCAGCCTAC
  TCTTCTTCAGTTTCCCGGTTCCTCCAAATTTCTGGGCTCCTACTTGTTTCCACAGAGATG
  GATACTGTGGAGGTCCAGGAAGCAGAGAGATGGCTAAGGCTCATCAGGACCGTATGATCT
  CCCAAGTGTCCAGCTACTGAGTACCACAAGGTGATGGGTGGGAGGGTCCTCCCACGGAAG
  GATACCGCAGTCCCTAGGGGTTGCAAGCCCCACATGTTCCACTGGCTGCTAGAGCTACCT
  ACTCAATCAGCCCTGGGCATCACCATCAGGTACTCGGCCAAAATGACCTCTCTGCTTCCA
  GTCCTCAGTTCTGGTCAGCACCAGACAGGCCCATAATTA
  --------------------

Primer pair 2, product size : 941
  Primer left                            Primer right
  start: 25211383                        start: 25212303
  end  : 25211403                        end  : 25212323
  tm   : 57                              tm   : 57
  seq  : ATGAAGCCAGAGTTGTTAGCG           seq  : TGTAATTATGGGCCTGTCTGG

  >78_primer_pair_2_product
  ATGAAGCCAGAGTTGTTAGCGAAGGACCAGATATCCCCCTCATTCCCTGTGTAGAAAAAG
  ATGGGCCCTTCGCCCATCTTCCAGAACTTATCTGTTGGAAGTAAATGAGTTTCCATAAGG
  CCAGGGAAACGCAGGTAGGAACCCATGCGGTCGAGCCAGCACTCACCTGACACTAGGAAC
  CGCTGGCCAAAGGTTTTGTTGCCGAAACTCTCAAAGTTGAAATGGTCCATGTATTGCTCA
  AAATAATTCTCATGAAAGTCAGGGTCTAGAACTCTGTCGGCTGAGGGCAGGTGCAGAGAC
  TCAGGAGCTGGTTGGGATCATCAGGGATCTAGGCGGGTCAGGAGGAAGGGCAGCCAGTCT
  GTACTCACCTCTGGCCTGGAGGTTGCACAGTCCCAGTGACAGCAGCAGGACCAGGATCCA
  GGAGGGGACACCATGGTCCACAGGGTAACAAGGATGGAAGTTCATGCTTGATTCTGAGCC
  GGGCGCTGACTGTCATGTGATTTGGTCACATGACCGACACAACGGGCGGGGCAGCATCAC
  GTGATAGTCTGGCGGGGGCTGTCCTACTGTGGCTGGATTCTAGTTGGAGGATCAGCCTAC
  TCTTCTTCAGTTTCCCGGTTCCTCCAAATTTCTGGGCTCCTACTTGTTTCCACAGAGATG
  GATACTGTGGAGGTCCAGGAAGCAGAGAGATGGCTAAGGCTCATCAGGACCGTATGATCT
  CCCAAGTGTCCAGCTACTGAGTACCACAAGGTGATGGGTGGGAGGGTCCTCCCACGGAAG
  GATACCGCAGTCCCTAGGGGTTGCAAGCCCCACATGTTCCACTGGCTGCTAGAGCTACCT
  ACTCAATCAGCCCTGGGCATCACCATCAGGTACTCGGCCAAAATGACCTCTCTGCTTCCA
  GTCCTCAGTTCTGGTCAGCACCAGACAGGCCCATAATTACA
  ============================================================

Primers for ID: 79
Primer pair 1, product size : 838
  Primer left                            Primer right
  start: 25211600                        start: 25212417
  end  : 25211621                        end  : 25212437
  tm   : 57                              tm   : 57
  seq  : TGAAATGGTCCATGTATTGCTC          seq  : AGATTCCCAGAAAGTCCAAGG

  >79_primer_pair_1_product
  TGAAATGGTCCATGTATTGCTCAAAATAATTCTCATGAAAGTCAGGGTCTAGAACTCTGT
  CGGCTGAGGGCAGGTGCAGAGACTCAGGAGCTGGTTGGGATCATCAGGGATCTAGGCGGG
  TCAGGAGGAAGGGCAGCCAGTCTGTACTCACCTCTGGCCTGGAGGTTGCACAGTCCCAGT
  GACAGCAGCAGGACCAGGATCCAGGAGGGGACACCATGGTCCACAGGGTAACAAGGATGG
  AAGTTCATGCTTGATTCTGAGCCGGGCGCTGACTGTCATGTGATTTGGTCACATGACCGA
  CACAACGGGCGGGGCAGCATCACGTGATAGTCTGGCGGGGGCTGTCCTACTGTGGCTGGA
  TTCTAGTTGGAGGATCAGCCTACTCTTCTTCAGTTTCCCGGTTCCTCCAAATTTCTGGGC
  TCCTACTTGTTTCCACAGAGATGGATACTGTGGAGGTCCAGGAAGCAGAGAGATGGCTAA
  GGCTCATCAGGACCGTATGATCTCCCAAGTGTCCAGCTACTGAGTACCACAAGGTGATGG
  GTGGGAGGGTCCTCCCACGGAAGGATACCGCAGTCCCTAGGGGTTGCAAGCCCCACATGT
  TCCACTGGCTGCTAGAGCTACCTACTCAATCAGCCCTGGGCATCACCATCAGGTACTCGG
  CCAAAATGACCTCTCTGCTTCCAGTCCTCAGTTCTGGTCAGCACCAGACAGGCCCATAAT
  TACAGAGCCAGGGAAACTGGAACATTTGTCTCCCCTTAGACAGTGGCAGCAGGAAGGTGG
  GGGGTTGTTGCAGAGGAACAGTGTCTCTGAGAGAGGACCTTGGACTTTCTGGGAATCT
  --------------------

Primer pair 2, product size : 836
  Primer left                            Primer right
  start: 25211600                        start: 25212415
  end  : 25211621                        end  : 25212435
  tm   : 57                              tm   : 57
  seq  : TGAAATGGTCCATGTATTGCTC          seq  : ATTCCCAGAAAGTCCAAGGTC

  >79_primer_pair_2_product
  TGAAATGGTCCATGTATTGCTCAAAATAATTCTCATGAAAGTCAGGGTCTAGAACTCTGT
  CGGCTGAGGGCAGGTGCAGAGACTCAGGAGCTGGTTGGGATCATCAGGGATCTAGGCGGG
  TCAGGAGGAAGGGCAGCCAGTCTGTACTCACCTCTGGCCTGGAGGTTGCACAGTCCCAGT
  GACAGCAGCAGGACCAGGATCCAGGAGGGGACACCATGGTCCACAGGGTAACAAGGATGG
  AAGTTCATGCTTGATTCTGAGCCGGGCGCTGACTGTCATGTGATTTGGTCACATGACCGA
  CACAACGGGCGGGGCAGCATCACGTGATAGTCTGGCGGGGGCTGTCCTACTGTGGCTGGA
  TTCTAGTTGGAGGATCAGCCTACTCTTCTTCAGTTTCCCGGTTCCTCCAAATTTCTGGGC
  TCCTACTTGTTTCCACAGAGATGGATACTGTGGAGGTCCAGGAAGCAGAGAGATGGCTAA
  GGCTCATCAGGACCGTATGATCTCCCAAGTGTCCAGCTACTGAGTACCACAAGGTGATGG
  GTGGGAGGGTCCTCCCACGGAAGGATACCGCAGTCCCTAGGGGTTGCAAGCCCCACATGT
  TCCACTGGCTGCTAGAGCTACCTACTCAATCAGCCCTGGGCATCACCATCAGGTACTCGG
  CCAAAATGACCTCTCTGCTTCCAGTCCTCAGTTCTGGTCAGCACCAGACAGGCCCATAAT
  TACAGAGCCAGGGAAACTGGAACATTTGTCTCCCCTTAGACAGTGGCAGCAGGAAGGTGG
  GGGGTTGTTGCAGAGGAACAGTGTCTCTGAGAGAGGACCTTGGACTTTCTGGGAAT
  ============================================================

Primers for ID: 80
Primer pair 1, product size : 831
  Primer left                            Primer right
  start: 25211842                        start: 25212653
  end  : 25211862                        end  : 25212672
  tm   : 57                              tm   : 55
  seq  : GTTCATGCTTGATTCTGAGCC           seq  : ATGGACATGTTCCACTCACG

  >80_primer_pair_1_product
  GTTCATGCTTGATTCTGAGCCGGGCGCTGACTGTCATGTGATTTGGTCACATGACCGACA
  CAACGGGCGGGGCAGCATCACGTGATAGTCTGGCGGGGGCTGTCCTACTGTGGCTGGATT
  CTAGTTGGAGGATCAGCCTACTCTTCTTCAGTTTCCCGGTTCCTCCAAATTTCTGGGCTC
  CTACTTGTTTCCACAGAGATGGATACTGTGGAGGTCCAGGAAGCAGAGAGATGGCTAAGG
  CTCATCAGGACCGTATGATCTCCCAAGTGTCCAGCTACTGAGTACCACAAGGTGATGGGT
  GGGAGGGTCCTCCCACGGAAGGATACCGCAGTCCCTAGGGGTTGCAAGCCCCACATGTTC
  CACTGGCTGCTAGAGCTACCTACTCAATCAGCCCTGGGCATCACCATCAGGTACTCGGCC
  AAAATGACCTCTCTGCTTCCAGTCCTCAGTTCTGGTCAGCACCAGACAGGCCCATAATTA
  CAGAGCCAGGGAAACTGGAACATTTGTCTCCCCTTAGACAGTGGCAGCAGGAAGGTGGGG
  GGTTGTTGCAGAGGAACAGTGTCTCTGAGAGAGGACCTTGGACTTTCTGGGAATCTCTGA
  GCTGCCCGGTTCTCCCCACTGCTGGCACTGTGCCCACAGCCCAAACAGAATGGGGGAGAT
  GGAGGGGCAGGGCTTCTGTGGGAAGCTGCCCTCCACCTCATTGGCACAGAGTGTCTCATT
  GCAGAGAGAAAAAAGGACCAGTTTTCTCTCTGGCACCCAGGTCTGGAAGAGGAGTGACAT
  CCACGGAAGTTGGTGACTTGGACTGGCTGGCCGTGAGTGGAACATGTCCAT
  --------------------

Primer pair 2, product size : 806
  Primer left                            Primer right
  start: 25211867                        start: 25212653
  end  : 25211887                        end  : 25212672
  tm   : 57                              tm   : 55
  seq  : GCTGACTGTCATGTGATTTGG           seq  : ATGGACATGTTCCACTCACG

  >80_primer_pair_2_product
  GCTGACTGTCATGTGATTTGGTCACATGACCGACACAACGGGCGGGGCAGCATCACGTGA
  TAGTCTGGCGGGGGCTGTCCTACTGTGGCTGGATTCTAGTTGGAGGATCAGCCTACTCTT
  CTTCAGTTTCCCGGTTCCTCCAAATTTCTGGGCTCCTACTTGTTTCCACAGAGATGGATA
  CTGTGGAGGTCCAGGAAGCAGAGAGATGGCTAAGGCTCATCAGGACCGTATGATCTCCCA
  AGTGTCCAGCTACTGAGTACCACAAGGTGATGGGTGGGAGGGTCCTCCCACGGAAGGATA
  CCGCAGTCCCTAGGGGTTGCAAGCCCCACATGTTCCACTGGCTGCTAGAGCTACCTACTC
  AATCAGCCCTGGGCATCACCATCAGGTACTCGGCCAAAATGACCTCTCTGCTTCCAGTCC
  TCAGTTCTGGTCAGCACCAGACAGGCCCATAATTACAGAGCCAGGGAAACTGGAACATTT
  GTCTCCCCTTAGACAGTGGCAGCAGGAAGGTGGGGGGTTGTTGCAGAGGAACAGTGTCTC
  TGAGAGAGGACCTTGGACTTTCTGGGAATCTCTGAGCTGCCCGGTTCTCCCCACTGCTGG
  CACTGTGCCCACAGCCCAAACAGAATGGGGGAGATGGAGGGGCAGGGCTTCTGTGGGAAG
  CTGCCCTCCACCTCATTGGCACAGAGTGTCTCATTGCAGAGAGAAAAAAGGACCAGTTTT
  CTCTCTGGCACCCAGGTCTGGAAGAGGAGTGACATCCACGGAAGTTGGTGACTTGGACTG
  GCTGGCCGTGAGTGGAACATGTCCAT
  ============================================================

Primers for ID: 81
Primer pair 1, product size : 782
  Primer left                            Primer right
  start: 25209909                        start: 25210670
  end  : 25209929                        end  : 25210690
  tm   : 57                              tm   : 57
  seq  : GATGGCATGAAAGCAGATAGC           seq  : AGTTTCAGGTCCTTCTTTCCC

  >81_primer_pair_1_product
  GATGGCATGAAAGCAGATAGCTACAAGGCTCTTGGACCAGGCTAGCACTGGGTCCTGCAC
  CCAGGGAGAGCCACCTCACCTTGACAGGGTTGGCAGGAAGGGGCCCTAGAAAGTCAGTAG
  GATACGGGTAGTCCATCATGGCGAGCACAGTAAATGCATTTCGGGCAAACCCAAAGAGCT
  GAGTCAGGTCCTTTGGGCTGGAAAGTGATTGACAGGTACCAAAGTTCTGGCTGATGGTGT
  CATAGGCTGGGAAGAGAGAGGCCAGGAGAAAAGGCTGAGGAAACTGCTGGCAAATGTGAA
  GGGCAAGAATGAATGCCCAAGGTGGGCAGCAGGTGAGGAAAGAGTCCCTCACCTCCCTGG
  AGGAACAAGTCTTTGATTTGCTGAAAGGCATCCCGCACAGCCTGGGCGCACTTGGGACTC
  TGGCCATAAAAGTCCTGGAGAAGAGACCAAGGTTGCTGCTGCCATTCTTGCACTGGCCTG
  GGGTACCCAAGTCCCCTCACTCACCGCTGTGACATCTCGGAAGAATTGGTAGGAGTCCCC
  AAGGCCTGCAACAGCTACAACAGGAGCGCTGGCTGCCAGTGCCCCAGCCACCAGGTGGGG
  GTACTTCATCCTCATGTAGGCACTCAGCATCCCCCCATAACTGGGAGTACAGAGCACAGA
  TCATGGTTGTGGGAAGCTGCCCACAACTCAGGCGAGCAGCCTCACTGTCCTCCAGGCTGA
  GGTGCTAGGCTGCTCTTTCCCTGCTCAGAACGCCCAAGGGTGGGAAAGAAGGACCTGAAA
  CT
  --------------------

Primer pair 2, product size : 779
  Primer left                            Primer right
  start: 25209909                        start: 25210668
  end  : 25209929                        end  : 25210687
  tm   : 57                              tm   : 55
  seq  : GATGGCATGAAAGCAGATAGC           seq  : TTCAGGTCCTTCTTTCCCAC

  >81_primer_pair_2_product
  GATGGCATGAAAGCAGATAGCTACAAGGCTCTTGGACCAGGCTAGCACTGGGTCCTGCAC
  CCAGGGAGAGCCACCTCACCTTGACAGGGTTGGCAGGAAGGGGCCCTAGAAAGTCAGTAG
  GATACGGGTAGTCCATCATGGCGAGCACAGTAAATGCATTTCGGGCAAACCCAAAGAGCT
  GAGTCAGGTCCTTTGGGCTGGAAAGTGATTGACAGGTACCAAAGTTCTGGCTGATGGTGT
  CATAGGCTGGGAAGAGAGAGGCCAGGAGAAAAGGCTGAGGAAACTGCTGGCAAATGTGAA
  GGGCAAGAATGAATGCCCAAGGTGGGCAGCAGGTGAGGAAAGAGTCCCTCACCTCCCTGG
  AGGAACAAGTCTTTGATTTGCTGAAAGGCATCCCGCACAGCCTGGGCGCACTTGGGACTC
  TGGCCATAAAAGTCCTGGAGAAGAGACCAAGGTTGCTGCTGCCATTCTTGCACTGGCCTG
  GGGTACCCAAGTCCCCTCACTCACCGCTGTGACATCTCGGAAGAATTGGTAGGAGTCCCC
  AAGGCCTGCAACAGCTACAACAGGAGCGCTGGCTGCCAGTGCCCCAGCCACCAGGTGGGG
  GTACTTCATCCTCATGTAGGCACTCAGCATCCCCCCATAACTGGGAGTACAGAGCACAGA
  TCATGGTTGTGGGAAGCTGCCCACAACTCAGGCGAGCAGCCTCACTGTCCTCCAGGCTGA
  GGTGCTAGGCTGCTCTTTCCCTGCTCAGAACGCCCAAGGGTGGGAAAGAAGGACCTGAA
  ============================================================

Primers for ID: 82
Primer pair 1, product size : 881
  Primer left                            Primer right
  start: 25209909                        start: 25210770
  end  : 25209929                        end  : 25210789
  tm   : 57                              tm   : 55
  seq  : GATGGCATGAAAGCAGATAGC           seq  : TGTCTTCATTGCTGGGTCAG

  >82_primer_pair_1_product
  GATGGCATGAAAGCAGATAGCTACAAGGCTCTTGGACCAGGCTAGCACTGGGTCCTGCAC
  CCAGGGAGAGCCACCTCACCTTGACAGGGTTGGCAGGAAGGGGCCCTAGAAAGTCAGTAG
  GATACGGGTAGTCCATCATGGCGAGCACAGTAAATGCATTTCGGGCAAACCCAAAGAGCT
  GAGTCAGGTCCTTTGGGCTGGAAAGTGATTGACAGGTACCAAAGTTCTGGCTGATGGTGT
  CATAGGCTGGGAAGAGAGAGGCCAGGAGAAAAGGCTGAGGAAACTGCTGGCAAATGTGAA
  GGGCAAGAATGAATGCCCAAGGTGGGCAGCAGGTGAGGAAAGAGTCCCTCACCTCCCTGG
  AGGAACAAGTCTTTGATTTGCTGAAAGGCATCCCGCACAGCCTGGGCGCACTTGGGACTC
  TGGCCATAAAAGTCCTGGAGAAGAGACCAAGGTTGCTGCTGCCATTCTTGCACTGGCCTG
  GGGTACCCAAGTCCCCTCACTCACCGCTGTGACATCTCGGAAGAATTGGTAGGAGTCCCC
  AAGGCCTGCAACAGCTACAACAGGAGCGCTGGCTGCCAGTGCCCCAGCCACCAGGTGGGG
  GTACTTCATCCTCATGTAGGCACTCAGCATCCCCCCATAACTGGGAGTACAGAGCACAGA
  TCATGGTTGTGGGAAGCTGCCCACAACTCAGGCGAGCAGCCTCACTGTCCTCCAGGCTGA
  GGTGCTAGGCTGCTCTTTCCCTGCTCAGAACGCCCAAGGGTGGGAAAGAAGGACCTGAAA
  CTGTCAGGCCCACACACCCTGATCCCAGGGCCAAGGCAGATACAGCCTTCACTGGGAGAA
  GGCACCTGTGGGTGCCCTGCCCTGACCCAGCAATGAAGACA
  --------------------

Primer pair 2, product size : 782
  Primer left                            Primer right
  start: 25209909                        start: 25210670
  end  : 25209929                        end  : 25210690
  tm   : 57                              tm   : 57
  seq  : GATGGCATGAAAGCAGATAGC           seq  : AGTTTCAGGTCCTTCTTTCCC

  >82_primer_pair_2_product
  GATGGCATGAAAGCAGATAGCTACAAGGCTCTTGGACCAGGCTAGCACTGGGTCCTGCAC
  CCAGGGAGAGCCACCTCACCTTGACAGGGTTGGCAGGAAGGGGCCCTAGAAAGTCAGTAG
  GATACGGGTAGTCCATCATGGCGAGCACAGTAAATGCATTTCGGGCAAACCCAAAGAGCT
  GAGTCAGGTCCTTTGGGCTGGAAAGTGATTGACAGGTACCAAAGTTCTGGCTGATGGTGT
  CATAGGCTGGGAAGAGAGAGGCCAGGAGAAAAGGCTGAGGAAACTGCTGGCAAATGTGAA
  GGGCAAGAATGAATGCCCAAGGTGGGCAGCAGGTGAGGAAAGAGTCCCTCACCTCCCTGG
  AGGAACAAGTCTTTGATTTGCTGAAAGGCATCCCGCACAGCCTGGGCGCACTTGGGACTC
  TGGCCATAAAAGTCCTGGAGAAGAGACCAAGGTTGCTGCTGCCATTCTTGCACTGGCCTG
  GGGTACCCAAGTCCCCTCACTCACCGCTGTGACATCTCGGAAGAATTGGTAGGAGTCCCC
  AAGGCCTGCAACAGCTACAACAGGAGCGCTGGCTGCCAGTGCCCCAGCCACCAGGTGGGG
  GTACTTCATCCTCATGTAGGCACTCAGCATCCCCCCATAACTGGGAGTACAGAGCACAGA
  TCATGGTTGTGGGAAGCTGCCCACAACTCAGGCGAGCAGCCTCACTGTCCTCCAGGCTGA
  GGTGCTAGGCTGCTCTTTCCCTGCTCAGAACGCCCAAGGGTGGGAAAGAAGGACCTGAAA
  CT
  ============================================================

Primers for ID: 83
Primer pair 1, product size : 843
  Primer left                            Primer right
  start: 25210129                        start: 25210951
  end  : 25210149                        end  : 25210971
  tm   : 57                              tm   : 57
  seq  : AAAGTTCTGGCTGATGGTGTC           seq  : TATACAAGGAGGGCCAAAGAG

  >83_primer_pair_1_product
  AAAGTTCTGGCTGATGGTGTCATAGGCTGGGAAGAGAGAGGCCAGGAGAAAAGGCTGAGG
  AAACTGCTGGCAAATGTGAAGGGCAAGAATGAATGCCCAAGGTGGGCAGCAGGTGAGGAA
  AGAGTCCCTCACCTCCCTGGAGGAACAAGTCTTTGATTTGCTGAAAGGCATCCCGCACAG
  CCTGGGCGCACTTGGGACTCTGGCCATAAAAGTCCTGGAGAAGAGACCAAGGTTGCTGCT
  GCCATTCTTGCACTGGCCTGGGGTACCCAAGTCCCCTCACTCACCGCTGTGACATCTCGG
  AAGAATTGGTAGGAGTCCCCAAGGCCTGCAACAGCTACAACAGGAGCGCTGGCTGCCAGT
  GCCCCAGCCACCAGGTGGGGGTACTTCATCCTCATGTAGGCACTCAGCATCCCCCCATAA
  CTGGGAGTACAGAGCACAGATCATGGTTGTGGGAAGCTGCCCACAACTCAGGCGAGCAGC
  CTCACTGTCCTCCAGGCTGAGGTGCTAGGCTGCTCTTTCCCTGCTCAGAACGCCCAAGGG
  TGGGAAAGAAGGACCTGAAACTGTCAGGCCCACACACCCTGATCCCAGGGCCAAGGCAGA
  TACAGCCTTCACTGGGAGAAGGCACCTGTGGGTGCCCTGCCCTGACCCAGCAATGAAGAC
  ATTGCAGAGACAAAGTCAGAAGGAATTGTCCCACTAGTGGGAACAACATAGCATACACTG
  CCTATGAGGTCCACTCAAGGAGGGCTTCCAGAAGGAGGTAAAGCTAGACCCCGCCCTTCC
  ACATGTGGGGTAGGCATAGGATGTTGAGACTGTAAGAGACATCTCTTTGGCCCTCCTTGT
  ATA
  --------------------

Primer pair 2, product size : 844
  Primer left                            Primer right
  start: 25210129                        start: 25210951
  end  : 25210149                        end  : 25210972
  tm   : 57                              tm   : 61
  seq  : AAAGTTCTGGCTGATGGTGTC           seq  : CTATACAAGGAGGGCCAAAGAG

  >83_primer_pair_2_product
  AAAGTTCTGGCTGATGGTGTCATAGGCTGGGAAGAGAGAGGCCAGGAGAAAAGGCTGAGG
  AAACTGCTGGCAAATGTGAAGGGCAAGAATGAATGCCCAAGGTGGGCAGCAGGTGAGGAA
  AGAGTCCCTCACCTCCCTGGAGGAACAAGTCTTTGATTTGCTGAAAGGCATCCCGCACAG
  CCTGGGCGCACTTGGGACTCTGGCCATAAAAGTCCTGGAGAAGAGACCAAGGTTGCTGCT
  GCCATTCTTGCACTGGCCTGGGGTACCCAAGTCCCCTCACTCACCGCTGTGACATCTCGG
  AAGAATTGGTAGGAGTCCCCAAGGCCTGCAACAGCTACAACAGGAGCGCTGGCTGCCAGT
  GCCCCAGCCACCAGGTGGGGGTACTTCATCCTCATGTAGGCACTCAGCATCCCCCCATAA
  CTGGGAGTACAGAGCACAGATCATGGTTGTGGGAAGCTGCCCACAACTCAGGCGAGCAGC
  CTCACTGTCCTCCAGGCTGAGGTGCTAGGCTGCTCTTTCCCTGCTCAGAACGCCCAAGGG
  TGGGAAAGAAGGACCTGAAACTGTCAGGCCCACACACCCTGATCCCAGGGCCAAGGCAGA
  TACAGCCTTCACTGGGAGAAGGCACCTGTGGGTGCCCTGCCCTGACCCAGCAATGAAGAC
  ATTGCAGAGACAAAGTCAGAAGGAATTGTCCCACTAGTGGGAACAACATAGCATACACTG
  CCTATGAGGTCCACTCAAGGAGGGCTTCCAGAAGGAGGTAAAGCTAGACCCCGCCCTTCC
  ACATGTGGGGTAGGCATAGGATGTTGAGACTGTAAGAGACATCTCTTTGGCCCTCCTTGT
  ATAG
  ============================================================

Primers for ID: 84
Primer pair 1, product size : 843
  Primer left                            Primer right
  start: 25210129                        start: 25210951
  end  : 25210149                        end  : 25210971
  tm   : 57                              tm   : 57
  seq  : AAAGTTCTGGCTGATGGTGTC           seq  : TATACAAGGAGGGCCAAAGAG

  >84_primer_pair_1_product
  AAAGTTCTGGCTGATGGTGTCATAGGCTGGGAAGAGAGAGGCCAGGAGAAAAGGCTGAGG
  AAACTGCTGGCAAATGTGAAGGGCAAGAATGAATGCCCAAGGTGGGCAGCAGGTGAGGAA
  AGAGTCCCTCACCTCCCTGGAGGAACAAGTCTTTGATTTGCTGAAAGGCATCCCGCACAG
  CCTGGGCGCACTTGGGACTCTGGCCATAAAAGTCCTGGAGAAGAGACCAAGGTTGCTGCT
  GCCATTCTTGCACTGGCCTGGGGTACCCAAGTCCCCTCACTCACCGCTGTGACATCTCGG
  AAGAATTGGTAGGAGTCCCCAAGGCCTGCAACAGCTACAACAGGAGCGCTGGCTGCCAGT
  GCCCCAGCCACCAGGTGGGGGTACTTCATCCTCATGTAGGCACTCAGCATCCCCCCATAA
  CTGGGAGTACAGAGCACAGATCATGGTTGTGGGAAGCTGCCCACAACTCAGGCGAGCAGC
  CTCACTGTCCTCCAGGCTGAGGTGCTAGGCTGCTCTTTCCCTGCTCAGAACGCCCAAGGG
  TGGGAAAGAAGGACCTGAAACTGTCAGGCCCACACACCCTGATCCCAGGGCCAAGGCAGA
  TACAGCCTTCACTGGGAGAAGGCACCTGTGGGTGCCCTGCCCTGACCCAGCAATGAAGAC
  ATTGCAGAGACAAAGTCAGAAGGAATTGTCCCACTAGTGGGAACAACATAGCATACACTG
  CCTATGAGGTCCACTCAAGGAGGGCTTCCAGAAGGAGGTAAAGCTAGACCCCGCCCTTCC
  ACATGTGGGGTAGGCATAGGATGTTGAGACTGTAAGAGACATCTCTTTGGCCCTCCTTGT
  ATA
  --------------------

Primer pair 2, product size : 880
  Primer left                            Primer right
  start: 25210172                        start: 25211030
  end  : 25210192                        end  : 25211051
  tm   : 57                              tm   : 61
  seq  : AGGAGAAAAGGCTGAGGAAAC           seq  : AGATCTGGACCCATGAGAGAAC

  >84_primer_pair_2_product
  AGGAGAAAAGGCTGAGGAAACTGCTGGCAAATGTGAAGGGCAAGAATGAATGCCCAAGGT
  GGGCAGCAGGTGAGGAAAGAGTCCCTCACCTCCCTGGAGGAACAAGTCTTTGATTTGCTG
  AAAGGCATCCCGCACAGCCTGGGCGCACTTGGGACTCTGGCCATAAAAGTCCTGGAGAAG
  AGACCAAGGTTGCTGCTGCCATTCTTGCACTGGCCTGGGGTACCCAAGTCCCCTCACTCA
  CCGCTGTGACATCTCGGAAGAATTGGTAGGAGTCCCCAAGGCCTGCAACAGCTACAACAG
  GAGCGCTGGCTGCCAGTGCCCCAGCCACCAGGTGGGGGTACTTCATCCTCATGTAGGCAC
  TCAGCATCCCCCCATAACTGGGAGTACAGAGCACAGATCATGGTTGTGGGAAGCTGCCCA
  CAACTCAGGCGAGCAGCCTCACTGTCCTCCAGGCTGAGGTGCTAGGCTGCTCTTTCCCTG
  CTCAGAACGCCCAAGGGTGGGAAAGAAGGACCTGAAACTGTCAGGCCCACACACCCTGAT
  CCCAGGGCCAAGGCAGATACAGCCTTCACTGGGAGAAGGCACCTGTGGGTGCCCTGCCCT
  GACCCAGCAATGAAGACATTGCAGAGACAAAGTCAGAAGGAATTGTCCCACTAGTGGGAA
  CAACATAGCATACACTGCCTATGAGGTCCACTCAAGGAGGGCTTCCAGAAGGAGGTAAAG
  CTAGACCCCGCCCTTCCACATGTGGGGTAGGCATAGGATGTTGAGACTGTAAGAGACATC
  TCTTTGGCCCTCCTTGTATAGGGTGTCAATCGGCACAACAGGGTGGAGCCTTAGAGTAGG
  GTAAGATTAGGACTCTAGGTTCTCTCATGGGTCCAGATCT
  ============================================================

Primers for ID: 95
Primer pair 1, product size : 777
  Primer left                            Primer right
  start: 25211226                        start: 25211982
  end  : 25211245                        end  : 25212002
  tm   : 55                              tm   : 57
  seq  : ATTTCCCATAGTACCGCTGC            seq  : AACCGGGAAACTGAAGAAGAG

  >95_primer_pair_1_product
  ATTTCCCATAGTACCGCTGCAGAAAGCAGGAAGGGATGGCTAATCCACTCCTCGGTGCTC
  CCCACCTCCTTCAACTCAGGGACTGCCAGGAACTGTACAGGTACCCACGTGCTCAGCAAA
  GACAAGCAGGGCCTCCTGCTGGGCTGCCAGTTCCACCATGAAGCCAGAGTTGTTAGCGAA
  GGACCAGATATCCCCCTCATTCCCTGTGTAGAAAAAGATGGGCCCTTCGCCCATCTTCCA
  GAACTTATCTGTTGGAAGTAAATGAGTTTCCATAAGGCCAGGGAAACGCAGGTAGGAACC
  CATGCGGTCGAGCCAGCACTCACCTGACACTAGGAACCGCTGGCCAAAGGTTTTGTTGCC
  GAAACTCTCAAAGTTGAAATGGTCCATGTATTGCTCAAAATAATTCTCATGAAAGTCAGG
  GTCTAGAACTCTGTCGGCTGAGGGCAGGTGCAGAGACTCAGGAGCTGGTTGGGATCATCA
  GGGATCTAGGCGGGTCAGGAGGAAGGGCAGCCAGTCTGTACTCACCTCTGGCCTGGAGGT
  TGCACAGTCCCAGTGACAGCAGCAGGACCAGGATCCAGGAGGGGACACCATGGTCCACAG
  GGTAACAAGGATGGAAGTTCATGCTTGATTCTGAGCCGGGCGCTGACTGTCATGTGATTT
  GGTCACATGACCGACACAACGGGCGGGGCAGCATCACGTGATAGTCTGGCGGGGGCTGTC
  CTACTGTGGCTGGATTCTAGTTGGAGGATCAGCCTACTCTTCTTCAGTTTCCCGGTT
  --------------------

Primer pair 2, product size : 781
  Primer left                            Primer right
  start: 25211222                        start: 25211982
  end  : 25211241                        end  : 25212002
  tm   : 55                              tm   : 57
  seq  : AGCGATTTCCCATAGTACCG            seq  : AACCGGGAAACTGAAGAAGAG

  >95_primer_pair_2_product
  AGCGATTTCCCATAGTACCGCTGCAGAAAGCAGGAAGGGATGGCTAATCCACTCCTCGGT
  GCTCCCCACCTCCTTCAACTCAGGGACTGCCAGGAACTGTACAGGTACCCACGTGCTCAG
  CAAAGACAAGCAGGGCCTCCTGCTGGGCTGCCAGTTCCACCATGAAGCCAGAGTTGTTAG
  CGAAGGACCAGATATCCCCCTCATTCCCTGTGTAGAAAAAGATGGGCCCTTCGCCCATCT
  TCCAGAACTTATCTGTTGGAAGTAAATGAGTTTCCATAAGGCCAGGGAAACGCAGGTAGG
  AACCCATGCGGTCGAGCCAGCACTCACCTGACACTAGGAACCGCTGGCCAAAGGTTTTGT
  TGCCGAAACTCTCAAAGTTGAAATGGTCCATGTATTGCTCAAAATAATTCTCATGAAAGT
  CAGGGTCTAGAACTCTGTCGGCTGAGGGCAGGTGCAGAGACTCAGGAGCTGGTTGGGATC
  ATCAGGGATCTAGGCGGGTCAGGAGGAAGGGCAGCCAGTCTGTACTCACCTCTGGCCTGG
  AGGTTGCACAGTCCCAGTGACAGCAGCAGGACCAGGATCCAGGAGGGGACACCATGGTCC
  ACAGGGTAACAAGGATGGAAGTTCATGCTTGATTCTGAGCCGGGCGCTGACTGTCATGTG
  ATTTGGTCACATGACCGACACAACGGGCGGGGCAGCATCACGTGATAGTCTGGCGGGGGC
  TGTCCTACTGTGGCTGGATTCTAGTTGGAGGATCAGCCTACTCTTCTTCAGTTTCCCGGT
  T
  ============================================================

Primers for ID: 96
Primer pair 1, product size : 939
  Primer left                            Primer right
  start: 25211383                        start: 25212301
  end  : 25211403                        end  : 25212321
  tm   : 57                              tm   : 57
  seq  : ATGAAGCCAGAGTTGTTAGCG           seq  : TAATTATGGGCCTGTCTGGTG

  >96_primer_pair_1_product
  ATGAAGCCAGAGTTGTTAGCGAAGGACCAGATATCCCCCTCATTCCCTGTGTAGAAAAAG
  ATGGGCCCTTCGCCCATCTTCCAGAACTTATCTGTTGGAAGTAAATGAGTTTCCATAAGG
  CCAGGGAAACGCAGGTAGGAACCCATGCGGTCGAGCCAGCACTCACCTGACACTAGGAAC
  CGCTGGCCAAAGGTTTTGTTGCCGAAACTCTCAAAGTTGAAATGGTCCATGTATTGCTCA
  AAATAATTCTCATGAAAGTCAGGGTCTAGAACTCTGTCGGCTGAGGGCAGGTGCAGAGAC
  TCAGGAGCTGGTTGGGATCATCAGGGATCTAGGCGGGTCAGGAGGAAGGGCAGCCAGTCT
  GTACTCACCTCTGGCCTGGAGGTTGCACAGTCCCAGTGACAGCAGCAGGACCAGGATCCA
  GGAGGGGACACCATGGTCCACAGGGTAACAAGGATGGAAGTTCATGCTTGATTCTGAGCC
  GGGCGCTGACTGTCATGTGATTTGGTCACATGACCGACACAACGGGCGGGGCAGCATCAC
  GTGATAGTCTGGCGGGGGCTGTCCTACTGTGGCTGGATTCTAGTTGGAGGATCAGCCTAC
  TCTTCTTCAGTTTCCCGGTTCCTCCAAATTTCTGGGCTCCTACTTGTTTCCACAGAGATG
  GATACTGTGGAGGTCCAGGAAGCAGAGAGATGGCTAAGGCTCATCAGGACCGTATGATCT
  CCCAAGTGTCCAGCTACTGAGTACCACAAGGTGATGGGTGGGAGGGTCCTCCCACGGAAG
  GATACCGCAGTCCCTAGGGGTTGCAAGCCCCACATGTTCCACTGGCTGCTAGAGCTACCT
  ACTCAATCAGCCCTGGGCATCACCATCAGGTACTCGGCCAAAATGACCTCTCTGCTTCCA
  GTCCTCAGTTCTGGTCAGCACCAGACAGGCCCATAATTA
  --------------------

Primer pair 2, product size : 941
  Primer left                            Primer right
  start: 25211383                        start: 25212303
  end  : 25211403                        end  : 25212323
  tm   : 57                              tm   : 57
  seq  : ATGAAGCCAGAGTTGTTAGCG           seq  : TGTAATTATGGGCCTGTCTGG

  >96_primer_pair_2_product
  ATGAAGCCAGAGTTGTTAGCGAAGGACCAGATATCCCCCTCATTCCCTGTGTAGAAAAAG
  ATGGGCCCTTCGCCCATCTTCCAGAACTTATCTGTTGGAAGTAAATGAGTTTCCATAAGG
  CCAGGGAAACGCAGGTAGGAACCCATGCGGTCGAGCCAGCACTCACCTGACACTAGGAAC
  CGCTGGCCAAAGGTTTTGTTGCCGAAACTCTCAAAGTTGAAATGGTCCATGTATTGCTCA
  AAATAATTCTCATGAAAGTCAGGGTCTAGAACTCTGTCGGCTGAGGGCAGGTGCAGAGAC
  TCAGGAGCTGGTTGGGATCATCAGGGATCTAGGCGGGTCAGGAGGAAGGGCAGCCAGTCT
  GTACTCACCTCTGGCCTGGAGGTTGCACAGTCCCAGTGACAGCAGCAGGACCAGGATCCA
  GGAGGGGACACCATGGTCCACAGGGTAACAAGGATGGAAGTTCATGCTTGATTCTGAGCC
  GGGCGCTGACTGTCATGTGATTTGGTCACATGACCGACACAACGGGCGGGGCAGCATCAC
  GTGATAGTCTGGCGGGGGCTGTCCTACTGTGGCTGGATTCTAGTTGGAGGATCAGCCTAC
  TCTTCTTCAGTTTCCCGGTTCCTCCAAATTTCTGGGCTCCTACTTGTTTCCACAGAGATG
  GATACTGTGGAGGTCCAGGAAGCAGAGAGATGGCTAAGGCTCATCAGGACCGTATGATCT
  CCCAAGTGTCCAGCTACTGAGTACCACAAGGTGATGGGTGGGAGGGTCCTCCCACGGAAG
  GATACCGCAGTCCCTAGGGGTTGCAAGCCCCACATGTTCCACTGGCTGCTAGAGCTACCT
  ACTCAATCAGCCCTGGGCATCACCATCAGGTACTCGGCCAAAATGACCTCTCTGCTTCCA
  GTCCTCAGTTCTGGTCAGCACCAGACAGGCCCATAATTACA
  ============================================================

Primers for ID: 97
Primer pair 1, product size : 894
  Primer left                            Primer right
  start: 25211428                        start: 25212301
  end  : 25211448                        end  : 25212321
  tm   : 57                              tm   : 57
  seq  : CCTGTGTAGAAAAAGATGGGC           seq  : TAATTATGGGCCTGTCTGGTG

  >97_primer_pair_1_product
  CCTGTGTAGAAAAAGATGGGCCCTTCGCCCATCTTCCAGAACTTATCTGTTGGAAGTAAA
  TGAGTTTCCATAAGGCCAGGGAAACGCAGGTAGGAACCCATGCGGTCGAGCCAGCACTCA
  CCTGACACTAGGAACCGCTGGCCAAAGGTTTTGTTGCCGAAACTCTCAAAGTTGAAATGG
  TCCATGTATTGCTCAAAATAATTCTCATGAAAGTCAGGGTCTAGAACTCTGTCGGCTGAG
  GGCAGGTGCAGAGACTCAGGAGCTGGTTGGGATCATCAGGGATCTAGGCGGGTCAGGAGG
  AAGGGCAGCCAGTCTGTACTCACCTCTGGCCTGGAGGTTGCACAGTCCCAGTGACAGCAG
  CAGGACCAGGATCCAGGAGGGGACACCATGGTCCACAGGGTAACAAGGATGGAAGTTCAT
  GCTTGATTCTGAGCCGGGCGCTGACTGTCATGTGATTTGGTCACATGACCGACACAACGG
  GCGGGGCAGCATCACGTGATAGTCTGGCGGGGGCTGTCCTACTGTGGCTGGATTCTAGTT
  GGAGGATCAGCCTACTCTTCTTCAGTTTCCCGGTTCCTCCAAATTTCTGGGCTCCTACTT
  GTTTCCACAGAGATGGATACTGTGGAGGTCCAGGAAGCAGAGAGATGGCTAAGGCTCATC
  AGGACCGTATGATCTCCCAAGTGTCCAGCTACTGAGTACCACAAGGTGATGGGTGGGAGG
  GTCCTCCCACGGAAGGATACCGCAGTCCCTAGGGGTTGCAAGCCCCACATGTTCCACTGG
  CTGCTAGAGCTACCTACTCAATCAGCCCTGGGCATCACCATCAGGTACTCGGCCAAAATG
  ACCTCTCTGCTTCCAGTCCTCAGTTCTGGTCAGCACCAGACAGGCCCATAATTA
  --------------------

Primer pair 2, product size : 896
  Primer left                            Primer right
  start: 25211428                        start: 25212303
  end  : 25211448                        end  : 25212323
  tm   : 57                              tm   : 57
  seq  : CCTGTGTAGAAAAAGATGGGC           seq  : TGTAATTATGGGCCTGTCTGG

  >97_primer_pair_2_product
  CCTGTGTAGAAAAAGATGGGCCCTTCGCCCATCTTCCAGAACTTATCTGTTGGAAGTAAA
  TGAGTTTCCATAAGGCCAGGGAAACGCAGGTAGGAACCCATGCGGTCGAGCCAGCACTCA
  CCTGACACTAGGAACCGCTGGCCAAAGGTTTTGTTGCCGAAACTCTCAAAGTTGAAATGG
  TCCATGTATTGCTCAAAATAATTCTCATGAAAGTCAGGGTCTAGAACTCTGTCGGCTGAG
  GGCAGGTGCAGAGACTCAGGAGCTGGTTGGGATCATCAGGGATCTAGGCGGGTCAGGAGG
  AAGGGCAGCCAGTCTGTACTCACCTCTGGCCTGGAGGTTGCACAGTCCCAGTGACAGCAG
  CAGGACCAGGATCCAGGAGGGGACACCATGGTCCACAGGGTAACAAGGATGGAAGTTCAT
  GCTTGATTCTGAGCCGGGCGCTGACTGTCATGTGATTTGGTCACATGACCGACACAACGG
  GCGGGGCAGCATCACGTGATAGTCTGGCGGGGGCTGTCCTACTGTGGCTGGATTCTAGTT
  GGAGGATCAGCCTACTCTTCTTCAGTTTCCCGGTTCCTCCAAATTTCTGGGCTCCTACTT
  GTTTCCACAGAGATGGATACTGTGGAGGTCCAGGAAGCAGAGAGATGGCTAAGGCTCATC
  AGGACCGTATGATCTCCCAAGTGTCCAGCTACTGAGTACCACAAGGTGATGGGTGGGAGG
  GTCCTCCCACGGAAGGATACCGCAGTCCCTAGGGGTTGCAAGCCCCACATGTTCCACTGG
  CTGCTAGAGCTACCTACTCAATCAGCCCTGGGCATCACCATCAGGTACTCGGCCAAAATG
  ACCTCTCTGCTTCCAGTCCTCAGTTCTGGTCAGCACCAGACAGGCCCATAATTACA
  ============================================================

Primers for ID: 98
Primer pair 1, product size : 838
  Primer left                            Primer right
  start: 25211600                        start: 25212417
  end  : 25211621                        end  : 25212437
  tm   : 57                              tm   : 57
  seq  : TGAAATGGTCCATGTATTGCTC          seq  : AGATTCCCAGAAAGTCCAAGG

  >98_primer_pair_1_product
  TGAAATGGTCCATGTATTGCTCAAAATAATTCTCATGAAAGTCAGGGTCTAGAACTCTGT
  CGGCTGAGGGCAGGTGCAGAGACTCAGGAGCTGGTTGGGATCATCAGGGATCTAGGCGGG
  TCAGGAGGAAGGGCAGCCAGTCTGTACTCACCTCTGGCCTGGAGGTTGCACAGTCCCAGT
  GACAGCAGCAGGACCAGGATCCAGGAGGGGACACCATGGTCCACAGGGTAACAAGGATGG
  AAGTTCATGCTTGATTCTGAGCCGGGCGCTGACTGTCATGTGATTTGGTCACATGACCGA
  CACAACGGGCGGGGCAGCATCACGTGATAGTCTGGCGGGGGCTGTCCTACTGTGGCTGGA
  TTCTAGTTGGAGGATCAGCCTACTCTTCTTCAGTTTCCCGGTTCCTCCAAATTTCTGGGC
  TCCTACTTGTTTCCACAGAGATGGATACTGTGGAGGTCCAGGAAGCAGAGAGATGGCTAA
  GGCTCATCAGGACCGTATGATCTCCCAAGTGTCCAGCTACTGAGTACCACAAGGTGATGG
  GTGGGAGGGTCCTCCCACGGAAGGATACCGCAGTCCCTAGGGGTTGCAAGCCCCACATGT
  TCCACTGGCTGCTAGAGCTACCTACTCAATCAGCCCTGGGCATCACCATCAGGTACTCGG
  CCAAAATGACCTCTCTGCTTCCAGTCCTCAGTTCTGGTCAGCACCAGACAGGCCCATAAT
  TACAGAGCCAGGGAAACTGGAACATTTGTCTCCCCTTAGACAGTGGCAGCAGGAAGGTGG
  GGGGTTGTTGCAGAGGAACAGTGTCTCTGAGAGAGGACCTTGGACTTTCTGGGAATCT
  --------------------

Primer pair 2, product size : 836
  Primer left                            Primer right
  start: 25211600                        start: 25212415
  end  : 25211621                        end  : 25212435
  tm   : 57                              tm   : 57
  seq  : TGAAATGGTCCATGTATTGCTC          seq  : ATTCCCAGAAAGTCCAAGGTC

  >98_primer_pair_2_product
  TGAAATGGTCCATGTATTGCTCAAAATAATTCTCATGAAAGTCAGGGTCTAGAACTCTGT
  CGGCTGAGGGCAGGTGCAGAGACTCAGGAGCTGGTTGGGATCATCAGGGATCTAGGCGGG
  TCAGGAGGAAGGGCAGCCAGTCTGTACTCACCTCTGGCCTGGAGGTTGCACAGTCCCAGT
  GACAGCAGCAGGACCAGGATCCAGGAGGGGACACCATGGTCCACAGGGTAACAAGGATGG
  AAGTTCATGCTTGATTCTGAGCCGGGCGCTGACTGTCATGTGATTTGGTCACATGACCGA
  CACAACGGGCGGGGCAGCATCACGTGATAGTCTGGCGGGGGCTGTCCTACTGTGGCTGGA
  TTCTAGTTGGAGGATCAGCCTACTCTTCTTCAGTTTCCCGGTTCCTCCAAATTTCTGGGC
  TCCTACTTGTTTCCACAGAGATGGATACTGTGGAGGTCCAGGAAGCAGAGAGATGGCTAA
  GGCTCATCAGGACCGTATGATCTCCCAAGTGTCCAGCTACTGAGTACCACAAGGTGATGG
  GTGGGAGGGTCCTCCCACGGAAGGATACCGCAGTCCCTAGGGGTTGCAAGCCCCACATGT
  TCCACTGGCTGCTAGAGCTACCTACTCAATCAGCCCTGGGCATCACCATCAGGTACTCGG
  CCAAAATGACCTCTCTGCTTCCAGTCCTCAGTTCTGGTCAGCACCAGACAGGCCCATAAT
  TACAGAGCCAGGGAAACTGGAACATTTGTCTCCCCTTAGACAGTGGCAGCAGGAAGGTGG
  GGGGTTGTTGCAGAGGAACAGTGTCTCTGAGAGAGGACCTTGGACTTTCTGGGAAT
  ============================================================

Primers for ID: 99
Primer pair 1, product size : 831
  Primer left                            Primer right
  start: 25211842                        start: 25212653
  end  : 25211862                        end  : 25212672
  tm   : 57                              tm   : 55
  seq  : GTTCATGCTTGATTCTGAGCC           seq  : ATGGACATGTTCCACTCACG

  >99_primer_pair_1_product
  GTTCATGCTTGATTCTGAGCCGGGCGCTGACTGTCATGTGATTTGGTCACATGACCGACA
  CAACGGGCGGGGCAGCATCACGTGATAGTCTGGCGGGGGCTGTCCTACTGTGGCTGGATT
  CTAGTTGGAGGATCAGCCTACTCTTCTTCAGTTTCCCGGTTCCTCCAAATTTCTGGGCTC
  CTACTTGTTTCCACAGAGATGGATACTGTGGAGGTCCAGGAAGCAGAGAGATGGCTAAGG
  CTCATCAGGACCGTATGATCTCCCAAGTGTCCAGCTACTGAGTACCACAAGGTGATGGGT
  GGGAGGGTCCTCCCACGGAAGGATACCGCAGTCCCTAGGGGTTGCAAGCCCCACATGTTC
  CACTGGCTGCTAGAGCTACCTACTCAATCAGCCCTGGGCATCACCATCAGGTACTCGGCC
  AAAATGACCTCTCTGCTTCCAGTCCTCAGTTCTGGTCAGCACCAGACAGGCCCATAATTA
  CAGAGCCAGGGAAACTGGAACATTTGTCTCCCCTTAGACAGTGGCAGCAGGAAGGTGGGG
  GGTTGTTGCAGAGGAACAGTGTCTCTGAGAGAGGACCTTGGACTTTCTGGGAATCTCTGA
  GCTGCCCGGTTCTCCCCACTGCTGGCACTGTGCCCACAGCCCAAACAGAATGGGGGAGAT
  GGAGGGGCAGGGCTTCTGTGGGAAGCTGCCCTCCACCTCATTGGCACAGAGTGTCTCATT
  GCAGAGAGAAAAAAGGACCAGTTTTCTCTCTGGCACCCAGGTCTGGAAGAGGAGTGACAT
  CCACGGAAGTTGGTGACTTGGACTGGCTGGCCGTGAGTGGAACATGTCCAT
  --------------------

Primer pair 2, product size : 806
  Primer left                            Primer right
  start: 25211867                        start: 25212653
  end  : 25211887                        end  : 25212672
  tm   : 57                              tm   : 55
  seq  : GCTGACTGTCATGTGATTTGG           seq  : ATGGACATGTTCCACTCACG

  >99_primer_pair_2_product
  GCTGACTGTCATGTGATTTGGTCACATGACCGACACAACGGGCGGGGCAGCATCACGTGA
  TAGTCTGGCGGGGGCTGTCCTACTGTGGCTGGATTCTAGTTGGAGGATCAGCCTACTCTT
  CTTCAGTTTCCCGGTTCCTCCAAATTTCTGGGCTCCTACTTGTTTCCACAGAGATGGATA
  CTGTGGAGGTCCAGGAAGCAGAGAGATGGCTAAGGCTCATCAGGACCGTATGATCTCCCA
  AGTGTCCAGCTACTGAGTACCACAAGGTGATGGGTGGGAGGGTCCTCCCACGGAAGGATA
  CCGCAGTCCCTAGGGGTTGCAAGCCCCACATGTTCCACTGGCTGCTAGAGCTACCTACTC
  AATCAGCCCTGGGCATCACCATCAGGTACTCGGCCAAAATGACCTCTCTGCTTCCAGTCC
  TCAGTTCTGGTCAGCACCAGACAGGCCCATAATTACAGAGCCAGGGAAACTGGAACATTT
  GTCTCCCCTTAGACAGTGGCAGCAGGAAGGTGGGGGGTTGTTGCAGAGGAACAGTGTCTC
  TGAGAGAGGACCTTGGACTTTCTGGGAATCTCTGAGCTGCCCGGTTCTCCCCACTGCTGG
  CACTGTGCCCACAGCCCAAACAGAATGGGGGAGATGGAGGGGCAGGGCTTCTGTGGGAAG
  CTGCCCTCCACCTCATTGGCACAGAGTGTCTCATTGCAGAGAGAAAAAAGGACCAGTTTT
  CTCTCTGGCACCCAGGTCTGGAAGAGGAGTGACATCCACGGAAGTTGGTGACTTGGACTG
  GCTGGCCGTGAGTGGAACATGTCCAT
  ============================================================

Primers for ID: 100
Primer pair 1, product size : 782
  Primer left                            Primer right
  start: 25209909                        start: 25210670
  end  : 25209929                        end  : 25210690
  tm   : 57                              tm   : 57
  seq  : GATGGCATGAAAGCAGATAGC           seq  : AGTTTCAGGTCCTTCTTTCCC

  >100_primer_pair_1_product
  GATGGCATGAAAGCAGATAGCTACAAGGCTCTTGGACCAGGCTAGCACTGGGTCCTGCAC
  CCAGGGAGAGCCACCTCACCTTGACAGGGTTGGCAGGAAGGGGCCCTAGAAAGTCAGTAG
  GATACGGGTAGTCCATCATGGCGAGCACAGTAAATGCATTTCGGGCAAACCCAAAGAGCT
  GAGTCAGGTCCTTTGGGCTGGAAAGTGATTGACAGGTACCAAAGTTCTGGCTGATGGTGT
  CATAGGCTGGGAAGAGAGAGGCCAGGAGAAAAGGCTGAGGAAACTGCTGGCAAATGTGAA
  GGGCAAGAATGAATGCCCAAGGTGGGCAGCAGGTGAGGAAAGAGTCCCTCACCTCCCTGG
  AGGAACAAGTCTTTGATTTGCTGAAAGGCATCCCGCACAGCCTGGGCGCACTTGGGACTC
  TGGCCATAAAAGTCCTGGAGAAGAGACCAAGGTTGCTGCTGCCATTCTTGCACTGGCCTG
  GGGTACCCAAGTCCCCTCACTCACCGCTGTGACATCTCGGAAGAATTGGTAGGAGTCCCC
  AAGGCCTGCAACAGCTACAACAGGAGCGCTGGCTGCCAGTGCCCCAGCCACCAGGTGGGG
  GTACTTCATCCTCATGTAGGCACTCAGCATCCCCCCATAACTGGGAGTACAGAGCACAGA
  TCATGGTTGTGGGAAGCTGCCCACAACTCAGGCGAGCAGCCTCACTGTCCTCCAGGCTGA
  GGTGCTAGGCTGCTCTTTCCCTGCTCAGAACGCCCAAGGGTGGGAAAGAAGGACCTGAAA
  CT
  --------------------

Primer pair 2, product size : 779
  Primer left                            Primer right
  start: 25209909                        start: 25210668
  end  : 25209929                        end  : 25210687
  tm   : 57                              tm   : 55
  seq  : GATGGCATGAAAGCAGATAGC           seq  : TTCAGGTCCTTCTTTCCCAC

  >100_primer_pair_2_product
  GATGGCATGAAAGCAGATAGCTACAAGGCTCTTGGACCAGGCTAGCACTGGGTCCTGCAC
  CCAGGGAGAGCCACCTCACCTTGACAGGGTTGGCAGGAAGGGGCCCTAGAAAGTCAGTAG
  GATACGGGTAGTCCATCATGGCGAGCACAGTAAATGCATTTCGGGCAAACCCAAAGAGCT
  GAGTCAGGTCCTTTGGGCTGGAAAGTGATTGACAGGTACCAAAGTTCTGGCTGATGGTGT
  CATAGGCTGGGAAGAGAGAGGCCAGGAGAAAAGGCTGAGGAAACTGCTGGCAAATGTGAA
  GGGCAAGAATGAATGCCCAAGGTGGGCAGCAGGTGAGGAAAGAGTCCCTCACCTCCCTGG
  AGGAACAAGTCTTTGATTTGCTGAAAGGCATCCCGCACAGCCTGGGCGCACTTGGGACTC
  TGGCCATAAAAGTCCTGGAGAAGAGACCAAGGTTGCTGCTGCCATTCTTGCACTGGCCTG
  GGGTACCCAAGTCCCCTCACTCACCGCTGTGACATCTCGGAAGAATTGGTAGGAGTCCCC
  AAGGCCTGCAACAGCTACAACAGGAGCGCTGGCTGCCAGTGCCCCAGCCACCAGGTGGGG
  GTACTTCATCCTCATGTAGGCACTCAGCATCCCCCCATAACTGGGAGTACAGAGCACAGA
  TCATGGTTGTGGGAAGCTGCCCACAACTCAGGCGAGCAGCCTCACTGTCCTCCAGGCTGA
  GGTGCTAGGCTGCTCTTTCCCTGCTCAGAACGCCCAAGGGTGGGAAAGAAGGACCTGAA
  ============================================================

Primers for ID: 101
Primer pair 1, product size : 881
  Primer left                            Primer right
  start: 25209909                        start: 25210770
  end  : 25209929                        end  : 25210789
  tm   : 57                              tm   : 55
  seq  : GATGGCATGAAAGCAGATAGC           seq  : TGTCTTCATTGCTGGGTCAG

  >101_primer_pair_1_product
  GATGGCATGAAAGCAGATAGCTACAAGGCTCTTGGACCAGGCTAGCACTGGGTCCTGCAC
  CCAGGGAGAGCCACCTCACCTTGACAGGGTTGGCAGGAAGGGGCCCTAGAAAGTCAGTAG
  GATACGGGTAGTCCATCATGGCGAGCACAGTAAATGCATTTCGGGCAAACCCAAAGAGCT
  GAGTCAGGTCCTTTGGGCTGGAAAGTGATTGACAGGTACCAAAGTTCTGGCTGATGGTGT
  CATAGGCTGGGAAGAGAGAGGCCAGGAGAAAAGGCTGAGGAAACTGCTGGCAAATGTGAA
  GGGCAAGAATGAATGCCCAAGGTGGGCAGCAGGTGAGGAAAGAGTCCCTCACCTCCCTGG
  AGGAACAAGTCTTTGATTTGCTGAAAGGCATCCCGCACAGCCTGGGCGCACTTGGGACTC
  TGGCCATAAAAGTCCTGGAGAAGAGACCAAGGTTGCTGCTGCCATTCTTGCACTGGCCTG
  GGGTACCCAAGTCCCCTCACTCACCGCTGTGACATCTCGGAAGAATTGGTAGGAGTCCCC
  AAGGCCTGCAACAGCTACAACAGGAGCGCTGGCTGCCAGTGCCCCAGCCACCAGGTGGGG
  GTACTTCATCCTCATGTAGGCACTCAGCATCCCCCCATAACTGGGAGTACAGAGCACAGA
  TCATGGTTGTGGGAAGCTGCCCACAACTCAGGCGAGCAGCCTCACTGTCCTCCAGGCTGA
  GGTGCTAGGCTGCTCTTTCCCTGCTCAGAACGCCCAAGGGTGGGAAAGAAGGACCTGAAA
  CTGTCAGGCCCACACACCCTGATCCCAGGGCCAAGGCAGATACAGCCTTCACTGGGAGAA
  GGCACCTGTGGGTGCCCTGCCCTGACCCAGCAATGAAGACA
  --------------------

Primer pair 2, product size : 782
  Primer left                            Primer right
  start: 25209909                        start: 25210670
  end  : 25209929                        end  : 25210690
  tm   : 57                              tm   : 57
  seq  : GATGGCATGAAAGCAGATAGC           seq  : AGTTTCAGGTCCTTCTTTCCC

  >101_primer_pair_2_product
  GATGGCATGAAAGCAGATAGCTACAAGGCTCTTGGACCAGGCTAGCACTGGGTCCTGCAC
  CCAGGGAGAGCCACCTCACCTTGACAGGGTTGGCAGGAAGGGGCCCTAGAAAGTCAGTAG
  GATACGGGTAGTCCATCATGGCGAGCACAGTAAATGCATTTCGGGCAAACCCAAAGAGCT
  GAGTCAGGTCCTTTGGGCTGGAAAGTGATTGACAGGTACCAAAGTTCTGGCTGATGGTGT
  CATAGGCTGGGAAGAGAGAGGCCAGGAGAAAAGGCTGAGGAAACTGCTGGCAAATGTGAA
  GGGCAAGAATGAATGCCCAAGGTGGGCAGCAGGTGAGGAAAGAGTCCCTCACCTCCCTGG
  AGGAACAAGTCTTTGATTTGCTGAAAGGCATCCCGCACAGCCTGGGCGCACTTGGGACTC
  TGGCCATAAAAGTCCTGGAGAAGAGACCAAGGTTGCTGCTGCCATTCTTGCACTGGCCTG
  GGGTACCCAAGTCCCCTCACTCACCGCTGTGACATCTCGGAAGAATTGGTAGGAGTCCCC
  AAGGCCTGCAACAGCTACAACAGGAGCGCTGGCTGCCAGTGCCCCAGCCACCAGGTGGGG
  GTACTTCATCCTCATGTAGGCACTCAGCATCCCCCCATAACTGGGAGTACAGAGCACAGA
  TCATGGTTGTGGGAAGCTGCCCACAACTCAGGCGAGCAGCCTCACTGTCCTCCAGGCTGA
  GGTGCTAGGCTGCTCTTTCCCTGCTCAGAACGCCCAAGGGTGGGAAAGAAGGACCTGAAA
  CT
  ============================================================

Primers for ID: 102
Primer pair 1, product size : 762
  Primer left                            Primer right
  start: 25210129                        start: 25210871
  end  : 25210149                        end  : 25210890
  tm   : 57                              tm   : 55
  seq  : AAAGTTCTGGCTGATGGTGTC           seq  : TTTACCTCCTTCTGGAAGCC

  >102_primer_pair_1_product
  AAAGTTCTGGCTGATGGTGTCATAGGCTGGGAAGAGAGAGGCCAGGAGAAAAGGCTGAGG
  AAACTGCTGGCAAATGTGAAGGGCAAGAATGAATGCCCAAGGTGGGCAGCAGGTGAGGAA
  AGAGTCCCTCACCTCCCTGGAGGAACAAGTCTTTGATTTGCTGAAAGGCATCCCGCACAG
  CCTGGGCGCACTTGGGACTCTGGCCATAAAAGTCCTGGAGAAGAGACCAAGGTTGCTGCT
  GCCATTCTTGCACTGGCCTGGGGTACCCAAGTCCCCTCACTCACCGCTGTGACATCTCGG
  AAGAATTGGTAGGAGTCCCCAAGGCCTGCAACAGCTACAACAGGAGCGCTGGCTGCCAGT
  GCCCCAGCCACCAGGTGGGGGTACTTCATCCTCATGTAGGCACTCAGCATCCCCCCATAA
  CTGGGAGTACAGAGCACAGATCATGGTTGTGGGAAGCTGCCCACAACTCAGGCGAGCAGC
  CTCACTGTCCTCCAGGCTGAGGTGCTAGGCTGCTCTTTCCCTGCTCAGAACGCCCAAGGG
  TGGGAAAGAAGGACCTGAAACTGTCAGGCCCACACACCCTGATCCCAGGGCCAAGGCAGA
  TACAGCCTTCACTGGGAGAAGGCACCTGTGGGTGCCCTGCCCTGACCCAGCAATGAAGAC
  ATTGCAGAGACAAAGTCAGAAGGAATTGTCCCACTAGTGGGAACAACATAGCATACACTG
  CCTATGAGGTCCACTCAAGGAGGGCTTCCAGAAGGAGGTAAA
  --------------------

Primer pair 2, product size : 719
  Primer left                            Primer right
  start: 25210172                        start: 25210871
  end  : 25210192                        end  : 25210890
  tm   : 57                              tm   : 55
  seq  : AGGAGAAAAGGCTGAGGAAAC           seq  : TTTACCTCCTTCTGGAAGCC

  >102_primer_pair_2_product
  AGGAGAAAAGGCTGAGGAAACTGCTGGCAAATGTGAAGGGCAAGAATGAATGCCCAAGGT
  GGGCAGCAGGTGAGGAAAGAGTCCCTCACCTCCCTGGAGGAACAAGTCTTTGATTTGCTG
  AAAGGCATCCCGCACAGCCTGGGCGCACTTGGGACTCTGGCCATAAAAGTCCTGGAGAAG
  AGACCAAGGTTGCTGCTGCCATTCTTGCACTGGCCTGGGGTACCCAAGTCCCCTCACTCA
  CCGCTGTGACATCTCGGAAGAATTGGTAGGAGTCCCCAAGGCCTGCAACAGCTACAACAG
  GAGCGCTGGCTGCCAGTGCCCCAGCCACCAGGTGGGGGTACTTCATCCTCATGTAGGCAC
  TCAGCATCCCCCCATAACTGGGAGTACAGAGCACAGATCATGGTTGTGGGAAGCTGCCCA
  CAACTCAGGCGAGCAGCCTCACTGTCCTCCAGGCTGAGGTGCTAGGCTGCTCTTTCCCTG
  CTCAGAACGCCCAAGGGTGGGAAAGAAGGACCTGAAACTGTCAGGCCCACACACCCTGAT
  CCCAGGGCCAAGGCAGATACAGCCTTCACTGGGAGAAGGCACCTGTGGGTGCCCTGCCCT
  GACCCAGCAATGAAGACATTGCAGAGACAAAGTCAGAAGGAATTGTCCCACTAGTGGGAA
  CAACATAGCATACACTGCCTATGAGGTCCACTCAAGGAGGGCTTCCAGAAGGAGGTAAA
  ============================================================

Primers for ID: 103
Primer pair 1, product size : 843
  Primer left                            Primer right
  start: 25210129                        start: 25210951
  end  : 25210149                        end  : 25210971
  tm   : 57                              tm   : 57
  seq  : AAAGTTCTGGCTGATGGTGTC           seq  : TATACAAGGAGGGCCAAAGAG

  >103_primer_pair_1_product
  AAAGTTCTGGCTGATGGTGTCATAGGCTGGGAAGAGAGAGGCCAGGAGAAAAGGCTGAGG
  AAACTGCTGGCAAATGTGAAGGGCAAGAATGAATGCCCAAGGTGGGCAGCAGGTGAGGAA
  AGAGTCCCTCACCTCCCTGGAGGAACAAGTCTTTGATTTGCTGAAAGGCATCCCGCACAG
  CCTGGGCGCACTTGGGACTCTGGCCATAAAAGTCCTGGAGAAGAGACCAAGGTTGCTGCT
  GCCATTCTTGCACTGGCCTGGGGTACCCAAGTCCCCTCACTCACCGCTGTGACATCTCGG
  AAGAATTGGTAGGAGTCCCCAAGGCCTGCAACAGCTACAACAGGAGCGCTGGCTGCCAGT
  GCCCCAGCCACCAGGTGGGGGTACTTCATCCTCATGTAGGCACTCAGCATCCCCCCATAA
  CTGGGAGTACAGAGCACAGATCATGGTTGTGGGAAGCTGCCCACAACTCAGGCGAGCAGC
  CTCACTGTCCTCCAGGCTGAGGTGCTAGGCTGCTCTTTCCCTGCTCAGAACGCCCAAGGG
  TGGGAAAGAAGGACCTGAAACTGTCAGGCCCACACACCCTGATCCCAGGGCCAAGGCAGA
  TACAGCCTTCACTGGGAGAAGGCACCTGTGGGTGCCCTGCCCTGACCCAGCAATGAAGAC
  ATTGCAGAGACAAAGTCAGAAGGAATTGTCCCACTAGTGGGAACAACATAGCATACACTG
  CCTATGAGGTCCACTCAAGGAGGGCTTCCAGAAGGAGGTAAAGCTAGACCCCGCCCTTCC
  ACATGTGGGGTAGGCATAGGATGTTGAGACTGTAAGAGACATCTCTTTGGCCCTCCTTGT
  ATA
  --------------------

Primer pair 2, product size : 844
  Primer left                            Primer right
  start: 25210129                        start: 25210951
  end  : 25210149                        end  : 25210972
  tm   : 57                              tm   : 61
  seq  : AAAGTTCTGGCTGATGGTGTC           seq  : CTATACAAGGAGGGCCAAAGAG

  >103_primer_pair_2_product
  AAAGTTCTGGCTGATGGTGTCATAGGCTGGGAAGAGAGAGGCCAGGAGAAAAGGCTGAGG
  AAACTGCTGGCAAATGTGAAGGGCAAGAATGAATGCCCAAGGTGGGCAGCAGGTGAGGAA
  AGAGTCCCTCACCTCCCTGGAGGAACAAGTCTTTGATTTGCTGAAAGGCATCCCGCACAG
  CCTGGGCGCACTTGGGACTCTGGCCATAAAAGTCCTGGAGAAGAGACCAAGGTTGCTGCT
  GCCATTCTTGCACTGGCCTGGGGTACCCAAGTCCCCTCACTCACCGCTGTGACATCTCGG
  AAGAATTGGTAGGAGTCCCCAAGGCCTGCAACAGCTACAACAGGAGCGCTGGCTGCCAGT
  GCCCCAGCCACCAGGTGGGGGTACTTCATCCTCATGTAGGCACTCAGCATCCCCCCATAA
  CTGGGAGTACAGAGCACAGATCATGGTTGTGGGAAGCTGCCCACAACTCAGGCGAGCAGC
  CTCACTGTCCTCCAGGCTGAGGTGCTAGGCTGCTCTTTCCCTGCTCAGAACGCCCAAGGG
  TGGGAAAGAAGGACCTGAAACTGTCAGGCCCACACACCCTGATCCCAGGGCCAAGGCAGA
  TACAGCCTTCACTGGGAGAAGGCACCTGTGGGTGCCCTGCCCTGACCCAGCAATGAAGAC
  ATTGCAGAGACAAAGTCAGAAGGAATTGTCCCACTAGTGGGAACAACATAGCATACACTG
  CCTATGAGGTCCACTCAAGGAGGGCTTCCAGAAGGAGGTAAAGCTAGACCCCGCCCTTCC
  ACATGTGGGGTAGGCATAGGATGTTGAGACTGTAAGAGACATCTCTTTGGCCCTCCTTGT
  ATAG
  ============================================================

Primers for ID: 104
Primer pair 1, product size : 880
  Primer left                            Primer right
  start: 25210172                        start: 25211030
  end  : 25210192                        end  : 25211051
  tm   : 57                              tm   : 61
  seq  : AGGAGAAAAGGCTGAGGAAAC           seq  : AGATCTGGACCCATGAGAGAAC

  >104_primer_pair_1_product
  AGGAGAAAAGGCTGAGGAAACTGCTGGCAAATGTGAAGGGCAAGAATGAATGCCCAAGGT
  GGGCAGCAGGTGAGGAAAGAGTCCCTCACCTCCCTGGAGGAACAAGTCTTTGATTTGCTG
  AAAGGCATCCCGCACAGCCTGGGCGCACTTGGGACTCTGGCCATAAAAGTCCTGGAGAAG
  AGACCAAGGTTGCTGCTGCCATTCTTGCACTGGCCTGGGGTACCCAAGTCCCCTCACTCA
  CCGCTGTGACATCTCGGAAGAATTGGTAGGAGTCCCCAAGGCCTGCAACAGCTACAACAG
  GAGCGCTGGCTGCCAGTGCCCCAGCCACCAGGTGGGGGTACTTCATCCTCATGTAGGCAC
  TCAGCATCCCCCCATAACTGGGAGTACAGAGCACAGATCATGGTTGTGGGAAGCTGCCCA
  CAACTCAGGCGAGCAGCCTCACTGTCCTCCAGGCTGAGGTGCTAGGCTGCTCTTTCCCTG
  CTCAGAACGCCCAAGGGTGGGAAAGAAGGACCTGAAACTGTCAGGCCCACACACCCTGAT
  CCCAGGGCCAAGGCAGATACAGCCTTCACTGGGAGAAGGCACCTGTGGGTGCCCTGCCCT
  GACCCAGCAATGAAGACATTGCAGAGACAAAGTCAGAAGGAATTGTCCCACTAGTGGGAA
  CAACATAGCATACACTGCCTATGAGGTCCACTCAAGGAGGGCTTCCAGAAGGAGGTAAAG
  CTAGACCCCGCCCTTCCACATGTGGGGTAGGCATAGGATGTTGAGACTGTAAGAGACATC
  TCTTTGGCCCTCCTTGTATAGGGTGTCAATCGGCACAACAGGGTGGAGCCTTAGAGTAGG
  GTAAGATTAGGACTCTAGGTTCTCTCATGGGTCCAGATCT
  --------------------

Primer pair 2, product size : 775
  Primer left                            Primer right
  start: 25210277                        start: 25211030
  end  : 25210297                        end  : 25211051
  tm   : 55                              tm   : 61
  seq  : GTCTTTGATTTGCTGAAAGGC           seq  : AGATCTGGACCCATGAGAGAAC

  >104_primer_pair_2_product
  GTCTTTGATTTGCTGAAAGGCATCCCGCACAGCCTGGGCGCACTTGGGACTCTGGCCATA
  AAAGTCCTGGAGAAGAGACCAAGGTTGCTGCTGCCATTCTTGCACTGGCCTGGGGTACCC
  AAGTCCCCTCACTCACCGCTGTGACATCTCGGAAGAATTGGTAGGAGTCCCCAAGGCCTG
  CAACAGCTACAACAGGAGCGCTGGCTGCCAGTGCCCCAGCCACCAGGTGGGGGTACTTCA
  TCCTCATGTAGGCACTCAGCATCCCCCCATAACTGGGAGTACAGAGCACAGATCATGGTT
  GTGGGAAGCTGCCCACAACTCAGGCGAGCAGCCTCACTGTCCTCCAGGCTGAGGTGCTAG
  GCTGCTCTTTCCCTGCTCAGAACGCCCAAGGGTGGGAAAGAAGGACCTGAAACTGTCAGG
  CCCACACACCCTGATCCCAGGGCCAAGGCAGATACAGCCTTCACTGGGAGAAGGCACCTG
  TGGGTGCCCTGCCCTGACCCAGCAATGAAGACATTGCAGAGACAAAGTCAGAAGGAATTG
  TCCCACTAGTGGGAACAACATAGCATACACTGCCTATGAGGTCCACTCAAGGAGGGCTTC
  CAGAAGGAGGTAAAGCTAGACCCCGCCCTTCCACATGTGGGGTAGGCATAGGATGTTGAG
  ACTGTAAGAGACATCTCTTTGGCCCTCCTTGTATAGGGTGTCAATCGGCACAACAGGGTG
  GAGCCTTAGAGTAGGGTAAGATTAGGACTCTAGGTTCTCTCATGGGTCCAGATCT
  ============================================================

Primers for ID: 114
Primer pair 1, product size : 715
  Primer left                            Primer right
  start: 25211383                        start: 25212076
  end  : 25211403                        end  : 25212097
  tm   : 57                              tm   : 61
  seq  : ATGAAGCCAGAGTTGTTAGCG           seq  : ATACGGTCCTGATGAGCCTTAG

  >114_primer_pair_1_product
  ATGAAGCCAGAGTTGTTAGCGAAGGACCAGATATCCCCCTCATTCCCTGTGTAGAAAAAG
  ATGGGCCCTTCGCCCATCTTCCAGAACTTATCTGTTGGAAGTAAATGAGTTTCCATAAGG
  CCAGGGAAACGCAGGTAGGAACCCATGCGGTCGAGCCAGCACTCACCTGACACTAGGAAC
  CGCTGGCCAAAGGTTTTGTTGCCGAAACTCTCAAAGTTGAAATGGTCCATGTATTGCTCA
  AAATAATTCTCATGAAAGTCAGGGTCTAGAACTCTGTCGGCTGAGGGCAGGTGCAGAGAC
  TCAGGAGCTGGTTGGGATCATCAGGGATCTAGGCGGGTCAGGAGGAAGGGCAGCCAGTCT
  GTACTCACCTCTGGCCTGGAGGTTGCACAGTCCCAGTGACAGCAGCAGGACCAGGATCCA
  GGAGGGGACACCATGGTCCACAGGGTAACAAGGATGGAAGTTCATGCTTGATTCTGAGCC
  GGGCGCTGACTGTCATGTGATTTGGTCACATGACCGACACAACGGGCGGGGCAGCATCAC
  GTGATAGTCTGGCGGGGGCTGTCCTACTGTGGCTGGATTCTAGTTGGAGGATCAGCCTAC
  TCTTCTTCAGTTTCCCGGTTCCTCCAAATTTCTGGGCTCCTACTTGTTTCCACAGAGATG
  GATACTGTGGAGGTCCAGGAAGCAGAGAGATGGCTAAGGCTCATCAGGACCGTAT
  --------------------

Primer pair 2, product size : 777
  Primer left                            Primer right
  start: 25211226                        start: 25211982
  end  : 25211245                        end  : 25212002
  tm   : 55                              tm   : 57
  seq  : ATTTCCCATAGTACCGCTGC            seq  : AACCGGGAAACTGAAGAAGAG

  >114_primer_pair_2_product
  ATTTCCCATAGTACCGCTGCAGAAAGCAGGAAGGGATGGCTAATCCACTCCTCGGTGCTC
  CCCACCTCCTTCAACTCAGGGACTGCCAGGAACTGTACAGGTACCCACGTGCTCAGCAAA
  GACAAGCAGGGCCTCCTGCTGGGCTGCCAGTTCCACCATGAAGCCAGAGTTGTTAGCGAA
  GGACCAGATATCCCCCTCATTCCCTGTGTAGAAAAAGATGGGCCCTTCGCCCATCTTCCA
  GAACTTATCTGTTGGAAGTAAATGAGTTTCCATAAGGCCAGGGAAACGCAGGTAGGAACC
  CATGCGGTCGAGCCAGCACTCACCTGACACTAGGAACCGCTGGCCAAAGGTTTTGTTGCC
  GAAACTCTCAAAGTTGAAATGGTCCATGTATTGCTCAAAATAATTCTCATGAAAGTCAGG
  GTCTAGAACTCTGTCGGCTGAGGGCAGGTGCAGAGACTCAGGAGCTGGTTGGGATCATCA
  GGGATCTAGGCGGGTCAGGAGGAAGGGCAGCCAGTCTGTACTCACCTCTGGCCTGGAGGT
  TGCACAGTCCCAGTGACAGCAGCAGGACCAGGATCCAGGAGGGGACACCATGGTCCACAG
  GGTAACAAGGATGGAAGTTCATGCTTGATTCTGAGCCGGGCGCTGACTGTCATGTGATTT
  GGTCACATGACCGACACAACGGGCGGGGCAGCATCACGTGATAGTCTGGCGGGGGCTGTC
  CTACTGTGGCTGGATTCTAGTTGGAGGATCAGCCTACTCTTCTTCAGTTTCCCGGTT
  ============================================================

Primers for ID: 115
Primer pair 1, product size : 715
  Primer left                            Primer right
  start: 25211383                        start: 25212076
  end  : 25211403                        end  : 25212097
  tm   : 57                              tm   : 61
  seq  : ATGAAGCCAGAGTTGTTAGCG           seq  : ATACGGTCCTGATGAGCCTTAG

  >115_primer_pair_1_product
  ATGAAGCCAGAGTTGTTAGCGAAGGACCAGATATCCCCCTCATTCCCTGTGTAGAAAAAG
  ATGGGCCCTTCGCCCATCTTCCAGAACTTATCTGTTGGAAGTAAATGAGTTTCCATAAGG
  CCAGGGAAACGCAGGTAGGAACCCATGCGGTCGAGCCAGCACTCACCTGACACTAGGAAC
  CGCTGGCCAAAGGTTTTGTTGCCGAAACTCTCAAAGTTGAAATGGTCCATGTATTGCTCA
  AAATAATTCTCATGAAAGTCAGGGTCTAGAACTCTGTCGGCTGAGGGCAGGTGCAGAGAC
  TCAGGAGCTGGTTGGGATCATCAGGGATCTAGGCGGGTCAGGAGGAAGGGCAGCCAGTCT
  GTACTCACCTCTGGCCTGGAGGTTGCACAGTCCCAGTGACAGCAGCAGGACCAGGATCCA
  GGAGGGGACACCATGGTCCACAGGGTAACAAGGATGGAAGTTCATGCTTGATTCTGAGCC
  GGGCGCTGACTGTCATGTGATTTGGTCACATGACCGACACAACGGGCGGGGCAGCATCAC
  GTGATAGTCTGGCGGGGGCTGTCCTACTGTGGCTGGATTCTAGTTGGAGGATCAGCCTAC
  TCTTCTTCAGTTTCCCGGTTCCTCCAAATTTCTGGGCTCCTACTTGTTTCCACAGAGATG
  GATACTGTGGAGGTCCAGGAAGCAGAGAGATGGCTAAGGCTCATCAGGACCGTAT
  --------------------

Primer pair 2, product size : 707
  Primer left                            Primer right
  start: 25211391                        start: 25212076
  end  : 25211412                        end  : 25212097
  tm   : 61                              tm   : 61
  seq  : AGAGTTGTTAGCGAAGGACCAG          seq  : ATACGGTCCTGATGAGCCTTAG

  >115_primer_pair_2_product
  AGAGTTGTTAGCGAAGGACCAGATATCCCCCTCATTCCCTGTGTAGAAAAAGATGGGCCC
  TTCGCCCATCTTCCAGAACTTATCTGTTGGAAGTAAATGAGTTTCCATAAGGCCAGGGAA
  ACGCAGGTAGGAACCCATGCGGTCGAGCCAGCACTCACCTGACACTAGGAACCGCTGGCC
  AAAGGTTTTGTTGCCGAAACTCTCAAAGTTGAAATGGTCCATGTATTGCTCAAAATAATT
  CTCATGAAAGTCAGGGTCTAGAACTCTGTCGGCTGAGGGCAGGTGCAGAGACTCAGGAGC
  TGGTTGGGATCATCAGGGATCTAGGCGGGTCAGGAGGAAGGGCAGCCAGTCTGTACTCAC
  CTCTGGCCTGGAGGTTGCACAGTCCCAGTGACAGCAGCAGGACCAGGATCCAGGAGGGGA
  CACCATGGTCCACAGGGTAACAAGGATGGAAGTTCATGCTTGATTCTGAGCCGGGCGCTG
  ACTGTCATGTGATTTGGTCACATGACCGACACAACGGGCGGGGCAGCATCACGTGATAGT
  CTGGCGGGGGCTGTCCTACTGTGGCTGGATTCTAGTTGGAGGATCAGCCTACTCTTCTTC
  AGTTTCCCGGTTCCTCCAAATTTCTGGGCTCCTACTTGTTTCCACAGAGATGGATACTGT
  GGAGGTCCAGGAAGCAGAGAGATGGCTAAGGCTCATCAGGACCGTAT
  ============================================================

Primers for ID: 116
Primer pair 1, product size : 939
  Primer left                            Primer right
  start: 25211383                        start: 25212301
  end  : 25211403                        end  : 25212321
  tm   : 57                              tm   : 57
  seq  : ATGAAGCCAGAGTTGTTAGCG           seq  : TAATTATGGGCCTGTCTGGTG

  >116_primer_pair_1_product
  ATGAAGCCAGAGTTGTTAGCGAAGGACCAGATATCCCCCTCATTCCCTGTGTAGAAAAAG
  ATGGGCCCTTCGCCCATCTTCCAGAACTTATCTGTTGGAAGTAAATGAGTTTCCATAAGG
  CCAGGGAAACGCAGGTAGGAACCCATGCGGTCGAGCCAGCACTCACCTGACACTAGGAAC
  CGCTGGCCAAAGGTTTTGTTGCCGAAACTCTCAAAGTTGAAATGGTCCATGTATTGCTCA
  AAATAATTCTCATGAAAGTCAGGGTCTAGAACTCTGTCGGCTGAGGGCAGGTGCAGAGAC
  TCAGGAGCTGGTTGGGATCATCAGGGATCTAGGCGGGTCAGGAGGAAGGGCAGCCAGTCT
  GTACTCACCTCTGGCCTGGAGGTTGCACAGTCCCAGTGACAGCAGCAGGACCAGGATCCA
  GGAGGGGACACCATGGTCCACAGGGTAACAAGGATGGAAGTTCATGCTTGATTCTGAGCC
  GGGCGCTGACTGTCATGTGATTTGGTCACATGACCGACACAACGGGCGGGGCAGCATCAC
  GTGATAGTCTGGCGGGGGCTGTCCTACTGTGGCTGGATTCTAGTTGGAGGATCAGCCTAC
  TCTTCTTCAGTTTCCCGGTTCCTCCAAATTTCTGGGCTCCTACTTGTTTCCACAGAGATG
  GATACTGTGGAGGTCCAGGAAGCAGAGAGATGGCTAAGGCTCATCAGGACCGTATGATCT
  CCCAAGTGTCCAGCTACTGAGTACCACAAGGTGATGGGTGGGAGGGTCCTCCCACGGAAG
  GATACCGCAGTCCCTAGGGGTTGCAAGCCCCACATGTTCCACTGGCTGCTAGAGCTACCT
  ACTCAATCAGCCCTGGGCATCACCATCAGGTACTCGGCCAAAATGACCTCTCTGCTTCCA
  GTCCTCAGTTCTGGTCAGCACCAGACAGGCCCATAATTA
  --------------------

Primer pair 2, product size : 941
  Primer left                            Primer right
  start: 25211383                        start: 25212303
  end  : 25211403                        end  : 25212323
  tm   : 57                              tm   : 57
  seq  : ATGAAGCCAGAGTTGTTAGCG           seq  : TGTAATTATGGGCCTGTCTGG

  >116_primer_pair_2_product
  ATGAAGCCAGAGTTGTTAGCGAAGGACCAGATATCCCCCTCATTCCCTGTGTAGAAAAAG
  ATGGGCCCTTCGCCCATCTTCCAGAACTTATCTGTTGGAAGTAAATGAGTTTCCATAAGG
  CCAGGGAAACGCAGGTAGGAACCCATGCGGTCGAGCCAGCACTCACCTGACACTAGGAAC
  CGCTGGCCAAAGGTTTTGTTGCCGAAACTCTCAAAGTTGAAATGGTCCATGTATTGCTCA
  AAATAATTCTCATGAAAGTCAGGGTCTAGAACTCTGTCGGCTGAGGGCAGGTGCAGAGAC
  TCAGGAGCTGGTTGGGATCATCAGGGATCTAGGCGGGTCAGGAGGAAGGGCAGCCAGTCT
  GTACTCACCTCTGGCCTGGAGGTTGCACAGTCCCAGTGACAGCAGCAGGACCAGGATCCA
  GGAGGGGACACCATGGTCCACAGGGTAACAAGGATGGAAGTTCATGCTTGATTCTGAGCC
  GGGCGCTGACTGTCATGTGATTTGGTCACATGACCGACACAACGGGCGGGGCAGCATCAC
  GTGATAGTCTGGCGGGGGCTGTCCTACTGTGGCTGGATTCTAGTTGGAGGATCAGCCTAC
  TCTTCTTCAGTTTCCCGGTTCCTCCAAATTTCTGGGCTCCTACTTGTTTCCACAGAGATG
  GATACTGTGGAGGTCCAGGAAGCAGAGAGATGGCTAAGGCTCATCAGGACCGTATGATCT
  CCCAAGTGTCCAGCTACTGAGTACCACAAGGTGATGGGTGGGAGGGTCCTCCCACGGAAG
  GATACCGCAGTCCCTAGGGGTTGCAAGCCCCACATGTTCCACTGGCTGCTAGAGCTACCT
  ACTCAATCAGCCCTGGGCATCACCATCAGGTACTCGGCCAAAATGACCTCTCTGCTTCCA
  GTCCTCAGTTCTGGTCAGCACCAGACAGGCCCATAATTACA
  ============================================================

Primers for ID: 117
Primer pair 1, product size : 722
  Primer left                            Primer right
  start: 25211600                        start: 25212301
  end  : 25211621                        end  : 25212321
  tm   : 57                              tm   : 57
  seq  : TGAAATGGTCCATGTATTGCTC          seq  : TAATTATGGGCCTGTCTGGTG

  >117_primer_pair_1_product
  TGAAATGGTCCATGTATTGCTCAAAATAATTCTCATGAAAGTCAGGGTCTAGAACTCTGT
  CGGCTGAGGGCAGGTGCAGAGACTCAGGAGCTGGTTGGGATCATCAGGGATCTAGGCGGG
  TCAGGAGGAAGGGCAGCCAGTCTGTACTCACCTCTGGCCTGGAGGTTGCACAGTCCCAGT
  GACAGCAGCAGGACCAGGATCCAGGAGGGGACACCATGGTCCACAGGGTAACAAGGATGG
  AAGTTCATGCTTGATTCTGAGCCGGGCGCTGACTGTCATGTGATTTGGTCACATGACCGA
  CACAACGGGCGGGGCAGCATCACGTGATAGTCTGGCGGGGGCTGTCCTACTGTGGCTGGA
  TTCTAGTTGGAGGATCAGCCTACTCTTCTTCAGTTTCCCGGTTCCTCCAAATTTCTGGGC
  TCCTACTTGTTTCCACAGAGATGGATACTGTGGAGGTCCAGGAAGCAGAGAGATGGCTAA
  GGCTCATCAGGACCGTATGATCTCCCAAGTGTCCAGCTACTGAGTACCACAAGGTGATGG
  GTGGGAGGGTCCTCCCACGGAAGGATACCGCAGTCCCTAGGGGTTGCAAGCCCCACATGT
  TCCACTGGCTGCTAGAGCTACCTACTCAATCAGCCCTGGGCATCACCATCAGGTACTCGG
  CCAAAATGACCTCTCTGCTTCCAGTCCTCAGTTCTGGTCAGCACCAGACAGGCCCATAAT
  TA
  --------------------

Primer pair 2, product size : 724
  Primer left                            Primer right
  start: 25211600                        start: 25212303
  end  : 25211621                        end  : 25212323
  tm   : 57                              tm   : 57
  seq  : TGAAATGGTCCATGTATTGCTC          seq  : TGTAATTATGGGCCTGTCTGG

  >117_primer_pair_2_product
  TGAAATGGTCCATGTATTGCTCAAAATAATTCTCATGAAAGTCAGGGTCTAGAACTCTGT
  CGGCTGAGGGCAGGTGCAGAGACTCAGGAGCTGGTTGGGATCATCAGGGATCTAGGCGGG
  TCAGGAGGAAGGGCAGCCAGTCTGTACTCACCTCTGGCCTGGAGGTTGCACAGTCCCAGT
  GACAGCAGCAGGACCAGGATCCAGGAGGGGACACCATGGTCCACAGGGTAACAAGGATGG
  AAGTTCATGCTTGATTCTGAGCCGGGCGCTGACTGTCATGTGATTTGGTCACATGACCGA
  CACAACGGGCGGGGCAGCATCACGTGATAGTCTGGCGGGGGCTGTCCTACTGTGGCTGGA
  TTCTAGTTGGAGGATCAGCCTACTCTTCTTCAGTTTCCCGGTTCCTCCAAATTTCTGGGC
  TCCTACTTGTTTCCACAGAGATGGATACTGTGGAGGTCCAGGAAGCAGAGAGATGGCTAA
  GGCTCATCAGGACCGTATGATCTCCCAAGTGTCCAGCTACTGAGTACCACAAGGTGATGG
  GTGGGAGGGTCCTCCCACGGAAGGATACCGCAGTCCCTAGGGGTTGCAAGCCCCACATGT
  TCCACTGGCTGCTAGAGCTACCTACTCAATCAGCCCTGGGCATCACCATCAGGTACTCGG
  CCAAAATGACCTCTCTGCTTCCAGTCCTCAGTTCTGGTCAGCACCAGACAGGCCCATAAT
  TACA
  ============================================================

Primers for ID: 118
Primer pair 1, product size : 722
  Primer left                            Primer right
  start: 25211600                        start: 25212301
  end  : 25211621                        end  : 25212321
  tm   : 57                              tm   : 57
  seq  : TGAAATGGTCCATGTATTGCTC          seq  : TAATTATGGGCCTGTCTGGTG

  >118_primer_pair_1_product
  TGAAATGGTCCATGTATTGCTCAAAATAATTCTCATGAAAGTCAGGGTCTAGAACTCTGT
  CGGCTGAGGGCAGGTGCAGAGACTCAGGAGCTGGTTGGGATCATCAGGGATCTAGGCGGG
  TCAGGAGGAAGGGCAGCCAGTCTGTACTCACCTCTGGCCTGGAGGTTGCACAGTCCCAGT
  GACAGCAGCAGGACCAGGATCCAGGAGGGGACACCATGGTCCACAGGGTAACAAGGATGG
  AAGTTCATGCTTGATTCTGAGCCGGGCGCTGACTGTCATGTGATTTGGTCACATGACCGA
  CACAACGGGCGGGGCAGCATCACGTGATAGTCTGGCGGGGGCTGTCCTACTGTGGCTGGA
  TTCTAGTTGGAGGATCAGCCTACTCTTCTTCAGTTTCCCGGTTCCTCCAAATTTCTGGGC
  TCCTACTTGTTTCCACAGAGATGGATACTGTGGAGGTCCAGGAAGCAGAGAGATGGCTAA
  GGCTCATCAGGACCGTATGATCTCCCAAGTGTCCAGCTACTGAGTACCACAAGGTGATGG
  GTGGGAGGGTCCTCCCACGGAAGGATACCGCAGTCCCTAGGGGTTGCAAGCCCCACATGT
  TCCACTGGCTGCTAGAGCTACCTACTCAATCAGCCCTGGGCATCACCATCAGGTACTCGG
  CCAAAATGACCTCTCTGCTTCCAGTCCTCAGTTCTGGTCAGCACCAGACAGGCCCATAAT
  TA
  --------------------

Primer pair 2, product size : 724
  Primer left                            Primer right
  start: 25211600                        start: 25212303
  end  : 25211621                        end  : 25212323
  tm   : 57                              tm   : 57
  seq  : TGAAATGGTCCATGTATTGCTC          seq  : TGTAATTATGGGCCTGTCTGG

  >118_primer_pair_2_product
  TGAAATGGTCCATGTATTGCTCAAAATAATTCTCATGAAAGTCAGGGTCTAGAACTCTGT
  CGGCTGAGGGCAGGTGCAGAGACTCAGGAGCTGGTTGGGATCATCAGGGATCTAGGCGGG
  TCAGGAGGAAGGGCAGCCAGTCTGTACTCACCTCTGGCCTGGAGGTTGCACAGTCCCAGT
  GACAGCAGCAGGACCAGGATCCAGGAGGGGACACCATGGTCCACAGGGTAACAAGGATGG
  AAGTTCATGCTTGATTCTGAGCCGGGCGCTGACTGTCATGTGATTTGGTCACATGACCGA
  CACAACGGGCGGGGCAGCATCACGTGATAGTCTGGCGGGGGCTGTCCTACTGTGGCTGGA
  TTCTAGTTGGAGGATCAGCCTACTCTTCTTCAGTTTCCCGGTTCCTCCAAATTTCTGGGC
  TCCTACTTGTTTCCACAGAGATGGATACTGTGGAGGTCCAGGAAGCAGAGAGATGGCTAA
  GGCTCATCAGGACCGTATGATCTCCCAAGTGTCCAGCTACTGAGTACCACAAGGTGATGG
  GTGGGAGGGTCCTCCCACGGAAGGATACCGCAGTCCCTAGGGGTTGCAAGCCCCACATGT
  TCCACTGGCTGCTAGAGCTACCTACTCAATCAGCCCTGGGCATCACCATCAGGTACTCGG
  CCAAAATGACCTCTCTGCTTCCAGTCCTCAGTTCTGGTCAGCACCAGACAGGCCCATAAT
  TACA
  ============================================================

Primers for ID: 119
Primer pair 1, product size : 719
  Primer left                            Primer right
  start: 25211842                        start: 25212540
  end  : 25211862                        end  : 25212560
  tm   : 57                              tm   : 57
  seq  : GTTCATGCTTGATTCTGAGCC           seq  : ATGAGACACTCTGTGCCAATG

  >119_primer_pair_1_product
  GTTCATGCTTGATTCTGAGCCGGGCGCTGACTGTCATGTGATTTGGTCACATGACCGACA
  CAACGGGCGGGGCAGCATCACGTGATAGTCTGGCGGGGGCTGTCCTACTGTGGCTGGATT
  CTAGTTGGAGGATCAGCCTACTCTTCTTCAGTTTCCCGGTTCCTCCAAATTTCTGGGCTC
  CTACTTGTTTCCACAGAGATGGATACTGTGGAGGTCCAGGAAGCAGAGAGATGGCTAAGG
  CTCATCAGGACCGTATGATCTCCCAAGTGTCCAGCTACTGAGTACCACAAGGTGATGGGT
  GGGAGGGTCCTCCCACGGAAGGATACCGCAGTCCCTAGGGGTTGCAAGCCCCACATGTTC
  CACTGGCTGCTAGAGCTACCTACTCAATCAGCCCTGGGCATCACCATCAGGTACTCGGCC
  AAAATGACCTCTCTGCTTCCAGTCCTCAGTTCTGGTCAGCACCAGACAGGCCCATAATTA
  CAGAGCCAGGGAAACTGGAACATTTGTCTCCCCTTAGACAGTGGCAGCAGGAAGGTGGGG
  GGTTGTTGCAGAGGAACAGTGTCTCTGAGAGAGGACCTTGGACTTTCTGGGAATCTCTGA
  GCTGCCCGGTTCTCCCCACTGCTGGCACTGTGCCCACAGCCCAAACAGAATGGGGGAGAT
  GGAGGGGCAGGGCTTCTGTGGGAAGCTGCCCTCCACCTCATTGGCACAGAGTGTCTCAT
  --------------------

Primer pair 2, product size : 726
  Primer left                            Primer right
  start: 25211842                        start: 25212546
  end  : 25211862                        end  : 25212567
  tm   : 57                              tm   : 61
  seq  : GTTCATGCTTGATTCTGAGCC           seq  : CTCTGCAATGAGACACTCTGTG

  >119_primer_pair_2_product
  GTTCATGCTTGATTCTGAGCCGGGCGCTGACTGTCATGTGATTTGGTCACATGACCGACA
  CAACGGGCGGGGCAGCATCACGTGATAGTCTGGCGGGGGCTGTCCTACTGTGGCTGGATT
  CTAGTTGGAGGATCAGCCTACTCTTCTTCAGTTTCCCGGTTCCTCCAAATTTCTGGGCTC
  CTACTTGTTTCCACAGAGATGGATACTGTGGAGGTCCAGGAAGCAGAGAGATGGCTAAGG
  CTCATCAGGACCGTATGATCTCCCAAGTGTCCAGCTACTGAGTACCACAAGGTGATGGGT
  GGGAGGGTCCTCCCACGGAAGGATACCGCAGTCCCTAGGGGTTGCAAGCCCCACATGTTC
  CACTGGCTGCTAGAGCTACCTACTCAATCAGCCCTGGGCATCACCATCAGGTACTCGGCC
  AAAATGACCTCTCTGCTTCCAGTCCTCAGTTCTGGTCAGCACCAGACAGGCCCATAATTA
  CAGAGCCAGGGAAACTGGAACATTTGTCTCCCCTTAGACAGTGGCAGCAGGAAGGTGGGG
  GGTTGTTGCAGAGGAACAGTGTCTCTGAGAGAGGACCTTGGACTTTCTGGGAATCTCTGA
  GCTGCCCGGTTCTCCCCACTGCTGGCACTGTGCCCACAGCCCAAACAGAATGGGGGAGAT
  GGAGGGGCAGGGCTTCTGTGGGAAGCTGCCCTCCACCTCATTGGCACAGAGTGTCTCATT
  GCAGAG
  ============================================================

Primers for ID: 120
Primer pair 1, product size : 719
  Primer left                            Primer right
  start: 25211842                        start: 25212540
  end  : 25211862                        end  : 25212560
  tm   : 57                              tm   : 57
  seq  : GTTCATGCTTGATTCTGAGCC           seq  : ATGAGACACTCTGTGCCAATG

  >120_primer_pair_1_product
  GTTCATGCTTGATTCTGAGCCGGGCGCTGACTGTCATGTGATTTGGTCACATGACCGACA
  CAACGGGCGGGGCAGCATCACGTGATAGTCTGGCGGGGGCTGTCCTACTGTGGCTGGATT
  CTAGTTGGAGGATCAGCCTACTCTTCTTCAGTTTCCCGGTTCCTCCAAATTTCTGGGCTC
  CTACTTGTTTCCACAGAGATGGATACTGTGGAGGTCCAGGAAGCAGAGAGATGGCTAAGG
  CTCATCAGGACCGTATGATCTCCCAAGTGTCCAGCTACTGAGTACCACAAGGTGATGGGT
  GGGAGGGTCCTCCCACGGAAGGATACCGCAGTCCCTAGGGGTTGCAAGCCCCACATGTTC
  CACTGGCTGCTAGAGCTACCTACTCAATCAGCCCTGGGCATCACCATCAGGTACTCGGCC
  AAAATGACCTCTCTGCTTCCAGTCCTCAGTTCTGGTCAGCACCAGACAGGCCCATAATTA
  CAGAGCCAGGGAAACTGGAACATTTGTCTCCCCTTAGACAGTGGCAGCAGGAAGGTGGGG
  GGTTGTTGCAGAGGAACAGTGTCTCTGAGAGAGGACCTTGGACTTTCTGGGAATCTCTGA
  GCTGCCCGGTTCTCCCCACTGCTGGCACTGTGCCCACAGCCCAAACAGAATGGGGGAGAT
  GGAGGGGCAGGGCTTCTGTGGGAAGCTGCCCTCCACCTCATTGGCACAGAGTGTCTCAT
  --------------------

Primer pair 2, product size : 831
  Primer left                            Primer right
  start: 25211842                        start: 25212653
  end  : 25211862                        end  : 25212672
  tm   : 57                              tm   : 55
  seq  : GTTCATGCTTGATTCTGAGCC           seq  : ATGGACATGTTCCACTCACG

  >120_primer_pair_2_product
  GTTCATGCTTGATTCTGAGCCGGGCGCTGACTGTCATGTGATTTGGTCACATGACCGACA
  CAACGGGCGGGGCAGCATCACGTGATAGTCTGGCGGGGGCTGTCCTACTGTGGCTGGATT
  CTAGTTGGAGGATCAGCCTACTCTTCTTCAGTTTCCCGGTTCCTCCAAATTTCTGGGCTC
  CTACTTGTTTCCACAGAGATGGATACTGTGGAGGTCCAGGAAGCAGAGAGATGGCTAAGG
  CTCATCAGGACCGTATGATCTCCCAAGTGTCCAGCTACTGAGTACCACAAGGTGATGGGT
  GGGAGGGTCCTCCCACGGAAGGATACCGCAGTCCCTAGGGGTTGCAAGCCCCACATGTTC
  CACTGGCTGCTAGAGCTACCTACTCAATCAGCCCTGGGCATCACCATCAGGTACTCGGCC
  AAAATGACCTCTCTGCTTCCAGTCCTCAGTTCTGGTCAGCACCAGACAGGCCCATAATTA
  CAGAGCCAGGGAAACTGGAACATTTGTCTCCCCTTAGACAGTGGCAGCAGGAAGGTGGGG
  GGTTGTTGCAGAGGAACAGTGTCTCTGAGAGAGGACCTTGGACTTTCTGGGAATCTCTGA
  GCTGCCCGGTTCTCCCCACTGCTGGCACTGTGCCCACAGCCCAAACAGAATGGGGGAGAT
  GGAGGGGCAGGGCTTCTGTGGGAAGCTGCCCTCCACCTCATTGGCACAGAGTGTCTCATT
  GCAGAGAGAAAAAAGGACCAGTTTTCTCTCTGGCACCCAGGTCTGGAAGAGGAGTGACAT
  CCACGGAAGTTGGTGACTTGGACTGGCTGGCCGTGAGTGGAACATGTCCAT
  ============================================================

Primers for ID: 121
Primer pair 1, product size : 670
  Primer left                            Primer right
  start: 25209909                        start: 25210558
  end  : 25209929                        end  : 25210578
  tm   : 57                              tm   : 57
  seq  : GATGGCATGAAAGCAGATAGC           seq  : ACAACCATGATCTGTGCTCTG

  >121_primer_pair_1_product
  GATGGCATGAAAGCAGATAGCTACAAGGCTCTTGGACCAGGCTAGCACTGGGTCCTGCAC
  CCAGGGAGAGCCACCTCACCTTGACAGGGTTGGCAGGAAGGGGCCCTAGAAAGTCAGTAG
  GATACGGGTAGTCCATCATGGCGAGCACAGTAAATGCATTTCGGGCAAACCCAAAGAGCT
  GAGTCAGGTCCTTTGGGCTGGAAAGTGATTGACAGGTACCAAAGTTCTGGCTGATGGTGT
  CATAGGCTGGGAAGAGAGAGGCCAGGAGAAAAGGCTGAGGAAACTGCTGGCAAATGTGAA
  GGGCAAGAATGAATGCCCAAGGTGGGCAGCAGGTGAGGAAAGAGTCCCTCACCTCCCTGG
  AGGAACAAGTCTTTGATTTGCTGAAAGGCATCCCGCACAGCCTGGGCGCACTTGGGACTC
  TGGCCATAAAAGTCCTGGAGAAGAGACCAAGGTTGCTGCTGCCATTCTTGCACTGGCCTG
  GGGTACCCAAGTCCCCTCACTCACCGCTGTGACATCTCGGAAGAATTGGTAGGAGTCCCC
  AAGGCCTGCAACAGCTACAACAGGAGCGCTGGCTGCCAGTGCCCCAGCCACCAGGTGGGG
  GTACTTCATCCTCATGTAGGCACTCAGCATCCCCCCATAACTGGGAGTACAGAGCACAGA
  TCATGGTTGT
  --------------------

Primer pair 2, product size : 656
  Primer left                            Primer right
  start: 25209909                        start: 25210543
  end  : 25209929                        end  : 25210564
  tm   : 57                              tm   : 61
  seq  : GATGGCATGAAAGCAGATAGC           seq  : TGCTCTGTACTCCCAGTTATGG

  >121_primer_pair_2_product
  GATGGCATGAAAGCAGATAGCTACAAGGCTCTTGGACCAGGCTAGCACTGGGTCCTGCAC
  CCAGGGAGAGCCACCTCACCTTGACAGGGTTGGCAGGAAGGGGCCCTAGAAAGTCAGTAG
  GATACGGGTAGTCCATCATGGCGAGCACAGTAAATGCATTTCGGGCAAACCCAAAGAGCT
  GAGTCAGGTCCTTTGGGCTGGAAAGTGATTGACAGGTACCAAAGTTCTGGCTGATGGTGT
  CATAGGCTGGGAAGAGAGAGGCCAGGAGAAAAGGCTGAGGAAACTGCTGGCAAATGTGAA
  GGGCAAGAATGAATGCCCAAGGTGGGCAGCAGGTGAGGAAAGAGTCCCTCACCTCCCTGG
  AGGAACAAGTCTTTGATTTGCTGAAAGGCATCCCGCACAGCCTGGGCGCACTTGGGACTC
  TGGCCATAAAAGTCCTGGAGAAGAGACCAAGGTTGCTGCTGCCATTCTTGCACTGGCCTG
  GGGTACCCAAGTCCCCTCACTCACCGCTGTGACATCTCGGAAGAATTGGTAGGAGTCCCC
  AAGGCCTGCAACAGCTACAACAGGAGCGCTGGCTGCCAGTGCCCCAGCCACCAGGTGGGG
  GTACTTCATCCTCATGTAGGCACTCAGCATCCCCCCATAACTGGGAGTACAGAGCA
  ============================================================

Primers for ID: 122
Primer pair 1, product size : 670
  Primer left                            Primer right
  start: 25209909                        start: 25210558
  end  : 25209929                        end  : 25210578
  tm   : 57                              tm   : 57
  seq  : GATGGCATGAAAGCAGATAGC           seq  : ACAACCATGATCTGTGCTCTG

  >122_primer_pair_1_product
  GATGGCATGAAAGCAGATAGCTACAAGGCTCTTGGACCAGGCTAGCACTGGGTCCTGCAC
  CCAGGGAGAGCCACCTCACCTTGACAGGGTTGGCAGGAAGGGGCCCTAGAAAGTCAGTAG
  GATACGGGTAGTCCATCATGGCGAGCACAGTAAATGCATTTCGGGCAAACCCAAAGAGCT
  GAGTCAGGTCCTTTGGGCTGGAAAGTGATTGACAGGTACCAAAGTTCTGGCTGATGGTGT
  CATAGGCTGGGAAGAGAGAGGCCAGGAGAAAAGGCTGAGGAAACTGCTGGCAAATGTGAA
  GGGCAAGAATGAATGCCCAAGGTGGGCAGCAGGTGAGGAAAGAGTCCCTCACCTCCCTGG
  AGGAACAAGTCTTTGATTTGCTGAAAGGCATCCCGCACAGCCTGGGCGCACTTGGGACTC
  TGGCCATAAAAGTCCTGGAGAAGAGACCAAGGTTGCTGCTGCCATTCTTGCACTGGCCTG
  GGGTACCCAAGTCCCCTCACTCACCGCTGTGACATCTCGGAAGAATTGGTAGGAGTCCCC
  AAGGCCTGCAACAGCTACAACAGGAGCGCTGGCTGCCAGTGCCCCAGCCACCAGGTGGGG
  GTACTTCATCCTCATGTAGGCACTCAGCATCCCCCCATAACTGGGAGTACAGAGCACAGA
  TCATGGTTGT
  --------------------

Primer pair 2, product size : 782
  Primer left                            Primer right
  start: 25209909                        start: 25210670
  end  : 25209929                        end  : 25210690
  tm   : 57                              tm   : 57
  seq  : GATGGCATGAAAGCAGATAGC           seq  : AGTTTCAGGTCCTTCTTTCCC

  >122_primer_pair_2_product
  GATGGCATGAAAGCAGATAGCTACAAGGCTCTTGGACCAGGCTAGCACTGGGTCCTGCAC
  CCAGGGAGAGCCACCTCACCTTGACAGGGTTGGCAGGAAGGGGCCCTAGAAAGTCAGTAG
  GATACGGGTAGTCCATCATGGCGAGCACAGTAAATGCATTTCGGGCAAACCCAAAGAGCT
  GAGTCAGGTCCTTTGGGCTGGAAAGTGATTGACAGGTACCAAAGTTCTGGCTGATGGTGT
  CATAGGCTGGGAAGAGAGAGGCCAGGAGAAAAGGCTGAGGAAACTGCTGGCAAATGTGAA
  GGGCAAGAATGAATGCCCAAGGTGGGCAGCAGGTGAGGAAAGAGTCCCTCACCTCCCTGG
  AGGAACAAGTCTTTGATTTGCTGAAAGGCATCCCGCACAGCCTGGGCGCACTTGGGACTC
  TGGCCATAAAAGTCCTGGAGAAGAGACCAAGGTTGCTGCTGCCATTCTTGCACTGGCCTG
  GGGTACCCAAGTCCCCTCACTCACCGCTGTGACATCTCGGAAGAATTGGTAGGAGTCCCC
  AAGGCCTGCAACAGCTACAACAGGAGCGCTGGCTGCCAGTGCCCCAGCCACCAGGTGGGG
  GTACTTCATCCTCATGTAGGCACTCAGCATCCCCCCATAACTGGGAGTACAGAGCACAGA
  TCATGGTTGTGGGAAGCTGCCCACAACTCAGGCGAGCAGCCTCACTGTCCTCCAGGCTGA
  GGTGCTAGGCTGCTCTTTCCCTGCTCAGAACGCCCAAGGGTGGGAAAGAAGGACCTGAAA
  CT
  ============================================================

Primers for ID: 123
Primer pair 1, product size : 692
  Primer left                            Primer right
  start: 25210129                        start: 25210799
  end  : 25210149                        end  : 25210820
  tm   : 57                              tm   : 59
  seq  : AAAGTTCTGGCTGATGGTGTC           seq  : GGGACAATTCCTTCTGACTTTG

  >123_primer_pair_1_product
  AAAGTTCTGGCTGATGGTGTCATAGGCTGGGAAGAGAGAGGCCAGGAGAAAAGGCTGAGG
  AAACTGCTGGCAAATGTGAAGGGCAAGAATGAATGCCCAAGGTGGGCAGCAGGTGAGGAA
  AGAGTCCCTCACCTCCCTGGAGGAACAAGTCTTTGATTTGCTGAAAGGCATCCCGCACAG
  CCTGGGCGCACTTGGGACTCTGGCCATAAAAGTCCTGGAGAAGAGACCAAGGTTGCTGCT
  GCCATTCTTGCACTGGCCTGGGGTACCCAAGTCCCCTCACTCACCGCTGTGACATCTCGG
  AAGAATTGGTAGGAGTCCCCAAGGCCTGCAACAGCTACAACAGGAGCGCTGGCTGCCAGT
  GCCCCAGCCACCAGGTGGGGGTACTTCATCCTCATGTAGGCACTCAGCATCCCCCCATAA
  CTGGGAGTACAGAGCACAGATCATGGTTGTGGGAAGCTGCCCACAACTCAGGCGAGCAGC
  CTCACTGTCCTCCAGGCTGAGGTGCTAGGCTGCTCTTTCCCTGCTCAGAACGCCCAAGGG
  TGGGAAAGAAGGACCTGAAACTGTCAGGCCCACACACCCTGATCCCAGGGCCAAGGCAGA
  TACAGCCTTCACTGGGAGAAGGCACCTGTGGGTGCCCTGCCCTGACCCAGCAATGAAGAC
  ATTGCAGAGACAAAGTCAGAAGGAATTGTCCC
  --------------------

Primer pair 2, product size : 661
  Primer left                            Primer right
  start: 25210129                        start: 25210770
  end  : 25210149                        end  : 25210789
  tm   : 57                              tm   : 55
  seq  : AAAGTTCTGGCTGATGGTGTC           seq  : TGTCTTCATTGCTGGGTCAG

  >123_primer_pair_2_product
  AAAGTTCTGGCTGATGGTGTCATAGGCTGGGAAGAGAGAGGCCAGGAGAAAAGGCTGAGG
  AAACTGCTGGCAAATGTGAAGGGCAAGAATGAATGCCCAAGGTGGGCAGCAGGTGAGGAA
  AGAGTCCCTCACCTCCCTGGAGGAACAAGTCTTTGATTTGCTGAAAGGCATCCCGCACAG
  CCTGGGCGCACTTGGGACTCTGGCCATAAAAGTCCTGGAGAAGAGACCAAGGTTGCTGCT
  GCCATTCTTGCACTGGCCTGGGGTACCCAAGTCCCCTCACTCACCGCTGTGACATCTCGG
  AAGAATTGGTAGGAGTCCCCAAGGCCTGCAACAGCTACAACAGGAGCGCTGGCTGCCAGT
  GCCCCAGCCACCAGGTGGGGGTACTTCATCCTCATGTAGGCACTCAGCATCCCCCCATAA
  CTGGGAGTACAGAGCACAGATCATGGTTGTGGGAAGCTGCCCACAACTCAGGCGAGCAGC
  CTCACTGTCCTCCAGGCTGAGGTGCTAGGCTGCTCTTTCCCTGCTCAGAACGCCCAAGGG
  TGGGAAAGAAGGACCTGAAACTGTCAGGCCCACACACCCTGATCCCAGGGCCAAGGCAGA
  TACAGCCTTCACTGGGAGAAGGCACCTGTGGGTGCCCTGCCCTGACCCAGCAATGAAGAC
  A
  ============================================================

Primers for ID: 124
Primer pair 1, product size : 692
  Primer left                            Primer right
  start: 25210129                        start: 25210799
  end  : 25210149                        end  : 25210820
  tm   : 57                              tm   : 59
  seq  : AAAGTTCTGGCTGATGGTGTC           seq  : GGGACAATTCCTTCTGACTTTG

  >124_primer_pair_1_product
  AAAGTTCTGGCTGATGGTGTCATAGGCTGGGAAGAGAGAGGCCAGGAGAAAAGGCTGAGG
  AAACTGCTGGCAAATGTGAAGGGCAAGAATGAATGCCCAAGGTGGGCAGCAGGTGAGGAA
  AGAGTCCCTCACCTCCCTGGAGGAACAAGTCTTTGATTTGCTGAAAGGCATCCCGCACAG
  CCTGGGCGCACTTGGGACTCTGGCCATAAAAGTCCTGGAGAAGAGACCAAGGTTGCTGCT
  GCCATTCTTGCACTGGCCTGGGGTACCCAAGTCCCCTCACTCACCGCTGTGACATCTCGG
  AAGAATTGGTAGGAGTCCCCAAGGCCTGCAACAGCTACAACAGGAGCGCTGGCTGCCAGT
  GCCCCAGCCACCAGGTGGGGGTACTTCATCCTCATGTAGGCACTCAGCATCCCCCCATAA
  CTGGGAGTACAGAGCACAGATCATGGTTGTGGGAAGCTGCCCACAACTCAGGCGAGCAGC
  CTCACTGTCCTCCAGGCTGAGGTGCTAGGCTGCTCTTTCCCTGCTCAGAACGCCCAAGGG
  TGGGAAAGAAGGACCTGAAACTGTCAGGCCCACACACCCTGATCCCAGGGCCAAGGCAGA
  TACAGCCTTCACTGGGAGAAGGCACCTGTGGGTGCCCTGCCCTGACCCAGCAATGAAGAC
  ATTGCAGAGACAAAGTCAGAAGGAATTGTCCC
  --------------------

Primer pair 2, product size : 661
  Primer left                            Primer right
  start: 25210129                        start: 25210770
  end  : 25210149                        end  : 25210789
  tm   : 57                              tm   : 55
  seq  : AAAGTTCTGGCTGATGGTGTC           seq  : TGTCTTCATTGCTGGGTCAG

  >124_primer_pair_2_product
  AAAGTTCTGGCTGATGGTGTCATAGGCTGGGAAGAGAGAGGCCAGGAGAAAAGGCTGAGG
  AAACTGCTGGCAAATGTGAAGGGCAAGAATGAATGCCCAAGGTGGGCAGCAGGTGAGGAA
  AGAGTCCCTCACCTCCCTGGAGGAACAAGTCTTTGATTTGCTGAAAGGCATCCCGCACAG
  CCTGGGCGCACTTGGGACTCTGGCCATAAAAGTCCTGGAGAAGAGACCAAGGTTGCTGCT
  GCCATTCTTGCACTGGCCTGGGGTACCCAAGTCCCCTCACTCACCGCTGTGACATCTCGG
  AAGAATTGGTAGGAGTCCCCAAGGCCTGCAACAGCTACAACAGGAGCGCTGGCTGCCAGT
  GCCCCAGCCACCAGGTGGGGGTACTTCATCCTCATGTAGGCACTCAGCATCCCCCCATAA
  CTGGGAGTACAGAGCACAGATCATGGTTGTGGGAAGCTGCCCACAACTCAGGCGAGCAGC
  CTCACTGTCCTCCAGGCTGAGGTGCTAGGCTGCTCTTTCCCTGCTCAGAACGCCCAAGGG
  TGGGAAAGAAGGACCTGAAACTGTCAGGCCCACACACCCTGATCCCAGGGCCAAGGCAGA
  TACAGCCTTCACTGGGAGAAGGCACCTGTGGGTGCCCTGCCCTGACCCAGCAATGAAGAC
  A
  ============================================================

Primers for ID: 125
Primer pair 1, product size : 692
  Primer left                            Primer right
  start: 25210129                        start: 25210799
  end  : 25210149                        end  : 25210820
  tm   : 57                              tm   : 59
  seq  : AAAGTTCTGGCTGATGGTGTC           seq  : GGGACAATTCCTTCTGACTTTG

  >125_primer_pair_1_product
  AAAGTTCTGGCTGATGGTGTCATAGGCTGGGAAGAGAGAGGCCAGGAGAAAAGGCTGAGG
  AAACTGCTGGCAAATGTGAAGGGCAAGAATGAATGCCCAAGGTGGGCAGCAGGTGAGGAA
  AGAGTCCCTCACCTCCCTGGAGGAACAAGTCTTTGATTTGCTGAAAGGCATCCCGCACAG
  CCTGGGCGCACTTGGGACTCTGGCCATAAAAGTCCTGGAGAAGAGACCAAGGTTGCTGCT
  GCCATTCTTGCACTGGCCTGGGGTACCCAAGTCCCCTCACTCACCGCTGTGACATCTCGG
  AAGAATTGGTAGGAGTCCCCAAGGCCTGCAACAGCTACAACAGGAGCGCTGGCTGCCAGT
  GCCCCAGCCACCAGGTGGGGGTACTTCATCCTCATGTAGGCACTCAGCATCCCCCCATAA
  CTGGGAGTACAGAGCACAGATCATGGTTGTGGGAAGCTGCCCACAACTCAGGCGAGCAGC
  CTCACTGTCCTCCAGGCTGAGGTGCTAGGCTGCTCTTTCCCTGCTCAGAACGCCCAAGGG
  TGGGAAAGAAGGACCTGAAACTGTCAGGCCCACACACCCTGATCCCAGGGCCAAGGCAGA
  TACAGCCTTCACTGGGAGAAGGCACCTGTGGGTGCCCTGCCCTGACCCAGCAATGAAGAC
  ATTGCAGAGACAAAGTCAGAAGGAATTGTCCC
  --------------------

Primer pair 2, product size : 661
  Primer left                            Primer right
  start: 25210129                        start: 25210770
  end  : 25210149                        end  : 25210789
  tm   : 57                              tm   : 55
  seq  : AAAGTTCTGGCTGATGGTGTC           seq  : TGTCTTCATTGCTGGGTCAG

  >125_primer_pair_2_product
  AAAGTTCTGGCTGATGGTGTCATAGGCTGGGAAGAGAGAGGCCAGGAGAAAAGGCTGAGG
  AAACTGCTGGCAAATGTGAAGGGCAAGAATGAATGCCCAAGGTGGGCAGCAGGTGAGGAA
  AGAGTCCCTCACCTCCCTGGAGGAACAAGTCTTTGATTTGCTGAAAGGCATCCCGCACAG
  CCTGGGCGCACTTGGGACTCTGGCCATAAAAGTCCTGGAGAAGAGACCAAGGTTGCTGCT
  GCCATTCTTGCACTGGCCTGGGGTACCCAAGTCCCCTCACTCACCGCTGTGACATCTCGG
  AAGAATTGGTAGGAGTCCCCAAGGCCTGCAACAGCTACAACAGGAGCGCTGGCTGCCAGT
  GCCCCAGCCACCAGGTGGGGGTACTTCATCCTCATGTAGGCACTCAGCATCCCCCCATAA
  CTGGGAGTACAGAGCACAGATCATGGTTGTGGGAAGCTGCCCACAACTCAGGCGAGCAGC
  CTCACTGTCCTCCAGGCTGAGGTGCTAGGCTGCTCTTTCCCTGCTCAGAACGCCCAAGGG
  TGGGAAAGAAGGACCTGAAACTGTCAGGCCCACACACCCTGATCCCAGGGCCAAGGCAGA
  TACAGCCTTCACTGGGAGAAGGCACCTGTGGGTGCCCTGCCCTGACCCAGCAATGAAGAC
  A
  ============================================================

Primers for ID: 126
Primer pair 1, product size : 775
  Primer left                            Primer right
  start: 25210277                        start: 25211030
  end  : 25210297                        end  : 25211051
  tm   : 55                              tm   : 61
  seq  : GTCTTTGATTTGCTGAAAGGC           seq  : AGATCTGGACCCATGAGAGAAC

  >126_primer_pair_1_product
  GTCTTTGATTTGCTGAAAGGCATCCCGCACAGCCTGGGCGCACTTGGGACTCTGGCCATA
  AAAGTCCTGGAGAAGAGACCAAGGTTGCTGCTGCCATTCTTGCACTGGCCTGGGGTACCC
  AAGTCCCCTCACTCACCGCTGTGACATCTCGGAAGAATTGGTAGGAGTCCCCAAGGCCTG
  CAACAGCTACAACAGGAGCGCTGGCTGCCAGTGCCCCAGCCACCAGGTGGGGGTACTTCA
  TCCTCATGTAGGCACTCAGCATCCCCCCATAACTGGGAGTACAGAGCACAGATCATGGTT
  GTGGGAAGCTGCCCACAACTCAGGCGAGCAGCCTCACTGTCCTCCAGGCTGAGGTGCTAG
  GCTGCTCTTTCCCTGCTCAGAACGCCCAAGGGTGGGAAAGAAGGACCTGAAACTGTCAGG
  CCCACACACCCTGATCCCAGGGCCAAGGCAGATACAGCCTTCACTGGGAGAAGGCACCTG
  TGGGTGCCCTGCCCTGACCCAGCAATGAAGACATTGCAGAGACAAAGTCAGAAGGAATTG
  TCCCACTAGTGGGAACAACATAGCATACACTGCCTATGAGGTCCACTCAAGGAGGGCTTC
  CAGAAGGAGGTAAAGCTAGACCCCGCCCTTCCACATGTGGGGTAGGCATAGGATGTTGAG
  ACTGTAAGAGACATCTCTTTGGCCCTCCTTGTATAGGGTGTCAATCGGCACAACAGGGTG
  GAGCCTTAGAGTAGGGTAAGATTAGGACTCTAGGTTCTCTCATGGGTCCAGATCT
  --------------------

Primer pair 2, product size : 839
  Primer left                            Primer right
  start: 25210213                        start: 25211030
  end  : 25210232                        end  : 25211051
  tm   : 53                              tm   : 61
  seq  : AAGAATGAATGCCCAAGGTG            seq  : AGATCTGGACCCATGAGAGAAC

  >126_primer_pair_2_product
  AAGAATGAATGCCCAAGGTGGGCAGCAGGTGAGGAAAGAGTCCCTCACCTCCCTGGAGGA
  ACAAGTCTTTGATTTGCTGAAAGGCATCCCGCACAGCCTGGGCGCACTTGGGACTCTGGC
  CATAAAAGTCCTGGAGAAGAGACCAAGGTTGCTGCTGCCATTCTTGCACTGGCCTGGGGT
  ACCCAAGTCCCCTCACTCACCGCTGTGACATCTCGGAAGAATTGGTAGGAGTCCCCAAGG
  CCTGCAACAGCTACAACAGGAGCGCTGGCTGCCAGTGCCCCAGCCACCAGGTGGGGGTAC
  TTCATCCTCATGTAGGCACTCAGCATCCCCCCATAACTGGGAGTACAGAGCACAGATCAT
  GGTTGTGGGAAGCTGCCCACAACTCAGGCGAGCAGCCTCACTGTCCTCCAGGCTGAGGTG
  CTAGGCTGCTCTTTCCCTGCTCAGAACGCCCAAGGGTGGGAAAGAAGGACCTGAAACTGT
  CAGGCCCACACACCCTGATCCCAGGGCCAAGGCAGATACAGCCTTCACTGGGAGAAGGCA
  CCTGTGGGTGCCCTGCCCTGACCCAGCAATGAAGACATTGCAGAGACAAAGTCAGAAGGA
  ATTGTCCCACTAGTGGGAACAACATAGCATACACTGCCTATGAGGTCCACTCAAGGAGGG
  CTTCCAGAAGGAGGTAAAGCTAGACCCCGCCCTTCCACATGTGGGGTAGGCATAGGATGT
  TGAGACTGTAAGAGACATCTCTTTGGCCCTCCTTGTATAGGGTGTCAATCGGCACAACAG
  GGTGGAGCCTTAGAGTAGGGTAAGATTAGGACTCTAGGTTCTCTCATGGGTCCAGATCT
  ============================================================

Primers for ID: 136
Primer pair 1, product size : 715
  Primer left                            Primer right
  start: 25211383                        start: 25212076
  end  : 25211403                        end  : 25212097
  tm   : 57                              tm   : 61
  seq  : ATGAAGCCAGAGTTGTTAGCG           seq  : ATACGGTCCTGATGAGCCTTAG

  >136_primer_pair_1_product
  ATGAAGCCAGAGTTGTTAGCGAAGGACCAGATATCCCCCTCATTCCCTGTGTAGAAAAAG
  ATGGGCCCTTCGCCCATCTTCCAGAACTTATCTGTTGGAAGTAAATGAGTTTCCATAAGG
  CCAGGGAAACGCAGGTAGGAACCCATGCGGTCGAGCCAGCACTCACCTGACACTAGGAAC
  CGCTGGCCAAAGGTTTTGTTGCCGAAACTCTCAAAGTTGAAATGGTCCATGTATTGCTCA
  AAATAATTCTCATGAAAGTCAGGGTCTAGAACTCTGTCGGCTGAGGGCAGGTGCAGAGAC
  TCAGGAGCTGGTTGGGATCATCAGGGATCTAGGCGGGTCAGGAGGAAGGGCAGCCAGTCT
  GTACTCACCTCTGGCCTGGAGGTTGCACAGTCCCAGTGACAGCAGCAGGACCAGGATCCA
  GGAGGGGACACCATGGTCCACAGGGTAACAAGGATGGAAGTTCATGCTTGATTCTGAGCC
  GGGCGCTGACTGTCATGTGATTTGGTCACATGACCGACACAACGGGCGGGGCAGCATCAC
  GTGATAGTCTGGCGGGGGCTGTCCTACTGTGGCTGGATTCTAGTTGGAGGATCAGCCTAC
  TCTTCTTCAGTTTCCCGGTTCCTCCAAATTTCTGGGCTCCTACTTGTTTCCACAGAGATG
  GATACTGTGGAGGTCCAGGAAGCAGAGAGATGGCTAAGGCTCATCAGGACCGTAT
  --------------------

Primer pair 2, product size : 671
  Primer left                            Primer right
  start: 25211332                        start: 25211982
  end  : 25211351                        end  : 25212002
  tm   : 55                              tm   : 57
  seq  : ACGTGCTCAGCAAAGACAAG            seq  : AACCGGGAAACTGAAGAAGAG

  >136_primer_pair_2_product
  ACGTGCTCAGCAAAGACAAGCAGGGCCTCCTGCTGGGCTGCCAGTTCCACCATGAAGCCA
  GAGTTGTTAGCGAAGGACCAGATATCCCCCTCATTCCCTGTGTAGAAAAAGATGGGCCCT
  TCGCCCATCTTCCAGAACTTATCTGTTGGAAGTAAATGAGTTTCCATAAGGCCAGGGAAA
  CGCAGGTAGGAACCCATGCGGTCGAGCCAGCACTCACCTGACACTAGGAACCGCTGGCCA
  AAGGTTTTGTTGCCGAAACTCTCAAAGTTGAAATGGTCCATGTATTGCTCAAAATAATTC
  TCATGAAAGTCAGGGTCTAGAACTCTGTCGGCTGAGGGCAGGTGCAGAGACTCAGGAGCT
  GGTTGGGATCATCAGGGATCTAGGCGGGTCAGGAGGAAGGGCAGCCAGTCTGTACTCACC
  TCTGGCCTGGAGGTTGCACAGTCCCAGTGACAGCAGCAGGACCAGGATCCAGGAGGGGAC
  ACCATGGTCCACAGGGTAACAAGGATGGAAGTTCATGCTTGATTCTGAGCCGGGCGCTGA
  CTGTCATGTGATTTGGTCACATGACCGACACAACGGGCGGGGCAGCATCACGTGATAGTC
  TGGCGGGGGCTGTCCTACTGTGGCTGGATTCTAGTTGGAGGATCAGCCTACTCTTCTTCA
  GTTTCCCGGTT
  ============================================================

Primers for ID: 137
Primer pair 1, product size : 715
  Primer left                            Primer right
  start: 25211383                        start: 25212076
  end  : 25211403                        end  : 25212097
  tm   : 57                              tm   : 61
  seq  : ATGAAGCCAGAGTTGTTAGCG           seq  : ATACGGTCCTGATGAGCCTTAG

  >137_primer_pair_1_product
  ATGAAGCCAGAGTTGTTAGCGAAGGACCAGATATCCCCCTCATTCCCTGTGTAGAAAAAG
  ATGGGCCCTTCGCCCATCTTCCAGAACTTATCTGTTGGAAGTAAATGAGTTTCCATAAGG
  CCAGGGAAACGCAGGTAGGAACCCATGCGGTCGAGCCAGCACTCACCTGACACTAGGAAC
  CGCTGGCCAAAGGTTTTGTTGCCGAAACTCTCAAAGTTGAAATGGTCCATGTATTGCTCA
  AAATAATTCTCATGAAAGTCAGGGTCTAGAACTCTGTCGGCTGAGGGCAGGTGCAGAGAC
  TCAGGAGCTGGTTGGGATCATCAGGGATCTAGGCGGGTCAGGAGGAAGGGCAGCCAGTCT
  GTACTCACCTCTGGCCTGGAGGTTGCACAGTCCCAGTGACAGCAGCAGGACCAGGATCCA
  GGAGGGGACACCATGGTCCACAGGGTAACAAGGATGGAAGTTCATGCTTGATTCTGAGCC
  GGGCGCTGACTGTCATGTGATTTGGTCACATGACCGACACAACGGGCGGGGCAGCATCAC
  GTGATAGTCTGGCGGGGGCTGTCCTACTGTGGCTGGATTCTAGTTGGAGGATCAGCCTAC
  TCTTCTTCAGTTTCCCGGTTCCTCCAAATTTCTGGGCTCCTACTTGTTTCCACAGAGATG
  GATACTGTGGAGGTCCAGGAAGCAGAGAGATGGCTAAGGCTCATCAGGACCGTAT
  --------------------

Primer pair 2, product size : 671
  Primer left                            Primer right
  start: 25211332                        start: 25211982
  end  : 25211351                        end  : 25212002
  tm   : 55                              tm   : 57
  seq  : ACGTGCTCAGCAAAGACAAG            seq  : AACCGGGAAACTGAAGAAGAG

  >137_primer_pair_2_product
  ACGTGCTCAGCAAAGACAAGCAGGGCCTCCTGCTGGGCTGCCAGTTCCACCATGAAGCCA
  GAGTTGTTAGCGAAGGACCAGATATCCCCCTCATTCCCTGTGTAGAAAAAGATGGGCCCT
  TCGCCCATCTTCCAGAACTTATCTGTTGGAAGTAAATGAGTTTCCATAAGGCCAGGGAAA
  CGCAGGTAGGAACCCATGCGGTCGAGCCAGCACTCACCTGACACTAGGAACCGCTGGCCA
  AAGGTTTTGTTGCCGAAACTCTCAAAGTTGAAATGGTCCATGTATTGCTCAAAATAATTC
  TCATGAAAGTCAGGGTCTAGAACTCTGTCGGCTGAGGGCAGGTGCAGAGACTCAGGAGCT
  GGTTGGGATCATCAGGGATCTAGGCGGGTCAGGAGGAAGGGCAGCCAGTCTGTACTCACC
  TCTGGCCTGGAGGTTGCACAGTCCCAGTGACAGCAGCAGGACCAGGATCCAGGAGGGGAC
  ACCATGGTCCACAGGGTAACAAGGATGGAAGTTCATGCTTGATTCTGAGCCGGGCGCTGA
  CTGTCATGTGATTTGGTCACATGACCGACACAACGGGCGGGGCAGCATCACGTGATAGTC
  TGGCGGGGGCTGTCCTACTGTGGCTGGATTCTAGTTGGAGGATCAGCCTACTCTTCTTCA
  GTTTCCCGGTT
  ============================================================

Primers for ID: 138
Primer pair 1, product size : 898
  Primer left                            Primer right
  start: 25211383                        start: 25212260
  end  : 25211403                        end  : 25212280
  tm   : 57                              tm   : 57
  seq  : ATGAAGCCAGAGTTGTTAGCG           seq  : GAAGCAGAGAGGTCATTTTGG

  >138_primer_pair_1_product
  ATGAAGCCAGAGTTGTTAGCGAAGGACCAGATATCCCCCTCATTCCCTGTGTAGAAAAAG
  ATGGGCCCTTCGCCCATCTTCCAGAACTTATCTGTTGGAAGTAAATGAGTTTCCATAAGG
  CCAGGGAAACGCAGGTAGGAACCCATGCGGTCGAGCCAGCACTCACCTGACACTAGGAAC
  CGCTGGCCAAAGGTTTTGTTGCCGAAACTCTCAAAGTTGAAATGGTCCATGTATTGCTCA
  AAATAATTCTCATGAAAGTCAGGGTCTAGAACTCTGTCGGCTGAGGGCAGGTGCAGAGAC
  TCAGGAGCTGGTTGGGATCATCAGGGATCTAGGCGGGTCAGGAGGAAGGGCAGCCAGTCT
  GTACTCACCTCTGGCCTGGAGGTTGCACAGTCCCAGTGACAGCAGCAGGACCAGGATCCA
  GGAGGGGACACCATGGTCCACAGGGTAACAAGGATGGAAGTTCATGCTTGATTCTGAGCC
  GGGCGCTGACTGTCATGTGATTTGGTCACATGACCGACACAACGGGCGGGGCAGCATCAC
  GTGATAGTCTGGCGGGGGCTGTCCTACTGTGGCTGGATTCTAGTTGGAGGATCAGCCTAC
  TCTTCTTCAGTTTCCCGGTTCCTCCAAATTTCTGGGCTCCTACTTGTTTCCACAGAGATG
  GATACTGTGGAGGTCCAGGAAGCAGAGAGATGGCTAAGGCTCATCAGGACCGTATGATCT
  CCCAAGTGTCCAGCTACTGAGTACCACAAGGTGATGGGTGGGAGGGTCCTCCCACGGAAG
  GATACCGCAGTCCCTAGGGGTTGCAAGCCCCACATGTTCCACTGGCTGCTAGAGCTACCT
  ACTCAATCAGCCCTGGGCATCACCATCAGGTACTCGGCCAAAATGACCTCTCTGCTTC
  --------------------

Primer pair 2, product size : 899
  Primer left                            Primer right
  start: 25211383                        start: 25212261
  end  : 25211403                        end  : 25212281
  tm   : 57                              tm   : 57
  seq  : ATGAAGCCAGAGTTGTTAGCG           seq  : GGAAGCAGAGAGGTCATTTTG

  >138_primer_pair_2_product
  ATGAAGCCAGAGTTGTTAGCGAAGGACCAGATATCCCCCTCATTCCCTGTGTAGAAAAAG
  ATGGGCCCTTCGCCCATCTTCCAGAACTTATCTGTTGGAAGTAAATGAGTTTCCATAAGG
  CCAGGGAAACGCAGGTAGGAACCCATGCGGTCGAGCCAGCACTCACCTGACACTAGGAAC
  CGCTGGCCAAAGGTTTTGTTGCCGAAACTCTCAAAGTTGAAATGGTCCATGTATTGCTCA
  AAATAATTCTCATGAAAGTCAGGGTCTAGAACTCTGTCGGCTGAGGGCAGGTGCAGAGAC
  TCAGGAGCTGGTTGGGATCATCAGGGATCTAGGCGGGTCAGGAGGAAGGGCAGCCAGTCT
  GTACTCACCTCTGGCCTGGAGGTTGCACAGTCCCAGTGACAGCAGCAGGACCAGGATCCA
  GGAGGGGACACCATGGTCCACAGGGTAACAAGGATGGAAGTTCATGCTTGATTCTGAGCC
  GGGCGCTGACTGTCATGTGATTTGGTCACATGACCGACACAACGGGCGGGGCAGCATCAC
  GTGATAGTCTGGCGGGGGCTGTCCTACTGTGGCTGGATTCTAGTTGGAGGATCAGCCTAC
  TCTTCTTCAGTTTCCCGGTTCCTCCAAATTTCTGGGCTCCTACTTGTTTCCACAGAGATG
  GATACTGTGGAGGTCCAGGAAGCAGAGAGATGGCTAAGGCTCATCAGGACCGTATGATCT
  CCCAAGTGTCCAGCTACTGAGTACCACAAGGTGATGGGTGGGAGGGTCCTCCCACGGAAG
  GATACCGCAGTCCCTAGGGGTTGCAAGCCCCACATGTTCCACTGGCTGCTAGAGCTACCT
  ACTCAATCAGCCCTGGGCATCACCATCAGGTACTCGGCCAAAATGACCTCTCTGCTTCC
  ============================================================

Primers for ID: 139
Primer pair 1, product size : 722
  Primer left                            Primer right
  start: 25211600                        start: 25212301
  end  : 25211621                        end  : 25212321
  tm   : 57                              tm   : 57
  seq  : TGAAATGGTCCATGTATTGCTC          seq  : TAATTATGGGCCTGTCTGGTG

  >139_primer_pair_1_product
  TGAAATGGTCCATGTATTGCTCAAAATAATTCTCATGAAAGTCAGGGTCTAGAACTCTGT
  CGGCTGAGGGCAGGTGCAGAGACTCAGGAGCTGGTTGGGATCATCAGGGATCTAGGCGGG
  TCAGGAGGAAGGGCAGCCAGTCTGTACTCACCTCTGGCCTGGAGGTTGCACAGTCCCAGT
  GACAGCAGCAGGACCAGGATCCAGGAGGGGACACCATGGTCCACAGGGTAACAAGGATGG
  AAGTTCATGCTTGATTCTGAGCCGGGCGCTGACTGTCATGTGATTTGGTCACATGACCGA
  CACAACGGGCGGGGCAGCATCACGTGATAGTCTGGCGGGGGCTGTCCTACTGTGGCTGGA
  TTCTAGTTGGAGGATCAGCCTACTCTTCTTCAGTTTCCCGGTTCCTCCAAATTTCTGGGC
  TCCTACTTGTTTCCACAGAGATGGATACTGTGGAGGTCCAGGAAGCAGAGAGATGGCTAA
  GGCTCATCAGGACCGTATGATCTCCCAAGTGTCCAGCTACTGAGTACCACAAGGTGATGG
  GTGGGAGGGTCCTCCCACGGAAGGATACCGCAGTCCCTAGGGGTTGCAAGCCCCACATGT
  TCCACTGGCTGCTAGAGCTACCTACTCAATCAGCCCTGGGCATCACCATCAGGTACTCGG
  CCAAAATGACCTCTCTGCTTCCAGTCCTCAGTTCTGGTCAGCACCAGACAGGCCCATAAT
  TA
  --------------------

Primer pair 2, product size : 724
  Primer left                            Primer right
  start: 25211600                        start: 25212303
  end  : 25211621                        end  : 25212323
  tm   : 57                              tm   : 57
  seq  : TGAAATGGTCCATGTATTGCTC          seq  : TGTAATTATGGGCCTGTCTGG

  >139_primer_pair_2_product
  TGAAATGGTCCATGTATTGCTCAAAATAATTCTCATGAAAGTCAGGGTCTAGAACTCTGT
  CGGCTGAGGGCAGGTGCAGAGACTCAGGAGCTGGTTGGGATCATCAGGGATCTAGGCGGG
  TCAGGAGGAAGGGCAGCCAGTCTGTACTCACCTCTGGCCTGGAGGTTGCACAGTCCCAGT
  GACAGCAGCAGGACCAGGATCCAGGAGGGGACACCATGGTCCACAGGGTAACAAGGATGG
  AAGTTCATGCTTGATTCTGAGCCGGGCGCTGACTGTCATGTGATTTGGTCACATGACCGA
  CACAACGGGCGGGGCAGCATCACGTGATAGTCTGGCGGGGGCTGTCCTACTGTGGCTGGA
  TTCTAGTTGGAGGATCAGCCTACTCTTCTTCAGTTTCCCGGTTCCTCCAAATTTCTGGGC
  TCCTACTTGTTTCCACAGAGATGGATACTGTGGAGGTCCAGGAAGCAGAGAGATGGCTAA
  GGCTCATCAGGACCGTATGATCTCCCAAGTGTCCAGCTACTGAGTACCACAAGGTGATGG
  GTGGGAGGGTCCTCCCACGGAAGGATACCGCAGTCCCTAGGGGTTGCAAGCCCCACATGT
  TCCACTGGCTGCTAGAGCTACCTACTCAATCAGCCCTGGGCATCACCATCAGGTACTCGG
  CCAAAATGACCTCTCTGCTTCCAGTCCTCAGTTCTGGTCAGCACCAGACAGGCCCATAAT
  TACA
  ============================================================

Primers for ID: 140
Primer pair 1, product size : 722
  Primer left                            Primer right
  start: 25211600                        start: 25212301
  end  : 25211621                        end  : 25212321
  tm   : 57                              tm   : 57
  seq  : TGAAATGGTCCATGTATTGCTC          seq  : TAATTATGGGCCTGTCTGGTG

  >140_primer_pair_1_product
  TGAAATGGTCCATGTATTGCTCAAAATAATTCTCATGAAAGTCAGGGTCTAGAACTCTGT
  CGGCTGAGGGCAGGTGCAGAGACTCAGGAGCTGGTTGGGATCATCAGGGATCTAGGCGGG
  TCAGGAGGAAGGGCAGCCAGTCTGTACTCACCTCTGGCCTGGAGGTTGCACAGTCCCAGT
  GACAGCAGCAGGACCAGGATCCAGGAGGGGACACCATGGTCCACAGGGTAACAAGGATGG
  AAGTTCATGCTTGATTCTGAGCCGGGCGCTGACTGTCATGTGATTTGGTCACATGACCGA
  CACAACGGGCGGGGCAGCATCACGTGATAGTCTGGCGGGGGCTGTCCTACTGTGGCTGGA
  TTCTAGTTGGAGGATCAGCCTACTCTTCTTCAGTTTCCCGGTTCCTCCAAATTTCTGGGC
  TCCTACTTGTTTCCACAGAGATGGATACTGTGGAGGTCCAGGAAGCAGAGAGATGGCTAA
  GGCTCATCAGGACCGTATGATCTCCCAAGTGTCCAGCTACTGAGTACCACAAGGTGATGG
  GTGGGAGGGTCCTCCCACGGAAGGATACCGCAGTCCCTAGGGGTTGCAAGCCCCACATGT
  TCCACTGGCTGCTAGAGCTACCTACTCAATCAGCCCTGGGCATCACCATCAGGTACTCGG
  CCAAAATGACCTCTCTGCTTCCAGTCCTCAGTTCTGGTCAGCACCAGACAGGCCCATAAT
  TA
  --------------------

Primer pair 2, product size : 724
  Primer left                            Primer right
  start: 25211600                        start: 25212303
  end  : 25211621                        end  : 25212323
  tm   : 57                              tm   : 57
  seq  : TGAAATGGTCCATGTATTGCTC          seq  : TGTAATTATGGGCCTGTCTGG

  >140_primer_pair_2_product
  TGAAATGGTCCATGTATTGCTCAAAATAATTCTCATGAAAGTCAGGGTCTAGAACTCTGT
  CGGCTGAGGGCAGGTGCAGAGACTCAGGAGCTGGTTGGGATCATCAGGGATCTAGGCGGG
  TCAGGAGGAAGGGCAGCCAGTCTGTACTCACCTCTGGCCTGGAGGTTGCACAGTCCCAGT
  GACAGCAGCAGGACCAGGATCCAGGAGGGGACACCATGGTCCACAGGGTAACAAGGATGG
  AAGTTCATGCTTGATTCTGAGCCGGGCGCTGACTGTCATGTGATTTGGTCACATGACCGA
  CACAACGGGCGGGGCAGCATCACGTGATAGTCTGGCGGGGGCTGTCCTACTGTGGCTGGA
  TTCTAGTTGGAGGATCAGCCTACTCTTCTTCAGTTTCCCGGTTCCTCCAAATTTCTGGGC
  TCCTACTTGTTTCCACAGAGATGGATACTGTGGAGGTCCAGGAAGCAGAGAGATGGCTAA
  GGCTCATCAGGACCGTATGATCTCCCAAGTGTCCAGCTACTGAGTACCACAAGGTGATGG
  GTGGGAGGGTCCTCCCACGGAAGGATACCGCAGTCCCTAGGGGTTGCAAGCCCCACATGT
  TCCACTGGCTGCTAGAGCTACCTACTCAATCAGCCCTGGGCATCACCATCAGGTACTCGG
  CCAAAATGACCTCTCTGCTTCCAGTCCTCAGTTCTGGTCAGCACCAGACAGGCCCATAAT
  TACA
  ============================================================

Primers for ID: 141
Primer pair 1, product size : 719
  Primer left                            Primer right
  start: 25211842                        start: 25212540
  end  : 25211862                        end  : 25212560
  tm   : 57                              tm   : 57
  seq  : GTTCATGCTTGATTCTGAGCC           seq  : ATGAGACACTCTGTGCCAATG

  >141_primer_pair_1_product
  GTTCATGCTTGATTCTGAGCCGGGCGCTGACTGTCATGTGATTTGGTCACATGACCGACA
  CAACGGGCGGGGCAGCATCACGTGATAGTCTGGCGGGGGCTGTCCTACTGTGGCTGGATT
  CTAGTTGGAGGATCAGCCTACTCTTCTTCAGTTTCCCGGTTCCTCCAAATTTCTGGGCTC
  CTACTTGTTTCCACAGAGATGGATACTGTGGAGGTCCAGGAAGCAGAGAGATGGCTAAGG
  CTCATCAGGACCGTATGATCTCCCAAGTGTCCAGCTACTGAGTACCACAAGGTGATGGGT
  GGGAGGGTCCTCCCACGGAAGGATACCGCAGTCCCTAGGGGTTGCAAGCCCCACATGTTC
  CACTGGCTGCTAGAGCTACCTACTCAATCAGCCCTGGGCATCACCATCAGGTACTCGGCC
  AAAATGACCTCTCTGCTTCCAGTCCTCAGTTCTGGTCAGCACCAGACAGGCCCATAATTA
  CAGAGCCAGGGAAACTGGAACATTTGTCTCCCCTTAGACAGTGGCAGCAGGAAGGTGGGG
  GGTTGTTGCAGAGGAACAGTGTCTCTGAGAGAGGACCTTGGACTTTCTGGGAATCTCTGA
  GCTGCCCGGTTCTCCCCACTGCTGGCACTGTGCCCACAGCCCAAACAGAATGGGGGAGAT
  GGAGGGGCAGGGCTTCTGTGGGAAGCTGCCCTCCACCTCATTGGCACAGAGTGTCTCAT
  --------------------

Primer pair 2, product size : 694
  Primer left                            Primer right
  start: 25211867                        start: 25212540
  end  : 25211887                        end  : 25212560
  tm   : 57                              tm   : 57
  seq  : GCTGACTGTCATGTGATTTGG           seq  : ATGAGACACTCTGTGCCAATG

  >141_primer_pair_2_product
  GCTGACTGTCATGTGATTTGGTCACATGACCGACACAACGGGCGGGGCAGCATCACGTGA
  TAGTCTGGCGGGGGCTGTCCTACTGTGGCTGGATTCTAGTTGGAGGATCAGCCTACTCTT
  CTTCAGTTTCCCGGTTCCTCCAAATTTCTGGGCTCCTACTTGTTTCCACAGAGATGGATA
  CTGTGGAGGTCCAGGAAGCAGAGAGATGGCTAAGGCTCATCAGGACCGTATGATCTCCCA
  AGTGTCCAGCTACTGAGTACCACAAGGTGATGGGTGGGAGGGTCCTCCCACGGAAGGATA
  CCGCAGTCCCTAGGGGTTGCAAGCCCCACATGTTCCACTGGCTGCTAGAGCTACCTACTC
  AATCAGCCCTGGGCATCACCATCAGGTACTCGGCCAAAATGACCTCTCTGCTTCCAGTCC
  TCAGTTCTGGTCAGCACCAGACAGGCCCATAATTACAGAGCCAGGGAAACTGGAACATTT
  GTCTCCCCTTAGACAGTGGCAGCAGGAAGGTGGGGGGTTGTTGCAGAGGAACAGTGTCTC
  TGAGAGAGGACCTTGGACTTTCTGGGAATCTCTGAGCTGCCCGGTTCTCCCCACTGCTGG
  CACTGTGCCCACAGCCCAAACAGAATGGGGGAGATGGAGGGGCAGGGCTTCTGTGGGAAG
  CTGCCCTCCACCTCATTGGCACAGAGTGTCTCAT
  ============================================================

Primers for ID: 142
Primer pair 1, product size : 719
  Primer left                            Primer right
  start: 25211842                        start: 25212540
  end  : 25211862                        end  : 25212560
  tm   : 57                              tm   : 57
  seq  : GTTCATGCTTGATTCTGAGCC           seq  : ATGAGACACTCTGTGCCAATG

  >142_primer_pair_1_product
  GTTCATGCTTGATTCTGAGCCGGGCGCTGACTGTCATGTGATTTGGTCACATGACCGACA
  CAACGGGCGGGGCAGCATCACGTGATAGTCTGGCGGGGGCTGTCCTACTGTGGCTGGATT
  CTAGTTGGAGGATCAGCCTACTCTTCTTCAGTTTCCCGGTTCCTCCAAATTTCTGGGCTC
  CTACTTGTTTCCACAGAGATGGATACTGTGGAGGTCCAGGAAGCAGAGAGATGGCTAAGG
  CTCATCAGGACCGTATGATCTCCCAAGTGTCCAGCTACTGAGTACCACAAGGTGATGGGT
  GGGAGGGTCCTCCCACGGAAGGATACCGCAGTCCCTAGGGGTTGCAAGCCCCACATGTTC
  CACTGGCTGCTAGAGCTACCTACTCAATCAGCCCTGGGCATCACCATCAGGTACTCGGCC
  AAAATGACCTCTCTGCTTCCAGTCCTCAGTTCTGGTCAGCACCAGACAGGCCCATAATTA
  CAGAGCCAGGGAAACTGGAACATTTGTCTCCCCTTAGACAGTGGCAGCAGGAAGGTGGGG
  GGTTGTTGCAGAGGAACAGTGTCTCTGAGAGAGGACCTTGGACTTTCTGGGAATCTCTGA
  GCTGCCCGGTTCTCCCCACTGCTGGCACTGTGCCCACAGCCCAAACAGAATGGGGGAGAT
  GGAGGGGCAGGGCTTCTGTGGGAAGCTGCCCTCCACCTCATTGGCACAGAGTGTCTCAT
  --------------------

Primer pair 2, product size : 831
  Primer left                            Primer right
  start: 25211842                        start: 25212653
  end  : 25211862                        end  : 25212672
  tm   : 57                              tm   : 55
  seq  : GTTCATGCTTGATTCTGAGCC           seq  : ATGGACATGTTCCACTCACG

  >142_primer_pair_2_product
  GTTCATGCTTGATTCTGAGCCGGGCGCTGACTGTCATGTGATTTGGTCACATGACCGACA
  CAACGGGCGGGGCAGCATCACGTGATAGTCTGGCGGGGGCTGTCCTACTGTGGCTGGATT
  CTAGTTGGAGGATCAGCCTACTCTTCTTCAGTTTCCCGGTTCCTCCAAATTTCTGGGCTC
  CTACTTGTTTCCACAGAGATGGATACTGTGGAGGTCCAGGAAGCAGAGAGATGGCTAAGG
  CTCATCAGGACCGTATGATCTCCCAAGTGTCCAGCTACTGAGTACCACAAGGTGATGGGT
  GGGAGGGTCCTCCCACGGAAGGATACCGCAGTCCCTAGGGGTTGCAAGCCCCACATGTTC
  CACTGGCTGCTAGAGCTACCTACTCAATCAGCCCTGGGCATCACCATCAGGTACTCGGCC
  AAAATGACCTCTCTGCTTCCAGTCCTCAGTTCTGGTCAGCACCAGACAGGCCCATAATTA
  CAGAGCCAGGGAAACTGGAACATTTGTCTCCCCTTAGACAGTGGCAGCAGGAAGGTGGGG
  GGTTGTTGCAGAGGAACAGTGTCTCTGAGAGAGGACCTTGGACTTTCTGGGAATCTCTGA
  GCTGCCCGGTTCTCCCCACTGCTGGCACTGTGCCCACAGCCCAAACAGAATGGGGGAGAT
  GGAGGGGCAGGGCTTCTGTGGGAAGCTGCCCTCCACCTCATTGGCACAGAGTGTCTCATT
  GCAGAGAGAAAAAAGGACCAGTTTTCTCTCTGGCACCCAGGTCTGGAAGAGGAGTGACAT
  CCACGGAAGTTGGTGACTTGGACTGGCTGGCCGTGAGTGGAACATGTCCAT
  ============================================================

Primers for ID: 143
Primer pair 1, product size : 670
  Primer left                            Primer right
  start: 25209909                        start: 25210558
  end  : 25209929                        end  : 25210578
  tm   : 57                              tm   : 57
  seq  : GATGGCATGAAAGCAGATAGC           seq  : ACAACCATGATCTGTGCTCTG

  >143_primer_pair_1_product
  GATGGCATGAAAGCAGATAGCTACAAGGCTCTTGGACCAGGCTAGCACTGGGTCCTGCAC
  CCAGGGAGAGCCACCTCACCTTGACAGGGTTGGCAGGAAGGGGCCCTAGAAAGTCAGTAG
  GATACGGGTAGTCCATCATGGCGAGCACAGTAAATGCATTTCGGGCAAACCCAAAGAGCT
  GAGTCAGGTCCTTTGGGCTGGAAAGTGATTGACAGGTACCAAAGTTCTGGCTGATGGTGT
  CATAGGCTGGGAAGAGAGAGGCCAGGAGAAAAGGCTGAGGAAACTGCTGGCAAATGTGAA
  GGGCAAGAATGAATGCCCAAGGTGGGCAGCAGGTGAGGAAAGAGTCCCTCACCTCCCTGG
  AGGAACAAGTCTTTGATTTGCTGAAAGGCATCCCGCACAGCCTGGGCGCACTTGGGACTC
  TGGCCATAAAAGTCCTGGAGAAGAGACCAAGGTTGCTGCTGCCATTCTTGCACTGGCCTG
  GGGTACCCAAGTCCCCTCACTCACCGCTGTGACATCTCGGAAGAATTGGTAGGAGTCCCC
  AAGGCCTGCAACAGCTACAACAGGAGCGCTGGCTGCCAGTGCCCCAGCCACCAGGTGGGG
  GTACTTCATCCTCATGTAGGCACTCAGCATCCCCCCATAACTGGGAGTACAGAGCACAGA
  TCATGGTTGT
  --------------------

Primer pair 2, product size : 612
  Primer left                            Primer right
  start: 25209826                        start: 25210417
  end  : 25209847                        end  : 25210437
  tm   : 61                              tm   : 57
  seq  : AGCAGAATATCTCAGTGGAGGC          seq  : CCAATTCTTCCGAGATGTCAC

  >143_primer_pair_2_product
  AGCAGAATATCTCAGTGGAGGCCCCTTTCACAGAGCGTGGGTCAGGGCTGCTAGCTTCCA
  GGACACAACAGCAGATAGTGTCTGATGGCATGAAAGCAGATAGCTACAAGGCTCTTGGAC
  CAGGCTAGCACTGGGTCCTGCACCCAGGGAGAGCCACCTCACCTTGACAGGGTTGGCAGG
  AAGGGGCCCTAGAAAGTCAGTAGGATACGGGTAGTCCATCATGGCGAGCACAGTAAATGC
  ATTTCGGGCAAACCCAAAGAGCTGAGTCAGGTCCTTTGGGCTGGAAAGTGATTGACAGGT
  ACCAAAGTTCTGGCTGATGGTGTCATAGGCTGGGAAGAGAGAGGCCAGGAGAAAAGGCTG
  AGGAAACTGCTGGCAAATGTGAAGGGCAAGAATGAATGCCCAAGGTGGGCAGCAGGTGAG
  GAAAGAGTCCCTCACCTCCCTGGAGGAACAAGTCTTTGATTTGCTGAAAGGCATCCCGCA
  CAGCCTGGGCGCACTTGGGACTCTGGCCATAAAAGTCCTGGAGAAGAGACCAAGGTTGCT
  GCTGCCATTCTTGCACTGGCCTGGGGTACCCAAGTCCCCTCACTCACCGCTGTGACATCT
  CGGAAGAATTGG
  ============================================================

Primers for ID: 144
Primer pair 1, product size : 670
  Primer left                            Primer right
  start: 25209909                        start: 25210558
  end  : 25209929                        end  : 25210578
  tm   : 57                              tm   : 57
  seq  : GATGGCATGAAAGCAGATAGC           seq  : ACAACCATGATCTGTGCTCTG

  >144_primer_pair_1_product
  GATGGCATGAAAGCAGATAGCTACAAGGCTCTTGGACCAGGCTAGCACTGGGTCCTGCAC
  CCAGGGAGAGCCACCTCACCTTGACAGGGTTGGCAGGAAGGGGCCCTAGAAAGTCAGTAG
  GATACGGGTAGTCCATCATGGCGAGCACAGTAAATGCATTTCGGGCAAACCCAAAGAGCT
  GAGTCAGGTCCTTTGGGCTGGAAAGTGATTGACAGGTACCAAAGTTCTGGCTGATGGTGT
  CATAGGCTGGGAAGAGAGAGGCCAGGAGAAAAGGCTGAGGAAACTGCTGGCAAATGTGAA
  GGGCAAGAATGAATGCCCAAGGTGGGCAGCAGGTGAGGAAAGAGTCCCTCACCTCCCTGG
  AGGAACAAGTCTTTGATTTGCTGAAAGGCATCCCGCACAGCCTGGGCGCACTTGGGACTC
  TGGCCATAAAAGTCCTGGAGAAGAGACCAAGGTTGCTGCTGCCATTCTTGCACTGGCCTG
  GGGTACCCAAGTCCCCTCACTCACCGCTGTGACATCTCGGAAGAATTGGTAGGAGTCCCC
  AAGGCCTGCAACAGCTACAACAGGAGCGCTGGCTGCCAGTGCCCCAGCCACCAGGTGGGG
  GTACTTCATCCTCATGTAGGCACTCAGCATCCCCCCATAACTGGGAGTACAGAGCACAGA
  TCATGGTTGT
  --------------------

Primer pair 2, product size : 782
  Primer left                            Primer right
  start: 25209909                        start: 25210670
  end  : 25209929                        end  : 25210690
  tm   : 57                              tm   : 57
  seq  : GATGGCATGAAAGCAGATAGC           seq  : AGTTTCAGGTCCTTCTTTCCC

  >144_primer_pair_2_product
  GATGGCATGAAAGCAGATAGCTACAAGGCTCTTGGACCAGGCTAGCACTGGGTCCTGCAC
  CCAGGGAGAGCCACCTCACCTTGACAGGGTTGGCAGGAAGGGGCCCTAGAAAGTCAGTAG
  GATACGGGTAGTCCATCATGGCGAGCACAGTAAATGCATTTCGGGCAAACCCAAAGAGCT
  GAGTCAGGTCCTTTGGGCTGGAAAGTGATTGACAGGTACCAAAGTTCTGGCTGATGGTGT
  CATAGGCTGGGAAGAGAGAGGCCAGGAGAAAAGGCTGAGGAAACTGCTGGCAAATGTGAA
  GGGCAAGAATGAATGCCCAAGGTGGGCAGCAGGTGAGGAAAGAGTCCCTCACCTCCCTGG
  AGGAACAAGTCTTTGATTTGCTGAAAGGCATCCCGCACAGCCTGGGCGCACTTGGGACTC
  TGGCCATAAAAGTCCTGGAGAAGAGACCAAGGTTGCTGCTGCCATTCTTGCACTGGCCTG
  GGGTACCCAAGTCCCCTCACTCACCGCTGTGACATCTCGGAAGAATTGGTAGGAGTCCCC
  AAGGCCTGCAACAGCTACAACAGGAGCGCTGGCTGCCAGTGCCCCAGCCACCAGGTGGGG
  GTACTTCATCCTCATGTAGGCACTCAGCATCCCCCCATAACTGGGAGTACAGAGCACAGA
  TCATGGTTGTGGGAAGCTGCCCACAACTCAGGCGAGCAGCCTCACTGTCCTCCAGGCTGA
  GGTGCTAGGCTGCTCTTTCCCTGCTCAGAACGCCCAAGGGTGGGAAAGAAGGACCTGAAA
  CT
  ============================================================

Primers for ID: 145
Primer pair 1, product size : 661
  Primer left                            Primer right
  start: 25210129                        start: 25210770
  end  : 25210149                        end  : 25210789
  tm   : 57                              tm   : 55
  seq  : AAAGTTCTGGCTGATGGTGTC           seq  : TGTCTTCATTGCTGGGTCAG

  >145_primer_pair_1_product
  AAAGTTCTGGCTGATGGTGTCATAGGCTGGGAAGAGAGAGGCCAGGAGAAAAGGCTGAGG
  AAACTGCTGGCAAATGTGAAGGGCAAGAATGAATGCCCAAGGTGGGCAGCAGGTGAGGAA
  AGAGTCCCTCACCTCCCTGGAGGAACAAGTCTTTGATTTGCTGAAAGGCATCCCGCACAG
  CCTGGGCGCACTTGGGACTCTGGCCATAAAAGTCCTGGAGAAGAGACCAAGGTTGCTGCT
  GCCATTCTTGCACTGGCCTGGGGTACCCAAGTCCCCTCACTCACCGCTGTGACATCTCGG
  AAGAATTGGTAGGAGTCCCCAAGGCCTGCAACAGCTACAACAGGAGCGCTGGCTGCCAGT
  GCCCCAGCCACCAGGTGGGGGTACTTCATCCTCATGTAGGCACTCAGCATCCCCCCATAA
  CTGGGAGTACAGAGCACAGATCATGGTTGTGGGAAGCTGCCCACAACTCAGGCGAGCAGC
  CTCACTGTCCTCCAGGCTGAGGTGCTAGGCTGCTCTTTCCCTGCTCAGAACGCCCAAGGG
  TGGGAAAGAAGGACCTGAAACTGTCAGGCCCACACACCCTGATCCCAGGGCCAAGGCAGA
  TACAGCCTTCACTGGGAGAAGGCACCTGTGGGTGCCCTGCCCTGACCCAGCAATGAAGAC
  A
  --------------------

Primer pair 2, product size : 618
  Primer left                            Primer right
  start: 25210172                        start: 25210770
  end  : 25210192                        end  : 25210789
  tm   : 57                              tm   : 55
  seq  : AGGAGAAAAGGCTGAGGAAAC           seq  : TGTCTTCATTGCTGGGTCAG

  >145_primer_pair_2_product
  AGGAGAAAAGGCTGAGGAAACTGCTGGCAAATGTGAAGGGCAAGAATGAATGCCCAAGGT
  GGGCAGCAGGTGAGGAAAGAGTCCCTCACCTCCCTGGAGGAACAAGTCTTTGATTTGCTG
  AAAGGCATCCCGCACAGCCTGGGCGCACTTGGGACTCTGGCCATAAAAGTCCTGGAGAAG
  AGACCAAGGTTGCTGCTGCCATTCTTGCACTGGCCTGGGGTACCCAAGTCCCCTCACTCA
  CCGCTGTGACATCTCGGAAGAATTGGTAGGAGTCCCCAAGGCCTGCAACAGCTACAACAG
  GAGCGCTGGCTGCCAGTGCCCCAGCCACCAGGTGGGGGTACTTCATCCTCATGTAGGCAC
  TCAGCATCCCCCCATAACTGGGAGTACAGAGCACAGATCATGGTTGTGGGAAGCTGCCCA
  CAACTCAGGCGAGCAGCCTCACTGTCCTCCAGGCTGAGGTGCTAGGCTGCTCTTTCCCTG
  CTCAGAACGCCCAAGGGTGGGAAAGAAGGACCTGAAACTGTCAGGCCCACACACCCTGAT
  CCCAGGGCCAAGGCAGATACAGCCTTCACTGGGAGAAGGCACCTGTGGGTGCCCTGCCCT
  GACCCAGCAATGAAGACA
  ============================================================

Primers for ID: 146
Primer pair 1, product size : 692
  Primer left                            Primer right
  start: 25210129                        start: 25210799
  end  : 25210149                        end  : 25210820
  tm   : 57                              tm   : 59
  seq  : AAAGTTCTGGCTGATGGTGTC           seq  : GGGACAATTCCTTCTGACTTTG

  >146_primer_pair_1_product
  AAAGTTCTGGCTGATGGTGTCATAGGCTGGGAAGAGAGAGGCCAGGAGAAAAGGCTGAGG
  AAACTGCTGGCAAATGTGAAGGGCAAGAATGAATGCCCAAGGTGGGCAGCAGGTGAGGAA
  AGAGTCCCTCACCTCCCTGGAGGAACAAGTCTTTGATTTGCTGAAAGGCATCCCGCACAG
  CCTGGGCGCACTTGGGACTCTGGCCATAAAAGTCCTGGAGAAGAGACCAAGGTTGCTGCT
  GCCATTCTTGCACTGGCCTGGGGTACCCAAGTCCCCTCACTCACCGCTGTGACATCTCGG
  AAGAATTGGTAGGAGTCCCCAAGGCCTGCAACAGCTACAACAGGAGCGCTGGCTGCCAGT
  GCCCCAGCCACCAGGTGGGGGTACTTCATCCTCATGTAGGCACTCAGCATCCCCCCATAA
  CTGGGAGTACAGAGCACAGATCATGGTTGTGGGAAGCTGCCCACAACTCAGGCGAGCAGC
  CTCACTGTCCTCCAGGCTGAGGTGCTAGGCTGCTCTTTCCCTGCTCAGAACGCCCAAGGG
  TGGGAAAGAAGGACCTGAAACTGTCAGGCCCACACACCCTGATCCCAGGGCCAAGGCAGA
  TACAGCCTTCACTGGGAGAAGGCACCTGTGGGTGCCCTGCCCTGACCCAGCAATGAAGAC
  ATTGCAGAGACAAAGTCAGAAGGAATTGTCCC
  --------------------

Primer pair 2, product size : 661
  Primer left                            Primer right
  start: 25210129                        start: 25210770
  end  : 25210149                        end  : 25210789
  tm   : 57                              tm   : 55
  seq  : AAAGTTCTGGCTGATGGTGTC           seq  : TGTCTTCATTGCTGGGTCAG

  >146_primer_pair_2_product
  AAAGTTCTGGCTGATGGTGTCATAGGCTGGGAAGAGAGAGGCCAGGAGAAAAGGCTGAGG
  AAACTGCTGGCAAATGTGAAGGGCAAGAATGAATGCCCAAGGTGGGCAGCAGGTGAGGAA
  AGAGTCCCTCACCTCCCTGGAGGAACAAGTCTTTGATTTGCTGAAAGGCATCCCGCACAG
  CCTGGGCGCACTTGGGACTCTGGCCATAAAAGTCCTGGAGAAGAGACCAAGGTTGCTGCT
  GCCATTCTTGCACTGGCCTGGGGTACCCAAGTCCCCTCACTCACCGCTGTGACATCTCGG
  AAGAATTGGTAGGAGTCCCCAAGGCCTGCAACAGCTACAACAGGAGCGCTGGCTGCCAGT
  GCCCCAGCCACCAGGTGGGGGTACTTCATCCTCATGTAGGCACTCAGCATCCCCCCATAA
  CTGGGAGTACAGAGCACAGATCATGGTTGTGGGAAGCTGCCCACAACTCAGGCGAGCAGC
  CTCACTGTCCTCCAGGCTGAGGTGCTAGGCTGCTCTTTCCCTGCTCAGAACGCCCAAGGG
  TGGGAAAGAAGGACCTGAAACTGTCAGGCCCACACACCCTGATCCCAGGGCCAAGGCAGA
  TACAGCCTTCACTGGGAGAAGGCACCTGTGGGTGCCCTGCCCTGACCCAGCAATGAAGAC
  A
  ============================================================

Primers for ID: 147
Primer pair 1, product size : 692
  Primer left                            Primer right
  start: 25210129                        start: 25210799
  end  : 25210149                        end  : 25210820
  tm   : 57                              tm   : 59
  seq  : AAAGTTCTGGCTGATGGTGTC           seq  : GGGACAATTCCTTCTGACTTTG

  >147_primer_pair_1_product
  AAAGTTCTGGCTGATGGTGTCATAGGCTGGGAAGAGAGAGGCCAGGAGAAAAGGCTGAGG
  AAACTGCTGGCAAATGTGAAGGGCAAGAATGAATGCCCAAGGTGGGCAGCAGGTGAGGAA
  AGAGTCCCTCACCTCCCTGGAGGAACAAGTCTTTGATTTGCTGAAAGGCATCCCGCACAG
  CCTGGGCGCACTTGGGACTCTGGCCATAAAAGTCCTGGAGAAGAGACCAAGGTTGCTGCT
  GCCATTCTTGCACTGGCCTGGGGTACCCAAGTCCCCTCACTCACCGCTGTGACATCTCGG
  AAGAATTGGTAGGAGTCCCCAAGGCCTGCAACAGCTACAACAGGAGCGCTGGCTGCCAGT
  GCCCCAGCCACCAGGTGGGGGTACTTCATCCTCATGTAGGCACTCAGCATCCCCCCATAA
  CTGGGAGTACAGAGCACAGATCATGGTTGTGGGAAGCTGCCCACAACTCAGGCGAGCAGC
  CTCACTGTCCTCCAGGCTGAGGTGCTAGGCTGCTCTTTCCCTGCTCAGAACGCCCAAGGG
  TGGGAAAGAAGGACCTGAAACTGTCAGGCCCACACACCCTGATCCCAGGGCCAAGGCAGA
  TACAGCCTTCACTGGGAGAAGGCACCTGTGGGTGCCCTGCCCTGACCCAGCAATGAAGAC
  ATTGCAGAGACAAAGTCAGAAGGAATTGTCCC
  --------------------

Primer pair 2, product size : 661
  Primer left                            Primer right
  start: 25210129                        start: 25210770
  end  : 25210149                        end  : 25210789
  tm   : 57                              tm   : 55
  seq  : AAAGTTCTGGCTGATGGTGTC           seq  : TGTCTTCATTGCTGGGTCAG

  >147_primer_pair_2_product
  AAAGTTCTGGCTGATGGTGTCATAGGCTGGGAAGAGAGAGGCCAGGAGAAAAGGCTGAGG
  AAACTGCTGGCAAATGTGAAGGGCAAGAATGAATGCCCAAGGTGGGCAGCAGGTGAGGAA
  AGAGTCCCTCACCTCCCTGGAGGAACAAGTCTTTGATTTGCTGAAAGGCATCCCGCACAG
  CCTGGGCGCACTTGGGACTCTGGCCATAAAAGTCCTGGAGAAGAGACCAAGGTTGCTGCT
  GCCATTCTTGCACTGGCCTGGGGTACCCAAGTCCCCTCACTCACCGCTGTGACATCTCGG
  AAGAATTGGTAGGAGTCCCCAAGGCCTGCAACAGCTACAACAGGAGCGCTGGCTGCCAGT
  GCCCCAGCCACCAGGTGGGGGTACTTCATCCTCATGTAGGCACTCAGCATCCCCCCATAA
  CTGGGAGTACAGAGCACAGATCATGGTTGTGGGAAGCTGCCCACAACTCAGGCGAGCAGC
  CTCACTGTCCTCCAGGCTGAGGTGCTAGGCTGCTCTTTCCCTGCTCAGAACGCCCAAGGG
  TGGGAAAGAAGGACCTGAAACTGTCAGGCCCACACACCCTGATCCCAGGGCCAAGGCAGA
  TACAGCCTTCACTGGGAGAAGGCACCTGTGGGTGCCCTGCCCTGACCCAGCAATGAAGAC
  A
  ============================================================

Primers for ID: 148
Primer pair 1, product size : 635
  Primer left                            Primer right
  start: 25210417                        start: 25211030
  end  : 25210437                        end  : 25211051
  tm   : 57                              tm   : 61
  seq  : GTGACATCTCGGAAGAATTGG           seq  : AGATCTGGACCCATGAGAGAAC

  >148_primer_pair_1_product
  GTGACATCTCGGAAGAATTGGTAGGAGTCCCCAAGGCCTGCAACAGCTACAACAGGAGCG
  CTGGCTGCCAGTGCCCCAGCCACCAGGTGGGGGTACTTCATCCTCATGTAGGCACTCAGC
  ATCCCCCCATAACTGGGAGTACAGAGCACAGATCATGGTTGTGGGAAGCTGCCCACAACT
  CAGGCGAGCAGCCTCACTGTCCTCCAGGCTGAGGTGCTAGGCTGCTCTTTCCCTGCTCAG
  AACGCCCAAGGGTGGGAAAGAAGGACCTGAAACTGTCAGGCCCACACACCCTGATCCCAG
  GGCCAAGGCAGATACAGCCTTCACTGGGAGAAGGCACCTGTGGGTGCCCTGCCCTGACCC
  AGCAATGAAGACATTGCAGAGACAAAGTCAGAAGGAATTGTCCCACTAGTGGGAACAACA
  TAGCATACACTGCCTATGAGGTCCACTCAAGGAGGGCTTCCAGAAGGAGGTAAAGCTAGA
  CCCCGCCCTTCCACATGTGGGGTAGGCATAGGATGTTGAGACTGTAAGAGACATCTCTTT
  GGCCCTCCTTGTATAGGGTGTCAATCGGCACAACAGGGTGGAGCCTTAGAGTAGGGTAAG
  ATTAGGACTCTAGGTTCTCTCATGGGTCCAGATCT
  --------------------

Primer pair 2, product size : 608
  Primer left                            Primer right
  start: 25210213                        start: 25210799
  end  : 25210232                        end  : 25210820
  tm   : 53                              tm   : 59
  seq  : AAGAATGAATGCCCAAGGTG            seq  : GGGACAATTCCTTCTGACTTTG

  >148_primer_pair_2_product
  AAGAATGAATGCCCAAGGTGGGCAGCAGGTGAGGAAAGAGTCCCTCACCTCCCTGGAGGA
  ACAAGTCTTTGATTTGCTGAAAGGCATCCCGCACAGCCTGGGCGCACTTGGGACTCTGGC
  CATAAAAGTCCTGGAGAAGAGACCAAGGTTGCTGCTGCCATTCTTGCACTGGCCTGGGGT
  ACCCAAGTCCCCTCACTCACCGCTGTGACATCTCGGAAGAATTGGTAGGAGTCCCCAAGG
  CCTGCAACAGCTACAACAGGAGCGCTGGCTGCCAGTGCCCCAGCCACCAGGTGGGGGTAC
  TTCATCCTCATGTAGGCACTCAGCATCCCCCCATAACTGGGAGTACAGAGCACAGATCAT
  GGTTGTGGGAAGCTGCCCACAACTCAGGCGAGCAGCCTCACTGTCCTCCAGGCTGAGGTG
  CTAGGCTGCTCTTTCCCTGCTCAGAACGCCCAAGGGTGGGAAAGAAGGACCTGAAACTGT
  CAGGCCCACACACCCTGATCCCAGGGCCAAGGCAGATACAGCCTTCACTGGGAGAAGGCA
  CCTGTGGGTGCCCTGCCCTGACCCAGCAATGAAGACATTGCAGAGACAAAGTCAGAAGGA
  ATTGTCCC
  ============================================================

Primers for ID: 149
Primer pair 1, product size : 635
  Primer left                            Primer right
  start: 25210417                        start: 25211030
  end  : 25210437                        end  : 25211051
  tm   : 57                              tm   : 61
  seq  : GTGACATCTCGGAAGAATTGG           seq  : AGATCTGGACCCATGAGAGAAC

  >149_primer_pair_1_product
  GTGACATCTCGGAAGAATTGGTAGGAGTCCCCAAGGCCTGCAACAGCTACAACAGGAGCG
  CTGGCTGCCAGTGCCCCAGCCACCAGGTGGGGGTACTTCATCCTCATGTAGGCACTCAGC
  ATCCCCCCATAACTGGGAGTACAGAGCACAGATCATGGTTGTGGGAAGCTGCCCACAACT
  CAGGCGAGCAGCCTCACTGTCCTCCAGGCTGAGGTGCTAGGCTGCTCTTTCCCTGCTCAG
  AACGCCCAAGGGTGGGAAAGAAGGACCTGAAACTGTCAGGCCCACACACCCTGATCCCAG
  GGCCAAGGCAGATACAGCCTTCACTGGGAGAAGGCACCTGTGGGTGCCCTGCCCTGACCC
  AGCAATGAAGACATTGCAGAGACAAAGTCAGAAGGAATTGTCCCACTAGTGGGAACAACA
  TAGCATACACTGCCTATGAGGTCCACTCAAGGAGGGCTTCCAGAAGGAGGTAAAGCTAGA
  CCCCGCCCTTCCACATGTGGGGTAGGCATAGGATGTTGAGACTGTAAGAGACATCTCTTT
  GGCCCTCCTTGTATAGGGTGTCAATCGGCACAACAGGGTGGAGCCTTAGAGTAGGGTAAG
  ATTAGGACTCTAGGTTCTCTCATGGGTCCAGATCT
  --------------------

Primer pair 2, product size : 775
  Primer left                            Primer right
  start: 25210277                        start: 25211030
  end  : 25210297                        end  : 25211051
  tm   : 55                              tm   : 61
  seq  : GTCTTTGATTTGCTGAAAGGC           seq  : AGATCTGGACCCATGAGAGAAC

  >149_primer_pair_2_product
  GTCTTTGATTTGCTGAAAGGCATCCCGCACAGCCTGGGCGCACTTGGGACTCTGGCCATA
  AAAGTCCTGGAGAAGAGACCAAGGTTGCTGCTGCCATTCTTGCACTGGCCTGGGGTACCC
  AAGTCCCCTCACTCACCGCTGTGACATCTCGGAAGAATTGGTAGGAGTCCCCAAGGCCTG
  CAACAGCTACAACAGGAGCGCTGGCTGCCAGTGCCCCAGCCACCAGGTGGGGGTACTTCA
  TCCTCATGTAGGCACTCAGCATCCCCCCATAACTGGGAGTACAGAGCACAGATCATGGTT
  GTGGGAAGCTGCCCACAACTCAGGCGAGCAGCCTCACTGTCCTCCAGGCTGAGGTGCTAG
  GCTGCTCTTTCCCTGCTCAGAACGCCCAAGGGTGGGAAAGAAGGACCTGAAACTGTCAGG
  CCCACACACCCTGATCCCAGGGCCAAGGCAGATACAGCCTTCACTGGGAGAAGGCACCTG
  TGGGTGCCCTGCCCTGACCCAGCAATGAAGACATTGCAGAGACAAAGTCAGAAGGAATTG
  TCCCACTAGTGGGAACAACATAGCATACACTGCCTATGAGGTCCACTCAAGGAGGGCTTC
  CAGAAGGAGGTAAAGCTAGACCCCGCCCTTCCACATGTGGGGTAGGCATAGGATGTTGAG
  ACTGTAAGAGACATCTCTTTGGCCCTCCTTGTATAGGGTGTCAATCGGCACAACAGGGTG
  GAGCCTTAGAGTAGGGTAAGATTAGGACTCTAGGTTCTCTCATGGGTCCAGATCT
  ============================================================

Primers for ID: 159
Primer pair 1, product size : 620
  Primer left                            Primer right
  start: 25211383                        start: 25211982
  end  : 25211403                        end  : 25212002
  tm   : 57                              tm   : 57
  seq  : ATGAAGCCAGAGTTGTTAGCG           seq  : AACCGGGAAACTGAAGAAGAG

  >159_primer_pair_1_product
  ATGAAGCCAGAGTTGTTAGCGAAGGACCAGATATCCCCCTCATTCCCTGTGTAGAAAAAG
  ATGGGCCCTTCGCCCATCTTCCAGAACTTATCTGTTGGAAGTAAATGAGTTTCCATAAGG
  CCAGGGAAACGCAGGTAGGAACCCATGCGGTCGAGCCAGCACTCACCTGACACTAGGAAC
  CGCTGGCCAAAGGTTTTGTTGCCGAAACTCTCAAAGTTGAAATGGTCCATGTATTGCTCA
  AAATAATTCTCATGAAAGTCAGGGTCTAGAACTCTGTCGGCTGAGGGCAGGTGCAGAGAC
  TCAGGAGCTGGTTGGGATCATCAGGGATCTAGGCGGGTCAGGAGGAAGGGCAGCCAGTCT
  GTACTCACCTCTGGCCTGGAGGTTGCACAGTCCCAGTGACAGCAGCAGGACCAGGATCCA
  GGAGGGGACACCATGGTCCACAGGGTAACAAGGATGGAAGTTCATGCTTGATTCTGAGCC
  GGGCGCTGACTGTCATGTGATTTGGTCACATGACCGACACAACGGGCGGGGCAGCATCAC
  GTGATAGTCTGGCGGGGGCTGTCCTACTGTGGCTGGATTCTAGTTGGAGGATCAGCCTAC
  TCTTCTTCAGTTTCCCGGTT
  --------------------

Primer pair 2, product size : 612
  Primer left                            Primer right
  start: 25211391                        start: 25211982
  end  : 25211412                        end  : 25212002
  tm   : 61                              tm   : 57
  seq  : AGAGTTGTTAGCGAAGGACCAG          seq  : AACCGGGAAACTGAAGAAGAG

  >159_primer_pair_2_product
  AGAGTTGTTAGCGAAGGACCAGATATCCCCCTCATTCCCTGTGTAGAAAAAGATGGGCCC
  TTCGCCCATCTTCCAGAACTTATCTGTTGGAAGTAAATGAGTTTCCATAAGGCCAGGGAA
  ACGCAGGTAGGAACCCATGCGGTCGAGCCAGCACTCACCTGACACTAGGAACCGCTGGCC
  AAAGGTTTTGTTGCCGAAACTCTCAAAGTTGAAATGGTCCATGTATTGCTCAAAATAATT
  CTCATGAAAGTCAGGGTCTAGAACTCTGTCGGCTGAGGGCAGGTGCAGAGACTCAGGAGC
  TGGTTGGGATCATCAGGGATCTAGGCGGGTCAGGAGGAAGGGCAGCCAGTCTGTACTCAC
  CTCTGGCCTGGAGGTTGCACAGTCCCAGTGACAGCAGCAGGACCAGGATCCAGGAGGGGA
  CACCATGGTCCACAGGGTAACAAGGATGGAAGTTCATGCTTGATTCTGAGCCGGGCGCTG
  ACTGTCATGTGATTTGGTCACATGACCGACACAACGGGCGGGGCAGCATCACGTGATAGT
  CTGGCGGGGGCTGTCCTACTGTGGCTGGATTCTAGTTGGAGGATCAGCCTACTCTTCTTC
  AGTTTCCCGGTT
  ============================================================

Primers for ID: 160
Primer pair 1, product size : 620
  Primer left                            Primer right
  start: 25211383                        start: 25211982
  end  : 25211403                        end  : 25212002
  tm   : 57                              tm   : 57
  seq  : ATGAAGCCAGAGTTGTTAGCG           seq  : AACCGGGAAACTGAAGAAGAG

  >160_primer_pair_1_product
  ATGAAGCCAGAGTTGTTAGCGAAGGACCAGATATCCCCCTCATTCCCTGTGTAGAAAAAG
  ATGGGCCCTTCGCCCATCTTCCAGAACTTATCTGTTGGAAGTAAATGAGTTTCCATAAGG
  CCAGGGAAACGCAGGTAGGAACCCATGCGGTCGAGCCAGCACTCACCTGACACTAGGAAC
  CGCTGGCCAAAGGTTTTGTTGCCGAAACTCTCAAAGTTGAAATGGTCCATGTATTGCTCA
  AAATAATTCTCATGAAAGTCAGGGTCTAGAACTCTGTCGGCTGAGGGCAGGTGCAGAGAC
  TCAGGAGCTGGTTGGGATCATCAGGGATCTAGGCGGGTCAGGAGGAAGGGCAGCCAGTCT
  GTACTCACCTCTGGCCTGGAGGTTGCACAGTCCCAGTGACAGCAGCAGGACCAGGATCCA
  GGAGGGGACACCATGGTCCACAGGGTAACAAGGATGGAAGTTCATGCTTGATTCTGAGCC
  GGGCGCTGACTGTCATGTGATTTGGTCACATGACCGACACAACGGGCGGGGCAGCATCAC
  GTGATAGTCTGGCGGGGGCTGTCCTACTGTGGCTGGATTCTAGTTGGAGGATCAGCCTAC
  TCTTCTTCAGTTTCCCGGTT
  --------------------

Primer pair 2, product size : 612
  Primer left                            Primer right
  start: 25211391                        start: 25211982
  end  : 25211412                        end  : 25212002
  tm   : 61                              tm   : 57
  seq  : AGAGTTGTTAGCGAAGGACCAG          seq  : AACCGGGAAACTGAAGAAGAG

  >160_primer_pair_2_product
  AGAGTTGTTAGCGAAGGACCAGATATCCCCCTCATTCCCTGTGTAGAAAAAGATGGGCCC
  TTCGCCCATCTTCCAGAACTTATCTGTTGGAAGTAAATGAGTTTCCATAAGGCCAGGGAA
  ACGCAGGTAGGAACCCATGCGGTCGAGCCAGCACTCACCTGACACTAGGAACCGCTGGCC
  AAAGGTTTTGTTGCCGAAACTCTCAAAGTTGAAATGGTCCATGTATTGCTCAAAATAATT
  CTCATGAAAGTCAGGGTCTAGAACTCTGTCGGCTGAGGGCAGGTGCAGAGACTCAGGAGC
  TGGTTGGGATCATCAGGGATCTAGGCGGGTCAGGAGGAAGGGCAGCCAGTCTGTACTCAC
  CTCTGGCCTGGAGGTTGCACAGTCCCAGTGACAGCAGCAGGACCAGGATCCAGGAGGGGA
  CACCATGGTCCACAGGGTAACAAGGATGGAAGTTCATGCTTGATTCTGAGCCGGGCGCTG
  ACTGTCATGTGATTTGGTCACATGACCGACACAACGGGCGGGGCAGCATCACGTGATAGT
  CTGGCGGGGGCTGTCCTACTGTGGCTGGATTCTAGTTGGAGGATCAGCCTACTCTTCTTC
  AGTTTCCCGGTT
  ============================================================

Primers for ID: 161
Primer pair 1, product size : 620
  Primer left                            Primer right
  start: 25211383                        start: 25211982
  end  : 25211403                        end  : 25212002
  tm   : 57                              tm   : 57
  seq  : ATGAAGCCAGAGTTGTTAGCG           seq  : AACCGGGAAACTGAAGAAGAG

  >161_primer_pair_1_product
  ATGAAGCCAGAGTTGTTAGCGAAGGACCAGATATCCCCCTCATTCCCTGTGTAGAAAAAG
  ATGGGCCCTTCGCCCATCTTCCAGAACTTATCTGTTGGAAGTAAATGAGTTTCCATAAGG
  CCAGGGAAACGCAGGTAGGAACCCATGCGGTCGAGCCAGCACTCACCTGACACTAGGAAC
  CGCTGGCCAAAGGTTTTGTTGCCGAAACTCTCAAAGTTGAAATGGTCCATGTATTGCTCA
  AAATAATTCTCATGAAAGTCAGGGTCTAGAACTCTGTCGGCTGAGGGCAGGTGCAGAGAC
  TCAGGAGCTGGTTGGGATCATCAGGGATCTAGGCGGGTCAGGAGGAAGGGCAGCCAGTCT
  GTACTCACCTCTGGCCTGGAGGTTGCACAGTCCCAGTGACAGCAGCAGGACCAGGATCCA
  GGAGGGGACACCATGGTCCACAGGGTAACAAGGATGGAAGTTCATGCTTGATTCTGAGCC
  GGGCGCTGACTGTCATGTGATTTGGTCACATGACCGACACAACGGGCGGGGCAGCATCAC
  GTGATAGTCTGGCGGGGGCTGTCCTACTGTGGCTGGATTCTAGTTGGAGGATCAGCCTAC
  TCTTCTTCAGTTTCCCGGTT
  --------------------

Primer pair 2, product size : 612
  Primer left                            Primer right
  start: 25211391                        start: 25211982
  end  : 25211412                        end  : 25212002
  tm   : 61                              tm   : 57
  seq  : AGAGTTGTTAGCGAAGGACCAG          seq  : AACCGGGAAACTGAAGAAGAG

  >161_primer_pair_2_product
  AGAGTTGTTAGCGAAGGACCAGATATCCCCCTCATTCCCTGTGTAGAAAAAGATGGGCCC
  TTCGCCCATCTTCCAGAACTTATCTGTTGGAAGTAAATGAGTTTCCATAAGGCCAGGGAA
  ACGCAGGTAGGAACCCATGCGGTCGAGCCAGCACTCACCTGACACTAGGAACCGCTGGCC
  AAAGGTTTTGTTGCCGAAACTCTCAAAGTTGAAATGGTCCATGTATTGCTCAAAATAATT
  CTCATGAAAGTCAGGGTCTAGAACTCTGTCGGCTGAGGGCAGGTGCAGAGACTCAGGAGC
  TGGTTGGGATCATCAGGGATCTAGGCGGGTCAGGAGGAAGGGCAGCCAGTCTGTACTCAC
  CTCTGGCCTGGAGGTTGCACAGTCCCAGTGACAGCAGCAGGACCAGGATCCAGGAGGGGA
  CACCATGGTCCACAGGGTAACAAGGATGGAAGTTCATGCTTGATTCTGAGCCGGGCGCTG
  ACTGTCATGTGATTTGGTCACATGACCGACACAACGGGCGGGGCAGCATCACGTGATAGT
  CTGGCGGGGGCTGTCCTACTGTGGCTGGATTCTAGTTGGAGGATCAGCCTACTCTTCTTC
  AGTTTCCCGGTT
  ============================================================

Primers for ID: 162
Primer pair 1, product size : 722
  Primer left                            Primer right
  start: 25211600                        start: 25212301
  end  : 25211621                        end  : 25212321
  tm   : 57                              tm   : 57
  seq  : TGAAATGGTCCATGTATTGCTC          seq  : TAATTATGGGCCTGTCTGGTG

  >162_primer_pair_1_product
  TGAAATGGTCCATGTATTGCTCAAAATAATTCTCATGAAAGTCAGGGTCTAGAACTCTGT
  CGGCTGAGGGCAGGTGCAGAGACTCAGGAGCTGGTTGGGATCATCAGGGATCTAGGCGGG
  TCAGGAGGAAGGGCAGCCAGTCTGTACTCACCTCTGGCCTGGAGGTTGCACAGTCCCAGT
  GACAGCAGCAGGACCAGGATCCAGGAGGGGACACCATGGTCCACAGGGTAACAAGGATGG
  AAGTTCATGCTTGATTCTGAGCCGGGCGCTGACTGTCATGTGATTTGGTCACATGACCGA
  CACAACGGGCGGGGCAGCATCACGTGATAGTCTGGCGGGGGCTGTCCTACTGTGGCTGGA
  TTCTAGTTGGAGGATCAGCCTACTCTTCTTCAGTTTCCCGGTTCCTCCAAATTTCTGGGC
  TCCTACTTGTTTCCACAGAGATGGATACTGTGGAGGTCCAGGAAGCAGAGAGATGGCTAA
  GGCTCATCAGGACCGTATGATCTCCCAAGTGTCCAGCTACTGAGTACCACAAGGTGATGG
  GTGGGAGGGTCCTCCCACGGAAGGATACCGCAGTCCCTAGGGGTTGCAAGCCCCACATGT
  TCCACTGGCTGCTAGAGCTACCTACTCAATCAGCCCTGGGCATCACCATCAGGTACTCGG
  CCAAAATGACCTCTCTGCTTCCAGTCCTCAGTTCTGGTCAGCACCAGACAGGCCCATAAT
  TA
  --------------------

Primer pair 2, product size : 724
  Primer left                            Primer right
  start: 25211600                        start: 25212303
  end  : 25211621                        end  : 25212323
  tm   : 57                              tm   : 57
  seq  : TGAAATGGTCCATGTATTGCTC          seq  : TGTAATTATGGGCCTGTCTGG

  >162_primer_pair_2_product
  TGAAATGGTCCATGTATTGCTCAAAATAATTCTCATGAAAGTCAGGGTCTAGAACTCTGT
  CGGCTGAGGGCAGGTGCAGAGACTCAGGAGCTGGTTGGGATCATCAGGGATCTAGGCGGG
  TCAGGAGGAAGGGCAGCCAGTCTGTACTCACCTCTGGCCTGGAGGTTGCACAGTCCCAGT
  GACAGCAGCAGGACCAGGATCCAGGAGGGGACACCATGGTCCACAGGGTAACAAGGATGG
  AAGTTCATGCTTGATTCTGAGCCGGGCGCTGACTGTCATGTGATTTGGTCACATGACCGA
  CACAACGGGCGGGGCAGCATCACGTGATAGTCTGGCGGGGGCTGTCCTACTGTGGCTGGA
  TTCTAGTTGGAGGATCAGCCTACTCTTCTTCAGTTTCCCGGTTCCTCCAAATTTCTGGGC
  TCCTACTTGTTTCCACAGAGATGGATACTGTGGAGGTCCAGGAAGCAGAGAGATGGCTAA
  GGCTCATCAGGACCGTATGATCTCCCAAGTGTCCAGCTACTGAGTACCACAAGGTGATGG
  GTGGGAGGGTCCTCCCACGGAAGGATACCGCAGTCCCTAGGGGTTGCAAGCCCCACATGT
  TCCACTGGCTGCTAGAGCTACCTACTCAATCAGCCCTGGGCATCACCATCAGGTACTCGG
  CCAAAATGACCTCTCTGCTTCCAGTCCTCAGTTCTGGTCAGCACCAGACAGGCCCATAAT
  TACA
  ============================================================

Primers for ID: 163
Primer pair 1, product size : 722
  Primer left                            Primer right
  start: 25211600                        start: 25212301
  end  : 25211621                        end  : 25212321
  tm   : 57                              tm   : 57
  seq  : TGAAATGGTCCATGTATTGCTC          seq  : TAATTATGGGCCTGTCTGGTG

  >163_primer_pair_1_product
  TGAAATGGTCCATGTATTGCTCAAAATAATTCTCATGAAAGTCAGGGTCTAGAACTCTGT
  CGGCTGAGGGCAGGTGCAGAGACTCAGGAGCTGGTTGGGATCATCAGGGATCTAGGCGGG
  TCAGGAGGAAGGGCAGCCAGTCTGTACTCACCTCTGGCCTGGAGGTTGCACAGTCCCAGT
  GACAGCAGCAGGACCAGGATCCAGGAGGGGACACCATGGTCCACAGGGTAACAAGGATGG
  AAGTTCATGCTTGATTCTGAGCCGGGCGCTGACTGTCATGTGATTTGGTCACATGACCGA
  CACAACGGGCGGGGCAGCATCACGTGATAGTCTGGCGGGGGCTGTCCTACTGTGGCTGGA
  TTCTAGTTGGAGGATCAGCCTACTCTTCTTCAGTTTCCCGGTTCCTCCAAATTTCTGGGC
  TCCTACTTGTTTCCACAGAGATGGATACTGTGGAGGTCCAGGAAGCAGAGAGATGGCTAA
  GGCTCATCAGGACCGTATGATCTCCCAAGTGTCCAGCTACTGAGTACCACAAGGTGATGG
  GTGGGAGGGTCCTCCCACGGAAGGATACCGCAGTCCCTAGGGGTTGCAAGCCCCACATGT
  TCCACTGGCTGCTAGAGCTACCTACTCAATCAGCCCTGGGCATCACCATCAGGTACTCGG
  CCAAAATGACCTCTCTGCTTCCAGTCCTCAGTTCTGGTCAGCACCAGACAGGCCCATAAT
  TA
  --------------------

Primer pair 2, product size : 724
  Primer left                            Primer right
  start: 25211600                        start: 25212303
  end  : 25211621                        end  : 25212323
  tm   : 57                              tm   : 57
  seq  : TGAAATGGTCCATGTATTGCTC          seq  : TGTAATTATGGGCCTGTCTGG

  >163_primer_pair_2_product
  TGAAATGGTCCATGTATTGCTCAAAATAATTCTCATGAAAGTCAGGGTCTAGAACTCTGT
  CGGCTGAGGGCAGGTGCAGAGACTCAGGAGCTGGTTGGGATCATCAGGGATCTAGGCGGG
  TCAGGAGGAAGGGCAGCCAGTCTGTACTCACCTCTGGCCTGGAGGTTGCACAGTCCCAGT
  GACAGCAGCAGGACCAGGATCCAGGAGGGGACACCATGGTCCACAGGGTAACAAGGATGG
  AAGTTCATGCTTGATTCTGAGCCGGGCGCTGACTGTCATGTGATTTGGTCACATGACCGA
  CACAACGGGCGGGGCAGCATCACGTGATAGTCTGGCGGGGGCTGTCCTACTGTGGCTGGA
  TTCTAGTTGGAGGATCAGCCTACTCTTCTTCAGTTTCCCGGTTCCTCCAAATTTCTGGGC
  TCCTACTTGTTTCCACAGAGATGGATACTGTGGAGGTCCAGGAAGCAGAGAGATGGCTAA
  GGCTCATCAGGACCGTATGATCTCCCAAGTGTCCAGCTACTGAGTACCACAAGGTGATGG
  GTGGGAGGGTCCTCCCACGGAAGGATACCGCAGTCCCTAGGGGTTGCAAGCCCCACATGT
  TCCACTGGCTGCTAGAGCTACCTACTCAATCAGCCCTGGGCATCACCATCAGGTACTCGG
  CCAAAATGACCTCTCTGCTTCCAGTCCTCAGTTCTGGTCAGCACCAGACAGGCCCATAAT
  TACA
  ============================================================

Primers for ID: 164
Primer pair 1, product size : 602
  Primer left                            Primer right
  start: 25211842                        start: 25212422
  end  : 25211862                        end  : 25212443
  tm   : 57                              tm   : 61
  seq  : GTTCATGCTTGATTCTGAGCC           seq  : GCTCAGAGATTCCCAGAAAGTC

  >164_primer_pair_1_product
  GTTCATGCTTGATTCTGAGCCGGGCGCTGACTGTCATGTGATTTGGTCACATGACCGACA
  CAACGGGCGGGGCAGCATCACGTGATAGTCTGGCGGGGGCTGTCCTACTGTGGCTGGATT
  CTAGTTGGAGGATCAGCCTACTCTTCTTCAGTTTCCCGGTTCCTCCAAATTTCTGGGCTC
  CTACTTGTTTCCACAGAGATGGATACTGTGGAGGTCCAGGAAGCAGAGAGATGGCTAAGG
  CTCATCAGGACCGTATGATCTCCCAAGTGTCCAGCTACTGAGTACCACAAGGTGATGGGT
  GGGAGGGTCCTCCCACGGAAGGATACCGCAGTCCCTAGGGGTTGCAAGCCCCACATGTTC
  CACTGGCTGCTAGAGCTACCTACTCAATCAGCCCTGGGCATCACCATCAGGTACTCGGCC
  AAAATGACCTCTCTGCTTCCAGTCCTCAGTTCTGGTCAGCACCAGACAGGCCCATAATTA
  CAGAGCCAGGGAAACTGGAACATTTGTCTCCCCTTAGACAGTGGCAGCAGGAAGGTGGGG
  GGTTGTTGCAGAGGAACAGTGTCTCTGAGAGAGGACCTTGGACTTTCTGGGAATCTCTGA
  GC
  --------------------

Primer pair 2, product size : 601
  Primer left                            Primer right
  start: 25211842                        start: 25212421
  end  : 25211862                        end  : 25212442
  tm   : 57                              tm   : 61
  seq  : GTTCATGCTTGATTCTGAGCC           seq  : CTCAGAGATTCCCAGAAAGTCC

  >164_primer_pair_2_product
  GTTCATGCTTGATTCTGAGCCGGGCGCTGACTGTCATGTGATTTGGTCACATGACCGACA
  CAACGGGCGGGGCAGCATCACGTGATAGTCTGGCGGGGGCTGTCCTACTGTGGCTGGATT
  CTAGTTGGAGGATCAGCCTACTCTTCTTCAGTTTCCCGGTTCCTCCAAATTTCTGGGCTC
  CTACTTGTTTCCACAGAGATGGATACTGTGGAGGTCCAGGAAGCAGAGAGATGGCTAAGG
  CTCATCAGGACCGTATGATCTCCCAAGTGTCCAGCTACTGAGTACCACAAGGTGATGGGT
  GGGAGGGTCCTCCCACGGAAGGATACCGCAGTCCCTAGGGGTTGCAAGCCCCACATGTTC
  CACTGGCTGCTAGAGCTACCTACTCAATCAGCCCTGGGCATCACCATCAGGTACTCGGCC
  AAAATGACCTCTCTGCTTCCAGTCCTCAGTTCTGGTCAGCACCAGACAGGCCCATAATTA
  CAGAGCCAGGGAAACTGGAACATTTGTCTCCCCTTAGACAGTGGCAGCAGGAAGGTGGGG
  GGTTGTTGCAGAGGAACAGTGTCTCTGAGAGAGGACCTTGGACTTTCTGGGAATCTCTGA
  G
  ============================================================

Primers for ID: 165
Primer pair 1, product size : 719
  Primer left                            Primer right
  start: 25211842                        start: 25212540
  end  : 25211862                        end  : 25212560
  tm   : 57                              tm   : 57
  seq  : GTTCATGCTTGATTCTGAGCC           seq  : ATGAGACACTCTGTGCCAATG

  >165_primer_pair_1_product
  GTTCATGCTTGATTCTGAGCCGGGCGCTGACTGTCATGTGATTTGGTCACATGACCGACA
  CAACGGGCGGGGCAGCATCACGTGATAGTCTGGCGGGGGCTGTCCTACTGTGGCTGGATT
  CTAGTTGGAGGATCAGCCTACTCTTCTTCAGTTTCCCGGTTCCTCCAAATTTCTGGGCTC
  CTACTTGTTTCCACAGAGATGGATACTGTGGAGGTCCAGGAAGCAGAGAGATGGCTAAGG
  CTCATCAGGACCGTATGATCTCCCAAGTGTCCAGCTACTGAGTACCACAAGGTGATGGGT
  GGGAGGGTCCTCCCACGGAAGGATACCGCAGTCCCTAGGGGTTGCAAGCCCCACATGTTC
  CACTGGCTGCTAGAGCTACCTACTCAATCAGCCCTGGGCATCACCATCAGGTACTCGGCC
  AAAATGACCTCTCTGCTTCCAGTCCTCAGTTCTGGTCAGCACCAGACAGGCCCATAATTA
  CAGAGCCAGGGAAACTGGAACATTTGTCTCCCCTTAGACAGTGGCAGCAGGAAGGTGGGG
  GGTTGTTGCAGAGGAACAGTGTCTCTGAGAGAGGACCTTGGACTTTCTGGGAATCTCTGA
  GCTGCCCGGTTCTCCCCACTGCTGGCACTGTGCCCACAGCCCAAACAGAATGGGGGAGAT
  GGAGGGGCAGGGCTTCTGTGGGAAGCTGCCCTCCACCTCATTGGCACAGAGTGTCTCAT
  --------------------

Primer pair 2, product size : 602
  Primer left                            Primer right
  start: 25211842                        start: 25212422
  end  : 25211862                        end  : 25212443
  tm   : 57                              tm   : 61
  seq  : GTTCATGCTTGATTCTGAGCC           seq  : GCTCAGAGATTCCCAGAAAGTC

  >165_primer_pair_2_product
  GTTCATGCTTGATTCTGAGCCGGGCGCTGACTGTCATGTGATTTGGTCACATGACCGACA
  CAACGGGCGGGGCAGCATCACGTGATAGTCTGGCGGGGGCTGTCCTACTGTGGCTGGATT
  CTAGTTGGAGGATCAGCCTACTCTTCTTCAGTTTCCCGGTTCCTCCAAATTTCTGGGCTC
  CTACTTGTTTCCACAGAGATGGATACTGTGGAGGTCCAGGAAGCAGAGAGATGGCTAAGG
  CTCATCAGGACCGTATGATCTCCCAAGTGTCCAGCTACTGAGTACCACAAGGTGATGGGT
  GGGAGGGTCCTCCCACGGAAGGATACCGCAGTCCCTAGGGGTTGCAAGCCCCACATGTTC
  CACTGGCTGCTAGAGCTACCTACTCAATCAGCCCTGGGCATCACCATCAGGTACTCGGCC
  AAAATGACCTCTCTGCTTCCAGTCCTCAGTTCTGGTCAGCACCAGACAGGCCCATAATTA
  CAGAGCCAGGGAAACTGGAACATTTGTCTCCCCTTAGACAGTGGCAGCAGGAAGGTGGGG
  GGTTGTTGCAGAGGAACAGTGTCTCTGAGAGAGGACCTTGGACTTTCTGGGAATCTCTGA
  GC
  ============================================================

Primers for ID: 166
Primer pair 1, product size : 719
  Primer left                            Primer right
  start: 25211842                        start: 25212540
  end  : 25211862                        end  : 25212560
  tm   : 57                              tm   : 57
  seq  : GTTCATGCTTGATTCTGAGCC           seq  : ATGAGACACTCTGTGCCAATG

  >166_primer_pair_1_product
  GTTCATGCTTGATTCTGAGCCGGGCGCTGACTGTCATGTGATTTGGTCACATGACCGACA
  CAACGGGCGGGGCAGCATCACGTGATAGTCTGGCGGGGGCTGTCCTACTGTGGCTGGATT
  CTAGTTGGAGGATCAGCCTACTCTTCTTCAGTTTCCCGGTTCCTCCAAATTTCTGGGCTC
  CTACTTGTTTCCACAGAGATGGATACTGTGGAGGTCCAGGAAGCAGAGAGATGGCTAAGG
  CTCATCAGGACCGTATGATCTCCCAAGTGTCCAGCTACTGAGTACCACAAGGTGATGGGT
  GGGAGGGTCCTCCCACGGAAGGATACCGCAGTCCCTAGGGGTTGCAAGCCCCACATGTTC
  CACTGGCTGCTAGAGCTACCTACTCAATCAGCCCTGGGCATCACCATCAGGTACTCGGCC
  AAAATGACCTCTCTGCTTCCAGTCCTCAGTTCTGGTCAGCACCAGACAGGCCCATAATTA
  CAGAGCCAGGGAAACTGGAACATTTGTCTCCCCTTAGACAGTGGCAGCAGGAAGGTGGGG
  GGTTGTTGCAGAGGAACAGTGTCTCTGAGAGAGGACCTTGGACTTTCTGGGAATCTCTGA
  GCTGCCCGGTTCTCCCCACTGCTGGCACTGTGCCCACAGCCCAAACAGAATGGGGGAGAT
  GGAGGGGCAGGGCTTCTGTGGGAAGCTGCCCTCCACCTCATTGGCACAGAGTGTCTCAT
  --------------------

Primer pair 2, product size : 602
  Primer left                            Primer right
  start: 25211842                        start: 25212422
  end  : 25211862                        end  : 25212443
  tm   : 57                              tm   : 61
  seq  : GTTCATGCTTGATTCTGAGCC           seq  : GCTCAGAGATTCCCAGAAAGTC

  >166_primer_pair_2_product
  GTTCATGCTTGATTCTGAGCCGGGCGCTGACTGTCATGTGATTTGGTCACATGACCGACA
  CAACGGGCGGGGCAGCATCACGTGATAGTCTGGCGGGGGCTGTCCTACTGTGGCTGGATT
  CTAGTTGGAGGATCAGCCTACTCTTCTTCAGTTTCCCGGTTCCTCCAAATTTCTGGGCTC
  CTACTTGTTTCCACAGAGATGGATACTGTGGAGGTCCAGGAAGCAGAGAGATGGCTAAGG
  CTCATCAGGACCGTATGATCTCCCAAGTGTCCAGCTACTGAGTACCACAAGGTGATGGGT
  GGGAGGGTCCTCCCACGGAAGGATACCGCAGTCCCTAGGGGTTGCAAGCCCCACATGTTC
  CACTGGCTGCTAGAGCTACCTACTCAATCAGCCCTGGGCATCACCATCAGGTACTCGGCC
  AAAATGACCTCTCTGCTTCCAGTCCTCAGTTCTGGTCAGCACCAGACAGGCCCATAATTA
  CAGAGCCAGGGAAACTGGAACATTTGTCTCCCCTTAGACAGTGGCAGCAGGAAGGTGGGG
  GGTTGTTGCAGAGGAACAGTGTCTCTGAGAGAGGACCTTGGACTTTCTGGGAATCTCTGA
  GC
  ============================================================

Primers for ID: 167
Primer pair 1, product size : 670
  Primer left                            Primer right
  start: 25209909                        start: 25210558
  end  : 25209929                        end  : 25210578
  tm   : 57                              tm   : 57
  seq  : GATGGCATGAAAGCAGATAGC           seq  : ACAACCATGATCTGTGCTCTG

  >167_primer_pair_1_product
  GATGGCATGAAAGCAGATAGCTACAAGGCTCTTGGACCAGGCTAGCACTGGGTCCTGCAC
  CCAGGGAGAGCCACCTCACCTTGACAGGGTTGGCAGGAAGGGGCCCTAGAAAGTCAGTAG
  GATACGGGTAGTCCATCATGGCGAGCACAGTAAATGCATTTCGGGCAAACCCAAAGAGCT
  GAGTCAGGTCCTTTGGGCTGGAAAGTGATTGACAGGTACCAAAGTTCTGGCTGATGGTGT
  CATAGGCTGGGAAGAGAGAGGCCAGGAGAAAAGGCTGAGGAAACTGCTGGCAAATGTGAA
  GGGCAAGAATGAATGCCCAAGGTGGGCAGCAGGTGAGGAAAGAGTCCCTCACCTCCCTGG
  AGGAACAAGTCTTTGATTTGCTGAAAGGCATCCCGCACAGCCTGGGCGCACTTGGGACTC
  TGGCCATAAAAGTCCTGGAGAAGAGACCAAGGTTGCTGCTGCCATTCTTGCACTGGCCTG
  GGGTACCCAAGTCCCCTCACTCACCGCTGTGACATCTCGGAAGAATTGGTAGGAGTCCCC
  AAGGCCTGCAACAGCTACAACAGGAGCGCTGGCTGCCAGTGCCCCAGCCACCAGGTGGGG
  GTACTTCATCCTCATGTAGGCACTCAGCATCCCCCCATAACTGGGAGTACAGAGCACAGA
  TCATGGTTGT
  --------------------

Primer pair 2, product size : 656
  Primer left                            Primer right
  start: 25209909                        start: 25210543
  end  : 25209929                        end  : 25210564
  tm   : 57                              tm   : 61
  seq  : GATGGCATGAAAGCAGATAGC           seq  : TGCTCTGTACTCCCAGTTATGG

  >167_primer_pair_2_product
  GATGGCATGAAAGCAGATAGCTACAAGGCTCTTGGACCAGGCTAGCACTGGGTCCTGCAC
  CCAGGGAGAGCCACCTCACCTTGACAGGGTTGGCAGGAAGGGGCCCTAGAAAGTCAGTAG
  GATACGGGTAGTCCATCATGGCGAGCACAGTAAATGCATTTCGGGCAAACCCAAAGAGCT
  GAGTCAGGTCCTTTGGGCTGGAAAGTGATTGACAGGTACCAAAGTTCTGGCTGATGGTGT
  CATAGGCTGGGAAGAGAGAGGCCAGGAGAAAAGGCTGAGGAAACTGCTGGCAAATGTGAA
  GGGCAAGAATGAATGCCCAAGGTGGGCAGCAGGTGAGGAAAGAGTCCCTCACCTCCCTGG
  AGGAACAAGTCTTTGATTTGCTGAAAGGCATCCCGCACAGCCTGGGCGCACTTGGGACTC
  TGGCCATAAAAGTCCTGGAGAAGAGACCAAGGTTGCTGCTGCCATTCTTGCACTGGCCTG
  GGGTACCCAAGTCCCCTCACTCACCGCTGTGACATCTCGGAAGAATTGGTAGGAGTCCCC
  AAGGCCTGCAACAGCTACAACAGGAGCGCTGGCTGCCAGTGCCCCAGCCACCAGGTGGGG
  GTACTTCATCCTCATGTAGGCACTCAGCATCCCCCCATAACTGGGAGTACAGAGCA
  ============================================================

Primers for ID: 168
Primer pair 1, product size : 670
  Primer left                            Primer right
  start: 25209909                        start: 25210558
  end  : 25209929                        end  : 25210578
  tm   : 57                              tm   : 57
  seq  : GATGGCATGAAAGCAGATAGC           seq  : ACAACCATGATCTGTGCTCTG

  >168_primer_pair_1_product
  GATGGCATGAAAGCAGATAGCTACAAGGCTCTTGGACCAGGCTAGCACTGGGTCCTGCAC
  CCAGGGAGAGCCACCTCACCTTGACAGGGTTGGCAGGAAGGGGCCCTAGAAAGTCAGTAG
  GATACGGGTAGTCCATCATGGCGAGCACAGTAAATGCATTTCGGGCAAACCCAAAGAGCT
  GAGTCAGGTCCTTTGGGCTGGAAAGTGATTGACAGGTACCAAAGTTCTGGCTGATGGTGT
  CATAGGCTGGGAAGAGAGAGGCCAGGAGAAAAGGCTGAGGAAACTGCTGGCAAATGTGAA
  GGGCAAGAATGAATGCCCAAGGTGGGCAGCAGGTGAGGAAAGAGTCCCTCACCTCCCTGG
  AGGAACAAGTCTTTGATTTGCTGAAAGGCATCCCGCACAGCCTGGGCGCACTTGGGACTC
  TGGCCATAAAAGTCCTGGAGAAGAGACCAAGGTTGCTGCTGCCATTCTTGCACTGGCCTG
  GGGTACCCAAGTCCCCTCACTCACCGCTGTGACATCTCGGAAGAATTGGTAGGAGTCCCC
  AAGGCCTGCAACAGCTACAACAGGAGCGCTGGCTGCCAGTGCCCCAGCCACCAGGTGGGG
  GTACTTCATCCTCATGTAGGCACTCAGCATCCCCCCATAACTGGGAGTACAGAGCACAGA
  TCATGGTTGT
  --------------------

Primer pair 2, product size : 656
  Primer left                            Primer right
  start: 25209909                        start: 25210543
  end  : 25209929                        end  : 25210564
  tm   : 57                              tm   : 61
  seq  : GATGGCATGAAAGCAGATAGC           seq  : TGCTCTGTACTCCCAGTTATGG

  >168_primer_pair_2_product
  GATGGCATGAAAGCAGATAGCTACAAGGCTCTTGGACCAGGCTAGCACTGGGTCCTGCAC
  CCAGGGAGAGCCACCTCACCTTGACAGGGTTGGCAGGAAGGGGCCCTAGAAAGTCAGTAG
  GATACGGGTAGTCCATCATGGCGAGCACAGTAAATGCATTTCGGGCAAACCCAAAGAGCT
  GAGTCAGGTCCTTTGGGCTGGAAAGTGATTGACAGGTACCAAAGTTCTGGCTGATGGTGT
  CATAGGCTGGGAAGAGAGAGGCCAGGAGAAAAGGCTGAGGAAACTGCTGGCAAATGTGAA
  GGGCAAGAATGAATGCCCAAGGTGGGCAGCAGGTGAGGAAAGAGTCCCTCACCTCCCTGG
  AGGAACAAGTCTTTGATTTGCTGAAAGGCATCCCGCACAGCCTGGGCGCACTTGGGACTC
  TGGCCATAAAAGTCCTGGAGAAGAGACCAAGGTTGCTGCTGCCATTCTTGCACTGGCCTG
  GGGTACCCAAGTCCCCTCACTCACCGCTGTGACATCTCGGAAGAATTGGTAGGAGTCCCC
  AAGGCCTGCAACAGCTACAACAGGAGCGCTGGCTGCCAGTGCCCCAGCCACCAGGTGGGG
  GTACTTCATCCTCATGTAGGCACTCAGCATCCCCCCATAACTGGGAGTACAGAGCA
  ============================================================

Primers for ID: 169
Primer pair 1, product size : 670
  Primer left                            Primer right
  start: 25209909                        start: 25210558
  end  : 25209929                        end  : 25210578
  tm   : 57                              tm   : 57
  seq  : GATGGCATGAAAGCAGATAGC           seq  : ACAACCATGATCTGTGCTCTG

  >169_primer_pair_1_product
  GATGGCATGAAAGCAGATAGCTACAAGGCTCTTGGACCAGGCTAGCACTGGGTCCTGCAC
  CCAGGGAGAGCCACCTCACCTTGACAGGGTTGGCAGGAAGGGGCCCTAGAAAGTCAGTAG
  GATACGGGTAGTCCATCATGGCGAGCACAGTAAATGCATTTCGGGCAAACCCAAAGAGCT
  GAGTCAGGTCCTTTGGGCTGGAAAGTGATTGACAGGTACCAAAGTTCTGGCTGATGGTGT
  CATAGGCTGGGAAGAGAGAGGCCAGGAGAAAAGGCTGAGGAAACTGCTGGCAAATGTGAA
  GGGCAAGAATGAATGCCCAAGGTGGGCAGCAGGTGAGGAAAGAGTCCCTCACCTCCCTGG
  AGGAACAAGTCTTTGATTTGCTGAAAGGCATCCCGCACAGCCTGGGCGCACTTGGGACTC
  TGGCCATAAAAGTCCTGGAGAAGAGACCAAGGTTGCTGCTGCCATTCTTGCACTGGCCTG
  GGGTACCCAAGTCCCCTCACTCACCGCTGTGACATCTCGGAAGAATTGGTAGGAGTCCCC
  AAGGCCTGCAACAGCTACAACAGGAGCGCTGGCTGCCAGTGCCCCAGCCACCAGGTGGGG
  GTACTTCATCCTCATGTAGGCACTCAGCATCCCCCCATAACTGGGAGTACAGAGCACAGA
  TCATGGTTGT
  --------------------

Primer pair 2, product size : 782
  Primer left                            Primer right
  start: 25209909                        start: 25210670
  end  : 25209929                        end  : 25210690
  tm   : 57                              tm   : 57
  seq  : GATGGCATGAAAGCAGATAGC           seq  : AGTTTCAGGTCCTTCTTTCCC

  >169_primer_pair_2_product
  GATGGCATGAAAGCAGATAGCTACAAGGCTCTTGGACCAGGCTAGCACTGGGTCCTGCAC
  CCAGGGAGAGCCACCTCACCTTGACAGGGTTGGCAGGAAGGGGCCCTAGAAAGTCAGTAG
  GATACGGGTAGTCCATCATGGCGAGCACAGTAAATGCATTTCGGGCAAACCCAAAGAGCT
  GAGTCAGGTCCTTTGGGCTGGAAAGTGATTGACAGGTACCAAAGTTCTGGCTGATGGTGT
  CATAGGCTGGGAAGAGAGAGGCCAGGAGAAAAGGCTGAGGAAACTGCTGGCAAATGTGAA
  GGGCAAGAATGAATGCCCAAGGTGGGCAGCAGGTGAGGAAAGAGTCCCTCACCTCCCTGG
  AGGAACAAGTCTTTGATTTGCTGAAAGGCATCCCGCACAGCCTGGGCGCACTTGGGACTC
  TGGCCATAAAAGTCCTGGAGAAGAGACCAAGGTTGCTGCTGCCATTCTTGCACTGGCCTG
  GGGTACCCAAGTCCCCTCACTCACCGCTGTGACATCTCGGAAGAATTGGTAGGAGTCCCC
  AAGGCCTGCAACAGCTACAACAGGAGCGCTGGCTGCCAGTGCCCCAGCCACCAGGTGGGG
  GTACTTCATCCTCATGTAGGCACTCAGCATCCCCCCATAACTGGGAGTACAGAGCACAGA
  TCATGGTTGTGGGAAGCTGCCCACAACTCAGGCGAGCAGCCTCACTGTCCTCCAGGCTGA
  GGTGCTAGGCTGCTCTTTCCCTGCTCAGAACGCCCAAGGGTGGGAAAGAAGGACCTGAAA
  CT
  ============================================================

Primers for ID: 170
Primer pair 1, product size : 692
  Primer left                            Primer right
  start: 25210129                        start: 25210799
  end  : 25210149                        end  : 25210820
  tm   : 57                              tm   : 59
  seq  : AAAGTTCTGGCTGATGGTGTC           seq  : GGGACAATTCCTTCTGACTTTG

  >170_primer_pair_1_product
  AAAGTTCTGGCTGATGGTGTCATAGGCTGGGAAGAGAGAGGCCAGGAGAAAAGGCTGAGG
  AAACTGCTGGCAAATGTGAAGGGCAAGAATGAATGCCCAAGGTGGGCAGCAGGTGAGGAA
  AGAGTCCCTCACCTCCCTGGAGGAACAAGTCTTTGATTTGCTGAAAGGCATCCCGCACAG
  CCTGGGCGCACTTGGGACTCTGGCCATAAAAGTCCTGGAGAAGAGACCAAGGTTGCTGCT
  GCCATTCTTGCACTGGCCTGGGGTACCCAAGTCCCCTCACTCACCGCTGTGACATCTCGG
  AAGAATTGGTAGGAGTCCCCAAGGCCTGCAACAGCTACAACAGGAGCGCTGGCTGCCAGT
  GCCCCAGCCACCAGGTGGGGGTACTTCATCCTCATGTAGGCACTCAGCATCCCCCCATAA
  CTGGGAGTACAGAGCACAGATCATGGTTGTGGGAAGCTGCCCACAACTCAGGCGAGCAGC
  CTCACTGTCCTCCAGGCTGAGGTGCTAGGCTGCTCTTTCCCTGCTCAGAACGCCCAAGGG
  TGGGAAAGAAGGACCTGAAACTGTCAGGCCCACACACCCTGATCCCAGGGCCAAGGCAGA
  TACAGCCTTCACTGGGAGAAGGCACCTGTGGGTGCCCTGCCCTGACCCAGCAATGAAGAC
  ATTGCAGAGACAAAGTCAGAAGGAATTGTCCC
  --------------------

Primer pair 2, product size : 661
  Primer left                            Primer right
  start: 25210129                        start: 25210770
  end  : 25210149                        end  : 25210789
  tm   : 57                              tm   : 55
  seq  : AAAGTTCTGGCTGATGGTGTC           seq  : TGTCTTCATTGCTGGGTCAG

  >170_primer_pair_2_product
  AAAGTTCTGGCTGATGGTGTCATAGGCTGGGAAGAGAGAGGCCAGGAGAAAAGGCTGAGG
  AAACTGCTGGCAAATGTGAAGGGCAAGAATGAATGCCCAAGGTGGGCAGCAGGTGAGGAA
  AGAGTCCCTCACCTCCCTGGAGGAACAAGTCTTTGATTTGCTGAAAGGCATCCCGCACAG
  CCTGGGCGCACTTGGGACTCTGGCCATAAAAGTCCTGGAGAAGAGACCAAGGTTGCTGCT
  GCCATTCTTGCACTGGCCTGGGGTACCCAAGTCCCCTCACTCACCGCTGTGACATCTCGG
  AAGAATTGGTAGGAGTCCCCAAGGCCTGCAACAGCTACAACAGGAGCGCTGGCTGCCAGT
  GCCCCAGCCACCAGGTGGGGGTACTTCATCCTCATGTAGGCACTCAGCATCCCCCCATAA
  CTGGGAGTACAGAGCACAGATCATGGTTGTGGGAAGCTGCCCACAACTCAGGCGAGCAGC
  CTCACTGTCCTCCAGGCTGAGGTGCTAGGCTGCTCTTTCCCTGCTCAGAACGCCCAAGGG
  TGGGAAAGAAGGACCTGAAACTGTCAGGCCCACACACCCTGATCCCAGGGCCAAGGCAGA
  TACAGCCTTCACTGGGAGAAGGCACCTGTGGGTGCCCTGCCCTGACCCAGCAATGAAGAC
  A
  ============================================================

Primers for ID: 171
Primer pair 1, product size : 692
  Primer left                            Primer right
  start: 25210129                        start: 25210799
  end  : 25210149                        end  : 25210820
  tm   : 57                              tm   : 59
  seq  : AAAGTTCTGGCTGATGGTGTC           seq  : GGGACAATTCCTTCTGACTTTG

  >171_primer_pair_1_product
  AAAGTTCTGGCTGATGGTGTCATAGGCTGGGAAGAGAGAGGCCAGGAGAAAAGGCTGAGG
  AAACTGCTGGCAAATGTGAAGGGCAAGAATGAATGCCCAAGGTGGGCAGCAGGTGAGGAA
  AGAGTCCCTCACCTCCCTGGAGGAACAAGTCTTTGATTTGCTGAAAGGCATCCCGCACAG
  CCTGGGCGCACTTGGGACTCTGGCCATAAAAGTCCTGGAGAAGAGACCAAGGTTGCTGCT
  GCCATTCTTGCACTGGCCTGGGGTACCCAAGTCCCCTCACTCACCGCTGTGACATCTCGG
  AAGAATTGGTAGGAGTCCCCAAGGCCTGCAACAGCTACAACAGGAGCGCTGGCTGCCAGT
  GCCCCAGCCACCAGGTGGGGGTACTTCATCCTCATGTAGGCACTCAGCATCCCCCCATAA
  CTGGGAGTACAGAGCACAGATCATGGTTGTGGGAAGCTGCCCACAACTCAGGCGAGCAGC
  CTCACTGTCCTCCAGGCTGAGGTGCTAGGCTGCTCTTTCCCTGCTCAGAACGCCCAAGGG
  TGGGAAAGAAGGACCTGAAACTGTCAGGCCCACACACCCTGATCCCAGGGCCAAGGCAGA
  TACAGCCTTCACTGGGAGAAGGCACCTGTGGGTGCCCTGCCCTGACCCAGCAATGAAGAC
  ATTGCAGAGACAAAGTCAGAAGGAATTGTCCC
  --------------------

Primer pair 2, product size : 661
  Primer left                            Primer right
  start: 25210129                        start: 25210770
  end  : 25210149                        end  : 25210789
  tm   : 57                              tm   : 55
  seq  : AAAGTTCTGGCTGATGGTGTC           seq  : TGTCTTCATTGCTGGGTCAG

  >171_primer_pair_2_product
  AAAGTTCTGGCTGATGGTGTCATAGGCTGGGAAGAGAGAGGCCAGGAGAAAAGGCTGAGG
  AAACTGCTGGCAAATGTGAAGGGCAAGAATGAATGCCCAAGGTGGGCAGCAGGTGAGGAA
  AGAGTCCCTCACCTCCCTGGAGGAACAAGTCTTTGATTTGCTGAAAGGCATCCCGCACAG
  CCTGGGCGCACTTGGGACTCTGGCCATAAAAGTCCTGGAGAAGAGACCAAGGTTGCTGCT
  GCCATTCTTGCACTGGCCTGGGGTACCCAAGTCCCCTCACTCACCGCTGTGACATCTCGG
  AAGAATTGGTAGGAGTCCCCAAGGCCTGCAACAGCTACAACAGGAGCGCTGGCTGCCAGT
  GCCCCAGCCACCAGGTGGGGGTACTTCATCCTCATGTAGGCACTCAGCATCCCCCCATAA
  CTGGGAGTACAGAGCACAGATCATGGTTGTGGGAAGCTGCCCACAACTCAGGCGAGCAGC
  CTCACTGTCCTCCAGGCTGAGGTGCTAGGCTGCTCTTTCCCTGCTCAGAACGCCCAAGGG
  TGGGAAAGAAGGACCTGAAACTGTCAGGCCCACACACCCTGATCCCAGGGCCAAGGCAGA
  TACAGCCTTCACTGGGAGAAGGCACCTGTGGGTGCCCTGCCCTGACCCAGCAATGAAGAC
  A
  ============================================================

Primers for ID: 172
Primer pair 1, product size : 800
  Primer left                            Primer right
  start: 25210172                        start: 25210951
  end  : 25210192                        end  : 25210971
  tm   : 57                              tm   : 57
  seq  : AGGAGAAAAGGCTGAGGAAAC           seq  : TATACAAGGAGGGCCAAAGAG

  >172_primer_pair_1_product
  AGGAGAAAAGGCTGAGGAAACTGCTGGCAAATGTGAAGGGCAAGAATGAATGCCCAAGGT
  GGGCAGCAGGTGAGGAAAGAGTCCCTCACCTCCCTGGAGGAACAAGTCTTTGATTTGCTG
  AAAGGCATCCCGCACAGCCTGGGCGCACTTGGGACTCTGGCCATAAAAGTCCTGGAGAAG
  AGACCAAGGTTGCTGCTGCCATTCTTGCACTGGCCTGGGGTACCCAAGTCCCCTCACTCA
  CCGCTGTGACATCTCGGAAGAATTGGTAGGAGTCCCCAAGGCCTGCAACAGCTACAACAG
  GAGCGCTGGCTGCCAGTGCCCCAGCCACCAGGTGGGGGTACTTCATCCTCATGTAGGCAC
  TCAGCATCCCCCCATAACTGGGAGTACAGAGCACAGATCATGGTTGTGGGAAGCTGCCCA
  CAACTCAGGCGAGCAGCCTCACTGTCCTCCAGGCTGAGGTGCTAGGCTGCTCTTTCCCTG
  CTCAGAACGCCCAAGGGTGGGAAAGAAGGACCTGAAACTGTCAGGCCCACACACCCTGAT
  CCCAGGGCCAAGGCAGATACAGCCTTCACTGGGAGAAGGCACCTGTGGGTGCCCTGCCCT
  GACCCAGCAATGAAGACATTGCAGAGACAAAGTCAGAAGGAATTGTCCCACTAGTGGGAA
  CAACATAGCATACACTGCCTATGAGGTCCACTCAAGGAGGGCTTCCAGAAGGAGGTAAAG
  CTAGACCCCGCCCTTCCACATGTGGGGTAGGCATAGGATGTTGAGACTGTAAGAGACATC
  TCTTTGGCCCTCCTTGTATA
  --------------------

Primer pair 2, product size : 695
  Primer left                            Primer right
  start: 25210277                        start: 25210951
  end  : 25210297                        end  : 25210971
  tm   : 55                              tm   : 57
  seq  : GTCTTTGATTTGCTGAAAGGC           seq  : TATACAAGGAGGGCCAAAGAG

  >172_primer_pair_2_product
  GTCTTTGATTTGCTGAAAGGCATCCCGCACAGCCTGGGCGCACTTGGGACTCTGGCCATA
  AAAGTCCTGGAGAAGAGACCAAGGTTGCTGCTGCCATTCTTGCACTGGCCTGGGGTACCC
  AAGTCCCCTCACTCACCGCTGTGACATCTCGGAAGAATTGGTAGGAGTCCCCAAGGCCTG
  CAACAGCTACAACAGGAGCGCTGGCTGCCAGTGCCCCAGCCACCAGGTGGGGGTACTTCA
  TCCTCATGTAGGCACTCAGCATCCCCCCATAACTGGGAGTACAGAGCACAGATCATGGTT
  GTGGGAAGCTGCCCACAACTCAGGCGAGCAGCCTCACTGTCCTCCAGGCTGAGGTGCTAG
  GCTGCTCTTTCCCTGCTCAGAACGCCCAAGGGTGGGAAAGAAGGACCTGAAACTGTCAGG
  CCCACACACCCTGATCCCAGGGCCAAGGCAGATACAGCCTTCACTGGGAGAAGGCACCTG
  TGGGTGCCCTGCCCTGACCCAGCAATGAAGACATTGCAGAGACAAAGTCAGAAGGAATTG
  TCCCACTAGTGGGAACAACATAGCATACACTGCCTATGAGGTCCACTCAAGGAGGGCTTC
  CAGAAGGAGGTAAAGCTAGACCCCGCCCTTCCACATGTGGGGTAGGCATAGGATGTTGAG
  ACTGTAAGAGACATCTCTTTGGCCCTCCTTGTATA
  ============================================================

Primers for ID: 173
Primer pair 1, product size : 775
  Primer left                            Primer right
  start: 25210277                        start: 25211030
  end  : 25210297                        end  : 25211051
  tm   : 55                              tm   : 61
  seq  : GTCTTTGATTTGCTGAAAGGC           seq  : AGATCTGGACCCATGAGAGAAC

  >173_primer_pair_1_product
  GTCTTTGATTTGCTGAAAGGCATCCCGCACAGCCTGGGCGCACTTGGGACTCTGGCCATA
  AAAGTCCTGGAGAAGAGACCAAGGTTGCTGCTGCCATTCTTGCACTGGCCTGGGGTACCC
  AAGTCCCCTCACTCACCGCTGTGACATCTCGGAAGAATTGGTAGGAGTCCCCAAGGCCTG
  CAACAGCTACAACAGGAGCGCTGGCTGCCAGTGCCCCAGCCACCAGGTGGGGGTACTTCA
  TCCTCATGTAGGCACTCAGCATCCCCCCATAACTGGGAGTACAGAGCACAGATCATGGTT
  GTGGGAAGCTGCCCACAACTCAGGCGAGCAGCCTCACTGTCCTCCAGGCTGAGGTGCTAG
  GCTGCTCTTTCCCTGCTCAGAACGCCCAAGGGTGGGAAAGAAGGACCTGAAACTGTCAGG
  CCCACACACCCTGATCCCAGGGCCAAGGCAGATACAGCCTTCACTGGGAGAAGGCACCTG
  TGGGTGCCCTGCCCTGACCCAGCAATGAAGACATTGCAGAGACAAAGTCAGAAGGAATTG
  TCCCACTAGTGGGAACAACATAGCATACACTGCCTATGAGGTCCACTCAAGGAGGGCTTC
  CAGAAGGAGGTAAAGCTAGACCCCGCCCTTCCACATGTGGGGTAGGCATAGGATGTTGAG
  ACTGTAAGAGACATCTCTTTGGCCCTCCTTGTATAGGGTGTCAATCGGCACAACAGGGTG
  GAGCCTTAGAGTAGGGTAAGATTAGGACTCTAGGTTCTCTCATGGGTCCAGATCT
  --------------------

Primer pair 2, product size : 776
  Primer left                            Primer right
  start: 25210276                        start: 25211030
  end  : 25210297                        end  : 25211051
  tm   : 57                              tm   : 61
  seq  : AGTCTTTGATTTGCTGAAAGGC          seq  : AGATCTGGACCCATGAGAGAAC

  >173_primer_pair_2_product
  AGTCTTTGATTTGCTGAAAGGCATCCCGCACAGCCTGGGCGCACTTGGGACTCTGGCCAT
  AAAAGTCCTGGAGAAGAGACCAAGGTTGCTGCTGCCATTCTTGCACTGGCCTGGGGTACC
  CAAGTCCCCTCACTCACCGCTGTGACATCTCGGAAGAATTGGTAGGAGTCCCCAAGGCCT
  GCAACAGCTACAACAGGAGCGCTGGCTGCCAGTGCCCCAGCCACCAGGTGGGGGTACTTC
  ATCCTCATGTAGGCACTCAGCATCCCCCCATAACTGGGAGTACAGAGCACAGATCATGGT
  TGTGGGAAGCTGCCCACAACTCAGGCGAGCAGCCTCACTGTCCTCCAGGCTGAGGTGCTA
  GGCTGCTCTTTCCCTGCTCAGAACGCCCAAGGGTGGGAAAGAAGGACCTGAAACTGTCAG
  GCCCACACACCCTGATCCCAGGGCCAAGGCAGATACAGCCTTCACTGGGAGAAGGCACCT
  GTGGGTGCCCTGCCCTGACCCAGCAATGAAGACATTGCAGAGACAAAGTCAGAAGGAATT
  GTCCCACTAGTGGGAACAACATAGCATACACTGCCTATGAGGTCCACTCAAGGAGGGCTT
  CCAGAAGGAGGTAAAGCTAGACCCCGCCCTTCCACATGTGGGGTAGGCATAGGATGTTGA
  GACTGTAAGAGACATCTCTTTGGCCCTCCTTGTATAGGGTGTCAATCGGCACAACAGGGT
  GGAGCCTTAGAGTAGGGTAAGATTAGGACTCTAGGTTCTCTCATGGGTCCAGATCT
  ============================================================

Primers for ID: 184
Primer pair 1, product size : 671
  Primer left                            Primer right
  start: 25211332                        start: 25211982
  end  : 25211351                        end  : 25212002
  tm   : 55                              tm   : 57
  seq  : ACGTGCTCAGCAAAGACAAG            seq  : AACCGGGAAACTGAAGAAGAG

  >184_primer_pair_1_product
  ACGTGCTCAGCAAAGACAAGCAGGGCCTCCTGCTGGGCTGCCAGTTCCACCATGAAGCCA
  GAGTTGTTAGCGAAGGACCAGATATCCCCCTCATTCCCTGTGTAGAAAAAGATGGGCCCT
  TCGCCCATCTTCCAGAACTTATCTGTTGGAAGTAAATGAGTTTCCATAAGGCCAGGGAAA
  CGCAGGTAGGAACCCATGCGGTCGAGCCAGCACTCACCTGACACTAGGAACCGCTGGCCA
  AAGGTTTTGTTGCCGAAACTCTCAAAGTTGAAATGGTCCATGTATTGCTCAAAATAATTC
  TCATGAAAGTCAGGGTCTAGAACTCTGTCGGCTGAGGGCAGGTGCAGAGACTCAGGAGCT
  GGTTGGGATCATCAGGGATCTAGGCGGGTCAGGAGGAAGGGCAGCCAGTCTGTACTCACC
  TCTGGCCTGGAGGTTGCACAGTCCCAGTGACAGCAGCAGGACCAGGATCCAGGAGGGGAC
  ACCATGGTCCACAGGGTAACAAGGATGGAAGTTCATGCTTGATTCTGAGCCGGGCGCTGA
  CTGTCATGTGATTTGGTCACATGACCGACACAACGGGCGGGGCAGCATCACGTGATAGTC
  TGGCGGGGGCTGTCCTACTGTGGCTGGATTCTAGTTGGAGGATCAGCCTACTCTTCTTCA
  GTTTCCCGGTT
  --------------------

Primer pair 2, product size : 777
  Primer left                            Primer right
  start: 25211226                        start: 25211982
  end  : 25211245                        end  : 25212002
  tm   : 55                              tm   : 57
  seq  : ATTTCCCATAGTACCGCTGC            seq  : AACCGGGAAACTGAAGAAGAG

  >184_primer_pair_2_product
  ATTTCCCATAGTACCGCTGCAGAAAGCAGGAAGGGATGGCTAATCCACTCCTCGGTGCTC
  CCCACCTCCTTCAACTCAGGGACTGCCAGGAACTGTACAGGTACCCACGTGCTCAGCAAA
  GACAAGCAGGGCCTCCTGCTGGGCTGCCAGTTCCACCATGAAGCCAGAGTTGTTAGCGAA
  GGACCAGATATCCCCCTCATTCCCTGTGTAGAAAAAGATGGGCCCTTCGCCCATCTTCCA
  GAACTTATCTGTTGGAAGTAAATGAGTTTCCATAAGGCCAGGGAAACGCAGGTAGGAACC
  CATGCGGTCGAGCCAGCACTCACCTGACACTAGGAACCGCTGGCCAAAGGTTTTGTTGCC
  GAAACTCTCAAAGTTGAAATGGTCCATGTATTGCTCAAAATAATTCTCATGAAAGTCAGG
  GTCTAGAACTCTGTCGGCTGAGGGCAGGTGCAGAGACTCAGGAGCTGGTTGGGATCATCA
  GGGATCTAGGCGGGTCAGGAGGAAGGGCAGCCAGTCTGTACTCACCTCTGGCCTGGAGGT
  TGCACAGTCCCAGTGACAGCAGCAGGACCAGGATCCAGGAGGGGACACCATGGTCCACAG
  GGTAACAAGGATGGAAGTTCATGCTTGATTCTGAGCCGGGCGCTGACTGTCATGTGATTT
  GGTCACATGACCGACACAACGGGCGGGGCAGCATCACGTGATAGTCTGGCGGGGGCTGTC
  CTACTGTGGCTGGATTCTAGTTGGAGGATCAGCCTACTCTTCTTCAGTTTCCCGGTT
  ============================================================

Primers for ID: 185
Primer pair 1, product size : 715
  Primer left                            Primer right
  start: 25211383                        start: 25212076
  end  : 25211403                        end  : 25212097
  tm   : 57                              tm   : 61
  seq  : ATGAAGCCAGAGTTGTTAGCG           seq  : ATACGGTCCTGATGAGCCTTAG

  >185_primer_pair_1_product
  ATGAAGCCAGAGTTGTTAGCGAAGGACCAGATATCCCCCTCATTCCCTGTGTAGAAAAAG
  ATGGGCCCTTCGCCCATCTTCCAGAACTTATCTGTTGGAAGTAAATGAGTTTCCATAAGG
  CCAGGGAAACGCAGGTAGGAACCCATGCGGTCGAGCCAGCACTCACCTGACACTAGGAAC
  CGCTGGCCAAAGGTTTTGTTGCCGAAACTCTCAAAGTTGAAATGGTCCATGTATTGCTCA
  AAATAATTCTCATGAAAGTCAGGGTCTAGAACTCTGTCGGCTGAGGGCAGGTGCAGAGAC
  TCAGGAGCTGGTTGGGATCATCAGGGATCTAGGCGGGTCAGGAGGAAGGGCAGCCAGTCT
  GTACTCACCTCTGGCCTGGAGGTTGCACAGTCCCAGTGACAGCAGCAGGACCAGGATCCA
  GGAGGGGACACCATGGTCCACAGGGTAACAAGGATGGAAGTTCATGCTTGATTCTGAGCC
  GGGCGCTGACTGTCATGTGATTTGGTCACATGACCGACACAACGGGCGGGGCAGCATCAC
  GTGATAGTCTGGCGGGGGCTGTCCTACTGTGGCTGGATTCTAGTTGGAGGATCAGCCTAC
  TCTTCTTCAGTTTCCCGGTTCCTCCAAATTTCTGGGCTCCTACTTGTTTCCACAGAGATG
  GATACTGTGGAGGTCCAGGAAGCAGAGAGATGGCTAAGGCTCATCAGGACCGTAT
  --------------------

Primer pair 2, product size : 671
  Primer left                            Primer right
  start: 25211332                        start: 25211982
  end  : 25211351                        end  : 25212002
  tm   : 55                              tm   : 57
  seq  : ACGTGCTCAGCAAAGACAAG            seq  : AACCGGGAAACTGAAGAAGAG

  >185_primer_pair_2_product
  ACGTGCTCAGCAAAGACAAGCAGGGCCTCCTGCTGGGCTGCCAGTTCCACCATGAAGCCA
  GAGTTGTTAGCGAAGGACCAGATATCCCCCTCATTCCCTGTGTAGAAAAAGATGGGCCCT
  TCGCCCATCTTCCAGAACTTATCTGTTGGAAGTAAATGAGTTTCCATAAGGCCAGGGAAA
  CGCAGGTAGGAACCCATGCGGTCGAGCCAGCACTCACCTGACACTAGGAACCGCTGGCCA
  AAGGTTTTGTTGCCGAAACTCTCAAAGTTGAAATGGTCCATGTATTGCTCAAAATAATTC
  TCATGAAAGTCAGGGTCTAGAACTCTGTCGGCTGAGGGCAGGTGCAGAGACTCAGGAGCT
  GGTTGGGATCATCAGGGATCTAGGCGGGTCAGGAGGAAGGGCAGCCAGTCTGTACTCACC
  TCTGGCCTGGAGGTTGCACAGTCCCAGTGACAGCAGCAGGACCAGGATCCAGGAGGGGAC
  ACCATGGTCCACAGGGTAACAAGGATGGAAGTTCATGCTTGATTCTGAGCCGGGCGCTGA
  CTGTCATGTGATTTGGTCACATGACCGACACAACGGGCGGGGCAGCATCACGTGATAGTC
  TGGCGGGGGCTGTCCTACTGTGGCTGGATTCTAGTTGGAGGATCAGCCTACTCTTCTTCA
  GTTTCCCGGTT
  ============================================================

Primers for ID: 186
Primer pair 1, product size : 715
  Primer left                            Primer right
  start: 25211383                        start: 25212076
  end  : 25211403                        end  : 25212097
  tm   : 57                              tm   : 61
  seq  : ATGAAGCCAGAGTTGTTAGCG           seq  : ATACGGTCCTGATGAGCCTTAG

  >186_primer_pair_1_product
  ATGAAGCCAGAGTTGTTAGCGAAGGACCAGATATCCCCCTCATTCCCTGTGTAGAAAAAG
  ATGGGCCCTTCGCCCATCTTCCAGAACTTATCTGTTGGAAGTAAATGAGTTTCCATAAGG
  CCAGGGAAACGCAGGTAGGAACCCATGCGGTCGAGCCAGCACTCACCTGACACTAGGAAC
  CGCTGGCCAAAGGTTTTGTTGCCGAAACTCTCAAAGTTGAAATGGTCCATGTATTGCTCA
  AAATAATTCTCATGAAAGTCAGGGTCTAGAACTCTGTCGGCTGAGGGCAGGTGCAGAGAC
  TCAGGAGCTGGTTGGGATCATCAGGGATCTAGGCGGGTCAGGAGGAAGGGCAGCCAGTCT
  GTACTCACCTCTGGCCTGGAGGTTGCACAGTCCCAGTGACAGCAGCAGGACCAGGATCCA
  GGAGGGGACACCATGGTCCACAGGGTAACAAGGATGGAAGTTCATGCTTGATTCTGAGCC
  GGGCGCTGACTGTCATGTGATTTGGTCACATGACCGACACAACGGGCGGGGCAGCATCAC
  GTGATAGTCTGGCGGGGGCTGTCCTACTGTGGCTGGATTCTAGTTGGAGGATCAGCCTAC
  TCTTCTTCAGTTTCCCGGTTCCTCCAAATTTCTGGGCTCCTACTTGTTTCCACAGAGATG
  GATACTGTGGAGGTCCAGGAAGCAGAGAGATGGCTAAGGCTCATCAGGACCGTAT
  --------------------

Primer pair 2, product size : 707
  Primer left                            Primer right
  start: 25211391                        start: 25212076
  end  : 25211412                        end  : 25212097
  tm   : 61                              tm   : 61
  seq  : AGAGTTGTTAGCGAAGGACCAG          seq  : ATACGGTCCTGATGAGCCTTAG

  >186_primer_pair_2_product
  AGAGTTGTTAGCGAAGGACCAGATATCCCCCTCATTCCCTGTGTAGAAAAAGATGGGCCC
  TTCGCCCATCTTCCAGAACTTATCTGTTGGAAGTAAATGAGTTTCCATAAGGCCAGGGAA
  ACGCAGGTAGGAACCCATGCGGTCGAGCCAGCACTCACCTGACACTAGGAACCGCTGGCC
  AAAGGTTTTGTTGCCGAAACTCTCAAAGTTGAAATGGTCCATGTATTGCTCAAAATAATT
  CTCATGAAAGTCAGGGTCTAGAACTCTGTCGGCTGAGGGCAGGTGCAGAGACTCAGGAGC
  TGGTTGGGATCATCAGGGATCTAGGCGGGTCAGGAGGAAGGGCAGCCAGTCTGTACTCAC
  CTCTGGCCTGGAGGTTGCACAGTCCCAGTGACAGCAGCAGGACCAGGATCCAGGAGGGGA
  CACCATGGTCCACAGGGTAACAAGGATGGAAGTTCATGCTTGATTCTGAGCCGGGCGCTG
  ACTGTCATGTGATTTGGTCACATGACCGACACAACGGGCGGGGCAGCATCACGTGATAGT
  CTGGCGGGGGCTGTCCTACTGTGGCTGGATTCTAGTTGGAGGATCAGCCTACTCTTCTTC
  AGTTTCCCGGTTCCTCCAAATTTCTGGGCTCCTACTTGTTTCCACAGAGATGGATACTGT
  GGAGGTCCAGGAAGCAGAGAGATGGCTAAGGCTCATCAGGACCGTAT
  ============================================================

Primers for ID: 187
Primer pair 1, product size : 681
  Primer left                            Primer right
  start: 25211600                        start: 25212260
  end  : 25211621                        end  : 25212280
  tm   : 57                              tm   : 57
  seq  : TGAAATGGTCCATGTATTGCTC          seq  : GAAGCAGAGAGGTCATTTTGG

  >187_primer_pair_1_product
  TGAAATGGTCCATGTATTGCTCAAAATAATTCTCATGAAAGTCAGGGTCTAGAACTCTGT
  CGGCTGAGGGCAGGTGCAGAGACTCAGGAGCTGGTTGGGATCATCAGGGATCTAGGCGGG
  TCAGGAGGAAGGGCAGCCAGTCTGTACTCACCTCTGGCCTGGAGGTTGCACAGTCCCAGT
  GACAGCAGCAGGACCAGGATCCAGGAGGGGACACCATGGTCCACAGGGTAACAAGGATGG
  AAGTTCATGCTTGATTCTGAGCCGGGCGCTGACTGTCATGTGATTTGGTCACATGACCGA
  CACAACGGGCGGGGCAGCATCACGTGATAGTCTGGCGGGGGCTGTCCTACTGTGGCTGGA
  TTCTAGTTGGAGGATCAGCCTACTCTTCTTCAGTTTCCCGGTTCCTCCAAATTTCTGGGC
  TCCTACTTGTTTCCACAGAGATGGATACTGTGGAGGTCCAGGAAGCAGAGAGATGGCTAA
  GGCTCATCAGGACCGTATGATCTCCCAAGTGTCCAGCTACTGAGTACCACAAGGTGATGG
  GTGGGAGGGTCCTCCCACGGAAGGATACCGCAGTCCCTAGGGGTTGCAAGCCCCACATGT
  TCCACTGGCTGCTAGAGCTACCTACTCAATCAGCCCTGGGCATCACCATCAGGTACTCGG
  CCAAAATGACCTCTCTGCTTC
  --------------------

Primer pair 2, product size : 682
  Primer left                            Primer right
  start: 25211600                        start: 25212261
  end  : 25211621                        end  : 25212281
  tm   : 57                              tm   : 57
  seq  : TGAAATGGTCCATGTATTGCTC          seq  : GGAAGCAGAGAGGTCATTTTG

  >187_primer_pair_2_product
  TGAAATGGTCCATGTATTGCTCAAAATAATTCTCATGAAAGTCAGGGTCTAGAACTCTGT
  CGGCTGAGGGCAGGTGCAGAGACTCAGGAGCTGGTTGGGATCATCAGGGATCTAGGCGGG
  TCAGGAGGAAGGGCAGCCAGTCTGTACTCACCTCTGGCCTGGAGGTTGCACAGTCCCAGT
  GACAGCAGCAGGACCAGGATCCAGGAGGGGACACCATGGTCCACAGGGTAACAAGGATGG
  AAGTTCATGCTTGATTCTGAGCCGGGCGCTGACTGTCATGTGATTTGGTCACATGACCGA
  CACAACGGGCGGGGCAGCATCACGTGATAGTCTGGCGGGGGCTGTCCTACTGTGGCTGGA
  TTCTAGTTGGAGGATCAGCCTACTCTTCTTCAGTTTCCCGGTTCCTCCAAATTTCTGGGC
  TCCTACTTGTTTCCACAGAGATGGATACTGTGGAGGTCCAGGAAGCAGAGAGATGGCTAA
  GGCTCATCAGGACCGTATGATCTCCCAAGTGTCCAGCTACTGAGTACCACAAGGTGATGG
  GTGGGAGGGTCCTCCCACGGAAGGATACCGCAGTCCCTAGGGGTTGCAAGCCCCACATGT
  TCCACTGGCTGCTAGAGCTACCTACTCAATCAGCCCTGGGCATCACCATCAGGTACTCGG
  CCAAAATGACCTCTCTGCTTCC
  ============================================================

Primers for ID: 188
Primer pair 1, product size : 722
  Primer left                            Primer right
  start: 25211600                        start: 25212301
  end  : 25211621                        end  : 25212321
  tm   : 57                              tm   : 57
  seq  : TGAAATGGTCCATGTATTGCTC          seq  : TAATTATGGGCCTGTCTGGTG

  >188_primer_pair_1_product
  TGAAATGGTCCATGTATTGCTCAAAATAATTCTCATGAAAGTCAGGGTCTAGAACTCTGT
  CGGCTGAGGGCAGGTGCAGAGACTCAGGAGCTGGTTGGGATCATCAGGGATCTAGGCGGG
  TCAGGAGGAAGGGCAGCCAGTCTGTACTCACCTCTGGCCTGGAGGTTGCACAGTCCCAGT
  GACAGCAGCAGGACCAGGATCCAGGAGGGGACACCATGGTCCACAGGGTAACAAGGATGG
  AAGTTCATGCTTGATTCTGAGCCGGGCGCTGACTGTCATGTGATTTGGTCACATGACCGA
  CACAACGGGCGGGGCAGCATCACGTGATAGTCTGGCGGGGGCTGTCCTACTGTGGCTGGA
  TTCTAGTTGGAGGATCAGCCTACTCTTCTTCAGTTTCCCGGTTCCTCCAAATTTCTGGGC
  TCCTACTTGTTTCCACAGAGATGGATACTGTGGAGGTCCAGGAAGCAGAGAGATGGCTAA
  GGCTCATCAGGACCGTATGATCTCCCAAGTGTCCAGCTACTGAGTACCACAAGGTGATGG
  GTGGGAGGGTCCTCCCACGGAAGGATACCGCAGTCCCTAGGGGTTGCAAGCCCCACATGT
  TCCACTGGCTGCTAGAGCTACCTACTCAATCAGCCCTGGGCATCACCATCAGGTACTCGG
  CCAAAATGACCTCTCTGCTTCCAGTCCTCAGTTCTGGTCAGCACCAGACAGGCCCATAAT
  TA
  --------------------

Primer pair 2, product size : 724
  Primer left                            Primer right
  start: 25211600                        start: 25212303
  end  : 25211621                        end  : 25212323
  tm   : 57                              tm   : 57
  seq  : TGAAATGGTCCATGTATTGCTC          seq  : TGTAATTATGGGCCTGTCTGG

  >188_primer_pair_2_product
  TGAAATGGTCCATGTATTGCTCAAAATAATTCTCATGAAAGTCAGGGTCTAGAACTCTGT
  CGGCTGAGGGCAGGTGCAGAGACTCAGGAGCTGGTTGGGATCATCAGGGATCTAGGCGGG
  TCAGGAGGAAGGGCAGCCAGTCTGTACTCACCTCTGGCCTGGAGGTTGCACAGTCCCAGT
  GACAGCAGCAGGACCAGGATCCAGGAGGGGACACCATGGTCCACAGGGTAACAAGGATGG
  AAGTTCATGCTTGATTCTGAGCCGGGCGCTGACTGTCATGTGATTTGGTCACATGACCGA
  CACAACGGGCGGGGCAGCATCACGTGATAGTCTGGCGGGGGCTGTCCTACTGTGGCTGGA
  TTCTAGTTGGAGGATCAGCCTACTCTTCTTCAGTTTCCCGGTTCCTCCAAATTTCTGGGC
  TCCTACTTGTTTCCACAGAGATGGATACTGTGGAGGTCCAGGAAGCAGAGAGATGGCTAA
  GGCTCATCAGGACCGTATGATCTCCCAAGTGTCCAGCTACTGAGTACCACAAGGTGATGG
  GTGGGAGGGTCCTCCCACGGAAGGATACCGCAGTCCCTAGGGGTTGCAAGCCCCACATGT
  TCCACTGGCTGCTAGAGCTACCTACTCAATCAGCCCTGGGCATCACCATCAGGTACTCGG
  CCAAAATGACCTCTCTGCTTCCAGTCCTCAGTTCTGGTCAGCACCAGACAGGCCCATAAT
  TACA
  ============================================================

Primers for ID: 189
Primer pair 1, product size : 695
  Primer left                            Primer right
  start: 25211627                        start: 25212301
  end  : 25211648                        end  : 25212321
  tm   : 57                              tm   : 57
  seq  : AATTCTCATGAAAGTCAGGGTC          seq  : TAATTATGGGCCTGTCTGGTG

  >189_primer_pair_1_product
  AATTCTCATGAAAGTCAGGGTCTAGAACTCTGTCGGCTGAGGGCAGGTGCAGAGACTCAG
  GAGCTGGTTGGGATCATCAGGGATCTAGGCGGGTCAGGAGGAAGGGCAGCCAGTCTGTAC
  TCACCTCTGGCCTGGAGGTTGCACAGTCCCAGTGACAGCAGCAGGACCAGGATCCAGGAG
  GGGACACCATGGTCCACAGGGTAACAAGGATGGAAGTTCATGCTTGATTCTGAGCCGGGC
  GCTGACTGTCATGTGATTTGGTCACATGACCGACACAACGGGCGGGGCAGCATCACGTGA
  TAGTCTGGCGGGGGCTGTCCTACTGTGGCTGGATTCTAGTTGGAGGATCAGCCTACTCTT
  CTTCAGTTTCCCGGTTCCTCCAAATTTCTGGGCTCCTACTTGTTTCCACAGAGATGGATA
  CTGTGGAGGTCCAGGAAGCAGAGAGATGGCTAAGGCTCATCAGGACCGTATGATCTCCCA
  AGTGTCCAGCTACTGAGTACCACAAGGTGATGGGTGGGAGGGTCCTCCCACGGAAGGATA
  CCGCAGTCCCTAGGGGTTGCAAGCCCCACATGTTCCACTGGCTGCTAGAGCTACCTACTC
  AATCAGCCCTGGGCATCACCATCAGGTACTCGGCCAAAATGACCTCTCTGCTTCCAGTCC
  TCAGTTCTGGTCAGCACCAGACAGGCCCATAATTA
  --------------------

Primer pair 2, product size : 697
  Primer left                            Primer right
  start: 25211627                        start: 25212303
  end  : 25211648                        end  : 25212323
  tm   : 57                              tm   : 57
  seq  : AATTCTCATGAAAGTCAGGGTC          seq  : TGTAATTATGGGCCTGTCTGG

  >189_primer_pair_2_product
  AATTCTCATGAAAGTCAGGGTCTAGAACTCTGTCGGCTGAGGGCAGGTGCAGAGACTCAG
  GAGCTGGTTGGGATCATCAGGGATCTAGGCGGGTCAGGAGGAAGGGCAGCCAGTCTGTAC
  TCACCTCTGGCCTGGAGGTTGCACAGTCCCAGTGACAGCAGCAGGACCAGGATCCAGGAG
  GGGACACCATGGTCCACAGGGTAACAAGGATGGAAGTTCATGCTTGATTCTGAGCCGGGC
  GCTGACTGTCATGTGATTTGGTCACATGACCGACACAACGGGCGGGGCAGCATCACGTGA
  TAGTCTGGCGGGGGCTGTCCTACTGTGGCTGGATTCTAGTTGGAGGATCAGCCTACTCTT
  CTTCAGTTTCCCGGTTCCTCCAAATTTCTGGGCTCCTACTTGTTTCCACAGAGATGGATA
  CTGTGGAGGTCCAGGAAGCAGAGAGATGGCTAAGGCTCATCAGGACCGTATGATCTCCCA
  AGTGTCCAGCTACTGAGTACCACAAGGTGATGGGTGGGAGGGTCCTCCCACGGAAGGATA
  CCGCAGTCCCTAGGGGTTGCAAGCCCCACATGTTCCACTGGCTGCTAGAGCTACCTACTC
  AATCAGCCCTGGGCATCACCATCAGGTACTCGGCCAAAATGACCTCTCTGCTTCCAGTCC
  TCAGTTCTGGTCAGCACCAGACAGGCCCATAATTACA
  ============================================================

Primers for ID: 190

      ***NONE***

===============================

Primers for ID: 191
Primer pair 1, product size : 719
  Primer left                            Primer right
  start: 25211842                        start: 25212540
  end  : 25211862                        end  : 25212560
  tm   : 57                              tm   : 57
  seq  : GTTCATGCTTGATTCTGAGCC           seq  : ATGAGACACTCTGTGCCAATG

  >191_primer_pair_1_product
  GTTCATGCTTGATTCTGAGCCGGGCGCTGACTGTCATGTGATTTGGTCACATGACCGACA
  CAACGGGCGGGGCAGCATCACGTGATAGTCTGGCGGGGGCTGTCCTACTGTGGCTGGATT
  CTAGTTGGAGGATCAGCCTACTCTTCTTCAGTTTCCCGGTTCCTCCAAATTTCTGGGCTC
  CTACTTGTTTCCACAGAGATGGATACTGTGGAGGTCCAGGAAGCAGAGAGATGGCTAAGG
  CTCATCAGGACCGTATGATCTCCCAAGTGTCCAGCTACTGAGTACCACAAGGTGATGGGT
  GGGAGGGTCCTCCCACGGAAGGATACCGCAGTCCCTAGGGGTTGCAAGCCCCACATGTTC
  CACTGGCTGCTAGAGCTACCTACTCAATCAGCCCTGGGCATCACCATCAGGTACTCGGCC
  AAAATGACCTCTCTGCTTCCAGTCCTCAGTTCTGGTCAGCACCAGACAGGCCCATAATTA
  CAGAGCCAGGGAAACTGGAACATTTGTCTCCCCTTAGACAGTGGCAGCAGGAAGGTGGGG
  GGTTGTTGCAGAGGAACAGTGTCTCTGAGAGAGGACCTTGGACTTTCTGGGAATCTCTGA
  GCTGCCCGGTTCTCCCCACTGCTGGCACTGTGCCCACAGCCCAAACAGAATGGGGGAGAT
  GGAGGGGCAGGGCTTCTGTGGGAAGCTGCCCTCCACCTCATTGGCACAGAGTGTCTCAT
  --------------------

Primer pair 2, product size : 694
  Primer left                            Primer right
  start: 25211867                        start: 25212540
  end  : 25211887                        end  : 25212560
  tm   : 57                              tm   : 57
  seq  : GCTGACTGTCATGTGATTTGG           seq  : ATGAGACACTCTGTGCCAATG

  >191_primer_pair_2_product
  GCTGACTGTCATGTGATTTGGTCACATGACCGACACAACGGGCGGGGCAGCATCACGTGA
  TAGTCTGGCGGGGGCTGTCCTACTGTGGCTGGATTCTAGTTGGAGGATCAGCCTACTCTT
  CTTCAGTTTCCCGGTTCCTCCAAATTTCTGGGCTCCTACTTGTTTCCACAGAGATGGATA
  CTGTGGAGGTCCAGGAAGCAGAGAGATGGCTAAGGCTCATCAGGACCGTATGATCTCCCA
  AGTGTCCAGCTACTGAGTACCACAAGGTGATGGGTGGGAGGGTCCTCCCACGGAAGGATA
  CCGCAGTCCCTAGGGGTTGCAAGCCCCACATGTTCCACTGGCTGCTAGAGCTACCTACTC
  AATCAGCCCTGGGCATCACCATCAGGTACTCGGCCAAAATGACCTCTCTGCTTCCAGTCC
  TCAGTTCTGGTCAGCACCAGACAGGCCCATAATTACAGAGCCAGGGAAACTGGAACATTT
  GTCTCCCCTTAGACAGTGGCAGCAGGAAGGTGGGGGGTTGTTGCAGAGGAACAGTGTCTC
  TGAGAGAGGACCTTGGACTTTCTGGGAATCTCTGAGCTGCCCGGTTCTCCCCACTGCTGG
  CACTGTGCCCACAGCCCAAACAGAATGGGGGAGATGGAGGGGCAGGGCTTCTGTGGGAAG
  CTGCCCTCCACCTCATTGGCACAGAGTGTCTCAT
  ============================================================

Primers for ID: 192
Primer pair 1, product size : 694
  Primer left                            Primer right
  start: 25211867                        start: 25212540
  end  : 25211887                        end  : 25212560
  tm   : 57                              tm   : 57
  seq  : GCTGACTGTCATGTGATTTGG           seq  : ATGAGACACTCTGTGCCAATG

  >192_primer_pair_1_product
  GCTGACTGTCATGTGATTTGGTCACATGACCGACACAACGGGCGGGGCAGCATCACGTGA
  TAGTCTGGCGGGGGCTGTCCTACTGTGGCTGGATTCTAGTTGGAGGATCAGCCTACTCTT
  CTTCAGTTTCCCGGTTCCTCCAAATTTCTGGGCTCCTACTTGTTTCCACAGAGATGGATA
  CTGTGGAGGTCCAGGAAGCAGAGAGATGGCTAAGGCTCATCAGGACCGTATGATCTCCCA
  AGTGTCCAGCTACTGAGTACCACAAGGTGATGGGTGGGAGGGTCCTCCCACGGAAGGATA
  CCGCAGTCCCTAGGGGTTGCAAGCCCCACATGTTCCACTGGCTGCTAGAGCTACCTACTC
  AATCAGCCCTGGGCATCACCATCAGGTACTCGGCCAAAATGACCTCTCTGCTTCCAGTCC
  TCAGTTCTGGTCAGCACCAGACAGGCCCATAATTACAGAGCCAGGGAAACTGGAACATTT
  GTCTCCCCTTAGACAGTGGCAGCAGGAAGGTGGGGGGTTGTTGCAGAGGAACAGTGTCTC
  TGAGAGAGGACCTTGGACTTTCTGGGAATCTCTGAGCTGCCCGGTTCTCCCCACTGCTGG
  CACTGTGCCCACAGCCCAAACAGAATGGGGGAGATGGAGGGGCAGGGCTTCTGTGGGAAG
  CTGCCCTCCACCTCATTGGCACAGAGTGTCTCAT
  --------------------

Primer pair 2, product size : 806
  Primer left                            Primer right
  start: 25211867                        start: 25212653
  end  : 25211887                        end  : 25212672
  tm   : 57                              tm   : 55
  seq  : GCTGACTGTCATGTGATTTGG           seq  : ATGGACATGTTCCACTCACG

  >192_primer_pair_2_product
  GCTGACTGTCATGTGATTTGGTCACATGACCGACACAACGGGCGGGGCAGCATCACGTGA
  TAGTCTGGCGGGGGCTGTCCTACTGTGGCTGGATTCTAGTTGGAGGATCAGCCTACTCTT
  CTTCAGTTTCCCGGTTCCTCCAAATTTCTGGGCTCCTACTTGTTTCCACAGAGATGGATA
  CTGTGGAGGTCCAGGAAGCAGAGAGATGGCTAAGGCTCATCAGGACCGTATGATCTCCCA
  AGTGTCCAGCTACTGAGTACCACAAGGTGATGGGTGGGAGGGTCCTCCCACGGAAGGATA
  CCGCAGTCCCTAGGGGTTGCAAGCCCCACATGTTCCACTGGCTGCTAGAGCTACCTACTC
  AATCAGCCCTGGGCATCACCATCAGGTACTCGGCCAAAATGACCTCTCTGCTTCCAGTCC
  TCAGTTCTGGTCAGCACCAGACAGGCCCATAATTACAGAGCCAGGGAAACTGGAACATTT
  GTCTCCCCTTAGACAGTGGCAGCAGGAAGGTGGGGGGTTGTTGCAGAGGAACAGTGTCTC
  TGAGAGAGGACCTTGGACTTTCTGGGAATCTCTGAGCTGCCCGGTTCTCCCCACTGCTGG
  CACTGTGCCCACAGCCCAAACAGAATGGGGGAGATGGAGGGGCAGGGCTTCTGTGGGAAG
  CTGCCCTCCACCTCATTGGCACAGAGTGTCTCATTGCAGAGAGAAAAAAGGACCAGTTTT
  CTCTCTGGCACCCAGGTCTGGAAGAGGAGTGACATCCACGGAAGTTGGTGACTTGGACTG
  GCTGGCCGTGAGTGGAACATGTCCAT
  ============================================================

Primers for ID: 193
Primer pair 1, product size : 612
  Primer left                            Primer right
  start: 25209826                        start: 25210417
  end  : 25209847                        end  : 25210437
  tm   : 61                              tm   : 57
  seq  : AGCAGAATATCTCAGTGGAGGC          seq  : CCAATTCTTCCGAGATGTCAC

  >193_primer_pair_1_product
  AGCAGAATATCTCAGTGGAGGCCCCTTTCACAGAGCGTGGGTCAGGGCTGCTAGCTTCCA
  GGACACAACAGCAGATAGTGTCTGATGGCATGAAAGCAGATAGCTACAAGGCTCTTGGAC
  CAGGCTAGCACTGGGTCCTGCACCCAGGGAGAGCCACCTCACCTTGACAGGGTTGGCAGG
  AAGGGGCCCTAGAAAGTCAGTAGGATACGGGTAGTCCATCATGGCGAGCACAGTAAATGC
  ATTTCGGGCAAACCCAAAGAGCTGAGTCAGGTCCTTTGGGCTGGAAAGTGATTGACAGGT
  ACCAAAGTTCTGGCTGATGGTGTCATAGGCTGGGAAGAGAGAGGCCAGGAGAAAAGGCTG
  AGGAAACTGCTGGCAAATGTGAAGGGCAAGAATGAATGCCCAAGGTGGGCAGCAGGTGAG
  GAAAGAGTCCCTCACCTCCCTGGAGGAACAAGTCTTTGATTTGCTGAAAGGCATCCCGCA
  CAGCCTGGGCGCACTTGGGACTCTGGCCATAAAAGTCCTGGAGAAGAGACCAAGGTTGCT
  GCTGCCATTCTTGCACTGGCCTGGGGTACCCAAGTCCCCTCACTCACCGCTGTGACATCT
  CGGAAGAATTGG
  --------------------

Primer pair 2, product size : 614
  Primer left                            Primer right
  start: 25209824                        start: 25210417
  end  : 25209845                        end  : 25210437
  tm   : 61                              tm   : 57
  seq  : GCAGCAGAATATCTCAGTGGAG          seq  : CCAATTCTTCCGAGATGTCAC

  >193_primer_pair_2_product
  GCAGCAGAATATCTCAGTGGAGGCCCCTTTCACAGAGCGTGGGTCAGGGCTGCTAGCTTC
  CAGGACACAACAGCAGATAGTGTCTGATGGCATGAAAGCAGATAGCTACAAGGCTCTTGG
  ACCAGGCTAGCACTGGGTCCTGCACCCAGGGAGAGCCACCTCACCTTGACAGGGTTGGCA
  GGAAGGGGCCCTAGAAAGTCAGTAGGATACGGGTAGTCCATCATGGCGAGCACAGTAAAT
  GCATTTCGGGCAAACCCAAAGAGCTGAGTCAGGTCCTTTGGGCTGGAAAGTGATTGACAG
  GTACCAAAGTTCTGGCTGATGGTGTCATAGGCTGGGAAGAGAGAGGCCAGGAGAAAAGGC
  TGAGGAAACTGCTGGCAAATGTGAAGGGCAAGAATGAATGCCCAAGGTGGGCAGCAGGTG
  AGGAAAGAGTCCCTCACCTCCCTGGAGGAACAAGTCTTTGATTTGCTGAAAGGCATCCCG
  CACAGCCTGGGCGCACTTGGGACTCTGGCCATAAAAGTCCTGGAGAAGAGACCAAGGTTG
  CTGCTGCCATTCTTGCACTGGCCTGGGGTACCCAAGTCCCCTCACTCACCGCTGTGACAT
  CTCGGAAGAATTGG
  ============================================================

Primers for ID: 194
Primer pair 1, product size : 670
  Primer left                            Primer right
  start: 25209909                        start: 25210558
  end  : 25209929                        end  : 25210578
  tm   : 57                              tm   : 57
  seq  : GATGGCATGAAAGCAGATAGC           seq  : ACAACCATGATCTGTGCTCTG

  >194_primer_pair_1_product
  GATGGCATGAAAGCAGATAGCTACAAGGCTCTTGGACCAGGCTAGCACTGGGTCCTGCAC
  CCAGGGAGAGCCACCTCACCTTGACAGGGTTGGCAGGAAGGGGCCCTAGAAAGTCAGTAG
  GATACGGGTAGTCCATCATGGCGAGCACAGTAAATGCATTTCGGGCAAACCCAAAGAGCT
  GAGTCAGGTCCTTTGGGCTGGAAAGTGATTGACAGGTACCAAAGTTCTGGCTGATGGTGT
  CATAGGCTGGGAAGAGAGAGGCCAGGAGAAAAGGCTGAGGAAACTGCTGGCAAATGTGAA
  GGGCAAGAATGAATGCCCAAGGTGGGCAGCAGGTGAGGAAAGAGTCCCTCACCTCCCTGG
  AGGAACAAGTCTTTGATTTGCTGAAAGGCATCCCGCACAGCCTGGGCGCACTTGGGACTC
  TGGCCATAAAAGTCCTGGAGAAGAGACCAAGGTTGCTGCTGCCATTCTTGCACTGGCCTG
  GGGTACCCAAGTCCCCTCACTCACCGCTGTGACATCTCGGAAGAATTGGTAGGAGTCCCC
  AAGGCCTGCAACAGCTACAACAGGAGCGCTGGCTGCCAGTGCCCCAGCCACCAGGTGGGG
  GTACTTCATCCTCATGTAGGCACTCAGCATCCCCCCATAACTGGGAGTACAGAGCACAGA
  TCATGGTTGT
  --------------------

Primer pair 2, product size : 612
  Primer left                            Primer right
  start: 25209826                        start: 25210417
  end  : 25209847                        end  : 25210437
  tm   : 61                              tm   : 57
  seq  : AGCAGAATATCTCAGTGGAGGC          seq  : CCAATTCTTCCGAGATGTCAC

  >194_primer_pair_2_product
  AGCAGAATATCTCAGTGGAGGCCCCTTTCACAGAGCGTGGGTCAGGGCTGCTAGCTTCCA
  GGACACAACAGCAGATAGTGTCTGATGGCATGAAAGCAGATAGCTACAAGGCTCTTGGAC
  CAGGCTAGCACTGGGTCCTGCACCCAGGGAGAGCCACCTCACCTTGACAGGGTTGGCAGG
  AAGGGGCCCTAGAAAGTCAGTAGGATACGGGTAGTCCATCATGGCGAGCACAGTAAATGC
  ATTTCGGGCAAACCCAAAGAGCTGAGTCAGGTCCTTTGGGCTGGAAAGTGATTGACAGGT
  ACCAAAGTTCTGGCTGATGGTGTCATAGGCTGGGAAGAGAGAGGCCAGGAGAAAAGGCTG
  AGGAAACTGCTGGCAAATGTGAAGGGCAAGAATGAATGCCCAAGGTGGGCAGCAGGTGAG
  GAAAGAGTCCCTCACCTCCCTGGAGGAACAAGTCTTTGATTTGCTGAAAGGCATCCCGCA
  CAGCCTGGGCGCACTTGGGACTCTGGCCATAAAAGTCCTGGAGAAGAGACCAAGGTTGCT
  GCTGCCATTCTTGCACTGGCCTGGGGTACCCAAGTCCCCTCACTCACCGCTGTGACATCT
  CGGAAGAATTGG
  ============================================================

Primers for ID: 195
Primer pair 1, product size : 670
  Primer left                            Primer right
  start: 25209909                        start: 25210558
  end  : 25209929                        end  : 25210578
  tm   : 57                              tm   : 57
  seq  : GATGGCATGAAAGCAGATAGC           seq  : ACAACCATGATCTGTGCTCTG

  >195_primer_pair_1_product
  GATGGCATGAAAGCAGATAGCTACAAGGCTCTTGGACCAGGCTAGCACTGGGTCCTGCAC
  CCAGGGAGAGCCACCTCACCTTGACAGGGTTGGCAGGAAGGGGCCCTAGAAAGTCAGTAG
  GATACGGGTAGTCCATCATGGCGAGCACAGTAAATGCATTTCGGGCAAACCCAAAGAGCT
  GAGTCAGGTCCTTTGGGCTGGAAAGTGATTGACAGGTACCAAAGTTCTGGCTGATGGTGT
  CATAGGCTGGGAAGAGAGAGGCCAGGAGAAAAGGCTGAGGAAACTGCTGGCAAATGTGAA
  GGGCAAGAATGAATGCCCAAGGTGGGCAGCAGGTGAGGAAAGAGTCCCTCACCTCCCTGG
  AGGAACAAGTCTTTGATTTGCTGAAAGGCATCCCGCACAGCCTGGGCGCACTTGGGACTC
  TGGCCATAAAAGTCCTGGAGAAGAGACCAAGGTTGCTGCTGCCATTCTTGCACTGGCCTG
  GGGTACCCAAGTCCCCTCACTCACCGCTGTGACATCTCGGAAGAATTGGTAGGAGTCCCC
  AAGGCCTGCAACAGCTACAACAGGAGCGCTGGCTGCCAGTGCCCCAGCCACCAGGTGGGG
  GTACTTCATCCTCATGTAGGCACTCAGCATCCCCCCATAACTGGGAGTACAGAGCACAGA
  TCATGGTTGT
  --------------------

Primer pair 2, product size : 782
  Primer left                            Primer right
  start: 25209909                        start: 25210670
  end  : 25209929                        end  : 25210690
  tm   : 57                              tm   : 57
  seq  : GATGGCATGAAAGCAGATAGC           seq  : AGTTTCAGGTCCTTCTTTCCC

  >195_primer_pair_2_product
  GATGGCATGAAAGCAGATAGCTACAAGGCTCTTGGACCAGGCTAGCACTGGGTCCTGCAC
  CCAGGGAGAGCCACCTCACCTTGACAGGGTTGGCAGGAAGGGGCCCTAGAAAGTCAGTAG
  GATACGGGTAGTCCATCATGGCGAGCACAGTAAATGCATTTCGGGCAAACCCAAAGAGCT
  GAGTCAGGTCCTTTGGGCTGGAAAGTGATTGACAGGTACCAAAGTTCTGGCTGATGGTGT
  CATAGGCTGGGAAGAGAGAGGCCAGGAGAAAAGGCTGAGGAAACTGCTGGCAAATGTGAA
  GGGCAAGAATGAATGCCCAAGGTGGGCAGCAGGTGAGGAAAGAGTCCCTCACCTCCCTGG
  AGGAACAAGTCTTTGATTTGCTGAAAGGCATCCCGCACAGCCTGGGCGCACTTGGGACTC
  TGGCCATAAAAGTCCTGGAGAAGAGACCAAGGTTGCTGCTGCCATTCTTGCACTGGCCTG
  GGGTACCCAAGTCCCCTCACTCACCGCTGTGACATCTCGGAAGAATTGGTAGGAGTCCCC
  AAGGCCTGCAACAGCTACAACAGGAGCGCTGGCTGCCAGTGCCCCAGCCACCAGGTGGGG
  GTACTTCATCCTCATGTAGGCACTCAGCATCCCCCCATAACTGGGAGTACAGAGCACAGA
  TCATGGTTGTGGGAAGCTGCCCACAACTCAGGCGAGCAGCCTCACTGTCCTCCAGGCTGA
  GGTGCTAGGCTGCTCTTTCCCTGCTCAGAACGCCCAAGGGTGGGAAAGAAGGACCTGAAA
  CT
  ============================================================

Primers for ID: 196
Primer pair 1, product size : 617
  Primer left                            Primer right
  start: 25210074                        start: 25210670
  end  : 25210093                        end  : 25210690
  tm   : 55                              tm   : 57
  seq  : CAAACCCAAAGAGCTGAGTC            seq  : AGTTTCAGGTCCTTCTTTCCC

  >196_primer_pair_1_product
  CAAACCCAAAGAGCTGAGTCAGGTCCTTTGGGCTGGAAAGTGATTGACAGGTACCAAAGT
  TCTGGCTGATGGTGTCATAGGCTGGGAAGAGAGAGGCCAGGAGAAAAGGCTGAGGAAACT
  GCTGGCAAATGTGAAGGGCAAGAATGAATGCCCAAGGTGGGCAGCAGGTGAGGAAAGAGT
  CCCTCACCTCCCTGGAGGAACAAGTCTTTGATTTGCTGAAAGGCATCCCGCACAGCCTGG
  GCGCACTTGGGACTCTGGCCATAAAAGTCCTGGAGAAGAGACCAAGGTTGCTGCTGCCAT
  TCTTGCACTGGCCTGGGGTACCCAAGTCCCCTCACTCACCGCTGTGACATCTCGGAAGAA
  TTGGTAGGAGTCCCCAAGGCCTGCAACAGCTACAACAGGAGCGCTGGCTGCCAGTGCCCC
  AGCCACCAGGTGGGGGTACTTCATCCTCATGTAGGCACTCAGCATCCCCCCATAACTGGG
  AGTACAGAGCACAGATCATGGTTGTGGGAAGCTGCCCACAACTCAGGCGAGCAGCCTCAC
  TGTCCTCCAGGCTGAGGTGCTAGGCTGCTCTTTCCCTGCTCAGAACGCCCAAGGGTGGGA
  AAGAAGGACCTGAAACT
  --------------------

Primer pair 2, product size : 614
  Primer left                            Primer right
  start: 25210074                        start: 25210668
  end  : 25210093                        end  : 25210687
  tm   : 55                              tm   : 55
  seq  : CAAACCCAAAGAGCTGAGTC            seq  : TTCAGGTCCTTCTTTCCCAC

  >196_primer_pair_2_product
  CAAACCCAAAGAGCTGAGTCAGGTCCTTTGGGCTGGAAAGTGATTGACAGGTACCAAAGT
  TCTGGCTGATGGTGTCATAGGCTGGGAAGAGAGAGGCCAGGAGAAAAGGCTGAGGAAACT
  GCTGGCAAATGTGAAGGGCAAGAATGAATGCCCAAGGTGGGCAGCAGGTGAGGAAAGAGT
  CCCTCACCTCCCTGGAGGAACAAGTCTTTGATTTGCTGAAAGGCATCCCGCACAGCCTGG
  GCGCACTTGGGACTCTGGCCATAAAAGTCCTGGAGAAGAGACCAAGGTTGCTGCTGCCAT
  TCTTGCACTGGCCTGGGGTACCCAAGTCCCCTCACTCACCGCTGTGACATCTCGGAAGAA
  TTGGTAGGAGTCCCCAAGGCCTGCAACAGCTACAACAGGAGCGCTGGCTGCCAGTGCCCC
  AGCCACCAGGTGGGGGTACTTCATCCTCATGTAGGCACTCAGCATCCCCCCATAACTGGG
  AGTACAGAGCACAGATCATGGTTGTGGGAAGCTGCCCACAACTCAGGCGAGCAGCCTCAC
  TGTCCTCCAGGCTGAGGTGCTAGGCTGCTCTTTCCCTGCTCAGAACGCCCAAGGGTGGGA
  AAGAAGGACCTGAA
  ============================================================

Primers for ID: 197
Primer pair 1, product size : 692
  Primer left                            Primer right
  start: 25210129                        start: 25210799
  end  : 25210149                        end  : 25210820
  tm   : 57                              tm   : 59
  seq  : AAAGTTCTGGCTGATGGTGTC           seq  : GGGACAATTCCTTCTGACTTTG

  >197_primer_pair_1_product
  AAAGTTCTGGCTGATGGTGTCATAGGCTGGGAAGAGAGAGGCCAGGAGAAAAGGCTGAGG
  AAACTGCTGGCAAATGTGAAGGGCAAGAATGAATGCCCAAGGTGGGCAGCAGGTGAGGAA
  AGAGTCCCTCACCTCCCTGGAGGAACAAGTCTTTGATTTGCTGAAAGGCATCCCGCACAG
  CCTGGGCGCACTTGGGACTCTGGCCATAAAAGTCCTGGAGAAGAGACCAAGGTTGCTGCT
  GCCATTCTTGCACTGGCCTGGGGTACCCAAGTCCCCTCACTCACCGCTGTGACATCTCGG
  AAGAATTGGTAGGAGTCCCCAAGGCCTGCAACAGCTACAACAGGAGCGCTGGCTGCCAGT
  GCCCCAGCCACCAGGTGGGGGTACTTCATCCTCATGTAGGCACTCAGCATCCCCCCATAA
  CTGGGAGTACAGAGCACAGATCATGGTTGTGGGAAGCTGCCCACAACTCAGGCGAGCAGC
  CTCACTGTCCTCCAGGCTGAGGTGCTAGGCTGCTCTTTCCCTGCTCAGAACGCCCAAGGG
  TGGGAAAGAAGGACCTGAAACTGTCAGGCCCACACACCCTGATCCCAGGGCCAAGGCAGA
  TACAGCCTTCACTGGGAGAAGGCACCTGTGGGTGCCCTGCCCTGACCCAGCAATGAAGAC
  ATTGCAGAGACAAAGTCAGAAGGAATTGTCCC
  --------------------

Primer pair 2, product size : 661
  Primer left                            Primer right
  start: 25210129                        start: 25210770
  end  : 25210149                        end  : 25210789
  tm   : 57                              tm   : 55
  seq  : AAAGTTCTGGCTGATGGTGTC           seq  : TGTCTTCATTGCTGGGTCAG

  >197_primer_pair_2_product
  AAAGTTCTGGCTGATGGTGTCATAGGCTGGGAAGAGAGAGGCCAGGAGAAAAGGCTGAGG
  AAACTGCTGGCAAATGTGAAGGGCAAGAATGAATGCCCAAGGTGGGCAGCAGGTGAGGAA
  AGAGTCCCTCACCTCCCTGGAGGAACAAGTCTTTGATTTGCTGAAAGGCATCCCGCACAG
  CCTGGGCGCACTTGGGACTCTGGCCATAAAAGTCCTGGAGAAGAGACCAAGGTTGCTGCT
  GCCATTCTTGCACTGGCCTGGGGTACCCAAGTCCCCTCACTCACCGCTGTGACATCTCGG
  AAGAATTGGTAGGAGTCCCCAAGGCCTGCAACAGCTACAACAGGAGCGCTGGCTGCCAGT
  GCCCCAGCCACCAGGTGGGGGTACTTCATCCTCATGTAGGCACTCAGCATCCCCCCATAA
  CTGGGAGTACAGAGCACAGATCATGGTTGTGGGAAGCTGCCCACAACTCAGGCGAGCAGC
  CTCACTGTCCTCCAGGCTGAGGTGCTAGGCTGCTCTTTCCCTGCTCAGAACGCCCAAGGG
  TGGGAAAGAAGGACCTGAAACTGTCAGGCCCACACACCCTGATCCCAGGGCCAAGGCAGA
  TACAGCCTTCACTGGGAGAAGGCACCTGTGGGTGCCCTGCCCTGACCCAGCAATGAAGAC
  A
  ============================================================

Primers for ID: 198
Primer pair 1, product size : 608
  Primer left                            Primer right
  start: 25210213                        start: 25210799
  end  : 25210232                        end  : 25210820
  tm   : 53                              tm   : 59
  seq  : AAGAATGAATGCCCAAGGTG            seq  : GGGACAATTCCTTCTGACTTTG

  >198_primer_pair_1_product
  AAGAATGAATGCCCAAGGTGGGCAGCAGGTGAGGAAAGAGTCCCTCACCTCCCTGGAGGA
  ACAAGTCTTTGATTTGCTGAAAGGCATCCCGCACAGCCTGGGCGCACTTGGGACTCTGGC
  CATAAAAGTCCTGGAGAAGAGACCAAGGTTGCTGCTGCCATTCTTGCACTGGCCTGGGGT
  ACCCAAGTCCCCTCACTCACCGCTGTGACATCTCGGAAGAATTGGTAGGAGTCCCCAAGG
  CCTGCAACAGCTACAACAGGAGCGCTGGCTGCCAGTGCCCCAGCCACCAGGTGGGGGTAC
  TTCATCCTCATGTAGGCACTCAGCATCCCCCCATAACTGGGAGTACAGAGCACAGATCAT
  GGTTGTGGGAAGCTGCCCACAACTCAGGCGAGCAGCCTCACTGTCCTCCAGGCTGAGGTG
  CTAGGCTGCTCTTTCCCTGCTCAGAACGCCCAAGGGTGGGAAAGAAGGACCTGAAACTGT
  CAGGCCCACACACCCTGATCCCAGGGCCAAGGCAGATACAGCCTTCACTGGGAGAAGGCA
  CCTGTGGGTGCCCTGCCCTGACCCAGCAATGAAGACATTGCAGAGACAAAGTCAGAAGGA
  ATTGTCCC
  --------------------

Primer pair 2, product size : 618
  Primer left                            Primer right
  start: 25210172                        start: 25210770
  end  : 25210192                        end  : 25210789
  tm   : 57                              tm   : 55
  seq  : AGGAGAAAAGGCTGAGGAAAC           seq  : TGTCTTCATTGCTGGGTCAG

  >198_primer_pair_2_product
  AGGAGAAAAGGCTGAGGAAACTGCTGGCAAATGTGAAGGGCAAGAATGAATGCCCAAGGT
  GGGCAGCAGGTGAGGAAAGAGTCCCTCACCTCCCTGGAGGAACAAGTCTTTGATTTGCTG
  AAAGGCATCCCGCACAGCCTGGGCGCACTTGGGACTCTGGCCATAAAAGTCCTGGAGAAG
  AGACCAAGGTTGCTGCTGCCATTCTTGCACTGGCCTGGGGTACCCAAGTCCCCTCACTCA
  CCGCTGTGACATCTCGGAAGAATTGGTAGGAGTCCCCAAGGCCTGCAACAGCTACAACAG
  GAGCGCTGGCTGCCAGTGCCCCAGCCACCAGGTGGGGGTACTTCATCCTCATGTAGGCAC
  TCAGCATCCCCCCATAACTGGGAGTACAGAGCACAGATCATGGTTGTGGGAAGCTGCCCA
  CAACTCAGGCGAGCAGCCTCACTGTCCTCCAGGCTGAGGTGCTAGGCTGCTCTTTCCCTG
  CTCAGAACGCCCAAGGGTGGGAAAGAAGGACCTGAAACTGTCAGGCCCACACACCCTGAT
  CCCAGGGCCAAGGCAGATACAGCCTTCACTGGGAGAAGGCACCTGTGGGTGCCCTGCCCT
  GACCCAGCAATGAAGACA
  ============================================================

Primers for ID: 199
Primer pair 1, product size : 608
  Primer left                            Primer right
  start: 25210213                        start: 25210799
  end  : 25210232                        end  : 25210820
  tm   : 53                              tm   : 59
  seq  : AAGAATGAATGCCCAAGGTG            seq  : GGGACAATTCCTTCTGACTTTG

  >199_primer_pair_1_product
  AAGAATGAATGCCCAAGGTGGGCAGCAGGTGAGGAAAGAGTCCCTCACCTCCCTGGAGGA
  ACAAGTCTTTGATTTGCTGAAAGGCATCCCGCACAGCCTGGGCGCACTTGGGACTCTGGC
  CATAAAAGTCCTGGAGAAGAGACCAAGGTTGCTGCTGCCATTCTTGCACTGGCCTGGGGT
  ACCCAAGTCCCCTCACTCACCGCTGTGACATCTCGGAAGAATTGGTAGGAGTCCCCAAGG
  CCTGCAACAGCTACAACAGGAGCGCTGGCTGCCAGTGCCCCAGCCACCAGGTGGGGGTAC
  TTCATCCTCATGTAGGCACTCAGCATCCCCCCATAACTGGGAGTACAGAGCACAGATCAT
  GGTTGTGGGAAGCTGCCCACAACTCAGGCGAGCAGCCTCACTGTCCTCCAGGCTGAGGTG
  CTAGGCTGCTCTTTCCCTGCTCAGAACGCCCAAGGGTGGGAAAGAAGGACCTGAAACTGT
  CAGGCCCACACACCCTGATCCCAGGGCCAAGGCAGATACAGCCTTCACTGGGAGAAGGCA
  CCTGTGGGTGCCCTGCCCTGACCCAGCAATGAAGACATTGCAGAGACAAAGTCAGAAGGA
  ATTGTCCC
  --------------------

Primer pair 2, product size : 695
  Primer left                            Primer right
  start: 25210277                        start: 25210951
  end  : 25210297                        end  : 25210971
  tm   : 55                              tm   : 57
  seq  : GTCTTTGATTTGCTGAAAGGC           seq  : TATACAAGGAGGGCCAAAGAG

  >199_primer_pair_2_product
  GTCTTTGATTTGCTGAAAGGCATCCCGCACAGCCTGGGCGCACTTGGGACTCTGGCCATA
  AAAGTCCTGGAGAAGAGACCAAGGTTGCTGCTGCCATTCTTGCACTGGCCTGGGGTACCC
  AAGTCCCCTCACTCACCGCTGTGACATCTCGGAAGAATTGGTAGGAGTCCCCAAGGCCTG
  CAACAGCTACAACAGGAGCGCTGGCTGCCAGTGCCCCAGCCACCAGGTGGGGGTACTTCA
  TCCTCATGTAGGCACTCAGCATCCCCCCATAACTGGGAGTACAGAGCACAGATCATGGTT
  GTGGGAAGCTGCCCACAACTCAGGCGAGCAGCCTCACTGTCCTCCAGGCTGAGGTGCTAG
  GCTGCTCTTTCCCTGCTCAGAACGCCCAAGGGTGGGAAAGAAGGACCTGAAACTGTCAGG
  CCCACACACCCTGATCCCAGGGCCAAGGCAGATACAGCCTTCACTGGGAGAAGGCACCTG
  TGGGTGCCCTGCCCTGACCCAGCAATGAAGACATTGCAGAGACAAAGTCAGAAGGAATTG
  TCCCACTAGTGGGAACAACATAGCATACACTGCCTATGAGGTCCACTCAAGGAGGGCTTC
  CAGAAGGAGGTAAAGCTAGACCCCGCCCTTCCACATGTGGGGTAGGCATAGGATGTTGAG
  ACTGTAAGAGACATCTCTTTGGCCCTCCTTGTATA
  ============================================================

Primers for ID: 200
Primer pair 1, product size : 635
  Primer left                            Primer right
  start: 25210417                        start: 25211030
  end  : 25210437                        end  : 25211051
  tm   : 57                              tm   : 61
  seq  : GTGACATCTCGGAAGAATTGG           seq  : AGATCTGGACCCATGAGAGAAC

  >200_primer_pair_1_product
  GTGACATCTCGGAAGAATTGGTAGGAGTCCCCAAGGCCTGCAACAGCTACAACAGGAGCG
  CTGGCTGCCAGTGCCCCAGCCACCAGGTGGGGGTACTTCATCCTCATGTAGGCACTCAGC
  ATCCCCCCATAACTGGGAGTACAGAGCACAGATCATGGTTGTGGGAAGCTGCCCACAACT
  CAGGCGAGCAGCCTCACTGTCCTCCAGGCTGAGGTGCTAGGCTGCTCTTTCCCTGCTCAG
  AACGCCCAAGGGTGGGAAAGAAGGACCTGAAACTGTCAGGCCCACACACCCTGATCCCAG
  GGCCAAGGCAGATACAGCCTTCACTGGGAGAAGGCACCTGTGGGTGCCCTGCCCTGACCC
  AGCAATGAAGACATTGCAGAGACAAAGTCAGAAGGAATTGTCCCACTAGTGGGAACAACA
  TAGCATACACTGCCTATGAGGTCCACTCAAGGAGGGCTTCCAGAAGGAGGTAAAGCTAGA
  CCCCGCCCTTCCACATGTGGGGTAGGCATAGGATGTTGAGACTGTAAGAGACATCTCTTT
  GGCCCTCCTTGTATAGGGTGTCAATCGGCACAACAGGGTGGAGCCTTAGAGTAGGGTAAG
  ATTAGGACTCTAGGTTCTCTCATGGGTCCAGATCT
  --------------------

Primer pair 2, product size : 637
  Primer left                            Primer right
  start: 25210415                        start: 25211030
  end  : 25210436                        end  : 25211051
  tm   : 59                              tm   : 61
  seq  : CTGTGACATCTCGGAAGAATTG          seq  : AGATCTGGACCCATGAGAGAAC

  >200_primer_pair_2_product
  CTGTGACATCTCGGAAGAATTGGTAGGAGTCCCCAAGGCCTGCAACAGCTACAACAGGAG
  CGCTGGCTGCCAGTGCCCCAGCCACCAGGTGGGGGTACTTCATCCTCATGTAGGCACTCA
  GCATCCCCCCATAACTGGGAGTACAGAGCACAGATCATGGTTGTGGGAAGCTGCCCACAA
  CTCAGGCGAGCAGCCTCACTGTCCTCCAGGCTGAGGTGCTAGGCTGCTCTTTCCCTGCTC
  AGAACGCCCAAGGGTGGGAAAGAAGGACCTGAAACTGTCAGGCCCACACACCCTGATCCC
  AGGGCCAAGGCAGATACAGCCTTCACTGGGAGAAGGCACCTGTGGGTGCCCTGCCCTGAC
  CCAGCAATGAAGACATTGCAGAGACAAAGTCAGAAGGAATTGTCCCACTAGTGGGAACAA
  CATAGCATACACTGCCTATGAGGTCCACTCAAGGAGGGCTTCCAGAAGGAGGTAAAGCTA
  GACCCCGCCCTTCCACATGTGGGGTAGGCATAGGATGTTGAGACTGTAAGAGACATCTCT
  TTGGCCCTCCTTGTATAGGGTGTCAATCGGCACAACAGGGTGGAGCCTTAGAGTAGGGTA
  AGATTAGGACTCTAGGTTCTCTCATGGGTCCAGATCT
  ============================================================

Primers for ID: 201
Primer pair 1, product size : 635
  Primer left                            Primer right
  start: 25210417                        start: 25211030
  end  : 25210437                        end  : 25211051
  tm   : 57                              tm   : 61
  seq  : GTGACATCTCGGAAGAATTGG           seq  : AGATCTGGACCCATGAGAGAAC

  >201_primer_pair_1_product
  GTGACATCTCGGAAGAATTGGTAGGAGTCCCCAAGGCCTGCAACAGCTACAACAGGAGCG
  CTGGCTGCCAGTGCCCCAGCCACCAGGTGGGGGTACTTCATCCTCATGTAGGCACTCAGC
  ATCCCCCCATAACTGGGAGTACAGAGCACAGATCATGGTTGTGGGAAGCTGCCCACAACT
  CAGGCGAGCAGCCTCACTGTCCTCCAGGCTGAGGTGCTAGGCTGCTCTTTCCCTGCTCAG
  AACGCCCAAGGGTGGGAAAGAAGGACCTGAAACTGTCAGGCCCACACACCCTGATCCCAG
  GGCCAAGGCAGATACAGCCTTCACTGGGAGAAGGCACCTGTGGGTGCCCTGCCCTGACCC
  AGCAATGAAGACATTGCAGAGACAAAGTCAGAAGGAATTGTCCCACTAGTGGGAACAACA
  TAGCATACACTGCCTATGAGGTCCACTCAAGGAGGGCTTCCAGAAGGAGGTAAAGCTAGA
  CCCCGCCCTTCCACATGTGGGGTAGGCATAGGATGTTGAGACTGTAAGAGACATCTCTTT
  GGCCCTCCTTGTATAGGGTGTCAATCGGCACAACAGGGTGGAGCCTTAGAGTAGGGTAAG
  ATTAGGACTCTAGGTTCTCTCATGGGTCCAGATCT
  --------------------

Primer pair 2, product size : 637
  Primer left                            Primer right
  start: 25210415                        start: 25211030
  end  : 25210436                        end  : 25211051
  tm   : 59                              tm   : 61
  seq  : CTGTGACATCTCGGAAGAATTG          seq  : AGATCTGGACCCATGAGAGAAC

  >201_primer_pair_2_product
  CTGTGACATCTCGGAAGAATTGGTAGGAGTCCCCAAGGCCTGCAACAGCTACAACAGGAG
  CGCTGGCTGCCAGTGCCCCAGCCACCAGGTGGGGGTACTTCATCCTCATGTAGGCACTCA
  GCATCCCCCCATAACTGGGAGTACAGAGCACAGATCATGGTTGTGGGAAGCTGCCCACAA
  CTCAGGCGAGCAGCCTCACTGTCCTCCAGGCTGAGGTGCTAGGCTGCTCTTTCCCTGCTC
  AGAACGCCCAAGGGTGGGAAAGAAGGACCTGAAACTGTCAGGCCCACACACCCTGATCCC
  AGGGCCAAGGCAGATACAGCCTTCACTGGGAGAAGGCACCTGTGGGTGCCCTGCCCTGAC
  CCAGCAATGAAGACATTGCAGAGACAAAGTCAGAAGGAATTGTCCCACTAGTGGGAACAA
  CATAGCATACACTGCCTATGAGGTCCACTCAAGGAGGGCTTCCAGAAGGAGGTAAAGCTA
  GACCCCGCCCTTCCACATGTGGGGTAGGCATAGGATGTTGAGACTGTAAGAGACATCTCT
  TTGGCCCTCCTTGTATAGGGTGTCAATCGGCACAACAGGGTGGAGCCTTAGAGTAGGGTA
  AGATTAGGACTCTAGGTTCTCTCATGGGTCCAGATCT
  ============================================================

Primers for ID: 211
Primer pair 1, product size : 616
  Primer left                            Primer right
  start: 25211226                        start: 25211821
  end  : 25211245                        end  : 25211841
  tm   : 55                              tm   : 57
  seq  : ATTTCCCATAGTACCGCTGC            seq  : TTCCATCCTTGTTACCCTGTG

  >211_primer_pair_1_product
  ATTTCCCATAGTACCGCTGCAGAAAGCAGGAAGGGATGGCTAATCCACTCCTCGGTGCTC
  CCCACCTCCTTCAACTCAGGGACTGCCAGGAACTGTACAGGTACCCACGTGCTCAGCAAA
  GACAAGCAGGGCCTCCTGCTGGGCTGCCAGTTCCACCATGAAGCCAGAGTTGTTAGCGAA
  GGACCAGATATCCCCCTCATTCCCTGTGTAGAAAAAGATGGGCCCTTCGCCCATCTTCCA
  GAACTTATCTGTTGGAAGTAAATGAGTTTCCATAAGGCCAGGGAAACGCAGGTAGGAACC
  CATGCGGTCGAGCCAGCACTCACCTGACACTAGGAACCGCTGGCCAAAGGTTTTGTTGCC
  GAAACTCTCAAAGTTGAAATGGTCCATGTATTGCTCAAAATAATTCTCATGAAAGTCAGG
  GTCTAGAACTCTGTCGGCTGAGGGCAGGTGCAGAGACTCAGGAGCTGGTTGGGATCATCA
  GGGATCTAGGCGGGTCAGGAGGAAGGGCAGCCAGTCTGTACTCACCTCTGGCCTGGAGGT
  TGCACAGTCCCAGTGACAGCAGCAGGACCAGGATCCAGGAGGGGACACCATGGTCCACAG
  GGTAACAAGGATGGAA
  --------------------

Primer pair 2, product size : 620
  Primer left                            Primer right
  start: 25211222                        start: 25211821
  end  : 25211241                        end  : 25211841
  tm   : 55                              tm   : 57
  seq  : AGCGATTTCCCATAGTACCG            seq  : TTCCATCCTTGTTACCCTGTG

  >211_primer_pair_2_product
  AGCGATTTCCCATAGTACCGCTGCAGAAAGCAGGAAGGGATGGCTAATCCACTCCTCGGT
  GCTCCCCACCTCCTTCAACTCAGGGACTGCCAGGAACTGTACAGGTACCCACGTGCTCAG
  CAAAGACAAGCAGGGCCTCCTGCTGGGCTGCCAGTTCCACCATGAAGCCAGAGTTGTTAG
  CGAAGGACCAGATATCCCCCTCATTCCCTGTGTAGAAAAAGATGGGCCCTTCGCCCATCT
  TCCAGAACTTATCTGTTGGAAGTAAATGAGTTTCCATAAGGCCAGGGAAACGCAGGTAGG
  AACCCATGCGGTCGAGCCAGCACTCACCTGACACTAGGAACCGCTGGCCAAAGGTTTTGT
  TGCCGAAACTCTCAAAGTTGAAATGGTCCATGTATTGCTCAAAATAATTCTCATGAAAGT
  CAGGGTCTAGAACTCTGTCGGCTGAGGGCAGGTGCAGAGACTCAGGAGCTGGTTGGGATC
  ATCAGGGATCTAGGCGGGTCAGGAGGAAGGGCAGCCAGTCTGTACTCACCTCTGGCCTGG
  AGGTTGCACAGTCCCAGTGACAGCAGCAGGACCAGGATCCAGGAGGGGACACCATGGTCC
  ACAGGGTAACAAGGATGGAA
  ============================================================

Primers for ID: 212
Primer pair 1, product size : 620
  Primer left                            Primer right
  start: 25211383                        start: 25211982
  end  : 25211403                        end  : 25212002
  tm   : 57                              tm   : 57
  seq  : ATGAAGCCAGAGTTGTTAGCG           seq  : AACCGGGAAACTGAAGAAGAG

  >212_primer_pair_1_product
  ATGAAGCCAGAGTTGTTAGCGAAGGACCAGATATCCCCCTCATTCCCTGTGTAGAAAAAG
  ATGGGCCCTTCGCCCATCTTCCAGAACTTATCTGTTGGAAGTAAATGAGTTTCCATAAGG
  CCAGGGAAACGCAGGTAGGAACCCATGCGGTCGAGCCAGCACTCACCTGACACTAGGAAC
  CGCTGGCCAAAGGTTTTGTTGCCGAAACTCTCAAAGTTGAAATGGTCCATGTATTGCTCA
  AAATAATTCTCATGAAAGTCAGGGTCTAGAACTCTGTCGGCTGAGGGCAGGTGCAGAGAC
  TCAGGAGCTGGTTGGGATCATCAGGGATCTAGGCGGGTCAGGAGGAAGGGCAGCCAGTCT
  GTACTCACCTCTGGCCTGGAGGTTGCACAGTCCCAGTGACAGCAGCAGGACCAGGATCCA
  GGAGGGGACACCATGGTCCACAGGGTAACAAGGATGGAAGTTCATGCTTGATTCTGAGCC
  GGGCGCTGACTGTCATGTGATTTGGTCACATGACCGACACAACGGGCGGGGCAGCATCAC
  GTGATAGTCTGGCGGGGGCTGTCCTACTGTGGCTGGATTCTAGTTGGAGGATCAGCCTAC
  TCTTCTTCAGTTTCCCGGTT
  --------------------

Primer pair 2, product size : 612
  Primer left                            Primer right
  start: 25211391                        start: 25211982
  end  : 25211412                        end  : 25212002
  tm   : 61                              tm   : 57
  seq  : AGAGTTGTTAGCGAAGGACCAG          seq  : AACCGGGAAACTGAAGAAGAG

  >212_primer_pair_2_product
  AGAGTTGTTAGCGAAGGACCAGATATCCCCCTCATTCCCTGTGTAGAAAAAGATGGGCCC
  TTCGCCCATCTTCCAGAACTTATCTGTTGGAAGTAAATGAGTTTCCATAAGGCCAGGGAA
  ACGCAGGTAGGAACCCATGCGGTCGAGCCAGCACTCACCTGACACTAGGAACCGCTGGCC
  AAAGGTTTTGTTGCCGAAACTCTCAAAGTTGAAATGGTCCATGTATTGCTCAAAATAATT
  CTCATGAAAGTCAGGGTCTAGAACTCTGTCGGCTGAGGGCAGGTGCAGAGACTCAGGAGC
  TGGTTGGGATCATCAGGGATCTAGGCGGGTCAGGAGGAAGGGCAGCCAGTCTGTACTCAC
  CTCTGGCCTGGAGGTTGCACAGTCCCAGTGACAGCAGCAGGACCAGGATCCAGGAGGGGA
  CACCATGGTCCACAGGGTAACAAGGATGGAAGTTCATGCTTGATTCTGAGCCGGGCGCTG
  ACTGTCATGTGATTTGGTCACATGACCGACACAACGGGCGGGGCAGCATCACGTGATAGT
  CTGGCGGGGGCTGTCCTACTGTGGCTGGATTCTAGTTGGAGGATCAGCCTACTCTTCTTC
  AGTTTCCCGGTT
  ============================================================

Primers for ID: 213
Primer pair 1, product size : 620
  Primer left                            Primer right
  start: 25211383                        start: 25211982
  end  : 25211403                        end  : 25212002
  tm   : 57                              tm   : 57
  seq  : ATGAAGCCAGAGTTGTTAGCG           seq  : AACCGGGAAACTGAAGAAGAG

  >213_primer_pair_1_product
  ATGAAGCCAGAGTTGTTAGCGAAGGACCAGATATCCCCCTCATTCCCTGTGTAGAAAAAG
  ATGGGCCCTTCGCCCATCTTCCAGAACTTATCTGTTGGAAGTAAATGAGTTTCCATAAGG
  CCAGGGAAACGCAGGTAGGAACCCATGCGGTCGAGCCAGCACTCACCTGACACTAGGAAC
  CGCTGGCCAAAGGTTTTGTTGCCGAAACTCTCAAAGTTGAAATGGTCCATGTATTGCTCA
  AAATAATTCTCATGAAAGTCAGGGTCTAGAACTCTGTCGGCTGAGGGCAGGTGCAGAGAC
  TCAGGAGCTGGTTGGGATCATCAGGGATCTAGGCGGGTCAGGAGGAAGGGCAGCCAGTCT
  GTACTCACCTCTGGCCTGGAGGTTGCACAGTCCCAGTGACAGCAGCAGGACCAGGATCCA
  GGAGGGGACACCATGGTCCACAGGGTAACAAGGATGGAAGTTCATGCTTGATTCTGAGCC
  GGGCGCTGACTGTCATGTGATTTGGTCACATGACCGACACAACGGGCGGGGCAGCATCAC
  GTGATAGTCTGGCGGGGGCTGTCCTACTGTGGCTGGATTCTAGTTGGAGGATCAGCCTAC
  TCTTCTTCAGTTTCCCGGTT
  --------------------

Primer pair 2, product size : 612
  Primer left                            Primer right
  start: 25211391                        start: 25211982
  end  : 25211412                        end  : 25212002
  tm   : 61                              tm   : 57
  seq  : AGAGTTGTTAGCGAAGGACCAG          seq  : AACCGGGAAACTGAAGAAGAG

  >213_primer_pair_2_product
  AGAGTTGTTAGCGAAGGACCAGATATCCCCCTCATTCCCTGTGTAGAAAAAGATGGGCCC
  TTCGCCCATCTTCCAGAACTTATCTGTTGGAAGTAAATGAGTTTCCATAAGGCCAGGGAA
  ACGCAGGTAGGAACCCATGCGGTCGAGCCAGCACTCACCTGACACTAGGAACCGCTGGCC
  AAAGGTTTTGTTGCCGAAACTCTCAAAGTTGAAATGGTCCATGTATTGCTCAAAATAATT
  CTCATGAAAGTCAGGGTCTAGAACTCTGTCGGCTGAGGGCAGGTGCAGAGACTCAGGAGC
  TGGTTGGGATCATCAGGGATCTAGGCGGGTCAGGAGGAAGGGCAGCCAGTCTGTACTCAC
  CTCTGGCCTGGAGGTTGCACAGTCCCAGTGACAGCAGCAGGACCAGGATCCAGGAGGGGA
  CACCATGGTCCACAGGGTAACAAGGATGGAAGTTCATGCTTGATTCTGAGCCGGGCGCTG
  ACTGTCATGTGATTTGGTCACATGACCGACACAACGGGCGGGGCAGCATCACGTGATAGT
  CTGGCGGGGGCTGTCCTACTGTGGCTGGATTCTAGTTGGAGGATCAGCCTACTCTTCTTC
  AGTTTCCCGGTT
  ============================================================

Primers for ID: 214
Primer pair 1, product size : 670
  Primer left                            Primer right
  start: 25211428                        start: 25212076
  end  : 25211448                        end  : 25212097
  tm   : 57                              tm   : 61
  seq  : CCTGTGTAGAAAAAGATGGGC           seq  : ATACGGTCCTGATGAGCCTTAG

  >214_primer_pair_1_product
  CCTGTGTAGAAAAAGATGGGCCCTTCGCCCATCTTCCAGAACTTATCTGTTGGAAGTAAA
  TGAGTTTCCATAAGGCCAGGGAAACGCAGGTAGGAACCCATGCGGTCGAGCCAGCACTCA
  CCTGACACTAGGAACCGCTGGCCAAAGGTTTTGTTGCCGAAACTCTCAAAGTTGAAATGG
  TCCATGTATTGCTCAAAATAATTCTCATGAAAGTCAGGGTCTAGAACTCTGTCGGCTGAG
  GGCAGGTGCAGAGACTCAGGAGCTGGTTGGGATCATCAGGGATCTAGGCGGGTCAGGAGG
  AAGGGCAGCCAGTCTGTACTCACCTCTGGCCTGGAGGTTGCACAGTCCCAGTGACAGCAG
  CAGGACCAGGATCCAGGAGGGGACACCATGGTCCACAGGGTAACAAGGATGGAAGTTCAT
  GCTTGATTCTGAGCCGGGCGCTGACTGTCATGTGATTTGGTCACATGACCGACACAACGG
  GCGGGGCAGCATCACGTGATAGTCTGGCGGGGGCTGTCCTACTGTGGCTGGATTCTAGTT
  GGAGGATCAGCCTACTCTTCTTCAGTTTCCCGGTTCCTCCAAATTTCTGGGCTCCTACTT
  GTTTCCACAGAGATGGATACTGTGGAGGTCCAGGAAGCAGAGAGATGGCTAAGGCTCATC
  AGGACCGTAT
  --------------------

Primer pair 2, product size : 671
  Primer left                            Primer right
  start: 25211427                        start: 25212076
  end  : 25211447                        end  : 25212097
  tm   : 57                              tm   : 61
  seq  : CCCTGTGTAGAAAAAGATGGG           seq  : ATACGGTCCTGATGAGCCTTAG

  >214_primer_pair_2_product
  CCCTGTGTAGAAAAAGATGGGCCCTTCGCCCATCTTCCAGAACTTATCTGTTGGAAGTAA
  ATGAGTTTCCATAAGGCCAGGGAAACGCAGGTAGGAACCCATGCGGTCGAGCCAGCACTC
  ACCTGACACTAGGAACCGCTGGCCAAAGGTTTTGTTGCCGAAACTCTCAAAGTTGAAATG
  GTCCATGTATTGCTCAAAATAATTCTCATGAAAGTCAGGGTCTAGAACTCTGTCGGCTGA
  GGGCAGGTGCAGAGACTCAGGAGCTGGTTGGGATCATCAGGGATCTAGGCGGGTCAGGAG
  GAAGGGCAGCCAGTCTGTACTCACCTCTGGCCTGGAGGTTGCACAGTCCCAGTGACAGCA
  GCAGGACCAGGATCCAGGAGGGGACACCATGGTCCACAGGGTAACAAGGATGGAAGTTCA
  TGCTTGATTCTGAGCCGGGCGCTGACTGTCATGTGATTTGGTCACATGACCGACACAACG
  GGCGGGGCAGCATCACGTGATAGTCTGGCGGGGGCTGTCCTACTGTGGCTGGATTCTAGT
  TGGAGGATCAGCCTACTCTTCTTCAGTTTCCCGGTTCCTCCAAATTTCTGGGCTCCTACT
  TGTTTCCACAGAGATGGATACTGTGGAGGTCCAGGAAGCAGAGAGATGGCTAAGGCTCAT
  CAGGACCGTAT
  ============================================================

Primers for ID: 215
Primer pair 1, product size : 681
  Primer left                            Primer right
  start: 25211600                        start: 25212260
  end  : 25211621                        end  : 25212280
  tm   : 57                              tm   : 57
  seq  : TGAAATGGTCCATGTATTGCTC          seq  : GAAGCAGAGAGGTCATTTTGG

  >215_primer_pair_1_product
  TGAAATGGTCCATGTATTGCTCAAAATAATTCTCATGAAAGTCAGGGTCTAGAACTCTGT
  CGGCTGAGGGCAGGTGCAGAGACTCAGGAGCTGGTTGGGATCATCAGGGATCTAGGCGGG
  TCAGGAGGAAGGGCAGCCAGTCTGTACTCACCTCTGGCCTGGAGGTTGCACAGTCCCAGT
  GACAGCAGCAGGACCAGGATCCAGGAGGGGACACCATGGTCCACAGGGTAACAAGGATGG
  AAGTTCATGCTTGATTCTGAGCCGGGCGCTGACTGTCATGTGATTTGGTCACATGACCGA
  CACAACGGGCGGGGCAGCATCACGTGATAGTCTGGCGGGGGCTGTCCTACTGTGGCTGGA
  TTCTAGTTGGAGGATCAGCCTACTCTTCTTCAGTTTCCCGGTTCCTCCAAATTTCTGGGC
  TCCTACTTGTTTCCACAGAGATGGATACTGTGGAGGTCCAGGAAGCAGAGAGATGGCTAA
  GGCTCATCAGGACCGTATGATCTCCCAAGTGTCCAGCTACTGAGTACCACAAGGTGATGG
  GTGGGAGGGTCCTCCCACGGAAGGATACCGCAGTCCCTAGGGGTTGCAAGCCCCACATGT
  TCCACTGGCTGCTAGAGCTACCTACTCAATCAGCCCTGGGCATCACCATCAGGTACTCGG
  CCAAAATGACCTCTCTGCTTC
  --------------------

Primer pair 2, product size : 682
  Primer left                            Primer right
  start: 25211600                        start: 25212261
  end  : 25211621                        end  : 25212281
  tm   : 57                              tm   : 57
  seq  : TGAAATGGTCCATGTATTGCTC          seq  : GGAAGCAGAGAGGTCATTTTG

  >215_primer_pair_2_product
  TGAAATGGTCCATGTATTGCTCAAAATAATTCTCATGAAAGTCAGGGTCTAGAACTCTGT
  CGGCTGAGGGCAGGTGCAGAGACTCAGGAGCTGGTTGGGATCATCAGGGATCTAGGCGGG
  TCAGGAGGAAGGGCAGCCAGTCTGTACTCACCTCTGGCCTGGAGGTTGCACAGTCCCAGT
  GACAGCAGCAGGACCAGGATCCAGGAGGGGACACCATGGTCCACAGGGTAACAAGGATGG
  AAGTTCATGCTTGATTCTGAGCCGGGCGCTGACTGTCATGTGATTTGGTCACATGACCGA
  CACAACGGGCGGGGCAGCATCACGTGATAGTCTGGCGGGGGCTGTCCTACTGTGGCTGGA
  TTCTAGTTGGAGGATCAGCCTACTCTTCTTCAGTTTCCCGGTTCCTCCAAATTTCTGGGC
  TCCTACTTGTTTCCACAGAGATGGATACTGTGGAGGTCCAGGAAGCAGAGAGATGGCTAA
  GGCTCATCAGGACCGTATGATCTCCCAAGTGTCCAGCTACTGAGTACCACAAGGTGATGG
  GTGGGAGGGTCCTCCCACGGAAGGATACCGCAGTCCCTAGGGGTTGCAAGCCCCACATGT
  TCCACTGGCTGCTAGAGCTACCTACTCAATCAGCCCTGGGCATCACCATCAGGTACTCGG
  CCAAAATGACCTCTCTGCTTCC
  ============================================================

Primers for ID: 216
Primer pair 1, product size : 722
  Primer left                            Primer right
  start: 25211600                        start: 25212301
  end  : 25211621                        end  : 25212321
  tm   : 57                              tm   : 57
  seq  : TGAAATGGTCCATGTATTGCTC          seq  : TAATTATGGGCCTGTCTGGTG

  >216_primer_pair_1_product
  TGAAATGGTCCATGTATTGCTCAAAATAATTCTCATGAAAGTCAGGGTCTAGAACTCTGT
  CGGCTGAGGGCAGGTGCAGAGACTCAGGAGCTGGTTGGGATCATCAGGGATCTAGGCGGG
  TCAGGAGGAAGGGCAGCCAGTCTGTACTCACCTCTGGCCTGGAGGTTGCACAGTCCCAGT
  GACAGCAGCAGGACCAGGATCCAGGAGGGGACACCATGGTCCACAGGGTAACAAGGATGG
  AAGTTCATGCTTGATTCTGAGCCGGGCGCTGACTGTCATGTGATTTGGTCACATGACCGA
  CACAACGGGCGGGGCAGCATCACGTGATAGTCTGGCGGGGGCTGTCCTACTGTGGCTGGA
  TTCTAGTTGGAGGATCAGCCTACTCTTCTTCAGTTTCCCGGTTCCTCCAAATTTCTGGGC
  TCCTACTTGTTTCCACAGAGATGGATACTGTGGAGGTCCAGGAAGCAGAGAGATGGCTAA
  GGCTCATCAGGACCGTATGATCTCCCAAGTGTCCAGCTACTGAGTACCACAAGGTGATGG
  GTGGGAGGGTCCTCCCACGGAAGGATACCGCAGTCCCTAGGGGTTGCAAGCCCCACATGT
  TCCACTGGCTGCTAGAGCTACCTACTCAATCAGCCCTGGGCATCACCATCAGGTACTCGG
  CCAAAATGACCTCTCTGCTTCCAGTCCTCAGTTCTGGTCAGCACCAGACAGGCCCATAAT
  TA
  --------------------

Primer pair 2, product size : 724
  Primer left                            Primer right
  start: 25211600                        start: 25212303
  end  : 25211621                        end  : 25212323
  tm   : 57                              tm   : 57
  seq  : TGAAATGGTCCATGTATTGCTC          seq  : TGTAATTATGGGCCTGTCTGG

  >216_primer_pair_2_product
  TGAAATGGTCCATGTATTGCTCAAAATAATTCTCATGAAAGTCAGGGTCTAGAACTCTGT
  CGGCTGAGGGCAGGTGCAGAGACTCAGGAGCTGGTTGGGATCATCAGGGATCTAGGCGGG
  TCAGGAGGAAGGGCAGCCAGTCTGTACTCACCTCTGGCCTGGAGGTTGCACAGTCCCAGT
  GACAGCAGCAGGACCAGGATCCAGGAGGGGACACCATGGTCCACAGGGTAACAAGGATGG
  AAGTTCATGCTTGATTCTGAGCCGGGCGCTGACTGTCATGTGATTTGGTCACATGACCGA
  CACAACGGGCGGGGCAGCATCACGTGATAGTCTGGCGGGGGCTGTCCTACTGTGGCTGGA
  TTCTAGTTGGAGGATCAGCCTACTCTTCTTCAGTTTCCCGGTTCCTCCAAATTTCTGGGC
  TCCTACTTGTTTCCACAGAGATGGATACTGTGGAGGTCCAGGAAGCAGAGAGATGGCTAA
  GGCTCATCAGGACCGTATGATCTCCCAAGTGTCCAGCTACTGAGTACCACAAGGTGATGG
  GTGGGAGGGTCCTCCCACGGAAGGATACCGCAGTCCCTAGGGGTTGCAAGCCCCACATGT
  TCCACTGGCTGCTAGAGCTACCTACTCAATCAGCCCTGGGCATCACCATCAGGTACTCGG
  CCAAAATGACCTCTCTGCTTCCAGTCCTCAGTTCTGGTCAGCACCAGACAGGCCCATAAT
  TACA
  ============================================================

Primers for ID: 217
Primer pair 1, product size : 602
  Primer left                            Primer right
  start: 25211842                        start: 25212422
  end  : 25211862                        end  : 25212443
  tm   : 57                              tm   : 61
  seq  : GTTCATGCTTGATTCTGAGCC           seq  : GCTCAGAGATTCCCAGAAAGTC

  >217_primer_pair_1_product
  GTTCATGCTTGATTCTGAGCCGGGCGCTGACTGTCATGTGATTTGGTCACATGACCGACA
  CAACGGGCGGGGCAGCATCACGTGATAGTCTGGCGGGGGCTGTCCTACTGTGGCTGGATT
  CTAGTTGGAGGATCAGCCTACTCTTCTTCAGTTTCCCGGTTCCTCCAAATTTCTGGGCTC
  CTACTTGTTTCCACAGAGATGGATACTGTGGAGGTCCAGGAAGCAGAGAGATGGCTAAGG
  CTCATCAGGACCGTATGATCTCCCAAGTGTCCAGCTACTGAGTACCACAAGGTGATGGGT
  GGGAGGGTCCTCCCACGGAAGGATACCGCAGTCCCTAGGGGTTGCAAGCCCCACATGTTC
  CACTGGCTGCTAGAGCTACCTACTCAATCAGCCCTGGGCATCACCATCAGGTACTCGGCC
  AAAATGACCTCTCTGCTTCCAGTCCTCAGTTCTGGTCAGCACCAGACAGGCCCATAATTA
  CAGAGCCAGGGAAACTGGAACATTTGTCTCCCCTTAGACAGTGGCAGCAGGAAGGTGGGG
  GGTTGTTGCAGAGGAACAGTGTCTCTGAGAGAGGACCTTGGACTTTCTGGGAATCTCTGA
  GC
  --------------------

Primer pair 2, product size : 601
  Primer left                            Primer right
  start: 25211842                        start: 25212421
  end  : 25211862                        end  : 25212442
  tm   : 57                              tm   : 61
  seq  : GTTCATGCTTGATTCTGAGCC           seq  : CTCAGAGATTCCCAGAAAGTCC

  >217_primer_pair_2_product
  GTTCATGCTTGATTCTGAGCCGGGCGCTGACTGTCATGTGATTTGGTCACATGACCGACA
  CAACGGGCGGGGCAGCATCACGTGATAGTCTGGCGGGGGCTGTCCTACTGTGGCTGGATT
  CTAGTTGGAGGATCAGCCTACTCTTCTTCAGTTTCCCGGTTCCTCCAAATTTCTGGGCTC
  CTACTTGTTTCCACAGAGATGGATACTGTGGAGGTCCAGGAAGCAGAGAGATGGCTAAGG
  CTCATCAGGACCGTATGATCTCCCAAGTGTCCAGCTACTGAGTACCACAAGGTGATGGGT
  GGGAGGGTCCTCCCACGGAAGGATACCGCAGTCCCTAGGGGTTGCAAGCCCCACATGTTC
  CACTGGCTGCTAGAGCTACCTACTCAATCAGCCCTGGGCATCACCATCAGGTACTCGGCC
  AAAATGACCTCTCTGCTTCCAGTCCTCAGTTCTGGTCAGCACCAGACAGGCCCATAATTA
  CAGAGCCAGGGAAACTGGAACATTTGTCTCCCCTTAGACAGTGGCAGCAGGAAGGTGGGG
  GGTTGTTGCAGAGGAACAGTGTCTCTGAGAGAGGACCTTGGACTTTCTGGGAATCTCTGA
  G
  ============================================================

Primers for ID: 218
Primer pair 1, product size : 602
  Primer left                            Primer right
  start: 25211842                        start: 25212422
  end  : 25211862                        end  : 25212443
  tm   : 57                              tm   : 61
  seq  : GTTCATGCTTGATTCTGAGCC           seq  : GCTCAGAGATTCCCAGAAAGTC

  >218_primer_pair_1_product
  GTTCATGCTTGATTCTGAGCCGGGCGCTGACTGTCATGTGATTTGGTCACATGACCGACA
  CAACGGGCGGGGCAGCATCACGTGATAGTCTGGCGGGGGCTGTCCTACTGTGGCTGGATT
  CTAGTTGGAGGATCAGCCTACTCTTCTTCAGTTTCCCGGTTCCTCCAAATTTCTGGGCTC
  CTACTTGTTTCCACAGAGATGGATACTGTGGAGGTCCAGGAAGCAGAGAGATGGCTAAGG
  CTCATCAGGACCGTATGATCTCCCAAGTGTCCAGCTACTGAGTACCACAAGGTGATGGGT
  GGGAGGGTCCTCCCACGGAAGGATACCGCAGTCCCTAGGGGTTGCAAGCCCCACATGTTC
  CACTGGCTGCTAGAGCTACCTACTCAATCAGCCCTGGGCATCACCATCAGGTACTCGGCC
  AAAATGACCTCTCTGCTTCCAGTCCTCAGTTCTGGTCAGCACCAGACAGGCCCATAATTA
  CAGAGCCAGGGAAACTGGAACATTTGTCTCCCCTTAGACAGTGGCAGCAGGAAGGTGGGG
  GGTTGTTGCAGAGGAACAGTGTCTCTGAGAGAGGACCTTGGACTTTCTGGGAATCTCTGA
  GC
  --------------------

Primer pair 2, product size : 601
  Primer left                            Primer right
  start: 25211842                        start: 25212421
  end  : 25211862                        end  : 25212442
  tm   : 57                              tm   : 61
  seq  : GTTCATGCTTGATTCTGAGCC           seq  : CTCAGAGATTCCCAGAAAGTCC

  >218_primer_pair_2_product
  GTTCATGCTTGATTCTGAGCCGGGCGCTGACTGTCATGTGATTTGGTCACATGACCGACA
  CAACGGGCGGGGCAGCATCACGTGATAGTCTGGCGGGGGCTGTCCTACTGTGGCTGGATT
  CTAGTTGGAGGATCAGCCTACTCTTCTTCAGTTTCCCGGTTCCTCCAAATTTCTGGGCTC
  CTACTTGTTTCCACAGAGATGGATACTGTGGAGGTCCAGGAAGCAGAGAGATGGCTAAGG
  CTCATCAGGACCGTATGATCTCCCAAGTGTCCAGCTACTGAGTACCACAAGGTGATGGGT
  GGGAGGGTCCTCCCACGGAAGGATACCGCAGTCCCTAGGGGTTGCAAGCCCCACATGTTC
  CACTGGCTGCTAGAGCTACCTACTCAATCAGCCCTGGGCATCACCATCAGGTACTCGGCC
  AAAATGACCTCTCTGCTTCCAGTCCTCAGTTCTGGTCAGCACCAGACAGGCCCATAATTA
  CAGAGCCAGGGAAACTGGAACATTTGTCTCCCCTTAGACAGTGGCAGCAGGAAGGTGGGG
  GGTTGTTGCAGAGGAACAGTGTCTCTGAGAGAGGACCTTGGACTTTCTGGGAATCTCTGA
  G
  ============================================================

Primers for ID: 219
Primer pair 1, product size : 719
  Primer left                            Primer right
  start: 25211842                        start: 25212540
  end  : 25211862                        end  : 25212560
  tm   : 57                              tm   : 57
  seq  : GTTCATGCTTGATTCTGAGCC           seq  : ATGAGACACTCTGTGCCAATG

  >219_primer_pair_1_product
  GTTCATGCTTGATTCTGAGCCGGGCGCTGACTGTCATGTGATTTGGTCACATGACCGACA
  CAACGGGCGGGGCAGCATCACGTGATAGTCTGGCGGGGGCTGTCCTACTGTGGCTGGATT
  CTAGTTGGAGGATCAGCCTACTCTTCTTCAGTTTCCCGGTTCCTCCAAATTTCTGGGCTC
  CTACTTGTTTCCACAGAGATGGATACTGTGGAGGTCCAGGAAGCAGAGAGATGGCTAAGG
  CTCATCAGGACCGTATGATCTCCCAAGTGTCCAGCTACTGAGTACCACAAGGTGATGGGT
  GGGAGGGTCCTCCCACGGAAGGATACCGCAGTCCCTAGGGGTTGCAAGCCCCACATGTTC
  CACTGGCTGCTAGAGCTACCTACTCAATCAGCCCTGGGCATCACCATCAGGTACTCGGCC
  AAAATGACCTCTCTGCTTCCAGTCCTCAGTTCTGGTCAGCACCAGACAGGCCCATAATTA
  CAGAGCCAGGGAAACTGGAACATTTGTCTCCCCTTAGACAGTGGCAGCAGGAAGGTGGGG
  GGTTGTTGCAGAGGAACAGTGTCTCTGAGAGAGGACCTTGGACTTTCTGGGAATCTCTGA
  GCTGCCCGGTTCTCCCCACTGCTGGCACTGTGCCCACAGCCCAAACAGAATGGGGGAGAT
  GGAGGGGCAGGGCTTCTGTGGGAAGCTGCCCTCCACCTCATTGGCACAGAGTGTCTCAT
  --------------------

Primer pair 2, product size : 602
  Primer left                            Primer right
  start: 25211842                        start: 25212422
  end  : 25211862                        end  : 25212443
  tm   : 57                              tm   : 61
  seq  : GTTCATGCTTGATTCTGAGCC           seq  : GCTCAGAGATTCCCAGAAAGTC

  >219_primer_pair_2_product
  GTTCATGCTTGATTCTGAGCCGGGCGCTGACTGTCATGTGATTTGGTCACATGACCGACA
  CAACGGGCGGGGCAGCATCACGTGATAGTCTGGCGGGGGCTGTCCTACTGTGGCTGGATT
  CTAGTTGGAGGATCAGCCTACTCTTCTTCAGTTTCCCGGTTCCTCCAAATTTCTGGGCTC
  CTACTTGTTTCCACAGAGATGGATACTGTGGAGGTCCAGGAAGCAGAGAGATGGCTAAGG
  CTCATCAGGACCGTATGATCTCCCAAGTGTCCAGCTACTGAGTACCACAAGGTGATGGGT
  GGGAGGGTCCTCCCACGGAAGGATACCGCAGTCCCTAGGGGTTGCAAGCCCCACATGTTC
  CACTGGCTGCTAGAGCTACCTACTCAATCAGCCCTGGGCATCACCATCAGGTACTCGGCC
  AAAATGACCTCTCTGCTTCCAGTCCTCAGTTCTGGTCAGCACCAGACAGGCCCATAATTA
  CAGAGCCAGGGAAACTGGAACATTTGTCTCCCCTTAGACAGTGGCAGCAGGAAGGTGGGG
  GGTTGTTGCAGAGGAACAGTGTCTCTGAGAGAGGACCTTGGACTTTCTGGGAATCTCTGA
  GC
  ============================================================

Primers for ID: 220
Primer pair 1, product size : 662
  Primer left                            Primer right
  start: 25212011                        start: 25212653
  end  : 25212032                        end  : 25212672
  tm   : 59                              tm   : 55
  seq  : TTTCTGGGCTCCTACTTGTTTC          seq  : ATGGACATGTTCCACTCACG

  >220_primer_pair_1_product
  TTTCTGGGCTCCTACTTGTTTCCACAGAGATGGATACTGTGGAGGTCCAGGAAGCAGAGA
  GATGGCTAAGGCTCATCAGGACCGTATGATCTCCCAAGTGTCCAGCTACTGAGTACCACA
  AGGTGATGGGTGGGAGGGTCCTCCCACGGAAGGATACCGCAGTCCCTAGGGGTTGCAAGC
  CCCACATGTTCCACTGGCTGCTAGAGCTACCTACTCAATCAGCCCTGGGCATCACCATCA
  GGTACTCGGCCAAAATGACCTCTCTGCTTCCAGTCCTCAGTTCTGGTCAGCACCAGACAG
  GCCCATAATTACAGAGCCAGGGAAACTGGAACATTTGTCTCCCCTTAGACAGTGGCAGCA
  GGAAGGTGGGGGGTTGTTGCAGAGGAACAGTGTCTCTGAGAGAGGACCTTGGACTTTCTG
  GGAATCTCTGAGCTGCCCGGTTCTCCCCACTGCTGGCACTGTGCCCACAGCCCAAACAGA
  ATGGGGGAGATGGAGGGGCAGGGCTTCTGTGGGAAGCTGCCCTCCACCTCATTGGCACAG
  AGTGTCTCATTGCAGAGAGAAAAAAGGACCAGTTTTCTCTCTGGCACCCAGGTCTGGAAG
  AGGAGTGACATCCACGGAAGTTGGTGACTTGGACTGGCTGGCCGTGAGTGGAACATGTCC
  AT
  --------------------

Primer pair 2, product size : 604
  Primer left                            Primer right
  start: 25211957                        start: 25212540
  end  : 25211978                        end  : 25212560
  tm   : 61                              tm   : 57
  seq  : GGATTCTAGTTGGAGGATCAGC          seq  : ATGAGACACTCTGTGCCAATG

  >220_primer_pair_2_product
  GGATTCTAGTTGGAGGATCAGCCTACTCTTCTTCAGTTTCCCGGTTCCTCCAAATTTCTG
  GGCTCCTACTTGTTTCCACAGAGATGGATACTGTGGAGGTCCAGGAAGCAGAGAGATGGC
  TAAGGCTCATCAGGACCGTATGATCTCCCAAGTGTCCAGCTACTGAGTACCACAAGGTGA
  TGGGTGGGAGGGTCCTCCCACGGAAGGATACCGCAGTCCCTAGGGGTTGCAAGCCCCACA
  TGTTCCACTGGCTGCTAGAGCTACCTACTCAATCAGCCCTGGGCATCACCATCAGGTACT
  CGGCCAAAATGACCTCTCTGCTTCCAGTCCTCAGTTCTGGTCAGCACCAGACAGGCCCAT
  AATTACAGAGCCAGGGAAACTGGAACATTTGTCTCCCCTTAGACAGTGGCAGCAGGAAGG
  TGGGGGGTTGTTGCAGAGGAACAGTGTCTCTGAGAGAGGACCTTGGACTTTCTGGGAATC
  TCTGAGCTGCCCGGTTCTCCCCACTGCTGGCACTGTGCCCACAGCCCAAACAGAATGGGG
  GAGATGGAGGGGCAGGGCTTCTGTGGGAAGCTGCCCTCCACCTCATTGGCACAGAGTGTC
  TCAT
  ============================================================

Primers for ID: 221
Primer pair 1, product size : 612
  Primer left                            Primer right
  start: 25209826                        start: 25210417
  end  : 25209847                        end  : 25210437
  tm   : 61                              tm   : 57
  seq  : AGCAGAATATCTCAGTGGAGGC          seq  : CCAATTCTTCCGAGATGTCAC

  >221_primer_pair_1_product
  AGCAGAATATCTCAGTGGAGGCCCCTTTCACAGAGCGTGGGTCAGGGCTGCTAGCTTCCA
  GGACACAACAGCAGATAGTGTCTGATGGCATGAAAGCAGATAGCTACAAGGCTCTTGGAC
  CAGGCTAGCACTGGGTCCTGCACCCAGGGAGAGCCACCTCACCTTGACAGGGTTGGCAGG
  AAGGGGCCCTAGAAAGTCAGTAGGATACGGGTAGTCCATCATGGCGAGCACAGTAAATGC
  ATTTCGGGCAAACCCAAAGAGCTGAGTCAGGTCCTTTGGGCTGGAAAGTGATTGACAGGT
  ACCAAAGTTCTGGCTGATGGTGTCATAGGCTGGGAAGAGAGAGGCCAGGAGAAAAGGCTG
  AGGAAACTGCTGGCAAATGTGAAGGGCAAGAATGAATGCCCAAGGTGGGCAGCAGGTGAG
  GAAAGAGTCCCTCACCTCCCTGGAGGAACAAGTCTTTGATTTGCTGAAAGGCATCCCGCA
  CAGCCTGGGCGCACTTGGGACTCTGGCCATAAAAGTCCTGGAGAAGAGACCAAGGTTGCT
  GCTGCCATTCTTGCACTGGCCTGGGGTACCCAAGTCCCCTCACTCACCGCTGTGACATCT
  CGGAAGAATTGG
  --------------------

Primer pair 2, product size : 614
  Primer left                            Primer right
  start: 25209824                        start: 25210417
  end  : 25209845                        end  : 25210437
  tm   : 61                              tm   : 57
  seq  : GCAGCAGAATATCTCAGTGGAG          seq  : CCAATTCTTCCGAGATGTCAC

  >221_primer_pair_2_product
  GCAGCAGAATATCTCAGTGGAGGCCCCTTTCACAGAGCGTGGGTCAGGGCTGCTAGCTTC
  CAGGACACAACAGCAGATAGTGTCTGATGGCATGAAAGCAGATAGCTACAAGGCTCTTGG
  ACCAGGCTAGCACTGGGTCCTGCACCCAGGGAGAGCCACCTCACCTTGACAGGGTTGGCA
  GGAAGGGGCCCTAGAAAGTCAGTAGGATACGGGTAGTCCATCATGGCGAGCACAGTAAAT
  GCATTTCGGGCAAACCCAAAGAGCTGAGTCAGGTCCTTTGGGCTGGAAAGTGATTGACAG
  GTACCAAAGTTCTGGCTGATGGTGTCATAGGCTGGGAAGAGAGAGGCCAGGAGAAAAGGC
  TGAGGAAACTGCTGGCAAATGTGAAGGGCAAGAATGAATGCCCAAGGTGGGCAGCAGGTG
  AGGAAAGAGTCCCTCACCTCCCTGGAGGAACAAGTCTTTGATTTGCTGAAAGGCATCCCG
  CACAGCCTGGGCGCACTTGGGACTCTGGCCATAAAAGTCCTGGAGAAGAGACCAAGGTTG
  CTGCTGCCATTCTTGCACTGGCCTGGGGTACCCAAGTCCCCTCACTCACCGCTGTGACAT
  CTCGGAAGAATTGG
  ============================================================

Primers for ID: 222
Primer pair 1, product size : 612
  Primer left                            Primer right
  start: 25209826                        start: 25210417
  end  : 25209847                        end  : 25210437
  tm   : 61                              tm   : 57
  seq  : AGCAGAATATCTCAGTGGAGGC          seq  : CCAATTCTTCCGAGATGTCAC

  >222_primer_pair_1_product
  AGCAGAATATCTCAGTGGAGGCCCCTTTCACAGAGCGTGGGTCAGGGCTGCTAGCTTCCA
  GGACACAACAGCAGATAGTGTCTGATGGCATGAAAGCAGATAGCTACAAGGCTCTTGGAC
  CAGGCTAGCACTGGGTCCTGCACCCAGGGAGAGCCACCTCACCTTGACAGGGTTGGCAGG
  AAGGGGCCCTAGAAAGTCAGTAGGATACGGGTAGTCCATCATGGCGAGCACAGTAAATGC
  ATTTCGGGCAAACCCAAAGAGCTGAGTCAGGTCCTTTGGGCTGGAAAGTGATTGACAGGT
  ACCAAAGTTCTGGCTGATGGTGTCATAGGCTGGGAAGAGAGAGGCCAGGAGAAAAGGCTG
  AGGAAACTGCTGGCAAATGTGAAGGGCAAGAATGAATGCCCAAGGTGGGCAGCAGGTGAG
  GAAAGAGTCCCTCACCTCCCTGGAGGAACAAGTCTTTGATTTGCTGAAAGGCATCCCGCA
  CAGCCTGGGCGCACTTGGGACTCTGGCCATAAAAGTCCTGGAGAAGAGACCAAGGTTGCT
  GCTGCCATTCTTGCACTGGCCTGGGGTACCCAAGTCCCCTCACTCACCGCTGTGACATCT
  CGGAAGAATTGG
  --------------------

Primer pair 2, product size : 614
  Primer left                            Primer right
  start: 25209824                        start: 25210417
  end  : 25209845                        end  : 25210437
  tm   : 61                              tm   : 57
  seq  : GCAGCAGAATATCTCAGTGGAG          seq  : CCAATTCTTCCGAGATGTCAC

  >222_primer_pair_2_product
  GCAGCAGAATATCTCAGTGGAGGCCCCTTTCACAGAGCGTGGGTCAGGGCTGCTAGCTTC
  CAGGACACAACAGCAGATAGTGTCTGATGGCATGAAAGCAGATAGCTACAAGGCTCTTGG
  ACCAGGCTAGCACTGGGTCCTGCACCCAGGGAGAGCCACCTCACCTTGACAGGGTTGGCA
  GGAAGGGGCCCTAGAAAGTCAGTAGGATACGGGTAGTCCATCATGGCGAGCACAGTAAAT
  GCATTTCGGGCAAACCCAAAGAGCTGAGTCAGGTCCTTTGGGCTGGAAAGTGATTGACAG
  GTACCAAAGTTCTGGCTGATGGTGTCATAGGCTGGGAAGAGAGAGGCCAGGAGAAAAGGC
  TGAGGAAACTGCTGGCAAATGTGAAGGGCAAGAATGAATGCCCAAGGTGGGCAGCAGGTG
  AGGAAAGAGTCCCTCACCTCCCTGGAGGAACAAGTCTTTGATTTGCTGAAAGGCATCCCG
  CACAGCCTGGGCGCACTTGGGACTCTGGCCATAAAAGTCCTGGAGAAGAGACCAAGGTTG
  CTGCTGCCATTCTTGCACTGGCCTGGGGTACCCAAGTCCCCTCACTCACCGCTGTGACAT
  CTCGGAAGAATTGG
  ============================================================

Primers for ID: 223
Primer pair 1, product size : 670
  Primer left                            Primer right
  start: 25209909                        start: 25210558
  end  : 25209929                        end  : 25210578
  tm   : 57                              tm   : 57
  seq  : GATGGCATGAAAGCAGATAGC           seq  : ACAACCATGATCTGTGCTCTG

  >223_primer_pair_1_product
  GATGGCATGAAAGCAGATAGCTACAAGGCTCTTGGACCAGGCTAGCACTGGGTCCTGCAC
  CCAGGGAGAGCCACCTCACCTTGACAGGGTTGGCAGGAAGGGGCCCTAGAAAGTCAGTAG
  GATACGGGTAGTCCATCATGGCGAGCACAGTAAATGCATTTCGGGCAAACCCAAAGAGCT
  GAGTCAGGTCCTTTGGGCTGGAAAGTGATTGACAGGTACCAAAGTTCTGGCTGATGGTGT
  CATAGGCTGGGAAGAGAGAGGCCAGGAGAAAAGGCTGAGGAAACTGCTGGCAAATGTGAA
  GGGCAAGAATGAATGCCCAAGGTGGGCAGCAGGTGAGGAAAGAGTCCCTCACCTCCCTGG
  AGGAACAAGTCTTTGATTTGCTGAAAGGCATCCCGCACAGCCTGGGCGCACTTGGGACTC
  TGGCCATAAAAGTCCTGGAGAAGAGACCAAGGTTGCTGCTGCCATTCTTGCACTGGCCTG
  GGGTACCCAAGTCCCCTCACTCACCGCTGTGACATCTCGGAAGAATTGGTAGGAGTCCCC
  AAGGCCTGCAACAGCTACAACAGGAGCGCTGGCTGCCAGTGCCCCAGCCACCAGGTGGGG
  GTACTTCATCCTCATGTAGGCACTCAGCATCCCCCCATAACTGGGAGTACAGAGCACAGA
  TCATGGTTGT
  --------------------

Primer pair 2, product size : 656
  Primer left                            Primer right
  start: 25209909                        start: 25210543
  end  : 25209929                        end  : 25210564
  tm   : 57                              tm   : 61
  seq  : GATGGCATGAAAGCAGATAGC           seq  : TGCTCTGTACTCCCAGTTATGG

  >223_primer_pair_2_product
  GATGGCATGAAAGCAGATAGCTACAAGGCTCTTGGACCAGGCTAGCACTGGGTCCTGCAC
  CCAGGGAGAGCCACCTCACCTTGACAGGGTTGGCAGGAAGGGGCCCTAGAAAGTCAGTAG
  GATACGGGTAGTCCATCATGGCGAGCACAGTAAATGCATTTCGGGCAAACCCAAAGAGCT
  GAGTCAGGTCCTTTGGGCTGGAAAGTGATTGACAGGTACCAAAGTTCTGGCTGATGGTGT
  CATAGGCTGGGAAGAGAGAGGCCAGGAGAAAAGGCTGAGGAAACTGCTGGCAAATGTGAA
  GGGCAAGAATGAATGCCCAAGGTGGGCAGCAGGTGAGGAAAGAGTCCCTCACCTCCCTGG
  AGGAACAAGTCTTTGATTTGCTGAAAGGCATCCCGCACAGCCTGGGCGCACTTGGGACTC
  TGGCCATAAAAGTCCTGGAGAAGAGACCAAGGTTGCTGCTGCCATTCTTGCACTGGCCTG
  GGGTACCCAAGTCCCCTCACTCACCGCTGTGACATCTCGGAAGAATTGGTAGGAGTCCCC
  AAGGCCTGCAACAGCTACAACAGGAGCGCTGGCTGCCAGTGCCCCAGCCACCAGGTGGGG
  GTACTTCATCCTCATGTAGGCACTCAGCATCCCCCCATAACTGGGAGTACAGAGCA
  ============================================================

Primers for ID: 224
Primer pair 1, product size : 562
  Primer left                            Primer right
  start: 25210129                        start: 25210670
  end  : 25210149                        end  : 25210690
  tm   : 57                              tm   : 57
  seq  : AAAGTTCTGGCTGATGGTGTC           seq  : AGTTTCAGGTCCTTCTTTCCC

  >224_primer_pair_1_product
  AAAGTTCTGGCTGATGGTGTCATAGGCTGGGAAGAGAGAGGCCAGGAGAAAAGGCTGAGG
  AAACTGCTGGCAAATGTGAAGGGCAAGAATGAATGCCCAAGGTGGGCAGCAGGTGAGGAA
  AGAGTCCCTCACCTCCCTGGAGGAACAAGTCTTTGATTTGCTGAAAGGCATCCCGCACAG
  CCTGGGCGCACTTGGGACTCTGGCCATAAAAGTCCTGGAGAAGAGACCAAGGTTGCTGCT
  GCCATTCTTGCACTGGCCTGGGGTACCCAAGTCCCCTCACTCACCGCTGTGACATCTCGG
  AAGAATTGGTAGGAGTCCCCAAGGCCTGCAACAGCTACAACAGGAGCGCTGGCTGCCAGT
  GCCCCAGCCACCAGGTGGGGGTACTTCATCCTCATGTAGGCACTCAGCATCCCCCCATAA
  CTGGGAGTACAGAGCACAGATCATGGTTGTGGGAAGCTGCCCACAACTCAGGCGAGCAGC
  CTCACTGTCCTCCAGGCTGAGGTGCTAGGCTGCTCTTTCCCTGCTCAGAACGCCCAAGGG
  TGGGAAAGAAGGACCTGAAACT
  --------------------

Primer pair 2, product size : 558
  Primer left                            Primer right
  start: 25210133                        start: 25210670
  end  : 25210153                        end  : 25210690
  tm   : 57                              tm   : 57
  seq  : TTCTGGCTGATGGTGTCATAG           seq  : AGTTTCAGGTCCTTCTTTCCC

  >224_primer_pair_2_product
  TTCTGGCTGATGGTGTCATAGGCTGGGAAGAGAGAGGCCAGGAGAAAAGGCTGAGGAAAC
  TGCTGGCAAATGTGAAGGGCAAGAATGAATGCCCAAGGTGGGCAGCAGGTGAGGAAAGAG
  TCCCTCACCTCCCTGGAGGAACAAGTCTTTGATTTGCTGAAAGGCATCCCGCACAGCCTG
  GGCGCACTTGGGACTCTGGCCATAAAAGTCCTGGAGAAGAGACCAAGGTTGCTGCTGCCA
  TTCTTGCACTGGCCTGGGGTACCCAAGTCCCCTCACTCACCGCTGTGACATCTCGGAAGA
  ATTGGTAGGAGTCCCCAAGGCCTGCAACAGCTACAACAGGAGCGCTGGCTGCCAGTGCCC
  CAGCCACCAGGTGGGGGTACTTCATCCTCATGTAGGCACTCAGCATCCCCCCATAACTGG
  GAGTACAGAGCACAGATCATGGTTGTGGGAAGCTGCCCACAACTCAGGCGAGCAGCCTCA
  CTGTCCTCCAGGCTGAGGTGCTAGGCTGCTCTTTCCCTGCTCAGAACGCCCAAGGGTGGG
  AAAGAAGGACCTGAAACT
  ============================================================

Primers for ID: 225
Primer pair 1, product size : 562
  Primer left                            Primer right
  start: 25210129                        start: 25210670
  end  : 25210149                        end  : 25210690
  tm   : 57                              tm   : 57
  seq  : AAAGTTCTGGCTGATGGTGTC           seq  : AGTTTCAGGTCCTTCTTTCCC

  >225_primer_pair_1_product
  AAAGTTCTGGCTGATGGTGTCATAGGCTGGGAAGAGAGAGGCCAGGAGAAAAGGCTGAGG
  AAACTGCTGGCAAATGTGAAGGGCAAGAATGAATGCCCAAGGTGGGCAGCAGGTGAGGAA
  AGAGTCCCTCACCTCCCTGGAGGAACAAGTCTTTGATTTGCTGAAAGGCATCCCGCACAG
  CCTGGGCGCACTTGGGACTCTGGCCATAAAAGTCCTGGAGAAGAGACCAAGGTTGCTGCT
  GCCATTCTTGCACTGGCCTGGGGTACCCAAGTCCCCTCACTCACCGCTGTGACATCTCGG
  AAGAATTGGTAGGAGTCCCCAAGGCCTGCAACAGCTACAACAGGAGCGCTGGCTGCCAGT
  GCCCCAGCCACCAGGTGGGGGTACTTCATCCTCATGTAGGCACTCAGCATCCCCCCATAA
  CTGGGAGTACAGAGCACAGATCATGGTTGTGGGAAGCTGCCCACAACTCAGGCGAGCAGC
  CTCACTGTCCTCCAGGCTGAGGTGCTAGGCTGCTCTTTCCCTGCTCAGAACGCCCAAGGG
  TGGGAAAGAAGGACCTGAAACT
  --------------------

Primer pair 2, product size : 558
  Primer left                            Primer right
  start: 25210133                        start: 25210670
  end  : 25210153                        end  : 25210690
  tm   : 57                              tm   : 57
  seq  : TTCTGGCTGATGGTGTCATAG           seq  : AGTTTCAGGTCCTTCTTTCCC

  >225_primer_pair_2_product
  TTCTGGCTGATGGTGTCATAGGCTGGGAAGAGAGAGGCCAGGAGAAAAGGCTGAGGAAAC
  TGCTGGCAAATGTGAAGGGCAAGAATGAATGCCCAAGGTGGGCAGCAGGTGAGGAAAGAG
  TCCCTCACCTCCCTGGAGGAACAAGTCTTTGATTTGCTGAAAGGCATCCCGCACAGCCTG
  GGCGCACTTGGGACTCTGGCCATAAAAGTCCTGGAGAAGAGACCAAGGTTGCTGCTGCCA
  TTCTTGCACTGGCCTGGGGTACCCAAGTCCCCTCACTCACCGCTGTGACATCTCGGAAGA
  ATTGGTAGGAGTCCCCAAGGCCTGCAACAGCTACAACAGGAGCGCTGGCTGCCAGTGCCC
  CAGCCACCAGGTGGGGGTACTTCATCCTCATGTAGGCACTCAGCATCCCCCCATAACTGG
  GAGTACAGAGCACAGATCATGGTTGTGGGAAGCTGCCCACAACTCAGGCGAGCAGCCTCA
  CTGTCCTCCAGGCTGAGGTGCTAGGCTGCTCTTTCCCTGCTCAGAACGCCCAAGGGTGGG
  AAAGAAGGACCTGAAACT
  ============================================================

Primers for ID: 226
Primer pair 1, product size : 692
  Primer left                            Primer right
  start: 25210129                        start: 25210799
  end  : 25210149                        end  : 25210820
  tm   : 57                              tm   : 59
  seq  : AAAGTTCTGGCTGATGGTGTC           seq  : GGGACAATTCCTTCTGACTTTG

  >226_primer_pair_1_product
  AAAGTTCTGGCTGATGGTGTCATAGGCTGGGAAGAGAGAGGCCAGGAGAAAAGGCTGAGG
  AAACTGCTGGCAAATGTGAAGGGCAAGAATGAATGCCCAAGGTGGGCAGCAGGTGAGGAA
  AGAGTCCCTCACCTCCCTGGAGGAACAAGTCTTTGATTTGCTGAAAGGCATCCCGCACAG
  CCTGGGCGCACTTGGGACTCTGGCCATAAAAGTCCTGGAGAAGAGACCAAGGTTGCTGCT
  GCCATTCTTGCACTGGCCTGGGGTACCCAAGTCCCCTCACTCACCGCTGTGACATCTCGG
  AAGAATTGGTAGGAGTCCCCAAGGCCTGCAACAGCTACAACAGGAGCGCTGGCTGCCAGT
  GCCCCAGCCACCAGGTGGGGGTACTTCATCCTCATGTAGGCACTCAGCATCCCCCCATAA
  CTGGGAGTACAGAGCACAGATCATGGTTGTGGGAAGCTGCCCACAACTCAGGCGAGCAGC
  CTCACTGTCCTCCAGGCTGAGGTGCTAGGCTGCTCTTTCCCTGCTCAGAACGCCCAAGGG
  TGGGAAAGAAGGACCTGAAACTGTCAGGCCCACACACCCTGATCCCAGGGCCAAGGCAGA
  TACAGCCTTCACTGGGAGAAGGCACCTGTGGGTGCCCTGCCCTGACCCAGCAATGAAGAC
  ATTGCAGAGACAAAGTCAGAAGGAATTGTCCC
  --------------------

Primer pair 2, product size : 661
  Primer left                            Primer right
  start: 25210129                        start: 25210770
  end  : 25210149                        end  : 25210789
  tm   : 57                              tm   : 55
  seq  : AAAGTTCTGGCTGATGGTGTC           seq  : TGTCTTCATTGCTGGGTCAG

  >226_primer_pair_2_product
  AAAGTTCTGGCTGATGGTGTCATAGGCTGGGAAGAGAGAGGCCAGGAGAAAAGGCTGAGG
  AAACTGCTGGCAAATGTGAAGGGCAAGAATGAATGCCCAAGGTGGGCAGCAGGTGAGGAA
  AGAGTCCCTCACCTCCCTGGAGGAACAAGTCTTTGATTTGCTGAAAGGCATCCCGCACAG
  CCTGGGCGCACTTGGGACTCTGGCCATAAAAGTCCTGGAGAAGAGACCAAGGTTGCTGCT
  GCCATTCTTGCACTGGCCTGGGGTACCCAAGTCCCCTCACTCACCGCTGTGACATCTCGG
  AAGAATTGGTAGGAGTCCCCAAGGCCTGCAACAGCTACAACAGGAGCGCTGGCTGCCAGT
  GCCCCAGCCACCAGGTGGGGGTACTTCATCCTCATGTAGGCACTCAGCATCCCCCCATAA
  CTGGGAGTACAGAGCACAGATCATGGTTGTGGGAAGCTGCCCACAACTCAGGCGAGCAGC
  CTCACTGTCCTCCAGGCTGAGGTGCTAGGCTGCTCTTTCCCTGCTCAGAACGCCCAAGGG
  TGGGAAAGAAGGACCTGAAACTGTCAGGCCCACACACCCTGATCCCAGGGCCAAGGCAGA
  TACAGCCTTCACTGGGAGAAGGCACCTGTGGGTGCCCTGCCCTGACCCAGCAATGAAGAC
  A
  ============================================================

Primers for ID: 227
Primer pair 1, product size : 608
  Primer left                            Primer right
  start: 25210213                        start: 25210799
  end  : 25210232                        end  : 25210820
  tm   : 53                              tm   : 59
  seq  : AAGAATGAATGCCCAAGGTG            seq  : GGGACAATTCCTTCTGACTTTG

  >227_primer_pair_1_product
  AAGAATGAATGCCCAAGGTGGGCAGCAGGTGAGGAAAGAGTCCCTCACCTCCCTGGAGGA
  ACAAGTCTTTGATTTGCTGAAAGGCATCCCGCACAGCCTGGGCGCACTTGGGACTCTGGC
  CATAAAAGTCCTGGAGAAGAGACCAAGGTTGCTGCTGCCATTCTTGCACTGGCCTGGGGT
  ACCCAAGTCCCCTCACTCACCGCTGTGACATCTCGGAAGAATTGGTAGGAGTCCCCAAGG
  CCTGCAACAGCTACAACAGGAGCGCTGGCTGCCAGTGCCCCAGCCACCAGGTGGGGGTAC
  TTCATCCTCATGTAGGCACTCAGCATCCCCCCATAACTGGGAGTACAGAGCACAGATCAT
  GGTTGTGGGAAGCTGCCCACAACTCAGGCGAGCAGCCTCACTGTCCTCCAGGCTGAGGTG
  CTAGGCTGCTCTTTCCCTGCTCAGAACGCCCAAGGGTGGGAAAGAAGGACCTGAAACTGT
  CAGGCCCACACACCCTGATCCCAGGGCCAAGGCAGATACAGCCTTCACTGGGAGAAGGCA
  CCTGTGGGTGCCCTGCCCTGACCCAGCAATGAAGACATTGCAGAGACAAAGTCAGAAGGA
  ATTGTCCC
  --------------------

Primer pair 2, product size : 577
  Primer left                            Primer right
  start: 25210213                        start: 25210770
  end  : 25210232                        end  : 25210789
  tm   : 53                              tm   : 55
  seq  : AAGAATGAATGCCCAAGGTG            seq  : TGTCTTCATTGCTGGGTCAG

  >227_primer_pair_2_product
  AAGAATGAATGCCCAAGGTGGGCAGCAGGTGAGGAAAGAGTCCCTCACCTCCCTGGAGGA
  ACAAGTCTTTGATTTGCTGAAAGGCATCCCGCACAGCCTGGGCGCACTTGGGACTCTGGC
  CATAAAAGTCCTGGAGAAGAGACCAAGGTTGCTGCTGCCATTCTTGCACTGGCCTGGGGT
  ACCCAAGTCCCCTCACTCACCGCTGTGACATCTCGGAAGAATTGGTAGGAGTCCCCAAGG
  CCTGCAACAGCTACAACAGGAGCGCTGGCTGCCAGTGCCCCAGCCACCAGGTGGGGGTAC
  TTCATCCTCATGTAGGCACTCAGCATCCCCCCATAACTGGGAGTACAGAGCACAGATCAT
  GGTTGTGGGAAGCTGCCCACAACTCAGGCGAGCAGCCTCACTGTCCTCCAGGCTGAGGTG
  CTAGGCTGCTCTTTCCCTGCTCAGAACGCCCAAGGGTGGGAAAGAAGGACCTGAAACTGT
  CAGGCCCACACACCCTGATCCCAGGGCCAAGGCAGATACAGCCTTCACTGGGAGAAGGCA
  CCTGTGGGTGCCCTGCCCTGACCCAGCAATGAAGACA
  ============================================================

Primers for ID: 228
Primer pair 1, product size : 555
  Primer left                            Primer right
  start: 25210417                        start: 25210951
  end  : 25210437                        end  : 25210971
  tm   : 57                              tm   : 57
  seq  : GTGACATCTCGGAAGAATTGG           seq  : TATACAAGGAGGGCCAAAGAG

  >228_primer_pair_1_product
  GTGACATCTCGGAAGAATTGGTAGGAGTCCCCAAGGCCTGCAACAGCTACAACAGGAGCG
  CTGGCTGCCAGTGCCCCAGCCACCAGGTGGGGGTACTTCATCCTCATGTAGGCACTCAGC
  ATCCCCCCATAACTGGGAGTACAGAGCACAGATCATGGTTGTGGGAAGCTGCCCACAACT
  CAGGCGAGCAGCCTCACTGTCCTCCAGGCTGAGGTGCTAGGCTGCTCTTTCCCTGCTCAG
  AACGCCCAAGGGTGGGAAAGAAGGACCTGAAACTGTCAGGCCCACACACCCTGATCCCAG
  GGCCAAGGCAGATACAGCCTTCACTGGGAGAAGGCACCTGTGGGTGCCCTGCCCTGACCC
  AGCAATGAAGACATTGCAGAGACAAAGTCAGAAGGAATTGTCCCACTAGTGGGAACAACA
  TAGCATACACTGCCTATGAGGTCCACTCAAGGAGGGCTTCCAGAAGGAGGTAAAGCTAGA
  CCCCGCCCTTCCACATGTGGGGTAGGCATAGGATGTTGAGACTGTAAGAGACATCTCTTT
  GGCCCTCCTTGTATA
  --------------------

Primer pair 2, product size : 556
  Primer left                            Primer right
  start: 25210417                        start: 25210951
  end  : 25210437                        end  : 25210972
  tm   : 57                              tm   : 61
  seq  : GTGACATCTCGGAAGAATTGG           seq  : CTATACAAGGAGGGCCAAAGAG

  >228_primer_pair_2_product
  GTGACATCTCGGAAGAATTGGTAGGAGTCCCCAAGGCCTGCAACAGCTACAACAGGAGCG
  CTGGCTGCCAGTGCCCCAGCCACCAGGTGGGGGTACTTCATCCTCATGTAGGCACTCAGC
  ATCCCCCCATAACTGGGAGTACAGAGCACAGATCATGGTTGTGGGAAGCTGCCCACAACT
  CAGGCGAGCAGCCTCACTGTCCTCCAGGCTGAGGTGCTAGGCTGCTCTTTCCCTGCTCAG
  AACGCCCAAGGGTGGGAAAGAAGGACCTGAAACTGTCAGGCCCACACACCCTGATCCCAG
  GGCCAAGGCAGATACAGCCTTCACTGGGAGAAGGCACCTGTGGGTGCCCTGCCCTGACCC
  AGCAATGAAGACATTGCAGAGACAAAGTCAGAAGGAATTGTCCCACTAGTGGGAACAACA
  TAGCATACACTGCCTATGAGGTCCACTCAAGGAGGGCTTCCAGAAGGAGGTAAAGCTAGA
  CCCCGCCCTTCCACATGTGGGGTAGGCATAGGATGTTGAGACTGTAAGAGACATCTCTTT
  GGCCCTCCTTGTATAG
  ============================================================

Primers for ID: 229
Primer pair 1, product size : 635
  Primer left                            Primer right
  start: 25210417                        start: 25211030
  end  : 25210437                        end  : 25211051
  tm   : 57                              tm   : 61
  seq  : GTGACATCTCGGAAGAATTGG           seq  : AGATCTGGACCCATGAGAGAAC

  >229_primer_pair_1_product
  GTGACATCTCGGAAGAATTGGTAGGAGTCCCCAAGGCCTGCAACAGCTACAACAGGAGCG
  CTGGCTGCCAGTGCCCCAGCCACCAGGTGGGGGTACTTCATCCTCATGTAGGCACTCAGC
  ATCCCCCCATAACTGGGAGTACAGAGCACAGATCATGGTTGTGGGAAGCTGCCCACAACT
  CAGGCGAGCAGCCTCACTGTCCTCCAGGCTGAGGTGCTAGGCTGCTCTTTCCCTGCTCAG
  AACGCCCAAGGGTGGGAAAGAAGGACCTGAAACTGTCAGGCCCACACACCCTGATCCCAG
  GGCCAAGGCAGATACAGCCTTCACTGGGAGAAGGCACCTGTGGGTGCCCTGCCCTGACCC
  AGCAATGAAGACATTGCAGAGACAAAGTCAGAAGGAATTGTCCCACTAGTGGGAACAACA
  TAGCATACACTGCCTATGAGGTCCACTCAAGGAGGGCTTCCAGAAGGAGGTAAAGCTAGA
  CCCCGCCCTTCCACATGTGGGGTAGGCATAGGATGTTGAGACTGTAAGAGACATCTCTTT
  GGCCCTCCTTGTATAGGGTGTCAATCGGCACAACAGGGTGGAGCCTTAGAGTAGGGTAAG
  ATTAGGACTCTAGGTTCTCTCATGGGTCCAGATCT
  --------------------

Primer pair 2, product size : 555
  Primer left                            Primer right
  start: 25210417                        start: 25210951
  end  : 25210437                        end  : 25210971
  tm   : 57                              tm   : 57
  seq  : GTGACATCTCGGAAGAATTGG           seq  : TATACAAGGAGGGCCAAAGAG

  >229_primer_pair_2_product
  GTGACATCTCGGAAGAATTGGTAGGAGTCCCCAAGGCCTGCAACAGCTACAACAGGAGCG
  CTGGCTGCCAGTGCCCCAGCCACCAGGTGGGGGTACTTCATCCTCATGTAGGCACTCAGC
  ATCCCCCCATAACTGGGAGTACAGAGCACAGATCATGGTTGTGGGAAGCTGCCCACAACT
  CAGGCGAGCAGCCTCACTGTCCTCCAGGCTGAGGTGCTAGGCTGCTCTTTCCCTGCTCAG
  AACGCCCAAGGGTGGGAAAGAAGGACCTGAAACTGTCAGGCCCACACACCCTGATCCCAG
  GGCCAAGGCAGATACAGCCTTCACTGGGAGAAGGCACCTGTGGGTGCCCTGCCCTGACCC
  AGCAATGAAGACATTGCAGAGACAAAGTCAGAAGGAATTGTCCCACTAGTGGGAACAACA
  TAGCATACACTGCCTATGAGGTCCACTCAAGGAGGGCTTCCAGAAGGAGGTAAAGCTAGA
  CCCCGCCCTTCCACATGTGGGGTAGGCATAGGATGTTGAGACTGTAAGAGACATCTCTTT
  GGCCCTCCTTGTATA
  ============================================================

Primers for ID: 230
Primer pair 1, product size : 635
  Primer left                            Primer right
  start: 25210417                        start: 25211030
  end  : 25210437                        end  : 25211051
  tm   : 57                              tm   : 61
  seq  : GTGACATCTCGGAAGAATTGG           seq  : AGATCTGGACCCATGAGAGAAC

  >230_primer_pair_1_product
  GTGACATCTCGGAAGAATTGGTAGGAGTCCCCAAGGCCTGCAACAGCTACAACAGGAGCG
  CTGGCTGCCAGTGCCCCAGCCACCAGGTGGGGGTACTTCATCCTCATGTAGGCACTCAGC
  ATCCCCCCATAACTGGGAGTACAGAGCACAGATCATGGTTGTGGGAAGCTGCCCACAACT
  CAGGCGAGCAGCCTCACTGTCCTCCAGGCTGAGGTGCTAGGCTGCTCTTTCCCTGCTCAG
  AACGCCCAAGGGTGGGAAAGAAGGACCTGAAACTGTCAGGCCCACACACCCTGATCCCAG
  GGCCAAGGCAGATACAGCCTTCACTGGGAGAAGGCACCTGTGGGTGCCCTGCCCTGACCC
  AGCAATGAAGACATTGCAGAGACAAAGTCAGAAGGAATTGTCCCACTAGTGGGAACAACA
  TAGCATACACTGCCTATGAGGTCCACTCAAGGAGGGCTTCCAGAAGGAGGTAAAGCTAGA
  CCCCGCCCTTCCACATGTGGGGTAGGCATAGGATGTTGAGACTGTAAGAGACATCTCTTT
  GGCCCTCCTTGTATAGGGTGTCAATCGGCACAACAGGGTGGAGCCTTAGAGTAGGGTAAG
  ATTAGGACTCTAGGTTCTCTCATGGGTCCAGATCT
  --------------------

Primer pair 2, product size : 555
  Primer left                            Primer right
  start: 25210417                        start: 25210951
  end  : 25210437                        end  : 25210971
  tm   : 57                              tm   : 57
  seq  : GTGACATCTCGGAAGAATTGG           seq  : TATACAAGGAGGGCCAAAGAG

  >230_primer_pair_2_product
  GTGACATCTCGGAAGAATTGGTAGGAGTCCCCAAGGCCTGCAACAGCTACAACAGGAGCG
  CTGGCTGCCAGTGCCCCAGCCACCAGGTGGGGGTACTTCATCCTCATGTAGGCACTCAGC
  ATCCCCCCATAACTGGGAGTACAGAGCACAGATCATGGTTGTGGGAAGCTGCCCACAACT
  CAGGCGAGCAGCCTCACTGTCCTCCAGGCTGAGGTGCTAGGCTGCTCTTTCCCTGCTCAG
  AACGCCCAAGGGTGGGAAAGAAGGACCTGAAACTGTCAGGCCCACACACCCTGATCCCAG
  GGCCAAGGCAGATACAGCCTTCACTGGGAGAAGGCACCTGTGGGTGCCCTGCCCTGACCC
  AGCAATGAAGACATTGCAGAGACAAAGTCAGAAGGAATTGTCCCACTAGTGGGAACAACA
  TAGCATACACTGCCTATGAGGTCCACTCAAGGAGGGCTTCCAGAAGGAGGTAAAGCTAGA
  CCCCGCCCTTCCACATGTGGGGTAGGCATAGGATGTTGAGACTGTAAGAGACATCTCTTT
  GGCCCTCCTTGTATA
  ============================================================

Primers for ID: 240
Primer pair 1, product size : 616
  Primer left                            Primer right
  start: 25211226                        start: 25211821
  end  : 25211245                        end  : 25211841
  tm   : 55                              tm   : 57
  seq  : ATTTCCCATAGTACCGCTGC            seq  : TTCCATCCTTGTTACCCTGTG

  >240_primer_pair_1_product
  ATTTCCCATAGTACCGCTGCAGAAAGCAGGAAGGGATGGCTAATCCACTCCTCGGTGCTC
  CCCACCTCCTTCAACTCAGGGACTGCCAGGAACTGTACAGGTACCCACGTGCTCAGCAAA
  GACAAGCAGGGCCTCCTGCTGGGCTGCCAGTTCCACCATGAAGCCAGAGTTGTTAGCGAA
  GGACCAGATATCCCCCTCATTCCCTGTGTAGAAAAAGATGGGCCCTTCGCCCATCTTCCA
  GAACTTATCTGTTGGAAGTAAATGAGTTTCCATAAGGCCAGGGAAACGCAGGTAGGAACC
  CATGCGGTCGAGCCAGCACTCACCTGACACTAGGAACCGCTGGCCAAAGGTTTTGTTGCC
  GAAACTCTCAAAGTTGAAATGGTCCATGTATTGCTCAAAATAATTCTCATGAAAGTCAGG
  GTCTAGAACTCTGTCGGCTGAGGGCAGGTGCAGAGACTCAGGAGCTGGTTGGGATCATCA
  GGGATCTAGGCGGGTCAGGAGGAAGGGCAGCCAGTCTGTACTCACCTCTGGCCTGGAGGT
  TGCACAGTCCCAGTGACAGCAGCAGGACCAGGATCCAGGAGGGGACACCATGGTCCACAG
  GGTAACAAGGATGGAA
  --------------------

Primer pair 2, product size : 620
  Primer left                            Primer right
  start: 25211222                        start: 25211821
  end  : 25211241                        end  : 25211841
  tm   : 55                              tm   : 57
  seq  : AGCGATTTCCCATAGTACCG            seq  : TTCCATCCTTGTTACCCTGTG

  >240_primer_pair_2_product
  AGCGATTTCCCATAGTACCGCTGCAGAAAGCAGGAAGGGATGGCTAATCCACTCCTCGGT
  GCTCCCCACCTCCTTCAACTCAGGGACTGCCAGGAACTGTACAGGTACCCACGTGCTCAG
  CAAAGACAAGCAGGGCCTCCTGCTGGGCTGCCAGTTCCACCATGAAGCCAGAGTTGTTAG
  CGAAGGACCAGATATCCCCCTCATTCCCTGTGTAGAAAAAGATGGGCCCTTCGCCCATCT
  TCCAGAACTTATCTGTTGGAAGTAAATGAGTTTCCATAAGGCCAGGGAAACGCAGGTAGG
  AACCCATGCGGTCGAGCCAGCACTCACCTGACACTAGGAACCGCTGGCCAAAGGTTTTGT
  TGCCGAAACTCTCAAAGTTGAAATGGTCCATGTATTGCTCAAAATAATTCTCATGAAAGT
  CAGGGTCTAGAACTCTGTCGGCTGAGGGCAGGTGCAGAGACTCAGGAGCTGGTTGGGATC
  ATCAGGGATCTAGGCGGGTCAGGAGGAAGGGCAGCCAGTCTGTACTCACCTCTGGCCTGG
  AGGTTGCACAGTCCCAGTGACAGCAGCAGGACCAGGATCCAGGAGGGGACACCATGGTCC
  ACAGGGTAACAAGGATGGAA
  ============================================================

Primers for ID: 241
Primer pair 1, product size : 596
  Primer left                            Primer right
  start: 25211383                        start: 25211957
  end  : 25211403                        end  : 25211978
  tm   : 57                              tm   : 61
  seq  : ATGAAGCCAGAGTTGTTAGCG           seq  : GCTGATCCTCCAACTAGAATCC

  >241_primer_pair_1_product
  ATGAAGCCAGAGTTGTTAGCGAAGGACCAGATATCCCCCTCATTCCCTGTGTAGAAAAAG
  ATGGGCCCTTCGCCCATCTTCCAGAACTTATCTGTTGGAAGTAAATGAGTTTCCATAAGG
  CCAGGGAAACGCAGGTAGGAACCCATGCGGTCGAGCCAGCACTCACCTGACACTAGGAAC
  CGCTGGCCAAAGGTTTTGTTGCCGAAACTCTCAAAGTTGAAATGGTCCATGTATTGCTCA
  AAATAATTCTCATGAAAGTCAGGGTCTAGAACTCTGTCGGCTGAGGGCAGGTGCAGAGAC
  TCAGGAGCTGGTTGGGATCATCAGGGATCTAGGCGGGTCAGGAGGAAGGGCAGCCAGTCT
  GTACTCACCTCTGGCCTGGAGGTTGCACAGTCCCAGTGACAGCAGCAGGACCAGGATCCA
  GGAGGGGACACCATGGTCCACAGGGTAACAAGGATGGAAGTTCATGCTTGATTCTGAGCC
  GGGCGCTGACTGTCATGTGATTTGGTCACATGACCGACACAACGGGCGGGGCAGCATCAC
  GTGATAGTCTGGCGGGGGCTGTCCTACTGTGGCTGGATTCTAGTTGGAGGATCAGC
  --------------------

Primer pair 2, product size : 597
  Primer left                            Primer right
  start: 25211383                        start: 25211958
  end  : 25211403                        end  : 25211979
  tm   : 57                              tm   : 61
  seq  : ATGAAGCCAGAGTTGTTAGCG           seq  : GGCTGATCCTCCAACTAGAATC

  >241_primer_pair_2_product
  ATGAAGCCAGAGTTGTTAGCGAAGGACCAGATATCCCCCTCATTCCCTGTGTAGAAAAAG
  ATGGGCCCTTCGCCCATCTTCCAGAACTTATCTGTTGGAAGTAAATGAGTTTCCATAAGG
  CCAGGGAAACGCAGGTAGGAACCCATGCGGTCGAGCCAGCACTCACCTGACACTAGGAAC
  CGCTGGCCAAAGGTTTTGTTGCCGAAACTCTCAAAGTTGAAATGGTCCATGTATTGCTCA
  AAATAATTCTCATGAAAGTCAGGGTCTAGAACTCTGTCGGCTGAGGGCAGGTGCAGAGAC
  TCAGGAGCTGGTTGGGATCATCAGGGATCTAGGCGGGTCAGGAGGAAGGGCAGCCAGTCT
  GTACTCACCTCTGGCCTGGAGGTTGCACAGTCCCAGTGACAGCAGCAGGACCAGGATCCA
  GGAGGGGACACCATGGTCCACAGGGTAACAAGGATGGAAGTTCATGCTTGATTCTGAGCC
  GGGCGCTGACTGTCATGTGATTTGGTCACATGACCGACACAACGGGCGGGGCAGCATCAC
  GTGATAGTCTGGCGGGGGCTGTCCTACTGTGGCTGGATTCTAGTTGGAGGATCAGCC
  ============================================================

Primers for ID: 242
Primer pair 1, product size : 620
  Primer left                            Primer right
  start: 25211383                        start: 25211982
  end  : 25211403                        end  : 25212002
  tm   : 57                              tm   : 57
  seq  : ATGAAGCCAGAGTTGTTAGCG           seq  : AACCGGGAAACTGAAGAAGAG

  >242_primer_pair_1_product
  ATGAAGCCAGAGTTGTTAGCGAAGGACCAGATATCCCCCTCATTCCCTGTGTAGAAAAAG
  ATGGGCCCTTCGCCCATCTTCCAGAACTTATCTGTTGGAAGTAAATGAGTTTCCATAAGG
  CCAGGGAAACGCAGGTAGGAACCCATGCGGTCGAGCCAGCACTCACCTGACACTAGGAAC
  CGCTGGCCAAAGGTTTTGTTGCCGAAACTCTCAAAGTTGAAATGGTCCATGTATTGCTCA
  AAATAATTCTCATGAAAGTCAGGGTCTAGAACTCTGTCGGCTGAGGGCAGGTGCAGAGAC
  TCAGGAGCTGGTTGGGATCATCAGGGATCTAGGCGGGTCAGGAGGAAGGGCAGCCAGTCT
  GTACTCACCTCTGGCCTGGAGGTTGCACAGTCCCAGTGACAGCAGCAGGACCAGGATCCA
  GGAGGGGACACCATGGTCCACAGGGTAACAAGGATGGAAGTTCATGCTTGATTCTGAGCC
  GGGCGCTGACTGTCATGTGATTTGGTCACATGACCGACACAACGGGCGGGGCAGCATCAC
  GTGATAGTCTGGCGGGGGCTGTCCTACTGTGGCTGGATTCTAGTTGGAGGATCAGCCTAC
  TCTTCTTCAGTTTCCCGGTT
  --------------------

Primer pair 2, product size : 612
  Primer left                            Primer right
  start: 25211391                        start: 25211982
  end  : 25211412                        end  : 25212002
  tm   : 61                              tm   : 57
  seq  : AGAGTTGTTAGCGAAGGACCAG          seq  : AACCGGGAAACTGAAGAAGAG

  >242_primer_pair_2_product
  AGAGTTGTTAGCGAAGGACCAGATATCCCCCTCATTCCCTGTGTAGAAAAAGATGGGCCC
  TTCGCCCATCTTCCAGAACTTATCTGTTGGAAGTAAATGAGTTTCCATAAGGCCAGGGAA
  ACGCAGGTAGGAACCCATGCGGTCGAGCCAGCACTCACCTGACACTAGGAACCGCTGGCC
  AAAGGTTTTGTTGCCGAAACTCTCAAAGTTGAAATGGTCCATGTATTGCTCAAAATAATT
  CTCATGAAAGTCAGGGTCTAGAACTCTGTCGGCTGAGGGCAGGTGCAGAGACTCAGGAGC
  TGGTTGGGATCATCAGGGATCTAGGCGGGTCAGGAGGAAGGGCAGCCAGTCTGTACTCAC
  CTCTGGCCTGGAGGTTGCACAGTCCCAGTGACAGCAGCAGGACCAGGATCCAGGAGGGGA
  CACCATGGTCCACAGGGTAACAAGGATGGAAGTTCATGCTTGATTCTGAGCCGGGCGCTG
  ACTGTCATGTGATTTGGTCACATGACCGACACAACGGGCGGGGCAGCATCACGTGATAGT
  CTGGCGGGGGCTGTCCTACTGTGGCTGGATTCTAGTTGGAGGATCAGCCTACTCTTCTTC
  AGTTTCCCGGTT
  ============================================================

Primers for ID: 243
Primer pair 1, product size : 620
  Primer left                            Primer right
  start: 25211383                        start: 25211982
  end  : 25211403                        end  : 25212002
  tm   : 57                              tm   : 57
  seq  : ATGAAGCCAGAGTTGTTAGCG           seq  : AACCGGGAAACTGAAGAAGAG

  >243_primer_pair_1_product
  ATGAAGCCAGAGTTGTTAGCGAAGGACCAGATATCCCCCTCATTCCCTGTGTAGAAAAAG
  ATGGGCCCTTCGCCCATCTTCCAGAACTTATCTGTTGGAAGTAAATGAGTTTCCATAAGG
  CCAGGGAAACGCAGGTAGGAACCCATGCGGTCGAGCCAGCACTCACCTGACACTAGGAAC
  CGCTGGCCAAAGGTTTTGTTGCCGAAACTCTCAAAGTTGAAATGGTCCATGTATTGCTCA
  AAATAATTCTCATGAAAGTCAGGGTCTAGAACTCTGTCGGCTGAGGGCAGGTGCAGAGAC
  TCAGGAGCTGGTTGGGATCATCAGGGATCTAGGCGGGTCAGGAGGAAGGGCAGCCAGTCT
  GTACTCACCTCTGGCCTGGAGGTTGCACAGTCCCAGTGACAGCAGCAGGACCAGGATCCA
  GGAGGGGACACCATGGTCCACAGGGTAACAAGGATGGAAGTTCATGCTTGATTCTGAGCC
  GGGCGCTGACTGTCATGTGATTTGGTCACATGACCGACACAACGGGCGGGGCAGCATCAC
  GTGATAGTCTGGCGGGGGCTGTCCTACTGTGGCTGGATTCTAGTTGGAGGATCAGCCTAC
  TCTTCTTCAGTTTCCCGGTT
  --------------------

Primer pair 2, product size : 612
  Primer left                            Primer right
  start: 25211391                        start: 25211982
  end  : 25211412                        end  : 25212002
  tm   : 61                              tm   : 57
  seq  : AGAGTTGTTAGCGAAGGACCAG          seq  : AACCGGGAAACTGAAGAAGAG

  >243_primer_pair_2_product
  AGAGTTGTTAGCGAAGGACCAGATATCCCCCTCATTCCCTGTGTAGAAAAAGATGGGCCC
  TTCGCCCATCTTCCAGAACTTATCTGTTGGAAGTAAATGAGTTTCCATAAGGCCAGGGAA
  ACGCAGGTAGGAACCCATGCGGTCGAGCCAGCACTCACCTGACACTAGGAACCGCTGGCC
  AAAGGTTTTGTTGCCGAAACTCTCAAAGTTGAAATGGTCCATGTATTGCTCAAAATAATT
  CTCATGAAAGTCAGGGTCTAGAACTCTGTCGGCTGAGGGCAGGTGCAGAGACTCAGGAGC
  TGGTTGGGATCATCAGGGATCTAGGCGGGTCAGGAGGAAGGGCAGCCAGTCTGTACTCAC
  CTCTGGCCTGGAGGTTGCACAGTCCCAGTGACAGCAGCAGGACCAGGATCCAGGAGGGGA
  CACCATGGTCCACAGGGTAACAAGGATGGAAGTTCATGCTTGATTCTGAGCCGGGCGCTG
  ACTGTCATGTGATTTGGTCACATGACCGACACAACGGGCGGGGCAGCATCACGTGATAGT
  CTGGCGGGGGCTGTCCTACTGTGGCTGGATTCTAGTTGGAGGATCAGCCTACTCTTCTTC
  AGTTTCCCGGTT
  ============================================================

Primers for ID: 244
Primer pair 1, product size : 630
  Primer left                            Primer right
  start: 25211485                        start: 25212094
  end  : 25211506                        end  : 25212114
  tm   : 57                              tm   : 57
  seq  : AAATGAGTTTCCATAAGGCCAG          seq  : TGGACACTTGGGAGATCATAC

  >244_primer_pair_1_product
  AAATGAGTTTCCATAAGGCCAGGGAAACGCAGGTAGGAACCCATGCGGTCGAGCCAGCAC
  TCACCTGACACTAGGAACCGCTGGCCAAAGGTTTTGTTGCCGAAACTCTCAAAGTTGAAA
  TGGTCCATGTATTGCTCAAAATAATTCTCATGAAAGTCAGGGTCTAGAACTCTGTCGGCT
  GAGGGCAGGTGCAGAGACTCAGGAGCTGGTTGGGATCATCAGGGATCTAGGCGGGTCAGG
  AGGAAGGGCAGCCAGTCTGTACTCACCTCTGGCCTGGAGGTTGCACAGTCCCAGTGACAG
  CAGCAGGACCAGGATCCAGGAGGGGACACCATGGTCCACAGGGTAACAAGGATGGAAGTT
  CATGCTTGATTCTGAGCCGGGCGCTGACTGTCATGTGATTTGGTCACATGACCGACACAA
  CGGGCGGGGCAGCATCACGTGATAGTCTGGCGGGGGCTGTCCTACTGTGGCTGGATTCTA
  GTTGGAGGATCAGCCTACTCTTCTTCAGTTTCCCGGTTCCTCCAAATTTCTGGGCTCCTA
  CTTGTTTCCACAGAGATGGATACTGTGGAGGTCCAGGAAGCAGAGAGATGGCTAAGGCTC
  ATCAGGACCGTATGATCTCCCAAGTGTCCA
  --------------------

Primer pair 2, product size : 555
  Primer left                            Primer right
  start: 25211485                        start: 25212018
  end  : 25211506                        end  : 25212039
  tm   : 57                              tm   : 61
  seq  : AAATGAGTTTCCATAAGGCCAG          seq  : CTCTGTGGAAACAAGTAGGAGC

  >244_primer_pair_2_product
  AAATGAGTTTCCATAAGGCCAGGGAAACGCAGGTAGGAACCCATGCGGTCGAGCCAGCAC
  TCACCTGACACTAGGAACCGCTGGCCAAAGGTTTTGTTGCCGAAACTCTCAAAGTTGAAA
  TGGTCCATGTATTGCTCAAAATAATTCTCATGAAAGTCAGGGTCTAGAACTCTGTCGGCT
  GAGGGCAGGTGCAGAGACTCAGGAGCTGGTTGGGATCATCAGGGATCTAGGCGGGTCAGG
  AGGAAGGGCAGCCAGTCTGTACTCACCTCTGGCCTGGAGGTTGCACAGTCCCAGTGACAG
  CAGCAGGACCAGGATCCAGGAGGGGACACCATGGTCCACAGGGTAACAAGGATGGAAGTT
  CATGCTTGATTCTGAGCCGGGCGCTGACTGTCATGTGATTTGGTCACATGACCGACACAA
  CGGGCGGGGCAGCATCACGTGATAGTCTGGCGGGGGCTGTCCTACTGTGGCTGGATTCTA
  GTTGGAGGATCAGCCTACTCTTCTTCAGTTTCCCGGTTCCTCCAAATTTCTGGGCTCCTA
  CTTGTTTCCACAGAG
  ============================================================

Primers for ID: 245
Primer pair 1, product size : 681
  Primer left                            Primer right
  start: 25211600                        start: 25212260
  end  : 25211621                        end  : 25212280
  tm   : 57                              tm   : 57
  seq  : TGAAATGGTCCATGTATTGCTC          seq  : GAAGCAGAGAGGTCATTTTGG

  >245_primer_pair_1_product
  TGAAATGGTCCATGTATTGCTCAAAATAATTCTCATGAAAGTCAGGGTCTAGAACTCTGT
  CGGCTGAGGGCAGGTGCAGAGACTCAGGAGCTGGTTGGGATCATCAGGGATCTAGGCGGG
  TCAGGAGGAAGGGCAGCCAGTCTGTACTCACCTCTGGCCTGGAGGTTGCACAGTCCCAGT
  GACAGCAGCAGGACCAGGATCCAGGAGGGGACACCATGGTCCACAGGGTAACAAGGATGG
  AAGTTCATGCTTGATTCTGAGCCGGGCGCTGACTGTCATGTGATTTGGTCACATGACCGA
  CACAACGGGCGGGGCAGCATCACGTGATAGTCTGGCGGGGGCTGTCCTACTGTGGCTGGA
  TTCTAGTTGGAGGATCAGCCTACTCTTCTTCAGTTTCCCGGTTCCTCCAAATTTCTGGGC
  TCCTACTTGTTTCCACAGAGATGGATACTGTGGAGGTCCAGGAAGCAGAGAGATGGCTAA
  GGCTCATCAGGACCGTATGATCTCCCAAGTGTCCAGCTACTGAGTACCACAAGGTGATGG
  GTGGGAGGGTCCTCCCACGGAAGGATACCGCAGTCCCTAGGGGTTGCAAGCCCCACATGT
  TCCACTGGCTGCTAGAGCTACCTACTCAATCAGCCCTGGGCATCACCATCAGGTACTCGG
  CCAAAATGACCTCTCTGCTTC
  --------------------

Primer pair 2, product size : 682
  Primer left                            Primer right
  start: 25211600                        start: 25212261
  end  : 25211621                        end  : 25212281
  tm   : 57                              tm   : 57
  seq  : TGAAATGGTCCATGTATTGCTC          seq  : GGAAGCAGAGAGGTCATTTTG

  >245_primer_pair_2_product
  TGAAATGGTCCATGTATTGCTCAAAATAATTCTCATGAAAGTCAGGGTCTAGAACTCTGT
  CGGCTGAGGGCAGGTGCAGAGACTCAGGAGCTGGTTGGGATCATCAGGGATCTAGGCGGG
  TCAGGAGGAAGGGCAGCCAGTCTGTACTCACCTCTGGCCTGGAGGTTGCACAGTCCCAGT
  GACAGCAGCAGGACCAGGATCCAGGAGGGGACACCATGGTCCACAGGGTAACAAGGATGG
  AAGTTCATGCTTGATTCTGAGCCGGGCGCTGACTGTCATGTGATTTGGTCACATGACCGA
  CACAACGGGCGGGGCAGCATCACGTGATAGTCTGGCGGGGGCTGTCCTACTGTGGCTGGA
  TTCTAGTTGGAGGATCAGCCTACTCTTCTTCAGTTTCCCGGTTCCTCCAAATTTCTGGGC
  TCCTACTTGTTTCCACAGAGATGGATACTGTGGAGGTCCAGGAAGCAGAGAGATGGCTAA
  GGCTCATCAGGACCGTATGATCTCCCAAGTGTCCAGCTACTGAGTACCACAAGGTGATGG
  GTGGGAGGGTCCTCCCACGGAAGGATACCGCAGTCCCTAGGGGTTGCAAGCCCCACATGT
  TCCACTGGCTGCTAGAGCTACCTACTCAATCAGCCCTGGGCATCACCATCAGGTACTCGG
  CCAAAATGACCTCTCTGCTTCC
  ============================================================

Primers for ID: 246
Primer pair 1, product size : 695
  Primer left                            Primer right
  start: 25211627                        start: 25212301
  end  : 25211648                        end  : 25212321
  tm   : 57                              tm   : 57
  seq  : AATTCTCATGAAAGTCAGGGTC          seq  : TAATTATGGGCCTGTCTGGTG

  >246_primer_pair_1_product
  AATTCTCATGAAAGTCAGGGTCTAGAACTCTGTCGGCTGAGGGCAGGTGCAGAGACTCAG
  GAGCTGGTTGGGATCATCAGGGATCTAGGCGGGTCAGGAGGAAGGGCAGCCAGTCTGTAC
  TCACCTCTGGCCTGGAGGTTGCACAGTCCCAGTGACAGCAGCAGGACCAGGATCCAGGAG
  GGGACACCATGGTCCACAGGGTAACAAGGATGGAAGTTCATGCTTGATTCTGAGCCGGGC
  GCTGACTGTCATGTGATTTGGTCACATGACCGACACAACGGGCGGGGCAGCATCACGTGA
  TAGTCTGGCGGGGGCTGTCCTACTGTGGCTGGATTCTAGTTGGAGGATCAGCCTACTCTT
  CTTCAGTTTCCCGGTTCCTCCAAATTTCTGGGCTCCTACTTGTTTCCACAGAGATGGATA
  CTGTGGAGGTCCAGGAAGCAGAGAGATGGCTAAGGCTCATCAGGACCGTATGATCTCCCA
  AGTGTCCAGCTACTGAGTACCACAAGGTGATGGGTGGGAGGGTCCTCCCACGGAAGGATA
  CCGCAGTCCCTAGGGGTTGCAAGCCCCACATGTTCCACTGGCTGCTAGAGCTACCTACTC
  AATCAGCCCTGGGCATCACCATCAGGTACTCGGCCAAAATGACCTCTCTGCTTCCAGTCC
  TCAGTTCTGGTCAGCACCAGACAGGCCCATAATTA
  --------------------

Primer pair 2, product size : 697
  Primer left                            Primer right
  start: 25211627                        start: 25212303
  end  : 25211648                        end  : 25212323
  tm   : 57                              tm   : 57
  seq  : AATTCTCATGAAAGTCAGGGTC          seq  : TGTAATTATGGGCCTGTCTGG

  >246_primer_pair_2_product
  AATTCTCATGAAAGTCAGGGTCTAGAACTCTGTCGGCTGAGGGCAGGTGCAGAGACTCAG
  GAGCTGGTTGGGATCATCAGGGATCTAGGCGGGTCAGGAGGAAGGGCAGCCAGTCTGTAC
  TCACCTCTGGCCTGGAGGTTGCACAGTCCCAGTGACAGCAGCAGGACCAGGATCCAGGAG
  GGGACACCATGGTCCACAGGGTAACAAGGATGGAAGTTCATGCTTGATTCTGAGCCGGGC
  GCTGACTGTCATGTGATTTGGTCACATGACCGACACAACGGGCGGGGCAGCATCACGTGA
  TAGTCTGGCGGGGGCTGTCCTACTGTGGCTGGATTCTAGTTGGAGGATCAGCCTACTCTT
  CTTCAGTTTCCCGGTTCCTCCAAATTTCTGGGCTCCTACTTGTTTCCACAGAGATGGATA
  CTGTGGAGGTCCAGGAAGCAGAGAGATGGCTAAGGCTCATCAGGACCGTATGATCTCCCA
  AGTGTCCAGCTACTGAGTACCACAAGGTGATGGGTGGGAGGGTCCTCCCACGGAAGGATA
  CCGCAGTCCCTAGGGGTTGCAAGCCCCACATGTTCCACTGGCTGCTAGAGCTACCTACTC
  AATCAGCCCTGGGCATCACCATCAGGTACTCGGCCAAAATGACCTCTCTGCTTCCAGTCC
  TCAGTTCTGGTCAGCACCAGACAGGCCCATAATTACA
  ============================================================

Primers for ID: 247
Primer pair 1, product size : 563
  Primer left                            Primer right
  start: 25211842                        start: 25212383
  end  : 25211862                        end  : 25212404
  tm   : 57                              tm   : 61
  seq  : GTTCATGCTTGATTCTGAGCC           seq  : GACACTGTTCCTCTGCAACAAC

  >247_primer_pair_1_product
  GTTCATGCTTGATTCTGAGCCGGGCGCTGACTGTCATGTGATTTGGTCACATGACCGACA
  CAACGGGCGGGGCAGCATCACGTGATAGTCTGGCGGGGGCTGTCCTACTGTGGCTGGATT
  CTAGTTGGAGGATCAGCCTACTCTTCTTCAGTTTCCCGGTTCCTCCAAATTTCTGGGCTC
  CTACTTGTTTCCACAGAGATGGATACTGTGGAGGTCCAGGAAGCAGAGAGATGGCTAAGG
  CTCATCAGGACCGTATGATCTCCCAAGTGTCCAGCTACTGAGTACCACAAGGTGATGGGT
  GGGAGGGTCCTCCCACGGAAGGATACCGCAGTCCCTAGGGGTTGCAAGCCCCACATGTTC
  CACTGGCTGCTAGAGCTACCTACTCAATCAGCCCTGGGCATCACCATCAGGTACTCGGCC
  AAAATGACCTCTCTGCTTCCAGTCCTCAGTTCTGGTCAGCACCAGACAGGCCCATAATTA
  CAGAGCCAGGGAAACTGGAACATTTGTCTCCCCTTAGACAGTGGCAGCAGGAAGGTGGGG
  GGTTGTTGCAGAGGAACAGTGTC
  --------------------

Primer pair 2, product size : 586
  Primer left                            Primer right
  start: 25211842                        start: 25212406
  end  : 25211862                        end  : 25212427
  tm   : 57                              tm   : 61
  seq  : GTTCATGCTTGATTCTGAGCC           seq  : AAAGTCCAAGGTCCTCTCTCAG

  >247_primer_pair_2_product
  GTTCATGCTTGATTCTGAGCCGGGCGCTGACTGTCATGTGATTTGGTCACATGACCGACA
  CAACGGGCGGGGCAGCATCACGTGATAGTCTGGCGGGGGCTGTCCTACTGTGGCTGGATT
  CTAGTTGGAGGATCAGCCTACTCTTCTTCAGTTTCCCGGTTCCTCCAAATTTCTGGGCTC
  CTACTTGTTTCCACAGAGATGGATACTGTGGAGGTCCAGGAAGCAGAGAGATGGCTAAGG
  CTCATCAGGACCGTATGATCTCCCAAGTGTCCAGCTACTGAGTACCACAAGGTGATGGGT
  GGGAGGGTCCTCCCACGGAAGGATACCGCAGTCCCTAGGGGTTGCAAGCCCCACATGTTC
  CACTGGCTGCTAGAGCTACCTACTCAATCAGCCCTGGGCATCACCATCAGGTACTCGGCC
  AAAATGACCTCTCTGCTTCCAGTCCTCAGTTCTGGTCAGCACCAGACAGGCCCATAATTA
  CAGAGCCAGGGAAACTGGAACATTTGTCTCCCCTTAGACAGTGGCAGCAGGAAGGTGGGG
  GGTTGTTGCAGAGGAACAGTGTCTCTGAGAGAGGACCTTGGACTTT
  ============================================================

Primers for ID: 248
Primer pair 1, product size : 596
  Primer left                            Primer right
  start: 25211842                        start: 25212417
  end  : 25211862                        end  : 25212437
  tm   : 57                              tm   : 57
  seq  : GTTCATGCTTGATTCTGAGCC           seq  : AGATTCCCAGAAAGTCCAAGG

  >248_primer_pair_1_product
  GTTCATGCTTGATTCTGAGCCGGGCGCTGACTGTCATGTGATTTGGTCACATGACCGACA
  CAACGGGCGGGGCAGCATCACGTGATAGTCTGGCGGGGGCTGTCCTACTGTGGCTGGATT
  CTAGTTGGAGGATCAGCCTACTCTTCTTCAGTTTCCCGGTTCCTCCAAATTTCTGGGCTC
  CTACTTGTTTCCACAGAGATGGATACTGTGGAGGTCCAGGAAGCAGAGAGATGGCTAAGG
  CTCATCAGGACCGTATGATCTCCCAAGTGTCCAGCTACTGAGTACCACAAGGTGATGGGT
  GGGAGGGTCCTCCCACGGAAGGATACCGCAGTCCCTAGGGGTTGCAAGCCCCACATGTTC
  CACTGGCTGCTAGAGCTACCTACTCAATCAGCCCTGGGCATCACCATCAGGTACTCGGCC
  AAAATGACCTCTCTGCTTCCAGTCCTCAGTTCTGGTCAGCACCAGACAGGCCCATAATTA
  CAGAGCCAGGGAAACTGGAACATTTGTCTCCCCTTAGACAGTGGCAGCAGGAAGGTGGGG
  GGTTGTTGCAGAGGAACAGTGTCTCTGAGAGAGGACCTTGGACTTTCTGGGAATCT
  --------------------

Primer pair 2, product size : 594
  Primer left                            Primer right
  start: 25211842                        start: 25212415
  end  : 25211862                        end  : 25212435
  tm   : 57                              tm   : 57
  seq  : GTTCATGCTTGATTCTGAGCC           seq  : ATTCCCAGAAAGTCCAAGGTC

  >248_primer_pair_2_product
  GTTCATGCTTGATTCTGAGCCGGGCGCTGACTGTCATGTGATTTGGTCACATGACCGACA
  CAACGGGCGGGGCAGCATCACGTGATAGTCTGGCGGGGGCTGTCCTACTGTGGCTGGATT
  CTAGTTGGAGGATCAGCCTACTCTTCTTCAGTTTCCCGGTTCCTCCAAATTTCTGGGCTC
  CTACTTGTTTCCACAGAGATGGATACTGTGGAGGTCCAGGAAGCAGAGAGATGGCTAAGG
  CTCATCAGGACCGTATGATCTCCCAAGTGTCCAGCTACTGAGTACCACAAGGTGATGGGT
  GGGAGGGTCCTCCCACGGAAGGATACCGCAGTCCCTAGGGGTTGCAAGCCCCACATGTTC
  CACTGGCTGCTAGAGCTACCTACTCAATCAGCCCTGGGCATCACCATCAGGTACTCGGCC
  AAAATGACCTCTCTGCTTCCAGTCCTCAGTTCTGGTCAGCACCAGACAGGCCCATAATTA
  CAGAGCCAGGGAAACTGGAACATTTGTCTCCCCTTAGACAGTGGCAGCAGGAAGGTGGGG
  GGTTGTTGCAGAGGAACAGTGTCTCTGAGAGAGGACCTTGGACTTTCTGGGAAT
  ============================================================

Primers for ID: 249
Primer pair 1, product size : 596
  Primer left                            Primer right
  start: 25211842                        start: 25212417
  end  : 25211862                        end  : 25212437
  tm   : 57                              tm   : 57
  seq  : GTTCATGCTTGATTCTGAGCC           seq  : AGATTCCCAGAAAGTCCAAGG

  >249_primer_pair_1_product
  GTTCATGCTTGATTCTGAGCCGGGCGCTGACTGTCATGTGATTTGGTCACATGACCGACA
  CAACGGGCGGGGCAGCATCACGTGATAGTCTGGCGGGGGCTGTCCTACTGTGGCTGGATT
  CTAGTTGGAGGATCAGCCTACTCTTCTTCAGTTTCCCGGTTCCTCCAAATTTCTGGGCTC
  CTACTTGTTTCCACAGAGATGGATACTGTGGAGGTCCAGGAAGCAGAGAGATGGCTAAGG
  CTCATCAGGACCGTATGATCTCCCAAGTGTCCAGCTACTGAGTACCACAAGGTGATGGGT
  GGGAGGGTCCTCCCACGGAAGGATACCGCAGTCCCTAGGGGTTGCAAGCCCCACATGTTC
  CACTGGCTGCTAGAGCTACCTACTCAATCAGCCCTGGGCATCACCATCAGGTACTCGGCC
  AAAATGACCTCTCTGCTTCCAGTCCTCAGTTCTGGTCAGCACCAGACAGGCCCATAATTA
  CAGAGCCAGGGAAACTGGAACATTTGTCTCCCCTTAGACAGTGGCAGCAGGAAGGTGGGG
  GGTTGTTGCAGAGGAACAGTGTCTCTGAGAGAGGACCTTGGACTTTCTGGGAATCT
  --------------------

Primer pair 2, product size : 594
  Primer left                            Primer right
  start: 25211842                        start: 25212415
  end  : 25211862                        end  : 25212435
  tm   : 57                              tm   : 57
  seq  : GTTCATGCTTGATTCTGAGCC           seq  : ATTCCCAGAAAGTCCAAGGTC

  >249_primer_pair_2_product
  GTTCATGCTTGATTCTGAGCCGGGCGCTGACTGTCATGTGATTTGGTCACATGACCGACA
  CAACGGGCGGGGCAGCATCACGTGATAGTCTGGCGGGGGCTGTCCTACTGTGGCTGGATT
  CTAGTTGGAGGATCAGCCTACTCTTCTTCAGTTTCCCGGTTCCTCCAAATTTCTGGGCTC
  CTACTTGTTTCCACAGAGATGGATACTGTGGAGGTCCAGGAAGCAGAGAGATGGCTAAGG
  CTCATCAGGACCGTATGATCTCCCAAGTGTCCAGCTACTGAGTACCACAAGGTGATGGGT
  GGGAGGGTCCTCCCACGGAAGGATACCGCAGTCCCTAGGGGTTGCAAGCCCCACATGTTC
  CACTGGCTGCTAGAGCTACCTACTCAATCAGCCCTGGGCATCACCATCAGGTACTCGGCC
  AAAATGACCTCTCTGCTTCCAGTCCTCAGTTCTGGTCAGCACCAGACAGGCCCATAATTA
  CAGAGCCAGGGAAACTGGAACATTTGTCTCCCCTTAGACAGTGGCAGCAGGAAGGTGGGG
  GGTTGTTGCAGAGGAACAGTGTCTCTGAGAGAGGACCTTGGACTTTCTGGGAAT
  ============================================================

Primers for ID: 250
Primer pair 1, product size : 550
  Primer left                            Primer right
  start: 25212011                        start: 25212540
  end  : 25212032                        end  : 25212560
  tm   : 59                              tm   : 57
  seq  : TTTCTGGGCTCCTACTTGTTTC          seq  : ATGAGACACTCTGTGCCAATG

  >250_primer_pair_1_product
  TTTCTGGGCTCCTACTTGTTTCCACAGAGATGGATACTGTGGAGGTCCAGGAAGCAGAGA
  GATGGCTAAGGCTCATCAGGACCGTATGATCTCCCAAGTGTCCAGCTACTGAGTACCACA
  AGGTGATGGGTGGGAGGGTCCTCCCACGGAAGGATACCGCAGTCCCTAGGGGTTGCAAGC
  CCCACATGTTCCACTGGCTGCTAGAGCTACCTACTCAATCAGCCCTGGGCATCACCATCA
  GGTACTCGGCCAAAATGACCTCTCTGCTTCCAGTCCTCAGTTCTGGTCAGCACCAGACAG
  GCCCATAATTACAGAGCCAGGGAAACTGGAACATTTGTCTCCCCTTAGACAGTGGCAGCA
  GGAAGGTGGGGGGTTGTTGCAGAGGAACAGTGTCTCTGAGAGAGGACCTTGGACTTTCTG
  GGAATCTCTGAGCTGCCCGGTTCTCCCCACTGCTGGCACTGTGCCCACAGCCCAAACAGA
  ATGGGGGAGATGGAGGGGCAGGGCTTCTGTGGGAAGCTGCCCTCCACCTCATTGGCACAG
  AGTGTCTCAT
  --------------------

Primer pair 2, product size : 583
  Primer left                            Primer right
  start: 25211978                        start: 25212540
  end  : 25211999                        end  : 25212560
  tm   : 61                              tm   : 57
  seq  : CCTACTCTTCTTCAGTTTCCCG          seq  : ATGAGACACTCTGTGCCAATG

  >250_primer_pair_2_product
  CCTACTCTTCTTCAGTTTCCCGGTTCCTCCAAATTTCTGGGCTCCTACTTGTTTCCACAG
  AGATGGATACTGTGGAGGTCCAGGAAGCAGAGAGATGGCTAAGGCTCATCAGGACCGTAT
  GATCTCCCAAGTGTCCAGCTACTGAGTACCACAAGGTGATGGGTGGGAGGGTCCTCCCAC
  GGAAGGATACCGCAGTCCCTAGGGGTTGCAAGCCCCACATGTTCCACTGGCTGCTAGAGC
  TACCTACTCAATCAGCCCTGGGCATCACCATCAGGTACTCGGCCAAAATGACCTCTCTGC
  TTCCAGTCCTCAGTTCTGGTCAGCACCAGACAGGCCCATAATTACAGAGCCAGGGAAACT
  GGAACATTTGTCTCCCCTTAGACAGTGGCAGCAGGAAGGTGGGGGGTTGTTGCAGAGGAA
  CAGTGTCTCTGAGAGAGGACCTTGGACTTTCTGGGAATCTCTGAGCTGCCCGGTTCTCCC
  CACTGCTGGCACTGTGCCCACAGCCCAAACAGAATGGGGGAGATGGAGGGGCAGGGCTTC
  TGTGGGAAGCTGCCCTCCACCTCATTGGCACAGAGTGTCTCAT
  ============================================================

Primers for ID: 251
Primer pair 1, product size : 550
  Primer left                            Primer right
  start: 25212011                        start: 25212540
  end  : 25212032                        end  : 25212560
  tm   : 59                              tm   : 57
  seq  : TTTCTGGGCTCCTACTTGTTTC          seq  : ATGAGACACTCTGTGCCAATG

  >251_primer_pair_1_product
  TTTCTGGGCTCCTACTTGTTTCCACAGAGATGGATACTGTGGAGGTCCAGGAAGCAGAGA
  GATGGCTAAGGCTCATCAGGACCGTATGATCTCCCAAGTGTCCAGCTACTGAGTACCACA
  AGGTGATGGGTGGGAGGGTCCTCCCACGGAAGGATACCGCAGTCCCTAGGGGTTGCAAGC
  CCCACATGTTCCACTGGCTGCTAGAGCTACCTACTCAATCAGCCCTGGGCATCACCATCA
  GGTACTCGGCCAAAATGACCTCTCTGCTTCCAGTCCTCAGTTCTGGTCAGCACCAGACAG
  GCCCATAATTACAGAGCCAGGGAAACTGGAACATTTGTCTCCCCTTAGACAGTGGCAGCA
  GGAAGGTGGGGGGTTGTTGCAGAGGAACAGTGTCTCTGAGAGAGGACCTTGGACTTTCTG
  GGAATCTCTGAGCTGCCCGGTTCTCCCCACTGCTGGCACTGTGCCCACAGCCCAAACAGA
  ATGGGGGAGATGGAGGGGCAGGGCTTCTGTGGGAAGCTGCCCTCCACCTCATTGGCACAG
  AGTGTCTCAT
  --------------------

Primer pair 2, product size : 662
  Primer left                            Primer right
  start: 25212011                        start: 25212653
  end  : 25212032                        end  : 25212672
  tm   : 59                              tm   : 55
  seq  : TTTCTGGGCTCCTACTTGTTTC          seq  : ATGGACATGTTCCACTCACG

  >251_primer_pair_2_product
  TTTCTGGGCTCCTACTTGTTTCCACAGAGATGGATACTGTGGAGGTCCAGGAAGCAGAGA
  GATGGCTAAGGCTCATCAGGACCGTATGATCTCCCAAGTGTCCAGCTACTGAGTACCACA
  AGGTGATGGGTGGGAGGGTCCTCCCACGGAAGGATACCGCAGTCCCTAGGGGTTGCAAGC
  CCCACATGTTCCACTGGCTGCTAGAGCTACCTACTCAATCAGCCCTGGGCATCACCATCA
  GGTACTCGGCCAAAATGACCTCTCTGCTTCCAGTCCTCAGTTCTGGTCAGCACCAGACAG
  GCCCATAATTACAGAGCCAGGGAAACTGGAACATTTGTCTCCCCTTAGACAGTGGCAGCA
  GGAAGGTGGGGGGTTGTTGCAGAGGAACAGTGTCTCTGAGAGAGGACCTTGGACTTTCTG
  GGAATCTCTGAGCTGCCCGGTTCTCCCCACTGCTGGCACTGTGCCCACAGCCCAAACAGA
  ATGGGGGAGATGGAGGGGCAGGGCTTCTGTGGGAAGCTGCCCTCCACCTCATTGGCACAG
  AGTGTCTCATTGCAGAGAGAAAAAAGGACCAGTTTTCTCTCTGGCACCCAGGTCTGGAAG
  AGGAGTGACATCCACGGAAGTTGGTGACTTGGACTGGCTGGCCGTGAGTGGAACATGTCC
  AT
  ============================================================

Primers for ID: 252
Primer pair 1, product size : 527
  Primer left                            Primer right
  start: 25209826                        start: 25210331
  end  : 25209847                        end  : 25210352
  tm   : 61                              tm   : 59
  seq  : AGCAGAATATCTCAGTGGAGGC          seq  : TCTTCTCCAGGACTTTTATGGC

  >252_primer_pair_1_product
  AGCAGAATATCTCAGTGGAGGCCCCTTTCACAGAGCGTGGGTCAGGGCTGCTAGCTTCCA
  GGACACAACAGCAGATAGTGTCTGATGGCATGAAAGCAGATAGCTACAAGGCTCTTGGAC
  CAGGCTAGCACTGGGTCCTGCACCCAGGGAGAGCCACCTCACCTTGACAGGGTTGGCAGG
  AAGGGGCCCTAGAAAGTCAGTAGGATACGGGTAGTCCATCATGGCGAGCACAGTAAATGC
  ATTTCGGGCAAACCCAAAGAGCTGAGTCAGGTCCTTTGGGCTGGAAAGTGATTGACAGGT
  ACCAAAGTTCTGGCTGATGGTGTCATAGGCTGGGAAGAGAGAGGCCAGGAGAAAAGGCTG
  AGGAAACTGCTGGCAAATGTGAAGGGCAAGAATGAATGCCCAAGGTGGGCAGCAGGTGAG
  GAAAGAGTCCCTCACCTCCCTGGAGGAACAAGTCTTTGATTTGCTGAAAGGCATCCCGCA
  CAGCCTGGGCGCACTTGGGACTCTGGCCATAAAAGTCCTGGAGAAGA
  --------------------

Primer pair 2, product size : 529
  Primer left                            Primer right
  start: 25209824                        start: 25210331
  end  : 25209845                        end  : 25210352
  tm   : 61                              tm   : 59
  seq  : GCAGCAGAATATCTCAGTGGAG          seq  : TCTTCTCCAGGACTTTTATGGC

  >252_primer_pair_2_product
  GCAGCAGAATATCTCAGTGGAGGCCCCTTTCACAGAGCGTGGGTCAGGGCTGCTAGCTTC
  CAGGACACAACAGCAGATAGTGTCTGATGGCATGAAAGCAGATAGCTACAAGGCTCTTGG
  ACCAGGCTAGCACTGGGTCCTGCACCCAGGGAGAGCCACCTCACCTTGACAGGGTTGGCA
  GGAAGGGGCCCTAGAAAGTCAGTAGGATACGGGTAGTCCATCATGGCGAGCACAGTAAAT
  GCATTTCGGGCAAACCCAAAGAGCTGAGTCAGGTCCTTTGGGCTGGAAAGTGATTGACAG
  GTACCAAAGTTCTGGCTGATGGTGTCATAGGCTGGGAAGAGAGAGGCCAGGAGAAAAGGC
  TGAGGAAACTGCTGGCAAATGTGAAGGGCAAGAATGAATGCCCAAGGTGGGCAGCAGGTG
  AGGAAAGAGTCCCTCACCTCCCTGGAGGAACAAGTCTTTGATTTGCTGAAAGGCATCCCG
  CACAGCCTGGGCGCACTTGGGACTCTGGCCATAAAAGTCCTGGAGAAGA
  ============================================================

Primers for ID: 253
Primer pair 1, product size : 529
  Primer left                            Primer right
  start: 25209909                        start: 25210417
  end  : 25209929                        end  : 25210437
  tm   : 57                              tm   : 57
  seq  : GATGGCATGAAAGCAGATAGC           seq  : CCAATTCTTCCGAGATGTCAC

  >253_primer_pair_1_product
  GATGGCATGAAAGCAGATAGCTACAAGGCTCTTGGACCAGGCTAGCACTGGGTCCTGCAC
  CCAGGGAGAGCCACCTCACCTTGACAGGGTTGGCAGGAAGGGGCCCTAGAAAGTCAGTAG
  GATACGGGTAGTCCATCATGGCGAGCACAGTAAATGCATTTCGGGCAAACCCAAAGAGCT
  GAGTCAGGTCCTTTGGGCTGGAAAGTGATTGACAGGTACCAAAGTTCTGGCTGATGGTGT
  CATAGGCTGGGAAGAGAGAGGCCAGGAGAAAAGGCTGAGGAAACTGCTGGCAAATGTGAA
  GGGCAAGAATGAATGCCCAAGGTGGGCAGCAGGTGAGGAAAGAGTCCCTCACCTCCCTGG
  AGGAACAAGTCTTTGATTTGCTGAAAGGCATCCCGCACAGCCTGGGCGCACTTGGGACTC
  TGGCCATAAAAGTCCTGGAGAAGAGACCAAGGTTGCTGCTGCCATTCTTGCACTGGCCTG
  GGGTACCCAAGTCCCCTCACTCACCGCTGTGACATCTCGGAAGAATTGG
  --------------------

Primer pair 2, product size : 528
  Primer left                            Primer right
  start: 25209909                        start: 25210415
  end  : 25209929                        end  : 25210436
  tm   : 57                              tm   : 59
  seq  : GATGGCATGAAAGCAGATAGC           seq  : CAATTCTTCCGAGATGTCACAG

  >253_primer_pair_2_product
  GATGGCATGAAAGCAGATAGCTACAAGGCTCTTGGACCAGGCTAGCACTGGGTCCTGCAC
  CCAGGGAGAGCCACCTCACCTTGACAGGGTTGGCAGGAAGGGGCCCTAGAAAGTCAGTAG
  GATACGGGTAGTCCATCATGGCGAGCACAGTAAATGCATTTCGGGCAAACCCAAAGAGCT
  GAGTCAGGTCCTTTGGGCTGGAAAGTGATTGACAGGTACCAAAGTTCTGGCTGATGGTGT
  CATAGGCTGGGAAGAGAGAGGCCAGGAGAAAAGGCTGAGGAAACTGCTGGCAAATGTGAA
  GGGCAAGAATGAATGCCCAAGGTGGGCAGCAGGTGAGGAAAGAGTCCCTCACCTCCCTGG
  AGGAACAAGTCTTTGATTTGCTGAAAGGCATCCCGCACAGCCTGGGCGCACTTGGGACTC
  TGGCCATAAAAGTCCTGGAGAAGAGACCAAGGTTGCTGCTGCCATTCTTGCACTGGCCTG
  GGGTACCCAAGTCCCCTCACTCACCGCTGTGACATCTCGGAAGAATTG
  ============================================================

Primers for ID: 254
Primer pair 1, product size : 529
  Primer left                            Primer right
  start: 25209909                        start: 25210417
  end  : 25209929                        end  : 25210437
  tm   : 57                              tm   : 57
  seq  : GATGGCATGAAAGCAGATAGC           seq  : CCAATTCTTCCGAGATGTCAC

  >254_primer_pair_1_product
  GATGGCATGAAAGCAGATAGCTACAAGGCTCTTGGACCAGGCTAGCACTGGGTCCTGCAC
  CCAGGGAGAGCCACCTCACCTTGACAGGGTTGGCAGGAAGGGGCCCTAGAAAGTCAGTAG
  GATACGGGTAGTCCATCATGGCGAGCACAGTAAATGCATTTCGGGCAAACCCAAAGAGCT
  GAGTCAGGTCCTTTGGGCTGGAAAGTGATTGACAGGTACCAAAGTTCTGGCTGATGGTGT
  CATAGGCTGGGAAGAGAGAGGCCAGGAGAAAAGGCTGAGGAAACTGCTGGCAAATGTGAA
  GGGCAAGAATGAATGCCCAAGGTGGGCAGCAGGTGAGGAAAGAGTCCCTCACCTCCCTGG
  AGGAACAAGTCTTTGATTTGCTGAAAGGCATCCCGCACAGCCTGGGCGCACTTGGGACTC
  TGGCCATAAAAGTCCTGGAGAAGAGACCAAGGTTGCTGCTGCCATTCTTGCACTGGCCTG
  GGGTACCCAAGTCCCCTCACTCACCGCTGTGACATCTCGGAAGAATTGG
  --------------------

Primer pair 2, product size : 528
  Primer left                            Primer right
  start: 25209909                        start: 25210415
  end  : 25209929                        end  : 25210436
  tm   : 57                              tm   : 59
  seq  : GATGGCATGAAAGCAGATAGC           seq  : CAATTCTTCCGAGATGTCACAG

  >254_primer_pair_2_product
  GATGGCATGAAAGCAGATAGCTACAAGGCTCTTGGACCAGGCTAGCACTGGGTCCTGCAC
  CCAGGGAGAGCCACCTCACCTTGACAGGGTTGGCAGGAAGGGGCCCTAGAAAGTCAGTAG
  GATACGGGTAGTCCATCATGGCGAGCACAGTAAATGCATTTCGGGCAAACCCAAAGAGCT
  GAGTCAGGTCCTTTGGGCTGGAAAGTGATTGACAGGTACCAAAGTTCTGGCTGATGGTGT
  CATAGGCTGGGAAGAGAGAGGCCAGGAGAAAAGGCTGAGGAAACTGCTGGCAAATGTGAA
  GGGCAAGAATGAATGCCCAAGGTGGGCAGCAGGTGAGGAAAGAGTCCCTCACCTCCCTGG
  AGGAACAAGTCTTTGATTTGCTGAAAGGCATCCCGCACAGCCTGGGCGCACTTGGGACTC
  TGGCCATAAAAGTCCTGGAGAAGAGACCAAGGTTGCTGCTGCCATTCTTGCACTGGCCTG
  GGGTACCCAAGTCCCCTCACTCACCGCTGTGACATCTCGGAAGAATTG
  ============================================================

Primers for ID: 255
Primer pair 1, product size : 563
  Primer left                            Primer right
  start: 25210016                        start: 25210558
  end  : 25210036                        end  : 25210578
  tm   : 57                              tm   : 57
  seq  : AGAAAGTCAGTAGGATACGGG           seq  : ACAACCATGATCTGTGCTCTG

  >255_primer_pair_1_product
  AGAAAGTCAGTAGGATACGGGTAGTCCATCATGGCGAGCACAGTAAATGCATTTCGGGCA
  AACCCAAAGAGCTGAGTCAGGTCCTTTGGGCTGGAAAGTGATTGACAGGTACCAAAGTTC
  TGGCTGATGGTGTCATAGGCTGGGAAGAGAGAGGCCAGGAGAAAAGGCTGAGGAAACTGC
  TGGCAAATGTGAAGGGCAAGAATGAATGCCCAAGGTGGGCAGCAGGTGAGGAAAGAGTCC
  CTCACCTCCCTGGAGGAACAAGTCTTTGATTTGCTGAAAGGCATCCCGCACAGCCTGGGC
  GCACTTGGGACTCTGGCCATAAAAGTCCTGGAGAAGAGACCAAGGTTGCTGCTGCCATTC
  TTGCACTGGCCTGGGGTACCCAAGTCCCCTCACTCACCGCTGTGACATCTCGGAAGAATT
  GGTAGGAGTCCCCAAGGCCTGCAACAGCTACAACAGGAGCGCTGGCTGCCAGTGCCCCAG
  CCACCAGGTGGGGGTACTTCATCCTCATGTAGGCACTCAGCATCCCCCCATAACTGGGAG
  TACAGAGCACAGATCATGGTTGT
  --------------------

Primer pair 2, product size : 549
  Primer left                            Primer right
  start: 25210016                        start: 25210543
  end  : 25210036                        end  : 25210564
  tm   : 57                              tm   : 61
  seq  : AGAAAGTCAGTAGGATACGGG           seq  : TGCTCTGTACTCCCAGTTATGG

  >255_primer_pair_2_product
  AGAAAGTCAGTAGGATACGGGTAGTCCATCATGGCGAGCACAGTAAATGCATTTCGGGCA
  AACCCAAAGAGCTGAGTCAGGTCCTTTGGGCTGGAAAGTGATTGACAGGTACCAAAGTTC
  TGGCTGATGGTGTCATAGGCTGGGAAGAGAGAGGCCAGGAGAAAAGGCTGAGGAAACTGC
  TGGCAAATGTGAAGGGCAAGAATGAATGCCCAAGGTGGGCAGCAGGTGAGGAAAGAGTCC
  CTCACCTCCCTGGAGGAACAAGTCTTTGATTTGCTGAAAGGCATCCCGCACAGCCTGGGC
  GCACTTGGGACTCTGGCCATAAAAGTCCTGGAGAAGAGACCAAGGTTGCTGCTGCCATTC
  TTGCACTGGCCTGGGGTACCCAAGTCCCCTCACTCACCGCTGTGACATCTCGGAAGAATT
  GGTAGGAGTCCCCAAGGCCTGCAACAGCTACAACAGGAGCGCTGGCTGCCAGTGCCCCAG
  CCACCAGGTGGGGGTACTTCATCCTCATGTAGGCACTCAGCATCCCCCCATAACTGGGAG
  TACAGAGCA
  ============================================================

Primers for ID: 256
Primer pair 1, product size : 562
  Primer left                            Primer right
  start: 25210129                        start: 25210670
  end  : 25210149                        end  : 25210690
  tm   : 57                              tm   : 57
  seq  : AAAGTTCTGGCTGATGGTGTC           seq  : AGTTTCAGGTCCTTCTTTCCC

  >256_primer_pair_1_product
  AAAGTTCTGGCTGATGGTGTCATAGGCTGGGAAGAGAGAGGCCAGGAGAAAAGGCTGAGG
  AAACTGCTGGCAAATGTGAAGGGCAAGAATGAATGCCCAAGGTGGGCAGCAGGTGAGGAA
  AGAGTCCCTCACCTCCCTGGAGGAACAAGTCTTTGATTTGCTGAAAGGCATCCCGCACAG
  CCTGGGCGCACTTGGGACTCTGGCCATAAAAGTCCTGGAGAAGAGACCAAGGTTGCTGCT
  GCCATTCTTGCACTGGCCTGGGGTACCCAAGTCCCCTCACTCACCGCTGTGACATCTCGG
  AAGAATTGGTAGGAGTCCCCAAGGCCTGCAACAGCTACAACAGGAGCGCTGGCTGCCAGT
  GCCCCAGCCACCAGGTGGGGGTACTTCATCCTCATGTAGGCACTCAGCATCCCCCCATAA
  CTGGGAGTACAGAGCACAGATCATGGTTGTGGGAAGCTGCCCACAACTCAGGCGAGCAGC
  CTCACTGTCCTCCAGGCTGAGGTGCTAGGCTGCTCTTTCCCTGCTCAGAACGCCCAAGGG
  TGGGAAAGAAGGACCTGAAACT
  --------------------

Primer pair 2, product size : 558
  Primer left                            Primer right
  start: 25210133                        start: 25210670
  end  : 25210153                        end  : 25210690
  tm   : 57                              tm   : 57
  seq  : TTCTGGCTGATGGTGTCATAG           seq  : AGTTTCAGGTCCTTCTTTCCC

  >256_primer_pair_2_product
  TTCTGGCTGATGGTGTCATAGGCTGGGAAGAGAGAGGCCAGGAGAAAAGGCTGAGGAAAC
  TGCTGGCAAATGTGAAGGGCAAGAATGAATGCCCAAGGTGGGCAGCAGGTGAGGAAAGAG
  TCCCTCACCTCCCTGGAGGAACAAGTCTTTGATTTGCTGAAAGGCATCCCGCACAGCCTG
  GGCGCACTTGGGACTCTGGCCATAAAAGTCCTGGAGAAGAGACCAAGGTTGCTGCTGCCA
  TTCTTGCACTGGCCTGGGGTACCCAAGTCCCCTCACTCACCGCTGTGACATCTCGGAAGA
  ATTGGTAGGAGTCCCCAAGGCCTGCAACAGCTACAACAGGAGCGCTGGCTGCCAGTGCCC
  CAGCCACCAGGTGGGGGTACTTCATCCTCATGTAGGCACTCAGCATCCCCCCATAACTGG
  GAGTACAGAGCACAGATCATGGTTGTGGGAAGCTGCCCACAACTCAGGCGAGCAGCCTCA
  CTGTCCTCCAGGCTGAGGTGCTAGGCTGCTCTTTCCCTGCTCAGAACGCCCAAGGGTGGG
  AAAGAAGGACCTGAAACT
  ============================================================

Primers for ID: 257
Primer pair 1, product size : 562
  Primer left                            Primer right
  start: 25210129                        start: 25210670
  end  : 25210149                        end  : 25210690
  tm   : 57                              tm   : 57
  seq  : AAAGTTCTGGCTGATGGTGTC           seq  : AGTTTCAGGTCCTTCTTTCCC

  >257_primer_pair_1_product
  AAAGTTCTGGCTGATGGTGTCATAGGCTGGGAAGAGAGAGGCCAGGAGAAAAGGCTGAGG
  AAACTGCTGGCAAATGTGAAGGGCAAGAATGAATGCCCAAGGTGGGCAGCAGGTGAGGAA
  AGAGTCCCTCACCTCCCTGGAGGAACAAGTCTTTGATTTGCTGAAAGGCATCCCGCACAG
  CCTGGGCGCACTTGGGACTCTGGCCATAAAAGTCCTGGAGAAGAGACCAAGGTTGCTGCT
  GCCATTCTTGCACTGGCCTGGGGTACCCAAGTCCCCTCACTCACCGCTGTGACATCTCGG
  AAGAATTGGTAGGAGTCCCCAAGGCCTGCAACAGCTACAACAGGAGCGCTGGCTGCCAGT
  GCCCCAGCCACCAGGTGGGGGTACTTCATCCTCATGTAGGCACTCAGCATCCCCCCATAA
  CTGGGAGTACAGAGCACAGATCATGGTTGTGGGAAGCTGCCCACAACTCAGGCGAGCAGC
  CTCACTGTCCTCCAGGCTGAGGTGCTAGGCTGCTCTTTCCCTGCTCAGAACGCCCAAGGG
  TGGGAAAGAAGGACCTGAAACT
  --------------------

Primer pair 2, product size : 558
  Primer left                            Primer right
  start: 25210133                        start: 25210670
  end  : 25210153                        end  : 25210690
  tm   : 57                              tm   : 57
  seq  : TTCTGGCTGATGGTGTCATAG           seq  : AGTTTCAGGTCCTTCTTTCCC

  >257_primer_pair_2_product
  TTCTGGCTGATGGTGTCATAGGCTGGGAAGAGAGAGGCCAGGAGAAAAGGCTGAGGAAAC
  TGCTGGCAAATGTGAAGGGCAAGAATGAATGCCCAAGGTGGGCAGCAGGTGAGGAAAGAG
  TCCCTCACCTCCCTGGAGGAACAAGTCTTTGATTTGCTGAAAGGCATCCCGCACAGCCTG
  GGCGCACTTGGGACTCTGGCCATAAAAGTCCTGGAGAAGAGACCAAGGTTGCTGCTGCCA
  TTCTTGCACTGGCCTGGGGTACCCAAGTCCCCTCACTCACCGCTGTGACATCTCGGAAGA
  ATTGGTAGGAGTCCCCAAGGCCTGCAACAGCTACAACAGGAGCGCTGGCTGCCAGTGCCC
  CAGCCACCAGGTGGGGGTACTTCATCCTCATGTAGGCACTCAGCATCCCCCCATAACTGG
  GAGTACAGAGCACAGATCATGGTTGTGGGAAGCTGCCCACAACTCAGGCGAGCAGCCTCA
  CTGTCCTCCAGGCTGAGGTGCTAGGCTGCTCTTTCCCTGCTCAGAACGCCCAAGGGTGGG
  AAAGAAGGACCTGAAACT
  ============================================================

Primers for ID: 258
Primer pair 1, product size : 544
  Primer left                            Primer right
  start: 25210277                        start: 25210799
  end  : 25210297                        end  : 25210820
  tm   : 55                              tm   : 59
  seq  : GTCTTTGATTTGCTGAAAGGC           seq  : GGGACAATTCCTTCTGACTTTG

  >258_primer_pair_1_product
  GTCTTTGATTTGCTGAAAGGCATCCCGCACAGCCTGGGCGCACTTGGGACTCTGGCCATA
  AAAGTCCTGGAGAAGAGACCAAGGTTGCTGCTGCCATTCTTGCACTGGCCTGGGGTACCC
  AAGTCCCCTCACTCACCGCTGTGACATCTCGGAAGAATTGGTAGGAGTCCCCAAGGCCTG
  CAACAGCTACAACAGGAGCGCTGGCTGCCAGTGCCCCAGCCACCAGGTGGGGGTACTTCA
  TCCTCATGTAGGCACTCAGCATCCCCCCATAACTGGGAGTACAGAGCACAGATCATGGTT
  GTGGGAAGCTGCCCACAACTCAGGCGAGCAGCCTCACTGTCCTCCAGGCTGAGGTGCTAG
  GCTGCTCTTTCCCTGCTCAGAACGCCCAAGGGTGGGAAAGAAGGACCTGAAACTGTCAGG
  CCCACACACCCTGATCCCAGGGCCAAGGCAGATACAGCCTTCACTGGGAGAAGGCACCTG
  TGGGTGCCCTGCCCTGACCCAGCAATGAAGACATTGCAGAGACAAAGTCAGAAGGAATTG
  TCCC
  --------------------

Primer pair 2, product size : 608
  Primer left                            Primer right
  start: 25210213                        start: 25210799
  end  : 25210232                        end  : 25210820
  tm   : 53                              tm   : 59
  seq  : AAGAATGAATGCCCAAGGTG            seq  : GGGACAATTCCTTCTGACTTTG

  >258_primer_pair_2_product
  AAGAATGAATGCCCAAGGTGGGCAGCAGGTGAGGAAAGAGTCCCTCACCTCCCTGGAGGA
  ACAAGTCTTTGATTTGCTGAAAGGCATCCCGCACAGCCTGGGCGCACTTGGGACTCTGGC
  CATAAAAGTCCTGGAGAAGAGACCAAGGTTGCTGCTGCCATTCTTGCACTGGCCTGGGGT
  ACCCAAGTCCCCTCACTCACCGCTGTGACATCTCGGAAGAATTGGTAGGAGTCCCCAAGG
  CCTGCAACAGCTACAACAGGAGCGCTGGCTGCCAGTGCCCCAGCCACCAGGTGGGGGTAC
  TTCATCCTCATGTAGGCACTCAGCATCCCCCCATAACTGGGAGTACAGAGCACAGATCAT
  GGTTGTGGGAAGCTGCCCACAACTCAGGCGAGCAGCCTCACTGTCCTCCAGGCTGAGGTG
  CTAGGCTGCTCTTTCCCTGCTCAGAACGCCCAAGGGTGGGAAAGAAGGACCTGAAACTGT
  CAGGCCCACACACCCTGATCCCAGGGCCAAGGCAGATACAGCCTTCACTGGGAGAAGGCA
  CCTGTGGGTGCCCTGCCCTGACCCAGCAATGAAGACATTGCAGAGACAAAGTCAGAAGGA
  ATTGTCCC
  ============================================================

Primers for ID: 259
Primer pair 1, product size : 544
  Primer left                            Primer right
  start: 25210277                        start: 25210799
  end  : 25210297                        end  : 25210820
  tm   : 55                              tm   : 59
  seq  : GTCTTTGATTTGCTGAAAGGC           seq  : GGGACAATTCCTTCTGACTTTG

  >259_primer_pair_1_product
  GTCTTTGATTTGCTGAAAGGCATCCCGCACAGCCTGGGCGCACTTGGGACTCTGGCCATA
  AAAGTCCTGGAGAAGAGACCAAGGTTGCTGCTGCCATTCTTGCACTGGCCTGGGGTACCC
  AAGTCCCCTCACTCACCGCTGTGACATCTCGGAAGAATTGGTAGGAGTCCCCAAGGCCTG
  CAACAGCTACAACAGGAGCGCTGGCTGCCAGTGCCCCAGCCACCAGGTGGGGGTACTTCA
  TCCTCATGTAGGCACTCAGCATCCCCCCATAACTGGGAGTACAGAGCACAGATCATGGTT
  GTGGGAAGCTGCCCACAACTCAGGCGAGCAGCCTCACTGTCCTCCAGGCTGAGGTGCTAG
  GCTGCTCTTTCCCTGCTCAGAACGCCCAAGGGTGGGAAAGAAGGACCTGAAACTGTCAGG
  CCCACACACCCTGATCCCAGGGCCAAGGCAGATACAGCCTTCACTGGGAGAAGGCACCTG
  TGGGTGCCCTGCCCTGACCCAGCAATGAAGACATTGCAGAGACAAAGTCAGAAGGAATTG
  TCCC
  --------------------

Primer pair 2, product size : 545
  Primer left                            Primer right
  start: 25210276                        start: 25210799
  end  : 25210297                        end  : 25210820
  tm   : 57                              tm   : 59
  seq  : AGTCTTTGATTTGCTGAAAGGC          seq  : GGGACAATTCCTTCTGACTTTG

  >259_primer_pair_2_product
  AGTCTTTGATTTGCTGAAAGGCATCCCGCACAGCCTGGGCGCACTTGGGACTCTGGCCAT
  AAAAGTCCTGGAGAAGAGACCAAGGTTGCTGCTGCCATTCTTGCACTGGCCTGGGGTACC
  CAAGTCCCCTCACTCACCGCTGTGACATCTCGGAAGAATTGGTAGGAGTCCCCAAGGCCT
  GCAACAGCTACAACAGGAGCGCTGGCTGCCAGTGCCCCAGCCACCAGGTGGGGGTACTTC
  ATCCTCATGTAGGCACTCAGCATCCCCCCATAACTGGGAGTACAGAGCACAGATCATGGT
  TGTGGGAAGCTGCCCACAACTCAGGCGAGCAGCCTCACTGTCCTCCAGGCTGAGGTGCTA
  GGCTGCTCTTTCCCTGCTCAGAACGCCCAAGGGTGGGAAAGAAGGACCTGAAACTGTCAG
  GCCCACACACCCTGATCCCAGGGCCAAGGCAGATACAGCCTTCACTGGGAGAAGGCACCT
  GTGGGTGCCCTGCCCTGACCCAGCAATGAAGACATTGCAGAGACAAAGTCAGAAGGAATT
  GTCCC
  ============================================================

Primers for ID: 260
Primer pair 1, product size : 555
  Primer left                            Primer right
  start: 25210417                        start: 25210951
  end  : 25210437                        end  : 25210971
  tm   : 57                              tm   : 57
  seq  : GTGACATCTCGGAAGAATTGG           seq  : TATACAAGGAGGGCCAAAGAG

  >260_primer_pair_1_product
  GTGACATCTCGGAAGAATTGGTAGGAGTCCCCAAGGCCTGCAACAGCTACAACAGGAGCG
  CTGGCTGCCAGTGCCCCAGCCACCAGGTGGGGGTACTTCATCCTCATGTAGGCACTCAGC
  ATCCCCCCATAACTGGGAGTACAGAGCACAGATCATGGTTGTGGGAAGCTGCCCACAACT
  CAGGCGAGCAGCCTCACTGTCCTCCAGGCTGAGGTGCTAGGCTGCTCTTTCCCTGCTCAG
  AACGCCCAAGGGTGGGAAAGAAGGACCTGAAACTGTCAGGCCCACACACCCTGATCCCAG
  GGCCAAGGCAGATACAGCCTTCACTGGGAGAAGGCACCTGTGGGTGCCCTGCCCTGACCC
  AGCAATGAAGACATTGCAGAGACAAAGTCAGAAGGAATTGTCCCACTAGTGGGAACAACA
  TAGCATACACTGCCTATGAGGTCCACTCAAGGAGGGCTTCCAGAAGGAGGTAAAGCTAGA
  CCCCGCCCTTCCACATGTGGGGTAGGCATAGGATGTTGAGACTGTAAGAGACATCTCTTT
  GGCCCTCCTTGTATA
  --------------------

Primer pair 2, product size : 556
  Primer left                            Primer right
  start: 25210417                        start: 25210951
  end  : 25210437                        end  : 25210972
  tm   : 57                              tm   : 61
  seq  : GTGACATCTCGGAAGAATTGG           seq  : CTATACAAGGAGGGCCAAAGAG

  >260_primer_pair_2_product
  GTGACATCTCGGAAGAATTGGTAGGAGTCCCCAAGGCCTGCAACAGCTACAACAGGAGCG
  CTGGCTGCCAGTGCCCCAGCCACCAGGTGGGGGTACTTCATCCTCATGTAGGCACTCAGC
  ATCCCCCCATAACTGGGAGTACAGAGCACAGATCATGGTTGTGGGAAGCTGCCCACAACT
  CAGGCGAGCAGCCTCACTGTCCTCCAGGCTGAGGTGCTAGGCTGCTCTTTCCCTGCTCAG
  AACGCCCAAGGGTGGGAAAGAAGGACCTGAAACTGTCAGGCCCACACACCCTGATCCCAG
  GGCCAAGGCAGATACAGCCTTCACTGGGAGAAGGCACCTGTGGGTGCCCTGCCCTGACCC
  AGCAATGAAGACATTGCAGAGACAAAGTCAGAAGGAATTGTCCCACTAGTGGGAACAACA
  TAGCATACACTGCCTATGAGGTCCACTCAAGGAGGGCTTCCAGAAGGAGGTAAAGCTAGA
  CCCCGCCCTTCCACATGTGGGGTAGGCATAGGATGTTGAGACTGTAAGAGACATCTCTTT
  GGCCCTCCTTGTATAG
  ============================================================

Primers for ID: 261
Primer pair 1, product size : 635
  Primer left                            Primer right
  start: 25210417                        start: 25211030
  end  : 25210437                        end  : 25211051
  tm   : 57                              tm   : 61
  seq  : GTGACATCTCGGAAGAATTGG           seq  : AGATCTGGACCCATGAGAGAAC

  >261_primer_pair_1_product
  GTGACATCTCGGAAGAATTGGTAGGAGTCCCCAAGGCCTGCAACAGCTACAACAGGAGCG
  CTGGCTGCCAGTGCCCCAGCCACCAGGTGGGGGTACTTCATCCTCATGTAGGCACTCAGC
  ATCCCCCCATAACTGGGAGTACAGAGCACAGATCATGGTTGTGGGAAGCTGCCCACAACT
  CAGGCGAGCAGCCTCACTGTCCTCCAGGCTGAGGTGCTAGGCTGCTCTTTCCCTGCTCAG
  AACGCCCAAGGGTGGGAAAGAAGGACCTGAAACTGTCAGGCCCACACACCCTGATCCCAG
  GGCCAAGGCAGATACAGCCTTCACTGGGAGAAGGCACCTGTGGGTGCCCTGCCCTGACCC
  AGCAATGAAGACATTGCAGAGACAAAGTCAGAAGGAATTGTCCCACTAGTGGGAACAACA
  TAGCATACACTGCCTATGAGGTCCACTCAAGGAGGGCTTCCAGAAGGAGGTAAAGCTAGA
  CCCCGCCCTTCCACATGTGGGGTAGGCATAGGATGTTGAGACTGTAAGAGACATCTCTTT
  GGCCCTCCTTGTATAGGGTGTCAATCGGCACAACAGGGTGGAGCCTTAGAGTAGGGTAAG
  ATTAGGACTCTAGGTTCTCTCATGGGTCCAGATCT
  --------------------

Primer pair 2, product size : 555
  Primer left                            Primer right
  start: 25210417                        start: 25210951
  end  : 25210437                        end  : 25210971
  tm   : 57                              tm   : 57
  seq  : GTGACATCTCGGAAGAATTGG           seq  : TATACAAGGAGGGCCAAAGAG

  >261_primer_pair_2_product
  GTGACATCTCGGAAGAATTGGTAGGAGTCCCCAAGGCCTGCAACAGCTACAACAGGAGCG
  CTGGCTGCCAGTGCCCCAGCCACCAGGTGGGGGTACTTCATCCTCATGTAGGCACTCAGC
  ATCCCCCCATAACTGGGAGTACAGAGCACAGATCATGGTTGTGGGAAGCTGCCCACAACT
  CAGGCGAGCAGCCTCACTGTCCTCCAGGCTGAGGTGCTAGGCTGCTCTTTCCCTGCTCAG
  AACGCCCAAGGGTGGGAAAGAAGGACCTGAAACTGTCAGGCCCACACACCCTGATCCCAG
  GGCCAAGGCAGATACAGCCTTCACTGGGAGAAGGCACCTGTGGGTGCCCTGCCCTGACCC
  AGCAATGAAGACATTGCAGAGACAAAGTCAGAAGGAATTGTCCCACTAGTGGGAACAACA
  TAGCATACACTGCCTATGAGGTCCACTCAAGGAGGGCTTCCAGAAGGAGGTAAAGCTAGA
  CCCCGCCCTTCCACATGTGGGGTAGGCATAGGATGTTGAGACTGTAAGAGACATCTCTTT
  GGCCCTCCTTGTATA
  ============================================================

Primers for ID: 262
Primer pair 1, product size : 540
  Primer left                            Primer right
  start: 25210512                        start: 25211030
  end  : 25210533                        end  : 25211051
  tm   : 61                              tm   : 61
  seq  : CTTCATCCTCATGTAGGCACTC          seq  : AGATCTGGACCCATGAGAGAAC

  >262_primer_pair_1_product
  CTTCATCCTCATGTAGGCACTCAGCATCCCCCCATAACTGGGAGTACAGAGCACAGATCA
  TGGTTGTGGGAAGCTGCCCACAACTCAGGCGAGCAGCCTCACTGTCCTCCAGGCTGAGGT
  GCTAGGCTGCTCTTTCCCTGCTCAGAACGCCCAAGGGTGGGAAAGAAGGACCTGAAACTG
  TCAGGCCCACACACCCTGATCCCAGGGCCAAGGCAGATACAGCCTTCACTGGGAGAAGGC
  ACCTGTGGGTGCCCTGCCCTGACCCAGCAATGAAGACATTGCAGAGACAAAGTCAGAAGG
  AATTGTCCCACTAGTGGGAACAACATAGCATACACTGCCTATGAGGTCCACTCAAGGAGG
  GCTTCCAGAAGGAGGTAAAGCTAGACCCCGCCCTTCCACATGTGGGGTAGGCATAGGATG
  TTGAGACTGTAAGAGACATCTCTTTGGCCCTCCTTGTATAGGGTGTCAATCGGCACAACA
  GGGTGGAGCCTTAGAGTAGGGTAAGATTAGGACTCTAGGTTCTCTCATGGGTCCAGATCT
  --------------------

Primer pair 2, product size : 539
  Primer left                            Primer right
  start: 25210513                        start: 25211030
  end  : 25210533                        end  : 25211051
  tm   : 57                              tm   : 61
  seq  : TTCATCCTCATGTAGGCACTC           seq  : AGATCTGGACCCATGAGAGAAC

  >262_primer_pair_2_product
  TTCATCCTCATGTAGGCACTCAGCATCCCCCCATAACTGGGAGTACAGAGCACAGATCAT
  GGTTGTGGGAAGCTGCCCACAACTCAGGCGAGCAGCCTCACTGTCCTCCAGGCTGAGGTG
  CTAGGCTGCTCTTTCCCTGCTCAGAACGCCCAAGGGTGGGAAAGAAGGACCTGAAACTGT
  CAGGCCCACACACCCTGATCCCAGGGCCAAGGCAGATACAGCCTTCACTGGGAGAAGGCA
  CCTGTGGGTGCCCTGCCCTGACCCAGCAATGAAGACATTGCAGAGACAAAGTCAGAAGGA
  ATTGTCCCACTAGTGGGAACAACATAGCATACACTGCCTATGAGGTCCACTCAAGGAGGG
  CTTCCAGAAGGAGGTAAAGCTAGACCCCGCCCTTCCACATGTGGGGTAGGCATAGGATGT
  TGAGACTGTAAGAGACATCTCTTTGGCCCTCCTTGTATAGGGTGTCAATCGGCACAACAG
  GGTGGAGCCTTAGAGTAGGGTAAGATTAGGACTCTAGGTTCTCTCATGGGTCCAGATCT
  ============================================================

Primers for ID: 272
Primer pair 1, product size : 541
  Primer left                            Primer right
  start: 25211173                        start: 25211693
  end  : 25211194                        end  : 25211713
  tm   : 61                              tm   : 57
  seq  : AGTCAGCAGCTGTGTATATCCC          seq  : TAGATCCCTGATGATCCCAAC

  >272_primer_pair_1_product
  AGTCAGCAGCTGTGTATATCCCCGCTGTGTGGACTGGACACCGAACGGAAGCGATTTCCC
  ATAGTACCGCTGCAGAAAGCAGGAAGGGATGGCTAATCCACTCCTCGGTGCTCCCCACCT
  CCTTCAACTCAGGGACTGCCAGGAACTGTACAGGTACCCACGTGCTCAGCAAAGACAAGC
  AGGGCCTCCTGCTGGGCTGCCAGTTCCACCATGAAGCCAGAGTTGTTAGCGAAGGACCAG
  ATATCCCCCTCATTCCCTGTGTAGAAAAAGATGGGCCCTTCGCCCATCTTCCAGAACTTA
  TCTGTTGGAAGTAAATGAGTTTCCATAAGGCCAGGGAAACGCAGGTAGGAACCCATGCGG
  TCGAGCCAGCACTCACCTGACACTAGGAACCGCTGGCCAAAGGTTTTGTTGCCGAAACTC
  TCAAAGTTGAAATGGTCCATGTATTGCTCAAAATAATTCTCATGAAAGTCAGGGTCTAGA
  ACTCTGTCGGCTGAGGGCAGGTGCAGAGACTCAGGAGCTGGTTGGGATCATCAGGGATCT
  A
  --------------------

Primer pair 2, product size : 542
  Primer left                            Primer right
  start: 25211173                        start: 25211693
  end  : 25211194                        end  : 25211714
  tm   : 61                              tm   : 61
  seq  : AGTCAGCAGCTGTGTATATCCC          seq  : CTAGATCCCTGATGATCCCAAC

  >272_primer_pair_2_product
  AGTCAGCAGCTGTGTATATCCCCGCTGTGTGGACTGGACACCGAACGGAAGCGATTTCCC
  ATAGTACCGCTGCAGAAAGCAGGAAGGGATGGCTAATCCACTCCTCGGTGCTCCCCACCT
  CCTTCAACTCAGGGACTGCCAGGAACTGTACAGGTACCCACGTGCTCAGCAAAGACAAGC
  AGGGCCTCCTGCTGGGCTGCCAGTTCCACCATGAAGCCAGAGTTGTTAGCGAAGGACCAG
  ATATCCCCCTCATTCCCTGTGTAGAAAAAGATGGGCCCTTCGCCCATCTTCCAGAACTTA
  TCTGTTGGAAGTAAATGAGTTTCCATAAGGCCAGGGAAACGCAGGTAGGAACCCATGCGG
  TCGAGCCAGCACTCACCTGACACTAGGAACCGCTGGCCAAAGGTTTTGTTGCCGAAACTC
  TCAAAGTTGAAATGGTCCATGTATTGCTCAAAATAATTCTCATGAAAGTCAGGGTCTAGA
  ACTCTGTCGGCTGAGGGCAGGTGCAGAGACTCAGGAGCTGGTTGGGATCATCAGGGATCT
  AG
  ============================================================

Primers for ID: 273
Primer pair 1, product size : 505
  Primer left                            Primer right
  start: 25211383                        start: 25211867
  end  : 25211403                        end  : 25211887
  tm   : 57                              tm   : 57
  seq  : ATGAAGCCAGAGTTGTTAGCG           seq  : CCAAATCACATGACAGTCAGC

  >273_primer_pair_1_product
  ATGAAGCCAGAGTTGTTAGCGAAGGACCAGATATCCCCCTCATTCCCTGTGTAGAAAAAG
  ATGGGCCCTTCGCCCATCTTCCAGAACTTATCTGTTGGAAGTAAATGAGTTTCCATAAGG
  CCAGGGAAACGCAGGTAGGAACCCATGCGGTCGAGCCAGCACTCACCTGACACTAGGAAC
  CGCTGGCCAAAGGTTTTGTTGCCGAAACTCTCAAAGTTGAAATGGTCCATGTATTGCTCA
  AAATAATTCTCATGAAAGTCAGGGTCTAGAACTCTGTCGGCTGAGGGCAGGTGCAGAGAC
  TCAGGAGCTGGTTGGGATCATCAGGGATCTAGGCGGGTCAGGAGGAAGGGCAGCCAGTCT
  GTACTCACCTCTGGCCTGGAGGTTGCACAGTCCCAGTGACAGCAGCAGGACCAGGATCCA
  GGAGGGGACACCATGGTCCACAGGGTAACAAGGATGGAAGTTCATGCTTGATTCTGAGCC
  GGGCGCTGACTGTCATGTGATTTGG
  --------------------

Primer pair 2, product size : 531
  Primer left                            Primer right
  start: 25211332                        start: 25211842
  end  : 25211351                        end  : 25211862
  tm   : 55                              tm   : 57
  seq  : ACGTGCTCAGCAAAGACAAG            seq  : GGCTCAGAATCAAGCATGAAC

  >273_primer_pair_2_product
  ACGTGCTCAGCAAAGACAAGCAGGGCCTCCTGCTGGGCTGCCAGTTCCACCATGAAGCCA
  GAGTTGTTAGCGAAGGACCAGATATCCCCCTCATTCCCTGTGTAGAAAAAGATGGGCCCT
  TCGCCCATCTTCCAGAACTTATCTGTTGGAAGTAAATGAGTTTCCATAAGGCCAGGGAAA
  CGCAGGTAGGAACCCATGCGGTCGAGCCAGCACTCACCTGACACTAGGAACCGCTGGCCA
  AAGGTTTTGTTGCCGAAACTCTCAAAGTTGAAATGGTCCATGTATTGCTCAAAATAATTC
  TCATGAAAGTCAGGGTCTAGAACTCTGTCGGCTGAGGGCAGGTGCAGAGACTCAGGAGCT
  GGTTGGGATCATCAGGGATCTAGGCGGGTCAGGAGGAAGGGCAGCCAGTCTGTACTCACC
  TCTGGCCTGGAGGTTGCACAGTCCCAGTGACAGCAGCAGGACCAGGATCCAGGAGGGGAC
  ACCATGGTCCACAGGGTAACAAGGATGGAAGTTCATGCTTGATTCTGAGCC
  ============================================================

Primers for ID: 274
Primer pair 1, product size : 505
  Primer left                            Primer right
  start: 25211383                        start: 25211867
  end  : 25211403                        end  : 25211887
  tm   : 57                              tm   : 57
  seq  : ATGAAGCCAGAGTTGTTAGCG           seq  : CCAAATCACATGACAGTCAGC

  >274_primer_pair_1_product
  ATGAAGCCAGAGTTGTTAGCGAAGGACCAGATATCCCCCTCATTCCCTGTGTAGAAAAAG
  ATGGGCCCTTCGCCCATCTTCCAGAACTTATCTGTTGGAAGTAAATGAGTTTCCATAAGG
  CCAGGGAAACGCAGGTAGGAACCCATGCGGTCGAGCCAGCACTCACCTGACACTAGGAAC
  CGCTGGCCAAAGGTTTTGTTGCCGAAACTCTCAAAGTTGAAATGGTCCATGTATTGCTCA
  AAATAATTCTCATGAAAGTCAGGGTCTAGAACTCTGTCGGCTGAGGGCAGGTGCAGAGAC
  TCAGGAGCTGGTTGGGATCATCAGGGATCTAGGCGGGTCAGGAGGAAGGGCAGCCAGTCT
  GTACTCACCTCTGGCCTGGAGGTTGCACAGTCCCAGTGACAGCAGCAGGACCAGGATCCA
  GGAGGGGACACCATGGTCCACAGGGTAACAAGGATGGAAGTTCATGCTTGATTCTGAGCC
  GGGCGCTGACTGTCATGTGATTTGG
  --------------------

Primer pair 2, product size : 531
  Primer left                            Primer right
  start: 25211332                        start: 25211842
  end  : 25211351                        end  : 25211862
  tm   : 55                              tm   : 57
  seq  : ACGTGCTCAGCAAAGACAAG            seq  : GGCTCAGAATCAAGCATGAAC

  >274_primer_pair_2_product
  ACGTGCTCAGCAAAGACAAGCAGGGCCTCCTGCTGGGCTGCCAGTTCCACCATGAAGCCA
  GAGTTGTTAGCGAAGGACCAGATATCCCCCTCATTCCCTGTGTAGAAAAAGATGGGCCCT
  TCGCCCATCTTCCAGAACTTATCTGTTGGAAGTAAATGAGTTTCCATAAGGCCAGGGAAA
  CGCAGGTAGGAACCCATGCGGTCGAGCCAGCACTCACCTGACACTAGGAACCGCTGGCCA
  AAGGTTTTGTTGCCGAAACTCTCAAAGTTGAAATGGTCCATGTATTGCTCAAAATAATTC
  TCATGAAAGTCAGGGTCTAGAACTCTGTCGGCTGAGGGCAGGTGCAGAGACTCAGGAGCT
  GGTTGGGATCATCAGGGATCTAGGCGGGTCAGGAGGAAGGGCAGCCAGTCTGTACTCACC
  TCTGGCCTGGAGGTTGCACAGTCCCAGTGACAGCAGCAGGACCAGGATCCAGGAGGGGAC
  ACCATGGTCCACAGGGTAACAAGGATGGAAGTTCATGCTTGATTCTGAGCC
  ============================================================

Primers for ID: 275
Primer pair 1, product size : 620
  Primer left                            Primer right
  start: 25211383                        start: 25211982
  end  : 25211403                        end  : 25212002
  tm   : 57                              tm   : 57
  seq  : ATGAAGCCAGAGTTGTTAGCG           seq  : AACCGGGAAACTGAAGAAGAG

  >275_primer_pair_1_product
  ATGAAGCCAGAGTTGTTAGCGAAGGACCAGATATCCCCCTCATTCCCTGTGTAGAAAAAG
  ATGGGCCCTTCGCCCATCTTCCAGAACTTATCTGTTGGAAGTAAATGAGTTTCCATAAGG
  CCAGGGAAACGCAGGTAGGAACCCATGCGGTCGAGCCAGCACTCACCTGACACTAGGAAC
  CGCTGGCCAAAGGTTTTGTTGCCGAAACTCTCAAAGTTGAAATGGTCCATGTATTGCTCA
  AAATAATTCTCATGAAAGTCAGGGTCTAGAACTCTGTCGGCTGAGGGCAGGTGCAGAGAC
  TCAGGAGCTGGTTGGGATCATCAGGGATCTAGGCGGGTCAGGAGGAAGGGCAGCCAGTCT
  GTACTCACCTCTGGCCTGGAGGTTGCACAGTCCCAGTGACAGCAGCAGGACCAGGATCCA
  GGAGGGGACACCATGGTCCACAGGGTAACAAGGATGGAAGTTCATGCTTGATTCTGAGCC
  GGGCGCTGACTGTCATGTGATTTGGTCACATGACCGACACAACGGGCGGGGCAGCATCAC
  GTGATAGTCTGGCGGGGGCTGTCCTACTGTGGCTGGATTCTAGTTGGAGGATCAGCCTAC
  TCTTCTTCAGTTTCCCGGTT
  --------------------

Primer pair 2, product size : 505
  Primer left                            Primer right
  start: 25211383                        start: 25211867
  end  : 25211403                        end  : 25211887
  tm   : 57                              tm   : 57
  seq  : ATGAAGCCAGAGTTGTTAGCG           seq  : CCAAATCACATGACAGTCAGC

  >275_primer_pair_2_product
  ATGAAGCCAGAGTTGTTAGCGAAGGACCAGATATCCCCCTCATTCCCTGTGTAGAAAAAG
  ATGGGCCCTTCGCCCATCTTCCAGAACTTATCTGTTGGAAGTAAATGAGTTTCCATAAGG
  CCAGGGAAACGCAGGTAGGAACCCATGCGGTCGAGCCAGCACTCACCTGACACTAGGAAC
  CGCTGGCCAAAGGTTTTGTTGCCGAAACTCTCAAAGTTGAAATGGTCCATGTATTGCTCA
  AAATAATTCTCATGAAAGTCAGGGTCTAGAACTCTGTCGGCTGAGGGCAGGTGCAGAGAC
  TCAGGAGCTGGTTGGGATCATCAGGGATCTAGGCGGGTCAGGAGGAAGGGCAGCCAGTCT
  GTACTCACCTCTGGCCTGGAGGTTGCACAGTCCCAGTGACAGCAGCAGGACCAGGATCCA
  GGAGGGGACACCATGGTCCACAGGGTAACAAGGATGGAAGTTCATGCTTGATTCTGAGCC
  GGGCGCTGACTGTCATGTGATTTGG
  ============================================================

Primers for ID: 276
Primer pair 1, product size : 575
  Primer left                            Primer right
  start: 25211428                        start: 25211982
  end  : 25211448                        end  : 25212002
  tm   : 57                              tm   : 57
  seq  : CCTGTGTAGAAAAAGATGGGC           seq  : AACCGGGAAACTGAAGAAGAG

  >276_primer_pair_1_product
  CCTGTGTAGAAAAAGATGGGCCCTTCGCCCATCTTCCAGAACTTATCTGTTGGAAGTAAA
  TGAGTTTCCATAAGGCCAGGGAAACGCAGGTAGGAACCCATGCGGTCGAGCCAGCACTCA
  CCTGACACTAGGAACCGCTGGCCAAAGGTTTTGTTGCCGAAACTCTCAAAGTTGAAATGG
  TCCATGTATTGCTCAAAATAATTCTCATGAAAGTCAGGGTCTAGAACTCTGTCGGCTGAG
  GGCAGGTGCAGAGACTCAGGAGCTGGTTGGGATCATCAGGGATCTAGGCGGGTCAGGAGG
  AAGGGCAGCCAGTCTGTACTCACCTCTGGCCTGGAGGTTGCACAGTCCCAGTGACAGCAG
  CAGGACCAGGATCCAGGAGGGGACACCATGGTCCACAGGGTAACAAGGATGGAAGTTCAT
  GCTTGATTCTGAGCCGGGCGCTGACTGTCATGTGATTTGGTCACATGACCGACACAACGG
  GCGGGGCAGCATCACGTGATAGTCTGGCGGGGGCTGTCCTACTGTGGCTGGATTCTAGTT
  GGAGGATCAGCCTACTCTTCTTCAGTTTCCCGGTT
  --------------------

Primer pair 2, product size : 576
  Primer left                            Primer right
  start: 25211427                        start: 25211982
  end  : 25211447                        end  : 25212002
  tm   : 57                              tm   : 57
  seq  : CCCTGTGTAGAAAAAGATGGG           seq  : AACCGGGAAACTGAAGAAGAG

  >276_primer_pair_2_product
  CCCTGTGTAGAAAAAGATGGGCCCTTCGCCCATCTTCCAGAACTTATCTGTTGGAAGTAA
  ATGAGTTTCCATAAGGCCAGGGAAACGCAGGTAGGAACCCATGCGGTCGAGCCAGCACTC
  ACCTGACACTAGGAACCGCTGGCCAAAGGTTTTGTTGCCGAAACTCTCAAAGTTGAAATG
  GTCCATGTATTGCTCAAAATAATTCTCATGAAAGTCAGGGTCTAGAACTCTGTCGGCTGA
  GGGCAGGTGCAGAGACTCAGGAGCTGGTTGGGATCATCAGGGATCTAGGCGGGTCAGGAG
  GAAGGGCAGCCAGTCTGTACTCACCTCTGGCCTGGAGGTTGCACAGTCCCAGTGACAGCA
  GCAGGACCAGGATCCAGGAGGGGACACCATGGTCCACAGGGTAACAAGGATGGAAGTTCA
  TGCTTGATTCTGAGCCGGGCGCTGACTGTCATGTGATTTGGTCACATGACCGACACAACG
  GGCGGGGCAGCATCACGTGATAGTCTGGCGGGGGCTGTCCTACTGTGGCTGGATTCTAGT
  TGGAGGATCAGCCTACTCTTCTTCAGTTTCCCGGTT
  ============================================================

Primers for ID: 277
Primer pair 1, product size : 518
  Primer left                            Primer right
  start: 25211485                        start: 25211982
  end  : 25211506                        end  : 25212002
  tm   : 57                              tm   : 57
  seq  : AAATGAGTTTCCATAAGGCCAG          seq  : AACCGGGAAACTGAAGAAGAG

  >277_primer_pair_1_product
  AAATGAGTTTCCATAAGGCCAGGGAAACGCAGGTAGGAACCCATGCGGTCGAGCCAGCAC
  TCACCTGACACTAGGAACCGCTGGCCAAAGGTTTTGTTGCCGAAACTCTCAAAGTTGAAA
  TGGTCCATGTATTGCTCAAAATAATTCTCATGAAAGTCAGGGTCTAGAACTCTGTCGGCT
  GAGGGCAGGTGCAGAGACTCAGGAGCTGGTTGGGATCATCAGGGATCTAGGCGGGTCAGG
  AGGAAGGGCAGCCAGTCTGTACTCACCTCTGGCCTGGAGGTTGCACAGTCCCAGTGACAG
  CAGCAGGACCAGGATCCAGGAGGGGACACCATGGTCCACAGGGTAACAAGGATGGAAGTT
  CATGCTTGATTCTGAGCCGGGCGCTGACTGTCATGTGATTTGGTCACATGACCGACACAA
  CGGGCGGGGCAGCATCACGTGATAGTCTGGCGGGGGCTGTCCTACTGTGGCTGGATTCTA
  GTTGGAGGATCAGCCTACTCTTCTTCAGTTTCCCGGTT
  --------------------

Primer pair 2, product size : 517
  Primer left                            Primer right
  start: 25211486                        start: 25211982
  end  : 25211506                        end  : 25212002
  tm   : 55                              tm   : 57
  seq  : AATGAGTTTCCATAAGGCCAG           seq  : AACCGGGAAACTGAAGAAGAG

  >277_primer_pair_2_product
  AATGAGTTTCCATAAGGCCAGGGAAACGCAGGTAGGAACCCATGCGGTCGAGCCAGCACT
  CACCTGACACTAGGAACCGCTGGCCAAAGGTTTTGTTGCCGAAACTCTCAAAGTTGAAAT
  GGTCCATGTATTGCTCAAAATAATTCTCATGAAAGTCAGGGTCTAGAACTCTGTCGGCTG
  AGGGCAGGTGCAGAGACTCAGGAGCTGGTTGGGATCATCAGGGATCTAGGCGGGTCAGGA
  GGAAGGGCAGCCAGTCTGTACTCACCTCTGGCCTGGAGGTTGCACAGTCCCAGTGACAGC
  AGCAGGACCAGGATCCAGGAGGGGACACCATGGTCCACAGGGTAACAAGGATGGAAGTTC
  ATGCTTGATTCTGAGCCGGGCGCTGACTGTCATGTGATTTGGTCACATGACCGACACAAC
  GGGCGGGGCAGCATCACGTGATAGTCTGGCGGGGGCTGTCCTACTGTGGCTGGATTCTAG
  TTGGAGGATCAGCCTACTCTTCTTCAGTTTCCCGGTT
  ============================================================

Primers for ID: 278
Primer pair 1, product size : 525
  Primer left                            Primer right
  start: 25211573                        start: 25212076
  end  : 25211592                        end  : 25212097
  tm   : 53                              tm   : 61
  seq  : AGGTTTTGTTGCCGAAACTC            seq  : ATACGGTCCTGATGAGCCTTAG

  >278_primer_pair_1_product
  AGGTTTTGTTGCCGAAACTCTCAAAGTTGAAATGGTCCATGTATTGCTCAAAATAATTCT
  CATGAAAGTCAGGGTCTAGAACTCTGTCGGCTGAGGGCAGGTGCAGAGACTCAGGAGCTG
  GTTGGGATCATCAGGGATCTAGGCGGGTCAGGAGGAAGGGCAGCCAGTCTGTACTCACCT
  CTGGCCTGGAGGTTGCACAGTCCCAGTGACAGCAGCAGGACCAGGATCCAGGAGGGGACA
  CCATGGTCCACAGGGTAACAAGGATGGAAGTTCATGCTTGATTCTGAGCCGGGCGCTGAC
  TGTCATGTGATTTGGTCACATGACCGACACAACGGGCGGGGCAGCATCACGTGATAGTCT
  GGCGGGGGCTGTCCTACTGTGGCTGGATTCTAGTTGGAGGATCAGCCTACTCTTCTTCAG
  TTTCCCGGTTCCTCCAAATTTCTGGGCTCCTACTTGTTTCCACAGAGATGGATACTGTGG
  AGGTCCAGGAAGCAGAGAGATGGCTAAGGCTCATCAGGACCGTAT
  --------------------

Primer pair 2, product size : 500
  Primer left                            Primer right
  start: 25211598                        start: 25212076
  end  : 25211619                        end  : 25212097
  tm   : 57                              tm   : 61
  seq  : GTTGAAATGGTCCATGTATTGC          seq  : ATACGGTCCTGATGAGCCTTAG

  >278_primer_pair_2_product
  GTTGAAATGGTCCATGTATTGCTCAAAATAATTCTCATGAAAGTCAGGGTCTAGAACTCT
  GTCGGCTGAGGGCAGGTGCAGAGACTCAGGAGCTGGTTGGGATCATCAGGGATCTAGGCG
  GGTCAGGAGGAAGGGCAGCCAGTCTGTACTCACCTCTGGCCTGGAGGTTGCACAGTCCCA
  GTGACAGCAGCAGGACCAGGATCCAGGAGGGGACACCATGGTCCACAGGGTAACAAGGAT
  GGAAGTTCATGCTTGATTCTGAGCCGGGCGCTGACTGTCATGTGATTTGGTCACATGACC
  GACACAACGGGCGGGGCAGCATCACGTGATAGTCTGGCGGGGGCTGTCCTACTGTGGCTG
  GATTCTAGTTGGAGGATCAGCCTACTCTTCTTCAGTTTCCCGGTTCCTCCAAATTTCTGG
  GCTCCTACTTGTTTCCACAGAGATGGATACTGTGGAGGTCCAGGAAGCAGAGAGATGGCT
  AAGGCTCATCAGGACCGTAT
  ============================================================

Primers for ID: 279
Primer pair 1, product size : 500
  Primer left                            Primer right
  start: 25211822                        start: 25212301
  end  : 25211842                        end  : 25212321
  tm   : 57                              tm   : 57
  seq  : ACAGGGTAACAAGGATGGAAG           seq  : TAATTATGGGCCTGTCTGGTG

  >279_primer_pair_1_product
  ACAGGGTAACAAGGATGGAAGTTCATGCTTGATTCTGAGCCGGGCGCTGACTGTCATGTG
  ATTTGGTCACATGACCGACACAACGGGCGGGGCAGCATCACGTGATAGTCTGGCGGGGGC
  TGTCCTACTGTGGCTGGATTCTAGTTGGAGGATCAGCCTACTCTTCTTCAGTTTCCCGGT
  TCCTCCAAATTTCTGGGCTCCTACTTGTTTCCACAGAGATGGATACTGTGGAGGTCCAGG
  AAGCAGAGAGATGGCTAAGGCTCATCAGGACCGTATGATCTCCCAAGTGTCCAGCTACTG
  AGTACCACAAGGTGATGGGTGGGAGGGTCCTCCCACGGAAGGATACCGCAGTCCCTAGGG
  GTTGCAAGCCCCACATGTTCCACTGGCTGCTAGAGCTACCTACTCAATCAGCCCTGGGCA
  TCACCATCAGGTACTCGGCCAAAATGACCTCTCTGCTTCCAGTCCTCAGTTCTGGTCAGC
  ACCAGACAGGCCCATAATTA
  --------------------

Primer pair 2, product size : 503
  Primer left                            Primer right
  start: 25211819                        start: 25212301
  end  : 25211838                        end  : 25212321
  tm   : 55                              tm   : 57
  seq  : TCCACAGGGTAACAAGGATG            seq  : TAATTATGGGCCTGTCTGGTG

  >279_primer_pair_2_product
  TCCACAGGGTAACAAGGATGGAAGTTCATGCTTGATTCTGAGCCGGGCGCTGACTGTCAT
  GTGATTTGGTCACATGACCGACACAACGGGCGGGGCAGCATCACGTGATAGTCTGGCGGG
  GGCTGTCCTACTGTGGCTGGATTCTAGTTGGAGGATCAGCCTACTCTTCTTCAGTTTCCC
  GGTTCCTCCAAATTTCTGGGCTCCTACTTGTTTCCACAGAGATGGATACTGTGGAGGTCC
  AGGAAGCAGAGAGATGGCTAAGGCTCATCAGGACCGTATGATCTCCCAAGTGTCCAGCTA
  CTGAGTACCACAAGGTGATGGGTGGGAGGGTCCTCCCACGGAAGGATACCGCAGTCCCTA
  GGGGTTGCAAGCCCCACATGTTCCACTGGCTGCTAGAGCTACCTACTCAATCAGCCCTGG
  GCATCACCATCAGGTACTCGGCCAAAATGACCTCTCTGCTTCCAGTCCTCAGTTCTGGTC
  AGCACCAGACAGGCCCATAATTA
  ============================================================

Primers for ID: 280
Primer pair 1, product size : 507
  Primer left                            Primer right
  start: 25211842                        start: 25212328
  end  : 25211862                        end  : 25212348
  tm   : 57                              tm   : 55
  seq  : GTTCATGCTTGATTCTGAGCC           seq  : ACAAATGTTCCAGTTTCCCTG

  >280_primer_pair_1_product
  GTTCATGCTTGATTCTGAGCCGGGCGCTGACTGTCATGTGATTTGGTCACATGACCGACA
  CAACGGGCGGGGCAGCATCACGTGATAGTCTGGCGGGGGCTGTCCTACTGTGGCTGGATT
  CTAGTTGGAGGATCAGCCTACTCTTCTTCAGTTTCCCGGTTCCTCCAAATTTCTGGGCTC
  CTACTTGTTTCCACAGAGATGGATACTGTGGAGGTCCAGGAAGCAGAGAGATGGCTAAGG
  CTCATCAGGACCGTATGATCTCCCAAGTGTCCAGCTACTGAGTACCACAAGGTGATGGGT
  GGGAGGGTCCTCCCACGGAAGGATACCGCAGTCCCTAGGGGTTGCAAGCCCCACATGTTC
  CACTGGCTGCTAGAGCTACCTACTCAATCAGCCCTGGGCATCACCATCAGGTACTCGGCC
  AAAATGACCTCTCTGCTTCCAGTCCTCAGTTCTGGTCAGCACCAGACAGGCCCATAATTA
  CAGAGCCAGGGAAACTGGAACATTTGT
  --------------------

Primer pair 2, product size : 511
  Primer left                            Primer right
  start: 25211842                        start: 25212331
  end  : 25211862                        end  : 25212352
  tm   : 57                              tm   : 59
  seq  : GTTCATGCTTGATTCTGAGCC           seq  : GGAGACAAATGTTCCAGTTTCC

  >280_primer_pair_2_product
  GTTCATGCTTGATTCTGAGCCGGGCGCTGACTGTCATGTGATTTGGTCACATGACCGACA
  CAACGGGCGGGGCAGCATCACGTGATAGTCTGGCGGGGGCTGTCCTACTGTGGCTGGATT
  CTAGTTGGAGGATCAGCCTACTCTTCTTCAGTTTCCCGGTTCCTCCAAATTTCTGGGCTC
  CTACTTGTTTCCACAGAGATGGATACTGTGGAGGTCCAGGAAGCAGAGAGATGGCTAAGG
  CTCATCAGGACCGTATGATCTCCCAAGTGTCCAGCTACTGAGTACCACAAGGTGATGGGT
  GGGAGGGTCCTCCCACGGAAGGATACCGCAGTCCCTAGGGGTTGCAAGCCCCACATGTTC
  CACTGGCTGCTAGAGCTACCTACTCAATCAGCCCTGGGCATCACCATCAGGTACTCGGCC
  AAAATGACCTCTCTGCTTCCAGTCCTCAGTTCTGGTCAGCACCAGACAGGCCCATAATTA
  CAGAGCCAGGGAAACTGGAACATTTGTCTCC
  ============================================================

Primers for ID: 281
Primer pair 1, product size : 596
  Primer left                            Primer right
  start: 25211842                        start: 25212417
  end  : 25211862                        end  : 25212437
  tm   : 57                              tm   : 57
  seq  : GTTCATGCTTGATTCTGAGCC           seq  : AGATTCCCAGAAAGTCCAAGG

  >281_primer_pair_1_product
  GTTCATGCTTGATTCTGAGCCGGGCGCTGACTGTCATGTGATTTGGTCACATGACCGACA
  CAACGGGCGGGGCAGCATCACGTGATAGTCTGGCGGGGGCTGTCCTACTGTGGCTGGATT
  CTAGTTGGAGGATCAGCCTACTCTTCTTCAGTTTCCCGGTTCCTCCAAATTTCTGGGCTC
  CTACTTGTTTCCACAGAGATGGATACTGTGGAGGTCCAGGAAGCAGAGAGATGGCTAAGG
  CTCATCAGGACCGTATGATCTCCCAAGTGTCCAGCTACTGAGTACCACAAGGTGATGGGT
  GGGAGGGTCCTCCCACGGAAGGATACCGCAGTCCCTAGGGGTTGCAAGCCCCACATGTTC
  CACTGGCTGCTAGAGCTACCTACTCAATCAGCCCTGGGCATCACCATCAGGTACTCGGCC
  AAAATGACCTCTCTGCTTCCAGTCCTCAGTTCTGGTCAGCACCAGACAGGCCCATAATTA
  CAGAGCCAGGGAAACTGGAACATTTGTCTCCCCTTAGACAGTGGCAGCAGGAAGGTGGGG
  GGTTGTTGCAGAGGAACAGTGTCTCTGAGAGAGGACCTTGGACTTTCTGGGAATCT
  --------------------

Primer pair 2, product size : 594
  Primer left                            Primer right
  start: 25211842                        start: 25212415
  end  : 25211862                        end  : 25212435
  tm   : 57                              tm   : 57
  seq  : GTTCATGCTTGATTCTGAGCC           seq  : ATTCCCAGAAAGTCCAAGGTC

  >281_primer_pair_2_product
  GTTCATGCTTGATTCTGAGCCGGGCGCTGACTGTCATGTGATTTGGTCACATGACCGACA
  CAACGGGCGGGGCAGCATCACGTGATAGTCTGGCGGGGGCTGTCCTACTGTGGCTGGATT
  CTAGTTGGAGGATCAGCCTACTCTTCTTCAGTTTCCCGGTTCCTCCAAATTTCTGGGCTC
  CTACTTGTTTCCACAGAGATGGATACTGTGGAGGTCCAGGAAGCAGAGAGATGGCTAAGG
  CTCATCAGGACCGTATGATCTCCCAAGTGTCCAGCTACTGAGTACCACAAGGTGATGGGT
  GGGAGGGTCCTCCCACGGAAGGATACCGCAGTCCCTAGGGGTTGCAAGCCCCACATGTTC
  CACTGGCTGCTAGAGCTACCTACTCAATCAGCCCTGGGCATCACCATCAGGTACTCGGCC
  AAAATGACCTCTCTGCTTCCAGTCCTCAGTTCTGGTCAGCACCAGACAGGCCCATAATTA
  CAGAGCCAGGGAAACTGGAACATTTGTCTCCCCTTAGACAGTGGCAGCAGGAAGGTGGGG
  GGTTGTTGCAGAGGAACAGTGTCTCTGAGAGAGGACCTTGGACTTTCTGGGAAT
  ============================================================

Primers for ID: 282
Primer pair 1, product size : 596
  Primer left                            Primer right
  start: 25211842                        start: 25212417
  end  : 25211862                        end  : 25212437
  tm   : 57                              tm   : 57
  seq  : GTTCATGCTTGATTCTGAGCC           seq  : AGATTCCCAGAAAGTCCAAGG

  >282_primer_pair_1_product
  GTTCATGCTTGATTCTGAGCCGGGCGCTGACTGTCATGTGATTTGGTCACATGACCGACA
  CAACGGGCGGGGCAGCATCACGTGATAGTCTGGCGGGGGCTGTCCTACTGTGGCTGGATT
  CTAGTTGGAGGATCAGCCTACTCTTCTTCAGTTTCCCGGTTCCTCCAAATTTCTGGGCTC
  CTACTTGTTTCCACAGAGATGGATACTGTGGAGGTCCAGGAAGCAGAGAGATGGCTAAGG
  CTCATCAGGACCGTATGATCTCCCAAGTGTCCAGCTACTGAGTACCACAAGGTGATGGGT
  GGGAGGGTCCTCCCACGGAAGGATACCGCAGTCCCTAGGGGTTGCAAGCCCCACATGTTC
  CACTGGCTGCTAGAGCTACCTACTCAATCAGCCCTGGGCATCACCATCAGGTACTCGGCC
  AAAATGACCTCTCTGCTTCCAGTCCTCAGTTCTGGTCAGCACCAGACAGGCCCATAATTA
  CAGAGCCAGGGAAACTGGAACATTTGTCTCCCCTTAGACAGTGGCAGCAGGAAGGTGGGG
  GGTTGTTGCAGAGGAACAGTGTCTCTGAGAGAGGACCTTGGACTTTCTGGGAATCT
  --------------------

Primer pair 2, product size : 594
  Primer left                            Primer right
  start: 25211842                        start: 25212415
  end  : 25211862                        end  : 25212435
  tm   : 57                              tm   : 57
  seq  : GTTCATGCTTGATTCTGAGCC           seq  : ATTCCCAGAAAGTCCAAGGTC

  >282_primer_pair_2_product
  GTTCATGCTTGATTCTGAGCCGGGCGCTGACTGTCATGTGATTTGGTCACATGACCGACA
  CAACGGGCGGGGCAGCATCACGTGATAGTCTGGCGGGGGCTGTCCTACTGTGGCTGGATT
  CTAGTTGGAGGATCAGCCTACTCTTCTTCAGTTTCCCGGTTCCTCCAAATTTCTGGGCTC
  CTACTTGTTTCCACAGAGATGGATACTGTGGAGGTCCAGGAAGCAGAGAGATGGCTAAGG
  CTCATCAGGACCGTATGATCTCCCAAGTGTCCAGCTACTGAGTACCACAAGGTGATGGGT
  GGGAGGGTCCTCCCACGGAAGGATACCGCAGTCCCTAGGGGTTGCAAGCCCCACATGTTC
  CACTGGCTGCTAGAGCTACCTACTCAATCAGCCCTGGGCATCACCATCAGGTACTCGGCC
  AAAATGACCTCTCTGCTTCCAGTCCTCAGTTCTGGTCAGCACCAGACAGGCCCATAATTA
  CAGAGCCAGGGAAACTGGAACATTTGTCTCCCCTTAGACAGTGGCAGCAGGAAGGTGGGG
  GGTTGTTGCAGAGGAACAGTGTCTCTGAGAGAGGACCTTGGACTTTCTGGGAAT
  ============================================================

Primers for ID: 283
Primer pair 1, product size : 550
  Primer left                            Primer right
  start: 25212011                        start: 25212540
  end  : 25212032                        end  : 25212560
  tm   : 59                              tm   : 57
  seq  : TTTCTGGGCTCCTACTTGTTTC          seq  : ATGAGACACTCTGTGCCAATG

  >283_primer_pair_1_product
  TTTCTGGGCTCCTACTTGTTTCCACAGAGATGGATACTGTGGAGGTCCAGGAAGCAGAGA
  GATGGCTAAGGCTCATCAGGACCGTATGATCTCCCAAGTGTCCAGCTACTGAGTACCACA
  AGGTGATGGGTGGGAGGGTCCTCCCACGGAAGGATACCGCAGTCCCTAGGGGTTGCAAGC
  CCCACATGTTCCACTGGCTGCTAGAGCTACCTACTCAATCAGCCCTGGGCATCACCATCA
  GGTACTCGGCCAAAATGACCTCTCTGCTTCCAGTCCTCAGTTCTGGTCAGCACCAGACAG
  GCCCATAATTACAGAGCCAGGGAAACTGGAACATTTGTCTCCCCTTAGACAGTGGCAGCA
  GGAAGGTGGGGGGTTGTTGCAGAGGAACAGTGTCTCTGAGAGAGGACCTTGGACTTTCTG
  GGAATCTCTGAGCTGCCCGGTTCTCCCCACTGCTGGCACTGTGCCCACAGCCCAAACAGA
  ATGGGGGAGATGGAGGGGCAGGGCTTCTGTGGGAAGCTGCCCTCCACCTCATTGGCACAG
  AGTGTCTCAT
  --------------------

Primer pair 2, product size : 549
  Primer left                            Primer right
  start: 25212012                        start: 25212540
  end  : 25212032                        end  : 25212560
  tm   : 57                              tm   : 57
  seq  : TTCTGGGCTCCTACTTGTTTC           seq  : ATGAGACACTCTGTGCCAATG

  >283_primer_pair_2_product
  TTCTGGGCTCCTACTTGTTTCCACAGAGATGGATACTGTGGAGGTCCAGGAAGCAGAGAG
  ATGGCTAAGGCTCATCAGGACCGTATGATCTCCCAAGTGTCCAGCTACTGAGTACCACAA
  GGTGATGGGTGGGAGGGTCCTCCCACGGAAGGATACCGCAGTCCCTAGGGGTTGCAAGCC
  CCACATGTTCCACTGGCTGCTAGAGCTACCTACTCAATCAGCCCTGGGCATCACCATCAG
  GTACTCGGCCAAAATGACCTCTCTGCTTCCAGTCCTCAGTTCTGGTCAGCACCAGACAGG
  CCCATAATTACAGAGCCAGGGAAACTGGAACATTTGTCTCCCCTTAGACAGTGGCAGCAG
  GAAGGTGGGGGGTTGTTGCAGAGGAACAGTGTCTCTGAGAGAGGACCTTGGACTTTCTGG
  GAATCTCTGAGCTGCCCGGTTCTCCCCACTGCTGGCACTGTGCCCACAGCCCAAACAGAA
  TGGGGGAGATGGAGGGGCAGGGCTTCTGTGGGAAGCTGCCCTCCACCTCATTGGCACAGA
  GTGTCTCAT
  ============================================================

Primers for ID: 284
Primer pair 1, product size : 550
  Primer left                            Primer right
  start: 25212011                        start: 25212540
  end  : 25212032                        end  : 25212560
  tm   : 59                              tm   : 57
  seq  : TTTCTGGGCTCCTACTTGTTTC          seq  : ATGAGACACTCTGTGCCAATG

  >284_primer_pair_1_product
  TTTCTGGGCTCCTACTTGTTTCCACAGAGATGGATACTGTGGAGGTCCAGGAAGCAGAGA
  GATGGCTAAGGCTCATCAGGACCGTATGATCTCCCAAGTGTCCAGCTACTGAGTACCACA
  AGGTGATGGGTGGGAGGGTCCTCCCACGGAAGGATACCGCAGTCCCTAGGGGTTGCAAGC
  CCCACATGTTCCACTGGCTGCTAGAGCTACCTACTCAATCAGCCCTGGGCATCACCATCA
  GGTACTCGGCCAAAATGACCTCTCTGCTTCCAGTCCTCAGTTCTGGTCAGCACCAGACAG
  GCCCATAATTACAGAGCCAGGGAAACTGGAACATTTGTCTCCCCTTAGACAGTGGCAGCA
  GGAAGGTGGGGGGTTGTTGCAGAGGAACAGTGTCTCTGAGAGAGGACCTTGGACTTTCTG
  GGAATCTCTGAGCTGCCCGGTTCTCCCCACTGCTGGCACTGTGCCCACAGCCCAAACAGA
  ATGGGGGAGATGGAGGGGCAGGGCTTCTGTGGGAAGCTGCCCTCCACCTCATTGGCACAG
  AGTGTCTCAT
  --------------------

Primer pair 2, product size : 549
  Primer left                            Primer right
  start: 25212012                        start: 25212540
  end  : 25212032                        end  : 25212560
  tm   : 57                              tm   : 57
  seq  : TTCTGGGCTCCTACTTGTTTC           seq  : ATGAGACACTCTGTGCCAATG

  >284_primer_pair_2_product
  TTCTGGGCTCCTACTTGTTTCCACAGAGATGGATACTGTGGAGGTCCAGGAAGCAGAGAG
  ATGGCTAAGGCTCATCAGGACCGTATGATCTCCCAAGTGTCCAGCTACTGAGTACCACAA
  GGTGATGGGTGGGAGGGTCCTCCCACGGAAGGATACCGCAGTCCCTAGGGGTTGCAAGCC
  CCACATGTTCCACTGGCTGCTAGAGCTACCTACTCAATCAGCCCTGGGCATCACCATCAG
  GTACTCGGCCAAAATGACCTCTCTGCTTCCAGTCCTCAGTTCTGGTCAGCACCAGACAGG
  CCCATAATTACAGAGCCAGGGAAACTGGAACATTTGTCTCCCCTTAGACAGTGGCAGCAG
  GAAGGTGGGGGGTTGTTGCAGAGGAACAGTGTCTCTGAGAGAGGACCTTGGACTTTCTGG
  GAATCTCTGAGCTGCCCGGTTCTCCCCACTGCTGGCACTGTGCCCACAGCCCAAACAGAA
  TGGGGGAGATGGAGGGGCAGGGCTTCTGTGGGAAGCTGCCCTCCACCTCATTGGCACAGA
  GTGTCTCAT
  ============================================================

Primers for ID: 285
Primer pair 1, product size : 527
  Primer left                            Primer right
  start: 25209826                        start: 25210331
  end  : 25209847                        end  : 25210352
  tm   : 61                              tm   : 59
  seq  : AGCAGAATATCTCAGTGGAGGC          seq  : TCTTCTCCAGGACTTTTATGGC

  >285_primer_pair_1_product
  AGCAGAATATCTCAGTGGAGGCCCCTTTCACAGAGCGTGGGTCAGGGCTGCTAGCTTCCA
  GGACACAACAGCAGATAGTGTCTGATGGCATGAAAGCAGATAGCTACAAGGCTCTTGGAC
  CAGGCTAGCACTGGGTCCTGCACCCAGGGAGAGCCACCTCACCTTGACAGGGTTGGCAGG
  AAGGGGCCCTAGAAAGTCAGTAGGATACGGGTAGTCCATCATGGCGAGCACAGTAAATGC
  ATTTCGGGCAAACCCAAAGAGCTGAGTCAGGTCCTTTGGGCTGGAAAGTGATTGACAGGT
  ACCAAAGTTCTGGCTGATGGTGTCATAGGCTGGGAAGAGAGAGGCCAGGAGAAAAGGCTG
  AGGAAACTGCTGGCAAATGTGAAGGGCAAGAATGAATGCCCAAGGTGGGCAGCAGGTGAG
  GAAAGAGTCCCTCACCTCCCTGGAGGAACAAGTCTTTGATTTGCTGAAAGGCATCCCGCA
  CAGCCTGGGCGCACTTGGGACTCTGGCCATAAAAGTCCTGGAGAAGA
  --------------------

Primer pair 2, product size : 529
  Primer left                            Primer right
  start: 25209824                        start: 25210331
  end  : 25209845                        end  : 25210352
  tm   : 61                              tm   : 59
  seq  : GCAGCAGAATATCTCAGTGGAG          seq  : TCTTCTCCAGGACTTTTATGGC

  >285_primer_pair_2_product
  GCAGCAGAATATCTCAGTGGAGGCCCCTTTCACAGAGCGTGGGTCAGGGCTGCTAGCTTC
  CAGGACACAACAGCAGATAGTGTCTGATGGCATGAAAGCAGATAGCTACAAGGCTCTTGG
  ACCAGGCTAGCACTGGGTCCTGCACCCAGGGAGAGCCACCTCACCTTGACAGGGTTGGCA
  GGAAGGGGCCCTAGAAAGTCAGTAGGATACGGGTAGTCCATCATGGCGAGCACAGTAAAT
  GCATTTCGGGCAAACCCAAAGAGCTGAGTCAGGTCCTTTGGGCTGGAAAGTGATTGACAG
  GTACCAAAGTTCTGGCTGATGGTGTCATAGGCTGGGAAGAGAGAGGCCAGGAGAAAAGGC
  TGAGGAAACTGCTGGCAAATGTGAAGGGCAAGAATGAATGCCCAAGGTGGGCAGCAGGTG
  AGGAAAGAGTCCCTCACCTCCCTGGAGGAACAAGTCTTTGATTTGCTGAAAGGCATCCCG
  CACAGCCTGGGCGCACTTGGGACTCTGGCCATAAAAGTCCTGGAGAAGA
  ============================================================

Primers for ID: 286
Primer pair 1, product size : 529
  Primer left                            Primer right
  start: 25209909                        start: 25210417
  end  : 25209929                        end  : 25210437
  tm   : 57                              tm   : 57
  seq  : GATGGCATGAAAGCAGATAGC           seq  : CCAATTCTTCCGAGATGTCAC

  >286_primer_pair_1_product
  GATGGCATGAAAGCAGATAGCTACAAGGCTCTTGGACCAGGCTAGCACTGGGTCCTGCAC
  CCAGGGAGAGCCACCTCACCTTGACAGGGTTGGCAGGAAGGGGCCCTAGAAAGTCAGTAG
  GATACGGGTAGTCCATCATGGCGAGCACAGTAAATGCATTTCGGGCAAACCCAAAGAGCT
  GAGTCAGGTCCTTTGGGCTGGAAAGTGATTGACAGGTACCAAAGTTCTGGCTGATGGTGT
  CATAGGCTGGGAAGAGAGAGGCCAGGAGAAAAGGCTGAGGAAACTGCTGGCAAATGTGAA
  GGGCAAGAATGAATGCCCAAGGTGGGCAGCAGGTGAGGAAAGAGTCCCTCACCTCCCTGG
  AGGAACAAGTCTTTGATTTGCTGAAAGGCATCCCGCACAGCCTGGGCGCACTTGGGACTC
  TGGCCATAAAAGTCCTGGAGAAGAGACCAAGGTTGCTGCTGCCATTCTTGCACTGGCCTG
  GGGTACCCAAGTCCCCTCACTCACCGCTGTGACATCTCGGAAGAATTGG
  --------------------

Primer pair 2, product size : 528
  Primer left                            Primer right
  start: 25209909                        start: 25210415
  end  : 25209929                        end  : 25210436
  tm   : 57                              tm   : 59
  seq  : GATGGCATGAAAGCAGATAGC           seq  : CAATTCTTCCGAGATGTCACAG

  >286_primer_pair_2_product
  GATGGCATGAAAGCAGATAGCTACAAGGCTCTTGGACCAGGCTAGCACTGGGTCCTGCAC
  CCAGGGAGAGCCACCTCACCTTGACAGGGTTGGCAGGAAGGGGCCCTAGAAAGTCAGTAG
  GATACGGGTAGTCCATCATGGCGAGCACAGTAAATGCATTTCGGGCAAACCCAAAGAGCT
  GAGTCAGGTCCTTTGGGCTGGAAAGTGATTGACAGGTACCAAAGTTCTGGCTGATGGTGT
  CATAGGCTGGGAAGAGAGAGGCCAGGAGAAAAGGCTGAGGAAACTGCTGGCAAATGTGAA
  GGGCAAGAATGAATGCCCAAGGTGGGCAGCAGGTGAGGAAAGAGTCCCTCACCTCCCTGG
  AGGAACAAGTCTTTGATTTGCTGAAAGGCATCCCGCACAGCCTGGGCGCACTTGGGACTC
  TGGCCATAAAAGTCCTGGAGAAGAGACCAAGGTTGCTGCTGCCATTCTTGCACTGGCCTG
  GGGTACCCAAGTCCCCTCACTCACCGCTGTGACATCTCGGAAGAATTG
  ============================================================

Primers for ID: 287
Primer pair 1, product size : 529
  Primer left                            Primer right
  start: 25209909                        start: 25210417
  end  : 25209929                        end  : 25210437
  tm   : 57                              tm   : 57
  seq  : GATGGCATGAAAGCAGATAGC           seq  : CCAATTCTTCCGAGATGTCAC

  >287_primer_pair_1_product
  GATGGCATGAAAGCAGATAGCTACAAGGCTCTTGGACCAGGCTAGCACTGGGTCCTGCAC
  CCAGGGAGAGCCACCTCACCTTGACAGGGTTGGCAGGAAGGGGCCCTAGAAAGTCAGTAG
  GATACGGGTAGTCCATCATGGCGAGCACAGTAAATGCATTTCGGGCAAACCCAAAGAGCT
  GAGTCAGGTCCTTTGGGCTGGAAAGTGATTGACAGGTACCAAAGTTCTGGCTGATGGTGT
  CATAGGCTGGGAAGAGAGAGGCCAGGAGAAAAGGCTGAGGAAACTGCTGGCAAATGTGAA
  GGGCAAGAATGAATGCCCAAGGTGGGCAGCAGGTGAGGAAAGAGTCCCTCACCTCCCTGG
  AGGAACAAGTCTTTGATTTGCTGAAAGGCATCCCGCACAGCCTGGGCGCACTTGGGACTC
  TGGCCATAAAAGTCCTGGAGAAGAGACCAAGGTTGCTGCTGCCATTCTTGCACTGGCCTG
  GGGTACCCAAGTCCCCTCACTCACCGCTGTGACATCTCGGAAGAATTGG
  --------------------

Primer pair 2, product size : 528
  Primer left                            Primer right
  start: 25209909                        start: 25210415
  end  : 25209929                        end  : 25210436
  tm   : 57                              tm   : 59
  seq  : GATGGCATGAAAGCAGATAGC           seq  : CAATTCTTCCGAGATGTCACAG

  >287_primer_pair_2_product
  GATGGCATGAAAGCAGATAGCTACAAGGCTCTTGGACCAGGCTAGCACTGGGTCCTGCAC
  CCAGGGAGAGCCACCTCACCTTGACAGGGTTGGCAGGAAGGGGCCCTAGAAAGTCAGTAG
  GATACGGGTAGTCCATCATGGCGAGCACAGTAAATGCATTTCGGGCAAACCCAAAGAGCT
  GAGTCAGGTCCTTTGGGCTGGAAAGTGATTGACAGGTACCAAAGTTCTGGCTGATGGTGT
  CATAGGCTGGGAAGAGAGAGGCCAGGAGAAAAGGCTGAGGAAACTGCTGGCAAATGTGAA
  GGGCAAGAATGAATGCCCAAGGTGGGCAGCAGGTGAGGAAAGAGTCCCTCACCTCCCTGG
  AGGAACAAGTCTTTGATTTGCTGAAAGGCATCCCGCACAGCCTGGGCGCACTTGGGACTC
  TGGCCATAAAAGTCCTGGAGAAGAGACCAAGGTTGCTGCTGCCATTCTTGCACTGGCCTG
  GGGTACCCAAGTCCCCTCACTCACCGCTGTGACATCTCGGAAGAATTG
  ============================================================

Primers for ID: 288
Primer pair 1, product size : 549
  Primer left                            Primer right
  start: 25210016                        start: 25210543
  end  : 25210036                        end  : 25210564
  tm   : 57                              tm   : 61
  seq  : AGAAAGTCAGTAGGATACGGG           seq  : TGCTCTGTACTCCCAGTTATGG

  >288_primer_pair_1_product
  AGAAAGTCAGTAGGATACGGGTAGTCCATCATGGCGAGCACAGTAAATGCATTTCGGGCA
  AACCCAAAGAGCTGAGTCAGGTCCTTTGGGCTGGAAAGTGATTGACAGGTACCAAAGTTC
  TGGCTGATGGTGTCATAGGCTGGGAAGAGAGAGGCCAGGAGAAAAGGCTGAGGAAACTGC
  TGGCAAATGTGAAGGGCAAGAATGAATGCCCAAGGTGGGCAGCAGGTGAGGAAAGAGTCC
  CTCACCTCCCTGGAGGAACAAGTCTTTGATTTGCTGAAAGGCATCCCGCACAGCCTGGGC
  GCACTTGGGACTCTGGCCATAAAAGTCCTGGAGAAGAGACCAAGGTTGCTGCTGCCATTC
  TTGCACTGGCCTGGGGTACCCAAGTCCCCTCACTCACCGCTGTGACATCTCGGAAGAATT
  GGTAGGAGTCCCCAAGGCCTGCAACAGCTACAACAGGAGCGCTGGCTGCCAGTGCCCCAG
  CCACCAGGTGGGGGTACTTCATCCTCATGTAGGCACTCAGCATCCCCCCATAACTGGGAG
  TACAGAGCA
  --------------------

Primer pair 2, product size : 500
  Primer left                            Primer right
  start: 25210074                        start: 25210552
  end  : 25210093                        end  : 25210573
  tm   : 55                              tm   : 61
  seq  : CAAACCCAAAGAGCTGAGTC            seq  : CATGATCTGTGCTCTGTACTCC

  >288_primer_pair_2_product
  CAAACCCAAAGAGCTGAGTCAGGTCCTTTGGGCTGGAAAGTGATTGACAGGTACCAAAGT
  TCTGGCTGATGGTGTCATAGGCTGGGAAGAGAGAGGCCAGGAGAAAAGGCTGAGGAAACT
  GCTGGCAAATGTGAAGGGCAAGAATGAATGCCCAAGGTGGGCAGCAGGTGAGGAAAGAGT
  CCCTCACCTCCCTGGAGGAACAAGTCTTTGATTTGCTGAAAGGCATCCCGCACAGCCTGG
  GCGCACTTGGGACTCTGGCCATAAAAGTCCTGGAGAAGAGACCAAGGTTGCTGCTGCCAT
  TCTTGCACTGGCCTGGGGTACCCAAGTCCCCTCACTCACCGCTGTGACATCTCGGAAGAA
  TTGGTAGGAGTCCCCAAGGCCTGCAACAGCTACAACAGGAGCGCTGGCTGCCAGTGCCCC
  AGCCACCAGGTGGGGGTACTTCATCCTCATGTAGGCACTCAGCATCCCCCCATAACTGGG
  AGTACAGAGCACAGATCATG
  ============================================================

Primers for ID: 289
Primer pair 1, product size : 563
  Primer left                            Primer right
  start: 25210016                        start: 25210558
  end  : 25210036                        end  : 25210578
  tm   : 57                              tm   : 57
  seq  : AGAAAGTCAGTAGGATACGGG           seq  : ACAACCATGATCTGTGCTCTG

  >289_primer_pair_1_product
  AGAAAGTCAGTAGGATACGGGTAGTCCATCATGGCGAGCACAGTAAATGCATTTCGGGCA
  AACCCAAAGAGCTGAGTCAGGTCCTTTGGGCTGGAAAGTGATTGACAGGTACCAAAGTTC
  TGGCTGATGGTGTCATAGGCTGGGAAGAGAGAGGCCAGGAGAAAAGGCTGAGGAAACTGC
  TGGCAAATGTGAAGGGCAAGAATGAATGCCCAAGGTGGGCAGCAGGTGAGGAAAGAGTCC
  CTCACCTCCCTGGAGGAACAAGTCTTTGATTTGCTGAAAGGCATCCCGCACAGCCTGGGC
  GCACTTGGGACTCTGGCCATAAAAGTCCTGGAGAAGAGACCAAGGTTGCTGCTGCCATTC
  TTGCACTGGCCTGGGGTACCCAAGTCCCCTCACTCACCGCTGTGACATCTCGGAAGAATT
  GGTAGGAGTCCCCAAGGCCTGCAACAGCTACAACAGGAGCGCTGGCTGCCAGTGCCCCAG
  CCACCAGGTGGGGGTACTTCATCCTCATGTAGGCACTCAGCATCCCCCCATAACTGGGAG
  TACAGAGCACAGATCATGGTTGT
  --------------------

Primer pair 2, product size : 549
  Primer left                            Primer right
  start: 25210016                        start: 25210543
  end  : 25210036                        end  : 25210564
  tm   : 57                              tm   : 61
  seq  : AGAAAGTCAGTAGGATACGGG           seq  : TGCTCTGTACTCCCAGTTATGG

  >289_primer_pair_2_product
  AGAAAGTCAGTAGGATACGGGTAGTCCATCATGGCGAGCACAGTAAATGCATTTCGGGCA
  AACCCAAAGAGCTGAGTCAGGTCCTTTGGGCTGGAAAGTGATTGACAGGTACCAAAGTTC
  TGGCTGATGGTGTCATAGGCTGGGAAGAGAGAGGCCAGGAGAAAAGGCTGAGGAAACTGC
  TGGCAAATGTGAAGGGCAAGAATGAATGCCCAAGGTGGGCAGCAGGTGAGGAAAGAGTCC
  CTCACCTCCCTGGAGGAACAAGTCTTTGATTTGCTGAAAGGCATCCCGCACAGCCTGGGC
  GCACTTGGGACTCTGGCCATAAAAGTCCTGGAGAAGAGACCAAGGTTGCTGCTGCCATTC
  TTGCACTGGCCTGGGGTACCCAAGTCCCCTCACTCACCGCTGTGACATCTCGGAAGAATT
  GGTAGGAGTCCCCAAGGCCTGCAACAGCTACAACAGGAGCGCTGGCTGCCAGTGCCCCAG
  CCACCAGGTGGGGGTACTTCATCCTCATGTAGGCACTCAGCATCCCCCCATAACTGGGAG
  TACAGAGCA
  ============================================================

Primers for ID: 290
Primer pair 1, product size : 562
  Primer left                            Primer right
  start: 25210129                        start: 25210670
  end  : 25210149                        end  : 25210690
  tm   : 57                              tm   : 57
  seq  : AAAGTTCTGGCTGATGGTGTC           seq  : AGTTTCAGGTCCTTCTTTCCC

  >290_primer_pair_1_product
  AAAGTTCTGGCTGATGGTGTCATAGGCTGGGAAGAGAGAGGCCAGGAGAAAAGGCTGAGG
  AAACTGCTGGCAAATGTGAAGGGCAAGAATGAATGCCCAAGGTGGGCAGCAGGTGAGGAA
  AGAGTCCCTCACCTCCCTGGAGGAACAAGTCTTTGATTTGCTGAAAGGCATCCCGCACAG
  CCTGGGCGCACTTGGGACTCTGGCCATAAAAGTCCTGGAGAAGAGACCAAGGTTGCTGCT
  GCCATTCTTGCACTGGCCTGGGGTACCCAAGTCCCCTCACTCACCGCTGTGACATCTCGG
  AAGAATTGGTAGGAGTCCCCAAGGCCTGCAACAGCTACAACAGGAGCGCTGGCTGCCAGT
  GCCCCAGCCACCAGGTGGGGGTACTTCATCCTCATGTAGGCACTCAGCATCCCCCCATAA
  CTGGGAGTACAGAGCACAGATCATGGTTGTGGGAAGCTGCCCACAACTCAGGCGAGCAGC
  CTCACTGTCCTCCAGGCTGAGGTGCTAGGCTGCTCTTTCCCTGCTCAGAACGCCCAAGGG
  TGGGAAAGAAGGACCTGAAACT
  --------------------

Primer pair 2, product size : 558
  Primer left                            Primer right
  start: 25210133                        start: 25210670
  end  : 25210153                        end  : 25210690
  tm   : 57                              tm   : 57
  seq  : TTCTGGCTGATGGTGTCATAG           seq  : AGTTTCAGGTCCTTCTTTCCC

  >290_primer_pair_2_product
  TTCTGGCTGATGGTGTCATAGGCTGGGAAGAGAGAGGCCAGGAGAAAAGGCTGAGGAAAC
  TGCTGGCAAATGTGAAGGGCAAGAATGAATGCCCAAGGTGGGCAGCAGGTGAGGAAAGAG
  TCCCTCACCTCCCTGGAGGAACAAGTCTTTGATTTGCTGAAAGGCATCCCGCACAGCCTG
  GGCGCACTTGGGACTCTGGCCATAAAAGTCCTGGAGAAGAGACCAAGGTTGCTGCTGCCA
  TTCTTGCACTGGCCTGGGGTACCCAAGTCCCCTCACTCACCGCTGTGACATCTCGGAAGA
  ATTGGTAGGAGTCCCCAAGGCCTGCAACAGCTACAACAGGAGCGCTGGCTGCCAGTGCCC
  CAGCCACCAGGTGGGGGTACTTCATCCTCATGTAGGCACTCAGCATCCCCCCATAACTGG
  GAGTACAGAGCACAGATCATGGTTGTGGGAAGCTGCCCACAACTCAGGCGAGCAGCCTCA
  CTGTCCTCCAGGCTGAGGTGCTAGGCTGCTCTTTCCCTGCTCAGAACGCCCAAGGGTGGG
  AAAGAAGGACCTGAAACT
  ============================================================

Primers for ID: 291
Primer pair 1, product size : 562
  Primer left                            Primer right
  start: 25210129                        start: 25210670
  end  : 25210149                        end  : 25210690
  tm   : 57                              tm   : 57
  seq  : AAAGTTCTGGCTGATGGTGTC           seq  : AGTTTCAGGTCCTTCTTTCCC

  >291_primer_pair_1_product
  AAAGTTCTGGCTGATGGTGTCATAGGCTGGGAAGAGAGAGGCCAGGAGAAAAGGCTGAGG
  AAACTGCTGGCAAATGTGAAGGGCAAGAATGAATGCCCAAGGTGGGCAGCAGGTGAGGAA
  AGAGTCCCTCACCTCCCTGGAGGAACAAGTCTTTGATTTGCTGAAAGGCATCCCGCACAG
  CCTGGGCGCACTTGGGACTCTGGCCATAAAAGTCCTGGAGAAGAGACCAAGGTTGCTGCT
  GCCATTCTTGCACTGGCCTGGGGTACCCAAGTCCCCTCACTCACCGCTGTGACATCTCGG
  AAGAATTGGTAGGAGTCCCCAAGGCCTGCAACAGCTACAACAGGAGCGCTGGCTGCCAGT
  GCCCCAGCCACCAGGTGGGGGTACTTCATCCTCATGTAGGCACTCAGCATCCCCCCATAA
  CTGGGAGTACAGAGCACAGATCATGGTTGTGGGAAGCTGCCCACAACTCAGGCGAGCAGC
  CTCACTGTCCTCCAGGCTGAGGTGCTAGGCTGCTCTTTCCCTGCTCAGAACGCCCAAGGG
  TGGGAAAGAAGGACCTGAAACT
  --------------------

Primer pair 2, product size : 558
  Primer left                            Primer right
  start: 25210133                        start: 25210670
  end  : 25210153                        end  : 25210690
  tm   : 57                              tm   : 57
  seq  : TTCTGGCTGATGGTGTCATAG           seq  : AGTTTCAGGTCCTTCTTTCCC

  >291_primer_pair_2_product
  TTCTGGCTGATGGTGTCATAGGCTGGGAAGAGAGAGGCCAGGAGAAAAGGCTGAGGAAAC
  TGCTGGCAAATGTGAAGGGCAAGAATGAATGCCCAAGGTGGGCAGCAGGTGAGGAAAGAG
  TCCCTCACCTCCCTGGAGGAACAAGTCTTTGATTTGCTGAAAGGCATCCCGCACAGCCTG
  GGCGCACTTGGGACTCTGGCCATAAAAGTCCTGGAGAAGAGACCAAGGTTGCTGCTGCCA
  TTCTTGCACTGGCCTGGGGTACCCAAGTCCCCTCACTCACCGCTGTGACATCTCGGAAGA
  ATTGGTAGGAGTCCCCAAGGCCTGCAACAGCTACAACAGGAGCGCTGGCTGCCAGTGCCC
  CAGCCACCAGGTGGGGGTACTTCATCCTCATGTAGGCACTCAGCATCCCCCCATAACTGG
  GAGTACAGAGCACAGATCATGGTTGTGGGAAGCTGCCCACAACTCAGGCGAGCAGCCTCA
  CTGTCCTCCAGGCTGAGGTGCTAGGCTGCTCTTTCCCTGCTCAGAACGCCCAAGGGTGGG
  AAAGAAGGACCTGAAACT
  ============================================================

Primers for ID: 292
Primer pair 1, product size : 544
  Primer left                            Primer right
  start: 25210277                        start: 25210799
  end  : 25210297                        end  : 25210820
  tm   : 55                              tm   : 59
  seq  : GTCTTTGATTTGCTGAAAGGC           seq  : GGGACAATTCCTTCTGACTTTG

  >292_primer_pair_1_product
  GTCTTTGATTTGCTGAAAGGCATCCCGCACAGCCTGGGCGCACTTGGGACTCTGGCCATA
  AAAGTCCTGGAGAAGAGACCAAGGTTGCTGCTGCCATTCTTGCACTGGCCTGGGGTACCC
  AAGTCCCCTCACTCACCGCTGTGACATCTCGGAAGAATTGGTAGGAGTCCCCAAGGCCTG
  CAACAGCTACAACAGGAGCGCTGGCTGCCAGTGCCCCAGCCACCAGGTGGGGGTACTTCA
  TCCTCATGTAGGCACTCAGCATCCCCCCATAACTGGGAGTACAGAGCACAGATCATGGTT
  GTGGGAAGCTGCCCACAACTCAGGCGAGCAGCCTCACTGTCCTCCAGGCTGAGGTGCTAG
  GCTGCTCTTTCCCTGCTCAGAACGCCCAAGGGTGGGAAAGAAGGACCTGAAACTGTCAGG
  CCCACACACCCTGATCCCAGGGCCAAGGCAGATACAGCCTTCACTGGGAGAAGGCACCTG
  TGGGTGCCCTGCCCTGACCCAGCAATGAAGACATTGCAGAGACAAAGTCAGAAGGAATTG
  TCCC
  --------------------

Primer pair 2, product size : 608
  Primer left                            Primer right
  start: 25210213                        start: 25210799
  end  : 25210232                        end  : 25210820
  tm   : 53                              tm   : 59
  seq  : AAGAATGAATGCCCAAGGTG            seq  : GGGACAATTCCTTCTGACTTTG

  >292_primer_pair_2_product
  AAGAATGAATGCCCAAGGTGGGCAGCAGGTGAGGAAAGAGTCCCTCACCTCCCTGGAGGA
  ACAAGTCTTTGATTTGCTGAAAGGCATCCCGCACAGCCTGGGCGCACTTGGGACTCTGGC
  CATAAAAGTCCTGGAGAAGAGACCAAGGTTGCTGCTGCCATTCTTGCACTGGCCTGGGGT
  ACCCAAGTCCCCTCACTCACCGCTGTGACATCTCGGAAGAATTGGTAGGAGTCCCCAAGG
  CCTGCAACAGCTACAACAGGAGCGCTGGCTGCCAGTGCCCCAGCCACCAGGTGGGGGTAC
  TTCATCCTCATGTAGGCACTCAGCATCCCCCCATAACTGGGAGTACAGAGCACAGATCAT
  GGTTGTGGGAAGCTGCCCACAACTCAGGCGAGCAGCCTCACTGTCCTCCAGGCTGAGGTG
  CTAGGCTGCTCTTTCCCTGCTCAGAACGCCCAAGGGTGGGAAAGAAGGACCTGAAACTGT
  CAGGCCCACACACCCTGATCCCAGGGCCAAGGCAGATACAGCCTTCACTGGGAGAAGGCA
  CCTGTGGGTGCCCTGCCCTGACCCAGCAATGAAGACATTGCAGAGACAAAGTCAGAAGGA
  ATTGTCCC
  ============================================================

Primers for ID: 293
Primer pair 1, product size : 544
  Primer left                            Primer right
  start: 25210277                        start: 25210799
  end  : 25210297                        end  : 25210820
  tm   : 55                              tm   : 59
  seq  : GTCTTTGATTTGCTGAAAGGC           seq  : GGGACAATTCCTTCTGACTTTG

  >293_primer_pair_1_product
  GTCTTTGATTTGCTGAAAGGCATCCCGCACAGCCTGGGCGCACTTGGGACTCTGGCCATA
  AAAGTCCTGGAGAAGAGACCAAGGTTGCTGCTGCCATTCTTGCACTGGCCTGGGGTACCC
  AAGTCCCCTCACTCACCGCTGTGACATCTCGGAAGAATTGGTAGGAGTCCCCAAGGCCTG
  CAACAGCTACAACAGGAGCGCTGGCTGCCAGTGCCCCAGCCACCAGGTGGGGGTACTTCA
  TCCTCATGTAGGCACTCAGCATCCCCCCATAACTGGGAGTACAGAGCACAGATCATGGTT
  GTGGGAAGCTGCCCACAACTCAGGCGAGCAGCCTCACTGTCCTCCAGGCTGAGGTGCTAG
  GCTGCTCTTTCCCTGCTCAGAACGCCCAAGGGTGGGAAAGAAGGACCTGAAACTGTCAGG
  CCCACACACCCTGATCCCAGGGCCAAGGCAGATACAGCCTTCACTGGGAGAAGGCACCTG
  TGGGTGCCCTGCCCTGACCCAGCAATGAAGACATTGCAGAGACAAAGTCAGAAGGAATTG
  TCCC
  --------------------

Primer pair 2, product size : 545
  Primer left                            Primer right
  start: 25210276                        start: 25210799
  end  : 25210297                        end  : 25210820
  tm   : 57                              tm   : 59
  seq  : AGTCTTTGATTTGCTGAAAGGC          seq  : GGGACAATTCCTTCTGACTTTG

  >293_primer_pair_2_product
  AGTCTTTGATTTGCTGAAAGGCATCCCGCACAGCCTGGGCGCACTTGGGACTCTGGCCAT
  AAAAGTCCTGGAGAAGAGACCAAGGTTGCTGCTGCCATTCTTGCACTGGCCTGGGGTACC
  CAAGTCCCCTCACTCACCGCTGTGACATCTCGGAAGAATTGGTAGGAGTCCCCAAGGCCT
  GCAACAGCTACAACAGGAGCGCTGGCTGCCAGTGCCCCAGCCACCAGGTGGGGGTACTTC
  ATCCTCATGTAGGCACTCAGCATCCCCCCATAACTGGGAGTACAGAGCACAGATCATGGT
  TGTGGGAAGCTGCCCACAACTCAGGCGAGCAGCCTCACTGTCCTCCAGGCTGAGGTGCTA
  GGCTGCTCTTTCCCTGCTCAGAACGCCCAAGGGTGGGAAAGAAGGACCTGAAACTGTCAG
  GCCCACACACCCTGATCCCAGGGCCAAGGCAGATACAGCCTTCACTGGGAGAAGGCACCT
  GTGGGTGCCCTGCCCTGACCCAGCAATGAAGACATTGCAGAGACAAAGTCAGAAGGAATT
  GTCCC
  ============================================================

Primers for ID: 294
Primer pair 1, product size : 521
  Primer left                            Primer right
  start: 25210417                        start: 25210917
  end  : 25210437                        end  : 25210937
  tm   : 57                              tm   : 57
  seq  : GTGACATCTCGGAAGAATTGG           seq  : TCTCAACATCCTATGCCTACC

  >294_primer_pair_1_product
  GTGACATCTCGGAAGAATTGGTAGGAGTCCCCAAGGCCTGCAACAGCTACAACAGGAGCG
  CTGGCTGCCAGTGCCCCAGCCACCAGGTGGGGGTACTTCATCCTCATGTAGGCACTCAGC
  ATCCCCCCATAACTGGGAGTACAGAGCACAGATCATGGTTGTGGGAAGCTGCCCACAACT
  CAGGCGAGCAGCCTCACTGTCCTCCAGGCTGAGGTGCTAGGCTGCTCTTTCCCTGCTCAG
  AACGCCCAAGGGTGGGAAAGAAGGACCTGAAACTGTCAGGCCCACACACCCTGATCCCAG
  GGCCAAGGCAGATACAGCCTTCACTGGGAGAAGGCACCTGTGGGTGCCCTGCCCTGACCC
  AGCAATGAAGACATTGCAGAGACAAAGTCAGAAGGAATTGTCCCACTAGTGGGAACAACA
  TAGCATACACTGCCTATGAGGTCCACTCAAGGAGGGCTTCCAGAAGGAGGTAAAGCTAGA
  CCCCGCCCTTCCACATGTGGGGTAGGCATAGGATGTTGAGA
  --------------------

Primer pair 2, product size : 525
  Primer left                            Primer right
  start: 25210417                        start: 25210921
  end  : 25210437                        end  : 25210941
  tm   : 57                              tm   : 57
  seq  : GTGACATCTCGGAAGAATTGG           seq  : ACAGTCTCAACATCCTATGCC

  >294_primer_pair_2_product
  GTGACATCTCGGAAGAATTGGTAGGAGTCCCCAAGGCCTGCAACAGCTACAACAGGAGCG
  CTGGCTGCCAGTGCCCCAGCCACCAGGTGGGGGTACTTCATCCTCATGTAGGCACTCAGC
  ATCCCCCCATAACTGGGAGTACAGAGCACAGATCATGGTTGTGGGAAGCTGCCCACAACT
  CAGGCGAGCAGCCTCACTGTCCTCCAGGCTGAGGTGCTAGGCTGCTCTTTCCCTGCTCAG
  AACGCCCAAGGGTGGGAAAGAAGGACCTGAAACTGTCAGGCCCACACACCCTGATCCCAG
  GGCCAAGGCAGATACAGCCTTCACTGGGAGAAGGCACCTGTGGGTGCCCTGCCCTGACCC
  AGCAATGAAGACATTGCAGAGACAAAGTCAGAAGGAATTGTCCCACTAGTGGGAACAACA
  TAGCATACACTGCCTATGAGGTCCACTCAAGGAGGGCTTCCAGAAGGAGGTAAAGCTAGA
  CCCCGCCCTTCCACATGTGGGGTAGGCATAGGATGTTGAGACTGT
  ============================================================

Primers for ID: 295
Primer pair 1, product size : 555
  Primer left                            Primer right
  start: 25210417                        start: 25210951
  end  : 25210437                        end  : 25210971
  tm   : 57                              tm   : 57
  seq  : GTGACATCTCGGAAGAATTGG           seq  : TATACAAGGAGGGCCAAAGAG

  >295_primer_pair_1_product
  GTGACATCTCGGAAGAATTGGTAGGAGTCCCCAAGGCCTGCAACAGCTACAACAGGAGCG
  CTGGCTGCCAGTGCCCCAGCCACCAGGTGGGGGTACTTCATCCTCATGTAGGCACTCAGC
  ATCCCCCCATAACTGGGAGTACAGAGCACAGATCATGGTTGTGGGAAGCTGCCCACAACT
  CAGGCGAGCAGCCTCACTGTCCTCCAGGCTGAGGTGCTAGGCTGCTCTTTCCCTGCTCAG
  AACGCCCAAGGGTGGGAAAGAAGGACCTGAAACTGTCAGGCCCACACACCCTGATCCCAG
  GGCCAAGGCAGATACAGCCTTCACTGGGAGAAGGCACCTGTGGGTGCCCTGCCCTGACCC
[truncated: 561,417 more chars]
