# Supplementary material for: Automated design of genomic Southern blot probes
Source: BMC Genomics. 2010 Jan 29;11:74. doi: 10.1186/1471-2164-11-74 (PMC2830989; doi:10.1186/1471-2164-11-74)
Supplement: Additional file 1 — Software package for automated design of genomic Southern blot probes. Archive of all the components of the pipeline packaged using "tar", and subsequently compressed with "gzip". Includes source code, example configuration files, example output, and a user's guide for installation. [file 1471-2164-11-74-S1.GZ › southern_blot_design/docs/example_run_output/test_probe_search.html]

test - Southern blot probe search results

```
./analyse_probe_search : Mon Aug 24 15:09:21 2009
Connected to host: host, as user: user
Database       : southern_blot_design

Fetched probe design: test (id: 1)
  assembly      : NCBIM37
  bias          : 5prime
  chromosome    : 2
  description   : testing
  end           : 25212733
  id            : 1
  name          : test
  start         : 25209733
  strand        : 1
  -------------

Design window length: 3001bp

All jobs for probe design are successful - total: 23
Fetched conf : Exonerate mouse_NCBIM37 (id: 1)
  Genome file(s) at: /data/blastdb/Ensembl/Mouse/NCBIM37/genome/softmasked_dusted
Fetched 458 putative probes to analyse

Selection criteria to rank probes:
  Minimum score ratio             : 10
  (i.e. self-hit must score at least 10 times higher than the next best hit)
  Maximum percent repetitive bases: 5
  Favour probes to the 5prime end of design window
  ------------------------

Putative probes without hits                    : 0
Putative probes with single_self_hit            : 458/458
Putative probes that are unique                 : 301/458
Putative probes exceeding selection criteria    : 458/458 (both unique and not unique)
Putative probes with successfully-picked primers: 439/458

Identified 0 'redundant' probes exceeding selection criteria
(but wholly contained by other probes, and higher repetitive DNA content)


UNIQUE (YELLOW), PASSED (GREEN) AND FAILED PUTATIVE PROBES IN THE DESIGN WINDOW
-------------------------------------------------------------------------------

  
UNIQUE PUTATIVE PROBES
----------------------

Ranked by repetitive DNA content and length

  1 ID:      1 length: 1300 UNIQUE  actual: 6500 %repetitive DNA   -  distance:     0  start: 25209733 end: 25211032
  2 ID:      2 length: 1300 UNIQUE  actual: 6500 %repetitive DNA   -  distance:   130  start: 25209863 end: 25211162
  3 ID:     12 length: 1300 UNIQUE  actual: 6500 %repetitive DNA   -  distance:  1430  start: 25211163 end: 25212462
  4 ID:     13 length: 1300 UNIQUE  actual: 6500 %repetitive DNA   -  distance:  1560  start: 25211293 end: 25212592
  5 ID:     14 length: 1300 UNIQUE  actual: 6500 %repetitive DNA   -  distance:  1690  start: 25211423 end: 25212722
  6 ID:     15 length: 1250 UNIQUE  actual: 6250 %repetitive DNA   -  distance:     0  start: 25209733 end: 25210982
  7 ID:     16 length: 1250 UNIQUE  actual: 6250 %repetitive DNA   -  distance:   125  start: 25209858 end: 25211107
  8 ID:     27 length: 1250 UNIQUE  actual: 6250 %repetitive DNA   -  distance:  1500  start: 25211233 end: 25212482
  9 ID:     28 length: 1250 UNIQUE  actual: 6250 %repetitive DNA   -  distance:  1625  start: 25211358 end: 25212607
 10 ID:     29 length: 1250 UNIQUE  actual: 6250 %repetitive DNA   -  distance:  1750  start: 25211483 end: 25212732
 11 ID:     30 length: 1200 UNIQUE  actual: 6000 %repetitive DNA   -  distance:     0  start: 25209733 end: 25210932
 12 ID:     31 length: 1200 UNIQUE  actual: 6000 %repetitive DNA   -  distance:   120  start: 25209853 end: 25211052
 13 ID:     32 length: 1200 UNIQUE  actual: 6000 %repetitive DNA   -  distance:   240  start: 25209973 end: 25211172
 14 ID:     42 length: 1200 UNIQUE  actual: 6000 %repetitive DNA   -  distance:  1440  start: 25211173 end: 25212372
 15 ID:     43 length: 1200 UNIQUE  actual: 6000 %repetitive DNA   -  distance:  1560  start: 25211293 end: 25212492
 16 ID:     44 length: 1200 UNIQUE  actual: 6000 %repetitive DNA   -  distance:  1680  start: 25211413 end: 25212612
 17 ID:     45 length: 1200 UNIQUE  actual: 6000 %repetitive DNA   -  distance:  1800  start: 25211533 end: 25212732
 18 ID:     46 length: 1150 UNIQUE  actual: 5750 %repetitive DNA   -  distance:     0  start: 25209733 end: 25210882
 19 ID:     47 length: 1150 UNIQUE  actual: 5750 %repetitive DNA   -  distance:   115  start: 25209848 end: 25210997
 20 ID:     48 length: 1150 UNIQUE  actual: 5750 %repetitive DNA   -  distance:   230  start: 25209963 end: 25211112
 21 ID:     59 length: 1150 UNIQUE  actual: 5750 %repetitive DNA   -  distance:  1495  start: 25211228 end: 25212377
 22 ID:     60 length: 1150 UNIQUE  actual: 5750 %repetitive DNA   -  distance:  1610  start: 25211343 end: 25212492
 23 ID:     61 length: 1150 UNIQUE  actual: 5750 %repetitive DNA   -  distance:  1725  start: 25211458 end: 25212607
 24 ID:     62 length: 1150 UNIQUE  actual: 5750 %repetitive DNA   -  distance:  1840  start: 25211573 end: 25212722
 25 ID:     63 length: 1100 UNIQUE  actual: 5500 %repetitive DNA   -  distance:     0  start: 25209733 end: 25210832
 26 ID:     64 length: 1100 UNIQUE  actual: 5500 %repetitive DNA   -  distance:   110  start: 25209843 end: 25210942
 27 ID:     65 length: 1100 UNIQUE  actual: 5500 %repetitive DNA   -  distance:   220  start: 25209953 end: 25211052
 28 ID:     66 length: 1100 UNIQUE  actual: 5500 %repetitive DNA   -  distance:   330  start: 25210063 end: 25211162
 29 ID:     76 length: 1100 UNIQUE  actual: 5500 %repetitive DNA   -  distance:  1430  start: 25211163 end: 25212262
 30 ID:     77 length: 1100 UNIQUE  actual: 5500 %repetitive DNA   -  distance:  1540  start: 25211273 end: 25212372
 31 ID:     78 length: 1100 UNIQUE  actual: 5500 %repetitive DNA   -  distance:  1650  start: 25211383 end: 25212482
 32 ID:     79 length: 1100 UNIQUE  actual: 5500 %repetitive DNA   -  distance:  1760  start: 25211493 end: 25212592
 33 ID:     80 length: 1100 UNIQUE  actual: 5500 %repetitive DNA   -  distance:  1870  start: 25211603 end: 25212702
 34 ID:     81 length: 1050 UNIQUE  actual: 5250 %repetitive DNA   -  distance:     0  start: 25209733 end: 25210782
 35 ID:     82 length: 1050 UNIQUE  actual: 5250 %repetitive DNA   -  distance:   105  start: 25209838 end: 25210887
 36 ID:     83 length: 1050 UNIQUE  actual: 5250 %repetitive DNA   -  distance:   210  start: 25209943 end: 25210992
 37 ID:     84 length: 1050 UNIQUE  actual: 5250 %repetitive DNA   -  distance:   315  start: 25210048 end: 25211097
 38 ID:     95 length: 1050 UNIQUE  actual: 5250 %repetitive DNA   -  distance:  1470  start: 25211203 end: 25212252
 39 ID:     96 length: 1050 UNIQUE  actual: 5250 %repetitive DNA   -  distance:  1575  start: 25211308 end: 25212357
 40 ID:     97 length: 1050 UNIQUE  actual: 5250 %repetitive DNA   -  distance:  1680  start: 25211413 end: 25212462
 41 ID:     98 length: 1050 UNIQUE  actual: 5250 %repetitive DNA   -  distance:  1785  start: 25211518 end: 25212567
 42 ID:     99 length: 1050 UNIQUE  actual: 5250 %repetitive DNA   -  distance:  1890  start: 25211623 end: 25212672
 43 ID:    100 length: 1000 UNIQUE  actual: 5000 %repetitive DNA   -  distance:     0  start: 25209733 end: 25210732
 44 ID:    101 length: 1000 UNIQUE  actual: 5000 %repetitive DNA   -  distance:   100  start: 25209833 end: 25210832
 45 ID:    102 length: 1000 UNIQUE  actual: 5000 %repetitive DNA   -  distance:   200  start: 25209933 end: 25210932
 46 ID:    103 length: 1000 UNIQUE  actual: 5000 %repetitive DNA   -  distance:   300  start: 25210033 end: 25211032
 47 ID:    104 length: 1000 UNIQUE  actual: 5000 %repetitive DNA   -  distance:   400  start: 25210133 end: 25211132
 48 ID:    114 length: 1000 UNIQUE  actual: 5000 %repetitive DNA   -  distance:  1400  start: 25211133 end: 25212132
 49 ID:    115 length: 1000 UNIQUE  actual: 5000 %repetitive DNA   -  distance:  1500  start: 25211233 end: 25212232
 50 ID:    116 length: 1000 UNIQUE  actual: 5000 %repetitive DNA   -  distance:  1600  start: 25211333 end: 25212332
 51 ID:    117 length: 1000 UNIQUE  actual: 5000 %repetitive DNA   -  distance:  1700  start: 25211433 end: 25212432
 52 ID:    118 length: 1000 UNIQUE  actual: 5000 %repetitive DNA   -  distance:  1800  start: 25211533 end: 25212532
 53 ID:    119 length: 1000 UNIQUE  actual: 5000 %repetitive DNA   -  distance:  1900  start: 25211633 end: 25212632
 54 ID:    120 length: 1000 UNIQUE  actual: 5000 %repetitive DNA   -  distance:  2000  start: 25211733 end: 25212732
 55 ID:    121 length:  950 UNIQUE  actual: 4750 %repetitive DNA   -  distance:     0  start: 25209733 end: 25210682
 56 ID:    122 length:  950 UNIQUE  actual: 4750 %repetitive DNA   -  distance:    95  start: 25209828 end: 25210777
 57 ID:    123 length:  950 UNIQUE  actual: 4750 %repetitive DNA   -  distance:   190  start: 25209923 end: 25210872
 58 ID:    124 length:  950 UNIQUE  actual: 4750 %repetitive DNA   -  distance:   285  start: 25210018 end: 25210967
 59 ID:    125 length:  950 UNIQUE  actual: 4750 %repetitive DNA   -  distance:   380  start: 25210113 end: 25211062
 60 ID:    126 length:  950 UNIQUE  actual: 4750 %repetitive DNA   -  distance:   475  start: 25210208 end: 25211157
 61 ID:    136 length:  950 UNIQUE  actual: 4750 %repetitive DNA   -  distance:  1425  start: 25211158 end: 25212107
 62 ID:    137 length:  950 UNIQUE  actual: 4750 %repetitive DNA   -  distance:  1520  start: 25211253 end: 25212202
 63 ID:    138 length:  950 UNIQUE  actual: 4750 %repetitive DNA   -  distance:  1615  start: 25211348 end: 25212297
 64 ID:    139 length:  950 UNIQUE  actual: 4750 %repetitive DNA   -  distance:  1710  start: 25211443 end: 25212392
 65 ID:    140 length:  950 UNIQUE  actual: 4750 %repetitive DNA   -  distance:  1805  start: 25211538 end: 25212487
 66 ID:    141 length:  950 UNIQUE  actual: 4750 %repetitive DNA   -  distance:  1900  start: 25211633 end: 25212582
 67 ID:    142 length:  950 UNIQUE  actual: 4750 %repetitive DNA   -  distance:  1995  start: 25211728 end: 25212677
 68 ID:    143 length:  900 UNIQUE  actual: 4500 %repetitive DNA   -  distance:     0  start: 25209733 end: 25210632
 69 ID:    144 length:  900 UNIQUE  actual: 4500 %repetitive DNA   -  distance:    90  start: 25209823 end: 25210722
 70 ID:    145 length:  900 UNIQUE  actual: 4500 %repetitive DNA   -  distance:   180  start: 25209913 end: 25210812
 71 ID:    146 length:  900 UNIQUE  actual: 4500 %repetitive DNA   -  distance:   270  start: 25210003 end: 25210902
 72 ID:    147 length:  900 UNIQUE  actual: 4500 %repetitive DNA   -  distance:   360  start: 25210093 end: 25210992
 73 ID:    148 length:  900 UNIQUE  actual: 4500 %repetitive DNA   -  distance:   450  start: 25210183 end: 25211082
 74 ID:    149 length:  900 UNIQUE  actual: 4500 %repetitive DNA   -  distance:   540  start: 25210273 end: 25211172
 75 ID:    159 length:  900 UNIQUE  actual: 4500 %repetitive DNA   -  distance:  1440  start: 25211173 end: 25212072
 76 ID:    160 length:  900 UNIQUE  actual: 4500 %repetitive DNA   -  distance:  1530  start: 25211263 end: 25212162
 77 ID:    161 length:  900 UNIQUE  actual: 4500 %repetitive DNA   -  distance:  1620  start: 25211353 end: 25212252
 78 ID:    162 length:  900 UNIQUE  actual: 4500 %repetitive DNA   -  distance:  1710  start: 25211443 end: 25212342
 79 ID:    163 length:  900 UNIQUE  actual: 4500 %repetitive DNA   -  distance:  1800  start: 25211533 end: 25212432
 80 ID:    164 length:  900 UNIQUE  actual: 4500 %repetitive DNA   -  distance:  1890  start: 25211623 end: 25212522
 81 ID:    165 length:  900 UNIQUE  actual: 4500 %repetitive DNA   -  distance:  1980  start: 25211713 end: 25212612
 82 ID:    166 length:  900 UNIQUE  actual: 4500 %repetitive DNA   -  distance:  2070  start: 25211803 end: 25212702
 83 ID:    167 length:  850 UNIQUE  actual: 4250 %repetitive DNA   -  distance:     0  start: 25209733 end: 25210582
 84 ID:    168 length:  850 UNIQUE  actual: 4250 %repetitive DNA   -  distance:    85  start: 25209818 end: 25210667
 85 ID:    169 length:  850 UNIQUE  actual: 4250 %repetitive DNA   -  distance:   170  start: 25209903 end: 25210752
 86 ID:    170 length:  850 UNIQUE  actual: 4250 %repetitive DNA   -  distance:   255  start: 25209988 end: 25210837
 87 ID:    171 length:  850 UNIQUE  actual: 4250 %repetitive DNA   -  distance:   340  start: 25210073 end: 25210922
 88 ID:    172 length:  850 UNIQUE  actual: 4250 %repetitive DNA   -  distance:   425  start: 25210158 end: 25211007
 89 ID:    173 length:  850 UNIQUE  actual: 4250 %repetitive DNA   -  distance:   510  start: 25210243 end: 25211092
 90 ID:    184 length:  850 UNIQUE  actual: 4250 %repetitive DNA   -  distance:  1445  start: 25211178 end: 25212027
 91 ID:    185 length:  850 UNIQUE  actual: 4250 %repetitive DNA   -  distance:  1530  start: 25211263 end: 25212112
 92 ID:    186 length:  850 UNIQUE  actual: 4250 %repetitive DNA   -  distance:  1615  start: 25211348 end: 25212197
 93 ID:    187 length:  850 UNIQUE  actual: 4250 %repetitive DNA   -  distance:  1700  start: 25211433 end: 25212282
 94 ID:    188 length:  850 UNIQUE  actual: 4250 %repetitive DNA   -  distance:  1785  start: 25211518 end: 25212367
 95 ID:    189 length:  850 UNIQUE  actual: 4250 %repetitive DNA   -  distance:  1870  start: 25211603 end: 25212452
 96 ID:    190 length:  850 UNIQUE  actual: 4250 %repetitive DNA   -  distance:  1955  start: 25211688 end: 25212537
 97 ID:    191 length:  850 UNIQUE  actual: 4250 %repetitive DNA   -  distance:  2040  start: 25211773 end: 25212622
 98 ID:    192 length:  850 UNIQUE  actual: 4250 %repetitive DNA   -  distance:  2125  start: 25211858 end: 25212707
 99 ID:    193 length:  800 UNIQUE  actual: 4000 %repetitive DNA   -  distance:     0  start: 25209733 end: 25210532
100 ID:    194 length:  800 UNIQUE  actual: 4000 %repetitive DNA   -  distance:    80  start: 25209813 end: 25210612
101 ID:    195 length:  800 UNIQUE  actual: 4000 %repetitive DNA   -  distance:   160  start: 25209893 end: 25210692
102 ID:    196 length:  800 UNIQUE  actual: 4000 %repetitive DNA   -  distance:   240  start: 25209973 end: 25210772
103 ID:    197 length:  800 UNIQUE  actual: 4000 %repetitive DNA   -  distance:   320  start: 25210053 end: 25210852
104 ID:    198 length:  800 UNIQUE  actual: 4000 %repetitive DNA   -  distance:   400  start: 25210133 end: 25210932
105 ID:    199 length:  800 UNIQUE  actual: 4000 %repetitive DNA   -  distance:   480  start: 25210213 end: 25211012
106 ID:    200 length:  800 UNIQUE  actual: 4000 %repetitive DNA   -  distance:   560  start: 25210293 end: 25211092
107 ID:    201 length:  800 UNIQUE  actual: 4000 %repetitive DNA   -  distance:   640  start: 25210373 end: 25211172
108 ID:    211 length:  800 UNIQUE  actual: 4000 %repetitive DNA   -  distance:  1440  start: 25211173 end: 25211972
109 ID:    212 length:  800 UNIQUE  actual: 4000 %repetitive DNA   -  distance:  1520  start: 25211253 end: 25212052
110 ID:    213 length:  800 UNIQUE  actual: 4000 %repetitive DNA   -  distance:  1600  start: 25211333 end: 25212132
111 ID:    214 length:  800 UNIQUE  actual: 4000 %repetitive DNA   -  distance:  1680  start: 25211413 end: 25212212
112 ID:    215 length:  800 UNIQUE  actual: 4000 %repetitive DNA   -  distance:  1760  start: 25211493 end: 25212292
113 ID:    216 length:  800 UNIQUE  actual: 4000 %repetitive DNA   -  distance:  1840  start: 25211573 end: 25212372
114 ID:    217 length:  800 UNIQUE  actual: 4000 %repetitive DNA   -  distance:  1920  start: 25211653 end: 25212452
115 ID:    218 length:  800 UNIQUE  actual: 4000 %repetitive DNA   -  distance:  2000  start: 25211733 end: 25212532
116 ID:    219 length:  800 UNIQUE  actual: 4000 %repetitive DNA   -  distance:  2080  start: 25211813 end: 25212612
117 ID:    220 length:  800 UNIQUE  actual: 4000 %repetitive DNA   -  distance:  2160  start: 25211893 end: 25212692
118 ID:    221 length:  750 UNIQUE  actual: 3750 %repetitive DNA   -  distance:     0  start: 25209733 end: 25210482
119 ID:    222 length:  750 UNIQUE  actual: 3750 %repetitive DNA   -  distance:    75  start: 25209808 end: 25210557
120 ID:    223 length:  750 UNIQUE  actual: 3750 %repetitive DNA   -  distance:   150  start: 25209883 end: 25210632
121 ID:    224 length:  750 UNIQUE  actual: 3750 %repetitive DNA   -  distance:   225  start: 25209958 end: 25210707
122 ID:    225 length:  750 UNIQUE  actual: 3750 %repetitive DNA   -  distance:   300  start: 25210033 end: 25210782
123 ID:    226 length:  750 UNIQUE  actual: 3750 %repetitive DNA   -  distance:   375  start: 25210108 end: 25210857
124 ID:    227 length:  750 UNIQUE  actual: 3750 %repetitive DNA   -  distance:   450  start: 25210183 end: 25210932
125 ID:    228 length:  750 UNIQUE  actual: 3750 %repetitive DNA   -  distance:   525  start: 25210258 end: 25211007
126 ID:    229 length:  750 UNIQUE  actual: 3750 %repetitive DNA   -  distance:   600  start: 25210333 end: 25211082
127 ID:    230 length:  750 UNIQUE  actual: 3750 %repetitive DNA   -  distance:   675  start: 25210408 end: 25211157
128 ID:    240 length:  750 UNIQUE  actual: 3750 %repetitive DNA   -  distance:  1425  start: 25211158 end: 25211907
129 ID:    241 length:  750 UNIQUE  actual: 3750 %repetitive DNA   -  distance:  1500  start: 25211233 end: 25211982
130 ID:    242 length:  750 UNIQUE  actual: 3750 %repetitive DNA   -  distance:  1575  start: 25211308 end: 25212057
131 ID:    243 length:  750 UNIQUE  actual: 3750 %repetitive DNA   -  distance:  1650  start: 25211383 end: 25212132
132 ID:    244 length:  750 UNIQUE  actual: 3750 %repetitive DNA   -  distance:  1725  start: 25211458 end: 25212207
133 ID:    245 length:  750 UNIQUE  actual: 3750 %repetitive DNA   -  distance:  1800  start: 25211533 end: 25212282
134 ID:    246 length:  750 UNIQUE  actual: 3750 %repetitive DNA   -  distance:  1875  start: 25211608 end: 25212357
135 ID:    247 length:  750 UNIQUE  actual: 3750 %repetitive DNA   -  distance:  1950  start: 25211683 end: 25212432
136 ID:    248 length:  750 UNIQUE  actual: 3750 %repetitive DNA   -  distance:  2025  start: 25211758 end: 25212507
137 ID:    249 length:  750 UNIQUE  actual: 3750 %repetitive DNA   -  distance:  2100  start: 25211833 end: 25212582
138 ID:    250 length:  750 UNIQUE  actual: 3750 %repetitive DNA   -  distance:  2175  start: 25211908 end: 25212657
139 ID:    251 length:  750 UNIQUE  actual: 3750 %repetitive DNA   -  distance:  2250  start: 25211983 end: 25212732
140 ID:    252 length:  700 UNIQUE  actual: 3500 %repetitive DNA   -  distance:     0  start: 25209733 end: 25210432
141 ID:    253 length:  700 UNIQUE  actual: 3500 %repetitive DNA   -  distance:    70  start: 25209803 end: 25210502
142 ID:    254 length:  700 UNIQUE  actual: 3500 %repetitive DNA   -  distance:   140  start: 25209873 end: 25210572
143 ID:    255 length:  700 UNIQUE  actual: 3500 %repetitive DNA   -  distance:   210  start: 25209943 end: 25210642
144 ID:    256 length:  700 UNIQUE  actual: 3500 %repetitive DNA   -  distance:   280  start: 25210013 end: 25210712
145 ID:    257 length:  700 UNIQUE  actual: 3500 %repetitive DNA   -  distance:   350  start: 25210083 end: 25210782
146 ID:    258 length:  700 UNIQUE  actual: 3500 %repetitive DNA   -  distance:   420  start: 25210153 end: 25210852
147 ID:    259 length:  700 UNIQUE  actual: 3500 %repetitive DNA   -  distance:   490  start: 25210223 end: 25210922
148 ID:    260 length:  700 UNIQUE  actual: 3500 %repetitive DNA   -  distance:   560  start: 25210293 end: 25210992
149 ID:    261 length:  700 UNIQUE  actual: 3500 %repetitive DNA   -  distance:   630  start: 25210363 end: 25211062
150 ID:    262 length:  700 UNIQUE  actual: 3500 %repetitive DNA   -  distance:   700  start: 25210433 end: 25211132
151 ID:    272 length:  700 UNIQUE  actual: 3500 %repetitive DNA   -  distance:  1400  start: 25211133 end: 25211832
152 ID:    273 length:  700 UNIQUE  actual: 3500 %repetitive DNA   -  distance:  1470  start: 25211203 end: 25211902
153 ID:    274 length:  700 UNIQUE  actual: 3500 %repetitive DNA   -  distance:  1540  start: 25211273 end: 25211972
154 ID:    275 length:  700 UNIQUE  actual: 3500 %repetitive DNA   -  distance:  1610  start: 25211343 end: 25212042
155 ID:    276 length:  700 UNIQUE  actual: 3500 %repetitive DNA   -  distance:  1680  start: 25211413 end: 25212112
156 ID:    277 length:  700 UNIQUE  actual: 3500 %repetitive DNA   -  distance:  1750  start: 25211483 end: 25212182
157 ID:    278 length:  700 UNIQUE  actual: 3500 %repetitive DNA   -  distance:  1820  start: 25211553 end: 25212252
158 ID:    279 length:  700 UNIQUE  actual: 3500 %repetitive DNA   -  distance:  1890  start: 25211623 end: 25212322
159 ID:    280 length:  700 UNIQUE  actual: 3500 %repetitive DNA   -  distance:  1960  start: 25211693 end: 25212392
160 ID:    281 length:  700 UNIQUE  actual: 3500 %repetitive DNA   -  distance:  2030  start: 25211763 end: 25212462
161 ID:    282 length:  700 UNIQUE  actual: 3500 %repetitive DNA   -  distance:  2100  start: 25211833 end: 25212532
162 ID:    283 length:  700 UNIQUE  actual: 3500 %repetitive DNA   -  distance:  2170  start: 25211903 end: 25212602
163 ID:    284 length:  700 UNIQUE  actual: 3500 %repetitive DNA   -  distance:  2240  start: 25211973 end: 25212672
164 ID:    285 length:  650 UNIQUE  actual: 3250 %repetitive DNA   -  distance:     0  start: 25209733 end: 25210382
165 ID:    286 length:  650 UNIQUE  actual: 3250 %repetitive DNA   -  distance:    65  start: 25209798 end: 25210447
166 ID:    287 length:  650 UNIQUE  actual: 3250 %repetitive DNA   -  distance:   130  start: 25209863 end: 25210512
167 ID:    288 length:  650 UNIQUE  actual: 3250 %repetitive DNA   -  distance:   195  start: 25209928 end: 25210577
168 ID:    289 length:  650 UNIQUE  actual: 3250 %repetitive DNA   -  distance:   260  start: 25209993 end: 25210642
169 ID:    290 length:  650 UNIQUE  actual: 3250 %repetitive DNA   -  distance:   325  start: 25210058 end: 25210707
170 ID:    291 length:  650 UNIQUE  actual: 3250 %repetitive DNA   -  distance:   390  start: 25210123 end: 25210772
171 ID:    292 length:  650 UNIQUE  actual: 3250 %repetitive DNA   -  distance:   455  start: 25210188 end: 25210837
172 ID:    293 length:  650 UNIQUE  actual: 3250 %repetitive DNA   -  distance:   520  start: 25210253 end: 25210902
173 ID:    294 length:  650 UNIQUE  actual: 3250 %repetitive DNA   -  distance:   585  start: 25210318 end: 25210967
174 ID:    295 length:  650 UNIQUE  actual: 3250 %repetitive DNA   -  distance:   650  start: 25210383 end: 25211032
175 ID:    296 length:  650 UNIQUE  actual: 3250 %repetitive DNA   -  distance:   715  start: 25210448 end: 25211097
176 ID:    297 length:  650 UNIQUE  actual: 3250 %repetitive DNA   -  distance:   780  start: 25210513 end: 25211162
177 ID:    307 length:  650 UNIQUE  actual: 3250 %repetitive DNA   -  distance:  1430  start: 25211163 end: 25211812
178 ID:    308 length:  650 UNIQUE  actual: 3250 %repetitive DNA   -  distance:  1495  start: 25211228 end: 25211877
179 ID:    309 length:  650 UNIQUE  actual: 3250 %repetitive DNA   -  distance:  1560  start: 25211293 end: 25211942
180 ID:    310 length:  650 UNIQUE  actual: 3250 %repetitive DNA   -  distance:  1625  start: 25211358 end: 25212007
181 ID:    311 length:  650 UNIQUE  actual: 3250 %repetitive DNA   -  distance:  1690  start: 25211423 end: 25212072
182 ID:    312 length:  650 UNIQUE  actual: 3250 %repetitive DNA   -  distance:  1755  start: 25211488 end: 25212137
183 ID:    313 length:  650 UNIQUE  actual: 3250 %repetitive DNA   -  distance:  1820  start: 25211553 end: 25212202
184 ID:    314 length:  650 UNIQUE  actual: 3250 %repetitive DNA   -  distance:  1885  start: 25211618 end: 25212267
185 ID:    315 length:  650 UNIQUE  actual: 3250 %repetitive DNA   -  distance:  1950  start: 25211683 end: 25212332
186 ID:    316 length:  650 UNIQUE  actual: 3250 %repetitive DNA   -  distance:  2015  start: 25211748 end: 25212397
187 ID:    317 length:  650 UNIQUE  actual: 3250 %repetitive DNA   -  distance:  2080  start: 25211813 end: 25212462
188 ID:    318 length:  650 UNIQUE  actual: 3250 %repetitive DNA   -  distance:  2145  start: 25211878 end: 25212527
189 ID:    319 length:  650 UNIQUE  actual: 3250 %repetitive DNA   -  distance:  2210  start: 25211943 end: 25212592
190 ID:    320 length:  650 UNIQUE  actual: 3250 %repetitive DNA   -  distance:  2275  start: 25212008 end: 25212657
191 ID:    321 length:  650 UNIQUE  actual: 3250 %repetitive DNA   -  distance:  2340  start: 25212073 end: 25212722
192 ID:    322 length:  600 UNIQUE  actual: 3000 %repetitive DNA   -  distance:     0  start: 25209733 end: 25210332
193 ID:    323 length:  600 UNIQUE  actual: 3000 %repetitive DNA   -  distance:    60  start: 25209793 end: 25210392
194 ID:    324 length:  600 UNIQUE  actual: 3000 %repetitive DNA   -  distance:   120  start: 25209853 end: 25210452
195 ID:    325 length:  600 UNIQUE  actual: 3000 %repetitive DNA   -  distance:   180  start: 25209913 end: 25210512
196 ID:    326 length:  600 UNIQUE  actual: 3000 %repetitive DNA   -  distance:   240  start: 25209973 end: 25210572
197 ID:    327 length:  600 UNIQUE  actual: 3000 %repetitive DNA   -  distance:   300  start: 25210033 end: 25210632
198 ID:    328 length:  600 UNIQUE  actual: 3000 %repetitive DNA   -  distance:   360  start: 25210093 end: 25210692
199 ID:    329 length:  600 UNIQUE  actual: 3000 %repetitive DNA   -  distance:   420  start: 25210153 end: 25210752
200 ID:    330 length:  600 UNIQUE  actual: 3000 %repetitive DNA   -  distance:   480  start: 25210213 end: 25210812
201 ID:    331 length:  600 UNIQUE  actual: 3000 %repetitive DNA   -  distance:   540  start: 25210273 end: 25210872
202 ID:    332 length:  600 UNIQUE  actual: 3000 %repetitive DNA   -  distance:   600  start: 25210333 end: 25210932
203 ID:    333 length:  600 UNIQUE  actual: 3000 %repetitive DNA   -  distance:   660  start: 25210393 end: 25210992
204 ID:    334 length:  600 UNIQUE  actual: 3000 %repetitive DNA   -  distance:   720  start: 25210453 end: 25211052
205 ID:    335 length:  600 UNIQUE  actual: 3000 %repetitive DNA   -  distance:   780  start: 25210513 end: 25211112
206 ID:    336 length:  600 UNIQUE  actual: 3000 %repetitive DNA   -  distance:   840  start: 25210573 end: 25211172
207 ID:    346 length:  600 UNIQUE  actual: 3000 %repetitive DNA   -  distance:  1440  start: 25211173 end: 25211772
208 ID:    347 length:  600 UNIQUE  actual: 3000 %repetitive DNA   -  distance:  1500  start: 25211233 end: 25211832
209 ID:    348 length:  600 UNIQUE  actual: 3000 %repetitive DNA   -  distance:  1560  start: 25211293 end: 25211892
210 ID:    349 length:  600 UNIQUE  actual: 3000 %repetitive DNA   -  distance:  1620  start: 25211353 end: 25211952
211 ID:    350 length:  600 UNIQUE  actual: 3000 %repetitive DNA   -  distance:  1680  start: 25211413 end: 25212012
212 ID:    351 length:  600 UNIQUE  actual: 3000 %repetitive DNA   -  distance:  1740  start: 25211473 end: 25212072
213 ID:    352 length:  600 UNIQUE  actual: 3000 %repetitive DNA   -  distance:  1800  start: 25211533 end: 25212132
214 ID:    353 length:  600 UNIQUE  actual: 3000 %repetitive DNA   -  distance:  1860  start: 25211593 end: 25212192
215 ID:    354 length:  600 UNIQUE  actual: 3000 %repetitive DNA   -  distance:  1920  start: 25211653 end: 25212252
216 ID:    355 length:  600 UNIQUE  actual: 3000 %repetitive DNA   -  distance:  1980  start: 25211713 end: 25212312
217 ID:    356 length:  600 UNIQUE  actual: 3000 %repetitive DNA   -  distance:  2040  start: 25211773 end: 25212372
218 ID:    357 length:  600 UNIQUE  actual: 3000 %repetitive DNA   -  distance:  2100  start: 25211833 end: 25212432
219 ID:    358 length:  600 UNIQUE  actual: 3000 %repetitive DNA   -  distance:  2160  start: 25211893 end: 25212492
220 ID:    359 length:  600 UNIQUE  actual: 3000 %repetitive DNA   -  distance:  2220  start: 25211953 end: 25212552
221 ID:    360 length:  600 UNIQUE  actual: 3000 %repetitive DNA   -  distance:  2280  start: 25212013 end: 25212612
222 ID:    361 length:  600 UNIQUE  actual: 3000 %repetitive DNA   -  distance:  2340  start: 25212073 end: 25212672
223 ID:    362 length:  600 UNIQUE  actual: 3000 %repetitive DNA   -  distance:  2400  start: 25212133 end: 25212732
224 ID:    363 length:  550 UNIQUE  actual: 2750 %repetitive DNA   -  distance:     0  start: 25209733 end: 25210282
225 ID:    364 length:  550 UNIQUE  actual: 2750 %repetitive DNA   -  distance:    55  start: 25209788 end: 25210337
226 ID:    365 length:  550 UNIQUE  actual: 2750 %repetitive DNA   -  distance:   110  start: 25209843 end: 25210392
227 ID:    366 length:  550 UNIQUE  actual: 2750 %repetitive DNA   -  distance:   165  start: 25209898 end: 25210447
228 ID:    367 length:  550 UNIQUE  actual: 2750 %repetitive DNA   -  distance:   220  start: 25209953 end: 25210502
229 ID:    368 length:  550 UNIQUE  actual: 2750 %repetitive DNA   -  distance:   275  start: 25210008 end: 25210557
230 ID:    369 length:  550 UNIQUE  actual: 2750 %repetitive DNA   -  distance:   330  start: 25210063 end: 25210612
231 ID:    370 length:  550 UNIQUE  actual: 2750 %repetitive DNA   -  distance:   385  start: 25210118 end: 25210667
232 ID:    371 length:  550 UNIQUE  actual: 2750 %repetitive DNA   -  distance:   440  start: 25210173 end: 25210722
233 ID:    372 length:  550 UNIQUE  actual: 2750 %repetitive DNA   -  distance:   495  start: 25210228 end: 25210777
234 ID:    373 length:  550 UNIQUE  actual: 2750 %repetitive DNA   -  distance:   550  start: 25210283 end: 25210832
235 ID:    374 length:  550 UNIQUE  actual: 2750 %repetitive DNA   -  distance:   605  start: 25210338 end: 25210887
236 ID:    375 length:  550 UNIQUE  actual: 2750 %repetitive DNA   -  distance:   660  start: 25210393 end: 25210942
237 ID:    376 length:  550 UNIQUE  actual: 2750 %repetitive DNA   -  distance:   715  start: 25210448 end: 25210997
238 ID:    377 length:  550 UNIQUE  actual: 2750 %repetitive DNA   -  distance:   770  start: 25210503 end: 25211052
239 ID:    378 length:  550 UNIQUE  actual: 2750 %repetitive DNA   -  distance:   825  start: 25210558 end: 25211107
240 ID:    379 length:  550 UNIQUE  actual: 2750 %repetitive DNA   -  distance:   880  start: 25210613 end: 25211162
241 ID:    389 length:  550 UNIQUE  actual: 2750 %repetitive DNA   -  distance:  1430  start: 25211163 end: 25211712
242 ID:    390 length:  550 UNIQUE  actual: 2750 %repetitive DNA   -  distance:  1485  start: 25211218 end: 25211767
243 ID:    391 length:  550 UNIQUE  actual: 2750 %repetitive DNA   -  distance:  1540  start: 25211273 end: 25211822
244 ID:    392 length:  550 UNIQUE  actual: 2750 %repetitive DNA   -  distance:  1595  start: 25211328 end: 25211877
245 ID:    393 length:  550 UNIQUE  actual: 2750 %repetitive DNA   -  distance:  1650  start: 25211383 end: 25211932
246 ID:    394 length:  550 UNIQUE  actual: 2750 %repetitive DNA   -  distance:  1705  start: 25211438 end: 25211987
247 ID:    395 length:  550 UNIQUE  actual: 2750 %repetitive DNA   -  distance:  1760  start: 25211493 end: 25212042
248 ID:    396 length:  550 UNIQUE  actual: 2750 %repetitive DNA   -  distance:  1815  start: 25211548 end: 25212097
249 ID:    397 length:  550 UNIQUE  actual: 2750 %repetitive DNA   -  distance:  1870  start: 25211603 end: 25212152
250 ID:    398 length:  550 UNIQUE  actual: 2750 %repetitive DNA   -  distance:  1925  start: 25211658 end: 25212207
251 ID:    399 length:  550 UNIQUE  actual: 2750 %repetitive DNA   -  distance:  1980  start: 25211713 end: 25212262
252 ID:    400 length:  550 UNIQUE  actual: 2750 %repetitive DNA   -  distance:  2035  start: 25211768 end: 25212317
253 ID:    401 length:  550 UNIQUE  actual: 2750 %repetitive DNA   -  distance:  2090  start: 25211823 end: 25212372
254 ID:    402 length:  550 UNIQUE  actual: 2750 %repetitive DNA   -  distance:  2145  start: 25211878 end: 25212427
255 ID:    403 length:  550 UNIQUE  actual: 2750 %repetitive DNA   -  distance:  2200  start: 25211933 end: 25212482
256 ID:    404 length:  550 UNIQUE  actual: 2750 %repetitive DNA   -  distance:  2255  start: 25211988 end: 25212537
257 ID:    405 length:  550 UNIQUE  actual: 2750 %repetitive DNA   -  distance:  2310  start: 25212043 end: 25212592
258 ID:    406 length:  550 UNIQUE  actual: 2750 %repetitive DNA   -  distance:  2365  start: 25212098 end: 25212647
259 ID:    407 length:  550 UNIQUE  actual: 2750 %repetitive DNA   -  distance:  2420  start: 25212153 end: 25212702
260 ID:    408 length:  500 UNIQUE  actual: 2500 %repetitive DNA   -  distance:     0  start: 25209733 end: 25210232
261 ID:    409 length:  500 UNIQUE  actual: 2500 %repetitive DNA   -  distance:    50  start: 25209783 end: 25210282
262 ID:    410 length:  500 UNIQUE  actual: 2500 %repetitive DNA   -  distance:   100  start: 25209833 end: 25210332
263 ID:    411 length:  500 UNIQUE  actual: 2500 %repetitive DNA   -  distance:   150  start: 25209883 end: 25210382
264 ID:    412 length:  500 UNIQUE  actual: 2500 %repetitive DNA   -  distance:   200  start: 25209933 end: 25210432
265 ID:    413 length:  500 UNIQUE  actual: 2500 %repetitive DNA   -  distance:   250  start: 25209983 end: 25210482
266 ID:    414 length:  500 UNIQUE  actual: 2500 %repetitive DNA   -  distance:   300  start: 25210033 end: 25210532
267 ID:    415 length:  500 UNIQUE  actual: 2500 %repetitive DNA   -  distance:   350  start: 25210083 end: 25210582
268 ID:    416 length:  500 UNIQUE  actual: 2500 %repetitive DNA   -  distance:   400  start: 25210133 end: 25210632
269 ID:    417 length:  500 UNIQUE  actual: 2500 %repetitive DNA   -  distance:   450  start: 25210183 end: 25210682
270 ID:    418 length:  500 UNIQUE  actual: 2500 %repetitive DNA   -  distance:   500  start: 25210233 end: 25210732
271 ID:    419 length:  500 UNIQUE  actual: 2500 %repetitive DNA   -  distance:   550  start: 25210283 end: 25210782
272 ID:    420 length:  500 UNIQUE  actual: 2500 %repetitive DNA   -  distance:   600  start: 25210333 end: 25210832
273 ID:    421 length:  500 UNIQUE  actual: 2500 %repetitive DNA   -  distance:   650  start: 25210383 end: 25210882
274 ID:    422 length:  500 UNIQUE  actual: 2500 %repetitive DNA   -  distance:   700  start: 25210433 end: 25210932
275 ID:    423 length:  500 UNIQUE  actual: 2500 %repetitive DNA   -  distance:   750  start: 25210483 end: 25210982
276 ID:    424 length:  500 UNIQUE  actual: 2500 %repetitive DNA   -  distance:   800  start: 25210533 end: 25211032
277 ID:    425 length:  500 UNIQUE  actual: 2500 %repetitive DNA   -  distance:   850  start: 25210583 end: 25211082
278 ID:    426 length:  500 UNIQUE  actual: 2500 %repetitive DNA   -  distance:   900  start: 25210633 end: 25211132
279 ID:    436 length:  500 UNIQUE  actual: 2500 %repetitive DNA   -  distance:  1400  start: 25211133 end: 25211632
280 ID:    437 length:  500 UNIQUE  actual: 2500 %repetitive DNA   -  distance:  1450  start: 25211183 end: 25211682
281 ID:    438 length:  500 UNIQUE  actual: 2500 %repetitive DNA   -  distance:  1500  start: 25211233 end: 25211732
282 ID:    439 length:  500 UNIQUE  actual: 2500 %repetitive DNA   -  distance:  1550  start: 25211283 end: 25211782
283 ID:    440 length:  500 UNIQUE  actual: 2500 %repetitive DNA   -  distance:  1600  start: 25211333 end: 25211832
284 ID:    441 length:  500 UNIQUE  actual: 2500 %repetitive DNA   -  distance:  1650  start: 25211383 end: 25211882
285 ID:    442 length:  500 UNIQUE  actual: 2500 %repetitive DNA   -  distance:  1700  start: 25211433 end: 25211932
286 ID:    443 length:  500 UNIQUE  actual: 2500 %repetitive DNA   -  distance:  1750  start: 25211483 end: 25211982
287 ID:    444 length:  500 UNIQUE  actual: 2500 %repetitive DNA   -  distance:  1800  start: 25211533 end: 25212032
288 ID:    445 length:  500 UNIQUE  actual: 2500 %repetitive DNA   -  distance:  1850  start: 25211583 end: 25212082
289 ID:    446 length:  500 UNIQUE  actual: 2500 %repetitive DNA   -  distance:  1900  start: 25211633 end: 25212132
290 ID:    447 length:  500 UNIQUE  actual: 2500 %repetitive DNA   -  distance:  1950  start: 25211683 end: 25212182
291 ID:    448 length:  500 UNIQUE  actual: 2500 %repetitive DNA   -  distance:  2000  start: 25211733 end: 25212232
292 ID:    449 length:  500 UNIQUE  actual: 2500 %repetitive DNA   -  distance:  2050  start: 25211783 end: 25212282
293 ID:    450 length:  500 UNIQUE  actual: 2500 %repetitive DNA   -  distance:  2100  start: 25211833 end: 25212332
294 ID:    451 length:  500 UNIQUE  actual: 2500 %repetitive DNA   -  distance:  2150  start: 25211883 end: 25212382
295 ID:    452 length:  500 UNIQUE  actual: 2500 %repetitive DNA   -  distance:  2200  start: 25211933 end: 25212432
296 ID:    453 length:  500 UNIQUE  actual: 2500 %repetitive DNA   -  distance:  2250  start: 25211983 end: 25212482
297 ID:    454 length:  500 UNIQUE  actual: 2500 %repetitive DNA   -  distance:  2300  start: 25212033 end: 25212532
298 ID:    455 length:  500 UNIQUE  actual: 2500 %repetitive DNA   -  distance:  2350  start: 25212083 end: 25212582
299 ID:    456 length:  500 UNIQUE  actual: 2500 %repetitive DNA   -  distance:  2400  start: 25212133 end: 25212632
300 ID:    457 length:  500 UNIQUE  actual: 2500 %repetitive DNA   -  distance:  2450  start: 25212183 end: 25212682
301 ID:    458 length:  500 UNIQUE  actual: 2500 %repetitive DNA   -  distance:  2500  start: 25212233 end: 25212732

PUTATIVE PROBES
---------------

Ranked by overall score = score_ratio - 2 * repetitive DNA content

  1 ID:      3 length: 1300 score ratio: 42.2 actual: 6500 %repetitive DNA   -  distance:   260  start: 25209993 end: 25211292 overall:  42.2
  2 ID:      4 length: 1300 score ratio: 42.2 actual: 6500 %repetitive DNA   -  distance:   390  start: 25210123 end: 25211422 overall:  42.2
  3 ID:      5 length: 1300 score ratio: 42.2 actual: 6500 %repetitive DNA   -  distance:   520  start: 25210253 end: 25211552 overall:  42.2
  4 ID:      6 length: 1300 score ratio: 42.2 actual: 6500 %repetitive DNA   -  distance:   650  start: 25210383 end: 25211682 overall:  42.2
  5 ID:      7 length: 1300 score ratio: 42.2 actual: 6500 %repetitive DNA   -  distance:   780  start: 25210513 end: 25211812 overall:  42.2
  6 ID:      8 length: 1300 score ratio: 42.2 actual: 6500 %repetitive DNA   -  distance:   910  start: 25210643 end: 25211942 overall:  42.2
  7 ID:      9 length: 1300 score ratio: 42.2 actual: 6500 %repetitive DNA   -  distance:  1040  start: 25210773 end: 25212072 overall:  42.2
  8 ID:     10 length: 1300 score ratio: 42.2 actual: 6500 %repetitive DNA   -  distance:  1170  start: 25210903 end: 25212202 overall:  42.2
  9 ID:     11 length: 1300 score ratio: 42.2 actual: 6500 %repetitive DNA   -  distance:  1300  start: 25211033 end: 25212332 overall:  42.2
 10 ID:     17 length: 1250 score ratio: 40.6 actual: 6250 %repetitive DNA   -  distance:   250  start: 25209983 end: 25211232 overall:  40.6
 11 ID:     18 length: 1250 score ratio: 40.6 actual: 6250 %repetitive DNA   -  distance:   375  start: 25210108 end: 25211357 overall:  40.6
 12 ID:     19 length: 1250 score ratio: 40.6 actual: 6250 %repetitive DNA   -  distance:   500  start: 25210233 end: 25211482 overall:  40.6
 13 ID:     20 length: 1250 score ratio: 40.6 actual: 6250 %repetitive DNA   -  distance:   625  start: 25210358 end: 25211607 overall:  40.6
 14 ID:     21 length: 1250 score ratio: 40.6 actual: 6250 %repetitive DNA   -  distance:   750  start: 25210483 end: 25211732 overall:  40.6
 15 ID:     22 length: 1250 score ratio: 40.6 actual: 6250 %repetitive DNA   -  distance:   875  start: 25210608 end: 25211857 overall:  40.6
 16 ID:     23 length: 1250 score ratio: 40.6 actual: 6250 %repetitive DNA   -  distance:  1000  start: 25210733 end: 25211982 overall:  40.6
 17 ID:     24 length: 1250 score ratio: 40.6 actual: 6250 %repetitive DNA   -  distance:  1125  start: 25210858 end: 25212107 overall:  40.6
 18 ID:     25 length: 1250 score ratio: 40.6 actual: 6250 %repetitive DNA   -  distance:  1250  start: 25210983 end: 25212232 overall:  40.6
 19 ID:     26 length: 1250 score ratio: 40.6 actual: 6250 %repetitive DNA   -  distance:  1375  start: 25211108 end: 25212357 overall:  40.6
 20 ID:     33 length: 1200 score ratio: 39.0 actual: 6000 %repetitive DNA   -  distance:   360  start: 25210093 end: 25211292 overall:  39.0
 21 ID:     34 length: 1200 score ratio: 39.0 actual: 6000 %repetitive DNA   -  distance:   480  start: 25210213 end: 25211412 overall:  39.0
 22 ID:     35 length: 1200 score ratio: 39.0 actual: 6000 %repetitive DNA   -  distance:   600  start: 25210333 end: 25211532 overall:  39.0
 23 ID:     36 length: 1200 score ratio: 39.0 actual: 6000 %repetitive DNA   -  distance:   720  start: 25210453 end: 25211652 overall:  39.0
 24 ID:     37 length: 1200 score ratio: 39.0 actual: 6000 %repetitive DNA   -  distance:   840  start: 25210573 end: 25211772 overall:  39.0
 25 ID:     38 length: 1200 score ratio: 39.0 actual: 6000 %repetitive DNA   -  distance:   960  start: 25210693 end: 25211892 overall:  39.0
 26 ID:     39 length: 1200 score ratio: 39.0 actual: 6000 %repetitive DNA   -  distance:  1080  start: 25210813 end: 25212012 overall:  39.0
 27 ID:     40 length: 1200 score ratio: 39.0 actual: 6000 %repetitive DNA   -  distance:  1200  start: 25210933 end: 25212132 overall:  39.0
 28 ID:     41 length: 1200 score ratio: 39.0 actual: 6000 %repetitive DNA   -  distance:  1320  start: 25211053 end: 25212252 overall:  39.0
 29 ID:     49 length: 1150 score ratio: 37.3 actual: 5750 %repetitive DNA   -  distance:   345  start: 25210078 end: 25211227 overall:  37.3
 30 ID:     50 length: 1150 score ratio: 37.3 actual: 5750 %repetitive DNA   -  distance:   460  start: 25210193 end: 25211342 overall:  37.3
 31 ID:     51 length: 1150 score ratio: 37.3 actual: 5750 %repetitive DNA   -  distance:   575  start: 25210308 end: 25211457 overall:  37.3
 32 ID:     52 length: 1150 score ratio: 37.3 actual: 5750 %repetitive DNA   -  distance:   690  start: 25210423 end: 25211572 overall:  37.3
 33 ID:     53 length: 1150 score ratio: 37.3 actual: 5750 %repetitive DNA   -  distance:   805  start: 25210538 end: 25211687 overall:  37.3
 34 ID:     54 length: 1150 score ratio: 37.3 actual: 5750 %repetitive DNA   -  distance:   920  start: 25210653 end: 25211802 overall:  37.3
 35 ID:     55 length: 1150 score ratio: 37.3 actual: 5750 %repetitive DNA   -  distance:  1035  start: 25210768 end: 25211917 overall:  37.3
 36 ID:     56 length: 1150 score ratio: 37.3 actual: 5750 %repetitive DNA   -  distance:  1150  start: 25210883 end: 25212032 overall:  37.3
 37 ID:     57 length: 1150 score ratio: 37.3 actual: 5750 %repetitive DNA   -  distance:  1265  start: 25210998 end: 25212147 overall:  37.3
 38 ID:     58 length: 1150 score ratio: 37.3 actual: 5750 %repetitive DNA   -  distance:  1380  start: 25211113 end: 25212262 overall:  37.3
 39 ID:     67 length: 1100 score ratio: 35.7 actual: 5500 %repetitive DNA   -  distance:   440  start: 25210173 end: 25211272 overall:  35.7
 40 ID:     68 length: 1100 score ratio: 35.7 actual: 5500 %repetitive DNA   -  distance:   550  start: 25210283 end: 25211382 overall:  35.7
 41 ID:     69 length: 1100 score ratio: 35.7 actual: 5500 %repetitive DNA   -  distance:   660  start: 25210393 end: 25211492 overall:  35.7
 42 ID:     70 length: 1100 score ratio: 35.7 actual: 5500 %repetitive DNA   -  distance:   770  start: 25210503 end: 25211602 overall:  35.7
 43 ID:     71 length: 1100 score ratio: 35.7 actual: 5500 %repetitive DNA   -  distance:   880  start: 25210613 end: 25211712 overall:  35.7
 44 ID:     72 length: 1100 score ratio: 35.7 actual: 5500 %repetitive DNA   -  distance:   990  start: 25210723 end: 25211822 overall:  35.7
 45 ID:     73 length: 1100 score ratio: 35.7 actual: 5500 %repetitive DNA   -  distance:  1100  start: 25210833 end: 25211932 overall:  35.7
 46 ID:     74 length: 1100 score ratio: 35.7 actual: 5500 %repetitive DNA   -  distance:  1210  start: 25210943 end: 25212042 overall:  35.7
 47 ID:     75 length: 1100 score ratio: 35.7 actual: 5500 %repetitive DNA   -  distance:  1320  start: 25211053 end: 25212152 overall:  35.7
 48 ID:     85 length: 1050 score ratio: 34.1 actual: 5250 %repetitive DNA   -  distance:   420  start: 25210153 end: 25211202 overall:  34.1
 49 ID:     86 length: 1050 score ratio: 34.1 actual: 5250 %repetitive DNA   -  distance:   525  start: 25210258 end: 25211307 overall:  34.1
 50 ID:     87 length: 1050 score ratio: 34.1 actual: 5250 %repetitive DNA   -  distance:   630  start: 25210363 end: 25211412 overall:  34.1
 51 ID:     88 length: 1050 score ratio: 34.1 actual: 5250 %repetitive DNA   -  distance:   735  start: 25210468 end: 25211517 overall:  34.1
 52 ID:     89 length: 1050 score ratio: 34.1 actual: 5250 %repetitive DNA   -  distance:   840  start: 25210573 end: 25211622 overall:  34.1
 53 ID:     90 length: 1050 score ratio: 34.1 actual: 5250 %repetitive DNA   -  distance:   945  start: 25210678 end: 25211727 overall:  34.1
 54 ID:     91 length: 1050 score ratio: 34.1 actual: 5250 %repetitive DNA   -  distance:  1050  start: 25210783 end: 25211832 overall:  34.1
 55 ID:     92 length: 1050 score ratio: 34.1 actual: 5250 %repetitive DNA   -  distance:  1155  start: 25210888 end: 25211937 overall:  34.1
 56 ID:     93 length: 1050 score ratio: 34.1 actual: 5250 %repetitive DNA   -  distance:  1260  start: 25210993 end: 25212042 overall:  34.1
 57 ID:     94 length: 1050 score ratio: 34.1 actual: 5250 %repetitive DNA   -  distance:  1365  start: 25211098 end: 25212147 overall:  34.1
 58 ID:    105 length: 1000 score ratio: 32.5 actual: 5000 %repetitive DNA   -  distance:   500  start: 25210233 end: 25211232 overall:  32.5
 59 ID:    106 length: 1000 score ratio: 32.5 actual: 5000 %repetitive DNA   -  distance:   600  start: 25210333 end: 25211332 overall:  32.5
 60 ID:    107 length: 1000 score ratio: 32.5 actual: 5000 %repetitive DNA   -  distance:   700  start: 25210433 end: 25211432 overall:  32.5
 61 ID:    108 length: 1000 score ratio: 32.5 actual: 5000 %repetitive DNA   -  distance:   800  start: 25210533 end: 25211532 overall:  32.5
 62 ID:    109 length: 1000 score ratio: 32.5 actual: 5000 %repetitive DNA   -  distance:   900  start: 25210633 end: 25211632 overall:  32.5
 63 ID:    110 length: 1000 score ratio: 32.5 actual: 5000 %repetitive DNA   -  distance:  1000  start: 25210733 end: 25211732 overall:  32.5
 64 ID:    111 length: 1000 score ratio: 32.5 actual: 5000 %repetitive DNA   -  distance:  1100  start: 25210833 end: 25211832 overall:  32.5
 65 ID:    112 length: 1000 score ratio: 32.5 actual: 5000 %repetitive DNA   -  distance:  1200  start: 25210933 end: 25211932 overall:  32.5
 66 ID:    113 length: 1000 score ratio: 32.5 actual: 5000 %repetitive DNA   -  distance:  1300  start: 25211033 end: 25212032 overall:  32.5
 67 ID:    127 length:  950 score ratio: 30.8 actual: 4750 %repetitive DNA   -  distance:   570  start: 25210303 end: 25211252 overall:  30.8
 68 ID:    128 length:  950 score ratio: 30.8 actual: 4750 %repetitive DNA   -  distance:   665  start: 25210398 end: 25211347 overall:  30.8
 69 ID:    129 length:  950 score ratio: 30.8 actual: 4750 %repetitive DNA   -  distance:   760  start: 25210493 end: 25211442 overall:  30.8
 70 ID:    130 length:  950 score ratio: 30.8 actual: 4750 %repetitive DNA   -  distance:   855  start: 25210588 end: 25211537 overall:  30.8
 71 ID:    131 length:  950 score ratio: 30.8 actual: 4750 %repetitive DNA   -  distance:   950  start: 25210683 end: 25211632 overall:  30.8
 72 ID:    132 length:  950 score ratio: 30.8 actual: 4750 %repetitive DNA   -  distance:  1045  start: 25210778 end: 25211727 overall:  30.8
 73 ID:    133 length:  950 score ratio: 30.8 actual: 4750 %repetitive DNA   -  distance:  1140  start: 25210873 end: 25211822 overall:  30.8
 74 ID:    134 length:  950 score ratio: 30.8 actual: 4750 %repetitive DNA   -  distance:  1235  start: 25210968 end: 25211917 overall:  30.8
 75 ID:    135 length:  950 score ratio: 30.8 actual: 4750 %repetitive DNA   -  distance:  1330  start: 25211063 end: 25212012 overall:  30.8
 76 ID:    150 length:  900 score ratio: 29.2 actual: 4500 %repetitive DNA   -  distance:   630  start: 25210363 end: 25211262 overall:  29.2
 77 ID:    151 length:  900 score ratio: 29.2 actual: 4500 %repetitive DNA   -  distance:   720  start: 25210453 end: 25211352 overall:  29.2
 78 ID:    152 length:  900 score ratio: 29.2 actual: 4500 %repetitive DNA   -  distance:   810  start: 25210543 end: 25211442 overall:  29.2
 79 ID:    153 length:  900 score ratio: 29.2 actual: 4500 %repetitive DNA   -  distance:   900  start: 25210633 end: 25211532 overall:  29.2
 80 ID:    154 length:  900 score ratio: 29.2 actual: 4500 %repetitive DNA   -  distance:   990  start: 25210723 end: 25211622 overall:  29.2
 81 ID:    155 length:  900 score ratio: 29.2 actual: 4500 %repetitive DNA   -  distance:  1080  start: 25210813 end: 25211712 overall:  29.2
 82 ID:    156 length:  900 score ratio: 29.2 actual: 4500 %repetitive DNA   -  distance:  1170  start: 25210903 end: 25211802 overall:  29.2
 83 ID:    157 length:  900 score ratio: 29.2 actual: 4500 %repetitive DNA   -  distance:  1260  start: 25210993 end: 25211892 overall:  29.2
 84 ID:    158 length:  900 score ratio: 29.2 actual: 4500 %repetitive DNA   -  distance:  1350  start: 25211083 end: 25211982 overall:  29.2
 85 ID:    174 length:  850 score ratio: 27.6 actual: 4250 %repetitive DNA   -  distance:   595  start: 25210328 end: 25211177 overall:  27.6
 86 ID:    175 length:  850 score ratio: 27.6 actual: 4250 %repetitive DNA   -  distance:   680  start: 25210413 end: 25211262 overall:  27.6
 87 ID:    176 length:  850 score ratio: 27.6 actual: 4250 %repetitive DNA   -  distance:   765  start: 25210498 end: 25211347 overall:  27.6
 88 ID:    177 length:  850 score ratio: 27.6 actual: 4250 %repetitive DNA   -  distance:   850  start: 25210583 end: 25211432 overall:  27.6
 89 ID:    178 length:  850 score ratio: 27.6 actual: 4250 %repetitive DNA   -  distance:   935  start: 25210668 end: 25211517 overall:  27.6
 90 ID:    179 length:  850 score ratio: 27.6 actual: 4250 %repetitive DNA   -  distance:  1020  start: 25210753 end: 25211602 overall:  27.6
 91 ID:    180 length:  850 score ratio: 27.6 actual: 4250 %repetitive DNA   -  distance:  1105  start: 25210838 end: 25211687 overall:  27.6
 92 ID:    181 length:  850 score ratio: 27.6 actual: 4250 %repetitive DNA   -  distance:  1190  start: 25210923 end: 25211772 overall:  27.6
 93 ID:    182 length:  850 score ratio: 27.6 actual: 4250 %repetitive DNA   -  distance:  1275  start: 25211008 end: 25211857 overall:  27.6
 94 ID:    183 length:  850 score ratio: 27.6 actual: 4250 %repetitive DNA   -  distance:  1360  start: 25211093 end: 25211942 overall:  27.6
 95 ID:    202 length:  800 score ratio: 26.0 actual: 4000 %repetitive DNA   -  distance:   720  start: 25210453 end: 25211252 overall:  26.0
 96 ID:    203 length:  800 score ratio: 26.0 actual: 4000 %repetitive DNA   -  distance:   800  start: 25210533 end: 25211332 overall:  26.0
 97 ID:    204 length:  800 score ratio: 26.0 actual: 4000 %repetitive DNA   -  distance:   880  start: 25210613 end: 25211412 overall:  26.0
 98 ID:    205 length:  800 score ratio: 26.0 actual: 4000 %repetitive DNA   -  distance:   960  start: 25210693 end: 25211492 overall:  26.0
 99 ID:    206 length:  800 score ratio: 26.0 actual: 4000 %repetitive DNA   -  distance:  1040  start: 25210773 end: 25211572 overall:  26.0
100 ID:    207 length:  800 score ratio: 26.0 actual: 4000 %repetitive DNA   -  distance:  1120  start: 25210853 end: 25211652 overall:  26.0
101 ID:    208 length:  800 score ratio: 26.0 actual: 4000 %repetitive DNA   -  distance:  1200  start: 25210933 end: 25211732 overall:  26.0
102 ID:    209 length:  800 score ratio: 26.0 actual: 4000 %repetitive DNA   -  distance:  1280  start: 25211013 end: 25211812 overall:  26.0
103 ID:    210 length:  800 score ratio: 26.0 actual: 4000 %repetitive DNA   -  distance:  1360  start: 25211093 end: 25211892 overall:  26.0
104 ID:    231 length:  750 score ratio: 24.4 actual: 3750 %repetitive DNA   -  distance:   750  start: 25210483 end: 25211232 overall:  24.4
105 ID:    232 length:  750 score ratio: 24.4 actual: 3750 %repetitive DNA   -  distance:   825  start: 25210558 end: 25211307 overall:  24.4
106 ID:    233 length:  750 score ratio: 24.4 actual: 3750 %repetitive DNA   -  distance:   900  start: 25210633 end: 25211382 overall:  24.4
107 ID:    234 length:  750 score ratio: 24.4 actual: 3750 %repetitive DNA   -  distance:   975  start: 25210708 end: 25211457 overall:  24.4
108 ID:    235 length:  750 score ratio: 24.4 actual: 3750 %repetitive DNA   -  distance:  1050  start: 25210783 end: 25211532 overall:  24.4
109 ID:    236 length:  750 score ratio: 24.4 actual: 3750 %repetitive DNA   -  distance:  1125  start: 25210858 end: 25211607 overall:  24.4
110 ID:    237 length:  750 score ratio: 24.4 actual: 3750 %repetitive DNA   -  distance:  1200  start: 25210933 end: 25211682 overall:  24.4
111 ID:    238 length:  750 score ratio: 24.4 actual: 3750 %repetitive DNA   -  distance:  1275  start: 25211008 end: 25211757 overall:  24.4
112 ID:    239 length:  750 score ratio: 24.4 actual: 3750 %repetitive DNA   -  distance:  1350  start: 25211083 end: 25211832 overall:  24.4
113 ID:    263 length:  700 score ratio: 22.7 actual: 3500 %repetitive DNA   -  distance:   770  start: 25210503 end: 25211202 overall:  22.7
114 ID:    264 length:  700 score ratio: 22.7 actual: 3500 %repetitive DNA   -  distance:   840  start: 25210573 end: 25211272 overall:  22.7
115 ID:    265 length:  700 score ratio: 22.7 actual: 3500 %repetitive DNA   -  distance:   910  start: 25210643 end: 25211342 overall:  22.7
116 ID:    266 length:  700 score ratio: 22.7 actual: 3500 %repetitive DNA   -  distance:   980  start: 25210713 end: 25211412 overall:  22.7
117 ID:    267 length:  700 score ratio: 22.7 actual: 3500 %repetitive DNA   -  distance:  1050  start: 25210783 end: 25211482 overall:  22.7
118 ID:    268 length:  700 score ratio: 22.7 actual: 3500 %repetitive DNA   -  distance:  1120  start: 25210853 end: 25211552 overall:  22.7
119 ID:    269 length:  700 score ratio: 22.7 actual: 3500 %repetitive DNA   -  distance:  1190  start: 25210923 end: 25211622 overall:  22.7
120 ID:    270 length:  700 score ratio: 22.7 actual: 3500 %repetitive DNA   -  distance:  1260  start: 25210993 end: 25211692 overall:  22.7
121 ID:    271 length:  700 score ratio: 22.7 actual: 3500 %repetitive DNA   -  distance:  1330  start: 25211063 end: 25211762 overall:  22.7
122 ID:    298 length:  650 score ratio: 21.1 actual: 3250 %repetitive DNA   -  distance:   845  start: 25210578 end: 25211227 overall:  21.1
123 ID:    299 length:  650 score ratio: 21.1 actual: 3250 %repetitive DNA   -  distance:   910  start: 25210643 end: 25211292 overall:  21.1
124 ID:    300 length:  650 score ratio: 21.1 actual: 3250 %repetitive DNA   -  distance:   975  start: 25210708 end: 25211357 overall:  21.1
125 ID:    301 length:  650 score ratio: 21.1 actual: 3250 %repetitive DNA   -  distance:  1040  start: 25210773 end: 25211422 overall:  21.1
126 ID:    302 length:  650 score ratio: 21.1 actual: 3250 %repetitive DNA   -  distance:  1105  start: 25210838 end: 25211487 overall:  21.1
127 ID:    303 length:  650 score ratio: 21.1 actual: 3250 %repetitive DNA   -  distance:  1170  start: 25210903 end: 25211552 overall:  21.1
128 ID:    304 length:  650 score ratio: 21.1 actual: 3250 %repetitive DNA   -  distance:  1235  start: 25210968 end: 25211617 overall:  21.1
129 ID:    305 length:  650 score ratio: 21.1 actual: 3250 %repetitive DNA   -  distance:  1300  start: 25211033 end: 25211682 overall:  21.1
130 ID:    306 length:  650 score ratio: 21.1 actual: 3250 %repetitive DNA   -  distance:  1365  start: 25211098 end: 25211747 overall:  21.1
131 ID:    337 length:  600 score ratio: 19.5 actual: 3000 %repetitive DNA   -  distance:   900  start: 25210633 end: 25211232 overall:  19.5
132 ID:    338 length:  600 score ratio: 19.5 actual: 3000 %repetitive DNA   -  distance:   960  start: 25210693 end: 25211292 overall:  19.5
133 ID:    339 length:  600 score ratio: 19.5 actual: 3000 %repetitive DNA   -  distance:  1020  start: 25210753 end: 25211352 overall:  19.5
134 ID:    340 length:  600 score ratio: 19.5 actual: 3000 %repetitive DNA   -  distance:  1080  start: 25210813 end: 25211412 overall:  19.5
135 ID:    341 length:  600 score ratio: 19.5 actual: 3000 %repetitive DNA   -  distance:  1140  start: 25210873 end: 25211472 overall:  19.5
136 ID:    342 length:  600 score ratio: 19.5 actual: 3000 %repetitive DNA   -  distance:  1200  start: 25210933 end: 25211532 overall:  19.5
137 ID:    343 length:  600 score ratio: 19.5 actual: 3000 %repetitive DNA   -  distance:  1260  start: 25210993 end: 25211592 overall:  19.5
138 ID:    344 length:  600 score ratio: 19.5 actual: 3000 %repetitive DNA   -  distance:  1320  start: 25211053 end: 25211652 overall:  19.5
139 ID:    345 length:  600 score ratio: 19.5 actual: 3000 %repetitive DNA   -  distance:  1380  start: 25211113 end: 25211712 overall:  19.5
140 ID:    380 length:  550 score ratio: 17.9 actual: 2750 %repetitive DNA   -  distance:   935  start: 25210668 end: 25211217 overall:  17.9
141 ID:    381 length:  550 score ratio: 17.9 actual: 2750 %repetitive DNA   -  distance:   990  start: 25210723 end: 25211272 overall:  17.9
142 ID:    382 length:  550 score ratio: 17.9 actual: 2750 %repetitive DNA   -  distance:  1045  start: 25210778 end: 25211327 overall:  17.9
143 ID:    383 length:  550 score ratio: 17.9 actual: 2750 %repetitive DNA   -  distance:  1100  start: 25210833 end: 25211382 overall:  17.9
144 ID:    384 length:  550 score ratio: 17.9 actual: 2750 %repetitive DNA   -  distance:  1155  start: 25210888 end: 25211437 overall:  17.9
145 ID:    385 length:  550 score ratio: 17.9 actual: 2750 %repetitive DNA   -  distance:  1210  start: 25210943 end: 25211492 overall:  17.9
146 ID:    386 length:  550 score ratio: 17.9 actual: 2750 %repetitive DNA   -  distance:  1265  start: 25210998 end: 25211547 overall:  17.9
147 ID:    387 length:  550 score ratio: 17.9 actual: 2750 %repetitive DNA   -  distance:  1320  start: 25211053 end: 25211602 overall:  17.9
148 ID:    388 length:  550 score ratio: 17.9 actual: 2750 %repetitive DNA   -  distance:  1375  start: 25211108 end: 25211657 overall:  17.9
149 ID:    427 length:  500 score ratio: 16.2 actual: 2500 %repetitive DNA   -  distance:   950  start: 25210683 end: 25211182 overall:  16.2
150 ID:    428 length:  500 score ratio: 16.2 actual: 2500 %repetitive DNA   -  distance:  1000  start: 25210733 end: 25211232 overall:  16.2
151 ID:    429 length:  500 score ratio: 16.2 actual: 2500 %repetitive DNA   -  distance:  1050  start: 25210783 end: 25211282 overall:  16.2
152 ID:    430 length:  500 score ratio: 16.2 actual: 2500 %repetitive DNA   -  distance:  1100  start: 25210833 end: 25211332 overall:  16.2
153 ID:    431 length:  500 score ratio: 16.2 actual: 2500 %repetitive DNA   -  distance:  1150  start: 25210883 end: 25211382 overall:  16.2
154 ID:    432 length:  500 score ratio: 16.2 actual: 2500 %repetitive DNA   -  distance:  1200  start: 25210933 end: 25211432 overall:  16.2
155 ID:    433 length:  500 score ratio: 16.2 actual: 2500 %repetitive DNA   -  distance:  1250  start: 25210983 end: 25211482 overall:  16.2
156 ID:    434 length:  500 score ratio: 16.2 actual: 2500 %repetitive DNA   -  distance:  1300  start: 25211033 end: 25211532 overall:  16.2
157 ID:    435 length:  500 score ratio: 16.2 actual: 2500 %repetitive DNA   -  distance:  1350  start: 25211083 end: 25211582 overall:  16.2

REDUNDANT PROBES
----------------

  - None

UNIQUE PUTATIVE PROBES
----------------------

>1 Get primers
AGGCCAAGAAGTAGACCCACCTGCCAGTGCCCGTAGACCCATGATCCTCTGGCCCTCATT
CAACAGCCGTTGACAGCCCACCTAGAGAAAGGCAGCAGAATATCTCAGTGGAGGCCCCTT
TCACAGAGCGTGGGTCAGGGCTGCTAGCTTCCAGGACACAACAGCAGATAGTGTCTGATG
GCATGAAAGCAGATAGCTACAAGGCTCTTGGACCAGGCTAGCACTGGGTCCTGCACCCAG
GGAGAGCCACCTCACCTTGACAGGGTTGGCAGGAAGGGGCCCTAGAAAGTCAGTAGGATA
CGGGTAGTCCATCATGGCGAGCACAGTAAATGCATTTCGGGCAAACCCAAAGAGCTGAGT
CAGGTCCTTTGGGCTGGAAAGTGATTGACAGGTACCAAAGTTCTGGCTGATGGTGTCATA
GGCTGGGAAGAGAGAGGCCAGGAGAAAAGGCTGAGGAAACTGCTGGCAAATGTGAAGGGC
AAGAATGAATGCCCAAGGTGGGCAGCAGGTGAGGAAAGAGTCCCTCACCTCCCTGGAGGA
ACAAGTCTTTGATTTGCTGAAAGGCATCCCGCACAGCCTGGGCGCACTTGGGACTCTGGC
CATAAAAGTCCTGGAGAAGAGACCAAGGTTGCTGCTGCCATTCTTGCACTGGCCTGGGGT
ACCCAAGTCCCCTCACTCACCGCTGTGACATCTCGGAAGAATTGGTAGGAGTCCCCAAGG
CCTGCAACAGCTACAACAGGAGCGCTGGCTGCCAGTGCCCCAGCCACCAGGTGGGGGTAC
TTCATCCTCATGTAGGCACTCAGCATCCCCCCATAACTGGGAGTACAGAGCACAGATCAT
GGTTGTGGGAAGCTGCCCACAACTCAGGCGAGCAGCCTCACTGTCCTCCAGGCTGAGGTG
CTAGGCTGCTCTTTCCCTGCTCAGAACGCCCAAGGGTGGGAAAGAAGGACCTGAAACTGT
CAGGCCCACACACCCTGATCCCAGGGCCAAGGCAGATACAGCCTTCACTGGGAGAAGGCA
CCTGTGGGTGCCCTGCCCTGACCCAGCAATGAAGACATTGCAGAGACAAAGTCAGAAGGA
ATTGTCCCACTAGTGGGAACAACATAGCATACACTGCCTATGAGGTCCACTCAAGGAGGG
CTTCCAGAAGGAGGTAAAGCTAGACCCCGCCCTTCCACATGTGGGGTAGGCATAGGATGT
TGAGACTGTAAGAGACATCTCTTTGGCCCTCCTTGTATAGGGTGTCAATCGGCACAACAG
GGTGGAGCCTTAGAGTAGGGTAAGATTAGGACTCTAGGTT

>2 Get primers
TGGGTCAGGGCTGCTAGCTTCCAGGACACAACAGCAGATAGTGTCTGATGGCATGAAAGC
AGATAGCTACAAGGCTCTTGGACCAGGCTAGCACTGGGTCCTGCACCCAGGGAGAGCCAC
CTCACCTTGACAGGGTTGGCAGGAAGGGGCCCTAGAAAGTCAGTAGGATACGGGTAGTCC
ATCATGGCGAGCACAGTAAATGCATTTCGGGCAAACCCAAAGAGCTGAGTCAGGTCCTTT
GGGCTGGAAAGTGATTGACAGGTACCAAAGTTCTGGCTGATGGTGTCATAGGCTGGGAAG
AGAGAGGCCAGGAGAAAAGGCTGAGGAAACTGCTGGCAAATGTGAAGGGCAAGAATGAAT
GCCCAAGGTGGGCAGCAGGTGAGGAAAGAGTCCCTCACCTCCCTGGAGGAACAAGTCTTT
GATTTGCTGAAAGGCATCCCGCACAGCCTGGGCGCACTTGGGACTCTGGCCATAAAAGTC
CTGGAGAAGAGACCAAGGTTGCTGCTGCCATTCTTGCACTGGCCTGGGGTACCCAAGTCC
CCTCACTCACCGCTGTGACATCTCGGAAGAATTGGTAGGAGTCCCCAAGGCCTGCAACAG
CTACAACAGGAGCGCTGGCTGCCAGTGCCCCAGCCACCAGGTGGGGGTACTTCATCCTCA
TGTAGGCACTCAGCATCCCCCCATAACTGGGAGTACAGAGCACAGATCATGGTTGTGGGA
AGCTGCCCACAACTCAGGCGAGCAGCCTCACTGTCCTCCAGGCTGAGGTGCTAGGCTGCT
CTTTCCCTGCTCAGAACGCCCAAGGGTGGGAAAGAAGGACCTGAAACTGTCAGGCCCACA
CACCCTGATCCCAGGGCCAAGGCAGATACAGCCTTCACTGGGAGAAGGCACCTGTGGGTG
CCCTGCCCTGACCCAGCAATGAAGACATTGCAGAGACAAAGTCAGAAGGAATTGTCCCAC
TAGTGGGAACAACATAGCATACACTGCCTATGAGGTCCACTCAAGGAGGGCTTCCAGAAG
GAGGTAAAGCTAGACCCCGCCCTTCCACATGTGGGGTAGGCATAGGATGTTGAGACTGTA
AGAGACATCTCTTTGGCCCTCCTTGTATAGGGTGTCAATCGGCACAACAGGGTGGAGCCT
TAGAGTAGGGTAAGATTAGGACTCTAGGTTCTCTCATGGGTCCAGATCTGTCATGAAGGG
AGGTCAAGGACCCACCTCCCTCCAAAGGCTATGGTGGGGGCATCATGGACCCCAAGGTCC
TGCCGCAGGGCCTGGAGCAGCACAGCAAAGTCGGCCAGCG

>12 Get primers
CCTGCTCCACAGTCAGCAGCTGTGTATATCCCCGCTGTGTGGACTGGACACCGAACGGAA
GCGATTTCCCATAGTACCGCTGCAGAAAGCAGGAAGGGATGGCTAATCCACTCCTCGGTG
CTCCCCACCTCCTTCAACTCAGGGACTGCCAGGAACTGTACAGGTACCCACGTGCTCAGC
AAAGACAAGCAGGGCCTCCTGCTGGGCTGCCAGTTCCACCATGAAGCCAGAGTTGTTAGC
GAAGGACCAGATATCCCCCTCATTCCCTGTGTAGAAAAAGATGGGCCCTTCGCCCATCTT
CCAGAACTTATCTGTTGGAAGTAAATGAGTTTCCATAAGGCCAGGGAAACGCAGGTAGGA
ACCCATGCGGTCGAGCCAGCACTCACCTGACACTAGGAACCGCTGGCCAAAGGTTTTGTT
GCCGAAACTCTCAAAGTTGAAATGGTCCATGTATTGCTCAAAATAATTCTCATGAAAGTC
AGGGTCTAGAACTCTGTCGGCTGAGGGCAGGTGCAGAGACTCAGGAGCTGGTTGGGATCA
TCAGGGATCTAGGCGGGTCAGGAGGAAGGGCAGCCAGTCTGTACTCACCTCTGGCCTGGA
GGTTGCACAGTCCCAGTGACAGCAGCAGGACCAGGATCCAGGAGGGGACACCATGGTCCA
CAGGGTAACAAGGATGGAAGTTCATGCTTGATTCTGAGCCGGGCGCTGACTGTCATGTGA
TTTGGTCACATGACCGACACAACGGGCGGGGCAGCATCACGTGATAGTCTGGCGGGGGCT
GTCCTACTGTGGCTGGATTCTAGTTGGAGGATCAGCCTACTCTTCTTCAGTTTCCCGGTT
CCTCCAAATTTCTGGGCTCCTACTTGTTTCCACAGAGATGGATACTGTGGAGGTCCAGGA
AGCAGAGAGATGGCTAAGGCTCATCAGGACCGTATGATCTCCCAAGTGTCCAGCTACTGA
GTACCACAAGGTGATGGGTGGGAGGGTCCTCCCACGGAAGGATACCGCAGTCCCTAGGGG
TTGCAAGCCCCACATGTTCCACTGGCTGCTAGAGCTACCTACTCAATCAGCCCTGGGCAT
CACCATCAGGTACTCGGCCAAAATGACCTCTCTGCTTCCAGTCCTCAGTTCTGGTCAGCA
CCAGACAGGCCCATAATTACAGAGCCAGGGAAACTGGAACATTTGTCTCCCCTTAGACAG
TGGCAGCAGGAAGGTGGGGGGTTGTTGCAGAGGAACAGTGTCTCTGAGAGAGGACCTTGG
ACTTTCTGGGAATCTCTGAGCTGCCCGGTTCTCCCCACTG

>13 Get primers
CCTTCAACTCAGGGACTGCCAGGAACTGTACAGGTACCCACGTGCTCAGCAAAGACAAGC
AGGGCCTCCTGCTGGGCTGCCAGTTCCACCATGAAGCCAGAGTTGTTAGCGAAGGACCAG
ATATCCCCCTCATTCCCTGTGTAGAAAAAGATGGGCCCTTCGCCCATCTTCCAGAACTTA
TCTGTTGGAAGTAAATGAGTTTCCATAAGGCCAGGGAAACGCAGGTAGGAACCCATGCGG
TCGAGCCAGCACTCACCTGACACTAGGAACCGCTGGCCAAAGGTTTTGTTGCCGAAACTC
TCAAAGTTGAAATGGTCCATGTATTGCTCAAAATAATTCTCATGAAAGTCAGGGTCTAGA
ACTCTGTCGGCTGAGGGCAGGTGCAGAGACTCAGGAGCTGGTTGGGATCATCAGGGATCT
AGGCGGGTCAGGAGGAAGGGCAGCCAGTCTGTACTCACCTCTGGCCTGGAGGTTGCACAG
TCCCAGTGACAGCAGCAGGACCAGGATCCAGGAGGGGACACCATGGTCCACAGGGTAACA
AGGATGGAAGTTCATGCTTGATTCTGAGCCGGGCGCTGACTGTCATGTGATTTGGTCACA
TGACCGACACAACGGGCGGGGCAGCATCACGTGATAGTCTGGCGGGGGCTGTCCTACTGT
GGCTGGATTCTAGTTGGAGGATCAGCCTACTCTTCTTCAGTTTCCCGGTTCCTCCAAATT
TCTGGGCTCCTACTTGTTTCCACAGAGATGGATACTGTGGAGGTCCAGGAAGCAGAGAGA
TGGCTAAGGCTCATCAGGACCGTATGATCTCCCAAGTGTCCAGCTACTGAGTACCACAAG
GTGATGGGTGGGAGGGTCCTCCCACGGAAGGATACCGCAGTCCCTAGGGGTTGCAAGCCC
CACATGTTCCACTGGCTGCTAGAGCTACCTACTCAATCAGCCCTGGGCATCACCATCAGG
TACTCGGCCAAAATGACCTCTCTGCTTCCAGTCCTCAGTTCTGGTCAGCACCAGACAGGC
CCATAATTACAGAGCCAGGGAAACTGGAACATTTGTCTCCCCTTAGACAGTGGCAGCAGG
AAGGTGGGGGGTTGTTGCAGAGGAACAGTGTCTCTGAGAGAGGACCTTGGACTTTCTGGG
AATCTCTGAGCTGCCCGGTTCTCCCCACTGCTGGCACTGTGCCCACAGCCCAAACAGAAT
GGGGGAGATGGAGGGGCAGGGCTTCTGTGGGAAGCTGCCCTCCACCTCATTGGCACAGAG
TGTCTCATTGCAGAGAGAAAAAAGGACCAGTTTTCTCTCT

>14 Get primers
CATTCCCTGTGTAGAAAAAGATGGGCCCTTCGCCCATCTTCCAGAACTTATCTGTTGGAA
GTAAATGAGTTTCCATAAGGCCAGGGAAACGCAGGTAGGAACCCATGCGGTCGAGCCAGC
ACTCACCTGACACTAGGAACCGCTGGCCAAAGGTTTTGTTGCCGAAACTCTCAAAGTTGA
AATGGTCCATGTATTGCTCAAAATAATTCTCATGAAAGTCAGGGTCTAGAACTCTGTCGG
CTGAGGGCAGGTGCAGAGACTCAGGAGCTGGTTGGGATCATCAGGGATCTAGGCGGGTCA
GGAGGAAGGGCAGCCAGTCTGTACTCACCTCTGGCCTGGAGGTTGCACAGTCCCAGTGAC
AGCAGCAGGACCAGGATCCAGGAGGGGACACCATGGTCCACAGGGTAACAAGGATGGAAG
TTCATGCTTGATTCTGAGCCGGGCGCTGACTGTCATGTGATTTGGTCACATGACCGACAC
AACGGGCGGGGCAGCATCACGTGATAGTCTGGCGGGGGCTGTCCTACTGTGGCTGGATTC
TAGTTGGAGGATCAGCCTACTCTTCTTCAGTTTCCCGGTTCCTCCAAATTTCTGGGCTCC
TACTTGTTTCCACAGAGATGGATACTGTGGAGGTCCAGGAAGCAGAGAGATGGCTAAGGC
TCATCAGGACCGTATGATCTCCCAAGTGTCCAGCTACTGAGTACCACAAGGTGATGGGTG
GGAGGGTCCTCCCACGGAAGGATACCGCAGTCCCTAGGGGTTGCAAGCCCCACATGTTCC
ACTGGCTGCTAGAGCTACCTACTCAATCAGCCCTGGGCATCACCATCAGGTACTCGGCCA
AAATGACCTCTCTGCTTCCAGTCCTCAGTTCTGGTCAGCACCAGACAGGCCCATAATTAC
AGAGCCAGGGAAACTGGAACATTTGTCTCCCCTTAGACAGTGGCAGCAGGAAGGTGGGGG
GTTGTTGCAGAGGAACAGTGTCTCTGAGAGAGGACCTTGGACTTTCTGGGAATCTCTGAG
CTGCCCGGTTCTCCCCACTGCTGGCACTGTGCCCACAGCCCAAACAGAATGGGGGAGATG
GAGGGGCAGGGCTTCTGTGGGAAGCTGCCCTCCACCTCATTGGCACAGAGTGTCTCATTG
CAGAGAGAAAAAAGGACCAGTTTTCTCTCTGGCACCCAGGTCTGGAAGAGGAGTGACATC
CACGGAAGTTGGTGACTTGGACTGGCTGGCCGTGAGTGGAACATGTCCATCCAGCATGGC
CACAGTCCAGTGGGACACACAGCCTAGAGCTGTGGAATGC

>15 Get primers
AGGCCAAGAAGTAGACCCACCTGCCAGTGCCCGTAGACCCATGATCCTCTGGCCCTCATT
CAACAGCCGTTGACAGCCCACCTAGAGAAAGGCAGCAGAATATCTCAGTGGAGGCCCCTT
TCACAGAGCGTGGGTCAGGGCTGCTAGCTTCCAGGACACAACAGCAGATAGTGTCTGATG
GCATGAAAGCAGATAGCTACAAGGCTCTTGGACCAGGCTAGCACTGGGTCCTGCACCCAG
GGAGAGCCACCTCACCTTGACAGGGTTGGCAGGAAGGGGCCCTAGAAAGTCAGTAGGATA
CGGGTAGTCCATCATGGCGAGCACAGTAAATGCATTTCGGGCAAACCCAAAGAGCTGAGT
CAGGTCCTTTGGGCTGGAAAGTGATTGACAGGTACCAAAGTTCTGGCTGATGGTGTCATA
GGCTGGGAAGAGAGAGGCCAGGAGAAAAGGCTGAGGAAACTGCTGGCAAATGTGAAGGGC
AAGAATGAATGCCCAAGGTGGGCAGCAGGTGAGGAAAGAGTCCCTCACCTCCCTGGAGGA
ACAAGTCTTTGATTTGCTGAAAGGCATCCCGCACAGCCTGGGCGCACTTGGGACTCTGGC
CATAAAAGTCCTGGAGAAGAGACCAAGGTTGCTGCTGCCATTCTTGCACTGGCCTGGGGT
ACCCAAGTCCCCTCACTCACCGCTGTGACATCTCGGAAGAATTGGTAGGAGTCCCCAAGG
CCTGCAACAGCTACAACAGGAGCGCTGGCTGCCAGTGCCCCAGCCACCAGGTGGGGGTAC
TTCATCCTCATGTAGGCACTCAGCATCCCCCCATAACTGGGAGTACAGAGCACAGATCAT
GGTTGTGGGAAGCTGCCCACAACTCAGGCGAGCAGCCTCACTGTCCTCCAGGCTGAGGTG
CTAGGCTGCTCTTTCCCTGCTCAGAACGCCCAAGGGTGGGAAAGAAGGACCTGAAACTGT
CAGGCCCACACACCCTGATCCCAGGGCCAAGGCAGATACAGCCTTCACTGGGAGAAGGCA
CCTGTGGGTGCCCTGCCCTGACCCAGCAATGAAGACATTGCAGAGACAAAGTCAGAAGGA
ATTGTCCCACTAGTGGGAACAACATAGCATACACTGCCTATGAGGTCCACTCAAGGAGGG
CTTCCAGAAGGAGGTAAAGCTAGACCCCGCCCTTCCACATGTGGGGTAGGCATAGGATGT
TGAGACTGTAAGAGACATCTCTTTGGCCCTCCTTGTATAGGGTGTCAATC

>16 Get primers
GAGCGTGGGTCAGGGCTGCTAGCTTCCAGGACACAACAGCAGATAGTGTCTGATGGCATG
AAAGCAGATAGCTACAAGGCTCTTGGACCAGGCTAGCACTGGGTCCTGCACCCAGGGAGA
GCCACCTCACCTTGACAGGGTTGGCAGGAAGGGGCCCTAGAAAGTCAGTAGGATACGGGT
AGTCCATCATGGCGAGCACAGTAAATGCATTTCGGGCAAACCCAAAGAGCTGAGTCAGGT
CCTTTGGGCTGGAAAGTGATTGACAGGTACCAAAGTTCTGGCTGATGGTGTCATAGGCTG
GGAAGAGAGAGGCCAGGAGAAAAGGCTGAGGAAACTGCTGGCAAATGTGAAGGGCAAGAA
TGAATGCCCAAGGTGGGCAGCAGGTGAGGAAAGAGTCCCTCACCTCCCTGGAGGAACAAG
TCTTTGATTTGCTGAAAGGCATCCCGCACAGCCTGGGCGCACTTGGGACTCTGGCCATAA
AAGTCCTGGAGAAGAGACCAAGGTTGCTGCTGCCATTCTTGCACTGGCCTGGGGTACCCA
AGTCCCCTCACTCACCGCTGTGACATCTCGGAAGAATTGGTAGGAGTCCCCAAGGCCTGC
AACAGCTACAACAGGAGCGCTGGCTGCCAGTGCCCCAGCCACCAGGTGGGGGTACTTCAT
CCTCATGTAGGCACTCAGCATCCCCCCATAACTGGGAGTACAGAGCACAGATCATGGTTG
TGGGAAGCTGCCCACAACTCAGGCGAGCAGCCTCACTGTCCTCCAGGCTGAGGTGCTAGG
CTGCTCTTTCCCTGCTCAGAACGCCCAAGGGTGGGAAAGAAGGACCTGAAACTGTCAGGC
CCACACACCCTGATCCCAGGGCCAAGGCAGATACAGCCTTCACTGGGAGAAGGCACCTGT
GGGTGCCCTGCCCTGACCCAGCAATGAAGACATTGCAGAGACAAAGTCAGAAGGAATTGT
CCCACTAGTGGGAACAACATAGCATACACTGCCTATGAGGTCCACTCAAGGAGGGCTTCC
AGAAGGAGGTAAAGCTAGACCCCGCCCTTCCACATGTGGGGTAGGCATAGGATGTTGAGA
CTGTAAGAGACATCTCTTTGGCCCTCCTTGTATAGGGTGTCAATCGGCACAACAGGGTGG
AGCCTTAGAGTAGGGTAAGATTAGGACTCTAGGTTCTCTCATGGGTCCAGATCTGTCATG
AAGGGAGGTCAAGGACCCACCTCCCTCCAAAGGCTATGGTGGGGGCATCA

>27 Get primers
ATAGTACCGCTGCAGAAAGCAGGAAGGGATGGCTAATCCACTCCTCGGTGCTCCCCACCT
CCTTCAACTCAGGGACTGCCAGGAACTGTACAGGTACCCACGTGCTCAGCAAAGACAAGC
AGGGCCTCCTGCTGGGCTGCCAGTTCCACCATGAAGCCAGAGTTGTTAGCGAAGGACCAG
ATATCCCCCTCATTCCCTGTGTAGAAAAAGATGGGCCCTTCGCCCATCTTCCAGAACTTA
TCTGTTGGAAGTAAATGAGTTTCCATAAGGCCAGGGAAACGCAGGTAGGAACCCATGCGG
TCGAGCCAGCACTCACCTGACACTAGGAACCGCTGGCCAAAGGTTTTGTTGCCGAAACTC
TCAAAGTTGAAATGGTCCATGTATTGCTCAAAATAATTCTCATGAAAGTCAGGGTCTAGA
ACTCTGTCGGCTGAGGGCAGGTGCAGAGACTCAGGAGCTGGTTGGGATCATCAGGGATCT
AGGCGGGTCAGGAGGAAGGGCAGCCAGTCTGTACTCACCTCTGGCCTGGAGGTTGCACAG
TCCCAGTGACAGCAGCAGGACCAGGATCCAGGAGGGGACACCATGGTCCACAGGGTAACA
AGGATGGAAGTTCATGCTTGATTCTGAGCCGGGCGCTGACTGTCATGTGATTTGGTCACA
TGACCGACACAACGGGCGGGGCAGCATCACGTGATAGTCTGGCGGGGGCTGTCCTACTGT
GGCTGGATTCTAGTTGGAGGATCAGCCTACTCTTCTTCAGTTTCCCGGTTCCTCCAAATT
TCTGGGCTCCTACTTGTTTCCACAGAGATGGATACTGTGGAGGTCCAGGAAGCAGAGAGA
TGGCTAAGGCTCATCAGGACCGTATGATCTCCCAAGTGTCCAGCTACTGAGTACCACAAG
GTGATGGGTGGGAGGGTCCTCCCACGGAAGGATACCGCAGTCCCTAGGGGTTGCAAGCCC
CACATGTTCCACTGGCTGCTAGAGCTACCTACTCAATCAGCCCTGGGCATCACCATCAGG
TACTCGGCCAAAATGACCTCTCTGCTTCCAGTCCTCAGTTCTGGTCAGCACCAGACAGGC
CCATAATTACAGAGCCAGGGAAACTGGAACATTTGTCTCCCCTTAGACAGTGGCAGCAGG
AAGGTGGGGGGTTGTTGCAGAGGAACAGTGTCTCTGAGAGAGGACCTTGGACTTTCTGGG
AATCTCTGAGCTGCCCGGTTCTCCCCACTGCTGGCACTGTGCCCACAGCC

>28 Get primers
CTCCTGCTGGGCTGCCAGTTCCACCATGAAGCCAGAGTTGTTAGCGAAGGACCAGATATC
CCCCTCATTCCCTGTGTAGAAAAAGATGGGCCCTTCGCCCATCTTCCAGAACTTATCTGT
TGGAAGTAAATGAGTTTCCATAAGGCCAGGGAAACGCAGGTAGGAACCCATGCGGTCGAG
CCAGCACTCACCTGACACTAGGAACCGCTGGCCAAAGGTTTTGTTGCCGAAACTCTCAAA
GTTGAAATGGTCCATGTATTGCTCAAAATAATTCTCATGAAAGTCAGGGTCTAGAACTCT
GTCGGCTGAGGGCAGGTGCAGAGACTCAGGAGCTGGTTGGGATCATCAGGGATCTAGGCG
GGTCAGGAGGAAGGGCAGCCAGTCTGTACTCACCTCTGGCCTGGAGGTTGCACAGTCCCA
GTGACAGCAGCAGGACCAGGATCCAGGAGGGGACACCATGGTCCACAGGGTAACAAGGAT
GGAAGTTCATGCTTGATTCTGAGCCGGGCGCTGACTGTCATGTGATTTGGTCACATGACC
GACACAACGGGCGGGGCAGCATCACGTGATAGTCTGGCGGGGGCTGTCCTACTGTGGCTG
GATTCTAGTTGGAGGATCAGCCTACTCTTCTTCAGTTTCCCGGTTCCTCCAAATTTCTGG
GCTCCTACTTGTTTCCACAGAGATGGATACTGTGGAGGTCCAGGAAGCAGAGAGATGGCT
AAGGCTCATCAGGACCGTATGATCTCCCAAGTGTCCAGCTACTGAGTACCACAAGGTGAT
GGGTGGGAGGGTCCTCCCACGGAAGGATACCGCAGTCCCTAGGGGTTGCAAGCCCCACAT
GTTCCACTGGCTGCTAGAGCTACCTACTCAATCAGCCCTGGGCATCACCATCAGGTACTC
GGCCAAAATGACCTCTCTGCTTCCAGTCCTCAGTTCTGGTCAGCACCAGACAGGCCCATA
ATTACAGAGCCAGGGAAACTGGAACATTTGTCTCCCCTTAGACAGTGGCAGCAGGAAGGT
GGGGGGTTGTTGCAGAGGAACAGTGTCTCTGAGAGAGGACCTTGGACTTTCTGGGAATCT
CTGAGCTGCCCGGTTCTCCCCACTGCTGGCACTGTGCCCACAGCCCAAACAGAATGGGGG
AGATGGAGGGGCAGGGCTTCTGTGGGAAGCTGCCCTCCACCTCATTGGCACAGAGTGTCT
CATTGCAGAGAGAAAAAAGGACCAGTTTTCTCTCTGGCACCCAGGTCTGG

>29 Get primers
GTAAATGAGTTTCCATAAGGCCAGGGAAACGCAGGTAGGAACCCATGCGGTCGAGCCAGC
ACTCACCTGACACTAGGAACCGCTGGCCAAAGGTTTTGTTGCCGAAACTCTCAAAGTTGA
AATGGTCCATGTATTGCTCAAAATAATTCTCATGAAAGTCAGGGTCTAGAACTCTGTCGG
CTGAGGGCAGGTGCAGAGACTCAGGAGCTGGTTGGGATCATCAGGGATCTAGGCGGGTCA
GGAGGAAGGGCAGCCAGTCTGTACTCACCTCTGGCCTGGAGGTTGCACAGTCCCAGTGAC
AGCAGCAGGACCAGGATCCAGGAGGGGACACCATGGTCCACAGGGTAACAAGGATGGAAG
TTCATGCTTGATTCTGAGCCGGGCGCTGACTGTCATGTGATTTGGTCACATGACCGACAC
AACGGGCGGGGCAGCATCACGTGATAGTCTGGCGGGGGCTGTCCTACTGTGGCTGGATTC
TAGTTGGAGGATCAGCCTACTCTTCTTCAGTTTCCCGGTTCCTCCAAATTTCTGGGCTCC
TACTTGTTTCCACAGAGATGGATACTGTGGAGGTCCAGGAAGCAGAGAGATGGCTAAGGC
TCATCAGGACCGTATGATCTCCCAAGTGTCCAGCTACTGAGTACCACAAGGTGATGGGTG
GGAGGGTCCTCCCACGGAAGGATACCGCAGTCCCTAGGGGTTGCAAGCCCCACATGTTCC
ACTGGCTGCTAGAGCTACCTACTCAATCAGCCCTGGGCATCACCATCAGGTACTCGGCCA
AAATGACCTCTCTGCTTCCAGTCCTCAGTTCTGGTCAGCACCAGACAGGCCCATAATTAC
AGAGCCAGGGAAACTGGAACATTTGTCTCCCCTTAGACAGTGGCAGCAGGAAGGTGGGGG
GTTGTTGCAGAGGAACAGTGTCTCTGAGAGAGGACCTTGGACTTTCTGGGAATCTCTGAG
CTGCCCGGTTCTCCCCACTGCTGGCACTGTGCCCACAGCCCAAACAGAATGGGGGAGATG
GAGGGGCAGGGCTTCTGTGGGAAGCTGCCCTCCACCTCATTGGCACAGAGTGTCTCATTG
CAGAGAGAAAAAAGGACCAGTTTTCTCTCTGGCACCCAGGTCTGGAAGAGGAGTGACATC
CACGGAAGTTGGTGACTTGGACTGGCTGGCCGTGAGTGGAACATGTCCATCCAGCATGGC
CACAGTCCAGTGGGACACACAGCCTAGAGCTGTGGAATGCCGTGCCACAG

>30 Get primers
AGGCCAAGAAGTAGACCCACCTGCCAGTGCCCGTAGACCCATGATCCTCTGGCCCTCATT
CAACAGCCGTTGACAGCCCACCTAGAGAAAGGCAGCAGAATATCTCAGTGGAGGCCCCTT
TCACAGAGCGTGGGTCAGGGCTGCTAGCTTCCAGGACACAACAGCAGATAGTGTCTGATG
GCATGAAAGCAGATAGCTACAAGGCTCTTGGACCAGGCTAGCACTGGGTCCTGCACCCAG
GGAGAGCCACCTCACCTTGACAGGGTTGGCAGGAAGGGGCCCTAGAAAGTCAGTAGGATA
CGGGTAGTCCATCATGGCGAGCACAGTAAATGCATTTCGGGCAAACCCAAAGAGCTGAGT
CAGGTCCTTTGGGCTGGAAAGTGATTGACAGGTACCAAAGTTCTGGCTGATGGTGTCATA
GGCTGGGAAGAGAGAGGCCAGGAGAAAAGGCTGAGGAAACTGCTGGCAAATGTGAAGGGC
AAGAATGAATGCCCAAGGTGGGCAGCAGGTGAGGAAAGAGTCCCTCACCTCCCTGGAGGA
ACAAGTCTTTGATTTGCTGAAAGGCATCCCGCACAGCCTGGGCGCACTTGGGACTCTGGC
CATAAAAGTCCTGGAGAAGAGACCAAGGTTGCTGCTGCCATTCTTGCACTGGCCTGGGGT
ACCCAAGTCCCCTCACTCACCGCTGTGACATCTCGGAAGAATTGGTAGGAGTCCCCAAGG
CCTGCAACAGCTACAACAGGAGCGCTGGCTGCCAGTGCCCCAGCCACCAGGTGGGGGTAC
TTCATCCTCATGTAGGCACTCAGCATCCCCCCATAACTGGGAGTACAGAGCACAGATCAT
GGTTGTGGGAAGCTGCCCACAACTCAGGCGAGCAGCCTCACTGTCCTCCAGGCTGAGGTG
CTAGGCTGCTCTTTCCCTGCTCAGAACGCCCAAGGGTGGGAAAGAAGGACCTGAAACTGT
CAGGCCCACACACCCTGATCCCAGGGCCAAGGCAGATACAGCCTTCACTGGGAGAAGGCA
CCTGTGGGTGCCCTGCCCTGACCCAGCAATGAAGACATTGCAGAGACAAAGTCAGAAGGA
ATTGTCCCACTAGTGGGAACAACATAGCATACACTGCCTATGAGGTCCACTCAAGGAGGG
CTTCCAGAAGGAGGTAAAGCTAGACCCCGCCCTTCCACATGTGGGGTAGGCATAGGATGT

>31 Get primers
TCACAGAGCGTGGGTCAGGGCTGCTAGCTTCCAGGACACAACAGCAGATAGTGTCTGATG
GCATGAAAGCAGATAGCTACAAGGCTCTTGGACCAGGCTAGCACTGGGTCCTGCACCCAG
GGAGAGCCACCTCACCTTGACAGGGTTGGCAGGAAGGGGCCCTAGAAAGTCAGTAGGATA
CGGGTAGTCCATCATGGCGAGCACAGTAAATGCATTTCGGGCAAACCCAAAGAGCTGAGT
CAGGTCCTTTGGGCTGGAAAGTGATTGACAGGTACCAAAGTTCTGGCTGATGGTGTCATA
GGCTGGGAAGAGAGAGGCCAGGAGAAAAGGCTGAGGAAACTGCTGGCAAATGTGAAGGGC
AAGAATGAATGCCCAAGGTGGGCAGCAGGTGAGGAAAGAGTCCCTCACCTCCCTGGAGGA
ACAAGTCTTTGATTTGCTGAAAGGCATCCCGCACAGCCTGGGCGCACTTGGGACTCTGGC
CATAAAAGTCCTGGAGAAGAGACCAAGGTTGCTGCTGCCATTCTTGCACTGGCCTGGGGT
ACCCAAGTCCCCTCACTCACCGCTGTGACATCTCGGAAGAATTGGTAGGAGTCCCCAAGG
CCTGCAACAGCTACAACAGGAGCGCTGGCTGCCAGTGCCCCAGCCACCAGGTGGGGGTAC
TTCATCCTCATGTAGGCACTCAGCATCCCCCCATAACTGGGAGTACAGAGCACAGATCAT
GGTTGTGGGAAGCTGCCCACAACTCAGGCGAGCAGCCTCACTGTCCTCCAGGCTGAGGTG
CTAGGCTGCTCTTTCCCTGCTCAGAACGCCCAAGGGTGGGAAAGAAGGACCTGAAACTGT
CAGGCCCACACACCCTGATCCCAGGGCCAAGGCAGATACAGCCTTCACTGGGAGAAGGCA
CCTGTGGGTGCCCTGCCCTGACCCAGCAATGAAGACATTGCAGAGACAAAGTCAGAAGGA
ATTGTCCCACTAGTGGGAACAACATAGCATACACTGCCTATGAGGTCCACTCAAGGAGGG
CTTCCAGAAGGAGGTAAAGCTAGACCCCGCCCTTCCACATGTGGGGTAGGCATAGGATGT
TGAGACTGTAAGAGACATCTCTTTGGCCCTCCTTGTATAGGGTGTCAATCGGCACAACAG
GGTGGAGCCTTAGAGTAGGGTAAGATTAGGACTCTAGGTTCTCTCATGGGTCCAGATCTG

>32 Get primers
GGAGAGCCACCTCACCTTGACAGGGTTGGCAGGAAGGGGCCCTAGAAAGTCAGTAGGATA
CGGGTAGTCCATCATGGCGAGCACAGTAAATGCATTTCGGGCAAACCCAAAGAGCTGAGT
CAGGTCCTTTGGGCTGGAAAGTGATTGACAGGTACCAAAGTTCTGGCTGATGGTGTCATA
GGCTGGGAAGAGAGAGGCCAGGAGAAAAGGCTGAGGAAACTGCTGGCAAATGTGAAGGGC
AAGAATGAATGCCCAAGGTGGGCAGCAGGTGAGGAAAGAGTCCCTCACCTCCCTGGAGGA
ACAAGTCTTTGATTTGCTGAAAGGCATCCCGCACAGCCTGGGCGCACTTGGGACTCTGGC
CATAAAAGTCCTGGAGAAGAGACCAAGGTTGCTGCTGCCATTCTTGCACTGGCCTGGGGT
ACCCAAGTCCCCTCACTCACCGCTGTGACATCTCGGAAGAATTGGTAGGAGTCCCCAAGG
CCTGCAACAGCTACAACAGGAGCGCTGGCTGCCAGTGCCCCAGCCACCAGGTGGGGGTAC
TTCATCCTCATGTAGGCACTCAGCATCCCCCCATAACTGGGAGTACAGAGCACAGATCAT
GGTTGTGGGAAGCTGCCCACAACTCAGGCGAGCAGCCTCACTGTCCTCCAGGCTGAGGTG
CTAGGCTGCTCTTTCCCTGCTCAGAACGCCCAAGGGTGGGAAAGAAGGACCTGAAACTGT
CAGGCCCACACACCCTGATCCCAGGGCCAAGGCAGATACAGCCTTCACTGGGAGAAGGCA
CCTGTGGGTGCCCTGCCCTGACCCAGCAATGAAGACATTGCAGAGACAAAGTCAGAAGGA
ATTGTCCCACTAGTGGGAACAACATAGCATACACTGCCTATGAGGTCCACTCAAGGAGGG
CTTCCAGAAGGAGGTAAAGCTAGACCCCGCCCTTCCACATGTGGGGTAGGCATAGGATGT
TGAGACTGTAAGAGACATCTCTTTGGCCCTCCTTGTATAGGGTGTCAATCGGCACAACAG
GGTGGAGCCTTAGAGTAGGGTAAGATTAGGACTCTAGGTTCTCTCATGGGTCCAGATCTG
TCATGAAGGGAGGTCAAGGACCCACCTCCCTCCAAAGGCTATGGTGGGGGCATCATGGAC
CCCAAGGTCCTGCCGCAGGGCCTGGAGCAGCACAGCAAAGTCGGCCAGCGCCTGCTCCAC

>42 Get primers
AGTCAGCAGCTGTGTATATCCCCGCTGTGTGGACTGGACACCGAACGGAAGCGATTTCCC
ATAGTACCGCTGCAGAAAGCAGGAAGGGATGGCTAATCCACTCCTCGGTGCTCCCCACCT
CCTTCAACTCAGGGACTGCCAGGAACTGTACAGGTACCCACGTGCTCAGCAAAGACAAGC
AGGGCCTCCTGCTGGGCTGCCAGTTCCACCATGAAGCCAGAGTTGTTAGCGAAGGACCAG
ATATCCCCCTCATTCCCTGTGTAGAAAAAGATGGGCCCTTCGCCCATCTTCCAGAACTTA
TCTGTTGGAAGTAAATGAGTTTCCATAAGGCCAGGGAAACGCAGGTAGGAACCCATGCGG
TCGAGCCAGCACTCACCTGACACTAGGAACCGCTGGCCAAAGGTTTTGTTGCCGAAACTC
TCAAAGTTGAAATGGTCCATGTATTGCTCAAAATAATTCTCATGAAAGTCAGGGTCTAGA
ACTCTGTCGGCTGAGGGCAGGTGCAGAGACTCAGGAGCTGGTTGGGATCATCAGGGATCT
AGGCGGGTCAGGAGGAAGGGCAGCCAGTCTGTACTCACCTCTGGCCTGGAGGTTGCACAG
TCCCAGTGACAGCAGCAGGACCAGGATCCAGGAGGGGACACCATGGTCCACAGGGTAACA
AGGATGGAAGTTCATGCTTGATTCTGAGCCGGGCGCTGACTGTCATGTGATTTGGTCACA
TGACCGACACAACGGGCGGGGCAGCATCACGTGATAGTCTGGCGGGGGCTGTCCTACTGT
GGCTGGATTCTAGTTGGAGGATCAGCCTACTCTTCTTCAGTTTCCCGGTTCCTCCAAATT
TCTGGGCTCCTACTTGTTTCCACAGAGATGGATACTGTGGAGGTCCAGGAAGCAGAGAGA
TGGCTAAGGCTCATCAGGACCGTATGATCTCCCAAGTGTCCAGCTACTGAGTACCACAAG
GTGATGGGTGGGAGGGTCCTCCCACGGAAGGATACCGCAGTCCCTAGGGGTTGCAAGCCC
CACATGTTCCACTGGCTGCTAGAGCTACCTACTCAATCAGCCCTGGGCATCACCATCAGG
TACTCGGCCAAAATGACCTCTCTGCTTCCAGTCCTCAGTTCTGGTCAGCACCAGACAGGC
CCATAATTACAGAGCCAGGGAAACTGGAACATTTGTCTCCCCTTAGACAGTGGCAGCAGG

>43 Get primers
CCTTCAACTCAGGGACTGCCAGGAACTGTACAGGTACCCACGTGCTCAGCAAAGACAAGC
AGGGCCTCCTGCTGGGCTGCCAGTTCCACCATGAAGCCAGAGTTGTTAGCGAAGGACCAG
ATATCCCCCTCATTCCCTGTGTAGAAAAAGATGGGCCCTTCGCCCATCTTCCAGAACTTA
TCTGTTGGAAGTAAATGAGTTTCCATAAGGCCAGGGAAACGCAGGTAGGAACCCATGCGG
TCGAGCCAGCACTCACCTGACACTAGGAACCGCTGGCCAAAGGTTTTGTTGCCGAAACTC
TCAAAGTTGAAATGGTCCATGTATTGCTCAAAATAATTCTCATGAAAGTCAGGGTCTAGA
ACTCTGTCGGCTGAGGGCAGGTGCAGAGACTCAGGAGCTGGTTGGGATCATCAGGGATCT
AGGCGGGTCAGGAGGAAGGGCAGCCAGTCTGTACTCACCTCTGGCCTGGAGGTTGCACAG
TCCCAGTGACAGCAGCAGGACCAGGATCCAGGAGGGGACACCATGGTCCACAGGGTAACA
AGGATGGAAGTTCATGCTTGATTCTGAGCCGGGCGCTGACTGTCATGTGATTTGGTCACA
TGACCGACACAACGGGCGGGGCAGCATCACGTGATAGTCTGGCGGGGGCTGTCCTACTGT
GGCTGGATTCTAGTTGGAGGATCAGCCTACTCTTCTTCAGTTTCCCGGTTCCTCCAAATT
TCTGGGCTCCTACTTGTTTCCACAGAGATGGATACTGTGGAGGTCCAGGAAGCAGAGAGA
TGGCTAAGGCTCATCAGGACCGTATGATCTCCCAAGTGTCCAGCTACTGAGTACCACAAG
GTGATGGGTGGGAGGGTCCTCCCACGGAAGGATACCGCAGTCCCTAGGGGTTGCAAGCCC
CACATGTTCCACTGGCTGCTAGAGCTACCTACTCAATCAGCCCTGGGCATCACCATCAGG
TACTCGGCCAAAATGACCTCTCTGCTTCCAGTCCTCAGTTCTGGTCAGCACCAGACAGGC
CCATAATTACAGAGCCAGGGAAACTGGAACATTTGTCTCCCCTTAGACAGTGGCAGCAGG
AAGGTGGGGGGTTGTTGCAGAGGAACAGTGTCTCTGAGAGAGGACCTTGGACTTTCTGGG
AATCTCTGAGCTGCCCGGTTCTCCCCACTGCTGGCACTGTGCCCACAGCCCAAACAGAAT

>44 Get primers
ATATCCCCCTCATTCCCTGTGTAGAAAAAGATGGGCCCTTCGCCCATCTTCCAGAACTTA
TCTGTTGGAAGTAAATGAGTTTCCATAAGGCCAGGGAAACGCAGGTAGGAACCCATGCGG
TCGAGCCAGCACTCACCTGACACTAGGAACCGCTGGCCAAAGGTTTTGTTGCCGAAACTC
TCAAAGTTGAAATGGTCCATGTATTGCTCAAAATAATTCTCATGAAAGTCAGGGTCTAGA
ACTCTGTCGGCTGAGGGCAGGTGCAGAGACTCAGGAGCTGGTTGGGATCATCAGGGATCT
AGGCGGGTCAGGAGGAAGGGCAGCCAGTCTGTACTCACCTCTGGCCTGGAGGTTGCACAG
TCCCAGTGACAGCAGCAGGACCAGGATCCAGGAGGGGACACCATGGTCCACAGGGTAACA
AGGATGGAAGTTCATGCTTGATTCTGAGCCGGGCGCTGACTGTCATGTGATTTGGTCACA
TGACCGACACAACGGGCGGGGCAGCATCACGTGATAGTCTGGCGGGGGCTGTCCTACTGT
GGCTGGATTCTAGTTGGAGGATCAGCCTACTCTTCTTCAGTTTCCCGGTTCCTCCAAATT
TCTGGGCTCCTACTTGTTTCCACAGAGATGGATACTGTGGAGGTCCAGGAAGCAGAGAGA
TGGCTAAGGCTCATCAGGACCGTATGATCTCCCAAGTGTCCAGCTACTGAGTACCACAAG
GTGATGGGTGGGAGGGTCCTCCCACGGAAGGATACCGCAGTCCCTAGGGGTTGCAAGCCC
CACATGTTCCACTGGCTGCTAGAGCTACCTACTCAATCAGCCCTGGGCATCACCATCAGG
TACTCGGCCAAAATGACCTCTCTGCTTCCAGTCCTCAGTTCTGGTCAGCACCAGACAGGC
CCATAATTACAGAGCCAGGGAAACTGGAACATTTGTCTCCCCTTAGACAGTGGCAGCAGG
AAGGTGGGGGGTTGTTGCAGAGGAACAGTGTCTCTGAGAGAGGACCTTGGACTTTCTGGG
AATCTCTGAGCTGCCCGGTTCTCCCCACTGCTGGCACTGTGCCCACAGCCCAAACAGAAT
GGGGGAGATGGAGGGGCAGGGCTTCTGTGGGAAGCTGCCCTCCACCTCATTGGCACAGAG
TGTCTCATTGCAGAGAGAAAAAAGGACCAGTTTTCTCTCTGGCACCCAGGTCTGGAAGAG

>45 Get primers
TCGAGCCAGCACTCACCTGACACTAGGAACCGCTGGCCAAAGGTTTTGTTGCCGAAACTC
TCAAAGTTGAAATGGTCCATGTATTGCTCAAAATAATTCTCATGAAAGTCAGGGTCTAGA
ACTCTGTCGGCTGAGGGCAGGTGCAGAGACTCAGGAGCTGGTTGGGATCATCAGGGATCT
AGGCGGGTCAGGAGGAAGGGCAGCCAGTCTGTACTCACCTCTGGCCTGGAGGTTGCACAG
TCCCAGTGACAGCAGCAGGACCAGGATCCAGGAGGGGACACCATGGTCCACAGGGTAACA
AGGATGGAAGTTCATGCTTGATTCTGAGCCGGGCGCTGACTGTCATGTGATTTGGTCACA
TGACCGACACAACGGGCGGGGCAGCATCACGTGATAGTCTGGCGGGGGCTGTCCTACTGT
GGCTGGATTCTAGTTGGAGGATCAGCCTACTCTTCTTCAGTTTCCCGGTTCCTCCAAATT
TCTGGGCTCCTACTTGTTTCCACAGAGATGGATACTGTGGAGGTCCAGGAAGCAGAGAGA
TGGCTAAGGCTCATCAGGACCGTATGATCTCCCAAGTGTCCAGCTACTGAGTACCACAAG
GTGATGGGTGGGAGGGTCCTCCCACGGAAGGATACCGCAGTCCCTAGGGGTTGCAAGCCC
CACATGTTCCACTGGCTGCTAGAGCTACCTACTCAATCAGCCCTGGGCATCACCATCAGG
TACTCGGCCAAAATGACCTCTCTGCTTCCAGTCCTCAGTTCTGGTCAGCACCAGACAGGC
CCATAATTACAGAGCCAGGGAAACTGGAACATTTGTCTCCCCTTAGACAGTGGCAGCAGG
AAGGTGGGGGGTTGTTGCAGAGGAACAGTGTCTCTGAGAGAGGACCTTGGACTTTCTGGG
AATCTCTGAGCTGCCCGGTTCTCCCCACTGCTGGCACTGTGCCCACAGCCCAAACAGAAT
GGGGGAGATGGAGGGGCAGGGCTTCTGTGGGAAGCTGCCCTCCACCTCATTGGCACAGAG
TGTCTCATTGCAGAGAGAAAAAAGGACCAGTTTTCTCTCTGGCACCCAGGTCTGGAAGAG
GAGTGACATCCACGGAAGTTGGTGACTTGGACTGGCTGGCCGTGAGTGGAACATGTCCAT
CCAGCATGGCCACAGTCCAGTGGGACACACAGCCTAGAGCTGTGGAATGCCGTGCCACAG

>46 Get primers
AGGCCAAGAAGTAGACCCACCTGCCAGTGCCCGTAGACCCATGATCCTCTGGCCCTCATT
CAACAGCCGTTGACAGCCCACCTAGAGAAAGGCAGCAGAATATCTCAGTGGAGGCCCCTT
TCACAGAGCGTGGGTCAGGGCTGCTAGCTTCCAGGACACAACAGCAGATAGTGTCTGATG
GCATGAAAGCAGATAGCTACAAGGCTCTTGGACCAGGCTAGCACTGGGTCCTGCACCCAG
GGAGAGCCACCTCACCTTGACAGGGTTGGCAGGAAGGGGCCCTAGAAAGTCAGTAGGATA
CGGGTAGTCCATCATGGCGAGCACAGTAAATGCATTTCGGGCAAACCCAAAGAGCTGAGT
CAGGTCCTTTGGGCTGGAAAGTGATTGACAGGTACCAAAGTTCTGGCTGATGGTGTCATA
GGCTGGGAAGAGAGAGGCCAGGAGAAAAGGCTGAGGAAACTGCTGGCAAATGTGAAGGGC
AAGAATGAATGCCCAAGGTGGGCAGCAGGTGAGGAAAGAGTCCCTCACCTCCCTGGAGGA
ACAAGTCTTTGATTTGCTGAAAGGCATCCCGCACAGCCTGGGCGCACTTGGGACTCTGGC
CATAAAAGTCCTGGAGAAGAGACCAAGGTTGCTGCTGCCATTCTTGCACTGGCCTGGGGT
ACCCAAGTCCCCTCACTCACCGCTGTGACATCTCGGAAGAATTGGTAGGAGTCCCCAAGG
CCTGCAACAGCTACAACAGGAGCGCTGGCTGCCAGTGCCCCAGCCACCAGGTGGGGGTAC
TTCATCCTCATGTAGGCACTCAGCATCCCCCCATAACTGGGAGTACAGAGCACAGATCAT
GGTTGTGGGAAGCTGCCCACAACTCAGGCGAGCAGCCTCACTGTCCTCCAGGCTGAGGTG
CTAGGCTGCTCTTTCCCTGCTCAGAACGCCCAAGGGTGGGAAAGAAGGACCTGAAACTGT
CAGGCCCACACACCCTGATCCCAGGGCCAAGGCAGATACAGCCTTCACTGGGAGAAGGCA
CCTGTGGGTGCCCTGCCCTGACCCAGCAATGAAGACATTGCAGAGACAAAGTCAGAAGGA
ATTGTCCCACTAGTGGGAACAACATAGCATACACTGCCTATGAGGTCCACTCAAGGAGGG
CTTCCAGAAG

>47 Get primers
CCCTTTCACAGAGCGTGGGTCAGGGCTGCTAGCTTCCAGGACACAACAGCAGATAGTGTC
TGATGGCATGAAAGCAGATAGCTACAAGGCTCTTGGACCAGGCTAGCACTGGGTCCTGCA
CCCAGGGAGAGCCACCTCACCTTGACAGGGTTGGCAGGAAGGGGCCCTAGAAAGTCAGTA
GGATACGGGTAGTCCATCATGGCGAGCACAGTAAATGCATTTCGGGCAAACCCAAAGAGC
TGAGTCAGGTCCTTTGGGCTGGAAAGTGATTGACAGGTACCAAAGTTCTGGCTGATGGTG
TCATAGGCTGGGAAGAGAGAGGCCAGGAGAAAAGGCTGAGGAAACTGCTGGCAAATGTGA
AGGGCAAGAATGAATGCCCAAGGTGGGCAGCAGGTGAGGAAAGAGTCCCTCACCTCCCTG
GAGGAACAAGTCTTTGATTTGCTGAAAGGCATCCCGCACAGCCTGGGCGCACTTGGGACT
CTGGCCATAAAAGTCCTGGAGAAGAGACCAAGGTTGCTGCTGCCATTCTTGCACTGGCCT
GGGGTACCCAAGTCCCCTCACTCACCGCTGTGACATCTCGGAAGAATTGGTAGGAGTCCC
CAAGGCCTGCAACAGCTACAACAGGAGCGCTGGCTGCCAGTGCCCCAGCCACCAGGTGGG
GGTACTTCATCCTCATGTAGGCACTCAGCATCCCCCCATAACTGGGAGTACAGAGCACAG
ATCATGGTTGTGGGAAGCTGCCCACAACTCAGGCGAGCAGCCTCACTGTCCTCCAGGCTG
AGGTGCTAGGCTGCTCTTTCCCTGCTCAGAACGCCCAAGGGTGGGAAAGAAGGACCTGAA
ACTGTCAGGCCCACACACCCTGATCCCAGGGCCAAGGCAGATACAGCCTTCACTGGGAGA
AGGCACCTGTGGGTGCCCTGCCCTGACCCAGCAATGAAGACATTGCAGAGACAAAGTCAG
AAGGAATTGTCCCACTAGTGGGAACAACATAGCATACACTGCCTATGAGGTCCACTCAAG
GAGGGCTTCCAGAAGGAGGTAAAGCTAGACCCCGCCCTTCCACATGTGGGGTAGGCATAG
GATGTTGAGACTGTAAGAGACATCTCTTTGGCCCTCCTTGTATAGGGTGTCAATCGGCAC
AACAGGGTGG

>48 Get primers
CTGCACCCAGGGAGAGCCACCTCACCTTGACAGGGTTGGCAGGAAGGGGCCCTAGAAAGT
CAGTAGGATACGGGTAGTCCATCATGGCGAGCACAGTAAATGCATTTCGGGCAAACCCAA
AGAGCTGAGTCAGGTCCTTTGGGCTGGAAAGTGATTGACAGGTACCAAAGTTCTGGCTGA
TGGTGTCATAGGCTGGGAAGAGAGAGGCCAGGAGAAAAGGCTGAGGAAACTGCTGGCAAA
TGTGAAGGGCAAGAATGAATGCCCAAGGTGGGCAGCAGGTGAGGAAAGAGTCCCTCACCT
CCCTGGAGGAACAAGTCTTTGATTTGCTGAAAGGCATCCCGCACAGCCTGGGCGCACTTG
GGACTCTGGCCATAAAAGTCCTGGAGAAGAGACCAAGGTTGCTGCTGCCATTCTTGCACT
GGCCTGGGGTACCCAAGTCCCCTCACTCACCGCTGTGACATCTCGGAAGAATTGGTAGGA
GTCCCCAAGGCCTGCAACAGCTACAACAGGAGCGCTGGCTGCCAGTGCCCCAGCCACCAG
GTGGGGGTACTTCATCCTCATGTAGGCACTCAGCATCCCCCCATAACTGGGAGTACAGAG
CACAGATCATGGTTGTGGGAAGCTGCCCACAACTCAGGCGAGCAGCCTCACTGTCCTCCA
GGCTGAGGTGCTAGGCTGCTCTTTCCCTGCTCAGAACGCCCAAGGGTGGGAAAGAAGGAC
CTGAAACTGTCAGGCCCACACACCCTGATCCCAGGGCCAAGGCAGATACAGCCTTCACTG
GGAGAAGGCACCTGTGGGTGCCCTGCCCTGACCCAGCAATGAAGACATTGCAGAGACAAA
GTCAGAAGGAATTGTCCCACTAGTGGGAACAACATAGCATACACTGCCTATGAGGTCCAC
TCAAGGAGGGCTTCCAGAAGGAGGTAAAGCTAGACCCCGCCCTTCCACATGTGGGGTAGG
CATAGGATGTTGAGACTGTAAGAGACATCTCTTTGGCCCTCCTTGTATAGGGTGTCAATC
GGCACAACAGGGTGGAGCCTTAGAGTAGGGTAAGATTAGGACTCTAGGTTCTCTCATGGG
TCCAGATCTGTCATGAAGGGAGGTCAAGGACCCACCTCCCTCCAAAGGCTATGGTGGGGG
CATCATGGAC

>59 Get primers
TTCCCATAGTACCGCTGCAGAAAGCAGGAAGGGATGGCTAATCCACTCCTCGGTGCTCCC
CACCTCCTTCAACTCAGGGACTGCCAGGAACTGTACAGGTACCCACGTGCTCAGCAAAGA
CAAGCAGGGCCTCCTGCTGGGCTGCCAGTTCCACCATGAAGCCAGAGTTGTTAGCGAAGG
ACCAGATATCCCCCTCATTCCCTGTGTAGAAAAAGATGGGCCCTTCGCCCATCTTCCAGA
ACTTATCTGTTGGAAGTAAATGAGTTTCCATAAGGCCAGGGAAACGCAGGTAGGAACCCA
TGCGGTCGAGCCAGCACTCACCTGACACTAGGAACCGCTGGCCAAAGGTTTTGTTGCCGA
AACTCTCAAAGTTGAAATGGTCCATGTATTGCTCAAAATAATTCTCATGAAAGTCAGGGT
CTAGAACTCTGTCGGCTGAGGGCAGGTGCAGAGACTCAGGAGCTGGTTGGGATCATCAGG
GATCTAGGCGGGTCAGGAGGAAGGGCAGCCAGTCTGTACTCACCTCTGGCCTGGAGGTTG
CACAGTCCCAGTGACAGCAGCAGGACCAGGATCCAGGAGGGGACACCATGGTCCACAGGG
TAACAAGGATGGAAGTTCATGCTTGATTCTGAGCCGGGCGCTGACTGTCATGTGATTTGG
TCACATGACCGACACAACGGGCGGGGCAGCATCACGTGATAGTCTGGCGGGGGCTGTCCT
ACTGTGGCTGGATTCTAGTTGGAGGATCAGCCTACTCTTCTTCAGTTTCCCGGTTCCTCC
AAATTTCTGGGCTCCTACTTGTTTCCACAGAGATGGATACTGTGGAGGTCCAGGAAGCAG
AGAGATGGCTAAGGCTCATCAGGACCGTATGATCTCCCAAGTGTCCAGCTACTGAGTACC
ACAAGGTGATGGGTGGGAGGGTCCTCCCACGGAAGGATACCGCAGTCCCTAGGGGTTGCA
AGCCCCACATGTTCCACTGGCTGCTAGAGCTACCTACTCAATCAGCCCTGGGCATCACCA
TCAGGTACTCGGCCAAAATGACCTCTCTGCTTCCAGTCCTCAGTTCTGGTCAGCACCAGA
CAGGCCCATAATTACAGAGCCAGGGAAACTGGAACATTTGTCTCCCCTTAGACAGTGGCA
GCAGGAAGGT

>60 Get primers
AAAGACAAGCAGGGCCTCCTGCTGGGCTGCCAGTTCCACCATGAAGCCAGAGTTGTTAGC
GAAGGACCAGATATCCCCCTCATTCCCTGTGTAGAAAAAGATGGGCCCTTCGCCCATCTT
CCAGAACTTATCTGTTGGAAGTAAATGAGTTTCCATAAGGCCAGGGAAACGCAGGTAGGA
ACCCATGCGGTCGAGCCAGCACTCACCTGACACTAGGAACCGCTGGCCAAAGGTTTTGTT
GCCGAAACTCTCAAAGTTGAAATGGTCCATGTATTGCTCAAAATAATTCTCATGAAAGTC
AGGGTCTAGAACTCTGTCGGCTGAGGGCAGGTGCAGAGACTCAGGAGCTGGTTGGGATCA
TCAGGGATCTAGGCGGGTCAGGAGGAAGGGCAGCCAGTCTGTACTCACCTCTGGCCTGGA
GGTTGCACAGTCCCAGTGACAGCAGCAGGACCAGGATCCAGGAGGGGACACCATGGTCCA
CAGGGTAACAAGGATGGAAGTTCATGCTTGATTCTGAGCCGGGCGCTGACTGTCATGTGA
TTTGGTCACATGACCGACACAACGGGCGGGGCAGCATCACGTGATAGTCTGGCGGGGGCT
GTCCTACTGTGGCTGGATTCTAGTTGGAGGATCAGCCTACTCTTCTTCAGTTTCCCGGTT
CCTCCAAATTTCTGGGCTCCTACTTGTTTCCACAGAGATGGATACTGTGGAGGTCCAGGA
AGCAGAGAGATGGCTAAGGCTCATCAGGACCGTATGATCTCCCAAGTGTCCAGCTACTGA
GTACCACAAGGTGATGGGTGGGAGGGTCCTCCCACGGAAGGATACCGCAGTCCCTAGGGG
TTGCAAGCCCCACATGTTCCACTGGCTGCTAGAGCTACCTACTCAATCAGCCCTGGGCAT
CACCATCAGGTACTCGGCCAAAATGACCTCTCTGCTTCCAGTCCTCAGTTCTGGTCAGCA
CCAGACAGGCCCATAATTACAGAGCCAGGGAAACTGGAACATTTGTCTCCCCTTAGACAG
TGGCAGCAGGAAGGTGGGGGGTTGTTGCAGAGGAACAGTGTCTCTGAGAGAGGACCTTGG
ACTTTCTGGGAATCTCTGAGCTGCCCGGTTCTCCCCACTGCTGGCACTGTGCCCACAGCC
CAAACAGAAT

>61 Get primers
ATCTTCCAGAACTTATCTGTTGGAAGTAAATGAGTTTCCATAAGGCCAGGGAAACGCAGG
TAGGAACCCATGCGGTCGAGCCAGCACTCACCTGACACTAGGAACCGCTGGCCAAAGGTT
TTGTTGCCGAAACTCTCAAAGTTGAAATGGTCCATGTATTGCTCAAAATAATTCTCATGA
AAGTCAGGGTCTAGAACTCTGTCGGCTGAGGGCAGGTGCAGAGACTCAGGAGCTGGTTGG
GATCATCAGGGATCTAGGCGGGTCAGGAGGAAGGGCAGCCAGTCTGTACTCACCTCTGGC
CTGGAGGTTGCACAGTCCCAGTGACAGCAGCAGGACCAGGATCCAGGAGGGGACACCATG
GTCCACAGGGTAACAAGGATGGAAGTTCATGCTTGATTCTGAGCCGGGCGCTGACTGTCA
TGTGATTTGGTCACATGACCGACACAACGGGCGGGGCAGCATCACGTGATAGTCTGGCGG
GGGCTGTCCTACTGTGGCTGGATTCTAGTTGGAGGATCAGCCTACTCTTCTTCAGTTTCC
CGGTTCCTCCAAATTTCTGGGCTCCTACTTGTTTCCACAGAGATGGATACTGTGGAGGTC
CAGGAAGCAGAGAGATGGCTAAGGCTCATCAGGACCGTATGATCTCCCAAGTGTCCAGCT
ACTGAGTACCACAAGGTGATGGGTGGGAGGGTCCTCCCACGGAAGGATACCGCAGTCCCT
AGGGGTTGCAAGCCCCACATGTTCCACTGGCTGCTAGAGCTACCTACTCAATCAGCCCTG
GGCATCACCATCAGGTACTCGGCCAAAATGACCTCTCTGCTTCCAGTCCTCAGTTCTGGT
CAGCACCAGACAGGCCCATAATTACAGAGCCAGGGAAACTGGAACATTTGTCTCCCCTTA
GACAGTGGCAGCAGGAAGGTGGGGGGTTGTTGCAGAGGAACAGTGTCTCTGAGAGAGGAC
CTTGGACTTTCTGGGAATCTCTGAGCTGCCCGGTTCTCCCCACTGCTGGCACTGTGCCCA
CAGCCCAAACAGAATGGGGGAGATGGAGGGGCAGGGCTTCTGTGGGAAGCTGCCCTCCAC
CTCATTGGCACAGAGTGTCTCATTGCAGAGAGAAAAAAGGACCAGTTTTCTCTCTGGCAC
CCAGGTCTGG

>62 Get primers
AGGTTTTGTTGCCGAAACTCTCAAAGTTGAAATGGTCCATGTATTGCTCAAAATAATTCT
CATGAAAGTCAGGGTCTAGAACTCTGTCGGCTGAGGGCAGGTGCAGAGACTCAGGAGCTG
GTTGGGATCATCAGGGATCTAGGCGGGTCAGGAGGAAGGGCAGCCAGTCTGTACTCACCT
CTGGCCTGGAGGTTGCACAGTCCCAGTGACAGCAGCAGGACCAGGATCCAGGAGGGGACA
CCATGGTCCACAGGGTAACAAGGATGGAAGTTCATGCTTGATTCTGAGCCGGGCGCTGAC
TGTCATGTGATTTGGTCACATGACCGACACAACGGGCGGGGCAGCATCACGTGATAGTCT
GGCGGGGGCTGTCCTACTGTGGCTGGATTCTAGTTGGAGGATCAGCCTACTCTTCTTCAG
TTTCCCGGTTCCTCCAAATTTCTGGGCTCCTACTTGTTTCCACAGAGATGGATACTGTGG
AGGTCCAGGAAGCAGAGAGATGGCTAAGGCTCATCAGGACCGTATGATCTCCCAAGTGTC
CAGCTACTGAGTACCACAAGGTGATGGGTGGGAGGGTCCTCCCACGGAAGGATACCGCAG
TCCCTAGGGGTTGCAAGCCCCACATGTTCCACTGGCTGCTAGAGCTACCTACTCAATCAG
CCCTGGGCATCACCATCAGGTACTCGGCCAAAATGACCTCTCTGCTTCCAGTCCTCAGTT
CTGGTCAGCACCAGACAGGCCCATAATTACAGAGCCAGGGAAACTGGAACATTTGTCTCC
CCTTAGACAGTGGCAGCAGGAAGGTGGGGGGTTGTTGCAGAGGAACAGTGTCTCTGAGAG
AGGACCTTGGACTTTCTGGGAATCTCTGAGCTGCCCGGTTCTCCCCACTGCTGGCACTGT
GCCCACAGCCCAAACAGAATGGGGGAGATGGAGGGGCAGGGCTTCTGTGGGAAGCTGCCC
TCCACCTCATTGGCACAGAGTGTCTCATTGCAGAGAGAAAAAAGGACCAGTTTTCTCTCT
GGCACCCAGGTCTGGAAGAGGAGTGACATCCACGGAAGTTGGTGACTTGGACTGGCTGGC
CGTGAGTGGAACATGTCCATCCAGCATGGCCACAGTCCAGTGGGACACACAGCCTAGAGC
TGTGGAATGC

>63 Get primers
AGGCCAAGAAGTAGACCCACCTGCCAGTGCCCGTAGACCCATGATCCTCTGGCCCTCATT
CAACAGCCGTTGACAGCCCACCTAGAGAAAGGCAGCAGAATATCTCAGTGGAGGCCCCTT
TCACAGAGCGTGGGTCAGGGCTGCTAGCTTCCAGGACACAACAGCAGATAGTGTCTGATG
GCATGAAAGCAGATAGCTACAAGGCTCTTGGACCAGGCTAGCACTGGGTCCTGCACCCAG
GGAGAGCCACCTCACCTTGACAGGGTTGGCAGGAAGGGGCCCTAGAAAGTCAGTAGGATA
CGGGTAGTCCATCATGGCGAGCACAGTAAATGCATTTCGGGCAAACCCAAAGAGCTGAGT
CAGGTCCTTTGGGCTGGAAAGTGATTGACAGGTACCAAAGTTCTGGCTGATGGTGTCATA
GGCTGGGAAGAGAGAGGCCAGGAGAAAAGGCTGAGGAAACTGCTGGCAAATGTGAAGGGC
AAGAATGAATGCCCAAGGTGGGCAGCAGGTGAGGAAAGAGTCCCTCACCTCCCTGGAGGA
ACAAGTCTTTGATTTGCTGAAAGGCATCCCGCACAGCCTGGGCGCACTTGGGACTCTGGC
CATAAAAGTCCTGGAGAAGAGACCAAGGTTGCTGCTGCCATTCTTGCACTGGCCTGGGGT
ACCCAAGTCCCCTCACTCACCGCTGTGACATCTCGGAAGAATTGGTAGGAGTCCCCAAGG
CCTGCAACAGCTACAACAGGAGCGCTGGCTGCCAGTGCCCCAGCCACCAGGTGGGGGTAC
TTCATCCTCATGTAGGCACTCAGCATCCCCCCATAACTGGGAGTACAGAGCACAGATCAT
GGTTGTGGGAAGCTGCCCACAACTCAGGCGAGCAGCCTCACTGTCCTCCAGGCTGAGGTG
CTAGGCTGCTCTTTCCCTGCTCAGAACGCCCAAGGGTGGGAAAGAAGGACCTGAAACTGT
CAGGCCCACACACCCTGATCCCAGGGCCAAGGCAGATACAGCCTTCACTGGGAGAAGGCA
CCTGTGGGTGCCCTGCCCTGACCCAGCAATGAAGACATTGCAGAGACAAAGTCAGAAGGA
ATTGTCCCACTAGTGGGAAC

>64 Get primers
GAGGCCCCTTTCACAGAGCGTGGGTCAGGGCTGCTAGCTTCCAGGACACAACAGCAGATA
GTGTCTGATGGCATGAAAGCAGATAGCTACAAGGCTCTTGGACCAGGCTAGCACTGGGTC
CTGCACCCAGGGAGAGCCACCTCACCTTGACAGGGTTGGCAGGAAGGGGCCCTAGAAAGT
CAGTAGGATACGGGTAGTCCATCATGGCGAGCACAGTAAATGCATTTCGGGCAAACCCAA
AGAGCTGAGTCAGGTCCTTTGGGCTGGAAAGTGATTGACAGGTACCAAAGTTCTGGCTGA
TGGTGTCATAGGCTGGGAAGAGAGAGGCCAGGAGAAAAGGCTGAGGAAACTGCTGGCAAA
TGTGAAGGGCAAGAATGAATGCCCAAGGTGGGCAGCAGGTGAGGAAAGAGTCCCTCACCT
CCCTGGAGGAACAAGTCTTTGATTTGCTGAAAGGCATCCCGCACAGCCTGGGCGCACTTG
GGACTCTGGCCATAAAAGTCCTGGAGAAGAGACCAAGGTTGCTGCTGCCATTCTTGCACT
GGCCTGGGGTACCCAAGTCCCCTCACTCACCGCTGTGACATCTCGGAAGAATTGGTAGGA
GTCCCCAAGGCCTGCAACAGCTACAACAGGAGCGCTGGCTGCCAGTGCCCCAGCCACCAG
GTGGGGGTACTTCATCCTCATGTAGGCACTCAGCATCCCCCCATAACTGGGAGTACAGAG
CACAGATCATGGTTGTGGGAAGCTGCCCACAACTCAGGCGAGCAGCCTCACTGTCCTCCA
GGCTGAGGTGCTAGGCTGCTCTTTCCCTGCTCAGAACGCCCAAGGGTGGGAAAGAAGGAC
CTGAAACTGTCAGGCCCACACACCCTGATCCCAGGGCCAAGGCAGATACAGCCTTCACTG
GGAGAAGGCACCTGTGGGTGCCCTGCCCTGACCCAGCAATGAAGACATTGCAGAGACAAA
GTCAGAAGGAATTGTCCCACTAGTGGGAACAACATAGCATACACTGCCTATGAGGTCCAC
TCAAGGAGGGCTTCCAGAAGGAGGTAAAGCTAGACCCCGCCCTTCCACATGTGGGGTAGG
CATAGGATGTTGAGACTGTA

>65 Get primers
GCACTGGGTCCTGCACCCAGGGAGAGCCACCTCACCTTGACAGGGTTGGCAGGAAGGGGC
CCTAGAAAGTCAGTAGGATACGGGTAGTCCATCATGGCGAGCACAGTAAATGCATTTCGG
GCAAACCCAAAGAGCTGAGTCAGGTCCTTTGGGCTGGAAAGTGATTGACAGGTACCAAAG
TTCTGGCTGATGGTGTCATAGGCTGGGAAGAGAGAGGCCAGGAGAAAAGGCTGAGGAAAC
TGCTGGCAAATGTGAAGGGCAAGAATGAATGCCCAAGGTGGGCAGCAGGTGAGGAAAGAG
TCCCTCACCTCCCTGGAGGAACAAGTCTTTGATTTGCTGAAAGGCATCCCGCACAGCCTG
GGCGCACTTGGGACTCTGGCCATAAAAGTCCTGGAGAAGAGACCAAGGTTGCTGCTGCCA
TTCTTGCACTGGCCTGGGGTACCCAAGTCCCCTCACTCACCGCTGTGACATCTCGGAAGA
ATTGGTAGGAGTCCCCAAGGCCTGCAACAGCTACAACAGGAGCGCTGGCTGCCAGTGCCC
CAGCCACCAGGTGGGGGTACTTCATCCTCATGTAGGCACTCAGCATCCCCCCATAACTGG
GAGTACAGAGCACAGATCATGGTTGTGGGAAGCTGCCCACAACTCAGGCGAGCAGCCTCA
CTGTCCTCCAGGCTGAGGTGCTAGGCTGCTCTTTCCCTGCTCAGAACGCCCAAGGGTGGG
AAAGAAGGACCTGAAACTGTCAGGCCCACACACCCTGATCCCAGGGCCAAGGCAGATACA
GCCTTCACTGGGAGAAGGCACCTGTGGGTGCCCTGCCCTGACCCAGCAATGAAGACATTG
CAGAGACAAAGTCAGAAGGAATTGTCCCACTAGTGGGAACAACATAGCATACACTGCCTA
TGAGGTCCACTCAAGGAGGGCTTCCAGAAGGAGGTAAAGCTAGACCCCGCCCTTCCACAT
GTGGGGTAGGCATAGGATGTTGAGACTGTAAGAGACATCTCTTTGGCCCTCCTTGTATAG
GGTGTCAATCGGCACAACAGGGTGGAGCCTTAGAGTAGGGTAAGATTAGGACTCTAGGTT
CTCTCATGGGTCCAGATCTG

>66 Get primers
TGCATTTCGGGCAAACCCAAAGAGCTGAGTCAGGTCCTTTGGGCTGGAAAGTGATTGACA
GGTACCAAAGTTCTGGCTGATGGTGTCATAGGCTGGGAAGAGAGAGGCCAGGAGAAAAGG
CTGAGGAAACTGCTGGCAAATGTGAAGGGCAAGAATGAATGCCCAAGGTGGGCAGCAGGT
GAGGAAAGAGTCCCTCACCTCCCTGGAGGAACAAGTCTTTGATTTGCTGAAAGGCATCCC
GCACAGCCTGGGCGCACTTGGGACTCTGGCCATAAAAGTCCTGGAGAAGAGACCAAGGTT
GCTGCTGCCATTCTTGCACTGGCCTGGGGTACCCAAGTCCCCTCACTCACCGCTGTGACA
TCTCGGAAGAATTGGTAGGAGTCCCCAAGGCCTGCAACAGCTACAACAGGAGCGCTGGCT
GCCAGTGCCCCAGCCACCAGGTGGGGGTACTTCATCCTCATGTAGGCACTCAGCATCCCC
CCATAACTGGGAGTACAGAGCACAGATCATGGTTGTGGGAAGCTGCCCACAACTCAGGCG
AGCAGCCTCACTGTCCTCCAGGCTGAGGTGCTAGGCTGCTCTTTCCCTGCTCAGAACGCC
CAAGGGTGGGAAAGAAGGACCTGAAACTGTCAGGCCCACACACCCTGATCCCAGGGCCAA
GGCAGATACAGCCTTCACTGGGAGAAGGCACCTGTGGGTGCCCTGCCCTGACCCAGCAAT
GAAGACATTGCAGAGACAAAGTCAGAAGGAATTGTCCCACTAGTGGGAACAACATAGCAT
ACACTGCCTATGAGGTCCACTCAAGGAGGGCTTCCAGAAGGAGGTAAAGCTAGACCCCGC
CCTTCCACATGTGGGGTAGGCATAGGATGTTGAGACTGTAAGAGACATCTCTTTGGCCCT
CCTTGTATAGGGTGTCAATCGGCACAACAGGGTGGAGCCTTAGAGTAGGGTAAGATTAGG
ACTCTAGGTTCTCTCATGGGTCCAGATCTGTCATGAAGGGAGGTCAAGGACCCACCTCCC
TCCAAAGGCTATGGTGGGGGCATCATGGACCCCAAGGTCCTGCCGCAGGGCCTGGAGCAG
CACAGCAAAGTCGGCCAGCG

>76 Get primers
CCTGCTCCACAGTCAGCAGCTGTGTATATCCCCGCTGTGTGGACTGGACACCGAACGGAA
GCGATTTCCCATAGTACCGCTGCAGAAAGCAGGAAGGGATGGCTAATCCACTCCTCGGTG
CTCCCCACCTCCTTCAACTCAGGGACTGCCAGGAACTGTACAGGTACCCACGTGCTCAGC
AAAGACAAGCAGGGCCTCCTGCTGGGCTGCCAGTTCCACCATGAAGCCAGAGTTGTTAGC
GAAGGACCAGATATCCCCCTCATTCCCTGTGTAGAAAAAGATGGGCCCTTCGCCCATCTT
CCAGAACTTATCTGTTGGAAGTAAATGAGTTTCCATAAGGCCAGGGAAACGCAGGTAGGA
ACCCATGCGGTCGAGCCAGCACTCACCTGACACTAGGAACCGCTGGCCAAAGGTTTTGTT
GCCGAAACTCTCAAAGTTGAAATGGTCCATGTATTGCTCAAAATAATTCTCATGAAAGTC
AGGGTCTAGAACTCTGTCGGCTGAGGGCAGGTGCAGAGACTCAGGAGCTGGTTGGGATCA
TCAGGGATCTAGGCGGGTCAGGAGGAAGGGCAGCCAGTCTGTACTCACCTCTGGCCTGGA
GGTTGCACAGTCCCAGTGACAGCAGCAGGACCAGGATCCAGGAGGGGACACCATGGTCCA
CAGGGTAACAAGGATGGAAGTTCATGCTTGATTCTGAGCCGGGCGCTGACTGTCATGTGA
TTTGGTCACATGACCGACACAACGGGCGGGGCAGCATCACGTGATAGTCTGGCGGGGGCT
GTCCTACTGTGGCTGGATTCTAGTTGGAGGATCAGCCTACTCTTCTTCAGTTTCCCGGTT
CCTCCAAATTTCTGGGCTCCTACTTGTTTCCACAGAGATGGATACTGTGGAGGTCCAGGA
AGCAGAGAGATGGCTAAGGCTCATCAGGACCGTATGATCTCCCAAGTGTCCAGCTACTGA
GTACCACAAGGTGATGGGTGGGAGGGTCCTCCCACGGAAGGATACCGCAGTCCCTAGGGG
TTGCAAGCCCCACATGTTCCACTGGCTGCTAGAGCTACCTACTCAATCAGCCCTGGGCAT
CACCATCAGGTACTCGGCCA

>77 Get primers
CTCCTCGGTGCTCCCCACCTCCTTCAACTCAGGGACTGCCAGGAACTGTACAGGTACCCA
CGTGCTCAGCAAAGACAAGCAGGGCCTCCTGCTGGGCTGCCAGTTCCACCATGAAGCCAG
AGTTGTTAGCGAAGGACCAGATATCCCCCTCATTCCCTGTGTAGAAAAAGATGGGCCCTT
CGCCCATCTTCCAGAACTTATCTGTTGGAAGTAAATGAGTTTCCATAAGGCCAGGGAAAC
GCAGGTAGGAACCCATGCGGTCGAGCCAGCACTCACCTGACACTAGGAACCGCTGGCCAA
AGGTTTTGTTGCCGAAACTCTCAAAGTTGAAATGGTCCATGTATTGCTCAAAATAATTCT
CATGAAAGTCAGGGTCTAGAACTCTGTCGGCTGAGGGCAGGTGCAGAGACTCAGGAGCTG
GTTGGGATCATCAGGGATCTAGGCGGGTCAGGAGGAAGGGCAGCCAGTCTGTACTCACCT
CTGGCCTGGAGGTTGCACAGTCCCAGTGACAGCAGCAGGACCAGGATCCAGGAGGGGACA
CCATGGTCCACAGGGTAACAAGGATGGAAGTTCATGCTTGATTCTGAGCCGGGCGCTGAC
TGTCATGTGATTTGGTCACATGACCGACACAACGGGCGGGGCAGCATCACGTGATAGTCT
GGCGGGGGCTGTCCTACTGTGGCTGGATTCTAGTTGGAGGATCAGCCTACTCTTCTTCAG
TTTCCCGGTTCCTCCAAATTTCTGGGCTCCTACTTGTTTCCACAGAGATGGATACTGTGG
AGGTCCAGGAAGCAGAGAGATGGCTAAGGCTCATCAGGACCGTATGATCTCCCAAGTGTC
CAGCTACTGAGTACCACAAGGTGATGGGTGGGAGGGTCCTCCCACGGAAGGATACCGCAG
TCCCTAGGGGTTGCAAGCCCCACATGTTCCACTGGCTGCTAGAGCTACCTACTCAATCAG
CCCTGGGCATCACCATCAGGTACTCGGCCAAAATGACCTCTCTGCTTCCAGTCCTCAGTT
CTGGTCAGCACCAGACAGGCCCATAATTACAGAGCCAGGGAAACTGGAACATTTGTCTCC
CCTTAGACAGTGGCAGCAGG

>78 Get primers
ATGAAGCCAGAGTTGTTAGCGAAGGACCAGATATCCCCCTCATTCCCTGTGTAGAAAAAG
ATGGGCCCTTCGCCCATCTTCCAGAACTTATCTGTTGGAAGTAAATGAGTTTCCATAAGG
CCAGGGAAACGCAGGTAGGAACCCATGCGGTCGAGCCAGCACTCACCTGACACTAGGAAC
CGCTGGCCAAAGGTTTTGTTGCCGAAACTCTCAAAGTTGAAATGGTCCATGTATTGCTCA
AAATAATTCTCATGAAAGTCAGGGTCTAGAACTCTGTCGGCTGAGGGCAGGTGCAGAGAC
TCAGGAGCTGGTTGGGATCATCAGGGATCTAGGCGGGTCAGGAGGAAGGGCAGCCAGTCT
GTACTCACCTCTGGCCTGGAGGTTGCACAGTCCCAGTGACAGCAGCAGGACCAGGATCCA
GGAGGGGACACCATGGTCCACAGGGTAACAAGGATGGAAGTTCATGCTTGATTCTGAGCC
GGGCGCTGACTGTCATGTGATTTGGTCACATGACCGACACAACGGGCGGGGCAGCATCAC
GTGATAGTCTGGCGGGGGCTGTCCTACTGTGGCTGGATTCTAGTTGGAGGATCAGCCTAC
TCTTCTTCAGTTTCCCGGTTCCTCCAAATTTCTGGGCTCCTACTTGTTTCCACAGAGATG
GATACTGTGGAGGTCCAGGAAGCAGAGAGATGGCTAAGGCTCATCAGGACCGTATGATCT
CCCAAGTGTCCAGCTACTGAGTACCACAAGGTGATGGGTGGGAGGGTCCTCCCACGGAAG
GATACCGCAGTCCCTAGGGGTTGCAAGCCCCACATGTTCCACTGGCTGCTAGAGCTACCT
ACTCAATCAGCCCTGGGCATCACCATCAGGTACTCGGCCAAAATGACCTCTCTGCTTCCA
GTCCTCAGTTCTGGTCAGCACCAGACAGGCCCATAATTACAGAGCCAGGGAAACTGGAAC
ATTTGTCTCCCCTTAGACAGTGGCAGCAGGAAGGTGGGGGGTTGTTGCAGAGGAACAGTG
TCTCTGAGAGAGGACCTTGGACTTTCTGGGAATCTCTGAGCTGCCCGGTTCTCCCCACTG
CTGGCACTGTGCCCACAGCC

>79 Get primers
TTCCATAAGGCCAGGGAAACGCAGGTAGGAACCCATGCGGTCGAGCCAGCACTCACCTGA
CACTAGGAACCGCTGGCCAAAGGTTTTGTTGCCGAAACTCTCAAAGTTGAAATGGTCCAT
GTATTGCTCAAAATAATTCTCATGAAAGTCAGGGTCTAGAACTCTGTCGGCTGAGGGCAG
GTGCAGAGACTCAGGAGCTGGTTGGGATCATCAGGGATCTAGGCGGGTCAGGAGGAAGGG
CAGCCAGTCTGTACTCACCTCTGGCCTGGAGGTTGCACAGTCCCAGTGACAGCAGCAGGA
CCAGGATCCAGGAGGGGACACCATGGTCCACAGGGTAACAAGGATGGAAGTTCATGCTTG
ATTCTGAGCCGGGCGCTGACTGTCATGTGATTTGGTCACATGACCGACACAACGGGCGGG
GCAGCATCACGTGATAGTCTGGCGGGGGCTGTCCTACTGTGGCTGGATTCTAGTTGGAGG
ATCAGCCTACTCTTCTTCAGTTTCCCGGTTCCTCCAAATTTCTGGGCTCCTACTTGTTTC
CACAGAGATGGATACTGTGGAGGTCCAGGAAGCAGAGAGATGGCTAAGGCTCATCAGGAC
CGTATGATCTCCCAAGTGTCCAGCTACTGAGTACCACAAGGTGATGGGTGGGAGGGTCCT
CCCACGGAAGGATACCGCAGTCCCTAGGGGTTGCAAGCCCCACATGTTCCACTGGCTGCT
AGAGCTACCTACTCAATCAGCCCTGGGCATCACCATCAGGTACTCGGCCAAAATGACCTC
TCTGCTTCCAGTCCTCAGTTCTGGTCAGCACCAGACAGGCCCATAATTACAGAGCCAGGG
AAACTGGAACATTTGTCTCCCCTTAGACAGTGGCAGCAGGAAGGTGGGGGGTTGTTGCAG
AGGAACAGTGTCTCTGAGAGAGGACCTTGGACTTTCTGGGAATCTCTGAGCTGCCCGGTT
CTCCCCACTGCTGGCACTGTGCCCACAGCCCAAACAGAATGGGGGAGATGGAGGGGCAGG
GCTTCTGTGGGAAGCTGCCCTCCACCTCATTGGCACAGAGTGTCTCATTGCAGAGAGAAA
AAAGGACCAGTTTTCTCTCT

>80 Get primers
AATGGTCCATGTATTGCTCAAAATAATTCTCATGAAAGTCAGGGTCTAGAACTCTGTCGG
CTGAGGGCAGGTGCAGAGACTCAGGAGCTGGTTGGGATCATCAGGGATCTAGGCGGGTCA
GGAGGAAGGGCAGCCAGTCTGTACTCACCTCTGGCCTGGAGGTTGCACAGTCCCAGTGAC
AGCAGCAGGACCAGGATCCAGGAGGGGACACCATGGTCCACAGGGTAACAAGGATGGAAG
TTCATGCTTGATTCTGAGCCGGGCGCTGACTGTCATGTGATTTGGTCACATGACCGACAC
AACGGGCGGGGCAGCATCACGTGATAGTCTGGCGGGGGCTGTCCTACTGTGGCTGGATTC
TAGTTGGAGGATCAGCCTACTCTTCTTCAGTTTCCCGGTTCCTCCAAATTTCTGGGCTCC
TACTTGTTTCCACAGAGATGGATACTGTGGAGGTCCAGGAAGCAGAGAGATGGCTAAGGC
TCATCAGGACCGTATGATCTCCCAAGTGTCCAGCTACTGAGTACCACAAGGTGATGGGTG
GGAGGGTCCTCCCACGGAAGGATACCGCAGTCCCTAGGGGTTGCAAGCCCCACATGTTCC
ACTGGCTGCTAGAGCTACCTACTCAATCAGCCCTGGGCATCACCATCAGGTACTCGGCCA
AAATGACCTCTCTGCTTCCAGTCCTCAGTTCTGGTCAGCACCAGACAGGCCCATAATTAC
AGAGCCAGGGAAACTGGAACATTTGTCTCCCCTTAGACAGTGGCAGCAGGAAGGTGGGGG
GTTGTTGCAGAGGAACAGTGTCTCTGAGAGAGGACCTTGGACTTTCTGGGAATCTCTGAG
CTGCCCGGTTCTCCCCACTGCTGGCACTGTGCCCACAGCCCAAACAGAATGGGGGAGATG
GAGGGGCAGGGCTTCTGTGGGAAGCTGCCCTCCACCTCATTGGCACAGAGTGTCTCATTG
CAGAGAGAAAAAAGGACCAGTTTTCTCTCTGGCACCCAGGTCTGGAAGAGGAGTGACATC
CACGGAAGTTGGTGACTTGGACTGGCTGGCCGTGAGTGGAACATGTCCATCCAGCATGGC
CACAGTCCAGTGGGACACAC

>81 Get primers
AGGCCAAGAAGTAGACCCACCTGCCAGTGCCCGTAGACCCATGATCCTCTGGCCCTCATT
CAACAGCCGTTGACAGCCCACCTAGAGAAAGGCAGCAGAATATCTCAGTGGAGGCCCCTT
TCACAGAGCGTGGGTCAGGGCTGCTAGCTTCCAGGACACAACAGCAGATAGTGTCTGATG
GCATGAAAGCAGATAGCTACAAGGCTCTTGGACCAGGCTAGCACTGGGTCCTGCACCCAG
GGAGAGCCACCTCACCTTGACAGGGTTGGCAGGAAGGGGCCCTAGAAAGTCAGTAGGATA
CGGGTAGTCCATCATGGCGAGCACAGTAAATGCATTTCGGGCAAACCCAAAGAGCTGAGT
CAGGTCCTTTGGGCTGGAAAGTGATTGACAGGTACCAAAGTTCTGGCTGATGGTGTCATA
GGCTGGGAAGAGAGAGGCCAGGAGAAAAGGCTGAGGAAACTGCTGGCAAATGTGAAGGGC
AAGAATGAATGCCCAAGGTGGGCAGCAGGTGAGGAAAGAGTCCCTCACCTCCCTGGAGGA
ACAAGTCTTTGATTTGCTGAAAGGCATCCCGCACAGCCTGGGCGCACTTGGGACTCTGGC
CATAAAAGTCCTGGAGAAGAGACCAAGGTTGCTGCTGCCATTCTTGCACTGGCCTGGGGT
ACCCAAGTCCCCTCACTCACCGCTGTGACATCTCGGAAGAATTGGTAGGAGTCCCCAAGG
CCTGCAACAGCTACAACAGGAGCGCTGGCTGCCAGTGCCCCAGCCACCAGGTGGGGGTAC
TTCATCCTCATGTAGGCACTCAGCATCCCCCCATAACTGGGAGTACAGAGCACAGATCAT
GGTTGTGGGAAGCTGCCCACAACTCAGGCGAGCAGCCTCACTGTCCTCCAGGCTGAGGTG
CTAGGCTGCTCTTTCCCTGCTCAGAACGCCCAAGGGTGGGAAAGAAGGACCTGAAACTGT
CAGGCCCACACACCCTGATCCCAGGGCCAAGGCAGATACAGCCTTCACTGGGAGAAGGCA
CCTGTGGGTGCCCTGCCCTGACCCAGCAAT

>82 Get primers
CAGTGGAGGCCCCTTTCACAGAGCGTGGGTCAGGGCTGCTAGCTTCCAGGACACAACAGC
AGATAGTGTCTGATGGCATGAAAGCAGATAGCTACAAGGCTCTTGGACCAGGCTAGCACT
GGGTCCTGCACCCAGGGAGAGCCACCTCACCTTGACAGGGTTGGCAGGAAGGGGCCCTAG
AAAGTCAGTAGGATACGGGTAGTCCATCATGGCGAGCACAGTAAATGCATTTCGGGCAAA
CCCAAAGAGCTGAGTCAGGTCCTTTGGGCTGGAAAGTGATTGACAGGTACCAAAGTTCTG
GCTGATGGTGTCATAGGCTGGGAAGAGAGAGGCCAGGAGAAAAGGCTGAGGAAACTGCTG
GCAAATGTGAAGGGCAAGAATGAATGCCCAAGGTGGGCAGCAGGTGAGGAAAGAGTCCCT
CACCTCCCTGGAGGAACAAGTCTTTGATTTGCTGAAAGGCATCCCGCACAGCCTGGGCGC
ACTTGGGACTCTGGCCATAAAAGTCCTGGAGAAGAGACCAAGGTTGCTGCTGCCATTCTT
GCACTGGCCTGGGGTACCCAAGTCCCCTCACTCACCGCTGTGACATCTCGGAAGAATTGG
TAGGAGTCCCCAAGGCCTGCAACAGCTACAACAGGAGCGCTGGCTGCCAGTGCCCCAGCC
ACCAGGTGGGGGTACTTCATCCTCATGTAGGCACTCAGCATCCCCCCATAACTGGGAGTA
CAGAGCACAGATCATGGTTGTGGGAAGCTGCCCACAACTCAGGCGAGCAGCCTCACTGTC
CTCCAGGCTGAGGTGCTAGGCTGCTCTTTCCCTGCTCAGAACGCCCAAGGGTGGGAAAGA
AGGACCTGAAACTGTCAGGCCCACACACCCTGATCCCAGGGCCAAGGCAGATACAGCCTT
CACTGGGAGAAGGCACCTGTGGGTGCCCTGCCCTGACCCAGCAATGAAGACATTGCAGAG
ACAAAGTCAGAAGGAATTGTCCCACTAGTGGGAACAACATAGCATACACTGCCTATGAGG
TCCACTCAAGGAGGGCTTCCAGAAGGAGGT

>83 Get primers
GACCAGGCTAGCACTGGGTCCTGCACCCAGGGAGAGCCACCTCACCTTGACAGGGTTGGC
AGGAAGGGGCCCTAGAAAGTCAGTAGGATACGGGTAGTCCATCATGGCGAGCACAGTAAA
TGCATTTCGGGCAAACCCAAAGAGCTGAGTCAGGTCCTTTGGGCTGGAAAGTGATTGACA
GGTACCAAAGTTCTGGCTGATGGTGTCATAGGCTGGGAAGAGAGAGGCCAGGAGAAAAGG
CTGAGGAAACTGCTGGCAAATGTGAAGGGCAAGAATGAATGCCCAAGGTGGGCAGCAGGT
GAGGAAAGAGTCCCTCACCTCCCTGGAGGAACAAGTCTTTGATTTGCTGAAAGGCATCCC
GCACAGCCTGGGCGCACTTGGGACTCTGGCCATAAAAGTCCTGGAGAAGAGACCAAGGTT
GCTGCTGCCATTCTTGCACTGGCCTGGGGTACCCAAGTCCCCTCACTCACCGCTGTGACA
TCTCGGAAGAATTGGTAGGAGTCCCCAAGGCCTGCAACAGCTACAACAGGAGCGCTGGCT
GCCAGTGCCCCAGCCACCAGGTGGGGGTACTTCATCCTCATGTAGGCACTCAGCATCCCC
CCATAACTGGGAGTACAGAGCACAGATCATGGTTGTGGGAAGCTGCCCACAACTCAGGCG
AGCAGCCTCACTGTCCTCCAGGCTGAGGTGCTAGGCTGCTCTTTCCCTGCTCAGAACGCC
CAAGGGTGGGAAAGAAGGACCTGAAACTGTCAGGCCCACACACCCTGATCCCAGGGCCAA
GGCAGATACAGCCTTCACTGGGAGAAGGCACCTGTGGGTGCCCTGCCCTGACCCAGCAAT
GAAGACATTGCAGAGACAAAGTCAGAAGGAATTGTCCCACTAGTGGGAACAACATAGCAT
ACACTGCCTATGAGGTCCACTCAAGGAGGGCTTCCAGAAGGAGGTAAAGCTAGACCCCGC
CCTTCCACATGTGGGGTAGGCATAGGATGTTGAGACTGTAAGAGACATCTCTTTGGCCCT
CCTTGTATAGGGTGTCAATCGGCACAACAG

>84 Get primers
GGCGAGCACAGTAAATGCATTTCGGGCAAACCCAAAGAGCTGAGTCAGGTCCTTTGGGCT
GGAAAGTGATTGACAGGTACCAAAGTTCTGGCTGATGGTGTCATAGGCTGGGAAGAGAGA
GGCCAGGAGAAAAGGCTGAGGAAACTGCTGGCAAATGTGAAGGGCAAGAATGAATGCCCA
AGGTGGGCAGCAGGTGAGGAAAGAGTCCCTCACCTCCCTGGAGGAACAAGTCTTTGATTT
GCTGAAAGGCATCCCGCACAGCCTGGGCGCACTTGGGACTCTGGCCATAAAAGTCCTGGA
GAAGAGACCAAGGTTGCTGCTGCCATTCTTGCACTGGCCTGGGGTACCCAAGTCCCCTCA
CTCACCGCTGTGACATCTCGGAAGAATTGGTAGGAGTCCCCAAGGCCTGCAACAGCTACA
ACAGGAGCGCTGGCTGCCAGTGCCCCAGCCACCAGGTGGGGGTACTTCATCCTCATGTAG
GCACTCAGCATCCCCCCATAACTGGGAGTACAGAGCACAGATCATGGTTGTGGGAAGCTG
CCCACAACTCAGGCGAGCAGCCTCACTGTCCTCCAGGCTGAGGTGCTAGGCTGCTCTTTC
CCTGCTCAGAACGCCCAAGGGTGGGAAAGAAGGACCTGAAACTGTCAGGCCCACACACCC
TGATCCCAGGGCCAAGGCAGATACAGCCTTCACTGGGAGAAGGCACCTGTGGGTGCCCTG
CCCTGACCCAGCAATGAAGACATTGCAGAGACAAAGTCAGAAGGAATTGTCCCACTAGTG
GGAACAACATAGCATACACTGCCTATGAGGTCCACTCAAGGAGGGCTTCCAGAAGGAGGT
AAAGCTAGACCCCGCCCTTCCACATGTGGGGTAGGCATAGGATGTTGAGACTGTAAGAGA
CATCTCTTTGGCCCTCCTTGTATAGGGTGTCAATCGGCACAACAGGGTGGAGCCTTAGAG
TAGGGTAAGATTAGGACTCTAGGTTCTCTCATGGGTCCAGATCTGTCATGAAGGGAGGTC
AAGGACCCACCTCCCTCCAAAGGCTATGGT

>95 Get primers
GGACTGGACACCGAACGGAAGCGATTTCCCATAGTACCGCTGCAGAAAGCAGGAAGGGAT
GGCTAATCCACTCCTCGGTGCTCCCCACCTCCTTCAACTCAGGGACTGCCAGGAACTGTA
CAGGTACCCACGTGCTCAGCAAAGACAAGCAGGGCCTCCTGCTGGGCTGCCAGTTCCACC
ATGAAGCCAGAGTTGTTAGCGAAGGACCAGATATCCCCCTCATTCCCTGTGTAGAAAAAG
ATGGGCCCTTCGCCCATCTTCCAGAACTTATCTGTTGGAAGTAAATGAGTTTCCATAAGG
CCAGGGAAACGCAGGTAGGAACCCATGCGGTCGAGCCAGCACTCACCTGACACTAGGAAC
CGCTGGCCAAAGGTTTTGTTGCCGAAACTCTCAAAGTTGAAATGGTCCATGTATTGCTCA
AAATAATTCTCATGAAAGTCAGGGTCTAGAACTCTGTCGGCTGAGGGCAGGTGCAGAGAC
TCAGGAGCTGGTTGGGATCATCAGGGATCTAGGCGGGTCAGGAGGAAGGGCAGCCAGTCT
GTACTCACCTCTGGCCTGGAGGTTGCACAGTCCCAGTGACAGCAGCAGGACCAGGATCCA
GGAGGGGACACCATGGTCCACAGGGTAACAAGGATGGAAGTTCATGCTTGATTCTGAGCC
GGGCGCTGACTGTCATGTGATTTGGTCACATGACCGACACAACGGGCGGGGCAGCATCAC
GTGATAGTCTGGCGGGGGCTGTCCTACTGTGGCTGGATTCTAGTTGGAGGATCAGCCTAC
TCTTCTTCAGTTTCCCGGTTCCTCCAAATTTCTGGGCTCCTACTTGTTTCCACAGAGATG
GATACTGTGGAGGTCCAGGAAGCAGAGAGATGGCTAAGGCTCATCAGGACCGTATGATCT
CCCAAGTGTCCAGCTACTGAGTACCACAAGGTGATGGGTGGGAGGGTCCTCCCACGGAAG
GATACCGCAGTCCCTAGGGGTTGCAAGCCCCACATGTTCCACTGGCTGCTAGAGCTACCT
ACTCAATCAGCCCTGGGCATCACCATCAGG

>96 Get primers
CTGCCAGGAACTGTACAGGTACCCACGTGCTCAGCAAAGACAAGCAGGGCCTCCTGCTGG
GCTGCCAGTTCCACCATGAAGCCAGAGTTGTTAGCGAAGGACCAGATATCCCCCTCATTC
CCTGTGTAGAAAAAGATGGGCCCTTCGCCCATCTTCCAGAACTTATCTGTTGGAAGTAAA
TGAGTTTCCATAAGGCCAGGGAAACGCAGGTAGGAACCCATGCGGTCGAGCCAGCACTCA
CCTGACACTAGGAACCGCTGGCCAAAGGTTTTGTTGCCGAAACTCTCAAAGTTGAAATGG
TCCATGTATTGCTCAAAATAATTCTCATGAAAGTCAGGGTCTAGAACTCTGTCGGCTGAG
GGCAGGTGCAGAGACTCAGGAGCTGGTTGGGATCATCAGGGATCTAGGCGGGTCAGGAGG
AAGGGCAGCCAGTCTGTACTCACCTCTGGCCTGGAGGTTGCACAGTCCCAGTGACAGCAG
CAGGACCAGGATCCAGGAGGGGACACCATGGTCCACAGGGTAACAAGGATGGAAGTTCAT
GCTTGATTCTGAGCCGGGCGCTGACTGTCATGTGATTTGGTCACATGACCGACACAACGG
GCGGGGCAGCATCACGTGATAGTCTGGCGGGGGCTGTCCTACTGTGGCTGGATTCTAGTT
GGAGGATCAGCCTACTCTTCTTCAGTTTCCCGGTTCCTCCAAATTTCTGGGCTCCTACTT
GTTTCCACAGAGATGGATACTGTGGAGGTCCAGGAAGCAGAGAGATGGCTAAGGCTCATC
AGGACCGTATGATCTCCCAAGTGTCCAGCTACTGAGTACCACAAGGTGATGGGTGGGAGG
GTCCTCCCACGGAAGGATACCGCAGTCCCTAGGGGTTGCAAGCCCCACATGTTCCACTGG
CTGCTAGAGCTACCTACTCAATCAGCCCTGGGCATCACCATCAGGTACTCGGCCAAAATG
ACCTCTCTGCTTCCAGTCCTCAGTTCTGGTCAGCACCAGACAGGCCCATAATTACAGAGC
CAGGGAAACTGGAACATTTGTCTCCCCTTA

>97 Get primers
ATATCCCCCTCATTCCCTGTGTAGAAAAAGATGGGCCCTTCGCCCATCTTCCAGAACTTA
TCTGTTGGAAGTAAATGAGTTTCCATAAGGCCAGGGAAACGCAGGTAGGAACCCATGCGG
TCGAGCCAGCACTCACCTGACACTAGGAACCGCTGGCCAAAGGTTTTGTTGCCGAAACTC
TCAAAGTTGAAATGGTCCATGTATTGCTCAAAATAATTCTCATGAAAGTCAGGGTCTAGA
ACTCTGTCGGCTGAGGGCAGGTGCAGAGACTCAGGAGCTGGTTGGGATCATCAGGGATCT
AGGCGGGTCAGGAGGAAGGGCAGCCAGTCTGTACTCACCTCTGGCCTGGAGGTTGCACAG
TCCCAGTGACAGCAGCAGGACCAGGATCCAGGAGGGGACACCATGGTCCACAGGGTAACA
AGGATGGAAGTTCATGCTTGATTCTGAGCCGGGCGCTGACTGTCATGTGATTTGGTCACA
TGACCGACACAACGGGCGGGGCAGCATCACGTGATAGTCTGGCGGGGGCTGTCCTACTGT
GGCTGGATTCTAGTTGGAGGATCAGCCTACTCTTCTTCAGTTTCCCGGTTCCTCCAAATT
TCTGGGCTCCTACTTGTTTCCACAGAGATGGATACTGTGGAGGTCCAGGAAGCAGAGAGA
TGGCTAAGGCTCATCAGGACCGTATGATCTCCCAAGTGTCCAGCTACTGAGTACCACAAG
GTGATGGGTGGGAGGGTCCTCCCACGGAAGGATACCGCAGTCCCTAGGGGTTGCAAGCCC
CACATGTTCCACTGGCTGCTAGAGCTACCTACTCAATCAGCCCTGGGCATCACCATCAGG
TACTCGGCCAAAATGACCTCTCTGCTTCCAGTCCTCAGTTCTGGTCAGCACCAGACAGGC
CCATAATTACAGAGCCAGGGAAACTGGAACATTTGTCTCCCCTTAGACAGTGGCAGCAGG
AAGGTGGGGGGTTGTTGCAGAGGAACAGTGTCTCTGAGAGAGGACCTTGGACTTTCTGGG
AATCTCTGAGCTGCCCGGTTCTCCCCACTG

>98 Get primers
TAGGAACCCATGCGGTCGAGCCAGCACTCACCTGACACTAGGAACCGCTGGCCAAAGGTT
TTGTTGCCGAAACTCTCAAAGTTGAAATGGTCCATGTATTGCTCAAAATAATTCTCATGA
AAGTCAGGGTCTAGAACTCTGTCGGCTGAGGGCAGGTGCAGAGACTCAGGAGCTGGTTGG
GATCATCAGGGATCTAGGCGGGTCAGGAGGAAGGGCAGCCAGTCTGTACTCACCTCTGGC
CTGGAGGTTGCACAGTCCCAGTGACAGCAGCAGGACCAGGATCCAGGAGGGGACACCATG
GTCCACAGGGTAACAAGGATGGAAGTTCATGCTTGATTCTGAGCCGGGCGCTGACTGTCA
TGTGATTTGGTCACATGACCGACACAACGGGCGGGGCAGCATCACGTGATAGTCTGGCGG
GGGCTGTCCTACTGTGGCTGGATTCTAGTTGGAGGATCAGCCTACTCTTCTTCAGTTTCC
CGGTTCCTCCAAATTTCTGGGCTCCTACTTGTTTCCACAGAGATGGATACTGTGGAGGTC
CAGGAAGCAGAGAGATGGCTAAGGCTCATCAGGACCGTATGATCTCCCAAGTGTCCAGCT
ACTGAGTACCACAAGGTGATGGGTGGGAGGGTCCTCCCACGGAAGGATACCGCAGTCCCT
AGGGGTTGCAAGCCCCACATGTTCCACTGGCTGCTAGAGCTACCTACTCAATCAGCCCTG
GGCATCACCATCAGGTACTCGGCCAAAATGACCTCTCTGCTTCCAGTCCTCAGTTCTGGT
CAGCACCAGACAGGCCCATAATTACAGAGCCAGGGAAACTGGAACATTTGTCTCCCCTTA
GACAGTGGCAGCAGGAAGGTGGGGGGTTGTTGCAGAGGAACAGTGTCTCTGAGAGAGGAC
CTTGGACTTTCTGGGAATCTCTGAGCTGCCCGGTTCTCCCCACTGCTGGCACTGTGCCCA
CAGCCCAAACAGAATGGGGGAGATGGAGGGGCAGGGCTTCTGTGGGAAGCTGCCCTCCAC
CTCATTGGCACAGAGTGTCTCATTGCAGAG

>99 Get primers
AAATAATTCTCATGAAAGTCAGGGTCTAGAACTCTGTCGGCTGAGGGCAGGTGCAGAGAC
TCAGGAGCTGGTTGGGATCATCAGGGATCTAGGCGGGTCAGGAGGAAGGGCAGCCAGTCT
GTACTCACCTCTGGCCTGGAGGTTGCACAGTCCCAGTGACAGCAGCAGGACCAGGATCCA
GGAGGGGACACCATGGTCCACAGGGTAACAAGGATGGAAGTTCATGCTTGATTCTGAGCC
GGGCGCTGACTGTCATGTGATTTGGTCACATGACCGACACAACGGGCGGGGCAGCATCAC
GTGATAGTCTGGCGGGGGCTGTCCTACTGTGGCTGGATTCTAGTTGGAGGATCAGCCTAC
TCTTCTTCAGTTTCCCGGTTCCTCCAAATTTCTGGGCTCCTACTTGTTTCCACAGAGATG
GATACTGTGGAGGTCCAGGAAGCAGAGAGATGGCTAAGGCTCATCAGGACCGTATGATCT
CCCAAGTGTCCAGCTACTGAGTACCACAAGGTGATGGGTGGGAGGGTCCTCCCACGGAAG
GATACCGCAGTCCCTAGGGGTTGCAAGCCCCACATGTTCCACTGGCTGCTAGAGCTACCT
ACTCAATCAGCCCTGGGCATCACCATCAGGTACTCGGCCAAAATGACCTCTCTGCTTCCA
GTCCTCAGTTCTGGTCAGCACCAGACAGGCCCATAATTACAGAGCCAGGGAAACTGGAAC
ATTTGTCTCCCCTTAGACAGTGGCAGCAGGAAGGTGGGGGGTTGTTGCAGAGGAACAGTG
TCTCTGAGAGAGGACCTTGGACTTTCTGGGAATCTCTGAGCTGCCCGGTTCTCCCCACTG
CTGGCACTGTGCCCACAGCCCAAACAGAATGGGGGAGATGGAGGGGCAGGGCTTCTGTGG
GAAGCTGCCCTCCACCTCATTGGCACAGAGTGTCTCATTGCAGAGAGAAAAAAGGACCAG
TTTTCTCTCTGGCACCCAGGTCTGGAAGAGGAGTGACATCCACGGAAGTTGGTGACTTGG
ACTGGCTGGCCGTGAGTGGAACATGTCCAT

>100 Get primers
AGGCCAAGAAGTAGACCCACCTGCCAGTGCCCGTAGACCCATGATCCTCTGGCCCTCATT
CAACAGCCGTTGACAGCCCACCTAGAGAAAGGCAGCAGAATATCTCAGTGGAGGCCCCTT
TCACAGAGCGTGGGTCAGGGCTGCTAGCTTCCAGGACACAACAGCAGATAGTGTCTGATG
GCATGAAAGCAGATAGCTACAAGGCTCTTGGACCAGGCTAGCACTGGGTCCTGCACCCAG
GGAGAGCCACCTCACCTTGACAGGGTTGGCAGGAAGGGGCCCTAGAAAGTCAGTAGGATA
CGGGTAGTCCATCATGGCGAGCACAGTAAATGCATTTCGGGCAAACCCAAAGAGCTGAGT
CAGGTCCTTTGGGCTGGAAAGTGATTGACAGGTACCAAAGTTCTGGCTGATGGTGTCATA
GGCTGGGAAGAGAGAGGCCAGGAGAAAAGGCTGAGGAAACTGCTGGCAAATGTGAAGGGC
AAGAATGAATGCCCAAGGTGGGCAGCAGGTGAGGAAAGAGTCCCTCACCTCCCTGGAGGA
ACAAGTCTTTGATTTGCTGAAAGGCATCCCGCACAGCCTGGGCGCACTTGGGACTCTGGC
CATAAAAGTCCTGGAGAAGAGACCAAGGTTGCTGCTGCCATTCTTGCACTGGCCTGGGGT
ACCCAAGTCCCCTCACTCACCGCTGTGACATCTCGGAAGAATTGGTAGGAGTCCCCAAGG
CCTGCAACAGCTACAACAGGAGCGCTGGCTGCCAGTGCCCCAGCCACCAGGTGGGGGTAC
TTCATCCTCATGTAGGCACTCAGCATCCCCCCATAACTGGGAGTACAGAGCACAGATCAT
GGTTGTGGGAAGCTGCCCACAACTCAGGCGAGCAGCCTCACTGTCCTCCAGGCTGAGGTG
CTAGGCTGCTCTTTCCCTGCTCAGAACGCCCAAGGGTGGGAAAGAAGGACCTGAAACTGT
CAGGCCCACACACCCTGATCCCAGGGCCAAGGCAGATACA

>101 Get primers
TATCTCAGTGGAGGCCCCTTTCACAGAGCGTGGGTCAGGGCTGCTAGCTTCCAGGACACA
ACAGCAGATAGTGTCTGATGGCATGAAAGCAGATAGCTACAAGGCTCTTGGACCAGGCTA
GCACTGGGTCCTGCACCCAGGGAGAGCCACCTCACCTTGACAGGGTTGGCAGGAAGGGGC
CCTAGAAAGTCAGTAGGATACGGGTAGTCCATCATGGCGAGCACAGTAAATGCATTTCGG
GCAAACCCAAAGAGCTGAGTCAGGTCCTTTGGGCTGGAAAGTGATTGACAGGTACCAAAG
TTCTGGCTGATGGTGTCATAGGCTGGGAAGAGAGAGGCCAGGAGAAAAGGCTGAGGAAAC
TGCTGGCAAATGTGAAGGGCAAGAATGAATGCCCAAGGTGGGCAGCAGGTGAGGAAAGAG
TCCCTCACCTCCCTGGAGGAACAAGTCTTTGATTTGCTGAAAGGCATCCCGCACAGCCTG
GGCGCACTTGGGACTCTGGCCATAAAAGTCCTGGAGAAGAGACCAAGGTTGCTGCTGCCA
TTCTTGCACTGGCCTGGGGTACCCAAGTCCCCTCACTCACCGCTGTGACATCTCGGAAGA
ATTGGTAGGAGTCCCCAAGGCCTGCAACAGCTACAACAGGAGCGCTGGCTGCCAGTGCCC
CAGCCACCAGGTGGGGGTACTTCATCCTCATGTAGGCACTCAGCATCCCCCCATAACTGG
GAGTACAGAGCACAGATCATGGTTGTGGGAAGCTGCCCACAACTCAGGCGAGCAGCCTCA
CTGTCCTCCAGGCTGAGGTGCTAGGCTGCTCTTTCCCTGCTCAGAACGCCCAAGGGTGGG
AAAGAAGGACCTGAAACTGTCAGGCCCACACACCCTGATCCCAGGGCCAAGGCAGATACA
GCCTTCACTGGGAGAAGGCACCTGTGGGTGCCCTGCCCTGACCCAGCAATGAAGACATTG
CAGAGACAAAGTCAGAAGGAATTGTCCCACTAGTGGGAAC

>102 Get primers
AAGGCTCTTGGACCAGGCTAGCACTGGGTCCTGCACCCAGGGAGAGCCACCTCACCTTGA
CAGGGTTGGCAGGAAGGGGCCCTAGAAAGTCAGTAGGATACGGGTAGTCCATCATGGCGA
GCACAGTAAATGCATTTCGGGCAAACCCAAAGAGCTGAGTCAGGTCCTTTGGGCTGGAAA
GTGATTGACAGGTACCAAAGTTCTGGCTGATGGTGTCATAGGCTGGGAAGAGAGAGGCCA
GGAGAAAAGGCTGAGGAAACTGCTGGCAAATGTGAAGGGCAAGAATGAATGCCCAAGGTG
GGCAGCAGGTGAGGAAAGAGTCCCTCACCTCCCTGGAGGAACAAGTCTTTGATTTGCTGA
AAGGCATCCCGCACAGCCTGGGCGCACTTGGGACTCTGGCCATAAAAGTCCTGGAGAAGA
GACCAAGGTTGCTGCTGCCATTCTTGCACTGGCCTGGGGTACCCAAGTCCCCTCACTCAC
CGCTGTGACATCTCGGAAGAATTGGTAGGAGTCCCCAAGGCCTGCAACAGCTACAACAGG
AGCGCTGGCTGCCAGTGCCCCAGCCACCAGGTGGGGGTACTTCATCCTCATGTAGGCACT
CAGCATCCCCCCATAACTGGGAGTACAGAGCACAGATCATGGTTGTGGGAAGCTGCCCAC
AACTCAGGCGAGCAGCCTCACTGTCCTCCAGGCTGAGGTGCTAGGCTGCTCTTTCCCTGC
TCAGAACGCCCAAGGGTGGGAAAGAAGGACCTGAAACTGTCAGGCCCACACACCCTGATC
CCAGGGCCAAGGCAGATACAGCCTTCACTGGGAGAAGGCACCTGTGGGTGCCCTGCCCTG
ACCCAGCAATGAAGACATTGCAGAGACAAAGTCAGAAGGAATTGTCCCACTAGTGGGAAC
AACATAGCATACACTGCCTATGAGGTCCACTCAAGGAGGGCTTCCAGAAGGAGGTAAAGC
TAGACCCCGCCCTTCCACATGTGGGGTAGGCATAGGATGT

>103 Get primers
CGGGTAGTCCATCATGGCGAGCACAGTAAATGCATTTCGGGCAAACCCAAAGAGCTGAGT
CAGGTCCTTTGGGCTGGAAAGTGATTGACAGGTACCAAAGTTCTGGCTGATGGTGTCATA
GGCTGGGAAGAGAGAGGCCAGGAGAAAAGGCTGAGGAAACTGCTGGCAAATGTGAAGGGC
AAGAATGAATGCCCAAGGTGGGCAGCAGGTGAGGAAAGAGTCCCTCACCTCCCTGGAGGA
ACAAGTCTTTGATTTGCTGAAAGGCATCCCGCACAGCCTGGGCGCACTTGGGACTCTGGC
CATAAAAGTCCTGGAGAAGAGACCAAGGTTGCTGCTGCCATTCTTGCACTGGCCTGGGGT
ACCCAAGTCCCCTCACTCACCGCTGTGACATCTCGGAAGAATTGGTAGGAGTCCCCAAGG
CCTGCAACAGCTACAACAGGAGCGCTGGCTGCCAGTGCCCCAGCCACCAGGTGGGGGTAC
TTCATCCTCATGTAGGCACTCAGCATCCCCCCATAACTGGGAGTACAGAGCACAGATCAT
GGTTGTGGGAAGCTGCCCACAACTCAGGCGAGCAGCCTCACTGTCCTCCAGGCTGAGGTG
CTAGGCTGCTCTTTCCCTGCTCAGAACGCCCAAGGGTGGGAAAGAAGGACCTGAAACTGT
CAGGCCCACACACCCTGATCCCAGGGCCAAGGCAGATACAGCCTTCACTGGGAGAAGGCA
CCTGTGGGTGCCCTGCCCTGACCCAGCAATGAAGACATTGCAGAGACAAAGTCAGAAGGA
ATTGTCCCACTAGTGGGAACAACATAGCATACACTGCCTATGAGGTCCACTCAAGGAGGG
CTTCCAGAAGGAGGTAAAGCTAGACCCCGCCCTTCCACATGTGGGGTAGGCATAGGATGT
TGAGACTGTAAGAGACATCTCTTTGGCCCTCCTTGTATAGGGTGTCAATCGGCACAACAG
GGTGGAGCCTTAGAGTAGGGTAAGATTAGGACTCTAGGTT

>104 Get primers
TTCTGGCTGATGGTGTCATAGGCTGGGAAGAGAGAGGCCAGGAGAAAAGGCTGAGGAAAC
TGCTGGCAAATGTGAAGGGCAAGAATGAATGCCCAAGGTGGGCAGCAGGTGAGGAAAGAG
TCCCTCACCTCCCTGGAGGAACAAGTCTTTGATTTGCTGAAAGGCATCCCGCACAGCCTG
GGCGCACTTGGGACTCTGGCCATAAAAGTCCTGGAGAAGAGACCAAGGTTGCTGCTGCCA
TTCTTGCACTGGCCTGGGGTACCCAAGTCCCCTCACTCACCGCTGTGACATCTCGGAAGA
ATTGGTAGGAGTCCCCAAGGCCTGCAACAGCTACAACAGGAGCGCTGGCTGCCAGTGCCC
CAGCCACCAGGTGGGGGTACTTCATCCTCATGTAGGCACTCAGCATCCCCCCATAACTGG
GAGTACAGAGCACAGATCATGGTTGTGGGAAGCTGCCCACAACTCAGGCGAGCAGCCTCA
CTGTCCTCCAGGCTGAGGTGCTAGGCTGCTCTTTCCCTGCTCAGAACGCCCAAGGGTGGG
AAAGAAGGACCTGAAACTGTCAGGCCCACACACCCTGATCCCAGGGCCAAGGCAGATACA
GCCTTCACTGGGAGAAGGCACCTGTGGGTGCCCTGCCCTGACCCAGCAATGAAGACATTG
CAGAGACAAAGTCAGAAGGAATTGTCCCACTAGTGGGAACAACATAGCATACACTGCCTA
TGAGGTCCACTCAAGGAGGGCTTCCAGAAGGAGGTAAAGCTAGACCCCGCCCTTCCACAT
GTGGGGTAGGCATAGGATGTTGAGACTGTAAGAGACATCTCTTTGGCCCTCCTTGTATAG
GGTGTCAATCGGCACAACAGGGTGGAGCCTTAGAGTAGGGTAAGATTAGGACTCTAGGTT
CTCTCATGGGTCCAGATCTGTCATGAAGGGAGGTCAAGGACCCACCTCCCTCCAAAGGCT
ATGGTGGGGGCATCATGGACCCCAAGGTCCTGCCGCAGGG

>114 Get primers
CCTGGAGCAGCACAGCAAAGTCGGCCAGCGCCTGCTCCACAGTCAGCAGCTGTGTATATC
CCCGCTGTGTGGACTGGACACCGAACGGAAGCGATTTCCCATAGTACCGCTGCAGAAAGC
AGGAAGGGATGGCTAATCCACTCCTCGGTGCTCCCCACCTCCTTCAACTCAGGGACTGCC
AGGAACTGTACAGGTACCCACGTGCTCAGCAAAGACAAGCAGGGCCTCCTGCTGGGCTGC
CAGTTCCACCATGAAGCCAGAGTTGTTAGCGAAGGACCAGATATCCCCCTCATTCCCTGT
GTAGAAAAAGATGGGCCCTTCGCCCATCTTCCAGAACTTATCTGTTGGAAGTAAATGAGT
TTCCATAAGGCCAGGGAAACGCAGGTAGGAACCCATGCGGTCGAGCCAGCACTCACCTGA
CACTAGGAACCGCTGGCCAAAGGTTTTGTTGCCGAAACTCTCAAAGTTGAAATGGTCCAT
GTATTGCTCAAAATAATTCTCATGAAAGTCAGGGTCTAGAACTCTGTCGGCTGAGGGCAG
GTGCAGAGACTCAGGAGCTGGTTGGGATCATCAGGGATCTAGGCGGGTCAGGAGGAAGGG
CAGCCAGTCTGTACTCACCTCTGGCCTGGAGGTTGCACAGTCCCAGTGACAGCAGCAGGA
CCAGGATCCAGGAGGGGACACCATGGTCCACAGGGTAACAAGGATGGAAGTTCATGCTTG
ATTCTGAGCCGGGCGCTGACTGTCATGTGATTTGGTCACATGACCGACACAACGGGCGGG
GCAGCATCACGTGATAGTCTGGCGGGGGCTGTCCTACTGTGGCTGGATTCTAGTTGGAGG
ATCAGCCTACTCTTCTTCAGTTTCCCGGTTCCTCCAAATTTCTGGGCTCCTACTTGTTTC
CACAGAGATGGATACTGTGGAGGTCCAGGAAGCAGAGAGATGGCTAAGGCTCATCAGGAC
CGTATGATCTCCCAAGTGTCCAGCTACTGAGTACCACAAG

>115 Get primers
ATAGTACCGCTGCAGAAAGCAGGAAGGGATGGCTAATCCACTCCTCGGTGCTCCCCACCT
CCTTCAACTCAGGGACTGCCAGGAACTGTACAGGTACCCACGTGCTCAGCAAAGACAAGC
AGGGCCTCCTGCTGGGCTGCCAGTTCCACCATGAAGCCAGAGTTGTTAGCGAAGGACCAG
ATATCCCCCTCATTCCCTGTGTAGAAAAAGATGGGCCCTTCGCCCATCTTCCAGAACTTA
TCTGTTGGAAGTAAATGAGTTTCCATAAGGCCAGGGAAACGCAGGTAGGAACCCATGCGG
TCGAGCCAGCACTCACCTGACACTAGGAACCGCTGGCCAAAGGTTTTGTTGCCGAAACTC
TCAAAGTTGAAATGGTCCATGTATTGCTCAAAATAATTCTCATGAAAGTCAGGGTCTAGA
ACTCTGTCGGCTGAGGGCAGGTGCAGAGACTCAGGAGCTGGTTGGGATCATCAGGGATCT
AGGCGGGTCAGGAGGAAGGGCAGCCAGTCTGTACTCACCTCTGGCCTGGAGGTTGCACAG
TCCCAGTGACAGCAGCAGGACCAGGATCCAGGAGGGGACACCATGGTCCACAGGGTAACA
AGGATGGAAGTTCATGCTTGATTCTGAGCCGGGCGCTGACTGTCATGTGATTTGGTCACA
TGACCGACACAACGGGCGGGGCAGCATCACGTGATAGTCTGGCGGGGGCTGTCCTACTGT
GGCTGGATTCTAGTTGGAGGATCAGCCTACTCTTCTTCAGTTTCCCGGTTCCTCCAAATT
TCTGGGCTCCTACTTGTTTCCACAGAGATGGATACTGTGGAGGTCCAGGAAGCAGAGAGA
TGGCTAAGGCTCATCAGGACCGTATGATCTCCCAAGTGTCCAGCTACTGAGTACCACAAG
GTGATGGGTGGGAGGGTCCTCCCACGGAAGGATACCGCAGTCCCTAGGGGTTGCAAGCCC
CACATGTTCCACTGGCTGCTAGAGCTACCTACTCAATCAG

>116 Get primers
CGTGCTCAGCAAAGACAAGCAGGGCCTCCTGCTGGGCTGCCAGTTCCACCATGAAGCCAG
AGTTGTTAGCGAAGGACCAGATATCCCCCTCATTCCCTGTGTAGAAAAAGATGGGCCCTT
CGCCCATCTTCCAGAACTTATCTGTTGGAAGTAAATGAGTTTCCATAAGGCCAGGGAAAC
GCAGGTAGGAACCCATGCGGTCGAGCCAGCACTCACCTGACACTAGGAACCGCTGGCCAA
AGGTTTTGTTGCCGAAACTCTCAAAGTTGAAATGGTCCATGTATTGCTCAAAATAATTCT
CATGAAAGTCAGGGTCTAGAACTCTGTCGGCTGAGGGCAGGTGCAGAGACTCAGGAGCTG
GTTGGGATCATCAGGGATCTAGGCGGGTCAGGAGGAAGGGCAGCCAGTCTGTACTCACCT
CTGGCCTGGAGGTTGCACAGTCCCAGTGACAGCAGCAGGACCAGGATCCAGGAGGGGACA
CCATGGTCCACAGGGTAACAAGGATGGAAGTTCATGCTTGATTCTGAGCCGGGCGCTGAC
TGTCATGTGATTTGGTCACATGACCGACACAACGGGCGGGGCAGCATCACGTGATAGTCT
GGCGGGGGCTGTCCTACTGTGGCTGGATTCTAGTTGGAGGATCAGCCTACTCTTCTTCAG
TTTCCCGGTTCCTCCAAATTTCTGGGCTCCTACTTGTTTCCACAGAGATGGATACTGTGG
AGGTCCAGGAAGCAGAGAGATGGCTAAGGCTCATCAGGACCGTATGATCTCCCAAGTGTC
CAGCTACTGAGTACCACAAGGTGATGGGTGGGAGGGTCCTCCCACGGAAGGATACCGCAG
TCCCTAGGGGTTGCAAGCCCCACATGTTCCACTGGCTGCTAGAGCTACCTACTCAATCAG
CCCTGGGCATCACCATCAGGTACTCGGCCAAAATGACCTCTCTGCTTCCAGTCCTCAGTT
CTGGTCAGCACCAGACAGGCCCATAATTACAGAGCCAGGG

>117 Get primers
GTAGAAAAAGATGGGCCCTTCGCCCATCTTCCAGAACTTATCTGTTGGAAGTAAATGAGT
TTCCATAAGGCCAGGGAAACGCAGGTAGGAACCCATGCGGTCGAGCCAGCACTCACCTGA
CACTAGGAACCGCTGGCCAAAGGTTTTGTTGCCGAAACTCTCAAAGTTGAAATGGTCCAT
GTATTGCTCAAAATAATTCTCATGAAAGTCAGGGTCTAGAACTCTGTCGGCTGAGGGCAG
GTGCAGAGACTCAGGAGCTGGTTGGGATCATCAGGGATCTAGGCGGGTCAGGAGGAAGGG
CAGCCAGTCTGTACTCACCTCTGGCCTGGAGGTTGCACAGTCCCAGTGACAGCAGCAGGA
CCAGGATCCAGGAGGGGACACCATGGTCCACAGGGTAACAAGGATGGAAGTTCATGCTTG
ATTCTGAGCCGGGCGCTGACTGTCATGTGATTTGGTCACATGACCGACACAACGGGCGGG
GCAGCATCACGTGATAGTCTGGCGGGGGCTGTCCTACTGTGGCTGGATTCTAGTTGGAGG
ATCAGCCTACTCTTCTTCAGTTTCCCGGTTCCTCCAAATTTCTGGGCTCCTACTTGTTTC
CACAGAGATGGATACTGTGGAGGTCCAGGAAGCAGAGAGATGGCTAAGGCTCATCAGGAC
CGTATGATCTCCCAAGTGTCCAGCTACTGAGTACCACAAGGTGATGGGTGGGAGGGTCCT
CCCACGGAAGGATACCGCAGTCCCTAGGGGTTGCAAGCCCCACATGTTCCACTGGCTGCT
AGAGCTACCTACTCAATCAGCCCTGGGCATCACCATCAGGTACTCGGCCAAAATGACCTC
TCTGCTTCCAGTCCTCAGTTCTGGTCAGCACCAGACAGGCCCATAATTACAGAGCCAGGG
AAACTGGAACATTTGTCTCCCCTTAGACAGTGGCAGCAGGAAGGTGGGGGGTTGTTGCAG
AGGAACAGTGTCTCTGAGAGAGGACCTTGGACTTTCTGGG

>118 Get primers
TCGAGCCAGCACTCACCTGACACTAGGAACCGCTGGCCAAAGGTTTTGTTGCCGAAACTC
TCAAAGTTGAAATGGTCCATGTATTGCTCAAAATAATTCTCATGAAAGTCAGGGTCTAGA
ACTCTGTCGGCTGAGGGCAGGTGCAGAGACTCAGGAGCTGGTTGGGATCATCAGGGATCT
AGGCGGGTCAGGAGGAAGGGCAGCCAGTCTGTACTCACCTCTGGCCTGGAGGTTGCACAG
TCCCAGTGACAGCAGCAGGACCAGGATCCAGGAGGGGACACCATGGTCCACAGGGTAACA
AGGATGGAAGTTCATGCTTGATTCTGAGCCGGGCGCTGACTGTCATGTGATTTGGTCACA
TGACCGACACAACGGGCGGGGCAGCATCACGTGATAGTCTGGCGGGGGCTGTCCTACTGT
GGCTGGATTCTAGTTGGAGGATCAGCCTACTCTTCTTCAGTTTCCCGGTTCCTCCAAATT
TCTGGGCTCCTACTTGTTTCCACAGAGATGGATACTGTGGAGGTCCAGGAAGCAGAGAGA
TGGCTAAGGCTCATCAGGACCGTATGATCTCCCAAGTGTCCAGCTACTGAGTACCACAAG
GTGATGGGTGGGAGGGTCCTCCCACGGAAGGATACCGCAGTCCCTAGGGGTTGCAAGCCC
CACATGTTCCACTGGCTGCTAGAGCTACCTACTCAATCAGCCCTGGGCATCACCATCAGG
TACTCGGCCAAAATGACCTCTCTGCTTCCAGTCCTCAGTTCTGGTCAGCACCAGACAGGC
CCATAATTACAGAGCCAGGGAAACTGGAACATTTGTCTCCCCTTAGACAGTGGCAGCAGG
AAGGTGGGGGGTTGTTGCAGAGGAACAGTGTCTCTGAGAGAGGACCTTGGACTTTCTGGG
AATCTCTGAGCTGCCCGGTTCTCCCCACTGCTGGCACTGTGCCCACAGCCCAAACAGAAT
GGGGGAGATGGAGGGGCAGGGCTTCTGTGGGAAGCTGCCC

>119 Get primers
CATGAAAGTCAGGGTCTAGAACTCTGTCGGCTGAGGGCAGGTGCAGAGACTCAGGAGCTG
GTTGGGATCATCAGGGATCTAGGCGGGTCAGGAGGAAGGGCAGCCAGTCTGTACTCACCT
CTGGCCTGGAGGTTGCACAGTCCCAGTGACAGCAGCAGGACCAGGATCCAGGAGGGGACA
CCATGGTCCACAGGGTAACAAGGATGGAAGTTCATGCTTGATTCTGAGCCGGGCGCTGAC
TGTCATGTGATTTGGTCACATGACCGACACAACGGGCGGGGCAGCATCACGTGATAGTCT
GGCGGGGGCTGTCCTACTGTGGCTGGATTCTAGTTGGAGGATCAGCCTACTCTTCTTCAG
TTTCCCGGTTCCTCCAAATTTCTGGGCTCCTACTTGTTTCCACAGAGATGGATACTGTGG
AGGTCCAGGAAGCAGAGAGATGGCTAAGGCTCATCAGGACCGTATGATCTCCCAAGTGTC
CAGCTACTGAGTACCACAAGGTGATGGGTGGGAGGGTCCTCCCACGGAAGGATACCGCAG
TCCCTAGGGGTTGCAAGCCCCACATGTTCCACTGGCTGCTAGAGCTACCTACTCAATCAG
CCCTGGGCATCACCATCAGGTACTCGGCCAAAATGACCTCTCTGCTTCCAGTCCTCAGTT
CTGGTCAGCACCAGACAGGCCCATAATTACAGAGCCAGGGAAACTGGAACATTTGTCTCC
CCTTAGACAGTGGCAGCAGGAAGGTGGGGGGTTGTTGCAGAGGAACAGTGTCTCTGAGAG
AGGACCTTGGACTTTCTGGGAATCTCTGAGCTGCCCGGTTCTCCCCACTGCTGGCACTGT
GCCCACAGCCCAAACAGAATGGGGGAGATGGAGGGGCAGGGCTTCTGTGGGAAGCTGCCC
TCCACCTCATTGGCACAGAGTGTCTCATTGCAGAGAGAAAAAAGGACCAGTTTTCTCTCT
GGCACCCAGGTCTGGAAGAGGAGTGACATCCACGGAAGTT

>120 Get primers
CAGCCAGTCTGTACTCACCTCTGGCCTGGAGGTTGCACAGTCCCAGTGACAGCAGCAGGA
CCAGGATCCAGGAGGGGACACCATGGTCCACAGGGTAACAAGGATGGAAGTTCATGCTTG
ATTCTGAGCCGGGCGCTGACTGTCATGTGATTTGGTCACATGACCGACACAACGGGCGGG
GCAGCATCACGTGATAGTCTGGCGGGGGCTGTCCTACTGTGGCTGGATTCTAGTTGGAGG
ATCAGCCTACTCTTCTTCAGTTTCCCGGTTCCTCCAAATTTCTGGGCTCCTACTTGTTTC
CACAGAGATGGATACTGTGGAGGTCCAGGAAGCAGAGAGATGGCTAAGGCTCATCAGGAC
CGTATGATCTCCCAAGTGTCCAGCTACTGAGTACCACAAGGTGATGGGTGGGAGGGTCCT
CCCACGGAAGGATACCGCAGTCCCTAGGGGTTGCAAGCCCCACATGTTCCACTGGCTGCT
AGAGCTACCTACTCAATCAGCCCTGGGCATCACCATCAGGTACTCGGCCAAAATGACCTC
TCTGCTTCCAGTCCTCAGTTCTGGTCAGCACCAGACAGGCCCATAATTACAGAGCCAGGG
AAACTGGAACATTTGTCTCCCCTTAGACAGTGGCAGCAGGAAGGTGGGGGGTTGTTGCAG
AGGAACAGTGTCTCTGAGAGAGGACCTTGGACTTTCTGGGAATCTCTGAGCTGCCCGGTT
CTCCCCACTGCTGGCACTGTGCCCACAGCCCAAACAGAATGGGGGAGATGGAGGGGCAGG
GCTTCTGTGGGAAGCTGCCCTCCACCTCATTGGCACAGAGTGTCTCATTGCAGAGAGAAA
AAAGGACCAGTTTTCTCTCTGGCACCCAGGTCTGGAAGAGGAGTGACATCCACGGAAGTT
GGTGACTTGGACTGGCTGGCCGTGAGTGGAACATGTCCATCCAGCATGGCCACAGTCCAG
TGGGACACACAGCCTAGAGCTGTGGAATGCCGTGCCACAG

>121 Get primers
AGGCCAAGAAGTAGACCCACCTGCCAGTGCCCGTAGACCCATGATCCTCTGGCCCTCATT
CAACAGCCGTTGACAGCCCACCTAGAGAAAGGCAGCAGAATATCTCAGTGGAGGCCCCTT
TCACAGAGCGTGGGTCAGGGCTGCTAGCTTCCAGGACACAACAGCAGATAGTGTCTGATG
GCATGAAAGCAGATAGCTACAAGGCTCTTGGACCAGGCTAGCACTGGGTCCTGCACCCAG
GGAGAGCCACCTCACCTTGACAGGGTTGGCAGGAAGGGGCCCTAGAAAGTCAGTAGGATA
CGGGTAGTCCATCATGGCGAGCACAGTAAATGCATTTCGGGCAAACCCAAAGAGCTGAGT
CAGGTCCTTTGGGCTGGAAAGTGATTGACAGGTACCAAAGTTCTGGCTGATGGTGTCATA
GGCTGGGAAGAGAGAGGCCAGGAGAAAAGGCTGAGGAAACTGCTGGCAAATGTGAAGGGC
AAGAATGAATGCCCAAGGTGGGCAGCAGGTGAGGAAAGAGTCCCTCACCTCCCTGGAGGA
ACAAGTCTTTGATTTGCTGAAAGGCATCCCGCACAGCCTGGGCGCACTTGGGACTCTGGC
CATAAAAGTCCTGGAGAAGAGACCAAGGTTGCTGCTGCCATTCTTGCACTGGCCTGGGGT
ACCCAAGTCCCCTCACTCACCGCTGTGACATCTCGGAAGAATTGGTAGGAGTCCCCAAGG
CCTGCAACAGCTACAACAGGAGCGCTGGCTGCCAGTGCCCCAGCCACCAGGTGGGGGTAC
TTCATCCTCATGTAGGCACTCAGCATCCCCCCATAACTGGGAGTACAGAGCACAGATCAT
GGTTGTGGGAAGCTGCCCACAACTCAGGCGAGCAGCCTCACTGTCCTCCAGGCTGAGGTG
CTAGGCTGCTCTTTCCCTGCTCAGAACGCCCAAGGGTGGGAAAGAAGGAC

>122 Get primers
CAGAATATCTCAGTGGAGGCCCCTTTCACAGAGCGTGGGTCAGGGCTGCTAGCTTCCAGG
ACACAACAGCAGATAGTGTCTGATGGCATGAAAGCAGATAGCTACAAGGCTCTTGGACCA
GGCTAGCACTGGGTCCTGCACCCAGGGAGAGCCACCTCACCTTGACAGGGTTGGCAGGAA
GGGGCCCTAGAAAGTCAGTAGGATACGGGTAGTCCATCATGGCGAGCACAGTAAATGCAT
TTCGGGCAAACCCAAAGAGCTGAGTCAGGTCCTTTGGGCTGGAAAGTGATTGACAGGTAC
CAAAGTTCTGGCTGATGGTGTCATAGGCTGGGAAGAGAGAGGCCAGGAGAAAAGGCTGAG
GAAACTGCTGGCAAATGTGAAGGGCAAGAATGAATGCCCAAGGTGGGCAGCAGGTGAGGA
AAGAGTCCCTCACCTCCCTGGAGGAACAAGTCTTTGATTTGCTGAAAGGCATCCCGCACA
GCCTGGGCGCACTTGGGACTCTGGCCATAAAAGTCCTGGAGAAGAGACCAAGGTTGCTGC
TGCCATTCTTGCACTGGCCTGGGGTACCCAAGTCCCCTCACTCACCGCTGTGACATCTCG
GAAGAATTGGTAGGAGTCCCCAAGGCCTGCAACAGCTACAACAGGAGCGCTGGCTGCCAG
TGCCCCAGCCACCAGGTGGGGGTACTTCATCCTCATGTAGGCACTCAGCATCCCCCCATA
ACTGGGAGTACAGAGCACAGATCATGGTTGTGGGAAGCTGCCCACAACTCAGGCGAGCAG
CCTCACTGTCCTCCAGGCTGAGGTGCTAGGCTGCTCTTTCCCTGCTCAGAACGCCCAAGG
GTGGGAAAGAAGGACCTGAAACTGTCAGGCCCACACACCCTGATCCCAGGGCCAAGGCAG
ATACAGCCTTCACTGGGAGAAGGCACCTGTGGGTGCCCTGCCCTGACCCA

>123 Get primers
AGATAGCTACAAGGCTCTTGGACCAGGCTAGCACTGGGTCCTGCACCCAGGGAGAGCCAC
CTCACCTTGACAGGGTTGGCAGGAAGGGGCCCTAGAAAGTCAGTAGGATACGGGTAGTCC
ATCATGGCGAGCACAGTAAATGCATTTCGGGCAAACCCAAAGAGCTGAGTCAGGTCCTTT
GGGCTGGAAAGTGATTGACAGGTACCAAAGTTCTGGCTGATGGTGTCATAGGCTGGGAAG
AGAGAGGCCAGGAGAAAAGGCTGAGGAAACTGCTGGCAAATGTGAAGGGCAAGAATGAAT
GCCCAAGGTGGGCAGCAGGTGAGGAAAGAGTCCCTCACCTCCCTGGAGGAACAAGTCTTT
GATTTGCTGAAAGGCATCCCGCACAGCCTGGGCGCACTTGGGACTCTGGCCATAAAAGTC
CTGGAGAAGAGACCAAGGTTGCTGCTGCCATTCTTGCACTGGCCTGGGGTACCCAAGTCC
CCTCACTCACCGCTGTGACATCTCGGAAGAATTGGTAGGAGTCCCCAAGGCCTGCAACAG
CTACAACAGGAGCGCTGGCTGCCAGTGCCCCAGCCACCAGGTGGGGGTACTTCATCCTCA
TGTAGGCACTCAGCATCCCCCCATAACTGGGAGTACAGAGCACAGATCATGGTTGTGGGA
AGCTGCCCACAACTCAGGCGAGCAGCCTCACTGTCCTCCAGGCTGAGGTGCTAGGCTGCT
CTTTCCCTGCTCAGAACGCCCAAGGGTGGGAAAGAAGGACCTGAAACTGTCAGGCCCACA
CACCCTGATCCCAGGGCCAAGGCAGATACAGCCTTCACTGGGAGAAGGCACCTGTGGGTG
CCCTGCCCTGACCCAGCAATGAAGACATTGCAGAGACAAAGTCAGAAGGAATTGTCCCAC
TAGTGGGAACAACATAGCATACACTGCCTATGAGGTCCACTCAAGGAGGG

>124 Get primers
AAAGTCAGTAGGATACGGGTAGTCCATCATGGCGAGCACAGTAAATGCATTTCGGGCAAA
CCCAAAGAGCTGAGTCAGGTCCTTTGGGCTGGAAAGTGATTGACAGGTACCAAAGTTCTG
GCTGATGGTGTCATAGGCTGGGAAGAGAGAGGCCAGGAGAAAAGGCTGAGGAAACTGCTG
GCAAATGTGAAGGGCAAGAATGAATGCCCAAGGTGGGCAGCAGGTGAGGAAAGAGTCCCT
CACCTCCCTGGAGGAACAAGTCTTTGATTTGCTGAAAGGCATCCCGCACAGCCTGGGCGC
ACTTGGGACTCTGGCCATAAAAGTCCTGGAGAAGAGACCAAGGTTGCTGCTGCCATTCTT
GCACTGGCCTGGGGTACCCAAGTCCCCTCACTCACCGCTGTGACATCTCGGAAGAATTGG
TAGGAGTCCCCAAGGCCTGCAACAGCTACAACAGGAGCGCTGGCTGCCAGTGCCCCAGCC
ACCAGGTGGGGGTACTTCATCCTCATGTAGGCACTCAGCATCCCCCCATAACTGGGAGTA
CAGAGCACAGATCATGGTTGTGGGAAGCTGCCCACAACTCAGGCGAGCAGCCTCACTGTC
CTCCAGGCTGAGGTGCTAGGCTGCTCTTTCCCTGCTCAGAACGCCCAAGGGTGGGAAAGA
AGGACCTGAAACTGTCAGGCCCACACACCCTGATCCCAGGGCCAAGGCAGATACAGCCTT
CACTGGGAGAAGGCACCTGTGGGTGCCCTGCCCTGACCCAGCAATGAAGACATTGCAGAG
ACAAAGTCAGAAGGAATTGTCCCACTAGTGGGAACAACATAGCATACACTGCCTATGAGG
TCCACTCAAGGAGGGCTTCCAGAAGGAGGTAAAGCTAGACCCCGCCCTTCCACATGTGGG
GTAGGCATAGGATGTTGAGACTGTAAGAGACATCTCTTTGGCCCTCCTTG

>125 Get primers
GTGATTGACAGGTACCAAAGTTCTGGCTGATGGTGTCATAGGCTGGGAAGAGAGAGGCCA
GGAGAAAAGGCTGAGGAAACTGCTGGCAAATGTGAAGGGCAAGAATGAATGCCCAAGGTG
GGCAGCAGGTGAGGAAAGAGTCCCTCACCTCCCTGGAGGAACAAGTCTTTGATTTGCTGA
AAGGCATCCCGCACAGCCTGGGCGCACTTGGGACTCTGGCCATAAAAGTCCTGGAGAAGA
GACCAAGGTTGCTGCTGCCATTCTTGCACTGGCCTGGGGTACCCAAGTCCCCTCACTCAC
CGCTGTGACATCTCGGAAGAATTGGTAGGAGTCCCCAAGGCCTGCAACAGCTACAACAGG
AGCGCTGGCTGCCAGTGCCCCAGCCACCAGGTGGGGGTACTTCATCCTCATGTAGGCACT
CAGCATCCCCCCATAACTGGGAGTACAGAGCACAGATCATGGTTGTGGGAAGCTGCCCAC
AACTCAGGCGAGCAGCCTCACTGTCCTCCAGGCTGAGGTGCTAGGCTGCTCTTTCCCTGC
TCAGAACGCCCAAGGGTGGGAAAGAAGGACCTGAAACTGTCAGGCCCACACACCCTGATC
CCAGGGCCAAGGCAGATACAGCCTTCACTGGGAGAAGGCACCTGTGGGTGCCCTGCCCTG
ACCCAGCAATGAAGACATTGCAGAGACAAAGTCAGAAGGAATTGTCCCACTAGTGGGAAC
AACATAGCATACACTGCCTATGAGGTCCACTCAAGGAGGGCTTCCAGAAGGAGGTAAAGC
TAGACCCCGCCCTTCCACATGTGGGGTAGGCATAGGATGTTGAGACTGTAAGAGACATCT
CTTTGGCCCTCCTTGTATAGGGTGTCAATCGGCACAACAGGGTGGAGCCTTAGAGTAGGG
TAAGATTAGGACTCTAGGTTCTCTCATGGGTCCAGATCTGTCATGAAGGG

>126 Get primers
AGGGCAAGAATGAATGCCCAAGGTGGGCAGCAGGTGAGGAAAGAGTCCCTCACCTCCCTG
GAGGAACAAGTCTTTGATTTGCTGAAAGGCATCCCGCACAGCCTGGGCGCACTTGGGACT
CTGGCCATAAAAGTCCTGGAGAAGAGACCAAGGTTGCTGCTGCCATTCTTGCACTGGCCT
GGGGTACCCAAGTCCCCTCACTCACCGCTGTGACATCTCGGAAGAATTGGTAGGAGTCCC
CAAGGCCTGCAACAGCTACAACAGGAGCGCTGGCTGCCAGTGCCCCAGCCACCAGGTGGG
GGTACTTCATCCTCATGTAGGCACTCAGCATCCCCCCATAACTGGGAGTACAGAGCACAG
ATCATGGTTGTGGGAAGCTGCCCACAACTCAGGCGAGCAGCCTCACTGTCCTCCAGGCTG
AGGTGCTAGGCTGCTCTTTCCCTGCTCAGAACGCCCAAGGGTGGGAAAGAAGGACCTGAA
ACTGTCAGGCCCACACACCCTGATCCCAGGGCCAAGGCAGATACAGCCTTCACTGGGAGA
AGGCACCTGTGGGTGCCCTGCCCTGACCCAGCAATGAAGACATTGCAGAGACAAAGTCAG
AAGGAATTGTCCCACTAGTGGGAACAACATAGCATACACTGCCTATGAGGTCCACTCAAG
GAGGGCTTCCAGAAGGAGGTAAAGCTAGACCCCGCCCTTCCACATGTGGGGTAGGCATAG
GATGTTGAGACTGTAAGAGACATCTCTTTGGCCCTCCTTGTATAGGGTGTCAATCGGCAC
AACAGGGTGGAGCCTTAGAGTAGGGTAAGATTAGGACTCTAGGTTCTCTCATGGGTCCAG
ATCTGTCATGAAGGGAGGTCAAGGACCCACCTCCCTCCAAAGGCTATGGTGGGGGCATCA
TGGACCCCAAGGTCCTGCCGCAGGGCCTGGAGCAGCACAGCAAAGTCGGC

>136 Get primers
CAGCGCCTGCTCCACAGTCAGCAGCTGTGTATATCCCCGCTGTGTGGACTGGACACCGAA
CGGAAGCGATTTCCCATAGTACCGCTGCAGAAAGCAGGAAGGGATGGCTAATCCACTCCT
CGGTGCTCCCCACCTCCTTCAACTCAGGGACTGCCAGGAACTGTACAGGTACCCACGTGC
TCAGCAAAGACAAGCAGGGCCTCCTGCTGGGCTGCCAGTTCCACCATGAAGCCAGAGTTG
TTAGCGAAGGACCAGATATCCCCCTCATTCCCTGTGTAGAAAAAGATGGGCCCTTCGCCC
ATCTTCCAGAACTTATCTGTTGGAAGTAAATGAGTTTCCATAAGGCCAGGGAAACGCAGG
TAGGAACCCATGCGGTCGAGCCAGCACTCACCTGACACTAGGAACCGCTGGCCAAAGGTT
TTGTTGCCGAAACTCTCAAAGTTGAAATGGTCCATGTATTGCTCAAAATAATTCTCATGA
AAGTCAGGGTCTAGAACTCTGTCGGCTGAGGGCAGGTGCAGAGACTCAGGAGCTGGTTGG
GATCATCAGGGATCTAGGCGGGTCAGGAGGAAGGGCAGCCAGTCTGTACTCACCTCTGGC
CTGGAGGTTGCACAGTCCCAGTGACAGCAGCAGGACCAGGATCCAGGAGGGGACACCATG
GTCCACAGGGTAACAAGGATGGAAGTTCATGCTTGATTCTGAGCCGGGCGCTGACTGTCA
TGTGATTTGGTCACATGACCGACACAACGGGCGGGGCAGCATCACGTGATAGTCTGGCGG
GGGCTGTCCTACTGTGGCTGGATTCTAGTTGGAGGATCAGCCTACTCTTCTTCAGTTTCC
CGGTTCCTCCAAATTTCTGGGCTCCTACTTGTTTCCACAGAGATGGATACTGTGGAGGTC
CAGGAAGCAGAGAGATGGCTAAGGCTCATCAGGACCGTATGATCTCCCAA

>137 Get primers
AGGAAGGGATGGCTAATCCACTCCTCGGTGCTCCCCACCTCCTTCAACTCAGGGACTGCC
AGGAACTGTACAGGTACCCACGTGCTCAGCAAAGACAAGCAGGGCCTCCTGCTGGGCTGC
CAGTTCCACCATGAAGCCAGAGTTGTTAGCGAAGGACCAGATATCCCCCTCATTCCCTGT
GTAGAAAAAGATGGGCCCTTCGCCCATCTTCCAGAACTTATCTGTTGGAAGTAAATGAGT
TTCCATAAGGCCAGGGAAACGCAGGTAGGAACCCATGCGGTCGAGCCAGCACTCACCTGA
CACTAGGAACCGCTGGCCAAAGGTTTTGTTGCCGAAACTCTCAAAGTTGAAATGGTCCAT
GTATTGCTCAAAATAATTCTCATGAAAGTCAGGGTCTAGAACTCTGTCGGCTGAGGGCAG
GTGCAGAGACTCAGGAGCTGGTTGGGATCATCAGGGATCTAGGCGGGTCAGGAGGAAGGG
CAGCCAGTCTGTACTCACCTCTGGCCTGGAGGTTGCACAGTCCCAGTGACAGCAGCAGGA
CCAGGATCCAGGAGGGGACACCATGGTCCACAGGGTAACAAGGATGGAAGTTCATGCTTG
ATTCTGAGCCGGGCGCTGACTGTCATGTGATTTGGTCACATGACCGACACAACGGGCGGG
GCAGCATCACGTGATAGTCTGGCGGGGGCTGTCCTACTGTGGCTGGATTCTAGTTGGAGG
ATCAGCCTACTCTTCTTCAGTTTCCCGGTTCCTCCAAATTTCTGGGCTCCTACTTGTTTC
CACAGAGATGGATACTGTGGAGGTCCAGGAAGCAGAGAGATGGCTAAGGCTCATCAGGAC
CGTATGATCTCCCAAGTGTCCAGCTACTGAGTACCACAAGGTGATGGGTGGGAGGGTCCT
CCCACGGAAGGATACCGCAGTCCCTAGGGGTTGCAAGCCCCACATGTTCC

>138 Get primers
CAAGCAGGGCCTCCTGCTGGGCTGCCAGTTCCACCATGAAGCCAGAGTTGTTAGCGAAGG
ACCAGATATCCCCCTCATTCCCTGTGTAGAAAAAGATGGGCCCTTCGCCCATCTTCCAGA
ACTTATCTGTTGGAAGTAAATGAGTTTCCATAAGGCCAGGGAAACGCAGGTAGGAACCCA
TGCGGTCGAGCCAGCACTCACCTGACACTAGGAACCGCTGGCCAAAGGTTTTGTTGCCGA
AACTCTCAAAGTTGAAATGGTCCATGTATTGCTCAAAATAATTCTCATGAAAGTCAGGGT
CTAGAACTCTGTCGGCTGAGGGCAGGTGCAGAGACTCAGGAGCTGGTTGGGATCATCAGG
GATCTAGGCGGGTCAGGAGGAAGGGCAGCCAGTCTGTACTCACCTCTGGCCTGGAGGTTG
CACAGTCCCAGTGACAGCAGCAGGACCAGGATCCAGGAGGGGACACCATGGTCCACAGGG
TAACAAGGATGGAAGTTCATGCTTGATTCTGAGCCGGGCGCTGACTGTCATGTGATTTGG
TCACATGACCGACACAACGGGCGGGGCAGCATCACGTGATAGTCTGGCGGGGGCTGTCCT
ACTGTGGCTGGATTCTAGTTGGAGGATCAGCCTACTCTTCTTCAGTTTCCCGGTTCCTCC
AAATTTCTGGGCTCCTACTTGTTTCCACAGAGATGGATACTGTGGAGGTCCAGGAAGCAG
AGAGATGGCTAAGGCTCATCAGGACCGTATGATCTCCCAAGTGTCCAGCTACTGAGTACC
ACAAGGTGATGGGTGGGAGGGTCCTCCCACGGAAGGATACCGCAGTCCCTAGGGGTTGCA
AGCCCCACATGTTCCACTGGCTGCTAGAGCTACCTACTCAATCAGCCCTGGGCATCACCA
TCAGGTACTCGGCCAAAATGACCTCTCTGCTTCCAGTCCTCAGTTCTGGT

>139 Get primers
ATGGGCCCTTCGCCCATCTTCCAGAACTTATCTGTTGGAAGTAAATGAGTTTCCATAAGG
CCAGGGAAACGCAGGTAGGAACCCATGCGGTCGAGCCAGCACTCACCTGACACTAGGAAC
CGCTGGCCAAAGGTTTTGTTGCCGAAACTCTCAAAGTTGAAATGGTCCATGTATTGCTCA
AAATAATTCTCATGAAAGTCAGGGTCTAGAACTCTGTCGGCTGAGGGCAGGTGCAGAGAC
TCAGGAGCTGGTTGGGATCATCAGGGATCTAGGCGGGTCAGGAGGAAGGGCAGCCAGTCT
GTACTCACCTCTGGCCTGGAGGTTGCACAGTCCCAGTGACAGCAGCAGGACCAGGATCCA
GGAGGGGACACCATGGTCCACAGGGTAACAAGGATGGAAGTTCATGCTTGATTCTGAGCC
GGGCGCTGACTGTCATGTGATTTGGTCACATGACCGACACAACGGGCGGGGCAGCATCAC
GTGATAGTCTGGCGGGGGCTGTCCTACTGTGGCTGGATTCTAGTTGGAGGATCAGCCTAC
TCTTCTTCAGTTTCCCGGTTCCTCCAAATTTCTGGGCTCCTACTTGTTTCCACAGAGATG
GATACTGTGGAGGTCCAGGAAGCAGAGAGATGGCTAAGGCTCATCAGGACCGTATGATCT
CCCAAGTGTCCAGCTACTGAGTACCACAAGGTGATGGGTGGGAGGGTCCTCCCACGGAAG
GATACCGCAGTCCCTAGGGGTTGCAAGCCCCACATGTTCCACTGGCTGCTAGAGCTACCT
ACTCAATCAGCCCTGGGCATCACCATCAGGTACTCGGCCAAAATGACCTCTCTGCTTCCA
GTCCTCAGTTCTGGTCAGCACCAGACAGGCCCATAATTACAGAGCCAGGGAAACTGGAAC
ATTTGTCTCCCCTTAGACAGTGGCAGCAGGAAGGTGGGGGGTTGTTGCAG

>140 Get primers
CCAGCACTCACCTGACACTAGGAACCGCTGGCCAAAGGTTTTGTTGCCGAAACTCTCAAA
GTTGAAATGGTCCATGTATTGCTCAAAATAATTCTCATGAAAGTCAGGGTCTAGAACTCT
GTCGGCTGAGGGCAGGTGCAGAGACTCAGGAGCTGGTTGGGATCATCAGGGATCTAGGCG
GGTCAGGAGGAAGGGCAGCCAGTCTGTACTCACCTCTGGCCTGGAGGTTGCACAGTCCCA
GTGACAGCAGCAGGACCAGGATCCAGGAGGGGACACCATGGTCCACAGGGTAACAAGGAT
GGAAGTTCATGCTTGATTCTGAGCCGGGCGCTGACTGTCATGTGATTTGGTCACATGACC
GACACAACGGGCGGGGCAGCATCACGTGATAGTCTGGCGGGGGCTGTCCTACTGTGGCTG
GATTCTAGTTGGAGGATCAGCCTACTCTTCTTCAGTTTCCCGGTTCCTCCAAATTTCTGG
GCTCCTACTTGTTTCCACAGAGATGGATACTGTGGAGGTCCAGGAAGCAGAGAGATGGCT
AAGGCTCATCAGGACCGTATGATCTCCCAAGTGTCCAGCTACTGAGTACCACAAGGTGAT
GGGTGGGAGGGTCCTCCCACGGAAGGATACCGCAGTCCCTAGGGGTTGCAAGCCCCACAT
GTTCCACTGGCTGCTAGAGCTACCTACTCAATCAGCCCTGGGCATCACCATCAGGTACTC
GGCCAAAATGACCTCTCTGCTTCCAGTCCTCAGTTCTGGTCAGCACCAGACAGGCCCATA
ATTACAGAGCCAGGGAAACTGGAACATTTGTCTCCCCTTAGACAGTGGCAGCAGGAAGGT
GGGGGGTTGTTGCAGAGGAACAGTGTCTCTGAGAGAGGACCTTGGACTTTCTGGGAATCT
CTGAGCTGCCCGGTTCTCCCCACTGCTGGCACTGTGCCCACAGCCCAAAC

>141 Get primers
CATGAAAGTCAGGGTCTAGAACTCTGTCGGCTGAGGGCAGGTGCAGAGACTCAGGAGCTG
GTTGGGATCATCAGGGATCTAGGCGGGTCAGGAGGAAGGGCAGCCAGTCTGTACTCACCT
CTGGCCTGGAGGTTGCACAGTCCCAGTGACAGCAGCAGGACCAGGATCCAGGAGGGGACA
CCATGGTCCACAGGGTAACAAGGATGGAAGTTCATGCTTGATTCTGAGCCGGGCGCTGAC
TGTCATGTGATTTGGTCACATGACCGACACAACGGGCGGGGCAGCATCACGTGATAGTCT
GGCGGGGGCTGTCCTACTGTGGCTGGATTCTAGTTGGAGGATCAGCCTACTCTTCTTCAG
TTTCCCGGTTCCTCCAAATTTCTGGGCTCCTACTTGTTTCCACAGAGATGGATACTGTGG
AGGTCCAGGAAGCAGAGAGATGGCTAAGGCTCATCAGGACCGTATGATCTCCCAAGTGTC
CAGCTACTGAGTACCACAAGGTGATGGGTGGGAGGGTCCTCCCACGGAAGGATACCGCAG
TCCCTAGGGGTTGCAAGCCCCACATGTTCCACTGGCTGCTAGAGCTACCTACTCAATCAG
CCCTGGGCATCACCATCAGGTACTCGGCCAAAATGACCTCTCTGCTTCCAGTCCTCAGTT
CTGGTCAGCACCAGACAGGCCCATAATTACAGAGCCAGGGAAACTGGAACATTTGTCTCC
CCTTAGACAGTGGCAGCAGGAAGGTGGGGGGTTGTTGCAGAGGAACAGTGTCTCTGAGAG
AGGACCTTGGACTTTCTGGGAATCTCTGAGCTGCCCGGTTCTCCCCACTGCTGGCACTGT
GCCCACAGCCCAAACAGAATGGGGGAGATGGAGGGGCAGGGCTTCTGTGGGAAGCTGCCC
TCCACCTCATTGGCACAGAGTGTCTCATTGCAGAGAGAAAAAAGGACCAG

>142 Get primers
AAGGGCAGCCAGTCTGTACTCACCTCTGGCCTGGAGGTTGCACAGTCCCAGTGACAGCAG
CAGGACCAGGATCCAGGAGGGGACACCATGGTCCACAGGGTAACAAGGATGGAAGTTCAT
GCTTGATTCTGAGCCGGGCGCTGACTGTCATGTGATTTGGTCACATGACCGACACAACGG
GCGGGGCAGCATCACGTGATAGTCTGGCGGGGGCTGTCCTACTGTGGCTGGATTCTAGTT
GGAGGATCAGCCTACTCTTCTTCAGTTTCCCGGTTCCTCCAAATTTCTGGGCTCCTACTT
GTTTCCACAGAGATGGATACTGTGGAGGTCCAGGAAGCAGAGAGATGGCTAAGGCTCATC
AGGACCGTATGATCTCCCAAGTGTCCAGCTACTGAGTACCACAAGGTGATGGGTGGGAGG
GTCCTCCCACGGAAGGATACCGCAGTCCCTAGGGGTTGCAAGCCCCACATGTTCCACTGG
CTGCTAGAGCTACCTACTCAATCAGCCCTGGGCATCACCATCAGGTACTCGGCCAAAATG
ACCTCTCTGCTTCCAGTCCTCAGTTCTGGTCAGCACCAGACAGGCCCATAATTACAGAGC
CAGGGAAACTGGAACATTTGTCTCCCCTTAGACAGTGGCAGCAGGAAGGTGGGGGGTTGT
TGCAGAGGAACAGTGTCTCTGAGAGAGGACCTTGGACTTTCTGGGAATCTCTGAGCTGCC
CGGTTCTCCCCACTGCTGGCACTGTGCCCACAGCCCAAACAGAATGGGGGAGATGGAGGG
GCAGGGCTTCTGTGGGAAGCTGCCCTCCACCTCATTGGCACAGAGTGTCTCATTGCAGAG
AGAAAAAAGGACCAGTTTTCTCTCTGGCACCCAGGTCTGGAAGAGGAGTGACATCCACGG
AAGTTGGTGACTTGGACTGGCTGGCCGTGAGTGGAACATGTCCATCCAGC

>143 Get primers
AGGCCAAGAAGTAGACCCACCTGCCAGTGCCCGTAGACCCATGATCCTCTGGCCCTCATT
CAACAGCCGTTGACAGCCCACCTAGAGAAAGGCAGCAGAATATCTCAGTGGAGGCCCCTT
TCACAGAGCGTGGGTCAGGGCTGCTAGCTTCCAGGACACAACAGCAGATAGTGTCTGATG
GCATGAAAGCAGATAGCTACAAGGCTCTTGGACCAGGCTAGCACTGGGTCCTGCACCCAG
GGAGAGCCACCTCACCTTGACAGGGTTGGCAGGAAGGGGCCCTAGAAAGTCAGTAGGATA
CGGGTAGTCCATCATGGCGAGCACAGTAAATGCATTTCGGGCAAACCCAAAGAGCTGAGT
CAGGTCCTTTGGGCTGGAAAGTGATTGACAGGTACCAAAGTTCTGGCTGATGGTGTCATA
GGCTGGGAAGAGAGAGGCCAGGAGAAAAGGCTGAGGAAACTGCTGGCAAATGTGAAGGGC
AAGAATGAATGCCCAAGGTGGGCAGCAGGTGAGGAAAGAGTCCCTCACCTCCCTGGAGGA
ACAAGTCTTTGATTTGCTGAAAGGCATCCCGCACAGCCTGGGCGCACTTGGGACTCTGGC
CATAAAAGTCCTGGAGAAGAGACCAAGGTTGCTGCTGCCATTCTTGCACTGGCCTGGGGT
ACCCAAGTCCCCTCACTCACCGCTGTGACATCTCGGAAGAATTGGTAGGAGTCCCCAAGG
CCTGCAACAGCTACAACAGGAGCGCTGGCTGCCAGTGCCCCAGCCACCAGGTGGGGGTAC
TTCATCCTCATGTAGGCACTCAGCATCCCCCCATAACTGGGAGTACAGAGCACAGATCAT
GGTTGTGGGAAGCTGCCCACAACTCAGGCGAGCAGCCTCACTGTCCTCCAGGCTGAGGTG

>144 Get primers
GGCAGCAGAATATCTCAGTGGAGGCCCCTTTCACAGAGCGTGGGTCAGGGCTGCTAGCTT
CCAGGACACAACAGCAGATAGTGTCTGATGGCATGAAAGCAGATAGCTACAAGGCTCTTG
GACCAGGCTAGCACTGGGTCCTGCACCCAGGGAGAGCCACCTCACCTTGACAGGGTTGGC
AGGAAGGGGCCCTAGAAAGTCAGTAGGATACGGGTAGTCCATCATGGCGAGCACAGTAAA
TGCATTTCGGGCAAACCCAAAGAGCTGAGTCAGGTCCTTTGGGCTGGAAAGTGATTGACA
GGTACCAAAGTTCTGGCTGATGGTGTCATAGGCTGGGAAGAGAGAGGCCAGGAGAAAAGG
CTGAGGAAACTGCTGGCAAATGTGAAGGGCAAGAATGAATGCCCAAGGTGGGCAGCAGGT
GAGGAAAGAGTCCCTCACCTCCCTGGAGGAACAAGTCTTTGATTTGCTGAAAGGCATCCC
GCACAGCCTGGGCGCACTTGGGACTCTGGCCATAAAAGTCCTGGAGAAGAGACCAAGGTT
GCTGCTGCCATTCTTGCACTGGCCTGGGGTACCCAAGTCCCCTCACTCACCGCTGTGACA
TCTCGGAAGAATTGGTAGGAGTCCCCAAGGCCTGCAACAGCTACAACAGGAGCGCTGGCT
GCCAGTGCCCCAGCCACCAGGTGGGGGTACTTCATCCTCATGTAGGCACTCAGCATCCCC
CCATAACTGGGAGTACAGAGCACAGATCATGGTTGTGGGAAGCTGCCCACAACTCAGGCG
AGCAGCCTCACTGTCCTCCAGGCTGAGGTGCTAGGCTGCTCTTTCCCTGCTCAGAACGCC
CAAGGGTGGGAAAGAAGGACCTGAAACTGTCAGGCCCACACACCCTGATCCCAGGGCCAA

>145 Get primers
GCATGAAAGCAGATAGCTACAAGGCTCTTGGACCAGGCTAGCACTGGGTCCTGCACCCAG
GGAGAGCCACCTCACCTTGACAGGGTTGGCAGGAAGGGGCCCTAGAAAGTCAGTAGGATA
CGGGTAGTCCATCATGGCGAGCACAGTAAATGCATTTCGGGCAAACCCAAAGAGCTGAGT
CAGGTCCTTTGGGCTGGAAAGTGATTGACAGGTACCAAAGTTCTGGCTGATGGTGTCATA
GGCTGGGAAGAGAGAGGCCAGGAGAAAAGGCTGAGGAAACTGCTGGCAAATGTGAAGGGC
AAGAATGAATGCCCAAGGTGGGCAGCAGGTGAGGAAAGAGTCCCTCACCTCCCTGGAGGA
ACAAGTCTTTGATTTGCTGAAAGGCATCCCGCACAGCCTGGGCGCACTTGGGACTCTGGC
CATAAAAGTCCTGGAGAAGAGACCAAGGTTGCTGCTGCCATTCTTGCACTGGCCTGGGGT
ACCCAAGTCCCCTCACTCACCGCTGTGACATCTCGGAAGAATTGGTAGGAGTCCCCAAGG
CCTGCAACAGCTACAACAGGAGCGCTGGCTGCCAGTGCCCCAGCCACCAGGTGGGGGTAC
TTCATCCTCATGTAGGCACTCAGCATCCCCCCATAACTGGGAGTACAGAGCACAGATCAT
GGTTGTGGGAAGCTGCCCACAACTCAGGCGAGCAGCCTCACTGTCCTCCAGGCTGAGGTG
CTAGGCTGCTCTTTCCCTGCTCAGAACGCCCAAGGGTGGGAAAGAAGGACCTGAAACTGT
CAGGCCCACACACCCTGATCCCAGGGCCAAGGCAGATACAGCCTTCACTGGGAGAAGGCA
CCTGTGGGTGCCCTGCCCTGACCCAGCAATGAAGACATTGCAGAGACAAAGTCAGAAGGA

>146 Get primers
AGGAAGGGGCCCTAGAAAGTCAGTAGGATACGGGTAGTCCATCATGGCGAGCACAGTAAA
TGCATTTCGGGCAAACCCAAAGAGCTGAGTCAGGTCCTTTGGGCTGGAAAGTGATTGACA
GGTACCAAAGTTCTGGCTGATGGTGTCATAGGCTGGGAAGAGAGAGGCCAGGAGAAAAGG
CTGAGGAAACTGCTGGCAAATGTGAAGGGCAAGAATGAATGCCCAAGGTGGGCAGCAGGT
GAGGAAAGAGTCCCTCACCTCCCTGGAGGAACAAGTCTTTGATTTGCTGAAAGGCATCCC
GCACAGCCTGGGCGCACTTGGGACTCTGGCCATAAAAGTCCTGGAGAAGAGACCAAGGTT
GCTGCTGCCATTCTTGCACTGGCCTGGGGTACCCAAGTCCCCTCACTCACCGCTGTGACA
TCTCGGAAGAATTGGTAGGAGTCCCCAAGGCCTGCAACAGCTACAACAGGAGCGCTGGCT
GCCAGTGCCCCAGCCACCAGGTGGGGGTACTTCATCCTCATGTAGGCACTCAGCATCCCC
CCATAACTGGGAGTACAGAGCACAGATCATGGTTGTGGGAAGCTGCCCACAACTCAGGCG
AGCAGCCTCACTGTCCTCCAGGCTGAGGTGCTAGGCTGCTCTTTCCCTGCTCAGAACGCC
CAAGGGTGGGAAAGAAGGACCTGAAACTGTCAGGCCCACACACCCTGATCCCAGGGCCAA
GGCAGATACAGCCTTCACTGGGAGAAGGCACCTGTGGGTGCCCTGCCCTGACCCAGCAAT
GAAGACATTGCAGAGACAAAGTCAGAAGGAATTGTCCCACTAGTGGGAACAACATAGCAT
ACACTGCCTATGAGGTCCACTCAAGGAGGGCTTCCAGAAGGAGGTAAAGCTAGACCCCGC

>147 Get primers
CAGGTCCTTTGGGCTGGAAAGTGATTGACAGGTACCAAAGTTCTGGCTGATGGTGTCATA
GGCTGGGAAGAGAGAGGCCAGGAGAAAAGGCTGAGGAAACTGCTGGCAAATGTGAAGGGC
AAGAATGAATGCCCAAGGTGGGCAGCAGGTGAGGAAAGAGTCCCTCACCTCCCTGGAGGA
ACAAGTCTTTGATTTGCTGAAAGGCATCCCGCACAGCCTGGGCGCACTTGGGACTCTGGC
CATAAAAGTCCTGGAGAAGAGACCAAGGTTGCTGCTGCCATTCTTGCACTGGCCTGGGGT
ACCCAAGTCCCCTCACTCACCGCTGTGACATCTCGGAAGAATTGGTAGGAGTCCCCAAGG
CCTGCAACAGCTACAACAGGAGCGCTGGCTGCCAGTGCCCCAGCCACCAGGTGGGGGTAC
TTCATCCTCATGTAGGCACTCAGCATCCCCCCATAACTGGGAGTACAGAGCACAGATCAT
GGTTGTGGGAAGCTGCCCACAACTCAGGCGAGCAGCCTCACTGTCCTCCAGGCTGAGGTG
CTAGGCTGCTCTTTCCCTGCTCAGAACGCCCAAGGGTGGGAAAGAAGGACCTGAAACTGT
CAGGCCCACACACCCTGATCCCAGGGCCAAGGCAGATACAGCCTTCACTGGGAGAAGGCA
CCTGTGGGTGCCCTGCCCTGACCCAGCAATGAAGACATTGCAGAGACAAAGTCAGAAGGA
ATTGTCCCACTAGTGGGAACAACATAGCATACACTGCCTATGAGGTCCACTCAAGGAGGG
CTTCCAGAAGGAGGTAAAGCTAGACCCCGCCCTTCCACATGTGGGGTAGGCATAGGATGT
TGAGACTGTAAGAGACATCTCTTTGGCCCTCCTTGTATAGGGTGTCAATCGGCACAACAG

>148 Get primers
CTGAGGAAACTGCTGGCAAATGTGAAGGGCAAGAATGAATGCCCAAGGTGGGCAGCAGGT
GAGGAAAGAGTCCCTCACCTCCCTGGAGGAACAAGTCTTTGATTTGCTGAAAGGCATCCC
GCACAGCCTGGGCGCACTTGGGACTCTGGCCATAAAAGTCCTGGAGAAGAGACCAAGGTT
GCTGCTGCCATTCTTGCACTGGCCTGGGGTACCCAAGTCCCCTCACTCACCGCTGTGACA
TCTCGGAAGAATTGGTAGGAGTCCCCAAGGCCTGCAACAGCTACAACAGGAGCGCTGGCT
GCCAGTGCCCCAGCCACCAGGTGGGGGTACTTCATCCTCATGTAGGCACTCAGCATCCCC
CCATAACTGGGAGTACAGAGCACAGATCATGGTTGTGGGAAGCTGCCCACAACTCAGGCG
AGCAGCCTCACTGTCCTCCAGGCTGAGGTGCTAGGCTGCTCTTTCCCTGCTCAGAACGCC
CAAGGGTGGGAAAGAAGGACCTGAAACTGTCAGGCCCACACACCCTGATCCCAGGGCCAA
GGCAGATACAGCCTTCACTGGGAGAAGGCACCTGTGGGTGCCCTGCCCTGACCCAGCAAT
GAAGACATTGCAGAGACAAAGTCAGAAGGAATTGTCCCACTAGTGGGAACAACATAGCAT
ACACTGCCTATGAGGTCCACTCAAGGAGGGCTTCCAGAAGGAGGTAAAGCTAGACCCCGC
CCTTCCACATGTGGGGTAGGCATAGGATGTTGAGACTGTAAGAGACATCTCTTTGGCCCT
CCTTGTATAGGGTGTCAATCGGCACAACAGGGTGGAGCCTTAGAGTAGGGTAAGATTAGG
ACTCTAGGTTCTCTCATGGGTCCAGATCTGTCATGAAGGGAGGTCAAGGACCCACCTCCC

>149 Get primers
ACAAGTCTTTGATTTGCTGAAAGGCATCCCGCACAGCCTGGGCGCACTTGGGACTCTGGC
CATAAAAGTCCTGGAGAAGAGACCAAGGTTGCTGCTGCCATTCTTGCACTGGCCTGGGGT
ACCCAAGTCCCCTCACTCACCGCTGTGACATCTCGGAAGAATTGGTAGGAGTCCCCAAGG
CCTGCAACAGCTACAACAGGAGCGCTGGCTGCCAGTGCCCCAGCCACCAGGTGGGGGTAC
TTCATCCTCATGTAGGCACTCAGCATCCCCCCATAACTGGGAGTACAGAGCACAGATCAT
GGTTGTGGGAAGCTGCCCACAACTCAGGCGAGCAGCCTCACTGTCCTCCAGGCTGAGGTG
CTAGGCTGCTCTTTCCCTGCTCAGAACGCCCAAGGGTGGGAAAGAAGGACCTGAAACTGT
CAGGCCCACACACCCTGATCCCAGGGCCAAGGCAGATACAGCCTTCACTGGGAGAAGGCA
CCTGTGGGTGCCCTGCCCTGACCCAGCAATGAAGACATTGCAGAGACAAAGTCAGAAGGA
ATTGTCCCACTAGTGGGAACAACATAGCATACACTGCCTATGAGGTCCACTCAAGGAGGG
CTTCCAGAAGGAGGTAAAGCTAGACCCCGCCCTTCCACATGTGGGGTAGGCATAGGATGT
TGAGACTGTAAGAGACATCTCTTTGGCCCTCCTTGTATAGGGTGTCAATCGGCACAACAG
GGTGGAGCCTTAGAGTAGGGTAAGATTAGGACTCTAGGTTCTCTCATGGGTCCAGATCTG
TCATGAAGGGAGGTCAAGGACCCACCTCCCTCCAAAGGCTATGGTGGGGGCATCATGGAC
CCCAAGGTCCTGCCGCAGGGCCTGGAGCAGCACAGCAAAGTCGGCCAGCGCCTGCTCCAC

>159 Get primers
AGTCAGCAGCTGTGTATATCCCCGCTGTGTGGACTGGACACCGAACGGAAGCGATTTCCC
ATAGTACCGCTGCAGAAAGCAGGAAGGGATGGCTAATCCACTCCTCGGTGCTCCCCACCT
CCTTCAACTCAGGGACTGCCAGGAACTGTACAGGTACCCACGTGCTCAGCAAAGACAAGC
AGGGCCTCCTGCTGGGCTGCCAGTTCCACCATGAAGCCAGAGTTGTTAGCGAAGGACCAG
ATATCCCCCTCATTCCCTGTGTAGAAAAAGATGGGCCCTTCGCCCATCTTCCAGAACTTA
TCTGTTGGAAGTAAATGAGTTTCCATAAGGCCAGGGAAACGCAGGTAGGAACCCATGCGG
TCGAGCCAGCACTCACCTGACACTAGGAACCGCTGGCCAAAGGTTTTGTTGCCGAAACTC
TCAAAGTTGAAATGGTCCATGTATTGCTCAAAATAATTCTCATGAAAGTCAGGGTCTAGA
ACTCTGTCGGCTGAGGGCAGGTGCAGAGACTCAGGAGCTGGTTGGGATCATCAGGGATCT
AGGCGGGTCAGGAGGAAGGGCAGCCAGTCTGTACTCACCTCTGGCCTGGAGGTTGCACAG
TCCCAGTGACAGCAGCAGGACCAGGATCCAGGAGGGGACACCATGGTCCACAGGGTAACA
AGGATGGAAGTTCATGCTTGATTCTGAGCCGGGCGCTGACTGTCATGTGATTTGGTCACA
TGACCGACACAACGGGCGGGGCAGCATCACGTGATAGTCTGGCGGGGGCTGTCCTACTGT
GGCTGGATTCTAGTTGGAGGATCAGCCTACTCTTCTTCAGTTTCCCGGTTCCTCCAAATT
TCTGGGCTCCTACTTGTTTCCACAGAGATGGATACTGTGGAGGTCCAGGAAGCAGAGAGA

>160 Get primers
GGCTAATCCACTCCTCGGTGCTCCCCACCTCCTTCAACTCAGGGACTGCCAGGAACTGTA
CAGGTACCCACGTGCTCAGCAAAGACAAGCAGGGCCTCCTGCTGGGCTGCCAGTTCCACC
ATGAAGCCAGAGTTGTTAGCGAAGGACCAGATATCCCCCTCATTCCCTGTGTAGAAAAAG
ATGGGCCCTTCGCCCATCTTCCAGAACTTATCTGTTGGAAGTAAATGAGTTTCCATAAGG
CCAGGGAAACGCAGGTAGGAACCCATGCGGTCGAGCCAGCACTCACCTGACACTAGGAAC
CGCTGGCCAAAGGTTTTGTTGCCGAAACTCTCAAAGTTGAAATGGTCCATGTATTGCTCA
AAATAATTCTCATGAAAGTCAGGGTCTAGAACTCTGTCGGCTGAGGGCAGGTGCAGAGAC
TCAGGAGCTGGTTGGGATCATCAGGGATCTAGGCGGGTCAGGAGGAAGGGCAGCCAGTCT
GTACTCACCTCTGGCCTGGAGGTTGCACAGTCCCAGTGACAGCAGCAGGACCAGGATCCA
GGAGGGGACACCATGGTCCACAGGGTAACAAGGATGGAAGTTCATGCTTGATTCTGAGCC
GGGCGCTGACTGTCATGTGATTTGGTCACATGACCGACACAACGGGCGGGGCAGCATCAC
GTGATAGTCTGGCGGGGGCTGTCCTACTGTGGCTGGATTCTAGTTGGAGGATCAGCCTAC
TCTTCTTCAGTTTCCCGGTTCCTCCAAATTTCTGGGCTCCTACTTGTTTCCACAGAGATG
GATACTGTGGAGGTCCAGGAAGCAGAGAGATGGCTAAGGCTCATCAGGACCGTATGATCT
CCCAAGTGTCCAGCTACTGAGTACCACAAGGTGATGGGTGGGAGGGTCCTCCCACGGAAG

>161 Get primers
AGGGCCTCCTGCTGGGCTGCCAGTTCCACCATGAAGCCAGAGTTGTTAGCGAAGGACCAG
ATATCCCCCTCATTCCCTGTGTAGAAAAAGATGGGCCCTTCGCCCATCTTCCAGAACTTA
TCTGTTGGAAGTAAATGAGTTTCCATAAGGCCAGGGAAACGCAGGTAGGAACCCATGCGG
TCGAGCCAGCACTCACCTGACACTAGGAACCGCTGGCCAAAGGTTTTGTTGCCGAAACTC
TCAAAGTTGAAATGGTCCATGTATTGCTCAAAATAATTCTCATGAAAGTCAGGGTCTAGA
ACTCTGTCGGCTGAGGGCAGGTGCAGAGACTCAGGAGCTGGTTGGGATCATCAGGGATCT
AGGCGGGTCAGGAGGAAGGGCAGCCAGTCTGTACTCACCTCTGGCCTGGAGGTTGCACAG
TCCCAGTGACAGCAGCAGGACCAGGATCCAGGAGGGGACACCATGGTCCACAGGGTAACA
AGGATGGAAGTTCATGCTTGATTCTGAGCCGGGCGCTGACTGTCATGTGATTTGGTCACA
TGACCGACACAACGGGCGGGGCAGCATCACGTGATAGTCTGGCGGGGGCTGTCCTACTGT
GGCTGGATTCTAGTTGGAGGATCAGCCTACTCTTCTTCAGTTTCCCGGTTCCTCCAAATT
TCTGGGCTCCTACTTGTTTCCACAGAGATGGATACTGTGGAGGTCCAGGAAGCAGAGAGA
TGGCTAAGGCTCATCAGGACCGTATGATCTCCCAAGTGTCCAGCTACTGAGTACCACAAG
GTGATGGGTGGGAGGGTCCTCCCACGGAAGGATACCGCAGTCCCTAGGGGTTGCAAGCCC
CACATGTTCCACTGGCTGCTAGAGCTACCTACTCAATCAGCCCTGGGCATCACCATCAGG

>162 Get primers
ATGGGCCCTTCGCCCATCTTCCAGAACTTATCTGTTGGAAGTAAATGAGTTTCCATAAGG
CCAGGGAAACGCAGGTAGGAACCCATGCGGTCGAGCCAGCACTCACCTGACACTAGGAAC
CGCTGGCCAAAGGTTTTGTTGCCGAAACTCTCAAAGTTGAAATGGTCCATGTATTGCTCA
AAATAATTCTCATGAAAGTCAGGGTCTAGAACTCTGTCGGCTGAGGGCAGGTGCAGAGAC
TCAGGAGCTGGTTGGGATCATCAGGGATCTAGGCGGGTCAGGAGGAAGGGCAGCCAGTCT
GTACTCACCTCTGGCCTGGAGGTTGCACAGTCCCAGTGACAGCAGCAGGACCAGGATCCA
GGAGGGGACACCATGGTCCACAGGGTAACAAGGATGGAAGTTCATGCTTGATTCTGAGCC
GGGCGCTGACTGTCATGTGATTTGGTCACATGACCGACACAACGGGCGGGGCAGCATCAC
GTGATAGTCTGGCGGGGGCTGTCCTACTGTGGCTGGATTCTAGTTGGAGGATCAGCCTAC
TCTTCTTCAGTTTCCCGGTTCCTCCAAATTTCTGGGCTCCTACTTGTTTCCACAGAGATG
GATACTGTGGAGGTCCAGGAAGCAGAGAGATGGCTAAGGCTCATCAGGACCGTATGATCT
CCCAAGTGTCCAGCTACTGAGTACCACAAGGTGATGGGTGGGAGGGTCCTCCCACGGAAG
GATACCGCAGTCCCTAGGGGTTGCAAGCCCCACATGTTCCACTGGCTGCTAGAGCTACCT
ACTCAATCAGCCCTGGGCATCACCATCAGGTACTCGGCCAAAATGACCTCTCTGCTTCCA
GTCCTCAGTTCTGGTCAGCACCAGACAGGCCCATAATTACAGAGCCAGGGAAACTGGAAC

>163 Get primers
TCGAGCCAGCACTCACCTGACACTAGGAACCGCTGGCCAAAGGTTTTGTTGCCGAAACTC
TCAAAGTTGAAATGGTCCATGTATTGCTCAAAATAATTCTCATGAAAGTCAGGGTCTAGA
ACTCTGTCGGCTGAGGGCAGGTGCAGAGACTCAGGAGCTGGTTGGGATCATCAGGGATCT
AGGCGGGTCAGGAGGAAGGGCAGCCAGTCTGTACTCACCTCTGGCCTGGAGGTTGCACAG
TCCCAGTGACAGCAGCAGGACCAGGATCCAGGAGGGGACACCATGGTCCACAGGGTAACA
AGGATGGAAGTTCATGCTTGATTCTGAGCCGGGCGCTGACTGTCATGTGATTTGGTCACA
TGACCGACACAACGGGCGGGGCAGCATCACGTGATAGTCTGGCGGGGGCTGTCCTACTGT
GGCTGGATTCTAGTTGGAGGATCAGCCTACTCTTCTTCAGTTTCCCGGTTCCTCCAAATT
TCTGGGCTCCTACTTGTTTCCACAGAGATGGATACTGTGGAGGTCCAGGAAGCAGAGAGA
TGGCTAAGGCTCATCAGGACCGTATGATCTCCCAAGTGTCCAGCTACTGAGTACCACAAG
GTGATGGGTGGGAGGGTCCTCCCACGGAAGGATACCGCAGTCCCTAGGGGTTGCAAGCCC
CACATGTTCCACTGGCTGCTAGAGCTACCTACTCAATCAGCCCTGGGCATCACCATCAGG
TACTCGGCCAAAATGACCTCTCTGCTTCCAGTCCTCAGTTCTGGTCAGCACCAGACAGGC
CCATAATTACAGAGCCAGGGAAACTGGAACATTTGTCTCCCCTTAGACAGTGGCAGCAGG
AAGGTGGGGGGTTGTTGCAGAGGAACAGTGTCTCTGAGAGAGGACCTTGGACTTTCTGGG

>164 Get primers
AAATAATTCTCATGAAAGTCAGGGTCTAGAACTCTGTCGGCTGAGGGCAGGTGCAGAGAC
TCAGGAGCTGGTTGGGATCATCAGGGATCTAGGCGGGTCAGGAGGAAGGGCAGCCAGTCT
GTACTCACCTCTGGCCTGGAGGTTGCACAGTCCCAGTGACAGCAGCAGGACCAGGATCCA
GGAGGGGACACCATGGTCCACAGGGTAACAAGGATGGAAGTTCATGCTTGATTCTGAGCC
GGGCGCTGACTGTCATGTGATTTGGTCACATGACCGACACAACGGGCGGGGCAGCATCAC
GTGATAGTCTGGCGGGGGCTGTCCTACTGTGGCTGGATTCTAGTTGGAGGATCAGCCTAC
TCTTCTTCAGTTTCCCGGTTCCTCCAAATTTCTGGGCTCCTACTTGTTTCCACAGAGATG
GATACTGTGGAGGTCCAGGAAGCAGAGAGATGGCTAAGGCTCATCAGGACCGTATGATCT
CCCAAGTGTCCAGCTACTGAGTACCACAAGGTGATGGGTGGGAGGGTCCTCCCACGGAAG
GATACCGCAGTCCCTAGGGGTTGCAAGCCCCACATGTTCCACTGGCTGCTAGAGCTACCT
ACTCAATCAGCCCTGGGCATCACCATCAGGTACTCGGCCAAAATGACCTCTCTGCTTCCA
GTCCTCAGTTCTGGTCAGCACCAGACAGGCCCATAATTACAGAGCCAGGGAAACTGGAAC
ATTTGTCTCCCCTTAGACAGTGGCAGCAGGAAGGTGGGGGGTTGTTGCAGAGGAACAGTG
TCTCTGAGAGAGGACCTTGGACTTTCTGGGAATCTCTGAGCTGCCCGGTTCTCCCCACTG
CTGGCACTGTGCCCACAGCCCAAACAGAATGGGGGAGATGGAGGGGCAGGGCTTCTGTGG

>165 Get primers
AGGCGGGTCAGGAGGAAGGGCAGCCAGTCTGTACTCACCTCTGGCCTGGAGGTTGCACAG
TCCCAGTGACAGCAGCAGGACCAGGATCCAGGAGGGGACACCATGGTCCACAGGGTAACA
AGGATGGAAGTTCATGCTTGATTCTGAGCCGGGCGCTGACTGTCATGTGATTTGGTCACA
TGACCGACACAACGGGCGGGGCAGCATCACGTGATAGTCTGGCGGGGGCTGTCCTACTGT
GGCTGGATTCTAGTTGGAGGATCAGCCTACTCTTCTTCAGTTTCCCGGTTCCTCCAAATT
TCTGGGCTCCTACTTGTTTCCACAGAGATGGATACTGTGGAGGTCCAGGAAGCAGAGAGA
TGGCTAAGGCTCATCAGGACCGTATGATCTCCCAAGTGTCCAGCTACTGAGTACCACAAG
GTGATGGGTGGGAGGGTCCTCCCACGGAAGGATACCGCAGTCCCTAGGGGTTGCAAGCCC
CACATGTTCCACTGGCTGCTAGAGCTACCTACTCAATCAGCCCTGGGCATCACCATCAGG
TACTCGGCCAAAATGACCTCTCTGCTTCCAGTCCTCAGTTCTGGTCAGCACCAGACAGGC
CCATAATTACAGAGCCAGGGAAACTGGAACATTTGTCTCCCCTTAGACAGTGGCAGCAGG
AAGGTGGGGGGTTGTTGCAGAGGAACAGTGTCTCTGAGAGAGGACCTTGGACTTTCTGGG
AATCTCTGAGCTGCCCGGTTCTCCCCACTGCTGGCACTGTGCCCACAGCCCAAACAGAAT
GGGGGAGATGGAGGGGCAGGGCTTCTGTGGGAAGCTGCCCTCCACCTCATTGGCACAGAG
TGTCTCATTGCAGAGAGAAAAAAGGACCAGTTTTCTCTCTGGCACCCAGGTCTGGAAGAG

>166 Get primers
GGAGGGGACACCATGGTCCACAGGGTAACAAGGATGGAAGTTCATGCTTGATTCTGAGCC
GGGCGCTGACTGTCATGTGATTTGGTCACATGACCGACACAACGGGCGGGGCAGCATCAC
GTGATAGTCTGGCGGGGGCTGTCCTACTGTGGCTGGATTCTAGTTGGAGGATCAGCCTAC
TCTTCTTCAGTTTCCCGGTTCCTCCAAATTTCTGGGCTCCTACTTGTTTCCACAGAGATG
GATACTGTGGAGGTCCAGGAAGCAGAGAGATGGCTAAGGCTCATCAGGACCGTATGATCT
CCCAAGTGTCCAGCTACTGAGTACCACAAGGTGATGGGTGGGAGGGTCCTCCCACGGAAG
GATACCGCAGTCCCTAGGGGTTGCAAGCCCCACATGTTCCACTGGCTGCTAGAGCTACCT
ACTCAATCAGCCCTGGGCATCACCATCAGGTACTCGGCCAAAATGACCTCTCTGCTTCCA
GTCCTCAGTTCTGGTCAGCACCAGACAGGCCCATAATTACAGAGCCAGGGAAACTGGAAC
ATTTGTCTCCCCTTAGACAGTGGCAGCAGGAAGGTGGGGGGTTGTTGCAGAGGAACAGTG
TCTCTGAGAGAGGACCTTGGACTTTCTGGGAATCTCTGAGCTGCCCGGTTCTCCCCACTG
CTGGCACTGTGCCCACAGCCCAAACAGAATGGGGGAGATGGAGGGGCAGGGCTTCTGTGG
GAAGCTGCCCTCCACCTCATTGGCACAGAGTGTCTCATTGCAGAGAGAAAAAAGGACCAG
TTTTCTCTCTGGCACCCAGGTCTGGAAGAGGAGTGACATCCACGGAAGTTGGTGACTTGG
ACTGGCTGGCCGTGAGTGGAACATGTCCATCCAGCATGGCCACAGTCCAGTGGGACACAC

>167 Get primers
AGGCCAAGAAGTAGACCCACCTGCCAGTGCCCGTAGACCCATGATCCTCTGGCCCTCATT
CAACAGCCGTTGACAGCCCACCTAGAGAAAGGCAGCAGAATATCTCAGTGGAGGCCCCTT
TCACAGAGCGTGGGTCAGGGCTGCTAGCTTCCAGGACACAACAGCAGATAGTGTCTGATG
GCATGAAAGCAGATAGCTACAAGGCTCTTGGACCAGGCTAGCACTGGGTCCTGCACCCAG
GGAGAGCCACCTCACCTTGACAGGGTTGGCAGGAAGGGGCCCTAGAAAGTCAGTAGGATA
CGGGTAGTCCATCATGGCGAGCACAGTAAATGCATTTCGGGCAAACCCAAAGAGCTGAGT
CAGGTCCTTTGGGCTGGAAAGTGATTGACAGGTACCAAAGTTCTGGCTGATGGTGTCATA
GGCTGGGAAGAGAGAGGCCAGGAGAAAAGGCTGAGGAAACTGCTGGCAAATGTGAAGGGC
AAGAATGAATGCCCAAGGTGGGCAGCAGGTGAGGAAAGAGTCCCTCACCTCCCTGGAGGA
ACAAGTCTTTGATTTGCTGAAAGGCATCCCGCACAGCCTGGGCGCACTTGGGACTCTGGC
CATAAAAGTCCTGGAGAAGAGACCAAGGTTGCTGCTGCCATTCTTGCACTGGCCTGGGGT
ACCCAAGTCCCCTCACTCACCGCTGTGACATCTCGGAAGAATTGGTAGGAGTCCCCAAGG
CCTGCAACAGCTACAACAGGAGCGCTGGCTGCCAGTGCCCCAGCCACCAGGTGGGGGTAC
TTCATCCTCATGTAGGCACTCAGCATCCCCCCATAACTGGGAGTACAGAGCACAGATCAT
GGTTGTGGGA

>168 Get primers
AGAAAGGCAGCAGAATATCTCAGTGGAGGCCCCTTTCACAGAGCGTGGGTCAGGGCTGCT
AGCTTCCAGGACACAACAGCAGATAGTGTCTGATGGCATGAAAGCAGATAGCTACAAGGC
TCTTGGACCAGGCTAGCACTGGGTCCTGCACCCAGGGAGAGCCACCTCACCTTGACAGGG
TTGGCAGGAAGGGGCCCTAGAAAGTCAGTAGGATACGGGTAGTCCATCATGGCGAGCACA
GTAAATGCATTTCGGGCAAACCCAAAGAGCTGAGTCAGGTCCTTTGGGCTGGAAAGTGAT
TGACAGGTACCAAAGTTCTGGCTGATGGTGTCATAGGCTGGGAAGAGAGAGGCCAGGAGA
AAAGGCTGAGGAAACTGCTGGCAAATGTGAAGGGCAAGAATGAATGCCCAAGGTGGGCAG
CAGGTGAGGAAAGAGTCCCTCACCTCCCTGGAGGAACAAGTCTTTGATTTGCTGAAAGGC
ATCCCGCACAGCCTGGGCGCACTTGGGACTCTGGCCATAAAAGTCCTGGAGAAGAGACCA
AGGTTGCTGCTGCCATTCTTGCACTGGCCTGGGGTACCCAAGTCCCCTCACTCACCGCTG
TGACATCTCGGAAGAATTGGTAGGAGTCCCCAAGGCCTGCAACAGCTACAACAGGAGCGC
TGGCTGCCAGTGCCCCAGCCACCAGGTGGGGGTACTTCATCCTCATGTAGGCACTCAGCA
TCCCCCCATAACTGGGAGTACAGAGCACAGATCATGGTTGTGGGAAGCTGCCCACAACTC
AGGCGAGCAGCCTCACTGTCCTCCAGGCTGAGGTGCTAGGCTGCTCTTTCCCTGCTCAGA
ACGCCCAAGG

>169 Get primers
GTGTCTGATGGCATGAAAGCAGATAGCTACAAGGCTCTTGGACCAGGCTAGCACTGGGTC
CTGCACCCAGGGAGAGCCACCTCACCTTGACAGGGTTGGCAGGAAGGGGCCCTAGAAAGT
CAGTAGGATACGGGTAGTCCATCATGGCGAGCACAGTAAATGCATTTCGGGCAAACCCAA
AGAGCTGAGTCAGGTCCTTTGGGCTGGAAAGTGATTGACAGGTACCAAAGTTCTGGCTGA
TGGTGTCATAGGCTGGGAAGAGAGAGGCCAGGAGAAAAGGCTGAGGAAACTGCTGGCAAA
TGTGAAGGGCAAGAATGAATGCCCAAGGTGGGCAGCAGGTGAGGAAAGAGTCCCTCACCT
CCCTGGAGGAACAAGTCTTTGATTTGCTGAAAGGCATCCCGCACAGCCTGGGCGCACTTG
GGACTCTGGCCATAAAAGTCCTGGAGAAGAGACCAAGGTTGCTGCTGCCATTCTTGCACT
GGCCTGGGGTACCCAAGTCCCCTCACTCACCGCTGTGACATCTCGGAAGAATTGGTAGGA
GTCCCCAAGGCCTGCAACAGCTACAACAGGAGCGCTGGCTGCCAGTGCCCCAGCCACCAG
GTGGGGGTACTTCATCCTCATGTAGGCACTCAGCATCCCCCCATAACTGGGAGTACAGAG
CACAGATCATGGTTGTGGGAAGCTGCCCACAACTCAGGCGAGCAGCCTCACTGTCCTCCA
GGCTGAGGTGCTAGGCTGCTCTTTCCCTGCTCAGAACGCCCAAGGGTGGGAAAGAAGGAC
CTGAAACTGTCAGGCCCACACACCCTGATCCCAGGGCCAAGGCAGATACAGCCTTCACTG
GGAGAAGGCA

>170 Get primers
CTTGACAGGGTTGGCAGGAAGGGGCCCTAGAAAGTCAGTAGGATACGGGTAGTCCATCAT
GGCGAGCACAGTAAATGCATTTCGGGCAAACCCAAAGAGCTGAGTCAGGTCCTTTGGGCT
GGAAAGTGATTGACAGGTACCAAAGTTCTGGCTGATGGTGTCATAGGCTGGGAAGAGAGA
GGCCAGGAGAAAAGGCTGAGGAAACTGCTGGCAAATGTGAAGGGCAAGAATGAATGCCCA
AGGTGGGCAGCAGGTGAGGAAAGAGTCCCTCACCTCCCTGGAGGAACAAGTCTTTGATTT
GCTGAAAGGCATCCCGCACAGCCTGGGCGCACTTGGGACTCTGGCCATAAAAGTCCTGGA
GAAGAGACCAAGGTTGCTGCTGCCATTCTTGCACTGGCCTGGGGTACCCAAGTCCCCTCA
CTCACCGCTGTGACATCTCGGAAGAATTGGTAGGAGTCCCCAAGGCCTGCAACAGCTACA
ACAGGAGCGCTGGCTGCCAGTGCCCCAGCCACCAGGTGGGGGTACTTCATCCTCATGTAG
GCACTCAGCATCCCCCCATAACTGGGAGTACAGAGCACAGATCATGGTTGTGGGAAGCTG
CCCACAACTCAGGCGAGCAGCCTCACTGTCCTCCAGGCTGAGGTGCTAGGCTGCTCTTTC
CCTGCTCAGAACGCCCAAGGGTGGGAAAGAAGGACCTGAAACTGTCAGGCCCACACACCC
TGATCCCAGGGCCAAGGCAGATACAGCCTTCACTGGGAGAAGGCACCTGTGGGTGCCCTG
CCCTGACCCAGCAATGAAGACATTGCAGAGACAAAGTCAGAAGGAATTGTCCCACTAGTG
GGAACAACAT

>171 Get primers
GCAAACCCAAAGAGCTGAGTCAGGTCCTTTGGGCTGGAAAGTGATTGACAGGTACCAAAG
TTCTGGCTGATGGTGTCATAGGCTGGGAAGAGAGAGGCCAGGAGAAAAGGCTGAGGAAAC
TGCTGGCAAATGTGAAGGGCAAGAATGAATGCCCAAGGTGGGCAGCAGGTGAGGAAAGAG
TCCCTCACCTCCCTGGAGGAACAAGTCTTTGATTTGCTGAAAGGCATCCCGCACAGCCTG
GGCGCACTTGGGACTCTGGCCATAAAAGTCCTGGAGAAGAGACCAAGGTTGCTGCTGCCA
TTCTTGCACTGGCCTGGGGTACCCAAGTCCCCTCACTCACCGCTGTGACATCTCGGAAGA
ATTGGTAGGAGTCCCCAAGGCCTGCAACAGCTACAACAGGAGCGCTGGCTGCCAGTGCCC
CAGCCACCAGGTGGGGGTACTTCATCCTCATGTAGGCACTCAGCATCCCCCCATAACTGG
GAGTACAGAGCACAGATCATGGTTGTGGGAAGCTGCCCACAACTCAGGCGAGCAGCCTCA
CTGTCCTCCAGGCTGAGGTGCTAGGCTGCTCTTTCCCTGCTCAGAACGCCCAAGGGTGGG
AAAGAAGGACCTGAAACTGTCAGGCCCACACACCCTGATCCCAGGGCCAAGGCAGATACA
GCCTTCACTGGGAGAAGGCACCTGTGGGTGCCCTGCCCTGACCCAGCAATGAAGACATTG
CAGAGACAAAGTCAGAAGGAATTGTCCCACTAGTGGGAACAACATAGCATACACTGCCTA
TGAGGTCCACTCAAGGAGGGCTTCCAGAAGGAGGTAAAGCTAGACCCCGCCCTTCCACAT
GTGGGGTAGG

>172 Get primers
GGAAGAGAGAGGCCAGGAGAAAAGGCTGAGGAAACTGCTGGCAAATGTGAAGGGCAAGAA
TGAATGCCCAAGGTGGGCAGCAGGTGAGGAAAGAGTCCCTCACCTCCCTGGAGGAACAAG
TCTTTGATTTGCTGAAAGGCATCCCGCACAGCCTGGGCGCACTTGGGACTCTGGCCATAA
AAGTCCTGGAGAAGAGACCAAGGTTGCTGCTGCCATTCTTGCACTGGCCTGGGGTACCCA
AGTCCCCTCACTCACCGCTGTGACATCTCGGAAGAATTGGTAGGAGTCCCCAAGGCCTGC
AACAGCTACAACAGGAGCGCTGGCTGCCAGTGCCCCAGCCACCAGGTGGGGGTACTTCAT
CCTCATGTAGGCACTCAGCATCCCCCCATAACTGGGAGTACAGAGCACAGATCATGGTTG
TGGGAAGCTGCCCACAACTCAGGCGAGCAGCCTCACTGTCCTCCAGGCTGAGGTGCTAGG
CTGCTCTTTCCCTGCTCAGAACGCCCAAGGGTGGGAAAGAAGGACCTGAAACTGTCAGGC
CCACACACCCTGATCCCAGGGCCAAGGCAGATACAGCCTTCACTGGGAGAAGGCACCTGT
GGGTGCCCTGCCCTGACCCAGCAATGAAGACATTGCAGAGACAAAGTCAGAAGGAATTGT
CCCACTAGTGGGAACAACATAGCATACACTGCCTATGAGGTCCACTCAAGGAGGGCTTCC
AGAAGGAGGTAAAGCTAGACCCCGCCCTTCCACATGTGGGGTAGGCATAGGATGTTGAGA
CTGTAAGAGACATCTCTTTGGCCCTCCTTGTATAGGGTGTCAATCGGCACAACAGGGTGG
AGCCTTAGAG

>173 Get primers
GAGGAAAGAGTCCCTCACCTCCCTGGAGGAACAAGTCTTTGATTTGCTGAAAGGCATCCC
GCACAGCCTGGGCGCACTTGGGACTCTGGCCATAAAAGTCCTGGAGAAGAGACCAAGGTT
GCTGCTGCCATTCTTGCACTGGCCTGGGGTACCCAAGTCCCCTCACTCACCGCTGTGACA
TCTCGGAAGAATTGGTAGGAGTCCCCAAGGCCTGCAACAGCTACAACAGGAGCGCTGGCT
GCCAGTGCCCCAGCCACCAGGTGGGGGTACTTCATCCTCATGTAGGCACTCAGCATCCCC
CCATAACTGGGAGTACAGAGCACAGATCATGGTTGTGGGAAGCTGCCCACAACTCAGGCG
AGCAGCCTCACTGTCCTCCAGGCTGAGGTGCTAGGCTGCTCTTTCCCTGCTCAGAACGCC
CAAGGGTGGGAAAGAAGGACCTGAAACTGTCAGGCCCACACACCCTGATCCCAGGGCCAA
GGCAGATACAGCCTTCACTGGGAGAAGGCACCTGTGGGTGCCCTGCCCTGACCCAGCAAT
GAAGACATTGCAGAGACAAAGTCAGAAGGAATTGTCCCACTAGTGGGAACAACATAGCAT
ACACTGCCTATGAGGTCCACTCAAGGAGGGCTTCCAGAAGGAGGTAAAGCTAGACCCCGC
CCTTCCACATGTGGGGTAGGCATAGGATGTTGAGACTGTAAGAGACATCTCTTTGGCCCT
CCTTGTATAGGGTGTCAATCGGCACAACAGGGTGGAGCCTTAGAGTAGGGTAAGATTAGG
ACTCTAGGTTCTCTCATGGGTCCAGATCTGTCATGAAGGGAGGTCAAGGACCCACCTCCC
TCCAAAGGCT

>184 Get primers
GCAGCTGTGTATATCCCCGCTGTGTGGACTGGACACCGAACGGAAGCGATTTCCCATAGT
ACCGCTGCAGAAAGCAGGAAGGGATGGCTAATCCACTCCTCGGTGCTCCCCACCTCCTTC
AACTCAGGGACTGCCAGGAACTGTACAGGTACCCACGTGCTCAGCAAAGACAAGCAGGGC
CTCCTGCTGGGCTGCCAGTTCCACCATGAAGCCAGAGTTGTTAGCGAAGGACCAGATATC
CCCCTCATTCCCTGTGTAGAAAAAGATGGGCCCTTCGCCCATCTTCCAGAACTTATCTGT
TGGAAGTAAATGAGTTTCCATAAGGCCAGGGAAACGCAGGTAGGAACCCATGCGGTCGAG
CCAGCACTCACCTGACACTAGGAACCGCTGGCCAAAGGTTTTGTTGCCGAAACTCTCAAA
GTTGAAATGGTCCATGTATTGCTCAAAATAATTCTCATGAAAGTCAGGGTCTAGAACTCT
GTCGGCTGAGGGCAGGTGCAGAGACTCAGGAGCTGGTTGGGATCATCAGGGATCTAGGCG
GGTCAGGAGGAAGGGCAGCCAGTCTGTACTCACCTCTGGCCTGGAGGTTGCACAGTCCCA
GTGACAGCAGCAGGACCAGGATCCAGGAGGGGACACCATGGTCCACAGGGTAACAAGGAT
GGAAGTTCATGCTTGATTCTGAGCCGGGCGCTGACTGTCATGTGATTTGGTCACATGACC
GACACAACGGGCGGGGCAGCATCACGTGATAGTCTGGCGGGGGCTGTCCTACTGTGGCTG
GATTCTAGTTGGAGGATCAGCCTACTCTTCTTCAGTTTCCCGGTTCCTCCAAATTTCTGG
GCTCCTACTT

>185 Get primers
GGCTAATCCACTCCTCGGTGCTCCCCACCTCCTTCAACTCAGGGACTGCCAGGAACTGTA
CAGGTACCCACGTGCTCAGCAAAGACAAGCAGGGCCTCCTGCTGGGCTGCCAGTTCCACC
ATGAAGCCAGAGTTGTTAGCGAAGGACCAGATATCCCCCTCATTCCCTGTGTAGAAAAAG
ATGGGCCCTTCGCCCATCTTCCAGAACTTATCTGTTGGAAGTAAATGAGTTTCCATAAGG
CCAGGGAAACGCAGGTAGGAACCCATGCGGTCGAGCCAGCACTCACCTGACACTAGGAAC
CGCTGGCCAAAGGTTTTGTTGCCGAAACTCTCAAAGTTGAAATGGTCCATGTATTGCTCA
AAATAATTCTCATGAAAGTCAGGGTCTAGAACTCTGTCGGCTGAGGGCAGGTGCAGAGAC
TCAGGAGCTGGTTGGGATCATCAGGGATCTAGGCGGGTCAGGAGGAAGGGCAGCCAGTCT
GTACTCACCTCTGGCCTGGAGGTTGCACAGTCCCAGTGACAGCAGCAGGACCAGGATCCA
GGAGGGGACACCATGGTCCACAGGGTAACAAGGATGGAAGTTCATGCTTGATTCTGAGCC
GGGCGCTGACTGTCATGTGATTTGGTCACATGACCGACACAACGGGCGGGGCAGCATCAC
GTGATAGTCTGGCGGGGGCTGTCCTACTGTGGCTGGATTCTAGTTGGAGGATCAGCCTAC
TCTTCTTCAGTTTCCCGGTTCCTCCAAATTTCTGGGCTCCTACTTGTTTCCACAGAGATG
GATACTGTGGAGGTCCAGGAAGCAGAGAGATGGCTAAGGCTCATCAGGACCGTATGATCT
CCCAAGTGTC

>186 Get primers
CAAGCAGGGCCTCCTGCTGGGCTGCCAGTTCCACCATGAAGCCAGAGTTGTTAGCGAAGG
ACCAGATATCCCCCTCATTCCCTGTGTAGAAAAAGATGGGCCCTTCGCCCATCTTCCAGA
ACTTATCTGTTGGAAGTAAATGAGTTTCCATAAGGCCAGGGAAACGCAGGTAGGAACCCA
TGCGGTCGAGCCAGCACTCACCTGACACTAGGAACCGCTGGCCAAAGGTTTTGTTGCCGA
AACTCTCAAAGTTGAAATGGTCCATGTATTGCTCAAAATAATTCTCATGAAAGTCAGGGT
CTAGAACTCTGTCGGCTGAGGGCAGGTGCAGAGACTCAGGAGCTGGTTGGGATCATCAGG
GATCTAGGCGGGTCAGGAGGAAGGGCAGCCAGTCTGTACTCACCTCTGGCCTGGAGGTTG
CACAGTCCCAGTGACAGCAGCAGGACCAGGATCCAGGAGGGGACACCATGGTCCACAGGG
TAACAAGGATGGAAGTTCATGCTTGATTCTGAGCCGGGCGCTGACTGTCATGTGATTTGG
TCACATGACCGACACAACGGGCGGGGCAGCATCACGTGATAGTCTGGCGGGGGCTGTCCT
ACTGTGGCTGGATTCTAGTTGGAGGATCAGCCTACTCTTCTTCAGTTTCCCGGTTCCTCC
AAATTTCTGGGCTCCTACTTGTTTCCACAGAGATGGATACTGTGGAGGTCCAGGAAGCAG
AGAGATGGCTAAGGCTCATCAGGACCGTATGATCTCCCAAGTGTCCAGCTACTGAGTACC
ACAAGGTGATGGGTGGGAGGGTCCTCCCACGGAAGGATACCGCAGTCCCTAGGGGTTGCA
AGCCCCACAT

>187 Get primers
GTAGAAAAAGATGGGCCCTTCGCCCATCTTCCAGAACTTATCTGTTGGAAGTAAATGAGT
TTCCATAAGGCCAGGGAAACGCAGGTAGGAACCCATGCGGTCGAGCCAGCACTCACCTGA
CACTAGGAACCGCTGGCCAAAGGTTTTGTTGCCGAAACTCTCAAAGTTGAAATGGTCCAT
GTATTGCTCAAAATAATTCTCATGAAAGTCAGGGTCTAGAACTCTGTCGGCTGAGGGCAG
GTGCAGAGACTCAGGAGCTGGTTGGGATCATCAGGGATCTAGGCGGGTCAGGAGGAAGGG
CAGCCAGTCTGTACTCACCTCTGGCCTGGAGGTTGCACAGTCCCAGTGACAGCAGCAGGA
CCAGGATCCAGGAGGGGACACCATGGTCCACAGGGTAACAAGGATGGAAGTTCATGCTTG
ATTCTGAGCCGGGCGCTGACTGTCATGTGATTTGGTCACATGACCGACACAACGGGCGGG
GCAGCATCACGTGATAGTCTGGCGGGGGCTGTCCTACTGTGGCTGGATTCTAGTTGGAGG
ATCAGCCTACTCTTCTTCAGTTTCCCGGTTCCTCCAAATTTCTGGGCTCCTACTTGTTTC
CACAGAGATGGATACTGTGGAGGTCCAGGAAGCAGAGAGATGGCTAAGGCTCATCAGGAC
CGTATGATCTCCCAAGTGTCCAGCTACTGAGTACCACAAGGTGATGGGTGGGAGGGTCCT
CCCACGGAAGGATACCGCAGTCCCTAGGGGTTGCAAGCCCCACATGTTCCACTGGCTGCT
AGAGCTACCTACTCAATCAGCCCTGGGCATCACCATCAGGTACTCGGCCAAAATGACCTC
TCTGCTTCCA

>188 Get primers
TAGGAACCCATGCGGTCGAGCCAGCACTCACCTGACACTAGGAACCGCTGGCCAAAGGTT
TTGTTGCCGAAACTCTCAAAGTTGAAATGGTCCATGTATTGCTCAAAATAATTCTCATGA
AAGTCAGGGTCTAGAACTCTGTCGGCTGAGGGCAGGTGCAGAGACTCAGGAGCTGGTTGG
GATCATCAGGGATCTAGGCGGGTCAGGAGGAAGGGCAGCCAGTCTGTACTCACCTCTGGC
CTGGAGGTTGCACAGTCCCAGTGACAGCAGCAGGACCAGGATCCAGGAGGGGACACCATG
GTCCACAGGGTAACAAGGATGGAAGTTCATGCTTGATTCTGAGCCGGGCGCTGACTGTCA
TGTGATTTGGTCACATGACCGACACAACGGGCGGGGCAGCATCACGTGATAGTCTGGCGG
GGGCTGTCCTACTGTGGCTGGATTCTAGTTGGAGGATCAGCCTACTCTTCTTCAGTTTCC
CGGTTCCTCCAAATTTCTGGGCTCCTACTTGTTTCCACAGAGATGGATACTGTGGAGGTC
CAGGAAGCAGAGAGATGGCTAAGGCTCATCAGGACCGTATGATCTCCCAAGTGTCCAGCT
ACTGAGTACCACAAGGTGATGGGTGGGAGGGTCCTCCCACGGAAGGATACCGCAGTCCCT
AGGGGTTGCAAGCCCCACATGTTCCACTGGCTGCTAGAGCTACCTACTCAATCAGCCCTG
GGCATCACCATCAGGTACTCGGCCAAAATGACCTCTCTGCTTCCAGTCCTCAGTTCTGGT
CAGCACCAGACAGGCCCATAATTACAGAGCCAGGGAAACTGGAACATTTGTCTCCCCTTA
GACAGTGGCA

>189 Get primers
AATGGTCCATGTATTGCTCAAAATAATTCTCATGAAAGTCAGGGTCTAGAACTCTGTCGG
CTGAGGGCAGGTGCAGAGACTCAGGAGCTGGTTGGGATCATCAGGGATCTAGGCGGGTCA
GGAGGAAGGGCAGCCAGTCTGTACTCACCTCTGGCCTGGAGGTTGCACAGTCCCAGTGAC
AGCAGCAGGACCAGGATCCAGGAGGGGACACCATGGTCCACAGGGTAACAAGGATGGAAG
TTCATGCTTGATTCTGAGCCGGGCGCTGACTGTCATGTGATTTGGTCACATGACCGACAC
AACGGGCGGGGCAGCATCACGTGATAGTCTGGCGGGGGCTGTCCTACTGTGGCTGGATTC
TAGTTGGAGGATCAGCCTACTCTTCTTCAGTTTCCCGGTTCCTCCAAATTTCTGGGCTCC
TACTTGTTTCCACAGAGATGGATACTGTGGAGGTCCAGGAAGCAGAGAGATGGCTAAGGC
TCATCAGGACCGTATGATCTCCCAAGTGTCCAGCTACTGAGTACCACAAGGTGATGGGTG
GGAGGGTCCTCCCACGGAAGGATACCGCAGTCCCTAGGGGTTGCAAGCCCCACATGTTCC
ACTGGCTGCTAGAGCTACCTACTCAATCAGCCCTGGGCATCACCATCAGGTACTCGGCCA
AAATGACCTCTCTGCTTCCAGTCCTCAGTTCTGGTCAGCACCAGACAGGCCCATAATTAC
AGAGCCAGGGAAACTGGAACATTTGTCTCCCCTTAGACAGTGGCAGCAGGAAGGTGGGGG
GTTGTTGCAGAGGAACAGTGTCTCTGAGAGAGGACCTTGGACTTTCTGGGAATCTCTGAG
CTGCCCGGTT

>190 Get primers
AGCTGGTTGGGATCATCAGGGATCTAGGCGGGTCAGGAGGAAGGGCAGCCAGTCTGTACT
CACCTCTGGCCTGGAGGTTGCACAGTCCCAGTGACAGCAGCAGGACCAGGATCCAGGAGG
GGACACCATGGTCCACAGGGTAACAAGGATGGAAGTTCATGCTTGATTCTGAGCCGGGCG
CTGACTGTCATGTGATTTGGTCACATGACCGACACAACGGGCGGGGCAGCATCACGTGAT
AGTCTGGCGGGGGCTGTCCTACTGTGGCTGGATTCTAGTTGGAGGATCAGCCTACTCTTC
TTCAGTTTCCCGGTTCCTCCAAATTTCTGGGCTCCTACTTGTTTCCACAGAGATGGATAC
TGTGGAGGTCCAGGAAGCAGAGAGATGGCTAAGGCTCATCAGGACCGTATGATCTCCCAA
GTGTCCAGCTACTGAGTACCACAAGGTGATGGGTGGGAGGGTCCTCCCACGGAAGGATAC
CGCAGTCCCTAGGGGTTGCAAGCCCCACATGTTCCACTGGCTGCTAGAGCTACCTACTCA
ATCAGCCCTGGGCATCACCATCAGGTACTCGGCCAAAATGACCTCTCTGCTTCCAGTCCT
CAGTTCTGGTCAGCACCAGACAGGCCCATAATTACAGAGCCAGGGAAACTGGAACATTTG
TCTCCCCTTAGACAGTGGCAGCAGGAAGGTGGGGGGTTGTTGCAGAGGAACAGTGTCTCT
GAGAGAGGACCTTGGACTTTCTGGGAATCTCTGAGCTGCCCGGTTCTCCCCACTGCTGGC
ACTGTGCCCACAGCCCAAACAGAATGGGGGAGATGGAGGGGCAGGGCTTCTGTGGGAAGC
TGCCCTCCAC

>191 Get primers
TCCCAGTGACAGCAGCAGGACCAGGATCCAGGAGGGGACACCATGGTCCACAGGGTAACA
AGGATGGAAGTTCATGCTTGATTCTGAGCCGGGCGCTGACTGTCATGTGATTTGGTCACA
TGACCGACACAACGGGCGGGGCAGCATCACGTGATAGTCTGGCGGGGGCTGTCCTACTGT
GGCTGGATTCTAGTTGGAGGATCAGCCTACTCTTCTTCAGTTTCCCGGTTCCTCCAAATT
TCTGGGCTCCTACTTGTTTCCACAGAGATGGATACTGTGGAGGTCCAGGAAGCAGAGAGA
TGGCTAAGGCTCATCAGGACCGTATGATCTCCCAAGTGTCCAGCTACTGAGTACCACAAG
GTGATGGGTGGGAGGGTCCTCCCACGGAAGGATACCGCAGTCCCTAGGGGTTGCAAGCCC
CACATGTTCCACTGGCTGCTAGAGCTACCTACTCAATCAGCCCTGGGCATCACCATCAGG
TACTCGGCCAAAATGACCTCTCTGCTTCCAGTCCTCAGTTCTGGTCAGCACCAGACAGGC
CCATAATTACAGAGCCAGGGAAACTGGAACATTTGTCTCCCCTTAGACAGTGGCAGCAGG
AAGGTGGGGGGTTGTTGCAGAGGAACAGTGTCTCTGAGAGAGGACCTTGGACTTTCTGGG
AATCTCTGAGCTGCCCGGTTCTCCCCACTGCTGGCACTGTGCCCACAGCCCAAACAGAAT
GGGGGAGATGGAGGGGCAGGGCTTCTGTGGGAAGCTGCCCTCCACCTCATTGGCACAGAG
TGTCTCATTGCAGAGAGAAAAAAGGACCAGTTTTCTCTCTGGCACCCAGGTCTGGAAGAG
GAGTGACATC

>192 Get primers
GAGCCGGGCGCTGACTGTCATGTGATTTGGTCACATGACCGACACAACGGGCGGGGCAGC
ATCACGTGATAGTCTGGCGGGGGCTGTCCTACTGTGGCTGGATTCTAGTTGGAGGATCAG
CCTACTCTTCTTCAGTTTCCCGGTTCCTCCAAATTTCTGGGCTCCTACTTGTTTCCACAG
AGATGGATACTGTGGAGGTCCAGGAAGCAGAGAGATGGCTAAGGCTCATCAGGACCGTAT
GATCTCCCAAGTGTCCAGCTACTGAGTACCACAAGGTGATGGGTGGGAGGGTCCTCCCAC
GGAAGGATACCGCAGTCCCTAGGGGTTGCAAGCCCCACATGTTCCACTGGCTGCTAGAGC
TACCTACTCAATCAGCCCTGGGCATCACCATCAGGTACTCGGCCAAAATGACCTCTCTGC
TTCCAGTCCTCAGTTCTGGTCAGCACCAGACAGGCCCATAATTACAGAGCCAGGGAAACT
GGAACATTTGTCTCCCCTTAGACAGTGGCAGCAGGAAGGTGGGGGGTTGTTGCAGAGGAA
CAGTGTCTCTGAGAGAGGACCTTGGACTTTCTGGGAATCTCTGAGCTGCCCGGTTCTCCC
CACTGCTGGCACTGTGCCCACAGCCCAAACAGAATGGGGGAGATGGAGGGGCAGGGCTTC
TGTGGGAAGCTGCCCTCCACCTCATTGGCACAGAGTGTCTCATTGCAGAGAGAAAAAAGG
ACCAGTTTTCTCTCTGGCACCCAGGTCTGGAAGAGGAGTGACATCCACGGAAGTTGGTGA
CTTGGACTGGCTGGCCGTGAGTGGAACATGTCCATCCAGCATGGCCACAGTCCAGTGGGA
CACACAGCCT

>193 Get primers
AGGCCAAGAAGTAGACCCACCTGCCAGTGCCCGTAGACCCATGATCCTCTGGCCCTCATT
CAACAGCCGTTGACAGCCCACCTAGAGAAAGGCAGCAGAATATCTCAGTGGAGGCCCCTT
TCACAGAGCGTGGGTCAGGGCTGCTAGCTTCCAGGACACAACAGCAGATAGTGTCTGATG
GCATGAAAGCAGATAGCTACAAGGCTCTTGGACCAGGCTAGCACTGGGTCCTGCACCCAG
GGAGAGCCACCTCACCTTGACAGGGTTGGCAGGAAGGGGCCCTAGAAAGTCAGTAGGATA
CGGGTAGTCCATCATGGCGAGCACAGTAAATGCATTTCGGGCAAACCCAAAGAGCTGAGT
CAGGTCCTTTGGGCTGGAAAGTGATTGACAGGTACCAAAGTTCTGGCTGATGGTGTCATA
GGCTGGGAAGAGAGAGGCCAGGAGAAAAGGCTGAGGAAACTGCTGGCAAATGTGAAGGGC
AAGAATGAATGCCCAAGGTGGGCAGCAGGTGAGGAAAGAGTCCCTCACCTCCCTGGAGGA
ACAAGTCTTTGATTTGCTGAAAGGCATCCCGCACAGCCTGGGCGCACTTGGGACTCTGGC
CATAAAAGTCCTGGAGAAGAGACCAAGGTTGCTGCTGCCATTCTTGCACTGGCCTGGGGT
ACCCAAGTCCCCTCACTCACCGCTGTGACATCTCGGAAGAATTGGTAGGAGTCCCCAAGG
CCTGCAACAGCTACAACAGGAGCGCTGGCTGCCAGTGCCCCAGCCACCAGGTGGGGGTAC
TTCATCCTCATGTAGGCACT

>194 Get primers
CCTAGAGAAAGGCAGCAGAATATCTCAGTGGAGGCCCCTTTCACAGAGCGTGGGTCAGGG
CTGCTAGCTTCCAGGACACAACAGCAGATAGTGTCTGATGGCATGAAAGCAGATAGCTAC
AAGGCTCTTGGACCAGGCTAGCACTGGGTCCTGCACCCAGGGAGAGCCACCTCACCTTGA
CAGGGTTGGCAGGAAGGGGCCCTAGAAAGTCAGTAGGATACGGGTAGTCCATCATGGCGA
GCACAGTAAATGCATTTCGGGCAAACCCAAAGAGCTGAGTCAGGTCCTTTGGGCTGGAAA
GTGATTGACAGGTACCAAAGTTCTGGCTGATGGTGTCATAGGCTGGGAAGAGAGAGGCCA
GGAGAAAAGGCTGAGGAAACTGCTGGCAAATGTGAAGGGCAAGAATGAATGCCCAAGGTG
GGCAGCAGGTGAGGAAAGAGTCCCTCACCTCCCTGGAGGAACAAGTCTTTGATTTGCTGA
AAGGCATCCCGCACAGCCTGGGCGCACTTGGGACTCTGGCCATAAAAGTCCTGGAGAAGA
GACCAAGGTTGCTGCTGCCATTCTTGCACTGGCCTGGGGTACCCAAGTCCCCTCACTCAC
CGCTGTGACATCTCGGAAGAATTGGTAGGAGTCCCCAAGGCCTGCAACAGCTACAACAGG
AGCGCTGGCTGCCAGTGCCCCAGCCACCAGGTGGGGGTACTTCATCCTCATGTAGGCACT
CAGCATCCCCCCATAACTGGGAGTACAGAGCACAGATCATGGTTGTGGGAAGCTGCCCAC
AACTCAGGCGAGCAGCCTCA

>195 Get primers
ACAGCAGATAGTGTCTGATGGCATGAAAGCAGATAGCTACAAGGCTCTTGGACCAGGCTA
GCACTGGGTCCTGCACCCAGGGAGAGCCACCTCACCTTGACAGGGTTGGCAGGAAGGGGC
CCTAGAAAGTCAGTAGGATACGGGTAGTCCATCATGGCGAGCACAGTAAATGCATTTCGG
GCAAACCCAAAGAGCTGAGTCAGGTCCTTTGGGCTGGAAAGTGATTGACAGGTACCAAAG
TTCTGGCTGATGGTGTCATAGGCTGGGAAGAGAGAGGCCAGGAGAAAAGGCTGAGGAAAC
TGCTGGCAAATGTGAAGGGCAAGAATGAATGCCCAAGGTGGGCAGCAGGTGAGGAAAGAG
TCCCTCACCTCCCTGGAGGAACAAGTCTTTGATTTGCTGAAAGGCATCCCGCACAGCCTG
GGCGCACTTGGGACTCTGGCCATAAAAGTCCTGGAGAAGAGACCAAGGTTGCTGCTGCCA
TTCTTGCACTGGCCTGGGGTACCCAAGTCCCCTCACTCACCGCTGTGACATCTCGGAAGA
ATTGGTAGGAGTCCCCAAGGCCTGCAACAGCTACAACAGGAGCGCTGGCTGCCAGTGCCC
CAGCCACCAGGTGGGGGTACTTCATCCTCATGTAGGCACTCAGCATCCCCCCATAACTGG
GAGTACAGAGCACAGATCATGGTTGTGGGAAGCTGCCCACAACTCAGGCGAGCAGCCTCA
CTGTCCTCCAGGCTGAGGTGCTAGGCTGCTCTTTCCCTGCTCAGAACGCCCAAGGGTGGG
AAAGAAGGACCTGAAACTGT

>196 Get primers
GGAGAGCCACCTCACCTTGACAGGGTTGGCAGGAAGGGGCCCTAGAAAGTCAGTAGGATA
CGGGTAGTCCATCATGGCGAGCACAGTAAATGCATTTCGGGCAAACCCAAAGAGCTGAGT
CAGGTCCTTTGGGCTGGAAAGTGATTGACAGGTACCAAAGTTCTGGCTGATGGTGTCATA
GGCTGGGAAGAGAGAGGCCAGGAGAAAAGGCTGAGGAAACTGCTGGCAAATGTGAAGGGC
AAGAATGAATGCCCAAGGTGGGCAGCAGGTGAGGAAAGAGTCCCTCACCTCCCTGGAGGA
ACAAGTCTTTGATTTGCTGAAAGGCATCCCGCACAGCCTGGGCGCACTTGGGACTCTGGC
CATAAAAGTCCTGGAGAAGAGACCAAGGTTGCTGCTGCCATTCTTGCACTGGCCTGGGGT
ACCCAAGTCCCCTCACTCACCGCTGTGACATCTCGGAAGAATTGGTAGGAGTCCCCAAGG
CCTGCAACAGCTACAACAGGAGCGCTGGCTGCCAGTGCCCCAGCCACCAGGTGGGGGTAC
TTCATCCTCATGTAGGCACTCAGCATCCCCCCATAACTGGGAGTACAGAGCACAGATCAT
GGTTGTGGGAAGCTGCCCACAACTCAGGCGAGCAGCCTCACTGTCCTCCAGGCTGAGGTG
CTAGGCTGCTCTTTCCCTGCTCAGAACGCCCAAGGGTGGGAAAGAAGGACCTGAAACTGT
CAGGCCCACACACCCTGATCCCAGGGCCAAGGCAGATACAGCCTTCACTGGGAGAAGGCA
CCTGTGGGTGCCCTGCCCTG

>197 Get primers
GCACAGTAAATGCATTTCGGGCAAACCCAAAGAGCTGAGTCAGGTCCTTTGGGCTGGAAA
GTGATTGACAGGTACCAAAGTTCTGGCTGATGGTGTCATAGGCTGGGAAGAGAGAGGCCA
GGAGAAAAGGCTGAGGAAACTGCTGGCAAATGTGAAGGGCAAGAATGAATGCCCAAGGTG
GGCAGCAGGTGAGGAAAGAGTCCCTCACCTCCCTGGAGGAACAAGTCTTTGATTTGCTGA
AAGGCATCCCGCACAGCCTGGGCGCACTTGGGACTCTGGCCATAAAAGTCCTGGAGAAGA
GACCAAGGTTGCTGCTGCCATTCTTGCACTGGCCTGGGGTACCCAAGTCCCCTCACTCAC
CGCTGTGACATCTCGGAAGAATTGGTAGGAGTCCCCAAGGCCTGCAACAGCTACAACAGG
AGCGCTGGCTGCCAGTGCCCCAGCCACCAGGTGGGGGTACTTCATCCTCATGTAGGCACT
CAGCATCCCCCCATAACTGGGAGTACAGAGCACAGATCATGGTTGTGGGAAGCTGCCCAC
AACTCAGGCGAGCAGCCTCACTGTCCTCCAGGCTGAGGTGCTAGGCTGCTCTTTCCCTGC
TCAGAACGCCCAAGGGTGGGAAAGAAGGACCTGAAACTGTCAGGCCCACACACCCTGATC
CCAGGGCCAAGGCAGATACAGCCTTCACTGGGAGAAGGCACCTGTGGGTGCCCTGCCCTG
ACCCAGCAATGAAGACATTGCAGAGACAAAGTCAGAAGGAATTGTCCCACTAGTGGGAAC
AACATAGCATACACTGCCTA

>198 Get primers
TTCTGGCTGATGGTGTCATAGGCTGGGAAGAGAGAGGCCAGGAGAAAAGGCTGAGGAAAC
TGCTGGCAAATGTGAAGGGCAAGAATGAATGCCCAAGGTGGGCAGCAGGTGAGGAAAGAG
TCCCTCACCTCCCTGGAGGAACAAGTCTTTGATTTGCTGAAAGGCATCCCGCACAGCCTG
GGCGCACTTGGGACTCTGGCCATAAAAGTCCTGGAGAAGAGACCAAGGTTGCTGCTGCCA
TTCTTGCACTGGCCTGGGGTACCCAAGTCCCCTCACTCACCGCTGTGACATCTCGGAAGA
ATTGGTAGGAGTCCCCAAGGCCTGCAACAGCTACAACAGGAGCGCTGGCTGCCAGTGCCC
CAGCCACCAGGTGGGGGTACTTCATCCTCATGTAGGCACTCAGCATCCCCCCATAACTGG
GAGTACAGAGCACAGATCATGGTTGTGGGAAGCTGCCCACAACTCAGGCGAGCAGCCTCA
CTGTCCTCCAGGCTGAGGTGCTAGGCTGCTCTTTCCCTGCTCAGAACGCCCAAGGGTGGG
AAAGAAGGACCTGAAACTGTCAGGCCCACACACCCTGATCCCAGGGCCAAGGCAGATACA
GCCTTCACTGGGAGAAGGCACCTGTGGGTGCCCTGCCCTGACCCAGCAATGAAGACATTG
CAGAGACAAAGTCAGAAGGAATTGTCCCACTAGTGGGAACAACATAGCATACACTGCCTA
TGAGGTCCACTCAAGGAGGGCTTCCAGAAGGAGGTAAAGCTAGACCCCGCCCTTCCACAT
GTGGGGTAGGCATAGGATGT

>199 Get primers
AAGAATGAATGCCCAAGGTGGGCAGCAGGTGAGGAAAGAGTCCCTCACCTCCCTGGAGGA
ACAAGTCTTTGATTTGCTGAAAGGCATCCCGCACAGCCTGGGCGCACTTGGGACTCTGGC
CATAAAAGTCCTGGAGAAGAGACCAAGGTTGCTGCTGCCATTCTTGCACTGGCCTGGGGT
ACCCAAGTCCCCTCACTCACCGCTGTGACATCTCGGAAGAATTGGTAGGAGTCCCCAAGG
CCTGCAACAGCTACAACAGGAGCGCTGGCTGCCAGTGCCCCAGCCACCAGGTGGGGGTAC
TTCATCCTCATGTAGGCACTCAGCATCCCCCCATAACTGGGAGTACAGAGCACAGATCAT
GGTTGTGGGAAGCTGCCCACAACTCAGGCGAGCAGCCTCACTGTCCTCCAGGCTGAGGTG
CTAGGCTGCTCTTTCCCTGCTCAGAACGCCCAAGGGTGGGAAAGAAGGACCTGAAACTGT
CAGGCCCACACACCCTGATCCCAGGGCCAAGGCAGATACAGCCTTCACTGGGAGAAGGCA
CCTGTGGGTGCCCTGCCCTGACCCAGCAATGAAGACATTGCAGAGACAAAGTCAGAAGGA
ATTGTCCCACTAGTGGGAACAACATAGCATACACTGCCTATGAGGTCCACTCAAGGAGGG
CTTCCAGAAGGAGGTAAAGCTAGACCCCGCCCTTCCACATGTGGGGTAGGCATAGGATGT
TGAGACTGTAAGAGACATCTCTTTGGCCCTCCTTGTATAGGGTGTCAATCGGCACAACAG
GGTGGAGCCTTAGAGTAGGG

>200 Get primers
AAGGCATCCCGCACAGCCTGGGCGCACTTGGGACTCTGGCCATAAAAGTCCTGGAGAAGA
GACCAAGGTTGCTGCTGCCATTCTTGCACTGGCCTGGGGTACCCAAGTCCCCTCACTCAC
CGCTGTGACATCTCGGAAGAATTGGTAGGAGTCCCCAAGGCCTGCAACAGCTACAACAGG
AGCGCTGGCTGCCAGTGCCCCAGCCACCAGGTGGGGGTACTTCATCCTCATGTAGGCACT
CAGCATCCCCCCATAACTGGGAGTACAGAGCACAGATCATGGTTGTGGGAAGCTGCCCAC
AACTCAGGCGAGCAGCCTCACTGTCCTCCAGGCTGAGGTGCTAGGCTGCTCTTTCCCTGC
TCAGAACGCCCAAGGGTGGGAAAGAAGGACCTGAAACTGTCAGGCCCACACACCCTGATC
CCAGGGCCAAGGCAGATACAGCCTTCACTGGGAGAAGGCACCTGTGGGTGCCCTGCCCTG
ACCCAGCAATGAAGACATTGCAGAGACAAAGTCAGAAGGAATTGTCCCACTAGTGGGAAC
AACATAGCATACACTGCCTATGAGGTCCACTCAAGGAGGGCTTCCAGAAGGAGGTAAAGC
TAGACCCCGCCCTTCCACATGTGGGGTAGGCATAGGATGTTGAGACTGTAAGAGACATCT
CTTTGGCCCTCCTTGTATAGGGTGTCAATCGGCACAACAGGGTGGAGCCTTAGAGTAGGG
TAAGATTAGGACTCTAGGTTCTCTCATGGGTCCAGATCTGTCATGAAGGGAGGTCAAGGA
CCCACCTCCCTCCAAAGGCT

>201 Get primers
TTCTTGCACTGGCCTGGGGTACCCAAGTCCCCTCACTCACCGCTGTGACATCTCGGAAGA
ATTGGTAGGAGTCCCCAAGGCCTGCAACAGCTACAACAGGAGCGCTGGCTGCCAGTGCCC
CAGCCACCAGGTGGGGGTACTTCATCCTCATGTAGGCACTCAGCATCCCCCCATAACTGG
GAGTACAGAGCACAGATCATGGTTGTGGGAAGCTGCCCACAACTCAGGCGAGCAGCCTCA
CTGTCCTCCAGGCTGAGGTGCTAGGCTGCTCTTTCCCTGCTCAGAACGCCCAAGGGTGGG
AAAGAAGGACCTGAAACTGTCAGGCCCACACACCCTGATCCCAGGGCCAAGGCAGATACA
GCCTTCACTGGGAGAAGGCACCTGTGGGTGCCCTGCCCTGACCCAGCAATGAAGACATTG
CAGAGACAAAGTCAGAAGGAATTGTCCCACTAGTGGGAACAACATAGCATACACTGCCTA
TGAGGTCCACTCAAGGAGGGCTTCCAGAAGGAGGTAAAGCTAGACCCCGCCCTTCCACAT
GTGGGGTAGGCATAGGATGTTGAGACTGTAAGAGACATCTCTTTGGCCCTCCTTGTATAG
GGTGTCAATCGGCACAACAGGGTGGAGCCTTAGAGTAGGGTAAGATTAGGACTCTAGGTT
CTCTCATGGGTCCAGATCTGTCATGAAGGGAGGTCAAGGACCCACCTCCCTCCAAAGGCT
ATGGTGGGGGCATCATGGACCCCAAGGTCCTGCCGCAGGGCCTGGAGCAGCACAGCAAAG
TCGGCCAGCGCCTGCTCCAC

>211 Get primers
AGTCAGCAGCTGTGTATATCCCCGCTGTGTGGACTGGACACCGAACGGAAGCGATTTCCC
ATAGTACCGCTGCAGAAAGCAGGAAGGGATGGCTAATCCACTCCTCGGTGCTCCCCACCT
CCTTCAACTCAGGGACTGCCAGGAACTGTACAGGTACCCACGTGCTCAGCAAAGACAAGC
AGGGCCTCCTGCTGGGCTGCCAGTTCCACCATGAAGCCAGAGTTGTTAGCGAAGGACCAG
ATATCCCCCTCATTCCCTGTGTAGAAAAAGATGGGCCCTTCGCCCATCTTCCAGAACTTA
TCTGTTGGAAGTAAATGAGTTTCCATAAGGCCAGGGAAACGCAGGTAGGAACCCATGCGG
TCGAGCCAGCACTCACCTGACACTAGGAACCGCTGGCCAAAGGTTTTGTTGCCGAAACTC
TCAAAGTTGAAATGGTCCATGTATTGCTCAAAATAATTCTCATGAAAGTCAGGGTCTAGA
ACTCTGTCGGCTGAGGGCAGGTGCAGAGACTCAGGAGCTGGTTGGGATCATCAGGGATCT
AGGCGGGTCAGGAGGAAGGGCAGCCAGTCTGTACTCACCTCTGGCCTGGAGGTTGCACAG
TCCCAGTGACAGCAGCAGGACCAGGATCCAGGAGGGGACACCATGGTCCACAGGGTAACA
AGGATGGAAGTTCATGCTTGATTCTGAGCCGGGCGCTGACTGTCATGTGATTTGGTCACA
TGACCGACACAACGGGCGGGGCAGCATCACGTGATAGTCTGGCGGGGGCTGTCCTACTGT
GGCTGGATTCTAGTTGGAGG

>212 Get primers
AGGAAGGGATGGCTAATCCACTCCTCGGTGCTCCCCACCTCCTTCAACTCAGGGACTGCC
AGGAACTGTACAGGTACCCACGTGCTCAGCAAAGACAAGCAGGGCCTCCTGCTGGGCTGC
CAGTTCCACCATGAAGCCAGAGTTGTTAGCGAAGGACCAGATATCCCCCTCATTCCCTGT
GTAGAAAAAGATGGGCCCTTCGCCCATCTTCCAGAACTTATCTGTTGGAAGTAAATGAGT
TTCCATAAGGCCAGGGAAACGCAGGTAGGAACCCATGCGGTCGAGCCAGCACTCACCTGA
CACTAGGAACCGCTGGCCAAAGGTTTTGTTGCCGAAACTCTCAAAGTTGAAATGGTCCAT
GTATTGCTCAAAATAATTCTCATGAAAGTCAGGGTCTAGAACTCTGTCGGCTGAGGGCAG
GTGCAGAGACTCAGGAGCTGGTTGGGATCATCAGGGATCTAGGCGGGTCAGGAGGAAGGG
CAGCCAGTCTGTACTCACCTCTGGCCTGGAGGTTGCACAGTCCCAGTGACAGCAGCAGGA
CCAGGATCCAGGAGGGGACACCATGGTCCACAGGGTAACAAGGATGGAAGTTCATGCTTG
ATTCTGAGCCGGGCGCTGACTGTCATGTGATTTGGTCACATGACCGACACAACGGGCGGG
GCAGCATCACGTGATAGTCTGGCGGGGGCTGTCCTACTGTGGCTGGATTCTAGTTGGAGG
ATCAGCCTACTCTTCTTCAGTTTCCCGGTTCCTCCAAATTTCTGGGCTCCTACTTGTTTC
CACAGAGATGGATACTGTGG

>213 Get primers
CGTGCTCAGCAAAGACAAGCAGGGCCTCCTGCTGGGCTGCCAGTTCCACCATGAAGCCAG
AGTTGTTAGCGAAGGACCAGATATCCCCCTCATTCCCTGTGTAGAAAAAGATGGGCCCTT
CGCCCATCTTCCAGAACTTATCTGTTGGAAGTAAATGAGTTTCCATAAGGCCAGGGAAAC
GCAGGTAGGAACCCATGCGGTCGAGCCAGCACTCACCTGACACTAGGAACCGCTGGCCAA
AGGTTTTGTTGCCGAAACTCTCAAAGTTGAAATGGTCCATGTATTGCTCAAAATAATTCT
CATGAAAGTCAGGGTCTAGAACTCTGTCGGCTGAGGGCAGGTGCAGAGACTCAGGAGCTG
GTTGGGATCATCAGGGATCTAGGCGGGTCAGGAGGAAGGGCAGCCAGTCTGTACTCACCT
CTGGCCTGGAGGTTGCACAGTCCCAGTGACAGCAGCAGGACCAGGATCCAGGAGGGGACA
CCATGGTCCACAGGGTAACAAGGATGGAAGTTCATGCTTGATTCTGAGCCGGGCGCTGAC
TGTCATGTGATTTGGTCACATGACCGACACAACGGGCGGGGCAGCATCACGTGATAGTCT
GGCGGGGGCTGTCCTACTGTGGCTGGATTCTAGTTGGAGGATCAGCCTACTCTTCTTCAG
TTTCCCGGTTCCTCCAAATTTCTGGGCTCCTACTTGTTTCCACAGAGATGGATACTGTGG
AGGTCCAGGAAGCAGAGAGATGGCTAAGGCTCATCAGGACCGTATGATCTCCCAAGTGTC
CAGCTACTGAGTACCACAAG

>214 Get primers
ATATCCCCCTCATTCCCTGTGTAGAAAAAGATGGGCCCTTCGCCCATCTTCCAGAACTTA
TCTGTTGGAAGTAAATGAGTTTCCATAAGGCCAGGGAAACGCAGGTAGGAACCCATGCGG
TCGAGCCAGCACTCACCTGACACTAGGAACCGCTGGCCAAAGGTTTTGTTGCCGAAACTC
TCAAAGTTGAAATGGTCCATGTATTGCTCAAAATAATTCTCATGAAAGTCAGGGTCTAGA
ACTCTGTCGGCTGAGGGCAGGTGCAGAGACTCAGGAGCTGGTTGGGATCATCAGGGATCT
AGGCGGGTCAGGAGGAAGGGCAGCCAGTCTGTACTCACCTCTGGCCTGGAGGTTGCACAG
TCCCAGTGACAGCAGCAGGACCAGGATCCAGGAGGGGACACCATGGTCCACAGGGTAACA
AGGATGGAAGTTCATGCTTGATTCTGAGCCGGGCGCTGACTGTCATGTGATTTGGTCACA
TGACCGACACAACGGGCGGGGCAGCATCACGTGATAGTCTGGCGGGGGCTGTCCTACTGT
GGCTGGATTCTAGTTGGAGGATCAGCCTACTCTTCTTCAGTTTCCCGGTTCCTCCAAATT
TCTGGGCTCCTACTTGTTTCCACAGAGATGGATACTGTGGAGGTCCAGGAAGCAGAGAGA
TGGCTAAGGCTCATCAGGACCGTATGATCTCCCAAGTGTCCAGCTACTGAGTACCACAAG
GTGATGGGTGGGAGGGTCCTCCCACGGAAGGATACCGCAGTCCCTAGGGGTTGCAAGCCC
CACATGTTCCACTGGCTGCT

>215 Get primers
TTCCATAAGGCCAGGGAAACGCAGGTAGGAACCCATGCGGTCGAGCCAGCACTCACCTGA
CACTAGGAACCGCTGGCCAAAGGTTTTGTTGCCGAAACTCTCAAAGTTGAAATGGTCCAT
GTATTGCTCAAAATAATTCTCATGAAAGTCAGGGTCTAGAACTCTGTCGGCTGAGGGCAG
GTGCAGAGACTCAGGAGCTGGTTGGGATCATCAGGGATCTAGGCGGGTCAGGAGGAAGGG
CAGCCAGTCTGTACTCACCTCTGGCCTGGAGGTTGCACAGTCCCAGTGACAGCAGCAGGA
CCAGGATCCAGGAGGGGACACCATGGTCCACAGGGTAACAAGGATGGAAGTTCATGCTTG
ATTCTGAGCCGGGCGCTGACTGTCATGTGATTTGGTCACATGACCGACACAACGGGCGGG
GCAGCATCACGTGATAGTCTGGCGGGGGCTGTCCTACTGTGGCTGGATTCTAGTTGGAGG
ATCAGCCTACTCTTCTTCAGTTTCCCGGTTCCTCCAAATTTCTGGGCTCCTACTTGTTTC
CACAGAGATGGATACTGTGGAGGTCCAGGAAGCAGAGAGATGGCTAAGGCTCATCAGGAC
CGTATGATCTCCCAAGTGTCCAGCTACTGAGTACCACAAGGTGATGGGTGGGAGGGTCCT
CCCACGGAAGGATACCGCAGTCCCTAGGGGTTGCAAGCCCCACATGTTCCACTGGCTGCT
AGAGCTACCTACTCAATCAGCCCTGGGCATCACCATCAGGTACTCGGCCAAAATGACCTC
TCTGCTTCCAGTCCTCAGTT

>216 Get primers
AGGTTTTGTTGCCGAAACTCTCAAAGTTGAAATGGTCCATGTATTGCTCAAAATAATTCT
CATGAAAGTCAGGGTCTAGAACTCTGTCGGCTGAGGGCAGGTGCAGAGACTCAGGAGCTG
GTTGGGATCATCAGGGATCTAGGCGGGTCAGGAGGAAGGGCAGCCAGTCTGTACTCACCT
CTGGCCTGGAGGTTGCACAGTCCCAGTGACAGCAGCAGGACCAGGATCCAGGAGGGGACA
CCATGGTCCACAGGGTAACAAGGATGGAAGTTCATGCTTGATTCTGAGCCGGGCGCTGAC
TGTCATGTGATTTGGTCACATGACCGACACAACGGGCGGGGCAGCATCACGTGATAGTCT
GGCGGGGGCTGTCCTACTGTGGCTGGATTCTAGTTGGAGGATCAGCCTACTCTTCTTCAG
TTTCCCGGTTCCTCCAAATTTCTGGGCTCCTACTTGTTTCCACAGAGATGGATACTGTGG
AGGTCCAGGAAGCAGAGAGATGGCTAAGGCTCATCAGGACCGTATGATCTCCCAAGTGTC
CAGCTACTGAGTACCACAAGGTGATGGGTGGGAGGGTCCTCCCACGGAAGGATACCGCAG
TCCCTAGGGGTTGCAAGCCCCACATGTTCCACTGGCTGCTAGAGCTACCTACTCAATCAG
CCCTGGGCATCACCATCAGGTACTCGGCCAAAATGACCTCTCTGCTTCCAGTCCTCAGTT
CTGGTCAGCACCAGACAGGCCCATAATTACAGAGCCAGGGAAACTGGAACATTTGTCTCC
CCTTAGACAGTGGCAGCAGG

>217 Get primers
ACTCTGTCGGCTGAGGGCAGGTGCAGAGACTCAGGAGCTGGTTGGGATCATCAGGGATCT
AGGCGGGTCAGGAGGAAGGGCAGCCAGTCTGTACTCACCTCTGGCCTGGAGGTTGCACAG
TCCCAGTGACAGCAGCAGGACCAGGATCCAGGAGGGGACACCATGGTCCACAGGGTAACA
AGGATGGAAGTTCATGCTTGATTCTGAGCCGGGCGCTGACTGTCATGTGATTTGGTCACA
TGACCGACACAACGGGCGGGGCAGCATCACGTGATAGTCTGGCGGGGGCTGTCCTACTGT
GGCTGGATTCTAGTTGGAGGATCAGCCTACTCTTCTTCAGTTTCCCGGTTCCTCCAAATT
TCTGGGCTCCTACTTGTTTCCACAGAGATGGATACTGTGGAGGTCCAGGAAGCAGAGAGA
TGGCTAAGGCTCATCAGGACCGTATGATCTCCCAAGTGTCCAGCTACTGAGTACCACAAG
GTGATGGGTGGGAGGGTCCTCCCACGGAAGGATACCGCAGTCCCTAGGGGTTGCAAGCCC
CACATGTTCCACTGGCTGCTAGAGCTACCTACTCAATCAGCCCTGGGCATCACCATCAGG
TACTCGGCCAAAATGACCTCTCTGCTTCCAGTCCTCAGTTCTGGTCAGCACCAGACAGGC
CCATAATTACAGAGCCAGGGAAACTGGAACATTTGTCTCCCCTTAGACAGTGGCAGCAGG
AAGGTGGGGGGTTGTTGCAGAGGAACAGTGTCTCTGAGAGAGGACCTTGGACTTTCTGGG
AATCTCTGAGCTGCCCGGTT

>218 Get primers
CAGCCAGTCTGTACTCACCTCTGGCCTGGAGGTTGCACAGTCCCAGTGACAGCAGCAGGA
CCAGGATCCAGGAGGGGACACCATGGTCCACAGGGTAACAAGGATGGAAGTTCATGCTTG
ATTCTGAGCCGGGCGCTGACTGTCATGTGATTTGGTCACATGACCGACACAACGGGCGGG
GCAGCATCACGTGATAGTCTGGCGGGGGCTGTCCTACTGTGGCTGGATTCTAGTTGGAGG
ATCAGCCTACTCTTCTTCAGTTTCCCGGTTCCTCCAAATTTCTGGGCTCCTACTTGTTTC
CACAGAGATGGATACTGTGGAGGTCCAGGAAGCAGAGAGATGGCTAAGGCTCATCAGGAC
CGTATGATCTCCCAAGTGTCCAGCTACTGAGTACCACAAGGTGATGGGTGGGAGGGTCCT
CCCACGGAAGGATACCGCAGTCCCTAGGGGTTGCAAGCCCCACATGTTCCACTGGCTGCT
AGAGCTACCTACTCAATCAGCCCTGGGCATCACCATCAGGTACTCGGCCAAAATGACCTC
TCTGCTTCCAGTCCTCAGTTCTGGTCAGCACCAGACAGGCCCATAATTACAGAGCCAGGG
AAACTGGAACATTTGTCTCCCCTTAGACAGTGGCAGCAGGAAGGTGGGGGGTTGTTGCAG
AGGAACAGTGTCTCTGAGAGAGGACCTTGGACTTTCTGGGAATCTCTGAGCTGCCCGGTT
CTCCCCACTGCTGGCACTGTGCCCACAGCCCAAACAGAATGGGGGAGATGGAGGGGCAGG
GCTTCTGTGGGAAGCTGCCC

>219 Get primers
CCATGGTCCACAGGGTAACAAGGATGGAAGTTCATGCTTGATTCTGAGCCGGGCGCTGAC
TGTCATGTGATTTGGTCACATGACCGACACAACGGGCGGGGCAGCATCACGTGATAGTCT
GGCGGGGGCTGTCCTACTGTGGCTGGATTCTAGTTGGAGGATCAGCCTACTCTTCTTCAG
TTTCCCGGTTCCTCCAAATTTCTGGGCTCCTACTTGTTTCCACAGAGATGGATACTGTGG
AGGTCCAGGAAGCAGAGAGATGGCTAAGGCTCATCAGGACCGTATGATCTCCCAAGTGTC
CAGCTACTGAGTACCACAAGGTGATGGGTGGGAGGGTCCTCCCACGGAAGGATACCGCAG
TCCCTAGGGGTTGCAAGCCCCACATGTTCCACTGGCTGCTAGAGCTACCTACTCAATCAG
CCCTGGGCATCACCATCAGGTACTCGGCCAAAATGACCTCTCTGCTTCCAGTCCTCAGTT
CTGGTCAGCACCAGACAGGCCCATAATTACAGAGCCAGGGAAACTGGAACATTTGTCTCC
CCTTAGACAGTGGCAGCAGGAAGGTGGGGGGTTGTTGCAGAGGAACAGTGTCTCTGAGAG
AGGACCTTGGACTTTCTGGGAATCTCTGAGCTGCCCGGTTCTCCCCACTGCTGGCACTGT
GCCCACAGCCCAAACAGAATGGGGGAGATGGAGGGGCAGGGCTTCTGTGGGAAGCTGCCC
TCCACCTCATTGGCACAGAGTGTCTCATTGCAGAGAGAAAAAAGGACCAGTTTTCTCTCT
GGCACCCAGGTCTGGAAGAG

>220 Get primers
TGACCGACACAACGGGCGGGGCAGCATCACGTGATAGTCTGGCGGGGGCTGTCCTACTGT
GGCTGGATTCTAGTTGGAGGATCAGCCTACTCTTCTTCAGTTTCCCGGTTCCTCCAAATT
TCTGGGCTCCTACTTGTTTCCACAGAGATGGATACTGTGGAGGTCCAGGAAGCAGAGAGA
TGGCTAAGGCTCATCAGGACCGTATGATCTCCCAAGTGTCCAGCTACTGAGTACCACAAG
GTGATGGGTGGGAGGGTCCTCCCACGGAAGGATACCGCAGTCCCTAGGGGTTGCAAGCCC
CACATGTTCCACTGGCTGCTAGAGCTACCTACTCAATCAGCCCTGGGCATCACCATCAGG
TACTCGGCCAAAATGACCTCTCTGCTTCCAGTCCTCAGTTCTGGTCAGCACCAGACAGGC
CCATAATTACAGAGCCAGGGAAACTGGAACATTTGTCTCCCCTTAGACAGTGGCAGCAGG
AAGGTGGGGGGTTGTTGCAGAGGAACAGTGTCTCTGAGAGAGGACCTTGGACTTTCTGGG
AATCTCTGAGCTGCCCGGTTCTCCCCACTGCTGGCACTGTGCCCACAGCCCAAACAGAAT
GGGGGAGATGGAGGGGCAGGGCTTCTGTGGGAAGCTGCCCTCCACCTCATTGGCACAGAG
TGTCTCATTGCAGAGAGAAAAAAGGACCAGTTTTCTCTCTGGCACCCAGGTCTGGAAGAG
GAGTGACATCCACGGAAGTTGGTGACTTGGACTGGCTGGCCGTGAGTGGAACATGTCCAT
CCAGCATGGCCACAGTCCAG

>221 Get primers
AGGCCAAGAAGTAGACCCACCTGCCAGTGCCCGTAGACCCATGATCCTCTGGCCCTCATT
CAACAGCCGTTGACAGCCCACCTAGAGAAAGGCAGCAGAATATCTCAGTGGAGGCCCCTT
TCACAGAGCGTGGGTCAGGGCTGCTAGCTTCCAGGACACAACAGCAGATAGTGTCTGATG
GCATGAAAGCAGATAGCTACAAGGCTCTTGGACCAGGCTAGCACTGGGTCCTGCACCCAG
GGAGAGCCACCTCACCTTGACAGGGTTGGCAGGAAGGGGCCCTAGAAAGTCAGTAGGATA
CGGGTAGTCCATCATGGCGAGCACAGTAAATGCATTTCGGGCAAACCCAAAGAGCTGAGT
CAGGTCCTTTGGGCTGGAAAGTGATTGACAGGTACCAAAGTTCTGGCTGATGGTGTCATA
GGCTGGGAAGAGAGAGGCCAGGAGAAAAGGCTGAGGAAACTGCTGGCAAATGTGAAGGGC
AAGAATGAATGCCCAAGGTGGGCAGCAGGTGAGGAAAGAGTCCCTCACCTCCCTGGAGGA
ACAAGTCTTTGATTTGCTGAAAGGCATCCCGCACAGCCTGGGCGCACTTGGGACTCTGGC
CATAAAAGTCCTGGAGAAGAGACCAAGGTTGCTGCTGCCATTCTTGCACTGGCCTGGGGT
ACCCAAGTCCCCTCACTCACCGCTGTGACATCTCGGAAGAATTGGTAGGAGTCCCCAAGG
CCTGCAACAGCTACAACAGGAGCGCTGGCT

>222 Get primers
GCCCACCTAGAGAAAGGCAGCAGAATATCTCAGTGGAGGCCCCTTTCACAGAGCGTGGGT
CAGGGCTGCTAGCTTCCAGGACACAACAGCAGATAGTGTCTGATGGCATGAAAGCAGATA
GCTACAAGGCTCTTGGACCAGGCTAGCACTGGGTCCTGCACCCAGGGAGAGCCACCTCAC
CTTGACAGGGTTGGCAGGAAGGGGCCCTAGAAAGTCAGTAGGATACGGGTAGTCCATCAT
GGCGAGCACAGTAAATGCATTTCGGGCAAACCCAAAGAGCTGAGTCAGGTCCTTTGGGCT
GGAAAGTGATTGACAGGTACCAAAGTTCTGGCTGATGGTGTCATAGGCTGGGAAGAGAGA
GGCCAGGAGAAAAGGCTGAGGAAACTGCTGGCAAATGTGAAGGGCAAGAATGAATGCCCA
AGGTGGGCAGCAGGTGAGGAAAGAGTCCCTCACCTCCCTGGAGGAACAAGTCTTTGATTT
GCTGAAAGGCATCCCGCACAGCCTGGGCGCACTTGGGACTCTGGCCATAAAAGTCCTGGA
GAAGAGACCAAGGTTGCTGCTGCCATTCTTGCACTGGCCTGGGGTACCCAAGTCCCCTCA
CTCACCGCTGTGACATCTCGGAAGAATTGGTAGGAGTCCCCAAGGCCTGCAACAGCTACA
ACAGGAGCGCTGGCTGCCAGTGCCCCAGCCACCAGGTGGGGGTACTTCATCCTCATGTAG
GCACTCAGCATCCCCCCATAACTGGGAGTA

>223 Get primers
CCAGGACACAACAGCAGATAGTGTCTGATGGCATGAAAGCAGATAGCTACAAGGCTCTTG
GACCAGGCTAGCACTGGGTCCTGCACCCAGGGAGAGCCACCTCACCTTGACAGGGTTGGC
AGGAAGGGGCCCTAGAAAGTCAGTAGGATACGGGTAGTCCATCATGGCGAGCACAGTAAA
TGCATTTCGGGCAAACCCAAAGAGCTGAGTCAGGTCCTTTGGGCTGGAAAGTGATTGACA
GGTACCAAAGTTCTGGCTGATGGTGTCATAGGCTGGGAAGAGAGAGGCCAGGAGAAAAGG
CTGAGGAAACTGCTGGCAAATGTGAAGGGCAAGAATGAATGCCCAAGGTGGGCAGCAGGT
GAGGAAAGAGTCCCTCACCTCCCTGGAGGAACAAGTCTTTGATTTGCTGAAAGGCATCCC
GCACAGCCTGGGCGCACTTGGGACTCTGGCCATAAAAGTCCTGGAGAAGAGACCAAGGTT
GCTGCTGCCATTCTTGCACTGGCCTGGGGTACCCAAGTCCCCTCACTCACCGCTGTGACA
TCTCGGAAGAATTGGTAGGAGTCCCCAAGGCCTGCAACAGCTACAACAGGAGCGCTGGCT
GCCAGTGCCCCAGCCACCAGGTGGGGGTACTTCATCCTCATGTAGGCACTCAGCATCCCC
CCATAACTGGGAGTACAGAGCACAGATCATGGTTGTGGGAAGCTGCCCACAACTCAGGCG
AGCAGCCTCACTGTCCTCCAGGCTGAGGTG

>224 Get primers
GGGTCCTGCACCCAGGGAGAGCCACCTCACCTTGACAGGGTTGGCAGGAAGGGGCCCTAG
AAAGTCAGTAGGATACGGGTAGTCCATCATGGCGAGCACAGTAAATGCATTTCGGGCAAA
CCCAAAGAGCTGAGTCAGGTCCTTTGGGCTGGAAAGTGATTGACAGGTACCAAAGTTCTG
GCTGATGGTGTCATAGGCTGGGAAGAGAGAGGCCAGGAGAAAAGGCTGAGGAAACTGCTG
GCAAATGTGAAGGGCAAGAATGAATGCCCAAGGTGGGCAGCAGGTGAGGAAAGAGTCCCT
CACCTCCCTGGAGGAACAAGTCTTTGATTTGCTGAAAGGCATCCCGCACAGCCTGGGCGC
ACTTGGGACTCTGGCCATAAAAGTCCTGGAGAAGAGACCAAGGTTGCTGCTGCCATTCTT
GCACTGGCCTGGGGTACCCAAGTCCCCTCACTCACCGCTGTGACATCTCGGAAGAATTGG
TAGGAGTCCCCAAGGCCTGCAACAGCTACAACAGGAGCGCTGGCTGCCAGTGCCCCAGCC
ACCAGGTGGGGGTACTTCATCCTCATGTAGGCACTCAGCATCCCCCCATAACTGGGAGTA
CAGAGCACAGATCATGGTTGTGGGAAGCTGCCCACAACTCAGGCGAGCAGCCTCACTGTC
CTCCAGGCTGAGGTGCTAGGCTGCTCTTTCCCTGCTCAGAACGCCCAAGGGTGGGAAAGA
AGGACCTGAAACTGTCAGGCCCACACACCC

>225 Get primers
CGGGTAGTCCATCATGGCGAGCACAGTAAATGCATTTCGGGCAAACCCAAAGAGCTGAGT
CAGGTCCTTTGGGCTGGAAAGTGATTGACAGGTACCAAAGTTCTGGCTGATGGTGTCATA
GGCTGGGAAGAGAGAGGCCAGGAGAAAAGGCTGAGGAAACTGCTGGCAAATGTGAAGGGC
AAGAATGAATGCCCAAGGTGGGCAGCAGGTGAGGAAAGAGTCCCTCACCTCCCTGGAGGA
ACAAGTCTTTGATTTGCTGAAAGGCATCCCGCACAGCCTGGGCGCACTTGGGACTCTGGC
CATAAAAGTCCTGGAGAAGAGACCAAGGTTGCTGCTGCCATTCTTGCACTGGCCTGGGGT
ACCCAAGTCCCCTCACTCACCGCTGTGACATCTCGGAAGAATTGGTAGGAGTCCCCAAGG
CCTGCAACAGCTACAACAGGAGCGCTGGCTGCCAGTGCCCCAGCCACCAGGTGGGGGTAC
TTCATCCTCATGTAGGCACTCAGCATCCCCCCATAACTGGGAGTACAGAGCACAGATCAT
GGTTGTGGGAAGCTGCCCACAACTCAGGCGAGCAGCCTCACTGTCCTCCAGGCTGAGGTG
CTAGGCTGCTCTTTCCCTGCTCAGAACGCCCAAGGGTGGGAAAGAAGGACCTGAAACTGT
CAGGCCCACACACCCTGATCCCAGGGCCAAGGCAGATACAGCCTTCACTGGGAGAAGGCA
CCTGTGGGTGCCCTGCCCTGACCCAGCAAT

>226 Get primers
GGAAAGTGATTGACAGGTACCAAAGTTCTGGCTGATGGTGTCATAGGCTGGGAAGAGAGA
GGCCAGGAGAAAAGGCTGAGGAAACTGCTGGCAAATGTGAAGGGCAAGAATGAATGCCCA
AGGTGGGCAGCAGGTGAGGAAAGAGTCCCTCACCTCCCTGGAGGAACAAGTCTTTGATTT
GCTGAAAGGCATCCCGCACAGCCTGGGCGCACTTGGGACTCTGGCCATAAAAGTCCTGGA
GAAGAGACCAAGGTTGCTGCTGCCATTCTTGCACTGGCCTGGGGTACCCAAGTCCCCTCA
CTCACCGCTGTGACATCTCGGAAGAATTGGTAGGAGTCCCCAAGGCCTGCAACAGCTACA
ACAGGAGCGCTGGCTGCCAGTGCCCCAGCCACCAGGTGGGGGTACTTCATCCTCATGTAG
GCACTCAGCATCCCCCCATAACTGGGAGTACAGAGCACAGATCATGGTTGTGGGAAGCTG
CCCACAACTCAGGCGAGCAGCCTCACTGTCCTCCAGGCTGAGGTGCTAGGCTGCTCTTTC
CCTGCTCAGAACGCCCAAGGGTGGGAAAGAAGGACCTGAAACTGTCAGGCCCACACACCC
TGATCCCAGGGCCAAGGCAGATACAGCCTTCACTGGGAGAAGGCACCTGTGGGTGCCCTG
CCCTGACCCAGCAATGAAGACATTGCAGAGACAAAGTCAGAAGGAATTGTCCCACTAGTG
GGAACAACATAGCATACACTGCCTATGAGG

>227 Get primers
CTGAGGAAACTGCTGGCAAATGTGAAGGGCAAGAATGAATGCCCAAGGTGGGCAGCAGGT
GAGGAAAGAGTCCCTCACCTCCCTGGAGGAACAAGTCTTTGATTTGCTGAAAGGCATCCC
GCACAGCCTGGGCGCACTTGGGACTCTGGCCATAAAAGTCCTGGAGAAGAGACCAAGGTT
GCTGCTGCCATTCTTGCACTGGCCTGGGGTACCCAAGTCCCCTCACTCACCGCTGTGACA
TCTCGGAAGAATTGGTAGGAGTCCCCAAGGCCTGCAACAGCTACAACAGGAGCGCTGGCT
GCCAGTGCCCCAGCCACCAGGTGGGGGTACTTCATCCTCATGTAGGCACTCAGCATCCCC
CCATAACTGGGAGTACAGAGCACAGATCATGGTTGTGGGAAGCTGCCCACAACTCAGGCG
AGCAGCCTCACTGTCCTCCAGGCTGAGGTGCTAGGCTGCTCTTTCCCTGCTCAGAACGCC
CAAGGGTGGGAAAGAAGGACCTGAAACTGTCAGGCCCACACACCCTGATCCCAGGGCCAA
GGCAGATACAGCCTTCACTGGGAGAAGGCACCTGTGGGTGCCCTGCCCTGACCCAGCAAT
GAAGACATTGCAGAGACAAAGTCAGAAGGAATTGTCCCACTAGTGGGAACAACATAGCAT
ACACTGCCTATGAGGTCCACTCAAGGAGGGCTTCCAGAAGGAGGTAAAGCTAGACCCCGC
CCTTCCACATGTGGGGTAGGCATAGGATGT

>228 Get primers
CACCTCCCTGGAGGAACAAGTCTTTGATTTGCTGAAAGGCATCCCGCACAGCCTGGGCGC
ACTTGGGACTCTGGCCATAAAAGTCCTGGAGAAGAGACCAAGGTTGCTGCTGCCATTCTT
GCACTGGCCTGGGGTACCCAAGTCCCCTCACTCACCGCTGTGACATCTCGGAAGAATTGG
TAGGAGTCCCCAAGGCCTGCAACAGCTACAACAGGAGCGCTGGCTGCCAGTGCCCCAGCC
ACCAGGTGGGGGTACTTCATCCTCATGTAGGCACTCAGCATCCCCCCATAACTGGGAGTA
CAGAGCACAGATCATGGTTGTGGGAAGCTGCCCACAACTCAGGCGAGCAGCCTCACTGTC
CTCCAGGCTGAGGTGCTAGGCTGCTCTTTCCCTGCTCAGAACGCCCAAGGGTGGGAAAGA
AGGACCTGAAACTGTCAGGCCCACACACCCTGATCCCAGGGCCAAGGCAGATACAGCCTT
CACTGGGAGAAGGCACCTGTGGGTGCCCTGCCCTGACCCAGCAATGAAGACATTGCAGAG
ACAAAGTCAGAAGGAATTGTCCCACTAGTGGGAACAACATAGCATACACTGCCTATGAGG
TCCACTCAAGGAGGGCTTCCAGAAGGAGGTAAAGCTAGACCCCGCCCTTCCACATGTGGG
GTAGGCATAGGATGTTGAGACTGTAAGAGACATCTCTTTGGCCCTCCTTGTATAGGGTGT
CAATCGGCACAACAGGGTGGAGCCTTAGAG

>229 Get primers
CATAAAAGTCCTGGAGAAGAGACCAAGGTTGCTGCTGCCATTCTTGCACTGGCCTGGGGT
ACCCAAGTCCCCTCACTCACCGCTGTGACATCTCGGAAGAATTGGTAGGAGTCCCCAAGG
CCTGCAACAGCTACAACAGGAGCGCTGGCTGCCAGTGCCCCAGCCACCAGGTGGGGGTAC
TTCATCCTCATGTAGGCACTCAGCATCCCCCCATAACTGGGAGTACAGAGCACAGATCAT
GGTTGTGGGAAGCTGCCCACAACTCAGGCGAGCAGCCTCACTGTCCTCCAGGCTGAGGTG
CTAGGCTGCTCTTTCCCTGCTCAGAACGCCCAAGGGTGGGAAAGAAGGACCTGAAACTGT
CAGGCCCACACACCCTGATCCCAGGGCCAAGGCAGATACAGCCTTCACTGGGAGAAGGCA
CCTGTGGGTGCCCTGCCCTGACCCAGCAATGAAGACATTGCAGAGACAAAGTCAGAAGGA
ATTGTCCCACTAGTGGGAACAACATAGCATACACTGCCTATGAGGTCCACTCAAGGAGGG
CTTCCAGAAGGAGGTAAAGCTAGACCCCGCCCTTCCACATGTGGGGTAGGCATAGGATGT
TGAGACTGTAAGAGACATCTCTTTGGCCCTCCTTGTATAGGGTGTCAATCGGCACAACAG
GGTGGAGCCTTAGAGTAGGGTAAGATTAGGACTCTAGGTTCTCTCATGGGTCCAGATCTG
TCATGAAGGGAGGTCAAGGACCCACCTCCC

>230 Get primers
CTCACCGCTGTGACATCTCGGAAGAATTGGTAGGAGTCCCCAAGGCCTGCAACAGCTACA
ACAGGAGCGCTGGCTGCCAGTGCCCCAGCCACCAGGTGGGGGTACTTCATCCTCATGTAG
GCACTCAGCATCCCCCCATAACTGGGAGTACAGAGCACAGATCATGGTTGTGGGAAGCTG
CCCACAACTCAGGCGAGCAGCCTCACTGTCCTCCAGGCTGAGGTGCTAGGCTGCTCTTTC
CCTGCTCAGAACGCCCAAGGGTGGGAAAGAAGGACCTGAAACTGTCAGGCCCACACACCC
TGATCCCAGGGCCAAGGCAGATACAGCCTTCACTGGGAGAAGGCACCTGTGGGTGCCCTG
CCCTGACCCAGCAATGAAGACATTGCAGAGACAAAGTCAGAAGGAATTGTCCCACTAGTG
GGAACAACATAGCATACACTGCCTATGAGGTCCACTCAAGGAGGGCTTCCAGAAGGAGGT
AAAGCTAGACCCCGCCCTTCCACATGTGGGGTAGGCATAGGATGTTGAGACTGTAAGAGA
CATCTCTTTGGCCCTCCTTGTATAGGGTGTCAATCGGCACAACAGGGTGGAGCCTTAGAG
TAGGGTAAGATTAGGACTCTAGGTTCTCTCATGGGTCCAGATCTGTCATGAAGGGAGGTC
AAGGACCCACCTCCCTCCAAAGGCTATGGTGGGGGCATCATGGACCCCAAGGTCCTGCCG
CAGGGCCTGGAGCAGCACAGCAAAGTCGGC

>240 Get primers
CAGCGCCTGCTCCACAGTCAGCAGCTGTGTATATCCCCGCTGTGTGGACTGGACACCGAA
CGGAAGCGATTTCCCATAGTACCGCTGCAGAAAGCAGGAAGGGATGGCTAATCCACTCCT
CGGTGCTCCCCACCTCCTTCAACTCAGGGACTGCCAGGAACTGTACAGGTACCCACGTGC
TCAGCAAAGACAAGCAGGGCCTCCTGCTGGGCTGCCAGTTCCACCATGAAGCCAGAGTTG
TTAGCGAAGGACCAGATATCCCCCTCATTCCCTGTGTAGAAAAAGATGGGCCCTTCGCCC
ATCTTCCAGAACTTATCTGTTGGAAGTAAATGAGTTTCCATAAGGCCAGGGAAACGCAGG
TAGGAACCCATGCGGTCGAGCCAGCACTCACCTGACACTAGGAACCGCTGGCCAAAGGTT
TTGTTGCCGAAACTCTCAAAGTTGAAATGGTCCATGTATTGCTCAAAATAATTCTCATGA
AAGTCAGGGTCTAGAACTCTGTCGGCTGAGGGCAGGTGCAGAGACTCAGGAGCTGGTTGG
GATCATCAGGGATCTAGGCGGGTCAGGAGGAAGGGCAGCCAGTCTGTACTCACCTCTGGC
CTGGAGGTTGCACAGTCCCAGTGACAGCAGCAGGACCAGGATCCAGGAGGGGACACCATG
GTCCACAGGGTAACAAGGATGGAAGTTCATGCTTGATTCTGAGCCGGGCGCTGACTGTCA
TGTGATTTGGTCACATGACCGACACAACGG

>241 Get primers
ATAGTACCGCTGCAGAAAGCAGGAAGGGATGGCTAATCCACTCCTCGGTGCTCCCCACCT
CCTTCAACTCAGGGACTGCCAGGAACTGTACAGGTACCCACGTGCTCAGCAAAGACAAGC
AGGGCCTCCTGCTGGGCTGCCAGTTCCACCATGAAGCCAGAGTTGTTAGCGAAGGACCAG
ATATCCCCCTCATTCCCTGTGTAGAAAAAGATGGGCCCTTCGCCCATCTTCCAGAACTTA
TCTGTTGGAAGTAAATGAGTTTCCATAAGGCCAGGGAAACGCAGGTAGGAACCCATGCGG
TCGAGCCAGCACTCACCTGACACTAGGAACCGCTGGCCAAAGGTTTTGTTGCCGAAACTC
TCAAAGTTGAAATGGTCCATGTATTGCTCAAAATAATTCTCATGAAAGTCAGGGTCTAGA
ACTCTGTCGGCTGAGGGCAGGTGCAGAGACTCAGGAGCTGGTTGGGATCATCAGGGATCT
AGGCGGGTCAGGAGGAAGGGCAGCCAGTCTGTACTCACCTCTGGCCTGGAGGTTGCACAG
TCCCAGTGACAGCAGCAGGACCAGGATCCAGGAGGGGACACCATGGTCCACAGGGTAACA
AGGATGGAAGTTCATGCTTGATTCTGAGCCGGGCGCTGACTGTCATGTGATTTGGTCACA
TGACCGACACAACGGGCGGGGCAGCATCACGTGATAGTCTGGCGGGGGCTGTCCTACTGT
GGCTGGATTCTAGTTGGAGGATCAGCCTAC

>242 Get primers
CTGCCAGGAACTGTACAGGTACCCACGTGCTCAGCAAAGACAAGCAGGGCCTCCTGCTGG
GCTGCCAGTTCCACCATGAAGCCAGAGTTGTTAGCGAAGGACCAGATATCCCCCTCATTC
CCTGTGTAGAAAAAGATGGGCCCTTCGCCCATCTTCCAGAACTTATCTGTTGGAAGTAAA
TGAGTTTCCATAAGGCCAGGGAAACGCAGGTAGGAACCCATGCGGTCGAGCCAGCACTCA
CCTGACACTAGGAACCGCTGGCCAAAGGTTTTGTTGCCGAAACTCTCAAAGTTGAAATGG
TCCATGTATTGCTCAAAATAATTCTCATGAAAGTCAGGGTCTAGAACTCTGTCGGCTGAG
GGCAGGTGCAGAGACTCAGGAGCTGGTTGGGATCATCAGGGATCTAGGCGGGTCAGGAGG
AAGGGCAGCCAGTCTGTACTCACCTCTGGCCTGGAGGTTGCACAGTCCCAGTGACAGCAG
CAGGACCAGGATCCAGGAGGGGACACCATGGTCCACAGGGTAACAAGGATGGAAGTTCAT
GCTTGATTCTGAGCCGGGCGCTGACTGTCATGTGATTTGGTCACATGACCGACACAACGG
GCGGGGCAGCATCACGTGATAGTCTGGCGGGGGCTGTCCTACTGTGGCTGGATTCTAGTT
GGAGGATCAGCCTACTCTTCTTCAGTTTCCCGGTTCCTCCAAATTTCTGGGCTCCTACTT
GTTTCCACAGAGATGGATACTGTGGAGGTC

>243 Get primers
ATGAAGCCAGAGTTGTTAGCGAAGGACCAGATATCCCCCTCATTCCCTGTGTAGAAAAAG
ATGGGCCCTTCGCCCATCTTCCAGAACTTATCTGTTGGAAGTAAATGAGTTTCCATAAGG
CCAGGGAAACGCAGGTAGGAACCCATGCGGTCGAGCCAGCACTCACCTGACACTAGGAAC
CGCTGGCCAAAGGTTTTGTTGCCGAAACTCTCAAAGTTGAAATGGTCCATGTATTGCTCA
AAATAATTCTCATGAAAGTCAGGGTCTAGAACTCTGTCGGCTGAGGGCAGGTGCAGAGAC
TCAGGAGCTGGTTGGGATCATCAGGGATCTAGGCGGGTCAGGAGGAAGGGCAGCCAGTCT
GTACTCACCTCTGGCCTGGAGGTTGCACAGTCCCAGTGACAGCAGCAGGACCAGGATCCA
GGAGGGGACACCATGGTCCACAGGGTAACAAGGATGGAAGTTCATGCTTGATTCTGAGCC
GGGCGCTGACTGTCATGTGATTTGGTCACATGACCGACACAACGGGCGGGGCAGCATCAC
GTGATAGTCTGGCGGGGGCTGTCCTACTGTGGCTGGATTCTAGTTGGAGGATCAGCCTAC
TCTTCTTCAGTTTCCCGGTTCCTCCAAATTTCTGGGCTCCTACTTGTTTCCACAGAGATG
GATACTGTGGAGGTCCAGGAAGCAGAGAGATGGCTAAGGCTCATCAGGACCGTATGATCT
CCCAAGTGTCCAGCTACTGAGTACCACAAG

>244 Get primers
ATCTTCCAGAACTTATCTGTTGGAAGTAAATGAGTTTCCATAAGGCCAGGGAAACGCAGG
TAGGAACCCATGCGGTCGAGCCAGCACTCACCTGACACTAGGAACCGCTGGCCAAAGGTT
TTGTTGCCGAAACTCTCAAAGTTGAAATGGTCCATGTATTGCTCAAAATAATTCTCATGA
AAGTCAGGGTCTAGAACTCTGTCGGCTGAGGGCAGGTGCAGAGACTCAGGAGCTGGTTGG
GATCATCAGGGATCTAGGCGGGTCAGGAGGAAGGGCAGCCAGTCTGTACTCACCTCTGGC
CTGGAGGTTGCACAGTCCCAGTGACAGCAGCAGGACCAGGATCCAGGAGGGGACACCATG
GTCCACAGGGTAACAAGGATGGAAGTTCATGCTTGATTCTGAGCCGGGCGCTGACTGTCA
TGTGATTTGGTCACATGACCGACACAACGGGCGGGGCAGCATCACGTGATAGTCTGGCGG
GGGCTGTCCTACTGTGGCTGGATTCTAGTTGGAGGATCAGCCTACTCTTCTTCAGTTTCC
CGGTTCCTCCAAATTTCTGGGCTCCTACTTGTTTCCACAGAGATGGATACTGTGGAGGTC
CAGGAAGCAGAGAGATGGCTAAGGCTCATCAGGACCGTATGATCTCCCAAGTGTCCAGCT
ACTGAGTACCACAAGGTGATGGGTGGGAGGGTCCTCCCACGGAAGGATACCGCAGTCCCT
AGGGGTTGCAAGCCCCACATGTTCCACTGG

>245 Get primers
TCGAGCCAGCACTCACCTGACACTAGGAACCGCTGGCCAAAGGTTTTGTTGCCGAAACTC
TCAAAGTTGAAATGGTCCATGTATTGCTCAAAATAATTCTCATGAAAGTCAGGGTCTAGA
ACTCTGTCGGCTGAGGGCAGGTGCAGAGACTCAGGAGCTGGTTGGGATCATCAGGGATCT
AGGCGGGTCAGGAGGAAGGGCAGCCAGTCTGTACTCACCTCTGGCCTGGAGGTTGCACAG
TCCCAGTGACAGCAGCAGGACCAGGATCCAGGAGGGGACACCATGGTCCACAGGGTAACA
AGGATGGAAGTTCATGCTTGATTCTGAGCCGGGCGCTGACTGTCATGTGATTTGGTCACA
TGACCGACACAACGGGCGGGGCAGCATCACGTGATAGTCTGGCGGGGGCTGTCCTACTGT
GGCTGGATTCTAGTTGGAGGATCAGCCTACTCTTCTTCAGTTTCCCGGTTCCTCCAAATT
TCTGGGCTCCTACTTGTTTCCACAGAGATGGATACTGTGGAGGTCCAGGAAGCAGAGAGA
TGGCTAAGGCTCATCAGGACCGTATGATCTCCCAAGTGTCCAGCTACTGAGTACCACAAG
GTGATGGGTGGGAGGGTCCTCCCACGGAAGGATACCGCAGTCCCTAGGGGTTGCAAGCCC
CACATGTTCCACTGGCTGCTAGAGCTACCTACTCAATCAGCCCTGGGCATCACCATCAGG
TACTCGGCCAAAATGACCTCTCTGCTTCCA

>246 Get primers
TCCATGTATTGCTCAAAATAATTCTCATGAAAGTCAGGGTCTAGAACTCTGTCGGCTGAG
GGCAGGTGCAGAGACTCAGGAGCTGGTTGGGATCATCAGGGATCTAGGCGGGTCAGGAGG
AAGGGCAGCCAGTCTGTACTCACCTCTGGCCTGGAGGTTGCACAGTCCCAGTGACAGCAG
CAGGACCAGGATCCAGGAGGGGACACCATGGTCCACAGGGTAACAAGGATGGAAGTTCAT
GCTTGATTCTGAGCCGGGCGCTGACTGTCATGTGATTTGGTCACATGACCGACACAACGG
GCGGGGCAGCATCACGTGATAGTCTGGCGGGGGCTGTCCTACTGTGGCTGGATTCTAGTT
GGAGGATCAGCCTACTCTTCTTCAGTTTCCCGGTTCCTCCAAATTTCTGGGCTCCTACTT
GTTTCCACAGAGATGGATACTGTGGAGGTCCAGGAAGCAGAGAGATGGCTAAGGCTCATC
AGGACCGTATGATCTCCCAAGTGTCCAGCTACTGAGTACCACAAGGTGATGGGTGGGAGG
GTCCTCCCACGGAAGGATACCGCAGTCCCTAGGGGTTGCAAGCCCCACATGTTCCACTGG
CTGCTAGAGCTACCTACTCAATCAGCCCTGGGCATCACCATCAGGTACTCGGCCAAAATG
ACCTCTCTGCTTCCAGTCCTCAGTTCTGGTCAGCACCAGACAGGCCCATAATTACAGAGC
CAGGGAAACTGGAACATTTGTCTCCCCTTA

>247 Get primers
TCAGGAGCTGGTTGGGATCATCAGGGATCTAGGCGGGTCAGGAGGAAGGGCAGCCAGTCT
GTACTCACCTCTGGCCTGGAGGTTGCACAGTCCCAGTGACAGCAGCAGGACCAGGATCCA
GGAGGGGACACCATGGTCCACAGGGTAACAAGGATGGAAGTTCATGCTTGATTCTGAGCC
GGGCGCTGACTGTCATGTGATTTGGTCACATGACCGACACAACGGGCGGGGCAGCATCAC
GTGATAGTCTGGCGGGGGCTGTCCTACTGTGGCTGGATTCTAGTTGGAGGATCAGCCTAC
TCTTCTTCAGTTTCCCGGTTCCTCCAAATTTCTGGGCTCCTACTTGTTTCCACAGAGATG
GATACTGTGGAGGTCCAGGAAGCAGAGAGATGGCTAAGGCTCATCAGGACCGTATGATCT
CCCAAGTGTCCAGCTACTGAGTACCACAAGGTGATGGGTGGGAGGGTCCTCCCACGGAAG
GATACCGCAGTCCCTAGGGGTTGCAAGCCCCACATGTTCCACTGGCTGCTAGAGCTACCT
ACTCAATCAGCCCTGGGCATCACCATCAGGTACTCGGCCAAAATGACCTCTCTGCTTCCA
GTCCTCAGTTCTGGTCAGCACCAGACAGGCCCATAATTACAGAGCCAGGGAAACTGGAAC
ATTTGTCTCCCCTTAGACAGTGGCAGCAGGAAGGTGGGGGGTTGTTGCAGAGGAACAGTG
TCTCTGAGAGAGGACCTTGGACTTTCTGGG

>248 Get primers
CTGGAGGTTGCACAGTCCCAGTGACAGCAGCAGGACCAGGATCCAGGAGGGGACACCATG
GTCCACAGGGTAACAAGGATGGAAGTTCATGCTTGATTCTGAGCCGGGCGCTGACTGTCA
TGTGATTTGGTCACATGACCGACACAACGGGCGGGGCAGCATCACGTGATAGTCTGGCGG
GGGCTGTCCTACTGTGGCTGGATTCTAGTTGGAGGATCAGCCTACTCTTCTTCAGTTTCC
CGGTTCCTCCAAATTTCTGGGCTCCTACTTGTTTCCACAGAGATGGATACTGTGGAGGTC
CAGGAAGCAGAGAGATGGCTAAGGCTCATCAGGACCGTATGATCTCCCAAGTGTCCAGCT
ACTGAGTACCACAAGGTGATGGGTGGGAGGGTCCTCCCACGGAAGGATACCGCAGTCCCT
AGGGGTTGCAAGCCCCACATGTTCCACTGGCTGCTAGAGCTACCTACTCAATCAGCCCTG
GGCATCACCATCAGGTACTCGGCCAAAATGACCTCTCTGCTTCCAGTCCTCAGTTCTGGT
CAGCACCAGACAGGCCCATAATTACAGAGCCAGGGAAACTGGAACATTTGTCTCCCCTTA
GACAGTGGCAGCAGGAAGGTGGGGGGTTGTTGCAGAGGAACAGTGTCTCTGAGAGAGGAC
CTTGGACTTTCTGGGAATCTCTGAGCTGCCCGGTTCTCCCCACTGCTGGCACTGTGCCCA
CAGCCCAAACAGAATGGGGGAGATGGAGGG

>249 Get primers
AGGATGGAAGTTCATGCTTGATTCTGAGCCGGGCGCTGACTGTCATGTGATTTGGTCACA
TGACCGACACAACGGGCGGGGCAGCATCACGTGATAGTCTGGCGGGGGCTGTCCTACTGT
GGCTGGATTCTAGTTGGAGGATCAGCCTACTCTTCTTCAGTTTCCCGGTTCCTCCAAATT
TCTGGGCTCCTACTTGTTTCCACAGAGATGGATACTGTGGAGGTCCAGGAAGCAGAGAGA
TGGCTAAGGCTCATCAGGACCGTATGATCTCCCAAGTGTCCAGCTACTGAGTACCACAAG
GTGATGGGTGGGAGGGTCCTCCCACGGAAGGATACCGCAGTCCCTAGGGGTTGCAAGCCC
CACATGTTCCACTGGCTGCTAGAGCTACCTACTCAATCAGCCCTGGGCATCACCATCAGG
TACTCGGCCAAAATGACCTCTCTGCTTCCAGTCCTCAGTTCTGGTCAGCACCAGACAGGC
CCATAATTACAGAGCCAGGGAAACTGGAACATTTGTCTCCCCTTAGACAGTGGCAGCAGG
AAGGTGGGGGGTTGTTGCAGAGGAACAGTGTCTCTGAGAGAGGACCTTGGACTTTCTGGG
AATCTCTGAGCTGCCCGGTTCTCCCCACTGCTGGCACTGTGCCCACAGCCCAAACAGAAT
GGGGGAGATGGAGGGGCAGGGCTTCTGTGGGAAGCTGCCCTCCACCTCATTGGCACAGAG
TGTCTCATTGCAGAGAGAAAAAAGGACCAG

>250 Get primers
GCGGGGCAGCATCACGTGATAGTCTGGCGGGGGCTGTCCTACTGTGGCTGGATTCTAGTT
GGAGGATCAGCCTACTCTTCTTCAGTTTCCCGGTTCCTCCAAATTTCTGGGCTCCTACTT
GTTTCCACAGAGATGGATACTGTGGAGGTCCAGGAAGCAGAGAGATGGCTAAGGCTCATC
AGGACCGTATGATCTCCCAAGTGTCCAGCTACTGAGTACCACAAGGTGATGGGTGGGAGG
GTCCTCCCACGGAAGGATACCGCAGTCCCTAGGGGTTGCAAGCCCCACATGTTCCACTGG
CTGCTAGAGCTACCTACTCAATCAGCCCTGGGCATCACCATCAGGTACTCGGCCAAAATG
ACCTCTCTGCTTCCAGTCCTCAGTTCTGGTCAGCACCAGACAGGCCCATAATTACAGAGC
CAGGGAAACTGGAACATTTGTCTCCCCTTAGACAGTGGCAGCAGGAAGGTGGGGGGTTGT
TGCAGAGGAACAGTGTCTCTGAGAGAGGACCTTGGACTTTCTGGGAATCTCTGAGCTGCC
CGGTTCTCCCCACTGCTGGCACTGTGCCCACAGCCCAAACAGAATGGGGGAGATGGAGGG
GCAGGGCTTCTGTGGGAAGCTGCCCTCCACCTCATTGGCACAGAGTGTCTCATTGCAGAG
AGAAAAAAGGACCAGTTTTCTCTCTGGCACCCAGGTCTGGAAGAGGAGTGACATCCACGG
AAGTTGGTGACTTGGACTGGCTGGCCGTGA

>251 Get primers
TCTTCTTCAGTTTCCCGGTTCCTCCAAATTTCTGGGCTCCTACTTGTTTCCACAGAGATG
GATACTGTGGAGGTCCAGGAAGCAGAGAGATGGCTAAGGCTCATCAGGACCGTATGATCT
CCCAAGTGTCCAGCTACTGAGTACCACAAGGTGATGGGTGGGAGGGTCCTCCCACGGAAG
GATACCGCAGTCCCTAGGGGTTGCAAGCCCCACATGTTCCACTGGCTGCTAGAGCTACCT
ACTCAATCAGCCCTGGGCATCACCATCAGGTACTCGGCCAAAATGACCTCTCTGCTTCCA
GTCCTCAGTTCTGGTCAGCACCAGACAGGCCCATAATTACAGAGCCAGGGAAACTGGAAC
ATTTGTCTCCCCTTAGACAGTGGCAGCAGGAAGGTGGGGGGTTGTTGCAGAGGAACAGTG
TCTCTGAGAGAGGACCTTGGACTTTCTGGGAATCTCTGAGCTGCCCGGTTCTCCCCACTG
CTGGCACTGTGCCCACAGCCCAAACAGAATGGGGGAGATGGAGGGGCAGGGCTTCTGTGG
GAAGCTGCCCTCCACCTCATTGGCACAGAGTGTCTCATTGCAGAGAGAAAAAAGGACCAG
TTTTCTCTCTGGCACCCAGGTCTGGAAGAGGAGTGACATCCACGGAAGTTGGTGACTTGG
ACTGGCTGGCCGTGAGTGGAACATGTCCATCCAGCATGGCCACAGTCCAGTGGGACACAC
AGCCTAGAGCTGTGGAATGCCGTGCCACAG

>252 Get primers
AGGCCAAGAAGTAGACCCACCTGCCAGTGCCCGTAGACCCATGATCCTCTGGCCCTCATT
CAACAGCCGTTGACAGCCCACCTAGAGAAAGGCAGCAGAATATCTCAGTGGAGGCCCCTT
TCACAGAGCGTGGGTCAGGGCTGCTAGCTTCCAGGACACAACAGCAGATAGTGTCTGATG
GCATGAAAGCAGATAGCTACAAGGCTCTTGGACCAGGCTAGCACTGGGTCCTGCACCCAG
GGAGAGCCACCTCACCTTGACAGGGTTGGCAGGAAGGGGCCCTAGAAAGTCAGTAGGATA
CGGGTAGTCCATCATGGCGAGCACAGTAAATGCATTTCGGGCAAACCCAAAGAGCTGAGT
CAGGTCCTTTGGGCTGGAAAGTGATTGACAGGTACCAAAGTTCTGGCTGATGGTGTCATA
GGCTGGGAAGAGAGAGGCCAGGAGAAAAGGCTGAGGAAACTGCTGGCAAATGTGAAGGGC
AAGAATGAATGCCCAAGGTGGGCAGCAGGTGAGGAAAGAGTCCCTCACCTCCCTGGAGGA
ACAAGTCTTTGATTTGCTGAAAGGCATCCCGCACAGCCTGGGCGCACTTGGGACTCTGGC
CATAAAAGTCCTGGAGAAGAGACCAAGGTTGCTGCTGCCATTCTTGCACTGGCCTGGGGT
ACCCAAGTCCCCTCACTCACCGCTGTGACATCTCGGAAGA

>253 Get primers
TGACAGCCCACCTAGAGAAAGGCAGCAGAATATCTCAGTGGAGGCCCCTTTCACAGAGCG
TGGGTCAGGGCTGCTAGCTTCCAGGACACAACAGCAGATAGTGTCTGATGGCATGAAAGC
AGATAGCTACAAGGCTCTTGGACCAGGCTAGCACTGGGTCCTGCACCCAGGGAGAGCCAC
CTCACCTTGACAGGGTTGGCAGGAAGGGGCCCTAGAAAGTCAGTAGGATACGGGTAGTCC
ATCATGGCGAGCACAGTAAATGCATTTCGGGCAAACCCAAAGAGCTGAGTCAGGTCCTTT
GGGCTGGAAAGTGATTGACAGGTACCAAAGTTCTGGCTGATGGTGTCATAGGCTGGGAAG
AGAGAGGCCAGGAGAAAAGGCTGAGGAAACTGCTGGCAAATGTGAAGGGCAAGAATGAAT
GCCCAAGGTGGGCAGCAGGTGAGGAAAGAGTCCCTCACCTCCCTGGAGGAACAAGTCTTT
GATTTGCTGAAAGGCATCCCGCACAGCCTGGGCGCACTTGGGACTCTGGCCATAAAAGTC
CTGGAGAAGAGACCAAGGTTGCTGCTGCCATTCTTGCACTGGCCTGGGGTACCCAAGTCC
CCTCACTCACCGCTGTGACATCTCGGAAGAATTGGTAGGAGTCCCCAAGGCCTGCAACAG
CTACAACAGGAGCGCTGGCTGCCAGTGCCCCAGCCACCAG

>254 Get primers
CTGCTAGCTTCCAGGACACAACAGCAGATAGTGTCTGATGGCATGAAAGCAGATAGCTAC
AAGGCTCTTGGACCAGGCTAGCACTGGGTCCTGCACCCAGGGAGAGCCACCTCACCTTGA
CAGGGTTGGCAGGAAGGGGCCCTAGAAAGTCAGTAGGATACGGGTAGTCCATCATGGCGA
GCACAGTAAATGCATTTCGGGCAAACCCAAAGAGCTGAGTCAGGTCCTTTGGGCTGGAAA
GTGATTGACAGGTACCAAAGTTCTGGCTGATGGTGTCATAGGCTGGGAAGAGAGAGGCCA
GGAGAAAAGGCTGAGGAAACTGCTGGCAAATGTGAAGGGCAAGAATGAATGCCCAAGGTG
GGCAGCAGGTGAGGAAAGAGTCCCTCACCTCCCTGGAGGAACAAGTCTTTGATTTGCTGA
AAGGCATCCCGCACAGCCTGGGCGCACTTGGGACTCTGGCCATAAAAGTCCTGGAGAAGA
GACCAAGGTTGCTGCTGCCATTCTTGCACTGGCCTGGGGTACCCAAGTCCCCTCACTCAC
CGCTGTGACATCTCGGAAGAATTGGTAGGAGTCCCCAAGGCCTGCAACAGCTACAACAGG
AGCGCTGGCTGCCAGTGCCCCAGCCACCAGGTGGGGGTACTTCATCCTCATGTAGGCACT
CAGCATCCCCCCATAACTGGGAGTACAGAGCACAGATCAT

>255 Get primers
GACCAGGCTAGCACTGGGTCCTGCACCCAGGGAGAGCCACCTCACCTTGACAGGGTTGGC
AGGAAGGGGCCCTAGAAAGTCAGTAGGATACGGGTAGTCCATCATGGCGAGCACAGTAAA
TGCATTTCGGGCAAACCCAAAGAGCTGAGTCAGGTCCTTTGGGCTGGAAAGTGATTGACA
GGTACCAAAGTTCTGGCTGATGGTGTCATAGGCTGGGAAGAGAGAGGCCAGGAGAAAAGG
CTGAGGAAACTGCTGGCAAATGTGAAGGGCAAGAATGAATGCCCAAGGTGGGCAGCAGGT
GAGGAAAGAGTCCCTCACCTCCCTGGAGGAACAAGTCTTTGATTTGCTGAAAGGCATCCC
GCACAGCCTGGGCGCACTTGGGACTCTGGCCATAAAAGTCCTGGAGAAGAGACCAAGGTT
GCTGCTGCCATTCTTGCACTGGCCTGGGGTACCCAAGTCCCCTCACTCACCGCTGTGACA
TCTCGGAAGAATTGGTAGGAGTCCCCAAGGCCTGCAACAGCTACAACAGGAGCGCTGGCT
GCCAGTGCCCCAGCCACCAGGTGGGGGTACTTCATCCTCATGTAGGCACTCAGCATCCCC
CCATAACTGGGAGTACAGAGCACAGATCATGGTTGTGGGAAGCTGCCCACAACTCAGGCG
AGCAGCCTCACTGTCCTCCAGGCTGAGGTGCTAGGCTGCT

>256 Get primers
CCTAGAAAGTCAGTAGGATACGGGTAGTCCATCATGGCGAGCACAGTAAATGCATTTCGG
GCAAACCCAAAGAGCTGAGTCAGGTCCTTTGGGCTGGAAAGTGATTGACAGGTACCAAAG
TTCTGGCTGATGGTGTCATAGGCTGGGAAGAGAGAGGCCAGGAGAAAAGGCTGAGGAAAC
TGCTGGCAAATGTGAAGGGCAAGAATGAATGCCCAAGGTGGGCAGCAGGTGAGGAAAGAG
TCCCTCACCTCCCTGGAGGAACAAGTCTTTGATTTGCTGAAAGGCATCCCGCACAGCCTG
GGCGCACTTGGGACTCTGGCCATAAAAGTCCTGGAGAAGAGACCAAGGTTGCTGCTGCCA
TTCTTGCACTGGCCTGGGGTACCCAAGTCCCCTCACTCACCGCTGTGACATCTCGGAAGA
ATTGGTAGGAGTCCCCAAGGCCTGCAACAGCTACAACAGGAGCGCTGGCTGCCAGTGCCC
CAGCCACCAGGTGGGGGTACTTCATCCTCATGTAGGCACTCAGCATCCCCCCATAACTGG
GAGTACAGAGCACAGATCATGGTTGTGGGAAGCTGCCCACAACTCAGGCGAGCAGCCTCA
CTGTCCTCCAGGCTGAGGTGCTAGGCTGCTCTTTCCCTGCTCAGAACGCCCAAGGGTGGG
AAAGAAGGACCTGAAACTGTCAGGCCCACACACCCTGATC

>257 Get primers
AGAGCTGAGTCAGGTCCTTTGGGCTGGAAAGTGATTGACAGGTACCAAAGTTCTGGCTGA
TGGTGTCATAGGCTGGGAAGAGAGAGGCCAGGAGAAAAGGCTGAGGAAACTGCTGGCAAA
TGTGAAGGGCAAGAATGAATGCCCAAGGTGGGCAGCAGGTGAGGAAAGAGTCCCTCACCT
CCCTGGAGGAACAAGTCTTTGATTTGCTGAAAGGCATCCCGCACAGCCTGGGCGCACTTG
GGACTCTGGCCATAAAAGTCCTGGAGAAGAGACCAAGGTTGCTGCTGCCATTCTTGCACT
GGCCTGGGGTACCCAAGTCCCCTCACTCACCGCTGTGACATCTCGGAAGAATTGGTAGGA
GTCCCCAAGGCCTGCAACAGCTACAACAGGAGCGCTGGCTGCCAGTGCCCCAGCCACCAG
GTGGGGGTACTTCATCCTCATGTAGGCACTCAGCATCCCCCCATAACTGGGAGTACAGAG
CACAGATCATGGTTGTGGGAAGCTGCCCACAACTCAGGCGAGCAGCCTCACTGTCCTCCA
GGCTGAGGTGCTAGGCTGCTCTTTCCCTGCTCAGAACGCCCAAGGGTGGGAAAGAAGGAC
CTGAAACTGTCAGGCCCACACACCCTGATCCCAGGGCCAAGGCAGATACAGCCTTCACTG
GGAGAAGGCACCTGTGGGTGCCCTGCCCTGACCCAGCAAT

>258 Get primers
GGCTGGGAAGAGAGAGGCCAGGAGAAAAGGCTGAGGAAACTGCTGGCAAATGTGAAGGGC
AAGAATGAATGCCCAAGGTGGGCAGCAGGTGAGGAAAGAGTCCCTCACCTCCCTGGAGGA
ACAAGTCTTTGATTTGCTGAAAGGCATCCCGCACAGCCTGGGCGCACTTGGGACTCTGGC
CATAAAAGTCCTGGAGAAGAGACCAAGGTTGCTGCTGCCATTCTTGCACTGGCCTGGGGT
ACCCAAGTCCCCTCACTCACCGCTGTGACATCTCGGAAGAATTGGTAGGAGTCCCCAAGG
CCTGCAACAGCTACAACAGGAGCGCTGGCTGCCAGTGCCCCAGCCACCAGGTGGGGGTAC
TTCATCCTCATGTAGGCACTCAGCATCCCCCCATAACTGGGAGTACAGAGCACAGATCAT
GGTTGTGGGAAGCTGCCCACAACTCAGGCGAGCAGCCTCACTGTCCTCCAGGCTGAGGTG
CTAGGCTGCTCTTTCCCTGCTCAGAACGCCCAAGGGTGGGAAAGAAGGACCTGAAACTGT
CAGGCCCACACACCCTGATCCCAGGGCCAAGGCAGATACAGCCTTCACTGGGAGAAGGCA
CCTGTGGGTGCCCTGCCCTGACCCAGCAATGAAGACATTGCAGAGACAAAGTCAGAAGGA
ATTGTCCCACTAGTGGGAACAACATAGCATACACTGCCTA

>259 Get primers
GCCCAAGGTGGGCAGCAGGTGAGGAAAGAGTCCCTCACCTCCCTGGAGGAACAAGTCTTT
GATTTGCTGAAAGGCATCCCGCACAGCCTGGGCGCACTTGGGACTCTGGCCATAAAAGTC
CTGGAGAAGAGACCAAGGTTGCTGCTGCCATTCTTGCACTGGCCTGGGGTACCCAAGTCC
CCTCACTCACCGCTGTGACATCTCGGAAGAATTGGTAGGAGTCCCCAAGGCCTGCAACAG
CTACAACAGGAGCGCTGGCTGCCAGTGCCCCAGCCACCAGGTGGGGGTACTTCATCCTCA
TGTAGGCACTCAGCATCCCCCCATAACTGGGAGTACAGAGCACAGATCATGGTTGTGGGA
AGCTGCCCACAACTCAGGCGAGCAGCCTCACTGTCCTCCAGGCTGAGGTGCTAGGCTGCT
CTTTCCCTGCTCAGAACGCCCAAGGGTGGGAAAGAAGGACCTGAAACTGTCAGGCCCACA
CACCCTGATCCCAGGGCCAAGGCAGATACAGCCTTCACTGGGAGAAGGCACCTGTGGGTG
CCCTGCCCTGACCCAGCAATGAAGACATTGCAGAGACAAAGTCAGAAGGAATTGTCCCAC
TAGTGGGAACAACATAGCATACACTGCCTATGAGGTCCACTCAAGGAGGGCTTCCAGAAG
GAGGTAAAGCTAGACCCCGCCCTTCCACATGTGGGGTAGG

>260 Get primers
AAGGCATCCCGCACAGCCTGGGCGCACTTGGGACTCTGGCCATAAAAGTCCTGGAGAAGA
GACCAAGGTTGCTGCTGCCATTCTTGCACTGGCCTGGGGTACCCAAGTCCCCTCACTCAC
CGCTGTGACATCTCGGAAGAATTGGTAGGAGTCCCCAAGGCCTGCAACAGCTACAACAGG
AGCGCTGGCTGCCAGTGCCCCAGCCACCAGGTGGGGGTACTTCATCCTCATGTAGGCACT
CAGCATCCCCCCATAACTGGGAGTACAGAGCACAGATCATGGTTGTGGGAAGCTGCCCAC
AACTCAGGCGAGCAGCCTCACTGTCCTCCAGGCTGAGGTGCTAGGCTGCTCTTTCCCTGC
TCAGAACGCCCAAGGGTGGGAAAGAAGGACCTGAAACTGTCAGGCCCACACACCCTGATC
CCAGGGCCAAGGCAGATACAGCCTTCACTGGGAGAAGGCACCTGTGGGTGCCCTGCCCTG
ACCCAGCAATGAAGACATTGCAGAGACAAAGTCAGAAGGAATTGTCCCACTAGTGGGAAC
AACATAGCATACACTGCCTATGAGGTCCACTCAAGGAGGGCTTCCAGAAGGAGGTAAAGC
TAGACCCCGCCCTTCCACATGTGGGGTAGGCATAGGATGTTGAGACTGTAAGAGACATCT
CTTTGGCCCTCCTTGTATAGGGTGTCAATCGGCACAACAG

>261 Get primers
GCTGCTGCCATTCTTGCACTGGCCTGGGGTACCCAAGTCCCCTCACTCACCGCTGTGACA
TCTCGGAAGAATTGGTAGGAGTCCCCAAGGCCTGCAACAGCTACAACAGGAGCGCTGGCT
GCCAGTGCCCCAGCCACCAGGTGGGGGTACTTCATCCTCATGTAGGCACTCAGCATCCCC
CCATAACTGGGAGTACAGAGCACAGATCATGGTTGTGGGAAGCTGCCCACAACTCAGGCG
AGCAGCCTCACTGTCCTCCAGGCTGAGGTGCTAGGCTGCTCTTTCCCTGCTCAGAACGCC
CAAGGGTGGGAAAGAAGGACCTGAAACTGTCAGGCCCACACACCCTGATCCCAGGGCCAA
GGCAGATACAGCCTTCACTGGGAGAAGGCACCTGTGGGTGCCCTGCCCTGACCCAGCAAT
GAAGACATTGCAGAGACAAAGTCAGAAGGAATTGTCCCACTAGTGGGAACAACATAGCAT
ACACTGCCTATGAGGTCCACTCAAGGAGGGCTTCCAGAAGGAGGTAAAGCTAGACCCCGC
CCTTCCACATGTGGGGTAGGCATAGGATGTTGAGACTGTAAGAGACATCTCTTTGGCCCT
CCTTGTATAGGGTGTCAATCGGCACAACAGGGTGGAGCCTTAGAGTAGGGTAAGATTAGG
ACTCTAGGTTCTCTCATGGGTCCAGATCTGTCATGAAGGG

>262 Get primers
ATTGGTAGGAGTCCCCAAGGCCTGCAACAGCTACAACAGGAGCGCTGGCTGCCAGTGCCC
CAGCCACCAGGTGGGGGTACTTCATCCTCATGTAGGCACTCAGCATCCCCCCATAACTGG
GAGTACAGAGCACAGATCATGGTTGTGGGAAGCTGCCCACAACTCAGGCGAGCAGCCTCA
CTGTCCTCCAGGCTGAGGTGCTAGGCTGCTCTTTCCCTGCTCAGAACGCCCAAGGGTGGG
AAAGAAGGACCTGAAACTGTCAGGCCCACACACCCTGATCCCAGGGCCAAGGCAGATACA
GCCTTCACTGGGAGAAGGCACCTGTGGGTGCCCTGCCCTGACCCAGCAATGAAGACATTG
CAGAGACAAAGTCAGAAGGAATTGTCCCACTAGTGGGAACAACATAGCATACACTGCCTA
TGAGGTCCACTCAAGGAGGGCTTCCAGAAGGAGGTAAAGCTAGACCCCGCCCTTCCACAT
GTGGGGTAGGCATAGGATGTTGAGACTGTAAGAGACATCTCTTTGGCCCTCCTTGTATAG
GGTGTCAATCGGCACAACAGGGTGGAGCCTTAGAGTAGGGTAAGATTAGGACTCTAGGTT
CTCTCATGGGTCCAGATCTGTCATGAAGGGAGGTCAAGGACCCACCTCCCTCCAAAGGCT
ATGGTGGGGGCATCATGGACCCCAAGGTCCTGCCGCAGGG

>272 Get primers
CCTGGAGCAGCACAGCAAAGTCGGCCAGCGCCTGCTCCACAGTCAGCAGCTGTGTATATC
CCCGCTGTGTGGACTGGACACCGAACGGAAGCGATTTCCCATAGTACCGCTGCAGAAAGC
AGGAAGGGATGGCTAATCCACTCCTCGGTGCTCCCCACCTCCTTCAACTCAGGGACTGCC
AGGAACTGTACAGGTACCCACGTGCTCAGCAAAGACAAGCAGGGCCTCCTGCTGGGCTGC
CAGTTCCACCATGAAGCCAGAGTTGTTAGCGAAGGACCAGATATCCCCCTCATTCCCTGT
GTAGAAAAAGATGGGCCCTTCGCCCATCTTCCAGAACTTATCTGTTGGAAGTAAATGAGT
TTCCATAAGGCCAGGGAAACGCAGGTAGGAACCCATGCGGTCGAGCCAGCACTCACCTGA
CACTAGGAACCGCTGGCCAAAGGTTTTGTTGCCGAAACTCTCAAAGTTGAAATGGTCCAT
GTATTGCTCAAAATAATTCTCATGAAAGTCAGGGTCTAGAACTCTGTCGGCTGAGGGCAG
GTGCAGAGACTCAGGAGCTGGTTGGGATCATCAGGGATCTAGGCGGGTCAGGAGGAAGGG
CAGCCAGTCTGTACTCACCTCTGGCCTGGAGGTTGCACAGTCCCAGTGACAGCAGCAGGA
CCAGGATCCAGGAGGGGACACCATGGTCCACAGGGTAACA

>273 Get primers
GGACTGGACACCGAACGGAAGCGATTTCCCATAGTACCGCTGCAGAAAGCAGGAAGGGAT
GGCTAATCCACTCCTCGGTGCTCCCCACCTCCTTCAACTCAGGGACTGCCAGGAACTGTA
CAGGTACCCACGTGCTCAGCAAAGACAAGCAGGGCCTCCTGCTGGGCTGCCAGTTCCACC
ATGAAGCCAGAGTTGTTAGCGAAGGACCAGATATCCCCCTCATTCCCTGTGTAGAAAAAG
ATGGGCCCTTCGCCCATCTTCCAGAACTTATCTGTTGGAAGTAAATGAGTTTCCATAAGG
CCAGGGAAACGCAGGTAGGAACCCATGCGGTCGAGCCAGCACTCACCTGACACTAGGAAC
CGCTGGCCAAAGGTTTTGTTGCCGAAACTCTCAAAGTTGAAATGGTCCATGTATTGCTCA
AAATAATTCTCATGAAAGTCAGGGTCTAGAACTCTGTCGGCTGAGGGCAGGTGCAGAGAC
TCAGGAGCTGGTTGGGATCATCAGGGATCTAGGCGGGTCAGGAGGAAGGGCAGCCAGTCT
GTACTCACCTCTGGCCTGGAGGTTGCACAGTCCCAGTGACAGCAGCAGGACCAGGATCCA
GGAGGGGACACCATGGTCCACAGGGTAACAAGGATGGAAGTTCATGCTTGATTCTGAGCC
GGGCGCTGACTGTCATGTGATTTGGTCACATGACCGACAC

>274 Get primers
CTCCTCGGTGCTCCCCACCTCCTTCAACTCAGGGACTGCCAGGAACTGTACAGGTACCCA
CGTGCTCAGCAAAGACAAGCAGGGCCTCCTGCTGGGCTGCCAGTTCCACCATGAAGCCAG
AGTTGTTAGCGAAGGACCAGATATCCCCCTCATTCCCTGTGTAGAAAAAGATGGGCCCTT
CGCCCATCTTCCAGAACTTATCTGTTGGAAGTAAATGAGTTTCCATAAGGCCAGGGAAAC
GCAGGTAGGAACCCATGCGGTCGAGCCAGCACTCACCTGACACTAGGAACCGCTGGCCAA
AGGTTTTGTTGCCGAAACTCTCAAAGTTGAAATGGTCCATGTATTGCTCAAAATAATTCT
CATGAAAGTCAGGGTCTAGAACTCTGTCGGCTGAGGGCAGGTGCAGAGACTCAGGAGCTG
GTTGGGATCATCAGGGATCTAGGCGGGTCAGGAGGAAGGGCAGCCAGTCTGTACTCACCT
CTGGCCTGGAGGTTGCACAGTCCCAGTGACAGCAGCAGGACCAGGATCCAGGAGGGGACA
CCATGGTCCACAGGGTAACAAGGATGGAAGTTCATGCTTGATTCTGAGCCGGGCGCTGAC
TGTCATGTGATTTGGTCACATGACCGACACAACGGGCGGGGCAGCATCACGTGATAGTCT
GGCGGGGGCTGTCCTACTGTGGCTGGATTCTAGTTGGAGG

>275 Get primers
AAAGACAAGCAGGGCCTCCTGCTGGGCTGCCAGTTCCACCATGAAGCCAGAGTTGTTAGC
GAAGGACCAGATATCCCCCTCATTCCCTGTGTAGAAAAAGATGGGCCCTTCGCCCATCTT
CCAGAACTTATCTGTTGGAAGTAAATGAGTTTCCATAAGGCCAGGGAAACGCAGGTAGGA
ACCCATGCGGTCGAGCCAGCACTCACCTGACACTAGGAACCGCTGGCCAAAGGTTTTGTT
GCCGAAACTCTCAAAGTTGAAATGGTCCATGTATTGCTCAAAATAATTCTCATGAAAGTC
AGGGTCTAGAACTCTGTCGGCTGAGGGCAGGTGCAGAGACTCAGGAGCTGGTTGGGATCA
TCAGGGATCTAGGCGGGTCAGGAGGAAGGGCAGCCAGTCTGTACTCACCTCTGGCCTGGA
GGTTGCACAGTCCCAGTGACAGCAGCAGGACCAGGATCCAGGAGGGGACACCATGGTCCA
CAGGGTAACAAGGATGGAAGTTCATGCTTGATTCTGAGCCGGGCGCTGACTGTCATGTGA
TTTGGTCACATGACCGACACAACGGGCGGGGCAGCATCACGTGATAGTCTGGCGGGGGCT
GTCCTACTGTGGCTGGATTCTAGTTGGAGGATCAGCCTACTCTTCTTCAGTTTCCCGGTT
CCTCCAAATTTCTGGGCTCCTACTTGTTTCCACAGAGATG

>276 Get primers
ATATCCCCCTCATTCCCTGTGTAGAAAAAGATGGGCCCTTCGCCCATCTTCCAGAACTTA
TCTGTTGGAAGTAAATGAGTTTCCATAAGGCCAGGGAAACGCAGGTAGGAACCCATGCGG
TCGAGCCAGCACTCACCTGACACTAGGAACCGCTGGCCAAAGGTTTTGTTGCCGAAACTC
TCAAAGTTGAAATGGTCCATGTATTGCTCAAAATAATTCTCATGAAAGTCAGGGTCTAGA
ACTCTGTCGGCTGAGGGCAGGTGCAGAGACTCAGGAGCTGGTTGGGATCATCAGGGATCT
AGGCGGGTCAGGAGGAAGGGCAGCCAGTCTGTACTCACCTCTGGCCTGGAGGTTGCACAG
TCCCAGTGACAGCAGCAGGACCAGGATCCAGGAGGGGACACCATGGTCCACAGGGTAACA
AGGATGGAAGTTCATGCTTGATTCTGAGCCGGGCGCTGACTGTCATGTGATTTGGTCACA
TGACCGACACAACGGGCGGGGCAGCATCACGTGATAGTCTGGCGGGGGCTGTCCTACTGT
GGCTGGATTCTAGTTGGAGGATCAGCCTACTCTTCTTCAGTTTCCCGGTTCCTCCAAATT
TCTGGGCTCCTACTTGTTTCCACAGAGATGGATACTGTGGAGGTCCAGGAAGCAGAGAGA
TGGCTAAGGCTCATCAGGACCGTATGATCTCCCAAGTGTC

>277 Get primers
GTAAATGAGTTTCCATAAGGCCAGGGAAACGCAGGTAGGAACCCATGCGGTCGAGCCAGC
ACTCACCTGACACTAGGAACCGCTGGCCAAAGGTTTTGTTGCCGAAACTCTCAAAGTTGA
AATGGTCCATGTATTGCTCAAAATAATTCTCATGAAAGTCAGGGTCTAGAACTCTGTCGG
CTGAGGGCAGGTGCAGAGACTCAGGAGCTGGTTGGGATCATCAGGGATCTAGGCGGGTCA
GGAGGAAGGGCAGCCAGTCTGTACTCACCTCTGGCCTGGAGGTTGCACAGTCCCAGTGAC
AGCAGCAGGACCAGGATCCAGGAGGGGACACCATGGTCCACAGGGTAACAAGGATGGAAG
TTCATGCTTGATTCTGAGCCGGGCGCTGACTGTCATGTGATTTGGTCACATGACCGACAC
AACGGGCGGGGCAGCATCACGTGATAGTCTGGCGGGGGCTGTCCTACTGTGGCTGGATTC
TAGTTGGAGGATCAGCCTACTCTTCTTCAGTTTCCCGGTTCCTCCAAATTTCTGGGCTCC
TACTTGTTTCCACAGAGATGGATACTGTGGAGGTCCAGGAAGCAGAGAGATGGCTAAGGC
TCATCAGGACCGTATGATCTCCCAAGTGTCCAGCTACTGAGTACCACAAGGTGATGGGTG
GGAGGGTCCTCCCACGGAAGGATACCGCAGTCCCTAGGGG

>278 Get primers
CACTAGGAACCGCTGGCCAAAGGTTTTGTTGCCGAAACTCTCAAAGTTGAAATGGTCCAT
GTATTGCTCAAAATAATTCTCATGAAAGTCAGGGTCTAGAACTCTGTCGGCTGAGGGCAG
GTGCAGAGACTCAGGAGCTGGTTGGGATCATCAGGGATCTAGGCGGGTCAGGAGGAAGGG
CAGCCAGTCTGTACTCACCTCTGGCCTGGAGGTTGCACAGTCCCAGTGACAGCAGCAGGA
CCAGGATCCAGGAGGGGACACCATGGTCCACAGGGTAACAAGGATGGAAGTTCATGCTTG
ATTCTGAGCCGGGCGCTGACTGTCATGTGATTTGGTCACATGACCGACACAACGGGCGGG
GCAGCATCACGTGATAGTCTGGCGGGGGCTGTCCTACTGTGGCTGGATTCTAGTTGGAGG
ATCAGCCTACTCTTCTTCAGTTTCCCGGTTCCTCCAAATTTCTGGGCTCCTACTTGTTTC
CACAGAGATGGATACTGTGGAGGTCCAGGAAGCAGAGAGATGGCTAAGGCTCATCAGGAC
CGTATGATCTCCCAAGTGTCCAGCTACTGAGTACCACAAGGTGATGGGTGGGAGGGTCCT
CCCACGGAAGGATACCGCAGTCCCTAGGGGTTGCAAGCCCCACATGTTCCACTGGCTGCT
AGAGCTACCTACTCAATCAGCCCTGGGCATCACCATCAGG

>279 Get primers
AAATAATTCTCATGAAAGTCAGGGTCTAGAACTCTGTCGGCTGAGGGCAGGTGCAGAGAC
TCAGGAGCTGGTTGGGATCATCAGGGATCTAGGCGGGTCAGGAGGAAGGGCAGCCAGTCT
GTACTCACCTCTGGCCTGGAGGTTGCACAGTCCCAGTGACAGCAGCAGGACCAGGATCCA
GGAGGGGACACCATGGTCCACAGGGTAACAAGGATGGAAGTTCATGCTTGATTCTGAGCC
GGGCGCTGACTGTCATGTGATTTGGTCACATGACCGACACAACGGGCGGGGCAGCATCAC
GTGATAGTCTGGCGGGGGCTGTCCTACTGTGGCTGGATTCTAGTTGGAGGATCAGCCTAC
TCTTCTTCAGTTTCCCGGTTCCTCCAAATTTCTGGGCTCCTACTTGTTTCCACAGAGATG
GATACTGTGGAGGTCCAGGAAGCAGAGAGATGGCTAAGGCTCATCAGGACCGTATGATCT
CCCAAGTGTCCAGCTACTGAGTACCACAAGGTGATGGGTGGGAGGGTCCTCCCACGGAAG
GATACCGCAGTCCCTAGGGGTTGCAAGCCCCACATGTTCCACTGGCTGCTAGAGCTACCT
ACTCAATCAGCCCTGGGCATCACCATCAGGTACTCGGCCAAAATGACCTCTCTGCTTCCA
GTCCTCAGTTCTGGTCAGCACCAGACAGGCCCATAATTAC

>280 Get primers
GTTGGGATCATCAGGGATCTAGGCGGGTCAGGAGGAAGGGCAGCCAGTCTGTACTCACCT
CTGGCCTGGAGGTTGCACAGTCCCAGTGACAGCAGCAGGACCAGGATCCAGGAGGGGACA
CCATGGTCCACAGGGTAACAAGGATGGAAGTTCATGCTTGATTCTGAGCCGGGCGCTGAC
TGTCATGTGATTTGGTCACATGACCGACACAACGGGCGGGGCAGCATCACGTGATAGTCT
GGCGGGGGCTGTCCTACTGTGGCTGGATTCTAGTTGGAGGATCAGCCTACTCTTCTTCAG
TTTCCCGGTTCCTCCAAATTTCTGGGCTCCTACTTGTTTCCACAGAGATGGATACTGTGG
AGGTCCAGGAAGCAGAGAGATGGCTAAGGCTCATCAGGACCGTATGATCTCCCAAGTGTC
CAGCTACTGAGTACCACAAGGTGATGGGTGGGAGGGTCCTCCCACGGAAGGATACCGCAG
TCCCTAGGGGTTGCAAGCCCCACATGTTCCACTGGCTGCTAGAGCTACCTACTCAATCAG
CCCTGGGCATCACCATCAGGTACTCGGCCAAAATGACCTCTCTGCTTCCAGTCCTCAGTT
CTGGTCAGCACCAGACAGGCCCATAATTACAGAGCCAGGGAAACTGGAACATTTGTCTCC
CCTTAGACAGTGGCAGCAGGAAGGTGGGGGGTTGTTGCAG

>281 Get primers
GGTTGCACAGTCCCAGTGACAGCAGCAGGACCAGGATCCAGGAGGGGACACCATGGTCCA
CAGGGTAACAAGGATGGAAGTTCATGCTTGATTCTGAGCCGGGCGCTGACTGTCATGTGA
TTTGGTCACATGACCGACACAACGGGCGGGGCAGCATCACGTGATAGTCTGGCGGGGGCT
GTCCTACTGTGGCTGGATTCTAGTTGGAGGATCAGCCTACTCTTCTTCAGTTTCCCGGTT
CCTCCAAATTTCTGGGCTCCTACTTGTTTCCACAGAGATGGATACTGTGGAGGTCCAGGA
AGCAGAGAGATGGCTAAGGCTCATCAGGACCGTATGATCTCCCAAGTGTCCAGCTACTGA
GTACCACAAGGTGATGGGTGGGAGGGTCCTCCCACGGAAGGATACCGCAGTCCCTAGGGG
TTGCAAGCCCCACATGTTCCACTGGCTGCTAGAGCTACCTACTCAATCAGCCCTGGGCAT
CACCATCAGGTACTCGGCCAAAATGACCTCTCTGCTTCCAGTCCTCAGTTCTGGTCAGCA
CCAGACAGGCCCATAATTACAGAGCCAGGGAAACTGGAACATTTGTCTCCCCTTAGACAG
TGGCAGCAGGAAGGTGGGGGGTTGTTGCAGAGGAACAGTGTCTCTGAGAGAGGACCTTGG
ACTTTCTGGGAATCTCTGAGCTGCCCGGTTCTCCCCACTG

>282 Get primers
AGGATGGAAGTTCATGCTTGATTCTGAGCCGGGCGCTGACTGTCATGTGATTTGGTCACA
TGACCGACACAACGGGCGGGGCAGCATCACGTGATAGTCTGGCGGGGGCTGTCCTACTGT
GGCTGGATTCTAGTTGGAGGATCAGCCTACTCTTCTTCAGTTTCCCGGTTCCTCCAAATT
TCTGGGCTCCTACTTGTTTCCACAGAGATGGATACTGTGGAGGTCCAGGAAGCAGAGAGA
TGGCTAAGGCTCATCAGGACCGTATGATCTCCCAAGTGTCCAGCTACTGAGTACCACAAG
GTGATGGGTGGGAGGGTCCTCCCACGGAAGGATACCGCAGTCCCTAGGGGTTGCAAGCCC
CACATGTTCCACTGGCTGCTAGAGCTACCTACTCAATCAGCCCTGGGCATCACCATCAGG
TACTCGGCCAAAATGACCTCTCTGCTTCCAGTCCTCAGTTCTGGTCAGCACCAGACAGGC
CCATAATTACAGAGCCAGGGAAACTGGAACATTTGTCTCCCCTTAGACAGTGGCAGCAGG
AAGGTGGGGGGTTGTTGCAGAGGAACAGTGTCTCTGAGAGAGGACCTTGGACTTTCTGGG
AATCTCTGAGCTGCCCGGTTCTCCCCACTGCTGGCACTGTGCCCACAGCCCAAACAGAAT
GGGGGAGATGGAGGGGCAGGGCTTCTGTGGGAAGCTGCCC

>283 Get primers
AACGGGCGGGGCAGCATCACGTGATAGTCTGGCGGGGGCTGTCCTACTGTGGCTGGATTC
TAGTTGGAGGATCAGCCTACTCTTCTTCAGTTTCCCGGTTCCTCCAAATTTCTGGGCTCC
TACTTGTTTCCACAGAGATGGATACTGTGGAGGTCCAGGAAGCAGAGAGATGGCTAAGGC
TCATCAGGACCGTATGATCTCCCAAGTGTCCAGCTACTGAGTACCACAAGGTGATGGGTG
GGAGGGTCCTCCCACGGAAGGATACCGCAGTCCCTAGGGGTTGCAAGCCCCACATGTTCC
ACTGGCTGCTAGAGCTACCTACTCAATCAGCCCTGGGCATCACCATCAGGTACTCGGCCA
AAATGACCTCTCTGCTTCCAGTCCTCAGTTCTGGTCAGCACCAGACAGGCCCATAATTAC
AGAGCCAGGGAAACTGGAACATTTGTCTCCCCTTAGACAGTGGCAGCAGGAAGGTGGGGG
GTTGTTGCAGAGGAACAGTGTCTCTGAGAGAGGACCTTGGACTTTCTGGGAATCTCTGAG
CTGCCCGGTTCTCCCCACTGCTGGCACTGTGCCCACAGCCCAAACAGAATGGGGGAGATG
GAGGGGCAGGGCTTCTGTGGGAAGCTGCCCTCCACCTCATTGGCACAGAGTGTCTCATTG
CAGAGAGAAAAAAGGACCAGTTTTCTCTCTGGCACCCAGG

>284 Get primers
ATCAGCCTACTCTTCTTCAGTTTCCCGGTTCCTCCAAATTTCTGGGCTCCTACTTGTTTC
CACAGAGATGGATACTGTGGAGGTCCAGGAAGCAGAGAGATGGCTAAGGCTCATCAGGAC
CGTATGATCTCCCAAGTGTCCAGCTACTGAGTACCACAAGGTGATGGGTGGGAGGGTCCT
CCCACGGAAGGATACCGCAGTCCCTAGGGGTTGCAAGCCCCACATGTTCCACTGGCTGCT
AGAGCTACCTACTCAATCAGCCCTGGGCATCACCATCAGGTACTCGGCCAAAATGACCTC
TCTGCTTCCAGTCCTCAGTTCTGGTCAGCACCAGACAGGCCCATAATTACAGAGCCAGGG
AAACTGGAACATTTGTCTCCCCTTAGACAGTGGCAGCAGGAAGGTGGGGGGTTGTTGCAG
AGGAACAGTGTCTCTGAGAGAGGACCTTGGACTTTCTGGGAATCTCTGAGCTGCCCGGTT
CTCCCCACTGCTGGCACTGTGCCCACAGCCCAAACAGAATGGGGGAGATGGAGGGGCAGG
GCTTCTGTGGGAAGCTGCCCTCCACCTCATTGGCACAGAGTGTCTCATTGCAGAGAGAAA
AAAGGACCAGTTTTCTCTCTGGCACCCAGGTCTGGAAGAGGAGTGACATCCACGGAAGTT
GGTGACTTGGACTGGCTGGCCGTGAGTGGAACATGTCCAT

>285 Get primers
AGGCCAAGAAGTAGACCCACCTGCCAGTGCCCGTAGACCCATGATCCTCTGGCCCTCATT
CAACAGCCGTTGACAGCCCACCTAGAGAAAGGCAGCAGAATATCTCAGTGGAGGCCCCTT
TCACAGAGCGTGGGTCAGGGCTGCTAGCTTCCAGGACACAACAGCAGATAGTGTCTGATG
GCATGAAAGCAGATAGCTACAAGGCTCTTGGACCAGGCTAGCACTGGGTCCTGCACCCAG
GGAGAGCCACCTCACCTTGACAGGGTTGGCAGGAAGGGGCCCTAGAAAGTCAGTAGGATA
CGGGTAGTCCATCATGGCGAGCACAGTAAATGCATTTCGGGCAAACCCAAAGAGCTGAGT
CAGGTCCTTTGGGCTGGAAAGTGATTGACAGGTACCAAAGTTCTGGCTGATGGTGTCATA
GGCTGGGAAGAGAGAGGCCAGGAGAAAAGGCTGAGGAAACTGCTGGCAAATGTGAAGGGC
AAGAATGAATGCCCAAGGTGGGCAGCAGGTGAGGAAAGAGTCCCTCACCTCCCTGGAGGA
ACAAGTCTTTGATTTGCTGAAAGGCATCCCGCACAGCCTGGGCGCACTTGGGACTCTGGC
CATAAAAGTCCTGGAGAAGAGACCAAGGTTGCTGCTGCCATTCTTGCACT

>286 Get primers
GCCGTTGACAGCCCACCTAGAGAAAGGCAGCAGAATATCTCAGTGGAGGCCCCTTTCACA
GAGCGTGGGTCAGGGCTGCTAGCTTCCAGGACACAACAGCAGATAGTGTCTGATGGCATG
AAAGCAGATAGCTACAAGGCTCTTGGACCAGGCTAGCACTGGGTCCTGCACCCAGGGAGA
GCCACCTCACCTTGACAGGGTTGGCAGGAAGGGGCCCTAGAAAGTCAGTAGGATACGGGT
AGTCCATCATGGCGAGCACAGTAAATGCATTTCGGGCAAACCCAAAGAGCTGAGTCAGGT
CCTTTGGGCTGGAAAGTGATTGACAGGTACCAAAGTTCTGGCTGATGGTGTCATAGGCTG
GGAAGAGAGAGGCCAGGAGAAAAGGCTGAGGAAACTGCTGGCAAATGTGAAGGGCAAGAA
TGAATGCCCAAGGTGGGCAGCAGGTGAGGAAAGAGTCCCTCACCTCCCTGGAGGAACAAG
TCTTTGATTTGCTGAAAGGCATCCCGCACAGCCTGGGCGCACTTGGGACTCTGGCCATAA
AAGTCCTGGAGAAGAGACCAAGGTTGCTGCTGCCATTCTTGCACTGGCCTGGGGTACCCA
AGTCCCCTCACTCACCGCTGTGACATCTCGGAAGAATTGGTAGGAGTCCC

>287 Get primers
TGGGTCAGGGCTGCTAGCTTCCAGGACACAACAGCAGATAGTGTCTGATGGCATGAAAGC
AGATAGCTACAAGGCTCTTGGACCAGGCTAGCACTGGGTCCTGCACCCAGGGAGAGCCAC
CTCACCTTGACAGGGTTGGCAGGAAGGGGCCCTAGAAAGTCAGTAGGATACGGGTAGTCC
ATCATGGCGAGCACAGTAAATGCATTTCGGGCAAACCCAAAGAGCTGAGTCAGGTCCTTT
GGGCTGGAAAGTGATTGACAGGTACCAAAGTTCTGGCTGATGGTGTCATAGGCTGGGAAG
AGAGAGGCCAGGAGAAAAGGCTGAGGAAACTGCTGGCAAATGTGAAGGGCAAGAATGAAT
GCCCAAGGTGGGCAGCAGGTGAGGAAAGAGTCCCTCACCTCCCTGGAGGAACAAGTCTTT
GATTTGCTGAAAGGCATCCCGCACAGCCTGGGCGCACTTGGGACTCTGGCCATAAAAGTC
CTGGAGAAGAGACCAAGGTTGCTGCTGCCATTCTTGCACTGGCCTGGGGTACCCAAGTCC
CCTCACTCACCGCTGTGACATCTCGGAAGAATTGGTAGGAGTCCCCAAGGCCTGCAACAG
CTACAACAGGAGCGCTGGCTGCCAGTGCCCCAGCCACCAGGTGGGGGTAC

>288 Get primers
GCTACAAGGCTCTTGGACCAGGCTAGCACTGGGTCCTGCACCCAGGGAGAGCCACCTCAC
CTTGACAGGGTTGGCAGGAAGGGGCCCTAGAAAGTCAGTAGGATACGGGTAGTCCATCAT
GGCGAGCACAGTAAATGCATTTCGGGCAAACCCAAAGAGCTGAGTCAGGTCCTTTGGGCT
GGAAAGTGATTGACAGGTACCAAAGTTCTGGCTGATGGTGTCATAGGCTGGGAAGAGAGA
GGCCAGGAGAAAAGGCTGAGGAAACTGCTGGCAAATGTGAAGGGCAAGAATGAATGCCCA
AGGTGGGCAGCAGGTGAGGAAAGAGTCCCTCACCTCCCTGGAGGAACAAGTCTTTGATTT
GCTGAAAGGCATCCCGCACAGCCTGGGCGCACTTGGGACTCTGGCCATAAAAGTCCTGGA
GAAGAGACCAAGGTTGCTGCTGCCATTCTTGCACTGGCCTGGGGTACCCAAGTCCCCTCA
CTCACCGCTGTGACATCTCGGAAGAATTGGTAGGAGTCCCCAAGGCCTGCAACAGCTACA
ACAGGAGCGCTGGCTGCCAGTGCCCCAGCCACCAGGTGGGGGTACTTCATCCTCATGTAG
GCACTCAGCATCCCCCCATAACTGGGAGTACAGAGCACAGATCATGGTTG

>289 Get primers
CAGGGTTGGCAGGAAGGGGCCCTAGAAAGTCAGTAGGATACGGGTAGTCCATCATGGCGA
GCACAGTAAATGCATTTCGGGCAAACCCAAAGAGCTGAGTCAGGTCCTTTGGGCTGGAAA
GTGATTGACAGGTACCAAAGTTCTGGCTGATGGTGTCATAGGCTGGGAAGAGAGAGGCCA
GGAGAAAAGGCTGAGGAAACTGCTGGCAAATGTGAAGGGCAAGAATGAATGCCCAAGGTG
GGCAGCAGGTGAGGAAAGAGTCCCTCACCTCCCTGGAGGAACAAGTCTTTGATTTGCTGA
AAGGCATCCCGCACAGCCTGGGCGCACTTGGGACTCTGGCCATAAAAGTCCTGGAGAAGA
GACCAAGGTTGCTGCTGCCATTCTTGCACTGGCCTGGGGTACCCAAGTCCCCTCACTCAC
CGCTGTGACATCTCGGAAGAATTGGTAGGAGTCCCCAAGGCCTGCAACAGCTACAACAGG
AGCGCTGGCTGCCAGTGCCCCAGCCACCAGGTGGGGGTACTTCATCCTCATGTAGGCACT
CAGCATCCCCCCATAACTGGGAGTACAGAGCACAGATCATGGTTGTGGGAAGCTGCCCAC
AACTCAGGCGAGCAGCCTCACTGTCCTCCAGGCTGAGGTGCTAGGCTGCT

>290 Get primers
GTAAATGCATTTCGGGCAAACCCAAAGAGCTGAGTCAGGTCCTTTGGGCTGGAAAGTGAT
TGACAGGTACCAAAGTTCTGGCTGATGGTGTCATAGGCTGGGAAGAGAGAGGCCAGGAGA
AAAGGCTGAGGAAACTGCTGGCAAATGTGAAGGGCAAGAATGAATGCCCAAGGTGGGCAG
CAGGTGAGGAAAGAGTCCCTCACCTCCCTGGAGGAACAAGTCTTTGATTTGCTGAAAGGC
ATCCCGCACAGCCTGGGCGCACTTGGGACTCTGGCCATAAAAGTCCTGGAGAAGAGACCA
AGGTTGCTGCTGCCATTCTTGCACTGGCCTGGGGTACCCAAGTCCCCTCACTCACCGCTG
TGACATCTCGGAAGAATTGGTAGGAGTCCCCAAGGCCTGCAACAGCTACAACAGGAGCGC
TGGCTGCCAGTGCCCCAGCCACCAGGTGGGGGTACTTCATCCTCATGTAGGCACTCAGCA
TCCCCCCATAACTGGGAGTACAGAGCACAGATCATGGTTGTGGGAAGCTGCCCACAACTC
AGGCGAGCAGCCTCACTGTCCTCCAGGCTGAGGTGCTAGGCTGCTCTTTCCCTGCTCAGA
ACGCCCAAGGGTGGGAAAGAAGGACCTGAAACTGTCAGGCCCACACACCC

>291 Get primers
GGTACCAAAGTTCTGGCTGATGGTGTCATAGGCTGGGAAGAGAGAGGCCAGGAGAAAAGG
CTGAGGAAACTGCTGGCAAATGTGAAGGGCAAGAATGAATGCCCAAGGTGGGCAGCAGGT
GAGGAAAGAGTCCCTCACCTCCCTGGAGGAACAAGTCTTTGATTTGCTGAAAGGCATCCC
GCACAGCCTGGGCGCACTTGGGACTCTGGCCATAAAAGTCCTGGAGAAGAGACCAAGGTT
GCTGCTGCCATTCTTGCACTGGCCTGGGGTACCCAAGTCCCCTCACTCACCGCTGTGACA
TCTCGGAAGAATTGGTAGGAGTCCCCAAGGCCTGCAACAGCTACAACAGGAGCGCTGGCT
GCCAGTGCCCCAGCCACCAGGTGGGGGTACTTCATCCTCATGTAGGCACTCAGCATCCCC
CCATAACTGGGAGTACAGAGCACAGATCATGGTTGTGGGAAGCTGCCCACAACTCAGGCG
AGCAGCCTCACTGTCCTCCAGGCTGAGGTGCTAGGCTGCTCTTTCCCTGCTCAGAACGCC
CAAGGGTGGGAAAGAAGGACCTGAAACTGTCAGGCCCACACACCCTGATCCCAGGGCCAA
GGCAGATACAGCCTTCACTGGGAGAAGGCACCTGTGGGTGCCCTGCCCTG

>292 Get primers
GAAACTGCTGGCAAATGTGAAGGGCAAGAATGAATGCCCAAGGTGGGCAGCAGGTGAGGA
AAGAGTCCCTCACCTCCCTGGAGGAACAAGTCTTTGATTTGCTGAAAGGCATCCCGCACA
GCCTGGGCGCACTTGGGACTCTGGCCATAAAAGTCCTGGAGAAGAGACCAAGGTTGCTGC
TGCCATTCTTGCACTGGCCTGGGGTACCCAAGTCCCCTCACTCACCGCTGTGACATCTCG
GAAGAATTGGTAGGAGTCCCCAAGGCCTGCAACAGCTACAACAGGAGCGCTGGCTGCCAG
TGCCCCAGCCACCAGGTGGGGGTACTTCATCCTCATGTAGGCACTCAGCATCCCCCCATA
ACTGGGAGTACAGAGCACAGATCATGGTTGTGGGAAGCTGCCCACAACTCAGGCGAGCAG
CCTCACTGTCCTCCAGGCTGAGGTGCTAGGCTGCTCTTTCCCTGCTCAGAACGCCCAAGG
GTGGGAAAGAAGGACCTGAAACTGTCAGGCCCACACACCCTGATCCCAGGGCCAAGGCAG
ATACAGCCTTCACTGGGAGAAGGCACCTGTGGGTGCCCTGCCCTGACCCAGCAATGAAGA
CATTGCAGAGACAAAGTCAGAAGGAATTGTCCCACTAGTGGGAACAACAT

>293 Get primers
TCCCTCACCTCCCTGGAGGAACAAGTCTTTGATTTGCTGAAAGGCATCCCGCACAGCCTG
GGCGCACTTGGGACTCTGGCCATAAAAGTCCTGGAGAAGAGACCAAGGTTGCTGCTGCCA
TTCTTGCACTGGCCTGGGGTACCCAAGTCCCCTCACTCACCGCTGTGACATCTCGGAAGA
ATTGGTAGGAGTCCCCAAGGCCTGCAACAGCTACAACAGGAGCGCTGGCTGCCAGTGCCC
CAGCCACCAGGTGGGGGTACTTCATCCTCATGTAGGCACTCAGCATCCCCCCATAACTGG
GAGTACAGAGCACAGATCATGGTTGTGGGAAGCTGCCCACAACTCAGGCGAGCAGCCTCA
CTGTCCTCCAGGCTGAGGTGCTAGGCTGCTCTTTCCCTGCTCAGAACGCCCAAGGGTGGG
AAAGAAGGACCTGAAACTGTCAGGCCCACACACCCTGATCCCAGGGCCAAGGCAGATACA
GCCTTCACTGGGAGAAGGCACCTGTGGGTGCCCTGCCCTGACCCAGCAATGAAGACATTG
CAGAGACAAAGTCAGAAGGAATTGTCCCACTAGTGGGAACAACATAGCATACACTGCCTA
TGAGGTCCACTCAAGGAGGGCTTCCAGAAGGAGGTAAAGCTAGACCCCGC

>294 Get primers
ACTTGGGACTCTGGCCATAAAAGTCCTGGAGAAGAGACCAAGGTTGCTGCTGCCATTCTT
GCACTGGCCTGGGGTACCCAAGTCCCCTCACTCACCGCTGTGACATCTCGGAAGAATTGG
TAGGAGTCCCCAAGGCCTGCAACAGCTACAACAGGAGCGCTGGCTGCCAGTGCCCCAGCC
ACCAGGTGGGGGTACTTCATCCTCATGTAGGCACTCAGCATCCCCCCATAACTGGGAGTA
CAGAGCACAGATCATGGTTGTGGGAAGCTGCCCACAACTCAGGCGAGCAGCCTCACTGTC
CTCCAGGCTGAGGTGCTAGGCTGCTCTTTCCCTGCTCAGAACGCCCAAGGGTGGGAAAGA
AGGACCTGAAACTGTCAGGCCCACACACCCTGATCCCAGGGCCAAGGCAGATACAGCCTT
CACTGGGAGAAGGCACCTGTGGGTGCCCTGCCCTGACCCAGCAATGAAGACATTGCAGAG
ACAAAGTCAGAAGGAATTGTCCCACTAGTGGGAACAACATAGCATACACTGCCTATGAGG
TCCACTCAAGGAGGGCTTCCAGAAGGAGGTAAAGCTAGACCCCGCCCTTCCACATGTGGG
GTAGGCATAGGATGTTGAGACTGTAAGAGACATCTCTTTGGCCCTCCTTG

>295 Get primers
GGCCTGGGGTACCCAAGTCCCCTCACTCACCGCTGTGACATCTCGGAAGAATTGGTAGGA
GTCCCCAAGGCCTGCAACAGCTACAACAGGAGCGCTGGCTGCCAGTGCCCCAGCCACCAG
GTGGGGGTACTTCATCCTCATGTAGGCACTCAGCATCCCCCCATAACTGGGAGTACAGAG
CACAGATCATGGTTGTGGGAAGCTGCCCACAACTCAGGCGAGCAGCCTCACTGTCCTCCA
GGCTGAGGTGCTAGGCTGCTCTTTCCCTGCTCAGAACGCCCAAGGGTGGGAAAGAAGGAC
CTGAAACTGTCAGGCCCACACACCCTGATCCCAGGGCCAAGGCAGATACAGCCTTCACTG
GGAGAAGGCACCTGTGGGTGCCCTGCCCTGACCCAGCAATGAAGACATTGCAGAGACAAA
GTCAGAAGGAATTGTCCCACTAGTGGGAACAACATAGCATACACTGCCTATGAGGTCCAC
TCAAGGAGGGCTTCCAGAAGGAGGTAAAGCTAGACCCCGCCCTTCCACATGTGGGGTAGG
CATAGGATGTTGAGACTGTAAGAGACATCTCTTTGGCCCTCCTTGTATAGGGTGTCAATC
GGCACAACAGGGTGGAGCCTTAGAGTAGGGTAAGATTAGGACTCTAGGTT

>296 Get primers
CAAGGCCTGCAACAGCTACAACAGGAGCGCTGGCTGCCAGTGCCCCAGCCACCAGGTGGG
GGTACTTCATCCTCATGTAGGCACTCAGCATCCCCCCATAACTGGGAGTACAGAGCACAG
ATCATGGTTGTGGGAAGCTGCCCACAACTCAGGCGAGCAGCCTCACTGTCCTCCAGGCTG
AGGTGCTAGGCTGCTCTTTCCCTGCTCAGAACGCCCAAGGGTGGGAAAGAAGGACCTGAA
ACTGTCAGGCCCACACACCCTGATCCCAGGGCCAAGGCAGATACAGCCTTCACTGGGAGA
AGGCACCTGTGGGTGCCCTGCCCTGACCCAGCAATGAAGACATTGCAGAGACAAAGTCAG
AAGGAATTGTCCCACTAGTGGGAACAACATAGCATACACTGCCTATGAGGTCCACTCAAG
GAGGGCTTCCAGAAGGAGGTAAAGCTAGACCCCGCCCTTCCACATGTGGGGTAGGCATAG
GATGTTGAGACTGTAAGAGACATCTCTTTGGCCCTCCTTGTATAGGGTGTCAATCGGCAC
AACAGGGTGGAGCCTTAGAGTAGGGTAAGATTAGGACTCTAGGTTCTCTCATGGGTCCAG
ATCTGTCATGAAGGGAGGTCAAGGACCCACCTCCCTCCAAAGGCTATGGT

>297 Get primers
TTCATCCTCATGTAGGCACTCAGCATCCCCCCATAACTGGGAGTACAGAGCACAGATCAT
GGTTGTGGGAAGCTGCCCACAACTCAGGCGAGCAGCCTCACTGTCCTCCAGGCTGAGGTG
CTAGGCTGCTCTTTCCCTGCTCAGAACGCCCAAGGGTGGGAAAGAAGGACCTGAAACTGT
CAGGCCCACACACCCTGATCCCAGGGCCAAGGCAGATACAGCCTTCACTGGGAGAAGGCA
CCTGTGGGTGCCCTGCCCTGACCCAGCAATGAAGACATTGCAGAGACAAAGTCAGAAGGA
ATTGTCCCACTAGTGGGAACAACATAGCATACACTGCCTATGAGGTCCACTCAAGGAGGG
CTTCCAGAAGGAGGTAAAGCTAGACCCCGCCCTTCCACATGTGGGGTAGGCATAGGATGT
TGAGACTGTAAGAGACATCTCTTTGGCCCTCCTTGTATAGGGTGTCAATCGGCACAACAG
GGTGGAGCCTTAGAGTAGGGTAAGATTAGGACTCTAGGTTCTCTCATGGGTCCAGATCTG
TCATGAAGGGAGGTCAAGGACCCACCTCCCTCCAAAGGCTATGGTGGGGGCATCATGGAC
CCCAAGGTCCTGCCGCAGGGCCTGGAGCAGCACAGCAAAGTCGGCCAGCG

>307 Get primers
CCTGCTCCACAGTCAGCAGCTGTGTATATCCCCGCTGTGTGGACTGGACACCGAACGGAA
GCGATTTCCCATAGTACCGCTGCAGAAAGCAGGAAGGGATGGCTAATCCACTCCTCGGTG
CTCCCCACCTCCTTCAACTCAGGGACTGCCAGGAACTGTACAGGTACCCACGTGCTCAGC
AAAGACAAGCAGGGCCTCCTGCTGGGCTGCCAGTTCCACCATGAAGCCAGAGTTGTTAGC
GAAGGACCAGATATCCCCCTCATTCCCTGTGTAGAAAAAGATGGGCCCTTCGCCCATCTT
CCAGAACTTATCTGTTGGAAGTAAATGAGTTTCCATAAGGCCAGGGAAACGCAGGTAGGA
ACCCATGCGGTCGAGCCAGCACTCACCTGACACTAGGAACCGCTGGCCAAAGGTTTTGTT
GCCGAAACTCTCAAAGTTGAAATGGTCCATGTATTGCTCAAAATAATTCTCATGAAAGTC
AGGGTCTAGAACTCTGTCGGCTGAGGGCAGGTGCAGAGACTCAGGAGCTGGTTGGGATCA
TCAGGGATCTAGGCGGGTCAGGAGGAAGGGCAGCCAGTCTGTACTCACCTCTGGCCTGGA
GGTTGCACAGTCCCAGTGACAGCAGCAGGACCAGGATCCAGGAGGGGACA

>308 Get primers
TTCCCATAGTACCGCTGCAGAAAGCAGGAAGGGATGGCTAATCCACTCCTCGGTGCTCCC
CACCTCCTTCAACTCAGGGACTGCCAGGAACTGTACAGGTACCCACGTGCTCAGCAAAGA
CAAGCAGGGCCTCCTGCTGGGCTGCCAGTTCCACCATGAAGCCAGAGTTGTTAGCGAAGG
ACCAGATATCCCCCTCATTCCCTGTGTAGAAAAAGATGGGCCCTTCGCCCATCTTCCAGA
ACTTATCTGTTGGAAGTAAATGAGTTTCCATAAGGCCAGGGAAACGCAGGTAGGAACCCA
TGCGGTCGAGCCAGCACTCACCTGACACTAGGAACCGCTGGCCAAAGGTTTTGTTGCCGA
AACTCTCAAAGTTGAAATGGTCCATGTATTGCTCAAAATAATTCTCATGAAAGTCAGGGT
CTAGAACTCTGTCGGCTGAGGGCAGGTGCAGAGACTCAGGAGCTGGTTGGGATCATCAGG
GATCTAGGCGGGTCAGGAGGAAGGGCAGCCAGTCTGTACTCACCTCTGGCCTGGAGGTTG
CACAGTCCCAGTGACAGCAGCAGGACCAGGATCCAGGAGGGGACACCATGGTCCACAGGG
TAACAAGGATGGAAGTTCATGCTTGATTCTGAGCCGGGCGCTGACTGTCA

>309 Get primers
CCTTCAACTCAGGGACTGCCAGGAACTGTACAGGTACCCACGTGCTCAGCAAAGACAAGC
AGGGCCTCCTGCTGGGCTGCCAGTTCCACCATGAAGCCAGAGTTGTTAGCGAAGGACCAG
ATATCCCCCTCATTCCCTGTGTAGAAAAAGATGGGCCCTTCGCCCATCTTCCAGAACTTA
TCTGTTGGAAGTAAATGAGTTTCCATAAGGCCAGGGAAACGCAGGTAGGAACCCATGCGG
TCGAGCCAGCACTCACCTGACACTAGGAACCGCTGGCCAAAGGTTTTGTTGCCGAAACTC
TCAAAGTTGAAATGGTCCATGTATTGCTCAAAATAATTCTCATGAAAGTCAGGGTCTAGA
ACTCTGTCGGCTGAGGGCAGGTGCAGAGACTCAGGAGCTGGTTGGGATCATCAGGGATCT
AGGCGGGTCAGGAGGAAGGGCAGCCAGTCTGTACTCACCTCTGGCCTGGAGGTTGCACAG
TCCCAGTGACAGCAGCAGGACCAGGATCCAGGAGGGGACACCATGGTCCACAGGGTAACA
AGGATGGAAGTTCATGCTTGATTCTGAGCCGGGCGCTGACTGTCATGTGATTTGGTCACA
TGACCGACACAACGGGCGGGGCAGCATCACGTGATAGTCTGGCGGGGGCT

>310 Get primers
CTCCTGCTGGGCTGCCAGTTCCACCATGAAGCCAGAGTTGTTAGCGAAGGACCAGATATC
CCCCTCATTCCCTGTGTAGAAAAAGATGGGCCCTTCGCCCATCTTCCAGAACTTATCTGT
TGGAAGTAAATGAGTTTCCATAAGGCCAGGGAAACGCAGGTAGGAACCCATGCGGTCGAG
CCAGCACTCACCTGACACTAGGAACCGCTGGCCAAAGGTTTTGTTGCCGAAACTCTCAAA
GTTGAAATGGTCCATGTATTGCTCAAAATAATTCTCATGAAAGTCAGGGTCTAGAACTCT
GTCGGCTGAGGGCAGGTGCAGAGACTCAGGAGCTGGTTGGGATCATCAGGGATCTAGGCG
GGTCAGGAGGAAGGGCAGCCAGTCTGTACTCACCTCTGGCCTGGAGGTTGCACAGTCCCA
GTGACAGCAGCAGGACCAGGATCCAGGAGGGGACACCATGGTCCACAGGGTAACAAGGAT
GGAAGTTCATGCTTGATTCTGAGCCGGGCGCTGACTGTCATGTGATTTGGTCACATGACC
GACACAACGGGCGGGGCAGCATCACGTGATAGTCTGGCGGGGGCTGTCCTACTGTGGCTG
GATTCTAGTTGGAGGATCAGCCTACTCTTCTTCAGTTTCCCGGTTCCTCC

>311 Get primers
CATTCCCTGTGTAGAAAAAGATGGGCCCTTCGCCCATCTTCCAGAACTTATCTGTTGGAA
GTAAATGAGTTTCCATAAGGCCAGGGAAACGCAGGTAGGAACCCATGCGGTCGAGCCAGC
ACTCACCTGACACTAGGAACCGCTGGCCAAAGGTTTTGTTGCCGAAACTCTCAAAGTTGA
AATGGTCCATGTATTGCTCAAAATAATTCTCATGAAAGTCAGGGTCTAGAACTCTGTCGG
CTGAGGGCAGGTGCAGAGACTCAGGAGCTGGTTGGGATCATCAGGGATCTAGGCGGGTCA
GGAGGAAGGGCAGCCAGTCTGTACTCACCTCTGGCCTGGAGGTTGCACAGTCCCAGTGAC
AGCAGCAGGACCAGGATCCAGGAGGGGACACCATGGTCCACAGGGTAACAAGGATGGAAG
TTCATGCTTGATTCTGAGCCGGGCGCTGACTGTCATGTGATTTGGTCACATGACCGACAC
AACGGGCGGGGCAGCATCACGTGATAGTCTGGCGGGGGCTGTCCTACTGTGGCTGGATTC
TAGTTGGAGGATCAGCCTACTCTTCTTCAGTTTCCCGGTTCCTCCAAATTTCTGGGCTCC
TACTTGTTTCCACAGAGATGGATACTGTGGAGGTCCAGGAAGCAGAGAGA

>312 Get primers
TGAGTTTCCATAAGGCCAGGGAAACGCAGGTAGGAACCCATGCGGTCGAGCCAGCACTCA
CCTGACACTAGGAACCGCTGGCCAAAGGTTTTGTTGCCGAAACTCTCAAAGTTGAAATGG
TCCATGTATTGCTCAAAATAATTCTCATGAAAGTCAGGGTCTAGAACTCTGTCGGCTGAG
GGCAGGTGCAGAGACTCAGGAGCTGGTTGGGATCATCAGGGATCTAGGCGGGTCAGGAGG
AAGGGCAGCCAGTCTGTACTCACCTCTGGCCTGGAGGTTGCACAGTCCCAGTGACAGCAG
CAGGACCAGGATCCAGGAGGGGACACCATGGTCCACAGGGTAACAAGGATGGAAGTTCAT
GCTTGATTCTGAGCCGGGCGCTGACTGTCATGTGATTTGGTCACATGACCGACACAACGG
GCGGGGCAGCATCACGTGATAGTCTGGCGGGGGCTGTCCTACTGTGGCTGGATTCTAGTT
GGAGGATCAGCCTACTCTTCTTCAGTTTCCCGGTTCCTCCAAATTTCTGGGCTCCTACTT
GTTTCCACAGAGATGGATACTGTGGAGGTCCAGGAAGCAGAGAGATGGCTAAGGCTCATC
AGGACCGTATGATCTCCCAAGTGTCCAGCTACTGAGTACCACAAGGTGAT

>313 Get primers
CACTAGGAACCGCTGGCCAAAGGTTTTGTTGCCGAAACTCTCAAAGTTGAAATGGTCCAT
GTATTGCTCAAAATAATTCTCATGAAAGTCAGGGTCTAGAACTCTGTCGGCTGAGGGCAG
GTGCAGAGACTCAGGAGCTGGTTGGGATCATCAGGGATCTAGGCGGGTCAGGAGGAAGGG
CAGCCAGTCTGTACTCACCTCTGGCCTGGAGGTTGCACAGTCCCAGTGACAGCAGCAGGA
CCAGGATCCAGGAGGGGACACCATGGTCCACAGGGTAACAAGGATGGAAGTTCATGCTTG
ATTCTGAGCCGGGCGCTGACTGTCATGTGATTTGGTCACATGACCGACACAACGGGCGGG
GCAGCATCACGTGATAGTCTGGCGGGGGCTGTCCTACTGTGGCTGGATTCTAGTTGGAGG
ATCAGCCTACTCTTCTTCAGTTTCCCGGTTCCTCCAAATTTCTGGGCTCCTACTTGTTTC
CACAGAGATGGATACTGTGGAGGTCCAGGAAGCAGAGAGATGGCTAAGGCTCATCAGGAC
CGTATGATCTCCCAAGTGTCCAGCTACTGAGTACCACAAGGTGATGGGTGGGAGGGTCCT
CCCACGGAAGGATACCGCAGTCCCTAGGGGTTGCAAGCCCCACATGTTCC

>314 Get primers
GCTCAAAATAATTCTCATGAAAGTCAGGGTCTAGAACTCTGTCGGCTGAGGGCAGGTGCA
GAGACTCAGGAGCTGGTTGGGATCATCAGGGATCTAGGCGGGTCAGGAGGAAGGGCAGCC
AGTCTGTACTCACCTCTGGCCTGGAGGTTGCACAGTCCCAGTGACAGCAGCAGGACCAGG
ATCCAGGAGGGGACACCATGGTCCACAGGGTAACAAGGATGGAAGTTCATGCTTGATTCT
GAGCCGGGCGCTGACTGTCATGTGATTTGGTCACATGACCGACACAACGGGCGGGGCAGC
ATCACGTGATAGTCTGGCGGGGGCTGTCCTACTGTGGCTGGATTCTAGTTGGAGGATCAG
CCTACTCTTCTTCAGTTTCCCGGTTCCTCCAAATTTCTGGGCTCCTACTTGTTTCCACAG
AGATGGATACTGTGGAGGTCCAGGAAGCAGAGAGATGGCTAAGGCTCATCAGGACCGTAT
GATCTCCCAAGTGTCCAGCTACTGAGTACCACAAGGTGATGGGTGGGAGGGTCCTCCCAC
GGAAGGATACCGCAGTCCCTAGGGGTTGCAAGCCCCACATGTTCCACTGGCTGCTAGAGC
TACCTACTCAATCAGCCCTGGGCATCACCATCAGGTACTCGGCCAAAATG

>315 Get primers
TCAGGAGCTGGTTGGGATCATCAGGGATCTAGGCGGGTCAGGAGGAAGGGCAGCCAGTCT
GTACTCACCTCTGGCCTGGAGGTTGCACAGTCCCAGTGACAGCAGCAGGACCAGGATCCA
GGAGGGGACACCATGGTCCACAGGGTAACAAGGATGGAAGTTCATGCTTGATTCTGAGCC
GGGCGCTGACTGTCATGTGATTTGGTCACATGACCGACACAACGGGCGGGGCAGCATCAC
GTGATAGTCTGGCGGGGGCTGTCCTACTGTGGCTGGATTCTAGTTGGAGGATCAGCCTAC
TCTTCTTCAGTTTCCCGGTTCCTCCAAATTTCTGGGCTCCTACTTGTTTCCACAGAGATG
GATACTGTGGAGGTCCAGGAAGCAGAGAGATGGCTAAGGCTCATCAGGACCGTATGATCT
CCCAAGTGTCCAGCTACTGAGTACCACAAGGTGATGGGTGGGAGGGTCCTCCCACGGAAG
GATACCGCAGTCCCTAGGGGTTGCAAGCCCCACATGTTCCACTGGCTGCTAGAGCTACCT
ACTCAATCAGCCCTGGGCATCACCATCAGGTACTCGGCCAAAATGACCTCTCTGCTTCCA
GTCCTCAGTTCTGGTCAGCACCAGACAGGCCCATAATTACAGAGCCAGGG

>316 Get primers
CACCTCTGGCCTGGAGGTTGCACAGTCCCAGTGACAGCAGCAGGACCAGGATCCAGGAGG
GGACACCATGGTCCACAGGGTAACAAGGATGGAAGTTCATGCTTGATTCTGAGCCGGGCG
CTGACTGTCATGTGATTTGGTCACATGACCGACACAACGGGCGGGGCAGCATCACGTGAT
AGTCTGGCGGGGGCTGTCCTACTGTGGCTGGATTCTAGTTGGAGGATCAGCCTACTCTTC
TTCAGTTTCCCGGTTCCTCCAAATTTCTGGGCTCCTACTTGTTTCCACAGAGATGGATAC
TGTGGAGGTCCAGGAAGCAGAGAGATGGCTAAGGCTCATCAGGACCGTATGATCTCCCAA
GTGTCCAGCTACTGAGTACCACAAGGTGATGGGTGGGAGGGTCCTCCCACGGAAGGATAC
CGCAGTCCCTAGGGGTTGCAAGCCCCACATGTTCCACTGGCTGCTAGAGCTACCTACTCA
ATCAGCCCTGGGCATCACCATCAGGTACTCGGCCAAAATGACCTCTCTGCTTCCAGTCCT
CAGTTCTGGTCAGCACCAGACAGGCCCATAATTACAGAGCCAGGGAAACTGGAACATTTG
TCTCCCCTTAGACAGTGGCAGCAGGAAGGTGGGGGGTTGTTGCAGAGGAA

>317 Get primers
CCATGGTCCACAGGGTAACAAGGATGGAAGTTCATGCTTGATTCTGAGCCGGGCGCTGAC
TGTCATGTGATTTGGTCACATGACCGACACAACGGGCGGGGCAGCATCACGTGATAGTCT
GGCGGGGGCTGTCCTACTGTGGCTGGATTCTAGTTGGAGGATCAGCCTACTCTTCTTCAG
TTTCCCGGTTCCTCCAAATTTCTGGGCTCCTACTTGTTTCCACAGAGATGGATACTGTGG
AGGTCCAGGAAGCAGAGAGATGGCTAAGGCTCATCAGGACCGTATGATCTCCCAAGTGTC
CAGCTACTGAGTACCACAAGGTGATGGGTGGGAGGGTCCTCCCACGGAAGGATACCGCAG
TCCCTAGGGGTTGCAAGCCCCACATGTTCCACTGGCTGCTAGAGCTACCTACTCAATCAG
CCCTGGGCATCACCATCAGGTACTCGGCCAAAATGACCTCTCTGCTTCCAGTCCTCAGTT
CTGGTCAGCACCAGACAGGCCCATAATTACAGAGCCAGGGAAACTGGAACATTTGTCTCC
CCTTAGACAGTGGCAGCAGGAAGGTGGGGGGTTGTTGCAGAGGAACAGTGTCTCTGAGAG
AGGACCTTGGACTTTCTGGGAATCTCTGAGCTGCCCGGTTCTCCCCACTG

>318 Get primers
TGTGATTTGGTCACATGACCGACACAACGGGCGGGGCAGCATCACGTGATAGTCTGGCGG
GGGCTGTCCTACTGTGGCTGGATTCTAGTTGGAGGATCAGCCTACTCTTCTTCAGTTTCC
CGGTTCCTCCAAATTTCTGGGCTCCTACTTGTTTCCACAGAGATGGATACTGTGGAGGTC
CAGGAAGCAGAGAGATGGCTAAGGCTCATCAGGACCGTATGATCTCCCAAGTGTCCAGCT
ACTGAGTACCACAAGGTGATGGGTGGGAGGGTCCTCCCACGGAAGGATACCGCAGTCCCT
AGGGGTTGCAAGCCCCACATGTTCCACTGGCTGCTAGAGCTACCTACTCAATCAGCCCTG
GGCATCACCATCAGGTACTCGGCCAAAATGACCTCTCTGCTTCCAGTCCTCAGTTCTGGT
CAGCACCAGACAGGCCCATAATTACAGAGCCAGGGAAACTGGAACATTTGTCTCCCCTTA
GACAGTGGCAGCAGGAAGGTGGGGGGTTGTTGCAGAGGAACAGTGTCTCTGAGAGAGGAC
CTTGGACTTTCTGGGAATCTCTGAGCTGCCCGGTTCTCCCCACTGCTGGCACTGTGCCCA
CAGCCCAAACAGAATGGGGGAGATGGAGGGGCAGGGCTTCTGTGGGAAGC

>319 Get primers
GTCCTACTGTGGCTGGATTCTAGTTGGAGGATCAGCCTACTCTTCTTCAGTTTCCCGGTT
CCTCCAAATTTCTGGGCTCCTACTTGTTTCCACAGAGATGGATACTGTGGAGGTCCAGGA
AGCAGAGAGATGGCTAAGGCTCATCAGGACCGTATGATCTCCCAAGTGTCCAGCTACTGA
GTACCACAAGGTGATGGGTGGGAGGGTCCTCCCACGGAAGGATACCGCAGTCCCTAGGGG
TTGCAAGCCCCACATGTTCCACTGGCTGCTAGAGCTACCTACTCAATCAGCCCTGGGCAT
CACCATCAGGTACTCGGCCAAAATGACCTCTCTGCTTCCAGTCCTCAGTTCTGGTCAGCA
CCAGACAGGCCCATAATTACAGAGCCAGGGAAACTGGAACATTTGTCTCCCCTTAGACAG
TGGCAGCAGGAAGGTGGGGGGTTGTTGCAGAGGAACAGTGTCTCTGAGAGAGGACCTTGG
ACTTTCTGGGAATCTCTGAGCTGCCCGGTTCTCCCCACTGCTGGCACTGTGCCCACAGCC
CAAACAGAATGGGGGAGATGGAGGGGCAGGGCTTCTGTGGGAAGCTGCCCTCCACCTCAT
TGGCACAGAGTGTCTCATTGCAGAGAGAAAAAAGGACCAGTTTTCTCTCT

>320 Get primers
AAATTTCTGGGCTCCTACTTGTTTCCACAGAGATGGATACTGTGGAGGTCCAGGAAGCAG
AGAGATGGCTAAGGCTCATCAGGACCGTATGATCTCCCAAGTGTCCAGCTACTGAGTACC
ACAAGGTGATGGGTGGGAGGGTCCTCCCACGGAAGGATACCGCAGTCCCTAGGGGTTGCA
AGCCCCACATGTTCCACTGGCTGCTAGAGCTACCTACTCAATCAGCCCTGGGCATCACCA
TCAGGTACTCGGCCAAAATGACCTCTCTGCTTCCAGTCCTCAGTTCTGGTCAGCACCAGA
CAGGCCCATAATTACAGAGCCAGGGAAACTGGAACATTTGTCTCCCCTTAGACAGTGGCA
GCAGGAAGGTGGGGGGTTGTTGCAGAGGAACAGTGTCTCTGAGAGAGGACCTTGGACTTT
CTGGGAATCTCTGAGCTGCCCGGTTCTCCCCACTGCTGGCACTGTGCCCACAGCCCAAAC
AGAATGGGGGAGATGGAGGGGCAGGGCTTCTGTGGGAAGCTGCCCTCCACCTCATTGGCA
CAGAGTGTCTCATTGCAGAGAGAAAAAAGGACCAGTTTTCTCTCTGGCACCCAGGTCTGG
AAGAGGAGTGACATCCACGGAAGTTGGTGACTTGGACTGGCTGGCCGTGA

>321 Get primers
TGGCTAAGGCTCATCAGGACCGTATGATCTCCCAAGTGTCCAGCTACTGAGTACCACAAG
GTGATGGGTGGGAGGGTCCTCCCACGGAAGGATACCGCAGTCCCTAGGGGTTGCAAGCCC
CACATGTTCCACTGGCTGCTAGAGCTACCTACTCAATCAGCCCTGGGCATCACCATCAGG
TACTCGGCCAAAATGACCTCTCTGCTTCCAGTCCTCAGTTCTGGTCAGCACCAGACAGGC
CCATAATTACAGAGCCAGGGAAACTGGAACATTTGTCTCCCCTTAGACAGTGGCAGCAGG
AAGGTGGGGGGTTGTTGCAGAGGAACAGTGTCTCTGAGAGAGGACCTTGGACTTTCTGGG
AATCTCTGAGCTGCCCGGTTCTCCCCACTGCTGGCACTGTGCCCACAGCCCAAACAGAAT
GGGGGAGATGGAGGGGCAGGGCTTCTGTGGGAAGCTGCCCTCCACCTCATTGGCACAGAG
TGTCTCATTGCAGAGAGAAAAAAGGACCAGTTTTCTCTCTGGCACCCAGGTCTGGAAGAG
GAGTGACATCCACGGAAGTTGGTGACTTGGACTGGCTGGCCGTGAGTGGAACATGTCCAT
CCAGCATGGCCACAGTCCAGTGGGACACACAGCCTAGAGCTGTGGAATGC

>322 Get primers
AGGCCAAGAAGTAGACCCACCTGCCAGTGCCCGTAGACCCATGATCCTCTGGCCCTCATT
CAACAGCCGTTGACAGCCCACCTAGAGAAAGGCAGCAGAATATCTCAGTGGAGGCCCCTT
TCACAGAGCGTGGGTCAGGGCTGCTAGCTTCCAGGACACAACAGCAGATAGTGTCTGATG
GCATGAAAGCAGATAGCTACAAGGCTCTTGGACCAGGCTAGCACTGGGTCCTGCACCCAG
GGAGAGCCACCTCACCTTGACAGGGTTGGCAGGAAGGGGCCCTAGAAAGTCAGTAGGATA
CGGGTAGTCCATCATGGCGAGCACAGTAAATGCATTTCGGGCAAACCCAAAGAGCTGAGT
CAGGTCCTTTGGGCTGGAAAGTGATTGACAGGTACCAAAGTTCTGGCTGATGGTGTCATA
GGCTGGGAAGAGAGAGGCCAGGAGAAAAGGCTGAGGAAACTGCTGGCAAATGTGAAGGGC
AAGAATGAATGCCCAAGGTGGGCAGCAGGTGAGGAAAGAGTCCCTCACCTCCCTGGAGGA
ACAAGTCTTTGATTTGCTGAAAGGCATCCCGCACAGCCTGGGCGCACTTGGGACTCTGGC

>323 Get primers
CAACAGCCGTTGACAGCCCACCTAGAGAAAGGCAGCAGAATATCTCAGTGGAGGCCCCTT
TCACAGAGCGTGGGTCAGGGCTGCTAGCTTCCAGGACACAACAGCAGATAGTGTCTGATG
GCATGAAAGCAGATAGCTACAAGGCTCTTGGACCAGGCTAGCACTGGGTCCTGCACCCAG
GGAGAGCCACCTCACCTTGACAGGGTTGGCAGGAAGGGGCCCTAGAAAGTCAGTAGGATA
CGGGTAGTCCATCATGGCGAGCACAGTAAATGCATTTCGGGCAAACCCAAAGAGCTGAGT
CAGGTCCTTTGGGCTGGAAAGTGATTGACAGGTACCAAAGTTCTGGCTGATGGTGTCATA
GGCTGGGAAGAGAGAGGCCAGGAGAAAAGGCTGAGGAAACTGCTGGCAAATGTGAAGGGC
AAGAATGAATGCCCAAGGTGGGCAGCAGGTGAGGAAAGAGTCCCTCACCTCCCTGGAGGA
ACAAGTCTTTGATTTGCTGAAAGGCATCCCGCACAGCCTGGGCGCACTTGGGACTCTGGC
CATAAAAGTCCTGGAGAAGAGACCAAGGTTGCTGCTGCCATTCTTGCACTGGCCTGGGGT

>324 Get primers
TCACAGAGCGTGGGTCAGGGCTGCTAGCTTCCAGGACACAACAGCAGATAGTGTCTGATG
GCATGAAAGCAGATAGCTACAAGGCTCTTGGACCAGGCTAGCACTGGGTCCTGCACCCAG
GGAGAGCCACCTCACCTTGACAGGGTTGGCAGGAAGGGGCCCTAGAAAGTCAGTAGGATA
CGGGTAGTCCATCATGGCGAGCACAGTAAATGCATTTCGGGCAAACCCAAAGAGCTGAGT
CAGGTCCTTTGGGCTGGAAAGTGATTGACAGGTACCAAAGTTCTGGCTGATGGTGTCATA
GGCTGGGAAGAGAGAGGCCAGGAGAAAAGGCTGAGGAAACTGCTGGCAAATGTGAAGGGC
AAGAATGAATGCCCAAGGTGGGCAGCAGGTGAGGAAAGAGTCCCTCACCTCCCTGGAGGA
ACAAGTCTTTGATTTGCTGAAAGGCATCCCGCACAGCCTGGGCGCACTTGGGACTCTGGC
CATAAAAGTCCTGGAGAAGAGACCAAGGTTGCTGCTGCCATTCTTGCACTGGCCTGGGGT
ACCCAAGTCCCCTCACTCACCGCTGTGACATCTCGGAAGAATTGGTAGGAGTCCCCAAGG

>325 Get primers
GCATGAAAGCAGATAGCTACAAGGCTCTTGGACCAGGCTAGCACTGGGTCCTGCACCCAG
GGAGAGCCACCTCACCTTGACAGGGTTGGCAGGAAGGGGCCCTAGAAAGTCAGTAGGATA
CGGGTAGTCCATCATGGCGAGCACAGTAAATGCATTTCGGGCAAACCCAAAGAGCTGAGT
CAGGTCCTTTGGGCTGGAAAGTGATTGACAGGTACCAAAGTTCTGGCTGATGGTGTCATA
GGCTGGGAAGAGAGAGGCCAGGAGAAAAGGCTGAGGAAACTGCTGGCAAATGTGAAGGGC
AAGAATGAATGCCCAAGGTGGGCAGCAGGTGAGGAAAGAGTCCCTCACCTCCCTGGAGGA
ACAAGTCTTTGATTTGCTGAAAGGCATCCCGCACAGCCTGGGCGCACTTGGGACTCTGGC
CATAAAAGTCCTGGAGAAGAGACCAAGGTTGCTGCTGCCATTCTTGCACTGGCCTGGGGT
ACCCAAGTCCCCTCACTCACCGCTGTGACATCTCGGAAGAATTGGTAGGAGTCCCCAAGG
CCTGCAACAGCTACAACAGGAGCGCTGGCTGCCAGTGCCCCAGCCACCAGGTGGGGGTAC

>326 Get primers
GGAGAGCCACCTCACCTTGACAGGGTTGGCAGGAAGGGGCCCTAGAAAGTCAGTAGGATA
CGGGTAGTCCATCATGGCGAGCACAGTAAATGCATTTCGGGCAAACCCAAAGAGCTGAGT
CAGGTCCTTTGGGCTGGAAAGTGATTGACAGGTACCAAAGTTCTGGCTGATGGTGTCATA
GGCTGGGAAGAGAGAGGCCAGGAGAAAAGGCTGAGGAAACTGCTGGCAAATGTGAAGGGC
AAGAATGAATGCCCAAGGTGGGCAGCAGGTGAGGAAAGAGTCCCTCACCTCCCTGGAGGA
ACAAGTCTTTGATTTGCTGAAAGGCATCCCGCACAGCCTGGGCGCACTTGGGACTCTGGC
CATAAAAGTCCTGGAGAAGAGACCAAGGTTGCTGCTGCCATTCTTGCACTGGCCTGGGGT
ACCCAAGTCCCCTCACTCACCGCTGTGACATCTCGGAAGAATTGGTAGGAGTCCCCAAGG
CCTGCAACAGCTACAACAGGAGCGCTGGCTGCCAGTGCCCCAGCCACCAGGTGGGGGTAC
TTCATCCTCATGTAGGCACTCAGCATCCCCCCATAACTGGGAGTACAGAGCACAGATCAT

>327 Get primers
CGGGTAGTCCATCATGGCGAGCACAGTAAATGCATTTCGGGCAAACCCAAAGAGCTGAGT
CAGGTCCTTTGGGCTGGAAAGTGATTGACAGGTACCAAAGTTCTGGCTGATGGTGTCATA
GGCTGGGAAGAGAGAGGCCAGGAGAAAAGGCTGAGGAAACTGCTGGCAAATGTGAAGGGC
AAGAATGAATGCCCAAGGTGGGCAGCAGGTGAGGAAAGAGTCCCTCACCTCCCTGGAGGA
ACAAGTCTTTGATTTGCTGAAAGGCATCCCGCACAGCCTGGGCGCACTTGGGACTCTGGC
CATAAAAGTCCTGGAGAAGAGACCAAGGTTGCTGCTGCCATTCTTGCACTGGCCTGGGGT
ACCCAAGTCCCCTCACTCACCGCTGTGACATCTCGGAAGAATTGGTAGGAGTCCCCAAGG
CCTGCAACAGCTACAACAGGAGCGCTGGCTGCCAGTGCCCCAGCCACCAGGTGGGGGTAC
TTCATCCTCATGTAGGCACTCAGCATCCCCCCATAACTGGGAGTACAGAGCACAGATCAT
GGTTGTGGGAAGCTGCCCACAACTCAGGCGAGCAGCCTCACTGTCCTCCAGGCTGAGGTG

>328 Get primers
CAGGTCCTTTGGGCTGGAAAGTGATTGACAGGTACCAAAGTTCTGGCTGATGGTGTCATA
GGCTGGGAAGAGAGAGGCCAGGAGAAAAGGCTGAGGAAACTGCTGGCAAATGTGAAGGGC
AAGAATGAATGCCCAAGGTGGGCAGCAGGTGAGGAAAGAGTCCCTCACCTCCCTGGAGGA
ACAAGTCTTTGATTTGCTGAAAGGCATCCCGCACAGCCTGGGCGCACTTGGGACTCTGGC
CATAAAAGTCCTGGAGAAGAGACCAAGGTTGCTGCTGCCATTCTTGCACTGGCCTGGGGT
ACCCAAGTCCCCTCACTCACCGCTGTGACATCTCGGAAGAATTGGTAGGAGTCCCCAAGG
CCTGCAACAGCTACAACAGGAGCGCTGGCTGCCAGTGCCCCAGCCACCAGGTGGGGGTAC
TTCATCCTCATGTAGGCACTCAGCATCCCCCCATAACTGGGAGTACAGAGCACAGATCAT
GGTTGTGGGAAGCTGCCCACAACTCAGGCGAGCAGCCTCACTGTCCTCCAGGCTGAGGTG
CTAGGCTGCTCTTTCCCTGCTCAGAACGCCCAAGGGTGGGAAAGAAGGACCTGAAACTGT

>329 Get primers
GGCTGGGAAGAGAGAGGCCAGGAGAAAAGGCTGAGGAAACTGCTGGCAAATGTGAAGGGC
AAGAATGAATGCCCAAGGTGGGCAGCAGGTGAGGAAAGAGTCCCTCACCTCCCTGGAGGA
ACAAGTCTTTGATTTGCTGAAAGGCATCCCGCACAGCCTGGGCGCACTTGGGACTCTGGC
CATAAAAGTCCTGGAGAAGAGACCAAGGTTGCTGCTGCCATTCTTGCACTGGCCTGGGGT
ACCCAAGTCCCCTCACTCACCGCTGTGACATCTCGGAAGAATTGGTAGGAGTCCCCAAGG
CCTGCAACAGCTACAACAGGAGCGCTGGCTGCCAGTGCCCCAGCCACCAGGTGGGGGTAC
TTCATCCTCATGTAGGCACTCAGCATCCCCCCATAACTGGGAGTACAGAGCACAGATCAT
GGTTGTGGGAAGCTGCCCACAACTCAGGCGAGCAGCCTCACTGTCCTCCAGGCTGAGGTG
CTAGGCTGCTCTTTCCCTGCTCAGAACGCCCAAGGGTGGGAAAGAAGGACCTGAAACTGT
CAGGCCCACACACCCTGATCCCAGGGCCAAGGCAGATACAGCCTTCACTGGGAGAAGGCA

>330 Get primers
AAGAATGAATGCCCAAGGTGGGCAGCAGGTGAGGAAAGAGTCCCTCACCTCCCTGGAGGA
ACAAGTCTTTGATTTGCTGAAAGGCATCCCGCACAGCCTGGGCGCACTTGGGACTCTGGC
CATAAAAGTCCTGGAGAAGAGACCAAGGTTGCTGCTGCCATTCTTGCACTGGCCTGGGGT
ACCCAAGTCCCCTCACTCACCGCTGTGACATCTCGGAAGAATTGGTAGGAGTCCCCAAGG
CCTGCAACAGCTACAACAGGAGCGCTGGCTGCCAGTGCCCCAGCCACCAGGTGGGGGTAC
TTCATCCTCATGTAGGCACTCAGCATCCCCCCATAACTGGGAGTACAGAGCACAGATCAT
GGTTGTGGGAAGCTGCCCACAACTCAGGCGAGCAGCCTCACTGTCCTCCAGGCTGAGGTG
CTAGGCTGCTCTTTCCCTGCTCAGAACGCCCAAGGGTGGGAAAGAAGGACCTGAAACTGT
CAGGCCCACACACCCTGATCCCAGGGCCAAGGCAGATACAGCCTTCACTGGGAGAAGGCA
CCTGTGGGTGCCCTGCCCTGACCCAGCAATGAAGACATTGCAGAGACAAAGTCAGAAGGA

>331 Get primers
ACAAGTCTTTGATTTGCTGAAAGGCATCCCGCACAGCCTGGGCGCACTTGGGACTCTGGC
CATAAAAGTCCTGGAGAAGAGACCAAGGTTGCTGCTGCCATTCTTGCACTGGCCTGGGGT
ACCCAAGTCCCCTCACTCACCGCTGTGACATCTCGGAAGAATTGGTAGGAGTCCCCAAGG
CCTGCAACAGCTACAACAGGAGCGCTGGCTGCCAGTGCCCCAGCCACCAGGTGGGGGTAC
TTCATCCTCATGTAGGCACTCAGCATCCCCCCATAACTGGGAGTACAGAGCACAGATCAT
GGTTGTGGGAAGCTGCCCACAACTCAGGCGAGCAGCCTCACTGTCCTCCAGGCTGAGGTG
CTAGGCTGCTCTTTCCCTGCTCAGAACGCCCAAGGGTGGGAAAGAAGGACCTGAAACTGT
CAGGCCCACACACCCTGATCCCAGGGCCAAGGCAGATACAGCCTTCACTGGGAGAAGGCA
CCTGTGGGTGCCCTGCCCTGACCCAGCAATGAAGACATTGCAGAGACAAAGTCAGAAGGA
ATTGTCCCACTAGTGGGAACAACATAGCATACACTGCCTATGAGGTCCACTCAAGGAGGG

>332 Get primers
CATAAAAGTCCTGGAGAAGAGACCAAGGTTGCTGCTGCCATTCTTGCACTGGCCTGGGGT
ACCCAAGTCCCCTCACTCACCGCTGTGACATCTCGGAAGAATTGGTAGGAGTCCCCAAGG
CCTGCAACAGCTACAACAGGAGCGCTGGCTGCCAGTGCCCCAGCCACCAGGTGGGGGTAC
TTCATCCTCATGTAGGCACTCAGCATCCCCCCATAACTGGGAGTACAGAGCACAGATCAT
GGTTGTGGGAAGCTGCCCACAACTCAGGCGAGCAGCCTCACTGTCCTCCAGGCTGAGGTG
CTAGGCTGCTCTTTCCCTGCTCAGAACGCCCAAGGGTGGGAAAGAAGGACCTGAAACTGT
CAGGCCCACACACCCTGATCCCAGGGCCAAGGCAGATACAGCCTTCACTGGGAGAAGGCA
CCTGTGGGTGCCCTGCCCTGACCCAGCAATGAAGACATTGCAGAGACAAAGTCAGAAGGA
ATTGTCCCACTAGTGGGAACAACATAGCATACACTGCCTATGAGGTCCACTCAAGGAGGG
CTTCCAGAAGGAGGTAAAGCTAGACCCCGCCCTTCCACATGTGGGGTAGGCATAGGATGT

>333 Get primers
ACCCAAGTCCCCTCACTCACCGCTGTGACATCTCGGAAGAATTGGTAGGAGTCCCCAAGG
CCTGCAACAGCTACAACAGGAGCGCTGGCTGCCAGTGCCCCAGCCACCAGGTGGGGGTAC
TTCATCCTCATGTAGGCACTCAGCATCCCCCCATAACTGGGAGTACAGAGCACAGATCAT
GGTTGTGGGAAGCTGCCCACAACTCAGGCGAGCAGCCTCACTGTCCTCCAGGCTGAGGTG
CTAGGCTGCTCTTTCCCTGCTCAGAACGCCCAAGGGTGGGAAAGAAGGACCTGAAACTGT
CAGGCCCACACACCCTGATCCCAGGGCCAAGGCAGATACAGCCTTCACTGGGAGAAGGCA
CCTGTGGGTGCCCTGCCCTGACCCAGCAATGAAGACATTGCAGAGACAAAGTCAGAAGGA
ATTGTCCCACTAGTGGGAACAACATAGCATACACTGCCTATGAGGTCCACTCAAGGAGGG
CTTCCAGAAGGAGGTAAAGCTAGACCCCGCCCTTCCACATGTGGGGTAGGCATAGGATGT
TGAGACTGTAAGAGACATCTCTTTGGCCCTCCTTGTATAGGGTGTCAATCGGCACAACAG

>334 Get primers
CCTGCAACAGCTACAACAGGAGCGCTGGCTGCCAGTGCCCCAGCCACCAGGTGGGGGTAC
TTCATCCTCATGTAGGCACTCAGCATCCCCCCATAACTGGGAGTACAGAGCACAGATCAT
GGTTGTGGGAAGCTGCCCACAACTCAGGCGAGCAGCCTCACTGTCCTCCAGGCTGAGGTG
CTAGGCTGCTCTTTCCCTGCTCAGAACGCCCAAGGGTGGGAAAGAAGGACCTGAAACTGT
CAGGCCCACACACCCTGATCCCAGGGCCAAGGCAGATACAGCCTTCACTGGGAGAAGGCA
CCTGTGGGTGCCCTGCCCTGACCCAGCAATGAAGACATTGCAGAGACAAAGTCAGAAGGA
ATTGTCCCACTAGTGGGAACAACATAGCATACACTGCCTATGAGGTCCACTCAAGGAGGG
CTTCCAGAAGGAGGTAAAGCTAGACCCCGCCCTTCCACATGTGGGGTAGGCATAGGATGT
TGAGACTGTAAGAGACATCTCTTTGGCCCTCCTTGTATAGGGTGTCAATCGGCACAACAG
GGTGGAGCCTTAGAGTAGGGTAAGATTAGGACTCTAGGTTCTCTCATGGGTCCAGATCTG

>335 Get primers
TTCATCCTCATGTAGGCACTCAGCATCCCCCCATAACTGGGAGTACAGAGCACAGATCAT
GGTTGTGGGAAGCTGCCCACAACTCAGGCGAGCAGCCTCACTGTCCTCCAGGCTGAGGTG
CTAGGCTGCTCTTTCCCTGCTCAGAACGCCCAAGGGTGGGAAAGAAGGACCTGAAACTGT
CAGGCCCACACACCCTGATCCCAGGGCCAAGGCAGATACAGCCTTCACTGGGAGAAGGCA
CCTGTGGGTGCCCTGCCCTGACCCAGCAATGAAGACATTGCAGAGACAAAGTCAGAAGGA
ATTGTCCCACTAGTGGGAACAACATAGCATACACTGCCTATGAGGTCCACTCAAGGAGGG
CTTCCAGAAGGAGGTAAAGCTAGACCCCGCCCTTCCACATGTGGGGTAGGCATAGGATGT
TGAGACTGTAAGAGACATCTCTTTGGCCCTCCTTGTATAGGGTGTCAATCGGCACAACAG
GGTGGAGCCTTAGAGTAGGGTAAGATTAGGACTCTAGGTTCTCTCATGGGTCCAGATCTG
TCATGAAGGGAGGTCAAGGACCCACCTCCCTCCAAAGGCTATGGTGGGGGCATCATGGAC

>336 Get primers
GGTTGTGGGAAGCTGCCCACAACTCAGGCGAGCAGCCTCACTGTCCTCCAGGCTGAGGTG
CTAGGCTGCTCTTTCCCTGCTCAGAACGCCCAAGGGTGGGAAAGAAGGACCTGAAACTGT
CAGGCCCACACACCCTGATCCCAGGGCCAAGGCAGATACAGCCTTCACTGGGAGAAGGCA
CCTGTGGGTGCCCTGCCCTGACCCAGCAATGAAGACATTGCAGAGACAAAGTCAGAAGGA
ATTGTCCCACTAGTGGGAACAACATAGCATACACTGCCTATGAGGTCCACTCAAGGAGGG
CTTCCAGAAGGAGGTAAAGCTAGACCCCGCCCTTCCACATGTGGGGTAGGCATAGGATGT
TGAGACTGTAAGAGACATCTCTTTGGCCCTCCTTGTATAGGGTGTCAATCGGCACAACAG
GGTGGAGCCTTAGAGTAGGGTAAGATTAGGACTCTAGGTTCTCTCATGGGTCCAGATCTG
TCATGAAGGGAGGTCAAGGACCCACCTCCCTCCAAAGGCTATGGTGGGGGCATCATGGAC
CCCAAGGTCCTGCCGCAGGGCCTGGAGCAGCACAGCAAAGTCGGCCAGCGCCTGCTCCAC

>346 Get primers
AGTCAGCAGCTGTGTATATCCCCGCTGTGTGGACTGGACACCGAACGGAAGCGATTTCCC
ATAGTACCGCTGCAGAAAGCAGGAAGGGATGGCTAATCCACTCCTCGGTGCTCCCCACCT
CCTTCAACTCAGGGACTGCCAGGAACTGTACAGGTACCCACGTGCTCAGCAAAGACAAGC
AGGGCCTCCTGCTGGGCTGCCAGTTCCACCATGAAGCCAGAGTTGTTAGCGAAGGACCAG
ATATCCCCCTCATTCCCTGTGTAGAAAAAGATGGGCCCTTCGCCCATCTTCCAGAACTTA
TCTGTTGGAAGTAAATGAGTTTCCATAAGGCCAGGGAAACGCAGGTAGGAACCCATGCGG
TCGAGCCAGCACTCACCTGACACTAGGAACCGCTGGCCAAAGGTTTTGTTGCCGAAACTC
TCAAAGTTGAAATGGTCCATGTATTGCTCAAAATAATTCTCATGAAAGTCAGGGTCTAGA
ACTCTGTCGGCTGAGGGCAGGTGCAGAGACTCAGGAGCTGGTTGGGATCATCAGGGATCT
AGGCGGGTCAGGAGGAAGGGCAGCCAGTCTGTACTCACCTCTGGCCTGGAGGTTGCACAG

>347 Get primers
ATAGTACCGCTGCAGAAAGCAGGAAGGGATGGCTAATCCACTCCTCGGTGCTCCCCACCT
CCTTCAACTCAGGGACTGCCAGGAACTGTACAGGTACCCACGTGCTCAGCAAAGACAAGC
AGGGCCTCCTGCTGGGCTGCCAGTTCCACCATGAAGCCAGAGTTGTTAGCGAAGGACCAG
ATATCCCCCTCATTCCCTGTGTAGAAAAAGATGGGCCCTTCGCCCATCTTCCAGAACTTA
TCTGTTGGAAGTAAATGAGTTTCCATAAGGCCAGGGAAACGCAGGTAGGAACCCATGCGG
TCGAGCCAGCACTCACCTGACACTAGGAACCGCTGGCCAAAGGTTTTGTTGCCGAAACTC
TCAAAGTTGAAATGGTCCATGTATTGCTCAAAATAATTCTCATGAAAGTCAGGGTCTAGA
ACTCTGTCGGCTGAGGGCAGGTGCAGAGACTCAGGAGCTGGTTGGGATCATCAGGGATCT
AGGCGGGTCAGGAGGAAGGGCAGCCAGTCTGTACTCACCTCTGGCCTGGAGGTTGCACAG
TCCCAGTGACAGCAGCAGGACCAGGATCCAGGAGGGGACACCATGGTCCACAGGGTAACA

>348 Get primers
CCTTCAACTCAGGGACTGCCAGGAACTGTACAGGTACCCACGTGCTCAGCAAAGACAAGC
AGGGCCTCCTGCTGGGCTGCCAGTTCCACCATGAAGCCAGAGTTGTTAGCGAAGGACCAG
ATATCCCCCTCATTCCCTGTGTAGAAAAAGATGGGCCCTTCGCCCATCTTCCAGAACTTA
TCTGTTGGAAGTAAATGAGTTTCCATAAGGCCAGGGAAACGCAGGTAGGAACCCATGCGG
TCGAGCCAGCACTCACCTGACACTAGGAACCGCTGGCCAAAGGTTTTGTTGCCGAAACTC
TCAAAGTTGAAATGGTCCATGTATTGCTCAAAATAATTCTCATGAAAGTCAGGGTCTAGA
ACTCTGTCGGCTGAGGGCAGGTGCAGAGACTCAGGAGCTGGTTGGGATCATCAGGGATCT
AGGCGGGTCAGGAGGAAGGGCAGCCAGTCTGTACTCACCTCTGGCCTGGAGGTTGCACAG
TCCCAGTGACAGCAGCAGGACCAGGATCCAGGAGGGGACACCATGGTCCACAGGGTAACA
AGGATGGAAGTTCATGCTTGATTCTGAGCCGGGCGCTGACTGTCATGTGATTTGGTCACA

>349 Get primers
AGGGCCTCCTGCTGGGCTGCCAGTTCCACCATGAAGCCAGAGTTGTTAGCGAAGGACCAG
ATATCCCCCTCATTCCCTGTGTAGAAAAAGATGGGCCCTTCGCCCATCTTCCAGAACTTA
TCTGTTGGAAGTAAATGAGTTTCCATAAGGCCAGGGAAACGCAGGTAGGAACCCATGCGG
TCGAGCCAGCACTCACCTGACACTAGGAACCGCTGGCCAAAGGTTTTGTTGCCGAAACTC
TCAAAGTTGAAATGGTCCATGTATTGCTCAAAATAATTCTCATGAAAGTCAGGGTCTAGA
ACTCTGTCGGCTGAGGGCAGGTGCAGAGACTCAGGAGCTGGTTGGGATCATCAGGGATCT
AGGCGGGTCAGGAGGAAGGGCAGCCAGTCTGTACTCACCTCTGGCCTGGAGGTTGCACAG
TCCCAGTGACAGCAGCAGGACCAGGATCCAGGAGGGGACACCATGGTCCACAGGGTAACA
AGGATGGAAGTTCATGCTTGATTCTGAGCCGGGCGCTGACTGTCATGTGATTTGGTCACA
TGACCGACACAACGGGCGGGGCAGCATCACGTGATAGTCTGGCGGGGGCTGTCCTACTGT

>350 Get primers
ATATCCCCCTCATTCCCTGTGTAGAAAAAGATGGGCCCTTCGCCCATCTTCCAGAACTTA
TCTGTTGGAAGTAAATGAGTTTCCATAAGGCCAGGGAAACGCAGGTAGGAACCCATGCGG
TCGAGCCAGCACTCACCTGACACTAGGAACCGCTGGCCAAAGGTTTTGTTGCCGAAACTC
TCAAAGTTGAAATGGTCCATGTATTGCTCAAAATAATTCTCATGAAAGTCAGGGTCTAGA
ACTCTGTCGGCTGAGGGCAGGTGCAGAGACTCAGGAGCTGGTTGGGATCATCAGGGATCT
AGGCGGGTCAGGAGGAAGGGCAGCCAGTCTGTACTCACCTCTGGCCTGGAGGTTGCACAG
TCCCAGTGACAGCAGCAGGACCAGGATCCAGGAGGGGACACCATGGTCCACAGGGTAACA
AGGATGGAAGTTCATGCTTGATTCTGAGCCGGGCGCTGACTGTCATGTGATTTGGTCACA
TGACCGACACAACGGGCGGGGCAGCATCACGTGATAGTCTGGCGGGGGCTGTCCTACTGT
GGCTGGATTCTAGTTGGAGGATCAGCCTACTCTTCTTCAGTTTCCCGGTTCCTCCAAATT

>351 Get primers
TCTGTTGGAAGTAAATGAGTTTCCATAAGGCCAGGGAAACGCAGGTAGGAACCCATGCGG
TCGAGCCAGCACTCACCTGACACTAGGAACCGCTGGCCAAAGGTTTTGTTGCCGAAACTC
TCAAAGTTGAAATGGTCCATGTATTGCTCAAAATAATTCTCATGAAAGTCAGGGTCTAGA
ACTCTGTCGGCTGAGGGCAGGTGCAGAGACTCAGGAGCTGGTTGGGATCATCAGGGATCT
AGGCGGGTCAGGAGGAAGGGCAGCCAGTCTGTACTCACCTCTGGCCTGGAGGTTGCACAG
TCCCAGTGACAGCAGCAGGACCAGGATCCAGGAGGGGACACCATGGTCCACAGGGTAACA
AGGATGGAAGTTCATGCTTGATTCTGAGCCGGGCGCTGACTGTCATGTGATTTGGTCACA
TGACCGACACAACGGGCGGGGCAGCATCACGTGATAGTCTGGCGGGGGCTGTCCTACTGT
GGCTGGATTCTAGTTGGAGGATCAGCCTACTCTTCTTCAGTTTCCCGGTTCCTCCAAATT
TCTGGGCTCCTACTTGTTTCCACAGAGATGGATACTGTGGAGGTCCAGGAAGCAGAGAGA

>352 Get primers
TCGAGCCAGCACTCACCTGACACTAGGAACCGCTGGCCAAAGGTTTTGTTGCCGAAACTC
TCAAAGTTGAAATGGTCCATGTATTGCTCAAAATAATTCTCATGAAAGTCAGGGTCTAGA
ACTCTGTCGGCTGAGGGCAGGTGCAGAGACTCAGGAGCTGGTTGGGATCATCAGGGATCT
AGGCGGGTCAGGAGGAAGGGCAGCCAGTCTGTACTCACCTCTGGCCTGGAGGTTGCACAG
TCCCAGTGACAGCAGCAGGACCAGGATCCAGGAGGGGACACCATGGTCCACAGGGTAACA
AGGATGGAAGTTCATGCTTGATTCTGAGCCGGGCGCTGACTGTCATGTGATTTGGTCACA
TGACCGACACAACGGGCGGGGCAGCATCACGTGATAGTCTGGCGGGGGCTGTCCTACTGT
GGCTGGATTCTAGTTGGAGGATCAGCCTACTCTTCTTCAGTTTCCCGGTTCCTCCAAATT
TCTGGGCTCCTACTTGTTTCCACAGAGATGGATACTGTGGAGGTCCAGGAAGCAGAGAGA
TGGCTAAGGCTCATCAGGACCGTATGATCTCCCAAGTGTCCAGCTACTGAGTACCACAAG

>353 Get primers
TCAAAGTTGAAATGGTCCATGTATTGCTCAAAATAATTCTCATGAAAGTCAGGGTCTAGA
ACTCTGTCGGCTGAGGGCAGGTGCAGAGACTCAGGAGCTGGTTGGGATCATCAGGGATCT
AGGCGGGTCAGGAGGAAGGGCAGCCAGTCTGTACTCACCTCTGGCCTGGAGGTTGCACAG
TCCCAGTGACAGCAGCAGGACCAGGATCCAGGAGGGGACACCATGGTCCACAGGGTAACA
AGGATGGAAGTTCATGCTTGATTCTGAGCCGGGCGCTGACTGTCATGTGATTTGGTCACA
TGACCGACACAACGGGCGGGGCAGCATCACGTGATAGTCTGGCGGGGGCTGTCCTACTGT
GGCTGGATTCTAGTTGGAGGATCAGCCTACTCTTCTTCAGTTTCCCGGTTCCTCCAAATT
TCTGGGCTCCTACTTGTTTCCACAGAGATGGATACTGTGGAGGTCCAGGAAGCAGAGAGA
TGGCTAAGGCTCATCAGGACCGTATGATCTCCCAAGTGTCCAGCTACTGAGTACCACAAG
GTGATGGGTGGGAGGGTCCTCCCACGGAAGGATACCGCAGTCCCTAGGGGTTGCAAGCCC

>354 Get primers
ACTCTGTCGGCTGAGGGCAGGTGCAGAGACTCAGGAGCTGGTTGGGATCATCAGGGATCT
AGGCGGGTCAGGAGGAAGGGCAGCCAGTCTGTACTCACCTCTGGCCTGGAGGTTGCACAG
TCCCAGTGACAGCAGCAGGACCAGGATCCAGGAGGGGACACCATGGTCCACAGGGTAACA
AGGATGGAAGTTCATGCTTGATTCTGAGCCGGGCGCTGACTGTCATGTGATTTGGTCACA
TGACCGACACAACGGGCGGGGCAGCATCACGTGATAGTCTGGCGGGGGCTGTCCTACTGT
GGCTGGATTCTAGTTGGAGGATCAGCCTACTCTTCTTCAGTTTCCCGGTTCCTCCAAATT
TCTGGGCTCCTACTTGTTTCCACAGAGATGGATACTGTGGAGGTCCAGGAAGCAGAGAGA
TGGCTAAGGCTCATCAGGACCGTATGATCTCCCAAGTGTCCAGCTACTGAGTACCACAAG
GTGATGGGTGGGAGGGTCCTCCCACGGAAGGATACCGCAGTCCCTAGGGGTTGCAAGCCC
CACATGTTCCACTGGCTGCTAGAGCTACCTACTCAATCAGCCCTGGGCATCACCATCAGG

>355 Get primers
AGGCGGGTCAGGAGGAAGGGCAGCCAGTCTGTACTCACCTCTGGCCTGGAGGTTGCACAG
TCCCAGTGACAGCAGCAGGACCAGGATCCAGGAGGGGACACCATGGTCCACAGGGTAACA
AGGATGGAAGTTCATGCTTGATTCTGAGCCGGGCGCTGACTGTCATGTGATTTGGTCACA
TGACCGACACAACGGGCGGGGCAGCATCACGTGATAGTCTGGCGGGGGCTGTCCTACTGT
GGCTGGATTCTAGTTGGAGGATCAGCCTACTCTTCTTCAGTTTCCCGGTTCCTCCAAATT
TCTGGGCTCCTACTTGTTTCCACAGAGATGGATACTGTGGAGGTCCAGGAAGCAGAGAGA
TGGCTAAGGCTCATCAGGACCGTATGATCTCCCAAGTGTCCAGCTACTGAGTACCACAAG
GTGATGGGTGGGAGGGTCCTCCCACGGAAGGATACCGCAGTCCCTAGGGGTTGCAAGCCC
CACATGTTCCACTGGCTGCTAGAGCTACCTACTCAATCAGCCCTGGGCATCACCATCAGG
TACTCGGCCAAAATGACCTCTCTGCTTCCAGTCCTCAGTTCTGGTCAGCACCAGACAGGC

>356 Get primers
TCCCAGTGACAGCAGCAGGACCAGGATCCAGGAGGGGACACCATGGTCCACAGGGTAACA
AGGATGGAAGTTCATGCTTGATTCTGAGCCGGGCGCTGACTGTCATGTGATTTGGTCACA
TGACCGACACAACGGGCGGGGCAGCATCACGTGATAGTCTGGCGGGGGCTGTCCTACTGT
GGCTGGATTCTAGTTGGAGGATCAGCCTACTCTTCTTCAGTTTCCCGGTTCCTCCAAATT
TCTGGGCTCCTACTTGTTTCCACAGAGATGGATACTGTGGAGGTCCAGGAAGCAGAGAGA
TGGCTAAGGCTCATCAGGACCGTATGATCTCCCAAGTGTCCAGCTACTGAGTACCACAAG
GTGATGGGTGGGAGGGTCCTCCCACGGAAGGATACCGCAGTCCCTAGGGGTTGCAAGCCC
CACATGTTCCACTGGCTGCTAGAGCTACCTACTCAATCAGCCCTGGGCATCACCATCAGG
TACTCGGCCAAAATGACCTCTCTGCTTCCAGTCCTCAGTTCTGGTCAGCACCAGACAGGC
CCATAATTACAGAGCCAGGGAAACTGGAACATTTGTCTCCCCTTAGACAGTGGCAGCAGG

>357 Get primers
AGGATGGAAGTTCATGCTTGATTCTGAGCCGGGCGCTGACTGTCATGTGATTTGGTCACA
TGACCGACACAACGGGCGGGGCAGCATCACGTGATAGTCTGGCGGGGGCTGTCCTACTGT
GGCTGGATTCTAGTTGGAGGATCAGCCTACTCTTCTTCAGTTTCCCGGTTCCTCCAAATT
TCTGGGCTCCTACTTGTTTCCACAGAGATGGATACTGTGGAGGTCCAGGAAGCAGAGAGA
TGGCTAAGGCTCATCAGGACCGTATGATCTCCCAAGTGTCCAGCTACTGAGTACCACAAG
GTGATGGGTGGGAGGGTCCTCCCACGGAAGGATACCGCAGTCCCTAGGGGTTGCAAGCCC
CACATGTTCCACTGGCTGCTAGAGCTACCTACTCAATCAGCCCTGGGCATCACCATCAGG
TACTCGGCCAAAATGACCTCTCTGCTTCCAGTCCTCAGTTCTGGTCAGCACCAGACAGGC
CCATAATTACAGAGCCAGGGAAACTGGAACATTTGTCTCCCCTTAGACAGTGGCAGCAGG
AAGGTGGGGGGTTGTTGCAGAGGAACAGTGTCTCTGAGAGAGGACCTTGGACTTTCTGGG

>358 Get primers
TGACCGACACAACGGGCGGGGCAGCATCACGTGATAGTCTGGCGGGGGCTGTCCTACTGT
GGCTGGATTCTAGTTGGAGGATCAGCCTACTCTTCTTCAGTTTCCCGGTTCCTCCAAATT
TCTGGGCTCCTACTTGTTTCCACAGAGATGGATACTGTGGAGGTCCAGGAAGCAGAGAGA
TGGCTAAGGCTCATCAGGACCGTATGATCTCCCAAGTGTCCAGCTACTGAGTACCACAAG
GTGATGGGTGGGAGGGTCCTCCCACGGAAGGATACCGCAGTCCCTAGGGGTTGCAAGCCC
CACATGTTCCACTGGCTGCTAGAGCTACCTACTCAATCAGCCCTGGGCATCACCATCAGG
TACTCGGCCAAAATGACCTCTCTGCTTCCAGTCCTCAGTTCTGGTCAGCACCAGACAGGC
CCATAATTACAGAGCCAGGGAAACTGGAACATTTGTCTCCCCTTAGACAGTGGCAGCAGG
AAGGTGGGGGGTTGTTGCAGAGGAACAGTGTCTCTGAGAGAGGACCTTGGACTTTCTGGG
AATCTCTGAGCTGCCCGGTTCTCCCCACTGCTGGCACTGTGCCCACAGCCCAAACAGAAT

>359 Get primers
GGCTGGATTCTAGTTGGAGGATCAGCCTACTCTTCTTCAGTTTCCCGGTTCCTCCAAATT
TCTGGGCTCCTACTTGTTTCCACAGAGATGGATACTGTGGAGGTCCAGGAAGCAGAGAGA
TGGCTAAGGCTCATCAGGACCGTATGATCTCCCAAGTGTCCAGCTACTGAGTACCACAAG
GTGATGGGTGGGAGGGTCCTCCCACGGAAGGATACCGCAGTCCCTAGGGGTTGCAAGCCC
CACATGTTCCACTGGCTGCTAGAGCTACCTACTCAATCAGCCCTGGGCATCACCATCAGG
TACTCGGCCAAAATGACCTCTCTGCTTCCAGTCCTCAGTTCTGGTCAGCACCAGACAGGC
CCATAATTACAGAGCCAGGGAAACTGGAACATTTGTCTCCCCTTAGACAGTGGCAGCAGG
AAGGTGGGGGGTTGTTGCAGAGGAACAGTGTCTCTGAGAGAGGACCTTGGACTTTCTGGG
AATCTCTGAGCTGCCCGGTTCTCCCCACTGCTGGCACTGTGCCCACAGCCCAAACAGAAT
GGGGGAGATGGAGGGGCAGGGCTTCTGTGGGAAGCTGCCCTCCACCTCATTGGCACAGAG

>360 Get primers
TCTGGGCTCCTACTTGTTTCCACAGAGATGGATACTGTGGAGGTCCAGGAAGCAGAGAGA
TGGCTAAGGCTCATCAGGACCGTATGATCTCCCAAGTGTCCAGCTACTGAGTACCACAAG
GTGATGGGTGGGAGGGTCCTCCCACGGAAGGATACCGCAGTCCCTAGGGGTTGCAAGCCC
CACATGTTCCACTGGCTGCTAGAGCTACCTACTCAATCAGCCCTGGGCATCACCATCAGG
TACTCGGCCAAAATGACCTCTCTGCTTCCAGTCCTCAGTTCTGGTCAGCACCAGACAGGC
CCATAATTACAGAGCCAGGGAAACTGGAACATTTGTCTCCCCTTAGACAGTGGCAGCAGG
AAGGTGGGGGGTTGTTGCAGAGGAACAGTGTCTCTGAGAGAGGACCTTGGACTTTCTGGG
AATCTCTGAGCTGCCCGGTTCTCCCCACTGCTGGCACTGTGCCCACAGCCCAAACAGAAT
GGGGGAGATGGAGGGGCAGGGCTTCTGTGGGAAGCTGCCCTCCACCTCATTGGCACAGAG
TGTCTCATTGCAGAGAGAAAAAAGGACCAGTTTTCTCTCTGGCACCCAGGTCTGGAAGAG

>361 Get primers
TGGCTAAGGCTCATCAGGACCGTATGATCTCCCAAGTGTCCAGCTACTGAGTACCACAAG
GTGATGGGTGGGAGGGTCCTCCCACGGAAGGATACCGCAGTCCCTAGGGGTTGCAAGCCC
CACATGTTCCACTGGCTGCTAGAGCTACCTACTCAATCAGCCCTGGGCATCACCATCAGG
TACTCGGCCAAAATGACCTCTCTGCTTCCAGTCCTCAGTTCTGGTCAGCACCAGACAGGC
CCATAATTACAGAGCCAGGGAAACTGGAACATTTGTCTCCCCTTAGACAGTGGCAGCAGG
AAGGTGGGGGGTTGTTGCAGAGGAACAGTGTCTCTGAGAGAGGACCTTGGACTTTCTGGG
AATCTCTGAGCTGCCCGGTTCTCCCCACTGCTGGCACTGTGCCCACAGCCCAAACAGAAT
GGGGGAGATGGAGGGGCAGGGCTTCTGTGGGAAGCTGCCCTCCACCTCATTGGCACAGAG
TGTCTCATTGCAGAGAGAAAAAAGGACCAGTTTTCTCTCTGGCACCCAGGTCTGGAAGAG
GAGTGACATCCACGGAAGTTGGTGACTTGGACTGGCTGGCCGTGAGTGGAACATGTCCAT

>362 Get primers
GTGATGGGTGGGAGGGTCCTCCCACGGAAGGATACCGCAGTCCCTAGGGGTTGCAAGCCC
CACATGTTCCACTGGCTGCTAGAGCTACCTACTCAATCAGCCCTGGGCATCACCATCAGG
TACTCGGCCAAAATGACCTCTCTGCTTCCAGTCCTCAGTTCTGGTCAGCACCAGACAGGC
CCATAATTACAGAGCCAGGGAAACTGGAACATTTGTCTCCCCTTAGACAGTGGCAGCAGG
AAGGTGGGGGGTTGTTGCAGAGGAACAGTGTCTCTGAGAGAGGACCTTGGACTTTCTGGG
AATCTCTGAGCTGCCCGGTTCTCCCCACTGCTGGCACTGTGCCCACAGCCCAAACAGAAT
GGGGGAGATGGAGGGGCAGGGCTTCTGTGGGAAGCTGCCCTCCACCTCATTGGCACAGAG
TGTCTCATTGCAGAGAGAAAAAAGGACCAGTTTTCTCTCTGGCACCCAGGTCTGGAAGAG
GAGTGACATCCACGGAAGTTGGTGACTTGGACTGGCTGGCCGTGAGTGGAACATGTCCAT
CCAGCATGGCCACAGTCCAGTGGGACACACAGCCTAGAGCTGTGGAATGCCGTGCCACAG

>363 Get primers
AGGCCAAGAAGTAGACCCACCTGCCAGTGCCCGTAGACCCATGATCCTCTGGCCCTCATT
CAACAGCCGTTGACAGCCCACCTAGAGAAAGGCAGCAGAATATCTCAGTGGAGGCCCCTT
TCACAGAGCGTGGGTCAGGGCTGCTAGCTTCCAGGACACAACAGCAGATAGTGTCTGATG
GCATGAAAGCAGATAGCTACAAGGCTCTTGGACCAGGCTAGCACTGGGTCCTGCACCCAG
GGAGAGCCACCTCACCTTGACAGGGTTGGCAGGAAGGGGCCCTAGAAAGTCAGTAGGATA
CGGGTAGTCCATCATGGCGAGCACAGTAAATGCATTTCGGGCAAACCCAAAGAGCTGAGT
CAGGTCCTTTGGGCTGGAAAGTGATTGACAGGTACCAAAGTTCTGGCTGATGGTGTCATA
GGCTGGGAAGAGAGAGGCCAGGAGAAAAGGCTGAGGAAACTGCTGGCAAATGTGAAGGGC
AAGAATGAATGCCCAAGGTGGGCAGCAGGTGAGGAAAGAGTCCCTCACCTCCCTGGAGGA
ACAAGTCTTT

>364 Get primers
TCATTCAACAGCCGTTGACAGCCCACCTAGAGAAAGGCAGCAGAATATCTCAGTGGAGGC
CCCTTTCACAGAGCGTGGGTCAGGGCTGCTAGCTTCCAGGACACAACAGCAGATAGTGTC
TGATGGCATGAAAGCAGATAGCTACAAGGCTCTTGGACCAGGCTAGCACTGGGTCCTGCA
CCCAGGGAGAGCCACCTCACCTTGACAGGGTTGGCAGGAAGGGGCCCTAGAAAGTCAGTA
GGATACGGGTAGTCCATCATGGCGAGCACAGTAAATGCATTTCGGGCAAACCCAAAGAGC
TGAGTCAGGTCCTTTGGGCTGGAAAGTGATTGACAGGTACCAAAGTTCTGGCTGATGGTG
TCATAGGCTGGGAAGAGAGAGGCCAGGAGAAAAGGCTGAGGAAACTGCTGGCAAATGTGA
AGGGCAAGAATGAATGCCCAAGGTGGGCAGCAGGTGAGGAAAGAGTCCCTCACCTCCCTG
GAGGAACAAGTCTTTGATTTGCTGAAAGGCATCCCGCACAGCCTGGGCGCACTTGGGACT
CTGGCCATAA

>365 Get primers
GAGGCCCCTTTCACAGAGCGTGGGTCAGGGCTGCTAGCTTCCAGGACACAACAGCAGATA
GTGTCTGATGGCATGAAAGCAGATAGCTACAAGGCTCTTGGACCAGGCTAGCACTGGGTC
CTGCACCCAGGGAGAGCCACCTCACCTTGACAGGGTTGGCAGGAAGGGGCCCTAGAAAGT
CAGTAGGATACGGGTAGTCCATCATGGCGAGCACAGTAAATGCATTTCGGGCAAACCCAA
AGAGCTGAGTCAGGTCCTTTGGGCTGGAAAGTGATTGACAGGTACCAAAGTTCTGGCTGA
TGGTGTCATAGGCTGGGAAGAGAGAGGCCAGGAGAAAAGGCTGAGGAAACTGCTGGCAAA
TGTGAAGGGCAAGAATGAATGCCCAAGGTGGGCAGCAGGTGAGGAAAGAGTCCCTCACCT
CCCTGGAGGAACAAGTCTTTGATTTGCTGAAAGGCATCCCGCACAGCCTGGGCGCACTTG
GGACTCTGGCCATAAAAGTCCTGGAGAAGAGACCAAGGTTGCTGCTGCCATTCTTGCACT
GGCCTGGGGT

>366 Get primers
AGATAGTGTCTGATGGCATGAAAGCAGATAGCTACAAGGCTCTTGGACCAGGCTAGCACT
GGGTCCTGCACCCAGGGAGAGCCACCTCACCTTGACAGGGTTGGCAGGAAGGGGCCCTAG
AAAGTCAGTAGGATACGGGTAGTCCATCATGGCGAGCACAGTAAATGCATTTCGGGCAAA
CCCAAAGAGCTGAGTCAGGTCCTTTGGGCTGGAAAGTGATTGACAGGTACCAAAGTTCTG
GCTGATGGTGTCATAGGCTGGGAAGAGAGAGGCCAGGAGAAAAGGCTGAGGAAACTGCTG
GCAAATGTGAAGGGCAAGAATGAATGCCCAAGGTGGGCAGCAGGTGAGGAAAGAGTCCCT
CACCTCCCTGGAGGAACAAGTCTTTGATTTGCTGAAAGGCATCCCGCACAGCCTGGGCGC
ACTTGGGACTCTGGCCATAAAAGTCCTGGAGAAGAGACCAAGGTTGCTGCTGCCATTCTT
GCACTGGCCTGGGGTACCCAAGTCCCCTCACTCACCGCTGTGACATCTCGGAAGAATTGG
TAGGAGTCCC

>367 Get primers
GCACTGGGTCCTGCACCCAGGGAGAGCCACCTCACCTTGACAGGGTTGGCAGGAAGGGGC
CCTAGAAAGTCAGTAGGATACGGGTAGTCCATCATGGCGAGCACAGTAAATGCATTTCGG
GCAAACCCAAAGAGCTGAGTCAGGTCCTTTGGGCTGGAAAGTGATTGACAGGTACCAAAG
TTCTGGCTGATGGTGTCATAGGCTGGGAAGAGAGAGGCCAGGAGAAAAGGCTGAGGAAAC
TGCTGGCAAATGTGAAGGGCAAGAATGAATGCCCAAGGTGGGCAGCAGGTGAGGAAAGAG
TCCCTCACCTCCCTGGAGGAACAAGTCTTTGATTTGCTGAAAGGCATCCCGCACAGCCTG
GGCGCACTTGGGACTCTGGCCATAAAAGTCCTGGAGAAGAGACCAAGGTTGCTGCTGCCA
TTCTTGCACTGGCCTGGGGTACCCAAGTCCCCTCACTCACCGCTGTGACATCTCGGAAGA
ATTGGTAGGAGTCCCCAAGGCCTGCAACAGCTACAACAGGAGCGCTGGCTGCCAGTGCCC
CAGCCACCAG

>368 Get primers
GGGGCCCTAGAAAGTCAGTAGGATACGGGTAGTCCATCATGGCGAGCACAGTAAATGCAT
TTCGGGCAAACCCAAAGAGCTGAGTCAGGTCCTTTGGGCTGGAAAGTGATTGACAGGTAC
CAAAGTTCTGGCTGATGGTGTCATAGGCTGGGAAGAGAGAGGCCAGGAGAAAAGGCTGAG
GAAACTGCTGGCAAATGTGAAGGGCAAGAATGAATGCCCAAGGTGGGCAGCAGGTGAGGA
AAGAGTCCCTCACCTCCCTGGAGGAACAAGTCTTTGATTTGCTGAAAGGCATCCCGCACA
GCCTGGGCGCACTTGGGACTCTGGCCATAAAAGTCCTGGAGAAGAGACCAAGGTTGCTGC
TGCCATTCTTGCACTGGCCTGGGGTACCCAAGTCCCCTCACTCACCGCTGTGACATCTCG
GAAGAATTGGTAGGAGTCCCCAAGGCCTGCAACAGCTACAACAGGAGCGCTGGCTGCCAG
TGCCCCAGCCACCAGGTGGGGGTACTTCATCCTCATGTAGGCACTCAGCATCCCCCCATA
ACTGGGAGTA

>369 Get primers
TGCATTTCGGGCAAACCCAAAGAGCTGAGTCAGGTCCTTTGGGCTGGAAAGTGATTGACA
GGTACCAAAGTTCTGGCTGATGGTGTCATAGGCTGGGAAGAGAGAGGCCAGGAGAAAAGG
CTGAGGAAACTGCTGGCAAATGTGAAGGGCAAGAATGAATGCCCAAGGTGGGCAGCAGGT
GAGGAAAGAGTCCCTCACCTCCCTGGAGGAACAAGTCTTTGATTTGCTGAAAGGCATCCC
GCACAGCCTGGGCGCACTTGGGACTCTGGCCATAAAAGTCCTGGAGAAGAGACCAAGGTT
GCTGCTGCCATTCTTGCACTGGCCTGGGGTACCCAAGTCCCCTCACTCACCGCTGTGACA
TCTCGGAAGAATTGGTAGGAGTCCCCAAGGCCTGCAACAGCTACAACAGGAGCGCTGGCT
GCCAGTGCCCCAGCCACCAGGTGGGGGTACTTCATCCTCATGTAGGCACTCAGCATCCCC
CCATAACTGGGAGTACAGAGCACAGATCATGGTTGTGGGAAGCTGCCCACAACTCAGGCG
AGCAGCCTCA

>370 Get primers
TGACAGGTACCAAAGTTCTGGCTGATGGTGTCATAGGCTGGGAAGAGAGAGGCCAGGAGA
AAAGGCTGAGGAAACTGCTGGCAAATGTGAAGGGCAAGAATGAATGCCCAAGGTGGGCAG
CAGGTGAGGAAAGAGTCCCTCACCTCCCTGGAGGAACAAGTCTTTGATTTGCTGAAAGGC
ATCCCGCACAGCCTGGGCGCACTTGGGACTCTGGCCATAAAAGTCCTGGAGAAGAGACCA
AGGTTGCTGCTGCCATTCTTGCACTGGCCTGGGGTACCCAAGTCCCCTCACTCACCGCTG
TGACATCTCGGAAGAATTGGTAGGAGTCCCCAAGGCCTGCAACAGCTACAACAGGAGCGC
TGGCTGCCAGTGCCCCAGCCACCAGGTGGGGGTACTTCATCCTCATGTAGGCACTCAGCA
TCCCCCCATAACTGGGAGTACAGAGCACAGATCATGGTTGTGGGAAGCTGCCCACAACTC
AGGCGAGCAGCCTCACTGTCCTCCAGGCTGAGGTGCTAGGCTGCTCTTTCCCTGCTCAGA
ACGCCCAAGG

>371 Get primers
GGAGAAAAGGCTGAGGAAACTGCTGGCAAATGTGAAGGGCAAGAATGAATGCCCAAGGTG
GGCAGCAGGTGAGGAAAGAGTCCCTCACCTCCCTGGAGGAACAAGTCTTTGATTTGCTGA
AAGGCATCCCGCACAGCCTGGGCGCACTTGGGACTCTGGCCATAAAAGTCCTGGAGAAGA
GACCAAGGTTGCTGCTGCCATTCTTGCACTGGCCTGGGGTACCCAAGTCCCCTCACTCAC
CGCTGTGACATCTCGGAAGAATTGGTAGGAGTCCCCAAGGCCTGCAACAGCTACAACAGG
AGCGCTGGCTGCCAGTGCCCCAGCCACCAGGTGGGGGTACTTCATCCTCATGTAGGCACT
CAGCATCCCCCCATAACTGGGAGTACAGAGCACAGATCATGGTTGTGGGAAGCTGCCCAC
AACTCAGGCGAGCAGCCTCACTGTCCTCCAGGCTGAGGTGCTAGGCTGCTCTTTCCCTGC
TCAGAACGCCCAAGGGTGGGAAAGAAGGACCTGAAACTGTCAGGCCCACACACCCTGATC
CCAGGGCCAA

>372 Get primers
AGGTGGGCAGCAGGTGAGGAAAGAGTCCCTCACCTCCCTGGAGGAACAAGTCTTTGATTT
GCTGAAAGGCATCCCGCACAGCCTGGGCGCACTTGGGACTCTGGCCATAAAAGTCCTGGA
GAAGAGACCAAGGTTGCTGCTGCCATTCTTGCACTGGCCTGGGGTACCCAAGTCCCCTCA
CTCACCGCTGTGACATCTCGGAAGAATTGGTAGGAGTCCCCAAGGCCTGCAACAGCTACA
ACAGGAGCGCTGGCTGCCAGTGCCCCAGCCACCAGGTGGGGGTACTTCATCCTCATGTAG
GCACTCAGCATCCCCCCATAACTGGGAGTACAGAGCACAGATCATGGTTGTGGGAAGCTG
CCCACAACTCAGGCGAGCAGCCTCACTGTCCTCCAGGCTGAGGTGCTAGGCTGCTCTTTC
CCTGCTCAGAACGCCCAAGGGTGGGAAAGAAGGACCTGAAACTGTCAGGCCCACACACCC
TGATCCCAGGGCCAAGGCAGATACAGCCTTCACTGGGAGAAGGCACCTGTGGGTGCCCTG
CCCTGACCCA

>373 Get primers
GATTTGCTGAAAGGCATCCCGCACAGCCTGGGCGCACTTGGGACTCTGGCCATAAAAGTC
CTGGAGAAGAGACCAAGGTTGCTGCTGCCATTCTTGCACTGGCCTGGGGTACCCAAGTCC
CCTCACTCACCGCTGTGACATCTCGGAAGAATTGGTAGGAGTCCCCAAGGCCTGCAACAG
CTACAACAGGAGCGCTGGCTGCCAGTGCCCCAGCCACCAGGTGGGGGTACTTCATCCTCA
TGTAGGCACTCAGCATCCCCCCATAACTGGGAGTACAGAGCACAGATCATGGTTGTGGGA
AGCTGCCCACAACTCAGGCGAGCAGCCTCACTGTCCTCCAGGCTGAGGTGCTAGGCTGCT
CTTTCCCTGCTCAGAACGCCCAAGGGTGGGAAAGAAGGACCTGAAACTGTCAGGCCCACA
CACCCTGATCCCAGGGCCAAGGCAGATACAGCCTTCACTGGGAGAAGGCACCTGTGGGTG
CCCTGCCCTGACCCAGCAATGAAGACATTGCAGAGACAAAGTCAGAAGGAATTGTCCCAC
TAGTGGGAAC

>374 Get primers
AAGTCCTGGAGAAGAGACCAAGGTTGCTGCTGCCATTCTTGCACTGGCCTGGGGTACCCA
AGTCCCCTCACTCACCGCTGTGACATCTCGGAAGAATTGGTAGGAGTCCCCAAGGCCTGC
AACAGCTACAACAGGAGCGCTGGCTGCCAGTGCCCCAGCCACCAGGTGGGGGTACTTCAT
CCTCATGTAGGCACTCAGCATCCCCCCATAACTGGGAGTACAGAGCACAGATCATGGTTG
TGGGAAGCTGCCCACAACTCAGGCGAGCAGCCTCACTGTCCTCCAGGCTGAGGTGCTAGG
CTGCTCTTTCCCTGCTCAGAACGCCCAAGGGTGGGAAAGAAGGACCTGAAACTGTCAGGC
CCACACACCCTGATCCCAGGGCCAAGGCAGATACAGCCTTCACTGGGAGAAGGCACCTGT
GGGTGCCCTGCCCTGACCCAGCAATGAAGACATTGCAGAGACAAAGTCAGAAGGAATTGT
CCCACTAGTGGGAACAACATAGCATACACTGCCTATGAGGTCCACTCAAGGAGGGCTTCC
AGAAGGAGGT

>375 Get primers
ACCCAAGTCCCCTCACTCACCGCTGTGACATCTCGGAAGAATTGGTAGGAGTCCCCAAGG
CCTGCAACAGCTACAACAGGAGCGCTGGCTGCCAGTGCCCCAGCCACCAGGTGGGGGTAC
TTCATCCTCATGTAGGCACTCAGCATCCCCCCATAACTGGGAGTACAGAGCACAGATCAT
GGTTGTGGGAAGCTGCCCACAACTCAGGCGAGCAGCCTCACTGTCCTCCAGGCTGAGGTG
CTAGGCTGCTCTTTCCCTGCTCAGAACGCCCAAGGGTGGGAAAGAAGGACCTGAAACTGT
CAGGCCCACACACCCTGATCCCAGGGCCAAGGCAGATACAGCCTTCACTGGGAGAAGGCA
CCTGTGGGTGCCCTGCCCTGACCCAGCAATGAAGACATTGCAGAGACAAAGTCAGAAGGA
ATTGTCCCACTAGTGGGAACAACATAGCATACACTGCCTATGAGGTCCACTCAAGGAGGG
CTTCCAGAAGGAGGTAAAGCTAGACCCCGCCCTTCCACATGTGGGGTAGGCATAGGATGT
TGAGACTGTA

>376 Get primers
CAAGGCCTGCAACAGCTACAACAGGAGCGCTGGCTGCCAGTGCCCCAGCCACCAGGTGGG
GGTACTTCATCCTCATGTAGGCACTCAGCATCCCCCCATAACTGGGAGTACAGAGCACAG
ATCATGGTTGTGGGAAGCTGCCCACAACTCAGGCGAGCAGCCTCACTGTCCTCCAGGCTG
AGGTGCTAGGCTGCTCTTTCCCTGCTCAGAACGCCCAAGGGTGGGAAAGAAGGACCTGAA
ACTGTCAGGCCCACACACCCTGATCCCAGGGCCAAGGCAGATACAGCCTTCACTGGGAGA
AGGCACCTGTGGGTGCCCTGCCCTGACCCAGCAATGAAGACATTGCAGAGACAAAGTCAG
AAGGAATTGTCCCACTAGTGGGAACAACATAGCATACACTGCCTATGAGGTCCACTCAAG
GAGGGCTTCCAGAAGGAGGTAAAGCTAGACCCCGCCCTTCCACATGTGGGGTAGGCATAG
GATGTTGAGACTGTAAGAGACATCTCTTTGGCCCTCCTTGTATAGGGTGTCAATCGGCAC
AACAGGGTGG

>377 Get primers
GTGGGGGTACTTCATCCTCATGTAGGCACTCAGCATCCCCCCATAACTGGGAGTACAGAG
CACAGATCATGGTTGTGGGAAGCTGCCCACAACTCAGGCGAGCAGCCTCACTGTCCTCCA
GGCTGAGGTGCTAGGCTGCTCTTTCCCTGCTCAGAACGCCCAAGGGTGGGAAAGAAGGAC
CTGAAACTGTCAGGCCCACACACCCTGATCCCAGGGCCAAGGCAGATACAGCCTTCACTG
GGAGAAGGCACCTGTGGGTGCCCTGCCCTGACCCAGCAATGAAGACATTGCAGAGACAAA
GTCAGAAGGAATTGTCCCACTAGTGGGAACAACATAGCATACACTGCCTATGAGGTCCAC
TCAAGGAGGGCTTCCAGAAGGAGGTAAAGCTAGACCCCGCCCTTCCACATGTGGGGTAGG
CATAGGATGTTGAGACTGTAAGAGACATCTCTTTGGCCCTCCTTGTATAGGGTGTCAATC
GGCACAACAGGGTGGAGCCTTAGAGTAGGGTAAGATTAGGACTCTAGGTTCTCTCATGGG
TCCAGATCTG

>378 Get primers
CAGAGCACAGATCATGGTTGTGGGAAGCTGCCCACAACTCAGGCGAGCAGCCTCACTGTC
CTCCAGGCTGAGGTGCTAGGCTGCTCTTTCCCTGCTCAGAACGCCCAAGGGTGGGAAAGA
AGGACCTGAAACTGTCAGGCCCACACACCCTGATCCCAGGGCCAAGGCAGATACAGCCTT
CACTGGGAGAAGGCACCTGTGGGTGCCCTGCCCTGACCCAGCAATGAAGACATTGCAGAG
ACAAAGTCAGAAGGAATTGTCCCACTAGTGGGAACAACATAGCATACACTGCCTATGAGG
TCCACTCAAGGAGGGCTTCCAGAAGGAGGTAAAGCTAGACCCCGCCCTTCCACATGTGGG
GTAGGCATAGGATGTTGAGACTGTAAGAGACATCTCTTTGGCCCTCCTTGTATAGGGTGT
CAATCGGCACAACAGGGTGGAGCCTTAGAGTAGGGTAAGATTAGGACTCTAGGTTCTCTC
ATGGGTCCAGATCTGTCATGAAGGGAGGTCAAGGACCCACCTCCCTCCAAAGGCTATGGT
GGGGGCATCA

>379 Get primers
CTGTCCTCCAGGCTGAGGTGCTAGGCTGCTCTTTCCCTGCTCAGAACGCCCAAGGGTGGG
AAAGAAGGACCTGAAACTGTCAGGCCCACACACCCTGATCCCAGGGCCAAGGCAGATACA
GCCTTCACTGGGAGAAGGCACCTGTGGGTGCCCTGCCCTGACCCAGCAATGAAGACATTG
CAGAGACAAAGTCAGAAGGAATTGTCCCACTAGTGGGAACAACATAGCATACACTGCCTA
TGAGGTCCACTCAAGGAGGGCTTCCAGAAGGAGGTAAAGCTAGACCCCGCCCTTCCACAT
GTGGGGTAGGCATAGGATGTTGAGACTGTAAGAGACATCTCTTTGGCCCTCCTTGTATAG
GGTGTCAATCGGCACAACAGGGTGGAGCCTTAGAGTAGGGTAAGATTAGGACTCTAGGTT
CTCTCATGGGTCCAGATCTGTCATGAAGGGAGGTCAAGGACCCACCTCCCTCCAAAGGCT
ATGGTGGGGGCATCATGGACCCCAAGGTCCTGCCGCAGGGCCTGGAGCAGCACAGCAAAG
TCGGCCAGCG

>389 Get primers
CCTGCTCCACAGTCAGCAGCTGTGTATATCCCCGCTGTGTGGACTGGACACCGAACGGAA
GCGATTTCCCATAGTACCGCTGCAGAAAGCAGGAAGGGATGGCTAATCCACTCCTCGGTG
CTCCCCACCTCCTTCAACTCAGGGACTGCCAGGAACTGTACAGGTACCCACGTGCTCAGC
AAAGACAAGCAGGGCCTCCTGCTGGGCTGCCAGTTCCACCATGAAGCCAGAGTTGTTAGC
GAAGGACCAGATATCCCCCTCATTCCCTGTGTAGAAAAAGATGGGCCCTTCGCCCATCTT
CCAGAACTTATCTGTTGGAAGTAAATGAGTTTCCATAAGGCCAGGGAAACGCAGGTAGGA
ACCCATGCGGTCGAGCCAGCACTCACCTGACACTAGGAACCGCTGGCCAAAGGTTTTGTT
GCCGAAACTCTCAAAGTTGAAATGGTCCATGTATTGCTCAAAATAATTCTCATGAAAGTC
AGGGTCTAGAACTCTGTCGGCTGAGGGCAGGTGCAGAGACTCAGGAGCTGGTTGGGATCA
TCAGGGATCT

>390 Get primers
CGGAAGCGATTTCCCATAGTACCGCTGCAGAAAGCAGGAAGGGATGGCTAATCCACTCCT
CGGTGCTCCCCACCTCCTTCAACTCAGGGACTGCCAGGAACTGTACAGGTACCCACGTGC
TCAGCAAAGACAAGCAGGGCCTCCTGCTGGGCTGCCAGTTCCACCATGAAGCCAGAGTTG
TTAGCGAAGGACCAGATATCCCCCTCATTCCCTGTGTAGAAAAAGATGGGCCCTTCGCCC
ATCTTCCAGAACTTATCTGTTGGAAGTAAATGAGTTTCCATAAGGCCAGGGAAACGCAGG
TAGGAACCCATGCGGTCGAGCCAGCACTCACCTGACACTAGGAACCGCTGGCCAAAGGTT
TTGTTGCCGAAACTCTCAAAGTTGAAATGGTCCATGTATTGCTCAAAATAATTCTCATGA
AAGTCAGGGTCTAGAACTCTGTCGGCTGAGGGCAGGTGCAGAGACTCAGGAGCTGGTTGG
GATCATCAGGGATCTAGGCGGGTCAGGAGGAAGGGCAGCCAGTCTGTACTCACCTCTGGC
CTGGAGGTTG

>391 Get primers
CTCCTCGGTGCTCCCCACCTCCTTCAACTCAGGGACTGCCAGGAACTGTACAGGTACCCA
CGTGCTCAGCAAAGACAAGCAGGGCCTCCTGCTGGGCTGCCAGTTCCACCATGAAGCCAG
AGTTGTTAGCGAAGGACCAGATATCCCCCTCATTCCCTGTGTAGAAAAAGATGGGCCCTT
CGCCCATCTTCCAGAACTTATCTGTTGGAAGTAAATGAGTTTCCATAAGGCCAGGGAAAC
GCAGGTAGGAACCCATGCGGTCGAGCCAGCACTCACCTGACACTAGGAACCGCTGGCCAA
AGGTTTTGTTGCCGAAACTCTCAAAGTTGAAATGGTCCATGTATTGCTCAAAATAATTCT
CATGAAAGTCAGGGTCTAGAACTCTGTCGGCTGAGGGCAGGTGCAGAGACTCAGGAGCTG
GTTGGGATCATCAGGGATCTAGGCGGGTCAGGAGGAAGGGCAGCCAGTCTGTACTCACCT
CTGGCCTGGAGGTTGCACAGTCCCAGTGACAGCAGCAGGACCAGGATCCAGGAGGGGACA
CCATGGTCCA

>392 Get primers
ACCCACGTGCTCAGCAAAGACAAGCAGGGCCTCCTGCTGGGCTGCCAGTTCCACCATGAA
GCCAGAGTTGTTAGCGAAGGACCAGATATCCCCCTCATTCCCTGTGTAGAAAAAGATGGG
CCCTTCGCCCATCTTCCAGAACTTATCTGTTGGAAGTAAATGAGTTTCCATAAGGCCAGG
GAAACGCAGGTAGGAACCCATGCGGTCGAGCCAGCACTCACCTGACACTAGGAACCGCTG
GCCAAAGGTTTTGTTGCCGAAACTCTCAAAGTTGAAATGGTCCATGTATTGCTCAAAATA
ATTCTCATGAAAGTCAGGGTCTAGAACTCTGTCGGCTGAGGGCAGGTGCAGAGACTCAGG
AGCTGGTTGGGATCATCAGGGATCTAGGCGGGTCAGGAGGAAGGGCAGCCAGTCTGTACT
CACCTCTGGCCTGGAGGTTGCACAGTCCCAGTGACAGCAGCAGGACCAGGATCCAGGAGG
GGACACCATGGTCCACAGGGTAACAAGGATGGAAGTTCATGCTTGATTCTGAGCCGGGCG
CTGACTGTCA

>393 Get primers
ATGAAGCCAGAGTTGTTAGCGAAGGACCAGATATCCCCCTCATTCCCTGTGTAGAAAAAG
ATGGGCCCTTCGCCCATCTTCCAGAACTTATCTGTTGGAAGTAAATGAGTTTCCATAAGG
CCAGGGAAACGCAGGTAGGAACCCATGCGGTCGAGCCAGCACTCACCTGACACTAGGAAC
CGCTGGCCAAAGGTTTTGTTGCCGAAACTCTCAAAGTTGAAATGGTCCATGTATTGCTCA
AAATAATTCTCATGAAAGTCAGGGTCTAGAACTCTGTCGGCTGAGGGCAGGTGCAGAGAC
TCAGGAGCTGGTTGGGATCATCAGGGATCTAGGCGGGTCAGGAGGAAGGGCAGCCAGTCT
GTACTCACCTCTGGCCTGGAGGTTGCACAGTCCCAGTGACAGCAGCAGGACCAGGATCCA
GGAGGGGACACCATGGTCCACAGGGTAACAAGGATGGAAGTTCATGCTTGATTCTGAGCC
GGGCGCTGACTGTCATGTGATTTGGTCACATGACCGACACAACGGGCGGGGCAGCATCAC
GTGATAGTCT

>394 Get primers
AAAAGATGGGCCCTTCGCCCATCTTCCAGAACTTATCTGTTGGAAGTAAATGAGTTTCCA
TAAGGCCAGGGAAACGCAGGTAGGAACCCATGCGGTCGAGCCAGCACTCACCTGACACTA
GGAACCGCTGGCCAAAGGTTTTGTTGCCGAAACTCTCAAAGTTGAAATGGTCCATGTATT
GCTCAAAATAATTCTCATGAAAGTCAGGGTCTAGAACTCTGTCGGCTGAGGGCAGGTGCA
GAGACTCAGGAGCTGGTTGGGATCATCAGGGATCTAGGCGGGTCAGGAGGAAGGGCAGCC
AGTCTGTACTCACCTCTGGCCTGGAGGTTGCACAGTCCCAGTGACAGCAGCAGGACCAGG
ATCCAGGAGGGGACACCATGGTCCACAGGGTAACAAGGATGGAAGTTCATGCTTGATTCT
GAGCCGGGCGCTGACTGTCATGTGATTTGGTCACATGACCGACACAACGGGCGGGGCAGC
ATCACGTGATAGTCTGGCGGGGGCTGTCCTACTGTGGCTGGATTCTAGTTGGAGGATCAG
CCTACTCTTC

>395 Get primers
TTCCATAAGGCCAGGGAAACGCAGGTAGGAACCCATGCGGTCGAGCCAGCACTCACCTGA
CACTAGGAACCGCTGGCCAAAGGTTTTGTTGCCGAAACTCTCAAAGTTGAAATGGTCCAT
GTATTGCTCAAAATAATTCTCATGAAAGTCAGGGTCTAGAACTCTGTCGGCTGAGGGCAG
GTGCAGAGACTCAGGAGCTGGTTGGGATCATCAGGGATCTAGGCGGGTCAGGAGGAAGGG
CAGCCAGTCTGTACTCACCTCTGGCCTGGAGGTTGCACAGTCCCAGTGACAGCAGCAGGA
CCAGGATCCAGGAGGGGACACCATGGTCCACAGGGTAACAAGGATGGAAGTTCATGCTTG
ATTCTGAGCCGGGCGCTGACTGTCATGTGATTTGGTCACATGACCGACACAACGGGCGGG
GCAGCATCACGTGATAGTCTGGCGGGGGCTGTCCTACTGTGGCTGGATTCTAGTTGGAGG
ATCAGCCTACTCTTCTTCAGTTTCCCGGTTCCTCCAAATTTCTGGGCTCCTACTTGTTTC
CACAGAGATG

>396 Get primers
CCTGACACTAGGAACCGCTGGCCAAAGGTTTTGTTGCCGAAACTCTCAAAGTTGAAATGG
TCCATGTATTGCTCAAAATAATTCTCATGAAAGTCAGGGTCTAGAACTCTGTCGGCTGAG
GGCAGGTGCAGAGACTCAGGAGCTGGTTGGGATCATCAGGGATCTAGGCGGGTCAGGAGG
AAGGGCAGCCAGTCTGTACTCACCTCTGGCCTGGAGGTTGCACAGTCCCAGTGACAGCAG
CAGGACCAGGATCCAGGAGGGGACACCATGGTCCACAGGGTAACAAGGATGGAAGTTCAT
GCTTGATTCTGAGCCGGGCGCTGACTGTCATGTGATTTGGTCACATGACCGACACAACGG
GCGGGGCAGCATCACGTGATAGTCTGGCGGGGGCTGTCCTACTGTGGCTGGATTCTAGTT
GGAGGATCAGCCTACTCTTCTTCAGTTTCCCGGTTCCTCCAAATTTCTGGGCTCCTACTT
GTTTCCACAGAGATGGATACTGTGGAGGTCCAGGAAGCAGAGAGATGGCTAAGGCTCATC
AGGACCGTAT

>397 Get primers
AATGGTCCATGTATTGCTCAAAATAATTCTCATGAAAGTCAGGGTCTAGAACTCTGTCGG
CTGAGGGCAGGTGCAGAGACTCAGGAGCTGGTTGGGATCATCAGGGATCTAGGCGGGTCA
GGAGGAAGGGCAGCCAGTCTGTACTCACCTCTGGCCTGGAGGTTGCACAGTCCCAGTGAC
AGCAGCAGGACCAGGATCCAGGAGGGGACACCATGGTCCACAGGGTAACAAGGATGGAAG
TTCATGCTTGATTCTGAGCCGGGCGCTGACTGTCATGTGATTTGGTCACATGACCGACAC
AACGGGCGGGGCAGCATCACGTGATAGTCTGGCGGGGGCTGTCCTACTGTGGCTGGATTC
TAGTTGGAGGATCAGCCTACTCTTCTTCAGTTTCCCGGTTCCTCCAAATTTCTGGGCTCC
TACTTGTTTCCACAGAGATGGATACTGTGGAGGTCCAGGAAGCAGAGAGATGGCTAAGGC
TCATCAGGACCGTATGATCTCCCAAGTGTCCAGCTACTGAGTACCACAAGGTGATGGGTG
GGAGGGTCCT

>398 Get primers
GTCGGCTGAGGGCAGGTGCAGAGACTCAGGAGCTGGTTGGGATCATCAGGGATCTAGGCG
GGTCAGGAGGAAGGGCAGCCAGTCTGTACTCACCTCTGGCCTGGAGGTTGCACAGTCCCA
GTGACAGCAGCAGGACCAGGATCCAGGAGGGGACACCATGGTCCACAGGGTAACAAGGAT
GGAAGTTCATGCTTGATTCTGAGCCGGGCGCTGACTGTCATGTGATTTGGTCACATGACC
GACACAACGGGCGGGGCAGCATCACGTGATAGTCTGGCGGGGGCTGTCCTACTGTGGCTG
GATTCTAGTTGGAGGATCAGCCTACTCTTCTTCAGTTTCCCGGTTCCTCCAAATTTCTGG
GCTCCTACTTGTTTCCACAGAGATGGATACTGTGGAGGTCCAGGAAGCAGAGAGATGGCT
AAGGCTCATCAGGACCGTATGATCTCCCAAGTGTCCAGCTACTGAGTACCACAAGGTGAT
GGGTGGGAGGGTCCTCCCACGGAAGGATACCGCAGTCCCTAGGGGTTGCAAGCCCCACAT
GTTCCACTGG

>399 Get primers
AGGCGGGTCAGGAGGAAGGGCAGCCAGTCTGTACTCACCTCTGGCCTGGAGGTTGCACAG
TCCCAGTGACAGCAGCAGGACCAGGATCCAGGAGGGGACACCATGGTCCACAGGGTAACA
AGGATGGAAGTTCATGCTTGATTCTGAGCCGGGCGCTGACTGTCATGTGATTTGGTCACA
TGACCGACACAACGGGCGGGGCAGCATCACGTGATAGTCTGGCGGGGGCTGTCCTACTGT
GGCTGGATTCTAGTTGGAGGATCAGCCTACTCTTCTTCAGTTTCCCGGTTCCTCCAAATT
TCTGGGCTCCTACTTGTTTCCACAGAGATGGATACTGTGGAGGTCCAGGAAGCAGAGAGA
TGGCTAAGGCTCATCAGGACCGTATGATCTCCCAAGTGTCCAGCTACTGAGTACCACAAG
GTGATGGGTGGGAGGGTCCTCCCACGGAAGGATACCGCAGTCCCTAGGGGTTGCAAGCCC
CACATGTTCCACTGGCTGCTAGAGCTACCTACTCAATCAGCCCTGGGCATCACCATCAGG
TACTCGGCCA

>400 Get primers
CACAGTCCCAGTGACAGCAGCAGGACCAGGATCCAGGAGGGGACACCATGGTCCACAGGG
TAACAAGGATGGAAGTTCATGCTTGATTCTGAGCCGGGCGCTGACTGTCATGTGATTTGG
TCACATGACCGACACAACGGGCGGGGCAGCATCACGTGATAGTCTGGCGGGGGCTGTCCT
ACTGTGGCTGGATTCTAGTTGGAGGATCAGCCTACTCTTCTTCAGTTTCCCGGTTCCTCC
AAATTTCTGGGCTCCTACTTGTTTCCACAGAGATGGATACTGTGGAGGTCCAGGAAGCAG
AGAGATGGCTAAGGCTCATCAGGACCGTATGATCTCCCAAGTGTCCAGCTACTGAGTACC
ACAAGGTGATGGGTGGGAGGGTCCTCCCACGGAAGGATACCGCAGTCCCTAGGGGTTGCA
AGCCCCACATGTTCCACTGGCTGCTAGAGCTACCTACTCAATCAGCCCTGGGCATCACCA
TCAGGTACTCGGCCAAAATGACCTCTCTGCTTCCAGTCCTCAGTTCTGGTCAGCACCAGA
CAGGCCCATA

>401 Get primers
CAGGGTAACAAGGATGGAAGTTCATGCTTGATTCTGAGCCGGGCGCTGACTGTCATGTGA
TTTGGTCACATGACCGACACAACGGGCGGGGCAGCATCACGTGATAGTCTGGCGGGGGCT
GTCCTACTGTGGCTGGATTCTAGTTGGAGGATCAGCCTACTCTTCTTCAGTTTCCCGGTT
CCTCCAAATTTCTGGGCTCCTACTTGTTTCCACAGAGATGGATACTGTGGAGGTCCAGGA
AGCAGAGAGATGGCTAAGGCTCATCAGGACCGTATGATCTCCCAAGTGTCCAGCTACTGA
GTACCACAAGGTGATGGGTGGGAGGGTCCTCCCACGGAAGGATACCGCAGTCCCTAGGGG
TTGCAAGCCCCACATGTTCCACTGGCTGCTAGAGCTACCTACTCAATCAGCCCTGGGCAT
CACCATCAGGTACTCGGCCAAAATGACCTCTCTGCTTCCAGTCCTCAGTTCTGGTCAGCA
CCAGACAGGCCCATAATTACAGAGCCAGGGAAACTGGAACATTTGTCTCCCCTTAGACAG
TGGCAGCAGG

>402 Get primers
TGTGATTTGGTCACATGACCGACACAACGGGCGGGGCAGCATCACGTGATAGTCTGGCGG
GGGCTGTCCTACTGTGGCTGGATTCTAGTTGGAGGATCAGCCTACTCTTCTTCAGTTTCC
CGGTTCCTCCAAATTTCTGGGCTCCTACTTGTTTCCACAGAGATGGATACTGTGGAGGTC
CAGGAAGCAGAGAGATGGCTAAGGCTCATCAGGACCGTATGATCTCCCAAGTGTCCAGCT
ACTGAGTACCACAAGGTGATGGGTGGGAGGGTCCTCCCACGGAAGGATACCGCAGTCCCT
AGGGGTTGCAAGCCCCACATGTTCCACTGGCTGCTAGAGCTACCTACTCAATCAGCCCTG
GGCATCACCATCAGGTACTCGGCCAAAATGACCTCTCTGCTTCCAGTCCTCAGTTCTGGT
CAGCACCAGACAGGCCCATAATTACAGAGCCAGGGAAACTGGAACATTTGTCTCCCCTTA
GACAGTGGCAGCAGGAAGGTGGGGGGTTGTTGCAGAGGAACAGTGTCTCTGAGAGAGGAC
CTTGGACTTT

>403 Get primers
GGCGGGGGCTGTCCTACTGTGGCTGGATTCTAGTTGGAGGATCAGCCTACTCTTCTTCAG
TTTCCCGGTTCCTCCAAATTTCTGGGCTCCTACTTGTTTCCACAGAGATGGATACTGTGG
AGGTCCAGGAAGCAGAGAGATGGCTAAGGCTCATCAGGACCGTATGATCTCCCAAGTGTC
CAGCTACTGAGTACCACAAGGTGATGGGTGGGAGGGTCCTCCCACGGAAGGATACCGCAG
TCCCTAGGGGTTGCAAGCCCCACATGTTCCACTGGCTGCTAGAGCTACCTACTCAATCAG
CCCTGGGCATCACCATCAGGTACTCGGCCAAAATGACCTCTCTGCTTCCAGTCCTCAGTT
CTGGTCAGCACCAGACAGGCCCATAATTACAGAGCCAGGGAAACTGGAACATTTGTCTCC
CCTTAGACAGTGGCAGCAGGAAGGTGGGGGGTTGTTGCAGAGGAACAGTGTCTCTGAGAG
AGGACCTTGGACTTTCTGGGAATCTCTGAGCTGCCCGGTTCTCCCCACTGCTGGCACTGT
GCCCACAGCC

>404 Get primers
TTCAGTTTCCCGGTTCCTCCAAATTTCTGGGCTCCTACTTGTTTCCACAGAGATGGATAC
TGTGGAGGTCCAGGAAGCAGAGAGATGGCTAAGGCTCATCAGGACCGTATGATCTCCCAA
GTGTCCAGCTACTGAGTACCACAAGGTGATGGGTGGGAGGGTCCTCCCACGGAAGGATAC
CGCAGTCCCTAGGGGTTGCAAGCCCCACATGTTCCACTGGCTGCTAGAGCTACCTACTCA
ATCAGCCCTGGGCATCACCATCAGGTACTCGGCCAAAATGACCTCTCTGCTTCCAGTCCT
CAGTTCTGGTCAGCACCAGACAGGCCCATAATTACAGAGCCAGGGAAACTGGAACATTTG
TCTCCCCTTAGACAGTGGCAGCAGGAAGGTGGGGGGTTGTTGCAGAGGAACAGTGTCTCT
GAGAGAGGACCTTGGACTTTCTGGGAATCTCTGAGCTGCCCGGTTCTCCCCACTGCTGGC
ACTGTGCCCACAGCCCAAACAGAATGGGGGAGATGGAGGGGCAGGGCTTCTGTGGGAAGC
TGCCCTCCAC

>405 Get primers
GATACTGTGGAGGTCCAGGAAGCAGAGAGATGGCTAAGGCTCATCAGGACCGTATGATCT
CCCAAGTGTCCAGCTACTGAGTACCACAAGGTGATGGGTGGGAGGGTCCTCCCACGGAAG
GATACCGCAGTCCCTAGGGGTTGCAAGCCCCACATGTTCCACTGGCTGCTAGAGCTACCT
ACTCAATCAGCCCTGGGCATCACCATCAGGTACTCGGCCAAAATGACCTCTCTGCTTCCA
GTCCTCAGTTCTGGTCAGCACCAGACAGGCCCATAATTACAGAGCCAGGGAAACTGGAAC
ATTTGTCTCCCCTTAGACAGTGGCAGCAGGAAGGTGGGGGGTTGTTGCAGAGGAACAGTG
TCTCTGAGAGAGGACCTTGGACTTTCTGGGAATCTCTGAGCTGCCCGGTTCTCCCCACTG
CTGGCACTGTGCCCACAGCCCAAACAGAATGGGGGAGATGGAGGGGCAGGGCTTCTGTGG
GAAGCTGCCCTCCACCTCATTGGCACAGAGTGTCTCATTGCAGAGAGAAAAAAGGACCAG
TTTTCTCTCT

>406 Get primers
GATCTCCCAAGTGTCCAGCTACTGAGTACCACAAGGTGATGGGTGGGAGGGTCCTCCCAC
GGAAGGATACCGCAGTCCCTAGGGGTTGCAAGCCCCACATGTTCCACTGGCTGCTAGAGC
TACCTACTCAATCAGCCCTGGGCATCACCATCAGGTACTCGGCCAAAATGACCTCTCTGC
TTCCAGTCCTCAGTTCTGGTCAGCACCAGACAGGCCCATAATTACAGAGCCAGGGAAACT
GGAACATTTGTCTCCCCTTAGACAGTGGCAGCAGGAAGGTGGGGGGTTGTTGCAGAGGAA
CAGTGTCTCTGAGAGAGGACCTTGGACTTTCTGGGAATCTCTGAGCTGCCCGGTTCTCCC
CACTGCTGGCACTGTGCCCACAGCCCAAACAGAATGGGGGAGATGGAGGGGCAGGGCTTC
TGTGGGAAGCTGCCCTCCACCTCATTGGCACAGAGTGTCTCATTGCAGAGAGAAAAAAGG
ACCAGTTTTCTCTCTGGCACCCAGGTCTGGAAGAGGAGTGACATCCACGGAAGTTGGTGA
CTTGGACTGG

>407 Get primers
CCCACGGAAGGATACCGCAGTCCCTAGGGGTTGCAAGCCCCACATGTTCCACTGGCTGCT
AGAGCTACCTACTCAATCAGCCCTGGGCATCACCATCAGGTACTCGGCCAAAATGACCTC
TCTGCTTCCAGTCCTCAGTTCTGGTCAGCACCAGACAGGCCCATAATTACAGAGCCAGGG
AAACTGGAACATTTGTCTCCCCTTAGACAGTGGCAGCAGGAAGGTGGGGGGTTGTTGCAG
AGGAACAGTGTCTCTGAGAGAGGACCTTGGACTTTCTGGGAATCTCTGAGCTGCCCGGTT
CTCCCCACTGCTGGCACTGTGCCCACAGCCCAAACAGAATGGGGGAGATGGAGGGGCAGG
GCTTCTGTGGGAAGCTGCCCTCCACCTCATTGGCACAGAGTGTCTCATTGCAGAGAGAAA
AAAGGACCAGTTTTCTCTCTGGCACCCAGGTCTGGAAGAGGAGTGACATCCACGGAAGTT
GGTGACTTGGACTGGCTGGCCGTGAGTGGAACATGTCCATCCAGCATGGCCACAGTCCAG
TGGGACACAC

>408 Get primers
AGGCCAAGAAGTAGACCCACCTGCCAGTGCCCGTAGACCCATGATCCTCTGGCCCTCATT
CAACAGCCGTTGACAGCCCACCTAGAGAAAGGCAGCAGAATATCTCAGTGGAGGCCCCTT
TCACAGAGCGTGGGTCAGGGCTGCTAGCTTCCAGGACACAACAGCAGATAGTGTCTGATG
GCATGAAAGCAGATAGCTACAAGGCTCTTGGACCAGGCTAGCACTGGGTCCTGCACCCAG
GGAGAGCCACCTCACCTTGACAGGGTTGGCAGGAAGGGGCCCTAGAAAGTCAGTAGGATA
CGGGTAGTCCATCATGGCGAGCACAGTAAATGCATTTCGGGCAAACCCAAAGAGCTGAGT
CAGGTCCTTTGGGCTGGAAAGTGATTGACAGGTACCAAAGTTCTGGCTGATGGTGTCATA
GGCTGGGAAGAGAGAGGCCAGGAGAAAAGGCTGAGGAAACTGCTGGCAAATGTGAAGGGC
AAGAATGAATGCCCAAGGTG

>409 Get primers
GGCCCTCATTCAACAGCCGTTGACAGCCCACCTAGAGAAAGGCAGCAGAATATCTCAGTG
GAGGCCCCTTTCACAGAGCGTGGGTCAGGGCTGCTAGCTTCCAGGACACAACAGCAGATA
GTGTCTGATGGCATGAAAGCAGATAGCTACAAGGCTCTTGGACCAGGCTAGCACTGGGTC
CTGCACCCAGGGAGAGCCACCTCACCTTGACAGGGTTGGCAGGAAGGGGCCCTAGAAAGT
CAGTAGGATACGGGTAGTCCATCATGGCGAGCACAGTAAATGCATTTCGGGCAAACCCAA
AGAGCTGAGTCAGGTCCTTTGGGCTGGAAAGTGATTGACAGGTACCAAAGTTCTGGCTGA
TGGTGTCATAGGCTGGGAAGAGAGAGGCCAGGAGAAAAGGCTGAGGAAACTGCTGGCAAA
TGTGAAGGGCAAGAATGAATGCCCAAGGTGGGCAGCAGGTGAGGAAAGAGTCCCTCACCT
CCCTGGAGGAACAAGTCTTT

>410 Get primers
TATCTCAGTGGAGGCCCCTTTCACAGAGCGTGGGTCAGGGCTGCTAGCTTCCAGGACACA
ACAGCAGATAGTGTCTGATGGCATGAAAGCAGATAGCTACAAGGCTCTTGGACCAGGCTA
GCACTGGGTCCTGCACCCAGGGAGAGCCACCTCACCTTGACAGGGTTGGCAGGAAGGGGC
CCTAGAAAGTCAGTAGGATACGGGTAGTCCATCATGGCGAGCACAGTAAATGCATTTCGG
GCAAACCCAAAGAGCTGAGTCAGGTCCTTTGGGCTGGAAAGTGATTGACAGGTACCAAAG
TTCTGGCTGATGGTGTCATAGGCTGGGAAGAGAGAGGCCAGGAGAAAAGGCTGAGGAAAC
TGCTGGCAAATGTGAAGGGCAAGAATGAATGCCCAAGGTGGGCAGCAGGTGAGGAAAGAG
TCCCTCACCTCCCTGGAGGAACAAGTCTTTGATTTGCTGAAAGGCATCCCGCACAGCCTG
GGCGCACTTGGGACTCTGGC

>411 Get primers
CCAGGACACAACAGCAGATAGTGTCTGATGGCATGAAAGCAGATAGCTACAAGGCTCTTG
GACCAGGCTAGCACTGGGTCCTGCACCCAGGGAGAGCCACCTCACCTTGACAGGGTTGGC
AGGAAGGGGCCCTAGAAAGTCAGTAGGATACGGGTAGTCCATCATGGCGAGCACAGTAAA
TGCATTTCGGGCAAACCCAAAGAGCTGAGTCAGGTCCTTTGGGCTGGAAAGTGATTGACA
GGTACCAAAGTTCTGGCTGATGGTGTCATAGGCTGGGAAGAGAGAGGCCAGGAGAAAAGG
CTGAGGAAACTGCTGGCAAATGTGAAGGGCAAGAATGAATGCCCAAGGTGGGCAGCAGGT
GAGGAAAGAGTCCCTCACCTCCCTGGAGGAACAAGTCTTTGATTTGCTGAAAGGCATCCC
GCACAGCCTGGGCGCACTTGGGACTCTGGCCATAAAAGTCCTGGAGAAGAGACCAAGGTT
GCTGCTGCCATTCTTGCACT

>412 Get primers
AAGGCTCTTGGACCAGGCTAGCACTGGGTCCTGCACCCAGGGAGAGCCACCTCACCTTGA
CAGGGTTGGCAGGAAGGGGCCCTAGAAAGTCAGTAGGATACGGGTAGTCCATCATGGCGA
GCACAGTAAATGCATTTCGGGCAAACCCAAAGAGCTGAGTCAGGTCCTTTGGGCTGGAAA
GTGATTGACAGGTACCAAAGTTCTGGCTGATGGTGTCATAGGCTGGGAAGAGAGAGGCCA
GGAGAAAAGGCTGAGGAAACTGCTGGCAAATGTGAAGGGCAAGAATGAATGCCCAAGGTG
GGCAGCAGGTGAGGAAAGAGTCCCTCACCTCCCTGGAGGAACAAGTCTTTGATTTGCTGA
AAGGCATCCCGCACAGCCTGGGCGCACTTGGGACTCTGGCCATAAAAGTCCTGGAGAAGA
GACCAAGGTTGCTGCTGCCATTCTTGCACTGGCCTGGGGTACCCAAGTCCCCTCACTCAC
CGCTGTGACATCTCGGAAGA

>413 Get primers
CTCACCTTGACAGGGTTGGCAGGAAGGGGCCCTAGAAAGTCAGTAGGATACGGGTAGTCC
ATCATGGCGAGCACAGTAAATGCATTTCGGGCAAACCCAAAGAGCTGAGTCAGGTCCTTT
GGGCTGGAAAGTGATTGACAGGTACCAAAGTTCTGGCTGATGGTGTCATAGGCTGGGAAG
AGAGAGGCCAGGAGAAAAGGCTGAGGAAACTGCTGGCAAATGTGAAGGGCAAGAATGAAT
GCCCAAGGTGGGCAGCAGGTGAGGAAAGAGTCCCTCACCTCCCTGGAGGAACAAGTCTTT
GATTTGCTGAAAGGCATCCCGCACAGCCTGGGCGCACTTGGGACTCTGGCCATAAAAGTC
CTGGAGAAGAGACCAAGGTTGCTGCTGCCATTCTTGCACTGGCCTGGGGTACCCAAGTCC
CCTCACTCACCGCTGTGACATCTCGGAAGAATTGGTAGGAGTCCCCAAGGCCTGCAACAG
CTACAACAGGAGCGCTGGCT

>414 Get primers
CGGGTAGTCCATCATGGCGAGCACAGTAAATGCATTTCGGGCAAACCCAAAGAGCTGAGT
CAGGTCCTTTGGGCTGGAAAGTGATTGACAGGTACCAAAGTTCTGGCTGATGGTGTCATA
GGCTGGGAAGAGAGAGGCCAGGAGAAAAGGCTGAGGAAACTGCTGGCAAATGTGAAGGGC
AAGAATGAATGCCCAAGGTGGGCAGCAGGTGAGGAAAGAGTCCCTCACCTCCCTGGAGGA
ACAAGTCTTTGATTTGCTGAAAGGCATCCCGCACAGCCTGGGCGCACTTGGGACTCTGGC
CATAAAAGTCCTGGAGAAGAGACCAAGGTTGCTGCTGCCATTCTTGCACTGGCCTGGGGT
ACCCAAGTCCCCTCACTCACCGCTGTGACATCTCGGAAGAATTGGTAGGAGTCCCCAAGG
CCTGCAACAGCTACAACAGGAGCGCTGGCTGCCAGTGCCCCAGCCACCAGGTGGGGGTAC
TTCATCCTCATGTAGGCACT

>415 Get primers
AGAGCTGAGTCAGGTCCTTTGGGCTGGAAAGTGATTGACAGGTACCAAAGTTCTGGCTGA
TGGTGTCATAGGCTGGGAAGAGAGAGGCCAGGAGAAAAGGCTGAGGAAACTGCTGGCAAA
TGTGAAGGGCAAGAATGAATGCCCAAGGTGGGCAGCAGGTGAGGAAAGAGTCCCTCACCT
CCCTGGAGGAACAAGTCTTTGATTTGCTGAAAGGCATCCCGCACAGCCTGGGCGCACTTG
GGACTCTGGCCATAAAAGTCCTGGAGAAGAGACCAAGGTTGCTGCTGCCATTCTTGCACT
GGCCTGGGGTACCCAAGTCCCCTCACTCACCGCTGTGACATCTCGGAAGAATTGGTAGGA
GTCCCCAAGGCCTGCAACAGCTACAACAGGAGCGCTGGCTGCCAGTGCCCCAGCCACCAG
GTGGGGGTACTTCATCCTCATGTAGGCACTCAGCATCCCCCCATAACTGGGAGTACAGAG
CACAGATCATGGTTGTGGGA

>416 Get primers
TTCTGGCTGATGGTGTCATAGGCTGGGAAGAGAGAGGCCAGGAGAAAAGGCTGAGGAAAC
TGCTGGCAAATGTGAAGGGCAAGAATGAATGCCCAAGGTGGGCAGCAGGTGAGGAAAGAG
TCCCTCACCTCCCTGGAGGAACAAGTCTTTGATTTGCTGAAAGGCATCCCGCACAGCCTG
GGCGCACTTGGGACTCTGGCCATAAAAGTCCTGGAGAAGAGACCAAGGTTGCTGCTGCCA
TTCTTGCACTGGCCTGGGGTACCCAAGTCCCCTCACTCACCGCTGTGACATCTCGGAAGA
ATTGGTAGGAGTCCCCAAGGCCTGCAACAGCTACAACAGGAGCGCTGGCTGCCAGTGCCC
CAGCCACCAGGTGGGGGTACTTCATCCTCATGTAGGCACTCAGCATCCCCCCATAACTGG
GAGTACAGAGCACAGATCATGGTTGTGGGAAGCTGCCCACAACTCAGGCGAGCAGCCTCA
CTGTCCTCCAGGCTGAGGTG

>417 Get primers
CTGAGGAAACTGCTGGCAAATGTGAAGGGCAAGAATGAATGCCCAAGGTGGGCAGCAGGT
GAGGAAAGAGTCCCTCACCTCCCTGGAGGAACAAGTCTTTGATTTGCTGAAAGGCATCCC
GCACAGCCTGGGCGCACTTGGGACTCTGGCCATAAAAGTCCTGGAGAAGAGACCAAGGTT
GCTGCTGCCATTCTTGCACTGGCCTGGGGTACCCAAGTCCCCTCACTCACCGCTGTGACA
TCTCGGAAGAATTGGTAGGAGTCCCCAAGGCCTGCAACAGCTACAACAGGAGCGCTGGCT
GCCAGTGCCCCAGCCACCAGGTGGGGGTACTTCATCCTCATGTAGGCACTCAGCATCCCC
CCATAACTGGGAGTACAGAGCACAGATCATGGTTGTGGGAAGCTGCCCACAACTCAGGCG
AGCAGCCTCACTGTCCTCCAGGCTGAGGTGCTAGGCTGCTCTTTCCCTGCTCAGAACGCC
CAAGGGTGGGAAAGAAGGAC

>418 Get primers
GGCAGCAGGTGAGGAAAGAGTCCCTCACCTCCCTGGAGGAACAAGTCTTTGATTTGCTGA
AAGGCATCCCGCACAGCCTGGGCGCACTTGGGACTCTGGCCATAAAAGTCCTGGAGAAGA
GACCAAGGTTGCTGCTGCCATTCTTGCACTGGCCTGGGGTACCCAAGTCCCCTCACTCAC
CGCTGTGACATCTCGGAAGAATTGGTAGGAGTCCCCAAGGCCTGCAACAGCTACAACAGG
AGCGCTGGCTGCCAGTGCCCCAGCCACCAGGTGGGGGTACTTCATCCTCATGTAGGCACT
CAGCATCCCCCCATAACTGGGAGTACAGAGCACAGATCATGGTTGTGGGAAGCTGCCCAC
AACTCAGGCGAGCAGCCTCACTGTCCTCCAGGCTGAGGTGCTAGGCTGCTCTTTCCCTGC
TCAGAACGCCCAAGGGTGGGAAAGAAGGACCTGAAACTGTCAGGCCCACACACCCTGATC
CCAGGGCCAAGGCAGATACA

>419 Get primers
GATTTGCTGAAAGGCATCCCGCACAGCCTGGGCGCACTTGGGACTCTGGCCATAAAAGTC
CTGGAGAAGAGACCAAGGTTGCTGCTGCCATTCTTGCACTGGCCTGGGGTACCCAAGTCC
CCTCACTCACCGCTGTGACATCTCGGAAGAATTGGTAGGAGTCCCCAAGGCCTGCAACAG
CTACAACAGGAGCGCTGGCTGCCAGTGCCCCAGCCACCAGGTGGGGGTACTTCATCCTCA
TGTAGGCACTCAGCATCCCCCCATAACTGGGAGTACAGAGCACAGATCATGGTTGTGGGA
AGCTGCCCACAACTCAGGCGAGCAGCCTCACTGTCCTCCAGGCTGAGGTGCTAGGCTGCT
CTTTCCCTGCTCAGAACGCCCAAGGGTGGGAAAGAAGGACCTGAAACTGTCAGGCCCACA
CACCCTGATCCCAGGGCCAAGGCAGATACAGCCTTCACTGGGAGAAGGCACCTGTGGGTG
CCCTGCCCTGACCCAGCAAT

>420 Get primers
CATAAAAGTCCTGGAGAAGAGACCAAGGTTGCTGCTGCCATTCTTGCACTGGCCTGGGGT
ACCCAAGTCCCCTCACTCACCGCTGTGACATCTCGGAAGAATTGGTAGGAGTCCCCAAGG
CCTGCAACAGCTACAACAGGAGCGCTGGCTGCCAGTGCCCCAGCCACCAGGTGGGGGTAC
TTCATCCTCATGTAGGCACTCAGCATCCCCCCATAACTGGGAGTACAGAGCACAGATCAT
GGTTGTGGGAAGCTGCCCACAACTCAGGCGAGCAGCCTCACTGTCCTCCAGGCTGAGGTG
CTAGGCTGCTCTTTCCCTGCTCAGAACGCCCAAGGGTGGGAAAGAAGGACCTGAAACTGT
CAGGCCCACACACCCTGATCCCAGGGCCAAGGCAGATACAGCCTTCACTGGGAGAAGGCA
CCTGTGGGTGCCCTGCCCTGACCCAGCAATGAAGACATTGCAGAGACAAAGTCAGAAGGA
ATTGTCCCACTAGTGGGAAC

>421 Get primers
GGCCTGGGGTACCCAAGTCCCCTCACTCACCGCTGTGACATCTCGGAAGAATTGGTAGGA
GTCCCCAAGGCCTGCAACAGCTACAACAGGAGCGCTGGCTGCCAGTGCCCCAGCCACCAG
GTGGGGGTACTTCATCCTCATGTAGGCACTCAGCATCCCCCCATAACTGGGAGTACAGAG
CACAGATCATGGTTGTGGGAAGCTGCCCACAACTCAGGCGAGCAGCCTCACTGTCCTCCA
GGCTGAGGTGCTAGGCTGCTCTTTCCCTGCTCAGAACGCCCAAGGGTGGGAAAGAAGGAC
CTGAAACTGTCAGGCCCACACACCCTGATCCCAGGGCCAAGGCAGATACAGCCTTCACTG
GGAGAAGGCACCTGTGGGTGCCCTGCCCTGACCCAGCAATGAAGACATTGCAGAGACAAA
GTCAGAAGGAATTGTCCCACTAGTGGGAACAACATAGCATACACTGCCTATGAGGTCCAC
TCAAGGAGGGCTTCCAGAAG

>422 Get primers
ATTGGTAGGAGTCCCCAAGGCCTGCAACAGCTACAACAGGAGCGCTGGCTGCCAGTGCCC
CAGCCACCAGGTGGGGGTACTTCATCCTCATGTAGGCACTCAGCATCCCCCCATAACTGG
GAGTACAGAGCACAGATCATGGTTGTGGGAAGCTGCCCACAACTCAGGCGAGCAGCCTCA
CTGTCCTCCAGGCTGAGGTGCTAGGCTGCTCTTTCCCTGCTCAGAACGCCCAAGGGTGGG
AAAGAAGGACCTGAAACTGTCAGGCCCACACACCCTGATCCCAGGGCCAAGGCAGATACA
GCCTTCACTGGGAGAAGGCACCTGTGGGTGCCCTGCCCTGACCCAGCAATGAAGACATTG
CAGAGACAAAGTCAGAAGGAATTGTCCCACTAGTGGGAACAACATAGCATACACTGCCTA
TGAGGTCCACTCAAGGAGGGCTTCCAGAAGGAGGTAAAGCTAGACCCCGCCCTTCCACAT
GTGGGGTAGGCATAGGATGT

>423 Get primers
GCCAGTGCCCCAGCCACCAGGTGGGGGTACTTCATCCTCATGTAGGCACTCAGCATCCCC
CCATAACTGGGAGTACAGAGCACAGATCATGGTTGTGGGAAGCTGCCCACAACTCAGGCG
AGCAGCCTCACTGTCCTCCAGGCTGAGGTGCTAGGCTGCTCTTTCCCTGCTCAGAACGCC
CAAGGGTGGGAAAGAAGGACCTGAAACTGTCAGGCCCACACACCCTGATCCCAGGGCCAA
GGCAGATACAGCCTTCACTGGGAGAAGGCACCTGTGGGTGCCCTGCCCTGACCCAGCAAT
GAAGACATTGCAGAGACAAAGTCAGAAGGAATTGTCCCACTAGTGGGAACAACATAGCAT
ACACTGCCTATGAGGTCCACTCAAGGAGGGCTTCCAGAAGGAGGTAAAGCTAGACCCCGC
CCTTCCACATGTGGGGTAGGCATAGGATGTTGAGACTGTAAGAGACATCTCTTTGGCCCT
CCTTGTATAGGGTGTCAATC

>424 Get primers
CAGCATCCCCCCATAACTGGGAGTACAGAGCACAGATCATGGTTGTGGGAAGCTGCCCAC
AACTCAGGCGAGCAGCCTCACTGTCCTCCAGGCTGAGGTGCTAGGCTGCTCTTTCCCTGC
TCAGAACGCCCAAGGGTGGGAAAGAAGGACCTGAAACTGTCAGGCCCACACACCCTGATC
CCAGGGCCAAGGCAGATACAGCCTTCACTGGGAGAAGGCACCTGTGGGTGCCCTGCCCTG
ACCCAGCAATGAAGACATTGCAGAGACAAAGTCAGAAGGAATTGTCCCACTAGTGGGAAC
AACATAGCATACACTGCCTATGAGGTCCACTCAAGGAGGGCTTCCAGAAGGAGGTAAAGC
TAGACCCCGCCCTTCCACATGTGGGGTAGGCATAGGATGTTGAGACTGTAAGAGACATCT
CTTTGGCCCTCCTTGTATAGGGTGTCAATCGGCACAACAGGGTGGAGCCTTAGAGTAGGG
TAAGATTAGGACTCTAGGTT

>425 Get primers
AGCTGCCCACAACTCAGGCGAGCAGCCTCACTGTCCTCCAGGCTGAGGTGCTAGGCTGCT
CTTTCCCTGCTCAGAACGCCCAAGGGTGGGAAAGAAGGACCTGAAACTGTCAGGCCCACA
CACCCTGATCCCAGGGCCAAGGCAGATACAGCCTTCACTGGGAGAAGGCACCTGTGGGTG
CCCTGCCCTGACCCAGCAATGAAGACATTGCAGAGACAAAGTCAGAAGGAATTGTCCCAC
TAGTGGGAACAACATAGCATACACTGCCTATGAGGTCCACTCAAGGAGGGCTTCCAGAAG
GAGGTAAAGCTAGACCCCGCCCTTCCACATGTGGGGTAGGCATAGGATGTTGAGACTGTA
AGAGACATCTCTTTGGCCCTCCTTGTATAGGGTGTCAATCGGCACAACAGGGTGGAGCCT
TAGAGTAGGGTAAGATTAGGACTCTAGGTTCTCTCATGGGTCCAGATCTGTCATGAAGGG
AGGTCAAGGACCCACCTCCC

>426 Get primers
CTAGGCTGCTCTTTCCCTGCTCAGAACGCCCAAGGGTGGGAAAGAAGGACCTGAAACTGT
CAGGCCCACACACCCTGATCCCAGGGCCAAGGCAGATACAGCCTTCACTGGGAGAAGGCA
CCTGTGGGTGCCCTGCCCTGACCCAGCAATGAAGACATTGCAGAGACAAAGTCAGAAGGA
ATTGTCCCACTAGTGGGAACAACATAGCATACACTGCCTATGAGGTCCACTCAAGGAGGG
CTTCCAGAAGGAGGTAAAGCTAGACCCCGCCCTTCCACATGTGGGGTAGGCATAGGATGT
TGAGACTGTAAGAGACATCTCTTTGGCCCTCCTTGTATAGGGTGTCAATCGGCACAACAG
GGTGGAGCCTTAGAGTAGGGTAAGATTAGGACTCTAGGTTCTCTCATGGGTCCAGATCTG
TCATGAAGGGAGGTCAAGGACCCACCTCCCTCCAAAGGCTATGGTGGGGGCATCATGGAC
CCCAAGGTCCTGCCGCAGGG

>436 Get primers
CCTGGAGCAGCACAGCAAAGTCGGCCAGCGCCTGCTCCACAGTCAGCAGCTGTGTATATC
CCCGCTGTGTGGACTGGACACCGAACGGAAGCGATTTCCCATAGTACCGCTGCAGAAAGC
AGGAAGGGATGGCTAATCCACTCCTCGGTGCTCCCCACCTCCTTCAACTCAGGGACTGCC
AGGAACTGTACAGGTACCCACGTGCTCAGCAAAGACAAGCAGGGCCTCCTGCTGGGCTGC
CAGTTCCACCATGAAGCCAGAGTTGTTAGCGAAGGACCAGATATCCCCCTCATTCCCTGT
GTAGAAAAAGATGGGCCCTTCGCCCATCTTCCAGAACTTATCTGTTGGAAGTAAATGAGT
TTCCATAAGGCCAGGGAAACGCAGGTAGGAACCCATGCGGTCGAGCCAGCACTCACCTGA
CACTAGGAACCGCTGGCCAAAGGTTTTGTTGCCGAAACTCTCAAAGTTGAAATGGTCCAT
GTATTGCTCAAAATAATTCT

>437 Get primers
TGTGTATATCCCCGCTGTGTGGACTGGACACCGAACGGAAGCGATTTCCCATAGTACCGC
TGCAGAAAGCAGGAAGGGATGGCTAATCCACTCCTCGGTGCTCCCCACCTCCTTCAACTC
AGGGACTGCCAGGAACTGTACAGGTACCCACGTGCTCAGCAAAGACAAGCAGGGCCTCCT
GCTGGGCTGCCAGTTCCACCATGAAGCCAGAGTTGTTAGCGAAGGACCAGATATCCCCCT
CATTCCCTGTGTAGAAAAAGATGGGCCCTTCGCCCATCTTCCAGAACTTATCTGTTGGAA
GTAAATGAGTTTCCATAAGGCCAGGGAAACGCAGGTAGGAACCCATGCGGTCGAGCCAGC
ACTCACCTGACACTAGGAACCGCTGGCCAAAGGTTTTGTTGCCGAAACTCTCAAAGTTGA
AATGGTCCATGTATTGCTCAAAATAATTCTCATGAAAGTCAGGGTCTAGAACTCTGTCGG
CTGAGGGCAGGTGCAGAGAC

>438 Get primers
ATAGTACCGCTGCAGAAAGCAGGAAGGGATGGCTAATCCACTCCTCGGTGCTCCCCACCT
CCTTCAACTCAGGGACTGCCAGGAACTGTACAGGTACCCACGTGCTCAGCAAAGACAAGC
AGGGCCTCCTGCTGGGCTGCCAGTTCCACCATGAAGCCAGAGTTGTTAGCGAAGGACCAG
ATATCCCCCTCATTCCCTGTGTAGAAAAAGATGGGCCCTTCGCCCATCTTCCAGAACTTA
TCTGTTGGAAGTAAATGAGTTTCCATAAGGCCAGGGAAACGCAGGTAGGAACCCATGCGG
TCGAGCCAGCACTCACCTGACACTAGGAACCGCTGGCCAAAGGTTTTGTTGCCGAAACTC
TCAAAGTTGAAATGGTCCATGTATTGCTCAAAATAATTCTCATGAAAGTCAGGGTCTAGA
ACTCTGTCGGCTGAGGGCAGGTGCAGAGACTCAGGAGCTGGTTGGGATCATCAGGGATCT
AGGCGGGTCAGGAGGAAGGG

>439 Get primers
CTCCCCACCTCCTTCAACTCAGGGACTGCCAGGAACTGTACAGGTACCCACGTGCTCAGC
AAAGACAAGCAGGGCCTCCTGCTGGGCTGCCAGTTCCACCATGAAGCCAGAGTTGTTAGC
GAAGGACCAGATATCCCCCTCATTCCCTGTGTAGAAAAAGATGGGCCCTTCGCCCATCTT
CCAGAACTTATCTGTTGGAAGTAAATGAGTTTCCATAAGGCCAGGGAAACGCAGGTAGGA
ACCCATGCGGTCGAGCCAGCACTCACCTGACACTAGGAACCGCTGGCCAAAGGTTTTGTT
GCCGAAACTCTCAAAGTTGAAATGGTCCATGTATTGCTCAAAATAATTCTCATGAAAGTC
AGGGTCTAGAACTCTGTCGGCTGAGGGCAGGTGCAGAGACTCAGGAGCTGGTTGGGATCA
TCAGGGATCTAGGCGGGTCAGGAGGAAGGGCAGCCAGTCTGTACTCACCTCTGGCCTGGA
GGTTGCACAGTCCCAGTGAC

>440 Get primers
CGTGCTCAGCAAAGACAAGCAGGGCCTCCTGCTGGGCTGCCAGTTCCACCATGAAGCCAG
AGTTGTTAGCGAAGGACCAGATATCCCCCTCATTCCCTGTGTAGAAAAAGATGGGCCCTT
CGCCCATCTTCCAGAACTTATCTGTTGGAAGTAAATGAGTTTCCATAAGGCCAGGGAAAC
GCAGGTAGGAACCCATGCGGTCGAGCCAGCACTCACCTGACACTAGGAACCGCTGGCCAA
AGGTTTTGTTGCCGAAACTCTCAAAGTTGAAATGGTCCATGTATTGCTCAAAATAATTCT
CATGAAAGTCAGGGTCTAGAACTCTGTCGGCTGAGGGCAGGTGCAGAGACTCAGGAGCTG
GTTGGGATCATCAGGGATCTAGGCGGGTCAGGAGGAAGGGCAGCCAGTCTGTACTCACCT
CTGGCCTGGAGGTTGCACAGTCCCAGTGACAGCAGCAGGACCAGGATCCAGGAGGGGACA
CCATGGTCCACAGGGTAACA

>441 Get primers
ATGAAGCCAGAGTTGTTAGCGAAGGACCAGATATCCCCCTCATTCCCTGTGTAGAAAAAG
ATGGGCCCTTCGCCCATCTTCCAGAACTTATCTGTTGGAAGTAAATGAGTTTCCATAAGG
CCAGGGAAACGCAGGTAGGAACCCATGCGGTCGAGCCAGCACTCACCTGACACTAGGAAC
CGCTGGCCAAAGGTTTTGTTGCCGAAACTCTCAAAGTTGAAATGGTCCATGTATTGCTCA
AAATAATTCTCATGAAAGTCAGGGTCTAGAACTCTGTCGGCTGAGGGCAGGTGCAGAGAC
TCAGGAGCTGGTTGGGATCATCAGGGATCTAGGCGGGTCAGGAGGAAGGGCAGCCAGTCT
GTACTCACCTCTGGCCTGGAGGTTGCACAGTCCCAGTGACAGCAGCAGGACCAGGATCCA
GGAGGGGACACCATGGTCCACAGGGTAACAAGGATGGAAGTTCATGCTTGATTCTGAGCC
GGGCGCTGACTGTCATGTGA

>442 Get primers
GTAGAAAAAGATGGGCCCTTCGCCCATCTTCCAGAACTTATCTGTTGGAAGTAAATGAGT
TTCCATAAGGCCAGGGAAACGCAGGTAGGAACCCATGCGGTCGAGCCAGCACTCACCTGA
CACTAGGAACCGCTGGCCAAAGGTTTTGTTGCCGAAACTCTCAAAGTTGAAATGGTCCAT
GTATTGCTCAAAATAATTCTCATGAAAGTCAGGGTCTAGAACTCTGTCGGCTGAGGGCAG
GTGCAGAGACTCAGGAGCTGGTTGGGATCATCAGGGATCTAGGCGGGTCAGGAGGAAGGG
CAGCCAGTCTGTACTCACCTCTGGCCTGGAGGTTGCACAGTCCCAGTGACAGCAGCAGGA
CCAGGATCCAGGAGGGGACACCATGGTCCACAGGGTAACAAGGATGGAAGTTCATGCTTG
ATTCTGAGCCGGGCGCTGACTGTCATGTGATTTGGTCACATGACCGACACAACGGGCGGG
GCAGCATCACGTGATAGTCT

>443 Get primers
GTAAATGAGTTTCCATAAGGCCAGGGAAACGCAGGTAGGAACCCATGCGGTCGAGCCAGC
ACTCACCTGACACTAGGAACCGCTGGCCAAAGGTTTTGTTGCCGAAACTCTCAAAGTTGA
AATGGTCCATGTATTGCTCAAAATAATTCTCATGAAAGTCAGGGTCTAGAACTCTGTCGG
CTGAGGGCAGGTGCAGAGACTCAGGAGCTGGTTGGGATCATCAGGGATCTAGGCGGGTCA
GGAGGAAGGGCAGCCAGTCTGTACTCACCTCTGGCCTGGAGGTTGCACAGTCCCAGTGAC
AGCAGCAGGACCAGGATCCAGGAGGGGACACCATGGTCCACAGGGTAACAAGGATGGAAG
TTCATGCTTGATTCTGAGCCGGGCGCTGACTGTCATGTGATTTGGTCACATGACCGACAC
AACGGGCGGGGCAGCATCACGTGATAGTCTGGCGGGGGCTGTCCTACTGTGGCTGGATTC
TAGTTGGAGGATCAGCCTAC

>444 Get primers
TCGAGCCAGCACTCACCTGACACTAGGAACCGCTGGCCAAAGGTTTTGTTGCCGAAACTC
TCAAAGTTGAAATGGTCCATGTATTGCTCAAAATAATTCTCATGAAAGTCAGGGTCTAGA
ACTCTGTCGGCTGAGGGCAGGTGCAGAGACTCAGGAGCTGGTTGGGATCATCAGGGATCT
AGGCGGGTCAGGAGGAAGGGCAGCCAGTCTGTACTCACCTCTGGCCTGGAGGTTGCACAG
TCCCAGTGACAGCAGCAGGACCAGGATCCAGGAGGGGACACCATGGTCCACAGGGTAACA
AGGATGGAAGTTCATGCTTGATTCTGAGCCGGGCGCTGACTGTCATGTGATTTGGTCACA
TGACCGACACAACGGGCGGGGCAGCATCACGTGATAGTCTGGCGGGGGCTGTCCTACTGT
GGCTGGATTCTAGTTGGAGGATCAGCCTACTCTTCTTCAGTTTCCCGGTTCCTCCAAATT
TCTGGGCTCCTACTTGTTTC

>445 Get primers
GCCGAAACTCTCAAAGTTGAAATGGTCCATGTATTGCTCAAAATAATTCTCATGAAAGTC
AGGGTCTAGAACTCTGTCGGCTGAGGGCAGGTGCAGAGACTCAGGAGCTGGTTGGGATCA
TCAGGGATCTAGGCGGGTCAGGAGGAAGGGCAGCCAGTCTGTACTCACCTCTGGCCTGGA
GGTTGCACAGTCCCAGTGACAGCAGCAGGACCAGGATCCAGGAGGGGACACCATGGTCCA
CAGGGTAACAAGGATGGAAGTTCATGCTTGATTCTGAGCCGGGCGCTGACTGTCATGTGA
TTTGGTCACATGACCGACACAACGGGCGGGGCAGCATCACGTGATAGTCTGGCGGGGGCT
GTCCTACTGTGGCTGGATTCTAGTTGGAGGATCAGCCTACTCTTCTTCAGTTTCCCGGTT
CCTCCAAATTTCTGGGCTCCTACTTGTTTCCACAGAGATGGATACTGTGGAGGTCCAGGA
AGCAGAGAGATGGCTAAGGC

>446 Get primers
CATGAAAGTCAGGGTCTAGAACTCTGTCGGCTGAGGGCAGGTGCAGAGACTCAGGAGCTG
GTTGGGATCATCAGGGATCTAGGCGGGTCAGGAGGAAGGGCAGCCAGTCTGTACTCACCT
CTGGCCTGGAGGTTGCACAGTCCCAGTGACAGCAGCAGGACCAGGATCCAGGAGGGGACA
CCATGGTCCACAGGGTAACAAGGATGGAAGTTCATGCTTGATTCTGAGCCGGGCGCTGAC
TGTCATGTGATTTGGTCACATGACCGACACAACGGGCGGGGCAGCATCACGTGATAGTCT
GGCGGGGGCTGTCCTACTGTGGCTGGATTCTAGTTGGAGGATCAGCCTACTCTTCTTCAG
TTTCCCGGTTCCTCCAAATTTCTGGGCTCCTACTTGTTTCCACAGAGATGGATACTGTGG
AGGTCCAGGAAGCAGAGAGATGGCTAAGGCTCATCAGGACCGTATGATCTCCCAAGTGTC
CAGCTACTGAGTACCACAAG

>447 Get primers
TCAGGAGCTGGTTGGGATCATCAGGGATCTAGGCGGGTCAGGAGGAAGGGCAGCCAGTCT
GTACTCACCTCTGGCCTGGAGGTTGCACAGTCCCAGTGACAGCAGCAGGACCAGGATCCA
GGAGGGGACACCATGGTCCACAGGGTAACAAGGATGGAAGTTCATGCTTGATTCTGAGCC
GGGCGCTGACTGTCATGTGATTTGGTCACATGACCGACACAACGGGCGGGGCAGCATCAC
GTGATAGTCTGGCGGGGGCTGTCCTACTGTGGCTGGATTCTAGTTGGAGGATCAGCCTAC
TCTTCTTCAGTTTCCCGGTTCCTCCAAATTTCTGGGCTCCTACTTGTTTCCACAGAGATG
GATACTGTGGAGGTCCAGGAAGCAGAGAGATGGCTAAGGCTCATCAGGACCGTATGATCT
CCCAAGTGTCCAGCTACTGAGTACCACAAGGTGATGGGTGGGAGGGTCCTCCCACGGAAG
GATACCGCAGTCCCTAGGGG

>448 Get primers
CAGCCAGTCTGTACTCACCTCTGGCCTGGAGGTTGCACAGTCCCAGTGACAGCAGCAGGA
CCAGGATCCAGGAGGGGACACCATGGTCCACAGGGTAACAAGGATGGAAGTTCATGCTTG
ATTCTGAGCCGGGCGCTGACTGTCATGTGATTTGGTCACATGACCGACACAACGGGCGGG
GCAGCATCACGTGATAGTCTGGCGGGGGCTGTCCTACTGTGGCTGGATTCTAGTTGGAGG
ATCAGCCTACTCTTCTTCAGTTTCCCGGTTCCTCCAAATTTCTGGGCTCCTACTTGTTTC
CACAGAGATGGATACTGTGGAGGTCCAGGAAGCAGAGAGATGGCTAAGGCTCATCAGGAC
CGTATGATCTCCCAAGTGTCCAGCTACTGAGTACCACAAGGTGATGGGTGGGAGGGTCCT
CCCACGGAAGGATACCGCAGTCCCTAGGGGTTGCAAGCCCCACATGTTCCACTGGCTGCT
AGAGCTACCTACTCAATCAG

>449 Get primers
AGCAGCAGGACCAGGATCCAGGAGGGGACACCATGGTCCACAGGGTAACAAGGATGGAAG
TTCATGCTTGATTCTGAGCCGGGCGCTGACTGTCATGTGATTTGGTCACATGACCGACAC
AACGGGCGGGGCAGCATCACGTGATAGTCTGGCGGGGGCTGTCCTACTGTGGCTGGATTC
TAGTTGGAGGATCAGCCTACTCTTCTTCAGTTTCCCGGTTCCTCCAAATTTCTGGGCTCC
TACTTGTTTCCACAGAGATGGATACTGTGGAGGTCCAGGAAGCAGAGAGATGGCTAAGGC
TCATCAGGACCGTATGATCTCCCAAGTGTCCAGCTACTGAGTACCACAAGGTGATGGGTG
GGAGGGTCCTCCCACGGAAGGATACCGCAGTCCCTAGGGGTTGCAAGCCCCACATGTTCC
ACTGGCTGCTAGAGCTACCTACTCAATCAGCCCTGGGCATCACCATCAGGTACTCGGCCA
AAATGACCTCTCTGCTTCCA

>450 Get primers
AGGATGGAAGTTCATGCTTGATTCTGAGCCGGGCGCTGACTGTCATGTGATTTGGTCACA
TGACCGACACAACGGGCGGGGCAGCATCACGTGATAGTCTGGCGGGGGCTGTCCTACTGT
GGCTGGATTCTAGTTGGAGGATCAGCCTACTCTTCTTCAGTTTCCCGGTTCCTCCAAATT
TCTGGGCTCCTACTTGTTTCCACAGAGATGGATACTGTGGAGGTCCAGGAAGCAGAGAGA
TGGCTAAGGCTCATCAGGACCGTATGATCTCCCAAGTGTCCAGCTACTGAGTACCACAAG
GTGATGGGTGGGAGGGTCCTCCCACGGAAGGATACCGCAGTCCCTAGGGGTTGCAAGCCC
CACATGTTCCACTGGCTGCTAGAGCTACCTACTCAATCAGCCCTGGGCATCACCATCAGG
TACTCGGCCAAAATGACCTCTCTGCTTCCAGTCCTCAGTTCTGGTCAGCACCAGACAGGC
CCATAATTACAGAGCCAGGG

>451 Get primers
TTTGGTCACATGACCGACACAACGGGCGGGGCAGCATCACGTGATAGTCTGGCGGGGGCT
GTCCTACTGTGGCTGGATTCTAGTTGGAGGATCAGCCTACTCTTCTTCAGTTTCCCGGTT
CCTCCAAATTTCTGGGCTCCTACTTGTTTCCACAGAGATGGATACTGTGGAGGTCCAGGA
AGCAGAGAGATGGCTAAGGCTCATCAGGACCGTATGATCTCCCAAGTGTCCAGCTACTGA
GTACCACAAGGTGATGGGTGGGAGGGTCCTCCCACGGAAGGATACCGCAGTCCCTAGGGG
TTGCAAGCCCCACATGTTCCACTGGCTGCTAGAGCTACCTACTCAATCAGCCCTGGGCAT
CACCATCAGGTACTCGGCCAAAATGACCTCTCTGCTTCCAGTCCTCAGTTCTGGTCAGCA
CCAGACAGGCCCATAATTACAGAGCCAGGGAAACTGGAACATTTGTCTCCCCTTAGACAG
TGGCAGCAGGAAGGTGGGGG

>452 Get primers
GGCGGGGGCTGTCCTACTGTGGCTGGATTCTAGTTGGAGGATCAGCCTACTCTTCTTCAG
TTTCCCGGTTCCTCCAAATTTCTGGGCTCCTACTTGTTTCCACAGAGATGGATACTGTGG
AGGTCCAGGAAGCAGAGAGATGGCTAAGGCTCATCAGGACCGTATGATCTCCCAAGTGTC
CAGCTACTGAGTACCACAAGGTGATGGGTGGGAGGGTCCTCCCACGGAAGGATACCGCAG
TCCCTAGGGGTTGCAAGCCCCACATGTTCCACTGGCTGCTAGAGCTACCTACTCAATCAG
CCCTGGGCATCACCATCAGGTACTCGGCCAAAATGACCTCTCTGCTTCCAGTCCTCAGTT
CTGGTCAGCACCAGACAGGCCCATAATTACAGAGCCAGGGAAACTGGAACATTTGTCTCC
CCTTAGACAGTGGCAGCAGGAAGGTGGGGGGTTGTTGCAGAGGAACAGTGTCTCTGAGAG
AGGACCTTGGACTTTCTGGG

>453 Get primers
TCTTCTTCAGTTTCCCGGTTCCTCCAAATTTCTGGGCTCCTACTTGTTTCCACAGAGATG
GATACTGTGGAGGTCCAGGAAGCAGAGAGATGGCTAAGGCTCATCAGGACCGTATGATCT
CCCAAGTGTCCAGCTACTGAGTACCACAAGGTGATGGGTGGGAGGGTCCTCCCACGGAAG
GATACCGCAGTCCCTAGGGGTTGCAAGCCCCACATGTTCCACTGGCTGCTAGAGCTACCT
ACTCAATCAGCCCTGGGCATCACCATCAGGTACTCGGCCAAAATGACCTCTCTGCTTCCA
GTCCTCAGTTCTGGTCAGCACCAGACAGGCCCATAATTACAGAGCCAGGGAAACTGGAAC
ATTTGTCTCCCCTTAGACAGTGGCAGCAGGAAGGTGGGGGGTTGTTGCAGAGGAACAGTG
TCTCTGAGAGAGGACCTTGGACTTTCTGGGAATCTCTGAGCTGCCCGGTTCTCCCCACTG
CTGGCACTGTGCCCACAGCC

>454 Get primers
CACAGAGATGGATACTGTGGAGGTCCAGGAAGCAGAGAGATGGCTAAGGCTCATCAGGAC
CGTATGATCTCCCAAGTGTCCAGCTACTGAGTACCACAAGGTGATGGGTGGGAGGGTCCT
CCCACGGAAGGATACCGCAGTCCCTAGGGGTTGCAAGCCCCACATGTTCCACTGGCTGCT
AGAGCTACCTACTCAATCAGCCCTGGGCATCACCATCAGGTACTCGGCCAAAATGACCTC
TCTGCTTCCAGTCCTCAGTTCTGGTCAGCACCAGACAGGCCCATAATTACAGAGCCAGGG
AAACTGGAACATTTGTCTCCCCTTAGACAGTGGCAGCAGGAAGGTGGGGGGTTGTTGCAG
AGGAACAGTGTCTCTGAGAGAGGACCTTGGACTTTCTGGGAATCTCTGAGCTGCCCGGTT
CTCCCCACTGCTGGCACTGTGCCCACAGCCCAAACAGAATGGGGGAGATGGAGGGGCAGG
GCTTCTGTGGGAAGCTGCCC

>455 Get primers
TCATCAGGACCGTATGATCTCCCAAGTGTCCAGCTACTGAGTACCACAAGGTGATGGGTG
GGAGGGTCCTCCCACGGAAGGATACCGCAGTCCCTAGGGGTTGCAAGCCCCACATGTTCC
ACTGGCTGCTAGAGCTACCTACTCAATCAGCCCTGGGCATCACCATCAGGTACTCGGCCA
AAATGACCTCTCTGCTTCCAGTCCTCAGTTCTGGTCAGCACCAGACAGGCCCATAATTAC
AGAGCCAGGGAAACTGGAACATTTGTCTCCCCTTAGACAGTGGCAGCAGGAAGGTGGGGG
GTTGTTGCAGAGGAACAGTGTCTCTGAGAGAGGACCTTGGACTTTCTGGGAATCTCTGAG
CTGCCCGGTTCTCCCCACTGCTGGCACTGTGCCCACAGCCCAAACAGAATGGGGGAGATG
GAGGGGCAGGGCTTCTGTGGGAAGCTGCCCTCCACCTCATTGGCACAGAGTGTCTCATTG
CAGAGAGAAAAAAGGACCAG

>456 Get primers
GTGATGGGTGGGAGGGTCCTCCCACGGAAGGATACCGCAGTCCCTAGGGGTTGCAAGCCC
CACATGTTCCACTGGCTGCTAGAGCTACCTACTCAATCAGCCCTGGGCATCACCATCAGG
TACTCGGCCAAAATGACCTCTCTGCTTCCAGTCCTCAGTTCTGGTCAGCACCAGACAGGC
CCATAATTACAGAGCCAGGGAAACTGGAACATTTGTCTCCCCTTAGACAGTGGCAGCAGG
AAGGTGGGGGGTTGTTGCAGAGGAACAGTGTCTCTGAGAGAGGACCTTGGACTTTCTGGG
AATCTCTGAGCTGCCCGGTTCTCCCCACTGCTGGCACTGTGCCCACAGCCCAAACAGAAT
GGGGGAGATGGAGGGGCAGGGCTTCTGTGGGAAGCTGCCCTCCACCTCATTGGCACAGAG
TGTCTCATTGCAGAGAGAAAAAAGGACCAGTTTTCTCTCTGGCACCCAGGTCTGGAAGAG
GAGTGACATCCACGGAAGTT

>457 Get primers
TTGCAAGCCCCACATGTTCCACTGGCTGCTAGAGCTACCTACTCAATCAGCCCTGGGCAT
CACCATCAGGTACTCGGCCAAAATGACCTCTCTGCTTCCAGTCCTCAGTTCTGGTCAGCA
CCAGACAGGCCCATAATTACAGAGCCAGGGAAACTGGAACATTTGTCTCCCCTTAGACAG
TGGCAGCAGGAAGGTGGGGGGTTGTTGCAGAGGAACAGTGTCTCTGAGAGAGGACCTTGG
ACTTTCTGGGAATCTCTGAGCTGCCCGGTTCTCCCCACTGCTGGCACTGTGCCCACAGCC
CAAACAGAATGGGGGAGATGGAGGGGCAGGGCTTCTGTGGGAAGCTGCCCTCCACCTCAT
TGGCACAGAGTGTCTCATTGCAGAGAGAAAAAAGGACCAGTTTTCTCTCTGGCACCCAGG
TCTGGAAGAGGAGTGACATCCACGGAAGTTGGTGACTTGGACTGGCTGGCCGTGAGTGGA
ACATGTCCATCCAGCATGGC

>458 Get primers
CCCTGGGCATCACCATCAGGTACTCGGCCAAAATGACCTCTCTGCTTCCAGTCCTCAGTT
CTGGTCAGCACCAGACAGGCCCATAATTACAGAGCCAGGGAAACTGGAACATTTGTCTCC
CCTTAGACAGTGGCAGCAGGAAGGTGGGGGGTTGTTGCAGAGGAACAGTGTCTCTGAGAG
AGGACCTTGGACTTTCTGGGAATCTCTGAGCTGCCCGGTTCTCCCCACTGCTGGCACTGT
GCCCACAGCCCAAACAGAATGGGGGAGATGGAGGGGCAGGGCTTCTGTGGGAAGCTGCCC
TCCACCTCATTGGCACAGAGTGTCTCATTGCAGAGAGAAAAAAGGACCAGTTTTCTCTCT
GGCACCCAGGTCTGGAAGAGGAGTGACATCCACGGAAGTTGGTGACTTGGACTGGCTGGC
CGTGAGTGGAACATGTCCATCCAGCATGGCCACAGTCCAGTGGGACACACAGCCTAGAGC
TGTGGAATGCCGTGCCACAG


PROBE SEQUENCES
---------------

>3 Get primers
CAGGGTTGGCAGGAAGGGGCCCTAGAAAGTCAGTAGGATACGGGTAGTCCATCATGGCGA
GCACAGTAAATGCATTTCGGGCAAACCCAAAGAGCTGAGTCAGGTCCTTTGGGCTGGAAA
GTGATTGACAGGTACCAAAGTTCTGGCTGATGGTGTCATAGGCTGGGAAGAGAGAGGCCA
GGAGAAAAGGCTGAGGAAACTGCTGGCAAATGTGAAGGGCAAGAATGAATGCCCAAGGTG
GGCAGCAGGTGAGGAAAGAGTCCCTCACCTCCCTGGAGGAACAAGTCTTTGATTTGCTGA
AAGGCATCCCGCACAGCCTGGGCGCACTTGGGACTCTGGCCATAAAAGTCCTGGAGAAGA
GACCAAGGTTGCTGCTGCCATTCTTGCACTGGCCTGGGGTACCCAAGTCCCCTCACTCAC
CGCTGTGACATCTCGGAAGAATTGGTAGGAGTCCCCAAGGCCTGCAACAGCTACAACAGG
AGCGCTGGCTGCCAGTGCCCCAGCCACCAGGTGGGGGTACTTCATCCTCATGTAGGCACT
CAGCATCCCCCCATAACTGGGAGTACAGAGCACAGATCATGGTTGTGGGAAGCTGCCCAC
AACTCAGGCGAGCAGCCTCACTGTCCTCCAGGCTGAGGTGCTAGGCTGCTCTTTCCCTGC
TCAGAACGCCCAAGGGTGGGAAAGAAGGACCTGAAACTGTCAGGCCCACACACCCTGATC
CCAGGGCCAAGGCAGATACAGCCTTCACTGGGAGAAGGCACCTGTGGGTGCCCTGCCCTG
ACCCAGCAATGAAGACATTGCAGAGACAAAGTCAGAAGGAATTGTCCCACTAGTGGGAAC
AACATAGCATACACTGCCTATGAGGTCCACTCAAGGAGGGCTTCCAGAAGGAGGTAAAGC
TAGACCCCGCCCTTCCACATGTGGGGTAGGCATAGGATGTTGAGACTGTAAGAGACATCT
CTTTGGCCCTCCTTGTATAGGGTGTCAATCGGCACAACAGGGTGGAGCCTTAGAGTAGGG
TAAGATTAGGACTCTAGGTTCTCTCATGGGTCCAGATCTGTCATGAAGGGAGGTCAAGGA
CCCACCTCCCTCCAAAGGCTATGGTGGGGGCATCATGGACCCCAAGGTCCTGCCGCAGGG
CCTGGAGCAGCACAGCAAAGTCGGCCAGCGCCTGCTCCACAGTCAGCAGCTGTGTATATC
CCCGCTGTGTGGACTGGACACCGAACGGAAGCGATTTCCCATAGTACCGCTGCAGAAAGC
AGGAAGGGATGGCTAATCCACTCCTCGGTGCTCCCCACCT

>4 Get primers
GGTACCAAAGTTCTGGCTGATGGTGTCATAGGCTGGGAAGAGAGAGGCCAGGAGAAAAGG
CTGAGGAAACTGCTGGCAAATGTGAAGGGCAAGAATGAATGCCCAAGGTGGGCAGCAGGT
GAGGAAAGAGTCCCTCACCTCCCTGGAGGAACAAGTCTTTGATTTGCTGAAAGGCATCCC
GCACAGCCTGGGCGCACTTGGGACTCTGGCCATAAAAGTCCTGGAGAAGAGACCAAGGTT
GCTGCTGCCATTCTTGCACTGGCCTGGGGTACCCAAGTCCCCTCACTCACCGCTGTGACA
TCTCGGAAGAATTGGTAGGAGTCCCCAAGGCCTGCAACAGCTACAACAGGAGCGCTGGCT
GCCAGTGCCCCAGCCACCAGGTGGGGGTACTTCATCCTCATGTAGGCACTCAGCATCCCC
CCATAACTGGGAGTACAGAGCACAGATCATGGTTGTGGGAAGCTGCCCACAACTCAGGCG
AGCAGCCTCACTGTCCTCCAGGCTGAGGTGCTAGGCTGCTCTTTCCCTGCTCAGAACGCC
CAAGGGTGGGAAAGAAGGACCTGAAACTGTCAGGCCCACACACCCTGATCCCAGGGCCAA
GGCAGATACAGCCTTCACTGGGAGAAGGCACCTGTGGGTGCCCTGCCCTGACCCAGCAAT
GAAGACATTGCAGAGACAAAGTCAGAAGGAATTGTCCCACTAGTGGGAACAACATAGCAT
ACACTGCCTATGAGGTCCACTCAAGGAGGGCTTCCAGAAGGAGGTAAAGCTAGACCCCGC
CCTTCCACATGTGGGGTAGGCATAGGATGTTGAGACTGTAAGAGACATCTCTTTGGCCCT
CCTTGTATAGGGTGTCAATCGGCACAACAGGGTGGAGCCTTAGAGTAGGGTAAGATTAGG
ACTCTAGGTTCTCTCATGGGTCCAGATCTGTCATGAAGGGAGGTCAAGGACCCACCTCCC
TCCAAAGGCTATGGTGGGGGCATCATGGACCCCAAGGTCCTGCCGCAGGGCCTGGAGCAG
CACAGCAAAGTCGGCCAGCGCCTGCTCCACAGTCAGCAGCTGTGTATATCCCCGCTGTGT
GGACTGGACACCGAACGGAAGCGATTTCCCATAGTACCGCTGCAGAAAGCAGGAAGGGAT
GGCTAATCCACTCCTCGGTGCTCCCCACCTCCTTCAACTCAGGGACTGCCAGGAACTGTA
CAGGTACCCACGTGCTCAGCAAAGACAAGCAGGGCCTCCTGCTGGGCTGCCAGTTCCACC
ATGAAGCCAGAGTTGTTAGCGAAGGACCAGATATCCCCCT

>5 Get primers
TCCCTCACCTCCCTGGAGGAACAAGTCTTTGATTTGCTGAAAGGCATCCCGCACAGCCTG
GGCGCACTTGGGACTCTGGCCATAAAAGTCCTGGAGAAGAGACCAAGGTTGCTGCTGCCA
TTCTTGCACTGGCCTGGGGTACCCAAGTCCCCTCACTCACCGCTGTGACATCTCGGAAGA
ATTGGTAGGAGTCCCCAAGGCCTGCAACAGCTACAACAGGAGCGCTGGCTGCCAGTGCCC
CAGCCACCAGGTGGGGGTACTTCATCCTCATGTAGGCACTCAGCATCCCCCCATAACTGG
GAGTACAGAGCACAGATCATGGTTGTGGGAAGCTGCCCACAACTCAGGCGAGCAGCCTCA
CTGTCCTCCAGGCTGAGGTGCTAGGCTGCTCTTTCCCTGCTCAGAACGCCCAAGGGTGGG
AAAGAAGGACCTGAAACTGTCAGGCCCACACACCCTGATCCCAGGGCCAAGGCAGATACA
GCCTTCACTGGGAGAAGGCACCTGTGGGTGCCCTGCCCTGACCCAGCAATGAAGACATTG
CAGAGACAAAGTCAGAAGGAATTGTCCCACTAGTGGGAACAACATAGCATACACTGCCTA
TGAGGTCCACTCAAGGAGGGCTTCCAGAAGGAGGTAAAGCTAGACCCCGCCCTTCCACAT
GTGGGGTAGGCATAGGATGTTGAGACTGTAAGAGACATCTCTTTGGCCCTCCTTGTATAG
GGTGTCAATCGGCACAACAGGGTGGAGCCTTAGAGTAGGGTAAGATTAGGACTCTAGGTT
CTCTCATGGGTCCAGATCTGTCATGAAGGGAGGTCAAGGACCCACCTCCCTCCAAAGGCT
ATGGTGGGGGCATCATGGACCCCAAGGTCCTGCCGCAGGGCCTGGAGCAGCACAGCAAAG
TCGGCCAGCGCCTGCTCCACAGTCAGCAGCTGTGTATATCCCCGCTGTGTGGACTGGACA
CCGAACGGAAGCGATTTCCCATAGTACCGCTGCAGAAAGCAGGAAGGGATGGCTAATCCA
CTCCTCGGTGCTCCCCACCTCCTTCAACTCAGGGACTGCCAGGAACTGTACAGGTACCCA
CGTGCTCAGCAAAGACAAGCAGGGCCTCCTGCTGGGCTGCCAGTTCCACCATGAAGCCAG
AGTTGTTAGCGAAGGACCAGATATCCCCCTCATTCCCTGTGTAGAAAAAGATGGGCCCTT
CGCCCATCTTCCAGAACTTATCTGTTGGAAGTAAATGAGTTTCCATAAGGCCAGGGAAAC
GCAGGTAGGAACCCATGCGGTCGAGCCAGCACTCACCTGA

>6 Get primers
GGCCTGGGGTACCCAAGTCCCCTCACTCACCGCTGTGACATCTCGGAAGAATTGGTAGGA
GTCCCCAAGGCCTGCAACAGCTACAACAGGAGCGCTGGCTGCCAGTGCCCCAGCCACCAG
GTGGGGGTACTTCATCCTCATGTAGGCACTCAGCATCCCCCCATAACTGGGAGTACAGAG
CACAGATCATGGTTGTGGGAAGCTGCCCACAACTCAGGCGAGCAGCCTCACTGTCCTCCA
GGCTGAGGTGCTAGGCTGCTCTTTCCCTGCTCAGAACGCCCAAGGGTGGGAAAGAAGGAC
CTGAAACTGTCAGGCCCACACACCCTGATCCCAGGGCCAAGGCAGATACAGCCTTCACTG
GGAGAAGGCACCTGTGGGTGCCCTGCCCTGACCCAGCAATGAAGACATTGCAGAGACAAA
GTCAGAAGGAATTGTCCCACTAGTGGGAACAACATAGCATACACTGCCTATGAGGTCCAC
TCAAGGAGGGCTTCCAGAAGGAGGTAAAGCTAGACCCCGCCCTTCCACATGTGGGGTAGG
CATAGGATGTTGAGACTGTAAGAGACATCTCTTTGGCCCTCCTTGTATAGGGTGTCAATC
GGCACAACAGGGTGGAGCCTTAGAGTAGGGTAAGATTAGGACTCTAGGTTCTCTCATGGG
TCCAGATCTGTCATGAAGGGAGGTCAAGGACCCACCTCCCTCCAAAGGCTATGGTGGGGG
CATCATGGACCCCAAGGTCCTGCCGCAGGGCCTGGAGCAGCACAGCAAAGTCGGCCAGCG
CCTGCTCCACAGTCAGCAGCTGTGTATATCCCCGCTGTGTGGACTGGACACCGAACGGAA
GCGATTTCCCATAGTACCGCTGCAGAAAGCAGGAAGGGATGGCTAATCCACTCCTCGGTG
CTCCCCACCTCCTTCAACTCAGGGACTGCCAGGAACTGTACAGGTACCCACGTGCTCAGC
AAAGACAAGCAGGGCCTCCTGCTGGGCTGCCAGTTCCACCATGAAGCCAGAGTTGTTAGC
GAAGGACCAGATATCCCCCTCATTCCCTGTGTAGAAAAAGATGGGCCCTTCGCCCATCTT
CCAGAACTTATCTGTTGGAAGTAAATGAGTTTCCATAAGGCCAGGGAAACGCAGGTAGGA
ACCCATGCGGTCGAGCCAGCACTCACCTGACACTAGGAACCGCTGGCCAAAGGTTTTGTT
GCCGAAACTCTCAAAGTTGAAATGGTCCATGTATTGCTCAAAATAATTCTCATGAAAGTC
AGGGTCTAGAACTCTGTCGGCTGAGGGCAGGTGCAGAGAC

>7 Get primers
TTCATCCTCATGTAGGCACTCAGCATCCCCCCATAACTGGGAGTACAGAGCACAGATCAT
GGTTGTGGGAAGCTGCCCACAACTCAGGCGAGCAGCCTCACTGTCCTCCAGGCTGAGGTG
CTAGGCTGCTCTTTCCCTGCTCAGAACGCCCAAGGGTGGGAAAGAAGGACCTGAAACTGT
CAGGCCCACACACCCTGATCCCAGGGCCAAGGCAGATACAGCCTTCACTGGGAGAAGGCA
CCTGTGGGTGCCCTGCCCTGACCCAGCAATGAAGACATTGCAGAGACAAAGTCAGAAGGA
ATTGTCCCACTAGTGGGAACAACATAGCATACACTGCCTATGAGGTCCACTCAAGGAGGG
CTTCCAGAAGGAGGTAAAGCTAGACCCCGCCCTTCCACATGTGGGGTAGGCATAGGATGT
TGAGACTGTAAGAGACATCTCTTTGGCCCTCCTTGTATAGGGTGTCAATCGGCACAACAG
GGTGGAGCCTTAGAGTAGGGTAAGATTAGGACTCTAGGTTCTCTCATGGGTCCAGATCTG
TCATGAAGGGAGGTCAAGGACCCACCTCCCTCCAAAGGCTATGGTGGGGGCATCATGGAC
CCCAAGGTCCTGCCGCAGGGCCTGGAGCAGCACAGCAAAGTCGGCCAGCGCCTGCTCCAC
AGTCAGCAGCTGTGTATATCCCCGCTGTGTGGACTGGACACCGAACGGAAGCGATTTCCC
ATAGTACCGCTGCAGAAAGCAGGAAGGGATGGCTAATCCACTCCTCGGTGCTCCCCACCT
CCTTCAACTCAGGGACTGCCAGGAACTGTACAGGTACCCACGTGCTCAGCAAAGACAAGC
AGGGCCTCCTGCTGGGCTGCCAGTTCCACCATGAAGCCAGAGTTGTTAGCGAAGGACCAG
ATATCCCCCTCATTCCCTGTGTAGAAAAAGATGGGCCCTTCGCCCATCTTCCAGAACTTA
TCTGTTGGAAGTAAATGAGTTTCCATAAGGCCAGGGAAACGCAGGTAGGAACCCATGCGG
TCGAGCCAGCACTCACCTGACACTAGGAACCGCTGGCCAAAGGTTTTGTTGCCGAAACTC
TCAAAGTTGAAATGGTCCATGTATTGCTCAAAATAATTCTCATGAAAGTCAGGGTCTAGA
ACTCTGTCGGCTGAGGGCAGGTGCAGAGACTCAGGAGCTGGTTGGGATCATCAGGGATCT
AGGCGGGTCAGGAGGAAGGGCAGCCAGTCTGTACTCACCTCTGGCCTGGAGGTTGCACAG
TCCCAGTGACAGCAGCAGGACCAGGATCCAGGAGGGGACA

>8 Get primers
CTTTCCCTGCTCAGAACGCCCAAGGGTGGGAAAGAAGGACCTGAAACTGTCAGGCCCACA
CACCCTGATCCCAGGGCCAAGGCAGATACAGCCTTCACTGGGAGAAGGCACCTGTGGGTG
CCCTGCCCTGACCCAGCAATGAAGACATTGCAGAGACAAAGTCAGAAGGAATTGTCCCAC
TAGTGGGAACAACATAGCATACACTGCCTATGAGGTCCACTCAAGGAGGGCTTCCAGAAG
GAGGTAAAGCTAGACCCCGCCCTTCCACATGTGGGGTAGGCATAGGATGTTGAGACTGTA
AGAGACATCTCTTTGGCCCTCCTTGTATAGGGTGTCAATCGGCACAACAGGGTGGAGCCT
TAGAGTAGGGTAAGATTAGGACTCTAGGTTCTCTCATGGGTCCAGATCTGTCATGAAGGG
AGGTCAAGGACCCACCTCCCTCCAAAGGCTATGGTGGGGGCATCATGGACCCCAAGGTCC
TGCCGCAGGGCCTGGAGCAGCACAGCAAAGTCGGCCAGCGCCTGCTCCACAGTCAGCAGC
TGTGTATATCCCCGCTGTGTGGACTGGACACCGAACGGAAGCGATTTCCCATAGTACCGC
TGCAGAAAGCAGGAAGGGATGGCTAATCCACTCCTCGGTGCTCCCCACCTCCTTCAACTC
AGGGACTGCCAGGAACTGTACAGGTACCCACGTGCTCAGCAAAGACAAGCAGGGCCTCCT
GCTGGGCTGCCAGTTCCACCATGAAGCCAGAGTTGTTAGCGAAGGACCAGATATCCCCCT
CATTCCCTGTGTAGAAAAAGATGGGCCCTTCGCCCATCTTCCAGAACTTATCTGTTGGAA
GTAAATGAGTTTCCATAAGGCCAGGGAAACGCAGGTAGGAACCCATGCGGTCGAGCCAGC
ACTCACCTGACACTAGGAACCGCTGGCCAAAGGTTTTGTTGCCGAAACTCTCAAAGTTGA
AATGGTCCATGTATTGCTCAAAATAATTCTCATGAAAGTCAGGGTCTAGAACTCTGTCGG
CTGAGGGCAGGTGCAGAGACTCAGGAGCTGGTTGGGATCATCAGGGATCTAGGCGGGTCA
GGAGGAAGGGCAGCCAGTCTGTACTCACCTCTGGCCTGGAGGTTGCACAGTCCCAGTGAC
AGCAGCAGGACCAGGATCCAGGAGGGGACACCATGGTCCACAGGGTAACAAGGATGGAAG
TTCATGCTTGATTCTGAGCCGGGCGCTGACTGTCATGTGATTTGGTCACATGACCGACAC
AACGGGCGGGGCAGCATCACGTGATAGTCTGGCGGGGGCT

>9 Get primers
ACCCAGCAATGAAGACATTGCAGAGACAAAGTCAGAAGGAATTGTCCCACTAGTGGGAAC
AACATAGCATACACTGCCTATGAGGTCCACTCAAGGAGGGCTTCCAGAAGGAGGTAAAGC
TAGACCCCGCCCTTCCACATGTGGGGTAGGCATAGGATGTTGAGACTGTAAGAGACATCT
CTTTGGCCCTCCTTGTATAGGGTGTCAATCGGCACAACAGGGTGGAGCCTTAGAGTAGGG
TAAGATTAGGACTCTAGGTTCTCTCATGGGTCCAGATCTGTCATGAAGGGAGGTCAAGGA
CCCACCTCCCTCCAAAGGCTATGGTGGGGGCATCATGGACCCCAAGGTCCTGCCGCAGGG
CCTGGAGCAGCACAGCAAAGTCGGCCAGCGCCTGCTCCACAGTCAGCAGCTGTGTATATC
CCCGCTGTGTGGACTGGACACCGAACGGAAGCGATTTCCCATAGTACCGCTGCAGAAAGC
AGGAAGGGATGGCTAATCCACTCCTCGGTGCTCCCCACCTCCTTCAACTCAGGGACTGCC
AGGAACTGTACAGGTACCCACGTGCTCAGCAAAGACAAGCAGGGCCTCCTGCTGGGCTGC
CAGTTCCACCATGAAGCCAGAGTTGTTAGCGAAGGACCAGATATCCCCCTCATTCCCTGT
GTAGAAAAAGATGGGCCCTTCGCCCATCTTCCAGAACTTATCTGTTGGAAGTAAATGAGT
TTCCATAAGGCCAGGGAAACGCAGGTAGGAACCCATGCGGTCGAGCCAGCACTCACCTGA
CACTAGGAACCGCTGGCCAAAGGTTTTGTTGCCGAAACTCTCAAAGTTGAAATGGTCCAT
GTATTGCTCAAAATAATTCTCATGAAAGTCAGGGTCTAGAACTCTGTCGGCTGAGGGCAG
GTGCAGAGACTCAGGAGCTGGTTGGGATCATCAGGGATCTAGGCGGGTCAGGAGGAAGGG
CAGCCAGTCTGTACTCACCTCTGGCCTGGAGGTTGCACAGTCCCAGTGACAGCAGCAGGA
CCAGGATCCAGGAGGGGACACCATGGTCCACAGGGTAACAAGGATGGAAGTTCATGCTTG
ATTCTGAGCCGGGCGCTGACTGTCATGTGATTTGGTCACATGACCGACACAACGGGCGGG
GCAGCATCACGTGATAGTCTGGCGGGGGCTGTCCTACTGTGGCTGGATTCTAGTTGGAGG
ATCAGCCTACTCTTCTTCAGTTTCCCGGTTCCTCCAAATTTCTGGGCTCCTACTTGTTTC
CACAGAGATGGATACTGTGGAGGTCCAGGAAGCAGAGAGA

>10 Get primers
CCTTCCACATGTGGGGTAGGCATAGGATGTTGAGACTGTAAGAGACATCTCTTTGGCCCT
CCTTGTATAGGGTGTCAATCGGCACAACAGGGTGGAGCCTTAGAGTAGGGTAAGATTAGG
ACTCTAGGTTCTCTCATGGGTCCAGATCTGTCATGAAGGGAGGTCAAGGACCCACCTCCC
TCCAAAGGCTATGGTGGGGGCATCATGGACCCCAAGGTCCTGCCGCAGGGCCTGGAGCAG
CACAGCAAAGTCGGCCAGCGCCTGCTCCACAGTCAGCAGCTGTGTATATCCCCGCTGTGT
GGACTGGACACCGAACGGAAGCGATTTCCCATAGTACCGCTGCAGAAAGCAGGAAGGGAT
GGCTAATCCACTCCTCGGTGCTCCCCACCTCCTTCAACTCAGGGACTGCCAGGAACTGTA
CAGGTACCCACGTGCTCAGCAAAGACAAGCAGGGCCTCCTGCTGGGCTGCCAGTTCCACC
ATGAAGCCAGAGTTGTTAGCGAAGGACCAGATATCCCCCTCATTCCCTGTGTAGAAAAAG
ATGGGCCCTTCGCCCATCTTCCAGAACTTATCTGTTGGAAGTAAATGAGTTTCCATAAGG
CCAGGGAAACGCAGGTAGGAACCCATGCGGTCGAGCCAGCACTCACCTGACACTAGGAAC
CGCTGGCCAAAGGTTTTGTTGCCGAAACTCTCAAAGTTGAAATGGTCCATGTATTGCTCA
AAATAATTCTCATGAAAGTCAGGGTCTAGAACTCTGTCGGCTGAGGGCAGGTGCAGAGAC
TCAGGAGCTGGTTGGGATCATCAGGGATCTAGGCGGGTCAGGAGGAAGGGCAGCCAGTCT
GTACTCACCTCTGGCCTGGAGGTTGCACAGTCCCAGTGACAGCAGCAGGACCAGGATCCA
GGAGGGGACACCATGGTCCACAGGGTAACAAGGATGGAAGTTCATGCTTGATTCTGAGCC
GGGCGCTGACTGTCATGTGATTTGGTCACATGACCGACACAACGGGCGGGGCAGCATCAC
GTGATAGTCTGGCGGGGGCTGTCCTACTGTGGCTGGATTCTAGTTGGAGGATCAGCCTAC
TCTTCTTCAGTTTCCCGGTTCCTCCAAATTTCTGGGCTCCTACTTGTTTCCACAGAGATG
GATACTGTGGAGGTCCAGGAAGCAGAGAGATGGCTAAGGCTCATCAGGACCGTATGATCT
CCCAAGTGTCCAGCTACTGAGTACCACAAGGTGATGGGTGGGAGGGTCCTCCCACGGAAG
GATACCGCAGTCCCTAGGGGTTGCAAGCCCCACATGTTCC

>11 Get primers
CTCTCATGGGTCCAGATCTGTCATGAAGGGAGGTCAAGGACCCACCTCCCTCCAAAGGCT
ATGGTGGGGGCATCATGGACCCCAAGGTCCTGCCGCAGGGCCTGGAGCAGCACAGCAAAG
TCGGCCAGCGCCTGCTCCACAGTCAGCAGCTGTGTATATCCCCGCTGTGTGGACTGGACA
CCGAACGGAAGCGATTTCCCATAGTACCGCTGCAGAAAGCAGGAAGGGATGGCTAATCCA
CTCCTCGGTGCTCCCCACCTCCTTCAACTCAGGGACTGCCAGGAACTGTACAGGTACCCA
CGTGCTCAGCAAAGACAAGCAGGGCCTCCTGCTGGGCTGCCAGTTCCACCATGAAGCCAG
AGTTGTTAGCGAAGGACCAGATATCCCCCTCATTCCCTGTGTAGAAAAAGATGGGCCCTT
CGCCCATCTTCCAGAACTTATCTGTTGGAAGTAAATGAGTTTCCATAAGGCCAGGGAAAC
GCAGGTAGGAACCCATGCGGTCGAGCCAGCACTCACCTGACACTAGGAACCGCTGGCCAA
AGGTTTTGTTGCCGAAACTCTCAAAGTTGAAATGGTCCATGTATTGCTCAAAATAATTCT
CATGAAAGTCAGGGTCTAGAACTCTGTCGGCTGAGGGCAGGTGCAGAGACTCAGGAGCTG
GTTGGGATCATCAGGGATCTAGGCGGGTCAGGAGGAAGGGCAGCCAGTCTGTACTCACCT
CTGGCCTGGAGGTTGCACAGTCCCAGTGACAGCAGCAGGACCAGGATCCAGGAGGGGACA
CCATGGTCCACAGGGTAACAAGGATGGAAGTTCATGCTTGATTCTGAGCCGGGCGCTGAC
TGTCATGTGATTTGGTCACATGACCGACACAACGGGCGGGGCAGCATCACGTGATAGTCT
GGCGGGGGCTGTCCTACTGTGGCTGGATTCTAGTTGGAGGATCAGCCTACTCTTCTTCAG
TTTCCCGGTTCCTCCAAATTTCTGGGCTCCTACTTGTTTCCACAGAGATGGATACTGTGG
AGGTCCAGGAAGCAGAGAGATGGCTAAGGCTCATCAGGACCGTATGATCTCCCAAGTGTC
CAGCTACTGAGTACCACAAGGTGATGGGTGGGAGGGTCCTCCCACGGAAGGATACCGCAG
TCCCTAGGGGTTGCAAGCCCCACATGTTCCACTGGCTGCTAGAGCTACCTACTCAATCAG
CCCTGGGCATCACCATCAGGTACTCGGCCAAAATGACCTCTCTGCTTCCAGTCCTCAGTT
CTGGTCAGCACCAGACAGGCCCATAATTACAGAGCCAGGG

>17 Get primers
CTCACCTTGACAGGGTTGGCAGGAAGGGGCCCTAGAAAGTCAGTAGGATACGGGTAGTCC
ATCATGGCGAGCACAGTAAATGCATTTCGGGCAAACCCAAAGAGCTGAGTCAGGTCCTTT
GGGCTGGAAAGTGATTGACAGGTACCAAAGTTCTGGCTGATGGTGTCATAGGCTGGGAAG
AGAGAGGCCAGGAGAAAAGGCTGAGGAAACTGCTGGCAAATGTGAAGGGCAAGAATGAAT
GCCCAAGGTGGGCAGCAGGTGAGGAAAGAGTCCCTCACCTCCCTGGAGGAACAAGTCTTT
GATTTGCTGAAAGGCATCCCGCACAGCCTGGGCGCACTTGGGACTCTGGCCATAAAAGTC
CTGGAGAAGAGACCAAGGTTGCTGCTGCCATTCTTGCACTGGCCTGGGGTACCCAAGTCC
CCTCACTCACCGCTGTGACATCTCGGAAGAATTGGTAGGAGTCCCCAAGGCCTGCAACAG
CTACAACAGGAGCGCTGGCTGCCAGTGCCCCAGCCACCAGGTGGGGGTACTTCATCCTCA
TGTAGGCACTCAGCATCCCCCCATAACTGGGAGTACAGAGCACAGATCATGGTTGTGGGA
AGCTGCCCACAACTCAGGCGAGCAGCCTCACTGTCCTCCAGGCTGAGGTGCTAGGCTGCT
CTTTCCCTGCTCAGAACGCCCAAGGGTGGGAAAGAAGGACCTGAAACTGTCAGGCCCACA
CACCCTGATCCCAGGGCCAAGGCAGATACAGCCTTCACTGGGAGAAGGCACCTGTGGGTG
CCCTGCCCTGACCCAGCAATGAAGACATTGCAGAGACAAAGTCAGAAGGAATTGTCCCAC
TAGTGGGAACAACATAGCATACACTGCCTATGAGGTCCACTCAAGGAGGGCTTCCAGAAG
GAGGTAAAGCTAGACCCCGCCCTTCCACATGTGGGGTAGGCATAGGATGTTGAGACTGTA
AGAGACATCTCTTTGGCCCTCCTTGTATAGGGTGTCAATCGGCACAACAGGGTGGAGCCT
TAGAGTAGGGTAAGATTAGGACTCTAGGTTCTCTCATGGGTCCAGATCTGTCATGAAGGG
AGGTCAAGGACCCACCTCCCTCCAAAGGCTATGGTGGGGGCATCATGGACCCCAAGGTCC
TGCCGCAGGGCCTGGAGCAGCACAGCAAAGTCGGCCAGCGCCTGCTCCACAGTCAGCAGC
TGTGTATATCCCCGCTGTGTGGACTGGACACCGAACGGAAGCGATTTCCC

>18 Get primers
GGAAAGTGATTGACAGGTACCAAAGTTCTGGCTGATGGTGTCATAGGCTGGGAAGAGAGA
GGCCAGGAGAAAAGGCTGAGGAAACTGCTGGCAAATGTGAAGGGCAAGAATGAATGCCCA
AGGTGGGCAGCAGGTGAGGAAAGAGTCCCTCACCTCCCTGGAGGAACAAGTCTTTGATTT
GCTGAAAGGCATCCCGCACAGCCTGGGCGCACTTGGGACTCTGGCCATAAAAGTCCTGGA
GAAGAGACCAAGGTTGCTGCTGCCATTCTTGCACTGGCCTGGGGTACCCAAGTCCCCTCA
CTCACCGCTGTGACATCTCGGAAGAATTGGTAGGAGTCCCCAAGGCCTGCAACAGCTACA
ACAGGAGCGCTGGCTGCCAGTGCCCCAGCCACCAGGTGGGGGTACTTCATCCTCATGTAG
GCACTCAGCATCCCCCCATAACTGGGAGTACAGAGCACAGATCATGGTTGTGGGAAGCTG
CCCACAACTCAGGCGAGCAGCCTCACTGTCCTCCAGGCTGAGGTGCTAGGCTGCTCTTTC
CCTGCTCAGAACGCCCAAGGGTGGGAAAGAAGGACCTGAAACTGTCAGGCCCACACACCC
TGATCCCAGGGCCAAGGCAGATACAGCCTTCACTGGGAGAAGGCACCTGTGGGTGCCCTG
CCCTGACCCAGCAATGAAGACATTGCAGAGACAAAGTCAGAAGGAATTGTCCCACTAGTG
GGAACAACATAGCATACACTGCCTATGAGGTCCACTCAAGGAGGGCTTCCAGAAGGAGGT
AAAGCTAGACCCCGCCCTTCCACATGTGGGGTAGGCATAGGATGTTGAGACTGTAAGAGA
CATCTCTTTGGCCCTCCTTGTATAGGGTGTCAATCGGCACAACAGGGTGGAGCCTTAGAG
TAGGGTAAGATTAGGACTCTAGGTTCTCTCATGGGTCCAGATCTGTCATGAAGGGAGGTC
AAGGACCCACCTCCCTCCAAAGGCTATGGTGGGGGCATCATGGACCCCAAGGTCCTGCCG
CAGGGCCTGGAGCAGCACAGCAAAGTCGGCCAGCGCCTGCTCCACAGTCAGCAGCTGTGT
ATATCCCCGCTGTGTGGACTGGACACCGAACGGAAGCGATTTCCCATAGTACCGCTGCAG
AAAGCAGGAAGGGATGGCTAATCCACTCCTCGGTGCTCCCCACCTCCTTCAACTCAGGGA
CTGCCAGGAACTGTACAGGTACCCACGTGCTCAGCAAAGACAAGCAGGGC

>19 Get primers
GGCAGCAGGTGAGGAAAGAGTCCCTCACCTCCCTGGAGGAACAAGTCTTTGATTTGCTGA
AAGGCATCCCGCACAGCCTGGGCGCACTTGGGACTCTGGCCATAAAAGTCCTGGAGAAGA
GACCAAGGTTGCTGCTGCCATTCTTGCACTGGCCTGGGGTACCCAAGTCCCCTCACTCAC
CGCTGTGACATCTCGGAAGAATTGGTAGGAGTCCCCAAGGCCTGCAACAGCTACAACAGG
AGCGCTGGCTGCCAGTGCCCCAGCCACCAGGTGGGGGTACTTCATCCTCATGTAGGCACT
CAGCATCCCCCCATAACTGGGAGTACAGAGCACAGATCATGGTTGTGGGAAGCTGCCCAC
AACTCAGGCGAGCAGCCTCACTGTCCTCCAGGCTGAGGTGCTAGGCTGCTCTTTCCCTGC
TCAGAACGCCCAAGGGTGGGAAAGAAGGACCTGAAACTGTCAGGCCCACACACCCTGATC
CCAGGGCCAAGGCAGATACAGCCTTCACTGGGAGAAGGCACCTGTGGGTGCCCTGCCCTG
ACCCAGCAATGAAGACATTGCAGAGACAAAGTCAGAAGGAATTGTCCCACTAGTGGGAAC
AACATAGCATACACTGCCTATGAGGTCCACTCAAGGAGGGCTTCCAGAAGGAGGTAAAGC
TAGACCCCGCCCTTCCACATGTGGGGTAGGCATAGGATGTTGAGACTGTAAGAGACATCT
CTTTGGCCCTCCTTGTATAGGGTGTCAATCGGCACAACAGGGTGGAGCCTTAGAGTAGGG
TAAGATTAGGACTCTAGGTTCTCTCATGGGTCCAGATCTGTCATGAAGGGAGGTCAAGGA
CCCACCTCCCTCCAAAGGCTATGGTGGGGGCATCATGGACCCCAAGGTCCTGCCGCAGGG
CCTGGAGCAGCACAGCAAAGTCGGCCAGCGCCTGCTCCACAGTCAGCAGCTGTGTATATC
CCCGCTGTGTGGACTGGACACCGAACGGAAGCGATTTCCCATAGTACCGCTGCAGAAAGC
AGGAAGGGATGGCTAATCCACTCCTCGGTGCTCCCCACCTCCTTCAACTCAGGGACTGCC
AGGAACTGTACAGGTACCCACGTGCTCAGCAAAGACAAGCAGGGCCTCCTGCTGGGCTGC
CAGTTCCACCATGAAGCCAGAGTTGTTAGCGAAGGACCAGATATCCCCCTCATTCCCTGT
GTAGAAAAAGATGGGCCCTTCGCCCATCTTCCAGAACTTATCTGTTGGAA

>20 Get primers
AGGTTGCTGCTGCCATTCTTGCACTGGCCTGGGGTACCCAAGTCCCCTCACTCACCGCTG
TGACATCTCGGAAGAATTGGTAGGAGTCCCCAAGGCCTGCAACAGCTACAACAGGAGCGC
TGGCTGCCAGTGCCCCAGCCACCAGGTGGGGGTACTTCATCCTCATGTAGGCACTCAGCA
TCCCCCCATAACTGGGAGTACAGAGCACAGATCATGGTTGTGGGAAGCTGCCCACAACTC
AGGCGAGCAGCCTCACTGTCCTCCAGGCTGAGGTGCTAGGCTGCTCTTTCCCTGCTCAGA
ACGCCCAAGGGTGGGAAAGAAGGACCTGAAACTGTCAGGCCCACACACCCTGATCCCAGG
GCCAAGGCAGATACAGCCTTCACTGGGAGAAGGCACCTGTGGGTGCCCTGCCCTGACCCA
GCAATGAAGACATTGCAGAGACAAAGTCAGAAGGAATTGTCCCACTAGTGGGAACAACAT
AGCATACACTGCCTATGAGGTCCACTCAAGGAGGGCTTCCAGAAGGAGGTAAAGCTAGAC
CCCGCCCTTCCACATGTGGGGTAGGCATAGGATGTTGAGACTGTAAGAGACATCTCTTTG
GCCCTCCTTGTATAGGGTGTCAATCGGCACAACAGGGTGGAGCCTTAGAGTAGGGTAAGA
TTAGGACTCTAGGTTCTCTCATGGGTCCAGATCTGTCATGAAGGGAGGTCAAGGACCCAC
CTCCCTCCAAAGGCTATGGTGGGGGCATCATGGACCCCAAGGTCCTGCCGCAGGGCCTGG
AGCAGCACAGCAAAGTCGGCCAGCGCCTGCTCCACAGTCAGCAGCTGTGTATATCCCCGC
TGTGTGGACTGGACACCGAACGGAAGCGATTTCCCATAGTACCGCTGCAGAAAGCAGGAA
GGGATGGCTAATCCACTCCTCGGTGCTCCCCACCTCCTTCAACTCAGGGACTGCCAGGAA
CTGTACAGGTACCCACGTGCTCAGCAAAGACAAGCAGGGCCTCCTGCTGGGCTGCCAGTT
CCACCATGAAGCCAGAGTTGTTAGCGAAGGACCAGATATCCCCCTCATTCCCTGTGTAGA
AAAAGATGGGCCCTTCGCCCATCTTCCAGAACTTATCTGTTGGAAGTAAATGAGTTTCCA
TAAGGCCAGGGAAACGCAGGTAGGAACCCATGCGGTCGAGCCAGCACTCACCTGACACTA
GGAACCGCTGGCCAAAGGTTTTGTTGCCGAAACTCTCAAAGTTGAAATGG

>21 Get primers
GCCAGTGCCCCAGCCACCAGGTGGGGGTACTTCATCCTCATGTAGGCACTCAGCATCCCC
CCATAACTGGGAGTACAGAGCACAGATCATGGTTGTGGGAAGCTGCCCACAACTCAGGCG
AGCAGCCTCACTGTCCTCCAGGCTGAGGTGCTAGGCTGCTCTTTCCCTGCTCAGAACGCC
CAAGGGTGGGAAAGAAGGACCTGAAACTGTCAGGCCCACACACCCTGATCCCAGGGCCAA
GGCAGATACAGCCTTCACTGGGAGAAGGCACCTGTGGGTGCCCTGCCCTGACCCAGCAAT
GAAGACATTGCAGAGACAAAGTCAGAAGGAATTGTCCCACTAGTGGGAACAACATAGCAT
ACACTGCCTATGAGGTCCACTCAAGGAGGGCTTCCAGAAGGAGGTAAAGCTAGACCCCGC
CCTTCCACATGTGGGGTAGGCATAGGATGTTGAGACTGTAAGAGACATCTCTTTGGCCCT
CCTTGTATAGGGTGTCAATCGGCACAACAGGGTGGAGCCTTAGAGTAGGGTAAGATTAGG
ACTCTAGGTTCTCTCATGGGTCCAGATCTGTCATGAAGGGAGGTCAAGGACCCACCTCCC
TCCAAAGGCTATGGTGGGGGCATCATGGACCCCAAGGTCCTGCCGCAGGGCCTGGAGCAG
CACAGCAAAGTCGGCCAGCGCCTGCTCCACAGTCAGCAGCTGTGTATATCCCCGCTGTGT
GGACTGGACACCGAACGGAAGCGATTTCCCATAGTACCGCTGCAGAAAGCAGGAAGGGAT
GGCTAATCCACTCCTCGGTGCTCCCCACCTCCTTCAACTCAGGGACTGCCAGGAACTGTA
CAGGTACCCACGTGCTCAGCAAAGACAAGCAGGGCCTCCTGCTGGGCTGCCAGTTCCACC
ATGAAGCCAGAGTTGTTAGCGAAGGACCAGATATCCCCCTCATTCCCTGTGTAGAAAAAG
ATGGGCCCTTCGCCCATCTTCCAGAACTTATCTGTTGGAAGTAAATGAGTTTCCATAAGG
CCAGGGAAACGCAGGTAGGAACCCATGCGGTCGAGCCAGCACTCACCTGACACTAGGAAC
CGCTGGCCAAAGGTTTTGTTGCCGAAACTCTCAAAGTTGAAATGGTCCATGTATTGCTCA
AAATAATTCTCATGAAAGTCAGGGTCTAGAACTCTGTCGGCTGAGGGCAGGTGCAGAGAC
TCAGGAGCTGGTTGGGATCATCAGGGATCTAGGCGGGTCAGGAGGAAGGG

>22 Get primers
CCTCACTGTCCTCCAGGCTGAGGTGCTAGGCTGCTCTTTCCCTGCTCAGAACGCCCAAGG
GTGGGAAAGAAGGACCTGAAACTGTCAGGCCCACACACCCTGATCCCAGGGCCAAGGCAG
ATACAGCCTTCACTGGGAGAAGGCACCTGTGGGTGCCCTGCCCTGACCCAGCAATGAAGA
CATTGCAGAGACAAAGTCAGAAGGAATTGTCCCACTAGTGGGAACAACATAGCATACACT
GCCTATGAGGTCCACTCAAGGAGGGCTTCCAGAAGGAGGTAAAGCTAGACCCCGCCCTTC
CACATGTGGGGTAGGCATAGGATGTTGAGACTGTAAGAGACATCTCTTTGGCCCTCCTTG
TATAGGGTGTCAATCGGCACAACAGGGTGGAGCCTTAGAGTAGGGTAAGATTAGGACTCT
AGGTTCTCTCATGGGTCCAGATCTGTCATGAAGGGAGGTCAAGGACCCACCTCCCTCCAA
AGGCTATGGTGGGGGCATCATGGACCCCAAGGTCCTGCCGCAGGGCCTGGAGCAGCACAG
CAAAGTCGGCCAGCGCCTGCTCCACAGTCAGCAGCTGTGTATATCCCCGCTGTGTGGACT
GGACACCGAACGGAAGCGATTTCCCATAGTACCGCTGCAGAAAGCAGGAAGGGATGGCTA
ATCCACTCCTCGGTGCTCCCCACCTCCTTCAACTCAGGGACTGCCAGGAACTGTACAGGT
ACCCACGTGCTCAGCAAAGACAAGCAGGGCCTCCTGCTGGGCTGCCAGTTCCACCATGAA
GCCAGAGTTGTTAGCGAAGGACCAGATATCCCCCTCATTCCCTGTGTAGAAAAAGATGGG
CCCTTCGCCCATCTTCCAGAACTTATCTGTTGGAAGTAAATGAGTTTCCATAAGGCCAGG
GAAACGCAGGTAGGAACCCATGCGGTCGAGCCAGCACTCACCTGACACTAGGAACCGCTG
GCCAAAGGTTTTGTTGCCGAAACTCTCAAAGTTGAAATGGTCCATGTATTGCTCAAAATA
ATTCTCATGAAAGTCAGGGTCTAGAACTCTGTCGGCTGAGGGCAGGTGCAGAGACTCAGG
AGCTGGTTGGGATCATCAGGGATCTAGGCGGGTCAGGAGGAAGGGCAGCCAGTCTGTACT
CACCTCTGGCCTGGAGGTTGCACAGTCCCAGTGACAGCAGCAGGACCAGGATCCAGGAGG
GGACACCATGGTCCACAGGGTAACAAGGATGGAAGTTCATGCTTGATTCT

>23 Get primers
GCCTTCACTGGGAGAAGGCACCTGTGGGTGCCCTGCCCTGACCCAGCAATGAAGACATTG
CAGAGACAAAGTCAGAAGGAATTGTCCCACTAGTGGGAACAACATAGCATACACTGCCTA
TGAGGTCCACTCAAGGAGGGCTTCCAGAAGGAGGTAAAGCTAGACCCCGCCCTTCCACAT
GTGGGGTAGGCATAGGATGTTGAGACTGTAAGAGACATCTCTTTGGCCCTCCTTGTATAG
GGTGTCAATCGGCACAACAGGGTGGAGCCTTAGAGTAGGGTAAGATTAGGACTCTAGGTT
CTCTCATGGGTCCAGATCTGTCATGAAGGGAGGTCAAGGACCCACCTCCCTCCAAAGGCT
ATGGTGGGGGCATCATGGACCCCAAGGTCCTGCCGCAGGGCCTGGAGCAGCACAGCAAAG
TCGGCCAGCGCCTGCTCCACAGTCAGCAGCTGTGTATATCCCCGCTGTGTGGACTGGACA
CCGAACGGAAGCGATTTCCCATAGTACCGCTGCAGAAAGCAGGAAGGGATGGCTAATCCA
CTCCTCGGTGCTCCCCACCTCCTTCAACTCAGGGACTGCCAGGAACTGTACAGGTACCCA
CGTGCTCAGCAAAGACAAGCAGGGCCTCCTGCTGGGCTGCCAGTTCCACCATGAAGCCAG
AGTTGTTAGCGAAGGACCAGATATCCCCCTCATTCCCTGTGTAGAAAAAGATGGGCCCTT
CGCCCATCTTCCAGAACTTATCTGTTGGAAGTAAATGAGTTTCCATAAGGCCAGGGAAAC
GCAGGTAGGAACCCATGCGGTCGAGCCAGCACTCACCTGACACTAGGAACCGCTGGCCAA
AGGTTTTGTTGCCGAAACTCTCAAAGTTGAAATGGTCCATGTATTGCTCAAAATAATTCT
CATGAAAGTCAGGGTCTAGAACTCTGTCGGCTGAGGGCAGGTGCAGAGACTCAGGAGCTG
GTTGGGATCATCAGGGATCTAGGCGGGTCAGGAGGAAGGGCAGCCAGTCTGTACTCACCT
CTGGCCTGGAGGTTGCACAGTCCCAGTGACAGCAGCAGGACCAGGATCCAGGAGGGGACA
CCATGGTCCACAGGGTAACAAGGATGGAAGTTCATGCTTGATTCTGAGCCGGGCGCTGAC
TGTCATGTGATTTGGTCACATGACCGACACAACGGGCGGGGCAGCATCACGTGATAGTCT
GGCGGGGGCTGTCCTACTGTGGCTGGATTCTAGTTGGAGGATCAGCCTAC

>24 Get primers
TCCACTCAAGGAGGGCTTCCAGAAGGAGGTAAAGCTAGACCCCGCCCTTCCACATGTGGG
GTAGGCATAGGATGTTGAGACTGTAAGAGACATCTCTTTGGCCCTCCTTGTATAGGGTGT
CAATCGGCACAACAGGGTGGAGCCTTAGAGTAGGGTAAGATTAGGACTCTAGGTTCTCTC
ATGGGTCCAGATCTGTCATGAAGGGAGGTCAAGGACCCACCTCCCTCCAAAGGCTATGGT
GGGGGCATCATGGACCCCAAGGTCCTGCCGCAGGGCCTGGAGCAGCACAGCAAAGTCGGC
CAGCGCCTGCTCCACAGTCAGCAGCTGTGTATATCCCCGCTGTGTGGACTGGACACCGAA
CGGAAGCGATTTCCCATAGTACCGCTGCAGAAAGCAGGAAGGGATGGCTAATCCACTCCT
CGGTGCTCCCCACCTCCTTCAACTCAGGGACTGCCAGGAACTGTACAGGTACCCACGTGC
TCAGCAAAGACAAGCAGGGCCTCCTGCTGGGCTGCCAGTTCCACCATGAAGCCAGAGTTG
TTAGCGAAGGACCAGATATCCCCCTCATTCCCTGTGTAGAAAAAGATGGGCCCTTCGCCC
ATCTTCCAGAACTTATCTGTTGGAAGTAAATGAGTTTCCATAAGGCCAGGGAAACGCAGG
TAGGAACCCATGCGGTCGAGCCAGCACTCACCTGACACTAGGAACCGCTGGCCAAAGGTT
TTGTTGCCGAAACTCTCAAAGTTGAAATGGTCCATGTATTGCTCAAAATAATTCTCATGA
AAGTCAGGGTCTAGAACTCTGTCGGCTGAGGGCAGGTGCAGAGACTCAGGAGCTGGTTGG
GATCATCAGGGATCTAGGCGGGTCAGGAGGAAGGGCAGCCAGTCTGTACTCACCTCTGGC
CTGGAGGTTGCACAGTCCCAGTGACAGCAGCAGGACCAGGATCCAGGAGGGGACACCATG
GTCCACAGGGTAACAAGGATGGAAGTTCATGCTTGATTCTGAGCCGGGCGCTGACTGTCA
TGTGATTTGGTCACATGACCGACACAACGGGCGGGGCAGCATCACGTGATAGTCTGGCGG
GGGCTGTCCTACTGTGGCTGGATTCTAGTTGGAGGATCAGCCTACTCTTCTTCAGTTTCC
CGGTTCCTCCAAATTTCTGGGCTCCTACTTGTTTCCACAGAGATGGATACTGTGGAGGTC
CAGGAAGCAGAGAGATGGCTAAGGCTCATCAGGACCGTATGATCTCCCAA

>25 Get primers
GGCACAACAGGGTGGAGCCTTAGAGTAGGGTAAGATTAGGACTCTAGGTTCTCTCATGGG
TCCAGATCTGTCATGAAGGGAGGTCAAGGACCCACCTCCCTCCAAAGGCTATGGTGGGGG
CATCATGGACCCCAAGGTCCTGCCGCAGGGCCTGGAGCAGCACAGCAAAGTCGGCCAGCG
CCTGCTCCACAGTCAGCAGCTGTGTATATCCCCGCTGTGTGGACTGGACACCGAACGGAA
GCGATTTCCCATAGTACCGCTGCAGAAAGCAGGAAGGGATGGCTAATCCACTCCTCGGTG
CTCCCCACCTCCTTCAACTCAGGGACTGCCAGGAACTGTACAGGTACCCACGTGCTCAGC
AAAGACAAGCAGGGCCTCCTGCTGGGCTGCCAGTTCCACCATGAAGCCAGAGTTGTTAGC
GAAGGACCAGATATCCCCCTCATTCCCTGTGTAGAAAAAGATGGGCCCTTCGCCCATCTT
CCAGAACTTATCTGTTGGAAGTAAATGAGTTTCCATAAGGCCAGGGAAACGCAGGTAGGA
ACCCATGCGGTCGAGCCAGCACTCACCTGACACTAGGAACCGCTGGCCAAAGGTTTTGTT
GCCGAAACTCTCAAAGTTGAAATGGTCCATGTATTGCTCAAAATAATTCTCATGAAAGTC
AGGGTCTAGAACTCTGTCGGCTGAGGGCAGGTGCAGAGACTCAGGAGCTGGTTGGGATCA
TCAGGGATCTAGGCGGGTCAGGAGGAAGGGCAGCCAGTCTGTACTCACCTCTGGCCTGGA
GGTTGCACAGTCCCAGTGACAGCAGCAGGACCAGGATCCAGGAGGGGACACCATGGTCCA
CAGGGTAACAAGGATGGAAGTTCATGCTTGATTCTGAGCCGGGCGCTGACTGTCATGTGA
TTTGGTCACATGACCGACACAACGGGCGGGGCAGCATCACGTGATAGTCTGGCGGGGGCT
GTCCTACTGTGGCTGGATTCTAGTTGGAGGATCAGCCTACTCTTCTTCAGTTTCCCGGTT
CCTCCAAATTTCTGGGCTCCTACTTGTTTCCACAGAGATGGATACTGTGGAGGTCCAGGA
AGCAGAGAGATGGCTAAGGCTCATCAGGACCGTATGATCTCCCAAGTGTCCAGCTACTGA
GTACCACAAGGTGATGGGTGGGAGGGTCCTCCCACGGAAGGATACCGCAGTCCCTAGGGG
TTGCAAGCCCCACATGTTCCACTGGCTGCTAGAGCTACCTACTCAATCAG

>26 Get primers
TGGACCCCAAGGTCCTGCCGCAGGGCCTGGAGCAGCACAGCAAAGTCGGCCAGCGCCTGC
TCCACAGTCAGCAGCTGTGTATATCCCCGCTGTGTGGACTGGACACCGAACGGAAGCGAT
TTCCCATAGTACCGCTGCAGAAAGCAGGAAGGGATGGCTAATCCACTCCTCGGTGCTCCC
CACCTCCTTCAACTCAGGGACTGCCAGGAACTGTACAGGTACCCACGTGCTCAGCAAAGA
CAAGCAGGGCCTCCTGCTGGGCTGCCAGTTCCACCATGAAGCCAGAGTTGTTAGCGAAGG
ACCAGATATCCCCCTCATTCCCTGTGTAGAAAAAGATGGGCCCTTCGCCCATCTTCCAGA
ACTTATCTGTTGGAAGTAAATGAGTTTCCATAAGGCCAGGGAAACGCAGGTAGGAACCCA
TGCGGTCGAGCCAGCACTCACCTGACACTAGGAACCGCTGGCCAAAGGTTTTGTTGCCGA
AACTCTCAAAGTTGAAATGGTCCATGTATTGCTCAAAATAATTCTCATGAAAGTCAGGGT
CTAGAACTCTGTCGGCTGAGGGCAGGTGCAGAGACTCAGGAGCTGGTTGGGATCATCAGG
GATCTAGGCGGGTCAGGAGGAAGGGCAGCCAGTCTGTACTCACCTCTGGCCTGGAGGTTG
CACAGTCCCAGTGACAGCAGCAGGACCAGGATCCAGGAGGGGACACCATGGTCCACAGGG
TAACAAGGATGGAAGTTCATGCTTGATTCTGAGCCGGGCGCTGACTGTCATGTGATTTGG
TCACATGACCGACACAACGGGCGGGGCAGCATCACGTGATAGTCTGGCGGGGGCTGTCCT
ACTGTGGCTGGATTCTAGTTGGAGGATCAGCCTACTCTTCTTCAGTTTCCCGGTTCCTCC
AAATTTCTGGGCTCCTACTTGTTTCCACAGAGATGGATACTGTGGAGGTCCAGGAAGCAG
AGAGATGGCTAAGGCTCATCAGGACCGTATGATCTCCCAAGTGTCCAGCTACTGAGTACC
ACAAGGTGATGGGTGGGAGGGTCCTCCCACGGAAGGATACCGCAGTCCCTAGGGGTTGCA
AGCCCCACATGTTCCACTGGCTGCTAGAGCTACCTACTCAATCAGCCCTGGGCATCACCA
TCAGGTACTCGGCCAAAATGACCTCTCTGCTTCCAGTCCTCAGTTCTGGTCAGCACCAGA
CAGGCCCATAATTACAGAGCCAGGGAAACTGGAACATTTGTCTCCCCTTA

>33 Get primers
CAGGTCCTTTGGGCTGGAAAGTGATTGACAGGTACCAAAGTTCTGGCTGATGGTGTCATA
GGCTGGGAAGAGAGAGGCCAGGAGAAAAGGCTGAGGAAACTGCTGGCAAATGTGAAGGGC
AAGAATGAATGCCCAAGGTGGGCAGCAGGTGAGGAAAGAGTCCCTCACCTCCCTGGAGGA
ACAAGTCTTTGATTTGCTGAAAGGCATCCCGCACAGCCTGGGCGCACTTGGGACTCTGGC
CATAAAAGTCCTGGAGAAGAGACCAAGGTTGCTGCTGCCATTCTTGCACTGGCCTGGGGT
ACCCAAGTCCCCTCACTCACCGCTGTGACATCTCGGAAGAATTGGTAGGAGTCCCCAAGG
CCTGCAACAGCTACAACAGGAGCGCTGGCTGCCAGTGCCCCAGCCACCAGGTGGGGGTAC
TTCATCCTCATGTAGGCACTCAGCATCCCCCCATAACTGGGAGTACAGAGCACAGATCAT
GGTTGTGGGAAGCTGCCCACAACTCAGGCGAGCAGCCTCACTGTCCTCCAGGCTGAGGTG
CTAGGCTGCTCTTTCCCTGCTCAGAACGCCCAAGGGTGGGAAAGAAGGACCTGAAACTGT
CAGGCCCACACACCCTGATCCCAGGGCCAAGGCAGATACAGCCTTCACTGGGAGAAGGCA
CCTGTGGGTGCCCTGCCCTGACCCAGCAATGAAGACATTGCAGAGACAAAGTCAGAAGGA
ATTGTCCCACTAGTGGGAACAACATAGCATACACTGCCTATGAGGTCCACTCAAGGAGGG
CTTCCAGAAGGAGGTAAAGCTAGACCCCGCCCTTCCACATGTGGGGTAGGCATAGGATGT
TGAGACTGTAAGAGACATCTCTTTGGCCCTCCTTGTATAGGGTGTCAATCGGCACAACAG
GGTGGAGCCTTAGAGTAGGGTAAGATTAGGACTCTAGGTTCTCTCATGGGTCCAGATCTG
TCATGAAGGGAGGTCAAGGACCCACCTCCCTCCAAAGGCTATGGTGGGGGCATCATGGAC
CCCAAGGTCCTGCCGCAGGGCCTGGAGCAGCACAGCAAAGTCGGCCAGCGCCTGCTCCAC
AGTCAGCAGCTGTGTATATCCCCGCTGTGTGGACTGGACACCGAACGGAAGCGATTTCCC
ATAGTACCGCTGCAGAAAGCAGGAAGGGATGGCTAATCCACTCCTCGGTGCTCCCCACCT

>34 Get primers
AAGAATGAATGCCCAAGGTGGGCAGCAGGTGAGGAAAGAGTCCCTCACCTCCCTGGAGGA
ACAAGTCTTTGATTTGCTGAAAGGCATCCCGCACAGCCTGGGCGCACTTGGGACTCTGGC
CATAAAAGTCCTGGAGAAGAGACCAAGGTTGCTGCTGCCATTCTTGCACTGGCCTGGGGT
ACCCAAGTCCCCTCACTCACCGCTGTGACATCTCGGAAGAATTGGTAGGAGTCCCCAAGG
CCTGCAACAGCTACAACAGGAGCGCTGGCTGCCAGTGCCCCAGCCACCAGGTGGGGGTAC
TTCATCCTCATGTAGGCACTCAGCATCCCCCCATAACTGGGAGTACAGAGCACAGATCAT
GGTTGTGGGAAGCTGCCCACAACTCAGGCGAGCAGCCTCACTGTCCTCCAGGCTGAGGTG
CTAGGCTGCTCTTTCCCTGCTCAGAACGCCCAAGGGTGGGAAAGAAGGACCTGAAACTGT
CAGGCCCACACACCCTGATCCCAGGGCCAAGGCAGATACAGCCTTCACTGGGAGAAGGCA
CCTGTGGGTGCCCTGCCCTGACCCAGCAATGAAGACATTGCAGAGACAAAGTCAGAAGGA
ATTGTCCCACTAGTGGGAACAACATAGCATACACTGCCTATGAGGTCCACTCAAGGAGGG
CTTCCAGAAGGAGGTAAAGCTAGACCCCGCCCTTCCACATGTGGGGTAGGCATAGGATGT
TGAGACTGTAAGAGACATCTCTTTGGCCCTCCTTGTATAGGGTGTCAATCGGCACAACAG
GGTGGAGCCTTAGAGTAGGGTAAGATTAGGACTCTAGGTTCTCTCATGGGTCCAGATCTG
TCATGAAGGGAGGTCAAGGACCCACCTCCCTCCAAAGGCTATGGTGGGGGCATCATGGAC
CCCAAGGTCCTGCCGCAGGGCCTGGAGCAGCACAGCAAAGTCGGCCAGCGCCTGCTCCAC
AGTCAGCAGCTGTGTATATCCCCGCTGTGTGGACTGGACACCGAACGGAAGCGATTTCCC
ATAGTACCGCTGCAGAAAGCAGGAAGGGATGGCTAATCCACTCCTCGGTGCTCCCCACCT
CCTTCAACTCAGGGACTGCCAGGAACTGTACAGGTACCCACGTGCTCAGCAAAGACAAGC
AGGGCCTCCTGCTGGGCTGCCAGTTCCACCATGAAGCCAGAGTTGTTAGCGAAGGACCAG

>35 Get primers
CATAAAAGTCCTGGAGAAGAGACCAAGGTTGCTGCTGCCATTCTTGCACTGGCCTGGGGT
ACCCAAGTCCCCTCACTCACCGCTGTGACATCTCGGAAGAATTGGTAGGAGTCCCCAAGG
CCTGCAACAGCTACAACAGGAGCGCTGGCTGCCAGTGCCCCAGCCACCAGGTGGGGGTAC
TTCATCCTCATGTAGGCACTCAGCATCCCCCCATAACTGGGAGTACAGAGCACAGATCAT
GGTTGTGGGAAGCTGCCCACAACTCAGGCGAGCAGCCTCACTGTCCTCCAGGCTGAGGTG
CTAGGCTGCTCTTTCCCTGCTCAGAACGCCCAAGGGTGGGAAAGAAGGACCTGAAACTGT
CAGGCCCACACACCCTGATCCCAGGGCCAAGGCAGATACAGCCTTCACTGGGAGAAGGCA
CCTGTGGGTGCCCTGCCCTGACCCAGCAATGAAGACATTGCAGAGACAAAGTCAGAAGGA
ATTGTCCCACTAGTGGGAACAACATAGCATACACTGCCTATGAGGTCCACTCAAGGAGGG
CTTCCAGAAGGAGGTAAAGCTAGACCCCGCCCTTCCACATGTGGGGTAGGCATAGGATGT
TGAGACTGTAAGAGACATCTCTTTGGCCCTCCTTGTATAGGGTGTCAATCGGCACAACAG
GGTGGAGCCTTAGAGTAGGGTAAGATTAGGACTCTAGGTTCTCTCATGGGTCCAGATCTG
TCATGAAGGGAGGTCAAGGACCCACCTCCCTCCAAAGGCTATGGTGGGGGCATCATGGAC
CCCAAGGTCCTGCCGCAGGGCCTGGAGCAGCACAGCAAAGTCGGCCAGCGCCTGCTCCAC
AGTCAGCAGCTGTGTATATCCCCGCTGTGTGGACTGGACACCGAACGGAAGCGATTTCCC
ATAGTACCGCTGCAGAAAGCAGGAAGGGATGGCTAATCCACTCCTCGGTGCTCCCCACCT
CCTTCAACTCAGGGACTGCCAGGAACTGTACAGGTACCCACGTGCTCAGCAAAGACAAGC
AGGGCCTCCTGCTGGGCTGCCAGTTCCACCATGAAGCCAGAGTTGTTAGCGAAGGACCAG
ATATCCCCCTCATTCCCTGTGTAGAAAAAGATGGGCCCTTCGCCCATCTTCCAGAACTTA
TCTGTTGGAAGTAAATGAGTTTCCATAAGGCCAGGGAAACGCAGGTAGGAACCCATGCGG

>36 Get primers
CCTGCAACAGCTACAACAGGAGCGCTGGCTGCCAGTGCCCCAGCCACCAGGTGGGGGTAC
TTCATCCTCATGTAGGCACTCAGCATCCCCCCATAACTGGGAGTACAGAGCACAGATCAT
GGTTGTGGGAAGCTGCCCACAACTCAGGCGAGCAGCCTCACTGTCCTCCAGGCTGAGGTG
CTAGGCTGCTCTTTCCCTGCTCAGAACGCCCAAGGGTGGGAAAGAAGGACCTGAAACTGT
CAGGCCCACACACCCTGATCCCAGGGCCAAGGCAGATACAGCCTTCACTGGGAGAAGGCA
CCTGTGGGTGCCCTGCCCTGACCCAGCAATGAAGACATTGCAGAGACAAAGTCAGAAGGA
ATTGTCCCACTAGTGGGAACAACATAGCATACACTGCCTATGAGGTCCACTCAAGGAGGG
CTTCCAGAAGGAGGTAAAGCTAGACCCCGCCCTTCCACATGTGGGGTAGGCATAGGATGT
TGAGACTGTAAGAGACATCTCTTTGGCCCTCCTTGTATAGGGTGTCAATCGGCACAACAG
GGTGGAGCCTTAGAGTAGGGTAAGATTAGGACTCTAGGTTCTCTCATGGGTCCAGATCTG
TCATGAAGGGAGGTCAAGGACCCACCTCCCTCCAAAGGCTATGGTGGGGGCATCATGGAC
CCCAAGGTCCTGCCGCAGGGCCTGGAGCAGCACAGCAAAGTCGGCCAGCGCCTGCTCCAC
AGTCAGCAGCTGTGTATATCCCCGCTGTGTGGACTGGACACCGAACGGAAGCGATTTCCC
ATAGTACCGCTGCAGAAAGCAGGAAGGGATGGCTAATCCACTCCTCGGTGCTCCCCACCT
CCTTCAACTCAGGGACTGCCAGGAACTGTACAGGTACCCACGTGCTCAGCAAAGACAAGC
AGGGCCTCCTGCTGGGCTGCCAGTTCCACCATGAAGCCAGAGTTGTTAGCGAAGGACCAG
ATATCCCCCTCATTCCCTGTGTAGAAAAAGATGGGCCCTTCGCCCATCTTCCAGAACTTA
TCTGTTGGAAGTAAATGAGTTTCCATAAGGCCAGGGAAACGCAGGTAGGAACCCATGCGG
TCGAGCCAGCACTCACCTGACACTAGGAACCGCTGGCCAAAGGTTTTGTTGCCGAAACTC
TCAAAGTTGAAATGGTCCATGTATTGCTCAAAATAATTCTCATGAAAGTCAGGGTCTAGA

>37 Get primers
GGTTGTGGGAAGCTGCCCACAACTCAGGCGAGCAGCCTCACTGTCCTCCAGGCTGAGGTG
CTAGGCTGCTCTTTCCCTGCTCAGAACGCCCAAGGGTGGGAAAGAAGGACCTGAAACTGT
CAGGCCCACACACCCTGATCCCAGGGCCAAGGCAGATACAGCCTTCACTGGGAGAAGGCA
CCTGTGGGTGCCCTGCCCTGACCCAGCAATGAAGACATTGCAGAGACAAAGTCAGAAGGA
ATTGTCCCACTAGTGGGAACAACATAGCATACACTGCCTATGAGGTCCACTCAAGGAGGG
CTTCCAGAAGGAGGTAAAGCTAGACCCCGCCCTTCCACATGTGGGGTAGGCATAGGATGT
TGAGACTGTAAGAGACATCTCTTTGGCCCTCCTTGTATAGGGTGTCAATCGGCACAACAG
GGTGGAGCCTTAGAGTAGGGTAAGATTAGGACTCTAGGTTCTCTCATGGGTCCAGATCTG
TCATGAAGGGAGGTCAAGGACCCACCTCCCTCCAAAGGCTATGGTGGGGGCATCATGGAC
CCCAAGGTCCTGCCGCAGGGCCTGGAGCAGCACAGCAAAGTCGGCCAGCGCCTGCTCCAC
AGTCAGCAGCTGTGTATATCCCCGCTGTGTGGACTGGACACCGAACGGAAGCGATTTCCC
ATAGTACCGCTGCAGAAAGCAGGAAGGGATGGCTAATCCACTCCTCGGTGCTCCCCACCT
CCTTCAACTCAGGGACTGCCAGGAACTGTACAGGTACCCACGTGCTCAGCAAAGACAAGC
AGGGCCTCCTGCTGGGCTGCCAGTTCCACCATGAAGCCAGAGTTGTTAGCGAAGGACCAG
ATATCCCCCTCATTCCCTGTGTAGAAAAAGATGGGCCCTTCGCCCATCTTCCAGAACTTA
TCTGTTGGAAGTAAATGAGTTTCCATAAGGCCAGGGAAACGCAGGTAGGAACCCATGCGG
TCGAGCCAGCACTCACCTGACACTAGGAACCGCTGGCCAAAGGTTTTGTTGCCGAAACTC
TCAAAGTTGAAATGGTCCATGTATTGCTCAAAATAATTCTCATGAAAGTCAGGGTCTAGA
ACTCTGTCGGCTGAGGGCAGGTGCAGAGACTCAGGAGCTGGTTGGGATCATCAGGGATCT
AGGCGGGTCAGGAGGAAGGGCAGCCAGTCTGTACTCACCTCTGGCCTGGAGGTTGCACAG

>38 Get primers
CAGGCCCACACACCCTGATCCCAGGGCCAAGGCAGATACAGCCTTCACTGGGAGAAGGCA
CCTGTGGGTGCCCTGCCCTGACCCAGCAATGAAGACATTGCAGAGACAAAGTCAGAAGGA
ATTGTCCCACTAGTGGGAACAACATAGCATACACTGCCTATGAGGTCCACTCAAGGAGGG
CTTCCAGAAGGAGGTAAAGCTAGACCCCGCCCTTCCACATGTGGGGTAGGCATAGGATGT
TGAGACTGTAAGAGACATCTCTTTGGCCCTCCTTGTATAGGGTGTCAATCGGCACAACAG
GGTGGAGCCTTAGAGTAGGGTAAGATTAGGACTCTAGGTTCTCTCATGGGTCCAGATCTG
TCATGAAGGGAGGTCAAGGACCCACCTCCCTCCAAAGGCTATGGTGGGGGCATCATGGAC
CCCAAGGTCCTGCCGCAGGGCCTGGAGCAGCACAGCAAAGTCGGCCAGCGCCTGCTCCAC
AGTCAGCAGCTGTGTATATCCCCGCTGTGTGGACTGGACACCGAACGGAAGCGATTTCCC
ATAGTACCGCTGCAGAAAGCAGGAAGGGATGGCTAATCCACTCCTCGGTGCTCCCCACCT
CCTTCAACTCAGGGACTGCCAGGAACTGTACAGGTACCCACGTGCTCAGCAAAGACAAGC
AGGGCCTCCTGCTGGGCTGCCAGTTCCACCATGAAGCCAGAGTTGTTAGCGAAGGACCAG
ATATCCCCCTCATTCCCTGTGTAGAAAAAGATGGGCCCTTCGCCCATCTTCCAGAACTTA
TCTGTTGGAAGTAAATGAGTTTCCATAAGGCCAGGGAAACGCAGGTAGGAACCCATGCGG
TCGAGCCAGCACTCACCTGACACTAGGAACCGCTGGCCAAAGGTTTTGTTGCCGAAACTC
TCAAAGTTGAAATGGTCCATGTATTGCTCAAAATAATTCTCATGAAAGTCAGGGTCTAGA
ACTCTGTCGGCTGAGGGCAGGTGCAGAGACTCAGGAGCTGGTTGGGATCATCAGGGATCT
AGGCGGGTCAGGAGGAAGGGCAGCCAGTCTGTACTCACCTCTGGCCTGGAGGTTGCACAG
TCCCAGTGACAGCAGCAGGACCAGGATCCAGGAGGGGACACCATGGTCCACAGGGTAACA
AGGATGGAAGTTCATGCTTGATTCTGAGCCGGGCGCTGACTGTCATGTGATTTGGTCACA

>39 Get primers
ATTGTCCCACTAGTGGGAACAACATAGCATACACTGCCTATGAGGTCCACTCAAGGAGGG
CTTCCAGAAGGAGGTAAAGCTAGACCCCGCCCTTCCACATGTGGGGTAGGCATAGGATGT
TGAGACTGTAAGAGACATCTCTTTGGCCCTCCTTGTATAGGGTGTCAATCGGCACAACAG
GGTGGAGCCTTAGAGTAGGGTAAGATTAGGACTCTAGGTTCTCTCATGGGTCCAGATCTG
TCATGAAGGGAGGTCAAGGACCCACCTCCCTCCAAAGGCTATGGTGGGGGCATCATGGAC
CCCAAGGTCCTGCCGCAGGGCCTGGAGCAGCACAGCAAAGTCGGCCAGCGCCTGCTCCAC
AGTCAGCAGCTGTGTATATCCCCGCTGTGTGGACTGGACACCGAACGGAAGCGATTTCCC
ATAGTACCGCTGCAGAAAGCAGGAAGGGATGGCTAATCCACTCCTCGGTGCTCCCCACCT
CCTTCAACTCAGGGACTGCCAGGAACTGTACAGGTACCCACGTGCTCAGCAAAGACAAGC
AGGGCCTCCTGCTGGGCTGCCAGTTCCACCATGAAGCCAGAGTTGTTAGCGAAGGACCAG
ATATCCCCCTCATTCCCTGTGTAGAAAAAGATGGGCCCTTCGCCCATCTTCCAGAACTTA
TCTGTTGGAAGTAAATGAGTTTCCATAAGGCCAGGGAAACGCAGGTAGGAACCCATGCGG
TCGAGCCAGCACTCACCTGACACTAGGAACCGCTGGCCAAAGGTTTTGTTGCCGAAACTC
TCAAAGTTGAAATGGTCCATGTATTGCTCAAAATAATTCTCATGAAAGTCAGGGTCTAGA
ACTCTGTCGGCTGAGGGCAGGTGCAGAGACTCAGGAGCTGGTTGGGATCATCAGGGATCT
AGGCGGGTCAGGAGGAAGGGCAGCCAGTCTGTACTCACCTCTGGCCTGGAGGTTGCACAG
TCCCAGTGACAGCAGCAGGACCAGGATCCAGGAGGGGACACCATGGTCCACAGGGTAACA
AGGATGGAAGTTCATGCTTGATTCTGAGCCGGGCGCTGACTGTCATGTGATTTGGTCACA
TGACCGACACAACGGGCGGGGCAGCATCACGTGATAGTCTGGCGGGGGCTGTCCTACTGT
GGCTGGATTCTAGTTGGAGGATCAGCCTACTCTTCTTCAGTTTCCCGGTTCCTCCAAATT

>40 Get primers
TGAGACTGTAAGAGACATCTCTTTGGCCCTCCTTGTATAGGGTGTCAATCGGCACAACAG
GGTGGAGCCTTAGAGTAGGGTAAGATTAGGACTCTAGGTTCTCTCATGGGTCCAGATCTG
TCATGAAGGGAGGTCAAGGACCCACCTCCCTCCAAAGGCTATGGTGGGGGCATCATGGAC
CCCAAGGTCCTGCCGCAGGGCCTGGAGCAGCACAGCAAAGTCGGCCAGCGCCTGCTCCAC
AGTCAGCAGCTGTGTATATCCCCGCTGTGTGGACTGGACACCGAACGGAAGCGATTTCCC
ATAGTACCGCTGCAGAAAGCAGGAAGGGATGGCTAATCCACTCCTCGGTGCTCCCCACCT
CCTTCAACTCAGGGACTGCCAGGAACTGTACAGGTACCCACGTGCTCAGCAAAGACAAGC
AGGGCCTCCTGCTGGGCTGCCAGTTCCACCATGAAGCCAGAGTTGTTAGCGAAGGACCAG
ATATCCCCCTCATTCCCTGTGTAGAAAAAGATGGGCCCTTCGCCCATCTTCCAGAACTTA
TCTGTTGGAAGTAAATGAGTTTCCATAAGGCCAGGGAAACGCAGGTAGGAACCCATGCGG
TCGAGCCAGCACTCACCTGACACTAGGAACCGCTGGCCAAAGGTTTTGTTGCCGAAACTC
TCAAAGTTGAAATGGTCCATGTATTGCTCAAAATAATTCTCATGAAAGTCAGGGTCTAGA
ACTCTGTCGGCTGAGGGCAGGTGCAGAGACTCAGGAGCTGGTTGGGATCATCAGGGATCT
AGGCGGGTCAGGAGGAAGGGCAGCCAGTCTGTACTCACCTCTGGCCTGGAGGTTGCACAG
TCCCAGTGACAGCAGCAGGACCAGGATCCAGGAGGGGACACCATGGTCCACAGGGTAACA
AGGATGGAAGTTCATGCTTGATTCTGAGCCGGGCGCTGACTGTCATGTGATTTGGTCACA
TGACCGACACAACGGGCGGGGCAGCATCACGTGATAGTCTGGCGGGGGCTGTCCTACTGT
GGCTGGATTCTAGTTGGAGGATCAGCCTACTCTTCTTCAGTTTCCCGGTTCCTCCAAATT
TCTGGGCTCCTACTTGTTTCCACAGAGATGGATACTGTGGAGGTCCAGGAAGCAGAGAGA
TGGCTAAGGCTCATCAGGACCGTATGATCTCCCAAGTGTCCAGCTACTGAGTACCACAAG

>41 Get primers
TCATGAAGGGAGGTCAAGGACCCACCTCCCTCCAAAGGCTATGGTGGGGGCATCATGGAC
CCCAAGGTCCTGCCGCAGGGCCTGGAGCAGCACAGCAAAGTCGGCCAGCGCCTGCTCCAC
AGTCAGCAGCTGTGTATATCCCCGCTGTGTGGACTGGACACCGAACGGAAGCGATTTCCC
ATAGTACCGCTGCAGAAAGCAGGAAGGGATGGCTAATCCACTCCTCGGTGCTCCCCACCT
CCTTCAACTCAGGGACTGCCAGGAACTGTACAGGTACCCACGTGCTCAGCAAAGACAAGC
AGGGCCTCCTGCTGGGCTGCCAGTTCCACCATGAAGCCAGAGTTGTTAGCGAAGGACCAG
ATATCCCCCTCATTCCCTGTGTAGAAAAAGATGGGCCCTTCGCCCATCTTCCAGAACTTA
TCTGTTGGAAGTAAATGAGTTTCCATAAGGCCAGGGAAACGCAGGTAGGAACCCATGCGG
TCGAGCCAGCACTCACCTGACACTAGGAACCGCTGGCCAAAGGTTTTGTTGCCGAAACTC
TCAAAGTTGAAATGGTCCATGTATTGCTCAAAATAATTCTCATGAAAGTCAGGGTCTAGA
ACTCTGTCGGCTGAGGGCAGGTGCAGAGACTCAGGAGCTGGTTGGGATCATCAGGGATCT
AGGCGGGTCAGGAGGAAGGGCAGCCAGTCTGTACTCACCTCTGGCCTGGAGGTTGCACAG
TCCCAGTGACAGCAGCAGGACCAGGATCCAGGAGGGGACACCATGGTCCACAGGGTAACA
AGGATGGAAGTTCATGCTTGATTCTGAGCCGGGCGCTGACTGTCATGTGATTTGGTCACA
TGACCGACACAACGGGCGGGGCAGCATCACGTGATAGTCTGGCGGGGGCTGTCCTACTGT
GGCTGGATTCTAGTTGGAGGATCAGCCTACTCTTCTTCAGTTTCCCGGTTCCTCCAAATT
TCTGGGCTCCTACTTGTTTCCACAGAGATGGATACTGTGGAGGTCCAGGAAGCAGAGAGA
TGGCTAAGGCTCATCAGGACCGTATGATCTCCCAAGTGTCCAGCTACTGAGTACCACAAG
GTGATGGGTGGGAGGGTCCTCCCACGGAAGGATACCGCAGTCCCTAGGGGTTGCAAGCCC
CACATGTTCCACTGGCTGCTAGAGCTACCTACTCAATCAGCCCTGGGCATCACCATCAGG

>49 Get primers
CCCAAAGAGCTGAGTCAGGTCCTTTGGGCTGGAAAGTGATTGACAGGTACCAAAGTTCTG
GCTGATGGTGTCATAGGCTGGGAAGAGAGAGGCCAGGAGAAAAGGCTGAGGAAACTGCTG
GCAAATGTGAAGGGCAAGAATGAATGCCCAAGGTGGGCAGCAGGTGAGGAAAGAGTCCCT
CACCTCCCTGGAGGAACAAGTCTTTGATTTGCTGAAAGGCATCCCGCACAGCCTGGGCGC
ACTTGGGACTCTGGCCATAAAAGTCCTGGAGAAGAGACCAAGGTTGCTGCTGCCATTCTT
GCACTGGCCTGGGGTACCCAAGTCCCCTCACTCACCGCTGTGACATCTCGGAAGAATTGG
TAGGAGTCCCCAAGGCCTGCAACAGCTACAACAGGAGCGCTGGCTGCCAGTGCCCCAGCC
ACCAGGTGGGGGTACTTCATCCTCATGTAGGCACTCAGCATCCCCCCATAACTGGGAGTA
CAGAGCACAGATCATGGTTGTGGGAAGCTGCCCACAACTCAGGCGAGCAGCCTCACTGTC
CTCCAGGCTGAGGTGCTAGGCTGCTCTTTCCCTGCTCAGAACGCCCAAGGGTGGGAAAGA
AGGACCTGAAACTGTCAGGCCCACACACCCTGATCCCAGGGCCAAGGCAGATACAGCCTT
CACTGGGAGAAGGCACCTGTGGGTGCCCTGCCCTGACCCAGCAATGAAGACATTGCAGAG
ACAAAGTCAGAAGGAATTGTCCCACTAGTGGGAACAACATAGCATACACTGCCTATGAGG
TCCACTCAAGGAGGGCTTCCAGAAGGAGGTAAAGCTAGACCCCGCCCTTCCACATGTGGG
GTAGGCATAGGATGTTGAGACTGTAAGAGACATCTCTTTGGCCCTCCTTGTATAGGGTGT
CAATCGGCACAACAGGGTGGAGCCTTAGAGTAGGGTAAGATTAGGACTCTAGGTTCTCTC
ATGGGTCCAGATCTGTCATGAAGGGAGGTCAAGGACCCACCTCCCTCCAAAGGCTATGGT
GGGGGCATCATGGACCCCAAGGTCCTGCCGCAGGGCCTGGAGCAGCACAGCAAAGTCGGC
CAGCGCCTGCTCCACAGTCAGCAGCTGTGTATATCCCCGCTGTGTGGACTGGACACCGAA
CGGAAGCGAT

>50 Get primers
TGCTGGCAAATGTGAAGGGCAAGAATGAATGCCCAAGGTGGGCAGCAGGTGAGGAAAGAG
TCCCTCACCTCCCTGGAGGAACAAGTCTTTGATTTGCTGAAAGGCATCCCGCACAGCCTG
GGCGCACTTGGGACTCTGGCCATAAAAGTCCTGGAGAAGAGACCAAGGTTGCTGCTGCCA
TTCTTGCACTGGCCTGGGGTACCCAAGTCCCCTCACTCACCGCTGTGACATCTCGGAAGA
ATTGGTAGGAGTCCCCAAGGCCTGCAACAGCTACAACAGGAGCGCTGGCTGCCAGTGCCC
CAGCCACCAGGTGGGGGTACTTCATCCTCATGTAGGCACTCAGCATCCCCCCATAACTGG
GAGTACAGAGCACAGATCATGGTTGTGGGAAGCTGCCCACAACTCAGGCGAGCAGCCTCA
CTGTCCTCCAGGCTGAGGTGCTAGGCTGCTCTTTCCCTGCTCAGAACGCCCAAGGGTGGG
AAAGAAGGACCTGAAACTGTCAGGCCCACACACCCTGATCCCAGGGCCAAGGCAGATACA
GCCTTCACTGGGAGAAGGCACCTGTGGGTGCCCTGCCCTGACCCAGCAATGAAGACATTG
CAGAGACAAAGTCAGAAGGAATTGTCCCACTAGTGGGAACAACATAGCATACACTGCCTA
TGAGGTCCACTCAAGGAGGGCTTCCAGAAGGAGGTAAAGCTAGACCCCGCCCTTCCACAT
GTGGGGTAGGCATAGGATGTTGAGACTGTAAGAGACATCTCTTTGGCCCTCCTTGTATAG
GGTGTCAATCGGCACAACAGGGTGGAGCCTTAGAGTAGGGTAAGATTAGGACTCTAGGTT
CTCTCATGGGTCCAGATCTGTCATGAAGGGAGGTCAAGGACCCACCTCCCTCCAAAGGCT
ATGGTGGGGGCATCATGGACCCCAAGGTCCTGCCGCAGGGCCTGGAGCAGCACAGCAAAG
TCGGCCAGCGCCTGCTCCACAGTCAGCAGCTGTGTATATCCCCGCTGTGTGGACTGGACA
CCGAACGGAAGCGATTTCCCATAGTACCGCTGCAGAAAGCAGGAAGGGATGGCTAATCCA
CTCCTCGGTGCTCCCCACCTCCTTCAACTCAGGGACTGCCAGGAACTGTACAGGTACCCA
CGTGCTCAGC

>51 Get primers
GCCTGGGCGCACTTGGGACTCTGGCCATAAAAGTCCTGGAGAAGAGACCAAGGTTGCTGC
TGCCATTCTTGCACTGGCCTGGGGTACCCAAGTCCCCTCACTCACCGCTGTGACATCTCG
GAAGAATTGGTAGGAGTCCCCAAGGCCTGCAACAGCTACAACAGGAGCGCTGGCTGCCAG
TGCCCCAGCCACCAGGTGGGGGTACTTCATCCTCATGTAGGCACTCAGCATCCCCCCATA
ACTGGGAGTACAGAGCACAGATCATGGTTGTGGGAAGCTGCCCACAACTCAGGCGAGCAG
CCTCACTGTCCTCCAGGCTGAGGTGCTAGGCTGCTCTTTCCCTGCTCAGAACGCCCAAGG
GTGGGAAAGAAGGACCTGAAACTGTCAGGCCCACACACCCTGATCCCAGGGCCAAGGCAG
ATACAGCCTTCACTGGGAGAAGGCACCTGTGGGTGCCCTGCCCTGACCCAGCAATGAAGA
CATTGCAGAGACAAAGTCAGAAGGAATTGTCCCACTAGTGGGAACAACATAGCATACACT
GCCTATGAGGTCCACTCAAGGAGGGCTTCCAGAAGGAGGTAAAGCTAGACCCCGCCCTTC
CACATGTGGGGTAGGCATAGGATGTTGAGACTGTAAGAGACATCTCTTTGGCCCTCCTTG
TATAGGGTGTCAATCGGCACAACAGGGTGGAGCCTTAGAGTAGGGTAAGATTAGGACTCT
AGGTTCTCTCATGGGTCCAGATCTGTCATGAAGGGAGGTCAAGGACCCACCTCCCTCCAA
AGGCTATGGTGGGGGCATCATGGACCCCAAGGTCCTGCCGCAGGGCCTGGAGCAGCACAG
CAAAGTCGGCCAGCGCCTGCTCCACAGTCAGCAGCTGTGTATATCCCCGCTGTGTGGACT
GGACACCGAACGGAAGCGATTTCCCATAGTACCGCTGCAGAAAGCAGGAAGGGATGGCTA
ATCCACTCCTCGGTGCTCCCCACCTCCTTCAACTCAGGGACTGCCAGGAACTGTACAGGT
ACCCACGTGCTCAGCAAAGACAAGCAGGGCCTCCTGCTGGGCTGCCAGTTCCACCATGAA
GCCAGAGTTGTTAGCGAAGGACCAGATATCCCCCTCATTCCCTGTGTAGAAAAAGATGGG
CCCTTCGCCC

>52 Get primers
TCTCGGAAGAATTGGTAGGAGTCCCCAAGGCCTGCAACAGCTACAACAGGAGCGCTGGCT
GCCAGTGCCCCAGCCACCAGGTGGGGGTACTTCATCCTCATGTAGGCACTCAGCATCCCC
CCATAACTGGGAGTACAGAGCACAGATCATGGTTGTGGGAAGCTGCCCACAACTCAGGCG
AGCAGCCTCACTGTCCTCCAGGCTGAGGTGCTAGGCTGCTCTTTCCCTGCTCAGAACGCC
CAAGGGTGGGAAAGAAGGACCTGAAACTGTCAGGCCCACACACCCTGATCCCAGGGCCAA
GGCAGATACAGCCTTCACTGGGAGAAGGCACCTGTGGGTGCCCTGCCCTGACCCAGCAAT
GAAGACATTGCAGAGACAAAGTCAGAAGGAATTGTCCCACTAGTGGGAACAACATAGCAT
ACACTGCCTATGAGGTCCACTCAAGGAGGGCTTCCAGAAGGAGGTAAAGCTAGACCCCGC
CCTTCCACATGTGGGGTAGGCATAGGATGTTGAGACTGTAAGAGACATCTCTTTGGCCCT
CCTTGTATAGGGTGTCAATCGGCACAACAGGGTGGAGCCTTAGAGTAGGGTAAGATTAGG
ACTCTAGGTTCTCTCATGGGTCCAGATCTGTCATGAAGGGAGGTCAAGGACCCACCTCCC
TCCAAAGGCTATGGTGGGGGCATCATGGACCCCAAGGTCCTGCCGCAGGGCCTGGAGCAG
CACAGCAAAGTCGGCCAGCGCCTGCTCCACAGTCAGCAGCTGTGTATATCCCCGCTGTGT
GGACTGGACACCGAACGGAAGCGATTTCCCATAGTACCGCTGCAGAAAGCAGGAAGGGAT
GGCTAATCCACTCCTCGGTGCTCCCCACCTCCTTCAACTCAGGGACTGCCAGGAACTGTA
CAGGTACCCACGTGCTCAGCAAAGACAAGCAGGGCCTCCTGCTGGGCTGCCAGTTCCACC
ATGAAGCCAGAGTTGTTAGCGAAGGACCAGATATCCCCCTCATTCCCTGTGTAGAAAAAG
ATGGGCCCTTCGCCCATCTTCCAGAACTTATCTGTTGGAAGTAAATGAGTTTCCATAAGG
CCAGGGAAACGCAGGTAGGAACCCATGCGGTCGAGCCAGCACTCACCTGACACTAGGAAC
CGCTGGCCAA

>53 Get primers
TCCCCCCATAACTGGGAGTACAGAGCACAGATCATGGTTGTGGGAAGCTGCCCACAACTC
AGGCGAGCAGCCTCACTGTCCTCCAGGCTGAGGTGCTAGGCTGCTCTTTCCCTGCTCAGA
ACGCCCAAGGGTGGGAAAGAAGGACCTGAAACTGTCAGGCCCACACACCCTGATCCCAGG
GCCAAGGCAGATACAGCCTTCACTGGGAGAAGGCACCTGTGGGTGCCCTGCCCTGACCCA
GCAATGAAGACATTGCAGAGACAAAGTCAGAAGGAATTGTCCCACTAGTGGGAACAACAT
AGCATACACTGCCTATGAGGTCCACTCAAGGAGGGCTTCCAGAAGGAGGTAAAGCTAGAC
CCCGCCCTTCCACATGTGGGGTAGGCATAGGATGTTGAGACTGTAAGAGACATCTCTTTG
GCCCTCCTTGTATAGGGTGTCAATCGGCACAACAGGGTGGAGCCTTAGAGTAGGGTAAGA
TTAGGACTCTAGGTTCTCTCATGGGTCCAGATCTGTCATGAAGGGAGGTCAAGGACCCAC
CTCCCTCCAAAGGCTATGGTGGGGGCATCATGGACCCCAAGGTCCTGCCGCAGGGCCTGG
AGCAGCACAGCAAAGTCGGCCAGCGCCTGCTCCACAGTCAGCAGCTGTGTATATCCCCGC
TGTGTGGACTGGACACCGAACGGAAGCGATTTCCCATAGTACCGCTGCAGAAAGCAGGAA
GGGATGGCTAATCCACTCCTCGGTGCTCCCCACCTCCTTCAACTCAGGGACTGCCAGGAA
CTGTACAGGTACCCACGTGCTCAGCAAAGACAAGCAGGGCCTCCTGCTGGGCTGCCAGTT
CCACCATGAAGCCAGAGTTGTTAGCGAAGGACCAGATATCCCCCTCATTCCCTGTGTAGA
AAAAGATGGGCCCTTCGCCCATCTTCCAGAACTTATCTGTTGGAAGTAAATGAGTTTCCA
TAAGGCCAGGGAAACGCAGGTAGGAACCCATGCGGTCGAGCCAGCACTCACCTGACACTA
GGAACCGCTGGCCAAAGGTTTTGTTGCCGAAACTCTCAAAGTTGAAATGGTCCATGTATT
GCTCAAAATAATTCTCATGAAAGTCAGGGTCTAGAACTCTGTCGGCTGAGGGCAGGTGCA
GAGACTCAGG

>54 Get primers
TCAGAACGCCCAAGGGTGGGAAAGAAGGACCTGAAACTGTCAGGCCCACACACCCTGATC
CCAGGGCCAAGGCAGATACAGCCTTCACTGGGAGAAGGCACCTGTGGGTGCCCTGCCCTG
ACCCAGCAATGAAGACATTGCAGAGACAAAGTCAGAAGGAATTGTCCCACTAGTGGGAAC
AACATAGCATACACTGCCTATGAGGTCCACTCAAGGAGGGCTTCCAGAAGGAGGTAAAGC
TAGACCCCGCCCTTCCACATGTGGGGTAGGCATAGGATGTTGAGACTGTAAGAGACATCT
CTTTGGCCCTCCTTGTATAGGGTGTCAATCGGCACAACAGGGTGGAGCCTTAGAGTAGGG
TAAGATTAGGACTCTAGGTTCTCTCATGGGTCCAGATCTGTCATGAAGGGAGGTCAAGGA
CCCACCTCCCTCCAAAGGCTATGGTGGGGGCATCATGGACCCCAAGGTCCTGCCGCAGGG
CCTGGAGCAGCACAGCAAAGTCGGCCAGCGCCTGCTCCACAGTCAGCAGCTGTGTATATC
CCCGCTGTGTGGACTGGACACCGAACGGAAGCGATTTCCCATAGTACCGCTGCAGAAAGC
AGGAAGGGATGGCTAATCCACTCCTCGGTGCTCCCCACCTCCTTCAACTCAGGGACTGCC
AGGAACTGTACAGGTACCCACGTGCTCAGCAAAGACAAGCAGGGCCTCCTGCTGGGCTGC
CAGTTCCACCATGAAGCCAGAGTTGTTAGCGAAGGACCAGATATCCCCCTCATTCCCTGT
GTAGAAAAAGATGGGCCCTTCGCCCATCTTCCAGAACTTATCTGTTGGAAGTAAATGAGT
TTCCATAAGGCCAGGGAAACGCAGGTAGGAACCCATGCGGTCGAGCCAGCACTCACCTGA
CACTAGGAACCGCTGGCCAAAGGTTTTGTTGCCGAAACTCTCAAAGTTGAAATGGTCCAT
GTATTGCTCAAAATAATTCTCATGAAAGTCAGGGTCTAGAACTCTGTCGGCTGAGGGCAG
GTGCAGAGACTCAGGAGCTGGTTGGGATCATCAGGGATCTAGGCGGGTCAGGAGGAAGGG
CAGCCAGTCTGTACTCACCTCTGGCCTGGAGGTTGCACAGTCCCAGTGACAGCAGCAGGA
CCAGGATCCA

>55 Get primers
CCCTGACCCAGCAATGAAGACATTGCAGAGACAAAGTCAGAAGGAATTGTCCCACTAGTG
GGAACAACATAGCATACACTGCCTATGAGGTCCACTCAAGGAGGGCTTCCAGAAGGAGGT
AAAGCTAGACCCCGCCCTTCCACATGTGGGGTAGGCATAGGATGTTGAGACTGTAAGAGA
CATCTCTTTGGCCCTCCTTGTATAGGGTGTCAATCGGCACAACAGGGTGGAGCCTTAGAG
TAGGGTAAGATTAGGACTCTAGGTTCTCTCATGGGTCCAGATCTGTCATGAAGGGAGGTC
AAGGACCCACCTCCCTCCAAAGGCTATGGTGGGGGCATCATGGACCCCAAGGTCCTGCCG
CAGGGCCTGGAGCAGCACAGCAAAGTCGGCCAGCGCCTGCTCCACAGTCAGCAGCTGTGT
ATATCCCCGCTGTGTGGACTGGACACCGAACGGAAGCGATTTCCCATAGTACCGCTGCAG
AAAGCAGGAAGGGATGGCTAATCCACTCCTCGGTGCTCCCCACCTCCTTCAACTCAGGGA
CTGCCAGGAACTGTACAGGTACCCACGTGCTCAGCAAAGACAAGCAGGGCCTCCTGCTGG
GCTGCCAGTTCCACCATGAAGCCAGAGTTGTTAGCGAAGGACCAGATATCCCCCTCATTC
CCTGTGTAGAAAAAGATGGGCCCTTCGCCCATCTTCCAGAACTTATCTGTTGGAAGTAAA
TGAGTTTCCATAAGGCCAGGGAAACGCAGGTAGGAACCCATGCGGTCGAGCCAGCACTCA
CCTGACACTAGGAACCGCTGGCCAAAGGTTTTGTTGCCGAAACTCTCAAAGTTGAAATGG
TCCATGTATTGCTCAAAATAATTCTCATGAAAGTCAGGGTCTAGAACTCTGTCGGCTGAG
GGCAGGTGCAGAGACTCAGGAGCTGGTTGGGATCATCAGGGATCTAGGCGGGTCAGGAGG
AAGGGCAGCCAGTCTGTACTCACCTCTGGCCTGGAGGTTGCACAGTCCCAGTGACAGCAG
CAGGACCAGGATCCAGGAGGGGACACCATGGTCCACAGGGTAACAAGGATGGAAGTTCAT
GCTTGATTCTGAGCCGGGCGCTGACTGTCATGTGATTTGGTCACATGACCGACACAACGG
GCGGGGCAGC

>56 Get primers
GAGGTAAAGCTAGACCCCGCCCTTCCACATGTGGGGTAGGCATAGGATGTTGAGACTGTA
AGAGACATCTCTTTGGCCCTCCTTGTATAGGGTGTCAATCGGCACAACAGGGTGGAGCCT
TAGAGTAGGGTAAGATTAGGACTCTAGGTTCTCTCATGGGTCCAGATCTGTCATGAAGGG
AGGTCAAGGACCCACCTCCCTCCAAAGGCTATGGTGGGGGCATCATGGACCCCAAGGTCC
TGCCGCAGGGCCTGGAGCAGCACAGCAAAGTCGGCCAGCGCCTGCTCCACAGTCAGCAGC
TGTGTATATCCCCGCTGTGTGGACTGGACACCGAACGGAAGCGATTTCCCATAGTACCGC
TGCAGAAAGCAGGAAGGGATGGCTAATCCACTCCTCGGTGCTCCCCACCTCCTTCAACTC
AGGGACTGCCAGGAACTGTACAGGTACCCACGTGCTCAGCAAAGACAAGCAGGGCCTCCT
GCTGGGCTGCCAGTTCCACCATGAAGCCAGAGTTGTTAGCGAAGGACCAGATATCCCCCT
CATTCCCTGTGTAGAAAAAGATGGGCCCTTCGCCCATCTTCCAGAACTTATCTGTTGGAA
GTAAATGAGTTTCCATAAGGCCAGGGAAACGCAGGTAGGAACCCATGCGGTCGAGCCAGC
ACTCACCTGACACTAGGAACCGCTGGCCAAAGGTTTTGTTGCCGAAACTCTCAAAGTTGA
AATGGTCCATGTATTGCTCAAAATAATTCTCATGAAAGTCAGGGTCTAGAACTCTGTCGG
CTGAGGGCAGGTGCAGAGACTCAGGAGCTGGTTGGGATCATCAGGGATCTAGGCGGGTCA
GGAGGAAGGGCAGCCAGTCTGTACTCACCTCTGGCCTGGAGGTTGCACAGTCCCAGTGAC
AGCAGCAGGACCAGGATCCAGGAGGGGACACCATGGTCCACAGGGTAACAAGGATGGAAG
TTCATGCTTGATTCTGAGCCGGGCGCTGACTGTCATGTGATTTGGTCACATGACCGACAC
AACGGGCGGGGCAGCATCACGTGATAGTCTGGCGGGGGCTGTCCTACTGTGGCTGGATTC
TAGTTGGAGGATCAGCCTACTCTTCTTCAGTTTCCCGGTTCCTCCAAATTTCTGGGCTCC
TACTTGTTTC

>57 Get primers
AGCCTTAGAGTAGGGTAAGATTAGGACTCTAGGTTCTCTCATGGGTCCAGATCTGTCATG
AAGGGAGGTCAAGGACCCACCTCCCTCCAAAGGCTATGGTGGGGGCATCATGGACCCCAA
GGTCCTGCCGCAGGGCCTGGAGCAGCACAGCAAAGTCGGCCAGCGCCTGCTCCACAGTCA
GCAGCTGTGTATATCCCCGCTGTGTGGACTGGACACCGAACGGAAGCGATTTCCCATAGT
ACCGCTGCAGAAAGCAGGAAGGGATGGCTAATCCACTCCTCGGTGCTCCCCACCTCCTTC
AACTCAGGGACTGCCAGGAACTGTACAGGTACCCACGTGCTCAGCAAAGACAAGCAGGGC
CTCCTGCTGGGCTGCCAGTTCCACCATGAAGCCAGAGTTGTTAGCGAAGGACCAGATATC
CCCCTCATTCCCTGTGTAGAAAAAGATGGGCCCTTCGCCCATCTTCCAGAACTTATCTGT
TGGAAGTAAATGAGTTTCCATAAGGCCAGGGAAACGCAGGTAGGAACCCATGCGGTCGAG
CCAGCACTCACCTGACACTAGGAACCGCTGGCCAAAGGTTTTGTTGCCGAAACTCTCAAA
GTTGAAATGGTCCATGTATTGCTCAAAATAATTCTCATGAAAGTCAGGGTCTAGAACTCT
GTCGGCTGAGGGCAGGTGCAGAGACTCAGGAGCTGGTTGGGATCATCAGGGATCTAGGCG
GGTCAGGAGGAAGGGCAGCCAGTCTGTACTCACCTCTGGCCTGGAGGTTGCACAGTCCCA
GTGACAGCAGCAGGACCAGGATCCAGGAGGGGACACCATGGTCCACAGGGTAACAAGGAT
GGAAGTTCATGCTTGATTCTGAGCCGGGCGCTGACTGTCATGTGATTTGGTCACATGACC
GACACAACGGGCGGGGCAGCATCACGTGATAGTCTGGCGGGGGCTGTCCTACTGTGGCTG
GATTCTAGTTGGAGGATCAGCCTACTCTTCTTCAGTTTCCCGGTTCCTCCAAATTTCTGG
GCTCCTACTTGTTTCCACAGAGATGGATACTGTGGAGGTCCAGGAAGCAGAGAGATGGCT
AAGGCTCATCAGGACCGTATGATCTCCCAAGTGTCCAGCTACTGAGTACCACAAGGTGAT
GGGTGGGAGG

>58 Get primers
CCCAAGGTCCTGCCGCAGGGCCTGGAGCAGCACAGCAAAGTCGGCCAGCGCCTGCTCCAC
AGTCAGCAGCTGTGTATATCCCCGCTGTGTGGACTGGACACCGAACGGAAGCGATTTCCC
ATAGTACCGCTGCAGAAAGCAGGAAGGGATGGCTAATCCACTCCTCGGTGCTCCCCACCT
CCTTCAACTCAGGGACTGCCAGGAACTGTACAGGTACCCACGTGCTCAGCAAAGACAAGC
AGGGCCTCCTGCTGGGCTGCCAGTTCCACCATGAAGCCAGAGTTGTTAGCGAAGGACCAG
ATATCCCCCTCATTCCCTGTGTAGAAAAAGATGGGCCCTTCGCCCATCTTCCAGAACTTA
TCTGTTGGAAGTAAATGAGTTTCCATAAGGCCAGGGAAACGCAGGTAGGAACCCATGCGG
TCGAGCCAGCACTCACCTGACACTAGGAACCGCTGGCCAAAGGTTTTGTTGCCGAAACTC
TCAAAGTTGAAATGGTCCATGTATTGCTCAAAATAATTCTCATGAAAGTCAGGGTCTAGA
ACTCTGTCGGCTGAGGGCAGGTGCAGAGACTCAGGAGCTGGTTGGGATCATCAGGGATCT
AGGCGGGTCAGGAGGAAGGGCAGCCAGTCTGTACTCACCTCTGGCCTGGAGGTTGCACAG
TCCCAGTGACAGCAGCAGGACCAGGATCCAGGAGGGGACACCATGGTCCACAGGGTAACA
AGGATGGAAGTTCATGCTTGATTCTGAGCCGGGCGCTGACTGTCATGTGATTTGGTCACA
TGACCGACACAACGGGCGGGGCAGCATCACGTGATAGTCTGGCGGGGGCTGTCCTACTGT
GGCTGGATTCTAGTTGGAGGATCAGCCTACTCTTCTTCAGTTTCCCGGTTCCTCCAAATT
TCTGGGCTCCTACTTGTTTCCACAGAGATGGATACTGTGGAGGTCCAGGAAGCAGAGAGA
TGGCTAAGGCTCATCAGGACCGTATGATCTCCCAAGTGTCCAGCTACTGAGTACCACAAG
GTGATGGGTGGGAGGGTCCTCCCACGGAAGGATACCGCAGTCCCTAGGGGTTGCAAGCCC
CACATGTTCCACTGGCTGCTAGAGCTACCTACTCAATCAGCCCTGGGCATCACCATCAGG
TACTCGGCCA

>67 Get primers
GGAGAAAAGGCTGAGGAAACTGCTGGCAAATGTGAAGGGCAAGAATGAATGCCCAAGGTG
GGCAGCAGGTGAGGAAAGAGTCCCTCACCTCCCTGGAGGAACAAGTCTTTGATTTGCTGA
AAGGCATCCCGCACAGCCTGGGCGCACTTGGGACTCTGGCCATAAAAGTCCTGGAGAAGA
GACCAAGGTTGCTGCTGCCATTCTTGCACTGGCCTGGGGTACCCAAGTCCCCTCACTCAC
CGCTGTGACATCTCGGAAGAATTGGTAGGAGTCCCCAAGGCCTGCAACAGCTACAACAGG
AGCGCTGGCTGCCAGTGCCCCAGCCACCAGGTGGGGGTACTTCATCCTCATGTAGGCACT
CAGCATCCCCCCATAACTGGGAGTACAGAGCACAGATCATGGTTGTGGGAAGCTGCCCAC
AACTCAGGCGAGCAGCCTCACTGTCCTCCAGGCTGAGGTGCTAGGCTGCTCTTTCCCTGC
TCAGAACGCCCAAGGGTGGGAAAGAAGGACCTGAAACTGTCAGGCCCACACACCCTGATC
CCAGGGCCAAGGCAGATACAGCCTTCACTGGGAGAAGGCACCTGTGGGTGCCCTGCCCTG
ACCCAGCAATGAAGACATTGCAGAGACAAAGTCAGAAGGAATTGTCCCACTAGTGGGAAC
AACATAGCATACACTGCCTATGAGGTCCACTCAAGGAGGGCTTCCAGAAGGAGGTAAAGC
TAGACCCCGCCCTTCCACATGTGGGGTAGGCATAGGATGTTGAGACTGTAAGAGACATCT
CTTTGGCCCTCCTTGTATAGGGTGTCAATCGGCACAACAGGGTGGAGCCTTAGAGTAGGG
TAAGATTAGGACTCTAGGTTCTCTCATGGGTCCAGATCTGTCATGAAGGGAGGTCAAGGA
CCCACCTCCCTCCAAAGGCTATGGTGGGGGCATCATGGACCCCAAGGTCCTGCCGCAGGG
CCTGGAGCAGCACAGCAAAGTCGGCCAGCGCCTGCTCCACAGTCAGCAGCTGTGTATATC
CCCGCTGTGTGGACTGGACACCGAACGGAAGCGATTTCCCATAGTACCGCTGCAGAAAGC
AGGAAGGGATGGCTAATCCA

>68 Get primers
GATTTGCTGAAAGGCATCCCGCACAGCCTGGGCGCACTTGGGACTCTGGCCATAAAAGTC
CTGGAGAAGAGACCAAGGTTGCTGCTGCCATTCTTGCACTGGCCTGGGGTACCCAAGTCC
CCTCACTCACCGCTGTGACATCTCGGAAGAATTGGTAGGAGTCCCCAAGGCCTGCAACAG
CTACAACAGGAGCGCTGGCTGCCAGTGCCCCAGCCACCAGGTGGGGGTACTTCATCCTCA
TGTAGGCACTCAGCATCCCCCCATAACTGGGAGTACAGAGCACAGATCATGGTTGTGGGA
AGCTGCCCACAACTCAGGCGAGCAGCCTCACTGTCCTCCAGGCTGAGGTGCTAGGCTGCT
CTTTCCCTGCTCAGAACGCCCAAGGGTGGGAAAGAAGGACCTGAAACTGTCAGGCCCACA
CACCCTGATCCCAGGGCCAAGGCAGATACAGCCTTCACTGGGAGAAGGCACCTGTGGGTG
CCCTGCCCTGACCCAGCAATGAAGACATTGCAGAGACAAAGTCAGAAGGAATTGTCCCAC
TAGTGGGAACAACATAGCATACACTGCCTATGAGGTCCACTCAAGGAGGGCTTCCAGAAG
GAGGTAAAGCTAGACCCCGCCCTTCCACATGTGGGGTAGGCATAGGATGTTGAGACTGTA
AGAGACATCTCTTTGGCCCTCCTTGTATAGGGTGTCAATCGGCACAACAGGGTGGAGCCT
TAGAGTAGGGTAAGATTAGGACTCTAGGTTCTCTCATGGGTCCAGATCTGTCATGAAGGG
AGGTCAAGGACCCACCTCCCTCCAAAGGCTATGGTGGGGGCATCATGGACCCCAAGGTCC
TGCCGCAGGGCCTGGAGCAGCACAGCAAAGTCGGCCAGCGCCTGCTCCACAGTCAGCAGC
TGTGTATATCCCCGCTGTGTGGACTGGACACCGAACGGAAGCGATTTCCCATAGTACCGC
TGCAGAAAGCAGGAAGGGATGGCTAATCCACTCCTCGGTGCTCCCCACCTCCTTCAACTC
AGGGACTGCCAGGAACTGTACAGGTACCCACGTGCTCAGCAAAGACAAGCAGGGCCTCCT
GCTGGGCTGCCAGTTCCACC

>69 Get primers
ACCCAAGTCCCCTCACTCACCGCTGTGACATCTCGGAAGAATTGGTAGGAGTCCCCAAGG
CCTGCAACAGCTACAACAGGAGCGCTGGCTGCCAGTGCCCCAGCCACCAGGTGGGGGTAC
TTCATCCTCATGTAGGCACTCAGCATCCCCCCATAACTGGGAGTACAGAGCACAGATCAT
GGTTGTGGGAAGCTGCCCACAACTCAGGCGAGCAGCCTCACTGTCCTCCAGGCTGAGGTG
CTAGGCTGCTCTTTCCCTGCTCAGAACGCCCAAGGGTGGGAAAGAAGGACCTGAAACTGT
CAGGCCCACACACCCTGATCCCAGGGCCAAGGCAGATACAGCCTTCACTGGGAGAAGGCA
CCTGTGGGTGCCCTGCCCTGACCCAGCAATGAAGACATTGCAGAGACAAAGTCAGAAGGA
ATTGTCCCACTAGTGGGAACAACATAGCATACACTGCCTATGAGGTCCACTCAAGGAGGG
CTTCCAGAAGGAGGTAAAGCTAGACCCCGCCCTTCCACATGTGGGGTAGGCATAGGATGT
TGAGACTGTAAGAGACATCTCTTTGGCCCTCCTTGTATAGGGTGTCAATCGGCACAACAG
GGTGGAGCCTTAGAGTAGGGTAAGATTAGGACTCTAGGTTCTCTCATGGGTCCAGATCTG
TCATGAAGGGAGGTCAAGGACCCACCTCCCTCCAAAGGCTATGGTGGGGGCATCATGGAC
CCCAAGGTCCTGCCGCAGGGCCTGGAGCAGCACAGCAAAGTCGGCCAGCGCCTGCTCCAC
AGTCAGCAGCTGTGTATATCCCCGCTGTGTGGACTGGACACCGAACGGAAGCGATTTCCC
ATAGTACCGCTGCAGAAAGCAGGAAGGGATGGCTAATCCACTCCTCGGTGCTCCCCACCT
CCTTCAACTCAGGGACTGCCAGGAACTGTACAGGTACCCACGTGCTCAGCAAAGACAAGC
AGGGCCTCCTGCTGGGCTGCCAGTTCCACCATGAAGCCAGAGTTGTTAGCGAAGGACCAG
ATATCCCCCTCATTCCCTGTGTAGAAAAAGATGGGCCCTTCGCCCATCTTCCAGAACTTA
TCTGTTGGAAGTAAATGAGT

>70 Get primers
GTGGGGGTACTTCATCCTCATGTAGGCACTCAGCATCCCCCCATAACTGGGAGTACAGAG
CACAGATCATGGTTGTGGGAAGCTGCCCACAACTCAGGCGAGCAGCCTCACTGTCCTCCA
GGCTGAGGTGCTAGGCTGCTCTTTCCCTGCTCAGAACGCCCAAGGGTGGGAAAGAAGGAC
CTGAAACTGTCAGGCCCACACACCCTGATCCCAGGGCCAAGGCAGATACAGCCTTCACTG
GGAGAAGGCACCTGTGGGTGCCCTGCCCTGACCCAGCAATGAAGACATTGCAGAGACAAA
GTCAGAAGGAATTGTCCCACTAGTGGGAACAACATAGCATACACTGCCTATGAGGTCCAC
TCAAGGAGGGCTTCCAGAAGGAGGTAAAGCTAGACCCCGCCCTTCCACATGTGGGGTAGG
CATAGGATGTTGAGACTGTAAGAGACATCTCTTTGGCCCTCCTTGTATAGGGTGTCAATC
GGCACAACAGGGTGGAGCCTTAGAGTAGGGTAAGATTAGGACTCTAGGTTCTCTCATGGG
TCCAGATCTGTCATGAAGGGAGGTCAAGGACCCACCTCCCTCCAAAGGCTATGGTGGGGG
CATCATGGACCCCAAGGTCCTGCCGCAGGGCCTGGAGCAGCACAGCAAAGTCGGCCAGCG
CCTGCTCCACAGTCAGCAGCTGTGTATATCCCCGCTGTGTGGACTGGACACCGAACGGAA
GCGATTTCCCATAGTACCGCTGCAGAAAGCAGGAAGGGATGGCTAATCCACTCCTCGGTG
CTCCCCACCTCCTTCAACTCAGGGACTGCCAGGAACTGTACAGGTACCCACGTGCTCAGC
AAAGACAAGCAGGGCCTCCTGCTGGGCTGCCAGTTCCACCATGAAGCCAGAGTTGTTAGC
GAAGGACCAGATATCCCCCTCATTCCCTGTGTAGAAAAAGATGGGCCCTTCGCCCATCTT
CCAGAACTTATCTGTTGGAAGTAAATGAGTTTCCATAAGGCCAGGGAAACGCAGGTAGGA
ACCCATGCGGTCGAGCCAGCACTCACCTGACACTAGGAACCGCTGGCCAAAGGTTTTGTT
GCCGAAACTCTCAAAGTTGA

>71 Get primers
CTGTCCTCCAGGCTGAGGTGCTAGGCTGCTCTTTCCCTGCTCAGAACGCCCAAGGGTGGG
AAAGAAGGACCTGAAACTGTCAGGCCCACACACCCTGATCCCAGGGCCAAGGCAGATACA
GCCTTCACTGGGAGAAGGCACCTGTGGGTGCCCTGCCCTGACCCAGCAATGAAGACATTG
CAGAGACAAAGTCAGAAGGAATTGTCCCACTAGTGGGAACAACATAGCATACACTGCCTA
TGAGGTCCACTCAAGGAGGGCTTCCAGAAGGAGGTAAAGCTAGACCCCGCCCTTCCACAT
GTGGGGTAGGCATAGGATGTTGAGACTGTAAGAGACATCTCTTTGGCCCTCCTTGTATAG
GGTGTCAATCGGCACAACAGGGTGGAGCCTTAGAGTAGGGTAAGATTAGGACTCTAGGTT
CTCTCATGGGTCCAGATCTGTCATGAAGGGAGGTCAAGGACCCACCTCCCTCCAAAGGCT
ATGGTGGGGGCATCATGGACCCCAAGGTCCTGCCGCAGGGCCTGGAGCAGCACAGCAAAG
TCGGCCAGCGCCTGCTCCACAGTCAGCAGCTGTGTATATCCCCGCTGTGTGGACTGGACA
CCGAACGGAAGCGATTTCCCATAGTACCGCTGCAGAAAGCAGGAAGGGATGGCTAATCCA
CTCCTCGGTGCTCCCCACCTCCTTCAACTCAGGGACTGCCAGGAACTGTACAGGTACCCA
CGTGCTCAGCAAAGACAAGCAGGGCCTCCTGCTGGGCTGCCAGTTCCACCATGAAGCCAG
AGTTGTTAGCGAAGGACCAGATATCCCCCTCATTCCCTGTGTAGAAAAAGATGGGCCCTT
CGCCCATCTTCCAGAACTTATCTGTTGGAAGTAAATGAGTTTCCATAAGGCCAGGGAAAC
GCAGGTAGGAACCCATGCGGTCGAGCCAGCACTCACCTGACACTAGGAACCGCTGGCCAA
AGGTTTTGTTGCCGAAACTCTCAAAGTTGAAATGGTCCATGTATTGCTCAAAATAATTCT
CATGAAAGTCAGGGTCTAGAACTCTGTCGGCTGAGGGCAGGTGCAGAGACTCAGGAGCTG
GTTGGGATCATCAGGGATCT

>72 Get primers
GGCAGATACAGCCTTCACTGGGAGAAGGCACCTGTGGGTGCCCTGCCCTGACCCAGCAAT
GAAGACATTGCAGAGACAAAGTCAGAAGGAATTGTCCCACTAGTGGGAACAACATAGCAT
ACACTGCCTATGAGGTCCACTCAAGGAGGGCTTCCAGAAGGAGGTAAAGCTAGACCCCGC
CCTTCCACATGTGGGGTAGGCATAGGATGTTGAGACTGTAAGAGACATCTCTTTGGCCCT
CCTTGTATAGGGTGTCAATCGGCACAACAGGGTGGAGCCTTAGAGTAGGGTAAGATTAGG
ACTCTAGGTTCTCTCATGGGTCCAGATCTGTCATGAAGGGAGGTCAAGGACCCACCTCCC
TCCAAAGGCTATGGTGGGGGCATCATGGACCCCAAGGTCCTGCCGCAGGGCCTGGAGCAG
CACAGCAAAGTCGGCCAGCGCCTGCTCCACAGTCAGCAGCTGTGTATATCCCCGCTGTGT
GGACTGGACACCGAACGGAAGCGATTTCCCATAGTACCGCTGCAGAAAGCAGGAAGGGAT
GGCTAATCCACTCCTCGGTGCTCCCCACCTCCTTCAACTCAGGGACTGCCAGGAACTGTA
CAGGTACCCACGTGCTCAGCAAAGACAAGCAGGGCCTCCTGCTGGGCTGCCAGTTCCACC
ATGAAGCCAGAGTTGTTAGCGAAGGACCAGATATCCCCCTCATTCCCTGTGTAGAAAAAG
ATGGGCCCTTCGCCCATCTTCCAGAACTTATCTGTTGGAAGTAAATGAGTTTCCATAAGG
CCAGGGAAACGCAGGTAGGAACCCATGCGGTCGAGCCAGCACTCACCTGACACTAGGAAC
CGCTGGCCAAAGGTTTTGTTGCCGAAACTCTCAAAGTTGAAATGGTCCATGTATTGCTCA
AAATAATTCTCATGAAAGTCAGGGTCTAGAACTCTGTCGGCTGAGGGCAGGTGCAGAGAC
TCAGGAGCTGGTTGGGATCATCAGGGATCTAGGCGGGTCAGGAGGAAGGGCAGCCAGTCT
GTACTCACCTCTGGCCTGGAGGTTGCACAGTCCCAGTGACAGCAGCAGGACCAGGATCCA
GGAGGGGACACCATGGTCCA

>73 Get primers
AACATAGCATACACTGCCTATGAGGTCCACTCAAGGAGGGCTTCCAGAAGGAGGTAAAGC
TAGACCCCGCCCTTCCACATGTGGGGTAGGCATAGGATGTTGAGACTGTAAGAGACATCT
CTTTGGCCCTCCTTGTATAGGGTGTCAATCGGCACAACAGGGTGGAGCCTTAGAGTAGGG
TAAGATTAGGACTCTAGGTTCTCTCATGGGTCCAGATCTGTCATGAAGGGAGGTCAAGGA
CCCACCTCCCTCCAAAGGCTATGGTGGGGGCATCATGGACCCCAAGGTCCTGCCGCAGGG
CCTGGAGCAGCACAGCAAAGTCGGCCAGCGCCTGCTCCACAGTCAGCAGCTGTGTATATC
CCCGCTGTGTGGACTGGACACCGAACGGAAGCGATTTCCCATAGTACCGCTGCAGAAAGC
AGGAAGGGATGGCTAATCCACTCCTCGGTGCTCCCCACCTCCTTCAACTCAGGGACTGCC
AGGAACTGTACAGGTACCCACGTGCTCAGCAAAGACAAGCAGGGCCTCCTGCTGGGCTGC
CAGTTCCACCATGAAGCCAGAGTTGTTAGCGAAGGACCAGATATCCCCCTCATTCCCTGT
GTAGAAAAAGATGGGCCCTTCGCCCATCTTCCAGAACTTATCTGTTGGAAGTAAATGAGT
TTCCATAAGGCCAGGGAAACGCAGGTAGGAACCCATGCGGTCGAGCCAGCACTCACCTGA
CACTAGGAACCGCTGGCCAAAGGTTTTGTTGCCGAAACTCTCAAAGTTGAAATGGTCCAT
GTATTGCTCAAAATAATTCTCATGAAAGTCAGGGTCTAGAACTCTGTCGGCTGAGGGCAG
GTGCAGAGACTCAGGAGCTGGTTGGGATCATCAGGGATCTAGGCGGGTCAGGAGGAAGGG
CAGCCAGTCTGTACTCACCTCTGGCCTGGAGGTTGCACAGTCCCAGTGACAGCAGCAGGA
CCAGGATCCAGGAGGGGACACCATGGTCCACAGGGTAACAAGGATGGAAGTTCATGCTTG
ATTCTGAGCCGGGCGCTGACTGTCATGTGATTTGGTCACATGACCGACACAACGGGCGGG
GCAGCATCACGTGATAGTCT

>74 Get primers
AGAGACATCTCTTTGGCCCTCCTTGTATAGGGTGTCAATCGGCACAACAGGGTGGAGCCT
TAGAGTAGGGTAAGATTAGGACTCTAGGTTCTCTCATGGGTCCAGATCTGTCATGAAGGG
AGGTCAAGGACCCACCTCCCTCCAAAGGCTATGGTGGGGGCATCATGGACCCCAAGGTCC
TGCCGCAGGGCCTGGAGCAGCACAGCAAAGTCGGCCAGCGCCTGCTCCACAGTCAGCAGC
TGTGTATATCCCCGCTGTGTGGACTGGACACCGAACGGAAGCGATTTCCCATAGTACCGC
TGCAGAAAGCAGGAAGGGATGGCTAATCCACTCCTCGGTGCTCCCCACCTCCTTCAACTC
AGGGACTGCCAGGAACTGTACAGGTACCCACGTGCTCAGCAAAGACAAGCAGGGCCTCCT
GCTGGGCTGCCAGTTCCACCATGAAGCCAGAGTTGTTAGCGAAGGACCAGATATCCCCCT
CATTCCCTGTGTAGAAAAAGATGGGCCCTTCGCCCATCTTCCAGAACTTATCTGTTGGAA
GTAAATGAGTTTCCATAAGGCCAGGGAAACGCAGGTAGGAACCCATGCGGTCGAGCCAGC
ACTCACCTGACACTAGGAACCGCTGGCCAAAGGTTTTGTTGCCGAAACTCTCAAAGTTGA
AATGGTCCATGTATTGCTCAAAATAATTCTCATGAAAGTCAGGGTCTAGAACTCTGTCGG
CTGAGGGCAGGTGCAGAGACTCAGGAGCTGGTTGGGATCATCAGGGATCTAGGCGGGTCA
GGAGGAAGGGCAGCCAGTCTGTACTCACCTCTGGCCTGGAGGTTGCACAGTCCCAGTGAC
AGCAGCAGGACCAGGATCCAGGAGGGGACACCATGGTCCACAGGGTAACAAGGATGGAAG
TTCATGCTTGATTCTGAGCCGGGCGCTGACTGTCATGTGATTTGGTCACATGACCGACAC
AACGGGCGGGGCAGCATCACGTGATAGTCTGGCGGGGGCTGTCCTACTGTGGCTGGATTC
TAGTTGGAGGATCAGCCTACTCTTCTTCAGTTTCCCGGTTCCTCCAAATTTCTGGGCTCC
TACTTGTTTCCACAGAGATG

>75 Get primers
TCATGAAGGGAGGTCAAGGACCCACCTCCCTCCAAAGGCTATGGTGGGGGCATCATGGAC
CCCAAGGTCCTGCCGCAGGGCCTGGAGCAGCACAGCAAAGTCGGCCAGCGCCTGCTCCAC
AGTCAGCAGCTGTGTATATCCCCGCTGTGTGGACTGGACACCGAACGGAAGCGATTTCCC
ATAGTACCGCTGCAGAAAGCAGGAAGGGATGGCTAATCCACTCCTCGGTGCTCCCCACCT
CCTTCAACTCAGGGACTGCCAGGAACTGTACAGGTACCCACGTGCTCAGCAAAGACAAGC
AGGGCCTCCTGCTGGGCTGCCAGTTCCACCATGAAGCCAGAGTTGTTAGCGAAGGACCAG
ATATCCCCCTCATTCCCTGTGTAGAAAAAGATGGGCCCTTCGCCCATCTTCCAGAACTTA
TCTGTTGGAAGTAAATGAGTTTCCATAAGGCCAGGGAAACGCAGGTAGGAACCCATGCGG
TCGAGCCAGCACTCACCTGACACTAGGAACCGCTGGCCAAAGGTTTTGTTGCCGAAACTC
TCAAAGTTGAAATGGTCCATGTATTGCTCAAAATAATTCTCATGAAAGTCAGGGTCTAGA
ACTCTGTCGGCTGAGGGCAGGTGCAGAGACTCAGGAGCTGGTTGGGATCATCAGGGATCT
AGGCGGGTCAGGAGGAAGGGCAGCCAGTCTGTACTCACCTCTGGCCTGGAGGTTGCACAG
TCCCAGTGACAGCAGCAGGACCAGGATCCAGGAGGGGACACCATGGTCCACAGGGTAACA
AGGATGGAAGTTCATGCTTGATTCTGAGCCGGGCGCTGACTGTCATGTGATTTGGTCACA
TGACCGACACAACGGGCGGGGCAGCATCACGTGATAGTCTGGCGGGGGCTGTCCTACTGT
GGCTGGATTCTAGTTGGAGGATCAGCCTACTCTTCTTCAGTTTCCCGGTTCCTCCAAATT
TCTGGGCTCCTACTTGTTTCCACAGAGATGGATACTGTGGAGGTCCAGGAAGCAGAGAGA
TGGCTAAGGCTCATCAGGACCGTATGATCTCCCAAGTGTCCAGCTACTGAGTACCACAAG
GTGATGGGTGGGAGGGTCCT

>85 Get primers
GGCTGGGAAGAGAGAGGCCAGGAGAAAAGGCTGAGGAAACTGCTGGCAAATGTGAAGGGC
AAGAATGAATGCCCAAGGTGGGCAGCAGGTGAGGAAAGAGTCCCTCACCTCCCTGGAGGA
ACAAGTCTTTGATTTGCTGAAAGGCATCCCGCACAGCCTGGGCGCACTTGGGACTCTGGC
CATAAAAGTCCTGGAGAAGAGACCAAGGTTGCTGCTGCCATTCTTGCACTGGCCTGGGGT
ACCCAAGTCCCCTCACTCACCGCTGTGACATCTCGGAAGAATTGGTAGGAGTCCCCAAGG
CCTGCAACAGCTACAACAGGAGCGCTGGCTGCCAGTGCCCCAGCCACCAGGTGGGGGTAC
TTCATCCTCATGTAGGCACTCAGCATCCCCCCATAACTGGGAGTACAGAGCACAGATCAT
GGTTGTGGGAAGCTGCCCACAACTCAGGCGAGCAGCCTCACTGTCCTCCAGGCTGAGGTG
CTAGGCTGCTCTTTCCCTGCTCAGAACGCCCAAGGGTGGGAAAGAAGGACCTGAAACTGT
CAGGCCCACACACCCTGATCCCAGGGCCAAGGCAGATACAGCCTTCACTGGGAGAAGGCA
CCTGTGGGTGCCCTGCCCTGACCCAGCAATGAAGACATTGCAGAGACAAAGTCAGAAGGA
ATTGTCCCACTAGTGGGAACAACATAGCATACACTGCCTATGAGGTCCACTCAAGGAGGG
CTTCCAGAAGGAGGTAAAGCTAGACCCCGCCCTTCCACATGTGGGGTAGGCATAGGATGT
TGAGACTGTAAGAGACATCTCTTTGGCCCTCCTTGTATAGGGTGTCAATCGGCACAACAG
GGTGGAGCCTTAGAGTAGGGTAAGATTAGGACTCTAGGTTCTCTCATGGGTCCAGATCTG
TCATGAAGGGAGGTCAAGGACCCACCTCCCTCCAAAGGCTATGGTGGGGGCATCATGGAC
CCCAAGGTCCTGCCGCAGGGCCTGGAGCAGCACAGCAAAGTCGGCCAGCGCCTGCTCCAC
AGTCAGCAGCTGTGTATATCCCCGCTGTGT

>86 Get primers
CACCTCCCTGGAGGAACAAGTCTTTGATTTGCTGAAAGGCATCCCGCACAGCCTGGGCGC
ACTTGGGACTCTGGCCATAAAAGTCCTGGAGAAGAGACCAAGGTTGCTGCTGCCATTCTT
GCACTGGCCTGGGGTACCCAAGTCCCCTCACTCACCGCTGTGACATCTCGGAAGAATTGG
TAGGAGTCCCCAAGGCCTGCAACAGCTACAACAGGAGCGCTGGCTGCCAGTGCCCCAGCC
ACCAGGTGGGGGTACTTCATCCTCATGTAGGCACTCAGCATCCCCCCATAACTGGGAGTA
CAGAGCACAGATCATGGTTGTGGGAAGCTGCCCACAACTCAGGCGAGCAGCCTCACTGTC
CTCCAGGCTGAGGTGCTAGGCTGCTCTTTCCCTGCTCAGAACGCCCAAGGGTGGGAAAGA
AGGACCTGAAACTGTCAGGCCCACACACCCTGATCCCAGGGCCAAGGCAGATACAGCCTT
CACTGGGAGAAGGCACCTGTGGGTGCCCTGCCCTGACCCAGCAATGAAGACATTGCAGAG
ACAAAGTCAGAAGGAATTGTCCCACTAGTGGGAACAACATAGCATACACTGCCTATGAGG
TCCACTCAAGGAGGGCTTCCAGAAGGAGGTAAAGCTAGACCCCGCCCTTCCACATGTGGG
GTAGGCATAGGATGTTGAGACTGTAAGAGACATCTCTTTGGCCCTCCTTGTATAGGGTGT
CAATCGGCACAACAGGGTGGAGCCTTAGAGTAGGGTAAGATTAGGACTCTAGGTTCTCTC
ATGGGTCCAGATCTGTCATGAAGGGAGGTCAAGGACCCACCTCCCTCCAAAGGCTATGGT
GGGGGCATCATGGACCCCAAGGTCCTGCCGCAGGGCCTGGAGCAGCACAGCAAAGTCGGC
CAGCGCCTGCTCCACAGTCAGCAGCTGTGTATATCCCCGCTGTGTGGACTGGACACCGAA
CGGAAGCGATTTCCCATAGTACCGCTGCAGAAAGCAGGAAGGGATGGCTAATCCACTCCT
CGGTGCTCCCCACCTCCTTCAACTCAGGGA

>87 Get primers
GCTGCTGCCATTCTTGCACTGGCCTGGGGTACCCAAGTCCCCTCACTCACCGCTGTGACA
TCTCGGAAGAATTGGTAGGAGTCCCCAAGGCCTGCAACAGCTACAACAGGAGCGCTGGCT
GCCAGTGCCCCAGCCACCAGGTGGGGGTACTTCATCCTCATGTAGGCACTCAGCATCCCC
CCATAACTGGGAGTACAGAGCACAGATCATGGTTGTGGGAAGCTGCCCACAACTCAGGCG
AGCAGCCTCACTGTCCTCCAGGCTGAGGTGCTAGGCTGCTCTTTCCCTGCTCAGAACGCC
CAAGGGTGGGAAAGAAGGACCTGAAACTGTCAGGCCCACACACCCTGATCCCAGGGCCAA
GGCAGATACAGCCTTCACTGGGAGAAGGCACCTGTGGGTGCCCTGCCCTGACCCAGCAAT
GAAGACATTGCAGAGACAAAGTCAGAAGGAATTGTCCCACTAGTGGGAACAACATAGCAT
ACACTGCCTATGAGGTCCACTCAAGGAGGGCTTCCAGAAGGAGGTAAAGCTAGACCCCGC
CCTTCCACATGTGGGGTAGGCATAGGATGTTGAGACTGTAAGAGACATCTCTTTGGCCCT
CCTTGTATAGGGTGTCAATCGGCACAACAGGGTGGAGCCTTAGAGTAGGGTAAGATTAGG
ACTCTAGGTTCTCTCATGGGTCCAGATCTGTCATGAAGGGAGGTCAAGGACCCACCTCCC
TCCAAAGGCTATGGTGGGGGCATCATGGACCCCAAGGTCCTGCCGCAGGGCCTGGAGCAG
CACAGCAAAGTCGGCCAGCGCCTGCTCCACAGTCAGCAGCTGTGTATATCCCCGCTGTGT
GGACTGGACACCGAACGGAAGCGATTTCCCATAGTACCGCTGCAGAAAGCAGGAAGGGAT
GGCTAATCCACTCCTCGGTGCTCCCCACCTCCTTCAACTCAGGGACTGCCAGGAACTGTA
CAGGTACCCACGTGCTCAGCAAAGACAAGCAGGGCCTCCTGCTGGGCTGCCAGTTCCACC
ATGAAGCCAGAGTTGTTAGCGAAGGACCAG

>88 Get primers
ACAGGAGCGCTGGCTGCCAGTGCCCCAGCCACCAGGTGGGGGTACTTCATCCTCATGTAG
GCACTCAGCATCCCCCCATAACTGGGAGTACAGAGCACAGATCATGGTTGTGGGAAGCTG
CCCACAACTCAGGCGAGCAGCCTCACTGTCCTCCAGGCTGAGGTGCTAGGCTGCTCTTTC
CCTGCTCAGAACGCCCAAGGGTGGGAAAGAAGGACCTGAAACTGTCAGGCCCACACACCC
TGATCCCAGGGCCAAGGCAGATACAGCCTTCACTGGGAGAAGGCACCTGTGGGTGCCCTG
CCCTGACCCAGCAATGAAGACATTGCAGAGACAAAGTCAGAAGGAATTGTCCCACTAGTG
GGAACAACATAGCATACACTGCCTATGAGGTCCACTCAAGGAGGGCTTCCAGAAGGAGGT
AAAGCTAGACCCCGCCCTTCCACATGTGGGGTAGGCATAGGATGTTGAGACTGTAAGAGA
CATCTCTTTGGCCCTCCTTGTATAGGGTGTCAATCGGCACAACAGGGTGGAGCCTTAGAG
TAGGGTAAGATTAGGACTCTAGGTTCTCTCATGGGTCCAGATCTGTCATGAAGGGAGGTC
AAGGACCCACCTCCCTCCAAAGGCTATGGTGGGGGCATCATGGACCCCAAGGTCCTGCCG
CAGGGCCTGGAGCAGCACAGCAAAGTCGGCCAGCGCCTGCTCCACAGTCAGCAGCTGTGT
ATATCCCCGCTGTGTGGACTGGACACCGAACGGAAGCGATTTCCCATAGTACCGCTGCAG
AAAGCAGGAAGGGATGGCTAATCCACTCCTCGGTGCTCCCCACCTCCTTCAACTCAGGGA
CTGCCAGGAACTGTACAGGTACCCACGTGCTCAGCAAAGACAAGCAGGGCCTCCTGCTGG
GCTGCCAGTTCCACCATGAAGCCAGAGTTGTTAGCGAAGGACCAGATATCCCCCTCATTC
CCTGTGTAGAAAAAGATGGGCCCTTCGCCCATCTTCCAGAACTTATCTGTTGGAAGTAAA
TGAGTTTCCATAAGGCCAGGGAAACGCAGG

>89 Get primers
GGTTGTGGGAAGCTGCCCACAACTCAGGCGAGCAGCCTCACTGTCCTCCAGGCTGAGGTG
CTAGGCTGCTCTTTCCCTGCTCAGAACGCCCAAGGGTGGGAAAGAAGGACCTGAAACTGT
CAGGCCCACACACCCTGATCCCAGGGCCAAGGCAGATACAGCCTTCACTGGGAGAAGGCA
CCTGTGGGTGCCCTGCCCTGACCCAGCAATGAAGACATTGCAGAGACAAAGTCAGAAGGA
ATTGTCCCACTAGTGGGAACAACATAGCATACACTGCCTATGAGGTCCACTCAAGGAGGG
CTTCCAGAAGGAGGTAAAGCTAGACCCCGCCCTTCCACATGTGGGGTAGGCATAGGATGT
TGAGACTGTAAGAGACATCTCTTTGGCCCTCCTTGTATAGGGTGTCAATCGGCACAACAG
GGTGGAGCCTTAGAGTAGGGTAAGATTAGGACTCTAGGTTCTCTCATGGGTCCAGATCTG
TCATGAAGGGAGGTCAAGGACCCACCTCCCTCCAAAGGCTATGGTGGGGGCATCATGGAC
CCCAAGGTCCTGCCGCAGGGCCTGGAGCAGCACAGCAAAGTCGGCCAGCGCCTGCTCCAC
AGTCAGCAGCTGTGTATATCCCCGCTGTGTGGACTGGACACCGAACGGAAGCGATTTCCC
ATAGTACCGCTGCAGAAAGCAGGAAGGGATGGCTAATCCACTCCTCGGTGCTCCCCACCT
CCTTCAACTCAGGGACTGCCAGGAACTGTACAGGTACCCACGTGCTCAGCAAAGACAAGC
AGGGCCTCCTGCTGGGCTGCCAGTTCCACCATGAAGCCAGAGTTGTTAGCGAAGGACCAG
ATATCCCCCTCATTCCCTGTGTAGAAAAAGATGGGCCCTTCGCCCATCTTCCAGAACTTA
TCTGTTGGAAGTAAATGAGTTTCCATAAGGCCAGGGAAACGCAGGTAGGAACCCATGCGG
TCGAGCCAGCACTCACCTGACACTAGGAACCGCTGGCCAAAGGTTTTGTTGCCGAAACTC
TCAAAGTTGAAATGGTCCATGTATTGCTCA

>90 Get primers
AGGACCTGAAACTGTCAGGCCCACACACCCTGATCCCAGGGCCAAGGCAGATACAGCCTT
CACTGGGAGAAGGCACCTGTGGGTGCCCTGCCCTGACCCAGCAATGAAGACATTGCAGAG
ACAAAGTCAGAAGGAATTGTCCCACTAGTGGGAACAACATAGCATACACTGCCTATGAGG
TCCACTCAAGGAGGGCTTCCAGAAGGAGGTAAAGCTAGACCCCGCCCTTCCACATGTGGG
GTAGGCATAGGATGTTGAGACTGTAAGAGACATCTCTTTGGCCCTCCTTGTATAGGGTGT
CAATCGGCACAACAGGGTGGAGCCTTAGAGTAGGGTAAGATTAGGACTCTAGGTTCTCTC
ATGGGTCCAGATCTGTCATGAAGGGAGGTCAAGGACCCACCTCCCTCCAAAGGCTATGGT
GGGGGCATCATGGACCCCAAGGTCCTGCCGCAGGGCCTGGAGCAGCACAGCAAAGTCGGC
CAGCGCCTGCTCCACAGTCAGCAGCTGTGTATATCCCCGCTGTGTGGACTGGACACCGAA
CGGAAGCGATTTCCCATAGTACCGCTGCAGAAAGCAGGAAGGGATGGCTAATCCACTCCT
CGGTGCTCCCCACCTCCTTCAACTCAGGGACTGCCAGGAACTGTACAGGTACCCACGTGC
TCAGCAAAGACAAGCAGGGCCTCCTGCTGGGCTGCCAGTTCCACCATGAAGCCAGAGTTG
TTAGCGAAGGACCAGATATCCCCCTCATTCCCTGTGTAGAAAAAGATGGGCCCTTCGCCC
ATCTTCCAGAACTTATCTGTTGGAAGTAAATGAGTTTCCATAAGGCCAGGGAAACGCAGG
TAGGAACCCATGCGGTCGAGCCAGCACTCACCTGACACTAGGAACCGCTGGCCAAAGGTT
TTGTTGCCGAAACTCTCAAAGTTGAAATGGTCCATGTATTGCTCAAAATAATTCTCATGA
AAGTCAGGGTCTAGAACTCTGTCGGCTGAGGGCAGGTGCAGAGACTCAGGAGCTGGTTGG
GATCATCAGGGATCTAGGCGGGTCAGGAGG

>91 Get primers
GAAGACATTGCAGAGACAAAGTCAGAAGGAATTGTCCCACTAGTGGGAACAACATAGCAT
ACACTGCCTATGAGGTCCACTCAAGGAGGGCTTCCAGAAGGAGGTAAAGCTAGACCCCGC
CCTTCCACATGTGGGGTAGGCATAGGATGTTGAGACTGTAAGAGACATCTCTTTGGCCCT
CCTTGTATAGGGTGTCAATCGGCACAACAGGGTGGAGCCTTAGAGTAGGGTAAGATTAGG
ACTCTAGGTTCTCTCATGGGTCCAGATCTGTCATGAAGGGAGGTCAAGGACCCACCTCCC
TCCAAAGGCTATGGTGGGGGCATCATGGACCCCAAGGTCCTGCCGCAGGGCCTGGAGCAG
CACAGCAAAGTCGGCCAGCGCCTGCTCCACAGTCAGCAGCTGTGTATATCCCCGCTGTGT
GGACTGGACACCGAACGGAAGCGATTTCCCATAGTACCGCTGCAGAAAGCAGGAAGGGAT
GGCTAATCCACTCCTCGGTGCTCCCCACCTCCTTCAACTCAGGGACTGCCAGGAACTGTA
CAGGTACCCACGTGCTCAGCAAAGACAAGCAGGGCCTCCTGCTGGGCTGCCAGTTCCACC
ATGAAGCCAGAGTTGTTAGCGAAGGACCAGATATCCCCCTCATTCCCTGTGTAGAAAAAG
ATGGGCCCTTCGCCCATCTTCCAGAACTTATCTGTTGGAAGTAAATGAGTTTCCATAAGG
CCAGGGAAACGCAGGTAGGAACCCATGCGGTCGAGCCAGCACTCACCTGACACTAGGAAC
CGCTGGCCAAAGGTTTTGTTGCCGAAACTCTCAAAGTTGAAATGGTCCATGTATTGCTCA
AAATAATTCTCATGAAAGTCAGGGTCTAGAACTCTGTCGGCTGAGGGCAGGTGCAGAGAC
TCAGGAGCTGGTTGGGATCATCAGGGATCTAGGCGGGTCAGGAGGAAGGGCAGCCAGTCT
GTACTCACCTCTGGCCTGGAGGTTGCACAGTCCCAGTGACAGCAGCAGGACCAGGATCCA
GGAGGGGACACCATGGTCCACAGGGTAACA

>92 Get primers
AAAGCTAGACCCCGCCCTTCCACATGTGGGGTAGGCATAGGATGTTGAGACTGTAAGAGA
CATCTCTTTGGCCCTCCTTGTATAGGGTGTCAATCGGCACAACAGGGTGGAGCCTTAGAG
TAGGGTAAGATTAGGACTCTAGGTTCTCTCATGGGTCCAGATCTGTCATGAAGGGAGGTC
AAGGACCCACCTCCCTCCAAAGGCTATGGTGGGGGCATCATGGACCCCAAGGTCCTGCCG
CAGGGCCTGGAGCAGCACAGCAAAGTCGGCCAGCGCCTGCTCCACAGTCAGCAGCTGTGT
ATATCCCCGCTGTGTGGACTGGACACCGAACGGAAGCGATTTCCCATAGTACCGCTGCAG
AAAGCAGGAAGGGATGGCTAATCCACTCCTCGGTGCTCCCCACCTCCTTCAACTCAGGGA
CTGCCAGGAACTGTACAGGTACCCACGTGCTCAGCAAAGACAAGCAGGGCCTCCTGCTGG
GCTGCCAGTTCCACCATGAAGCCAGAGTTGTTAGCGAAGGACCAGATATCCCCCTCATTC
CCTGTGTAGAAAAAGATGGGCCCTTCGCCCATCTTCCAGAACTTATCTGTTGGAAGTAAA
TGAGTTTCCATAAGGCCAGGGAAACGCAGGTAGGAACCCATGCGGTCGAGCCAGCACTCA
CCTGACACTAGGAACCGCTGGCCAAAGGTTTTGTTGCCGAAACTCTCAAAGTTGAAATGG
TCCATGTATTGCTCAAAATAATTCTCATGAAAGTCAGGGTCTAGAACTCTGTCGGCTGAG
GGCAGGTGCAGAGACTCAGGAGCTGGTTGGGATCATCAGGGATCTAGGCGGGTCAGGAGG
AAGGGCAGCCAGTCTGTACTCACCTCTGGCCTGGAGGTTGCACAGTCCCAGTGACAGCAG
CAGGACCAGGATCCAGGAGGGGACACCATGGTCCACAGGGTAACAAGGATGGAAGTTCAT
GCTTGATTCTGAGCCGGGCGCTGACTGTCATGTGATTTGGTCACATGACCGACACAACGG
GCGGGGCAGCATCACGTGATAGTCTGGCGG

>93 Get primers
GGTGGAGCCTTAGAGTAGGGTAAGATTAGGACTCTAGGTTCTCTCATGGGTCCAGATCTG
TCATGAAGGGAGGTCAAGGACCCACCTCCCTCCAAAGGCTATGGTGGGGGCATCATGGAC
CCCAAGGTCCTGCCGCAGGGCCTGGAGCAGCACAGCAAAGTCGGCCAGCGCCTGCTCCAC
AGTCAGCAGCTGTGTATATCCCCGCTGTGTGGACTGGACACCGAACGGAAGCGATTTCCC
ATAGTACCGCTGCAGAAAGCAGGAAGGGATGGCTAATCCACTCCTCGGTGCTCCCCACCT
CCTTCAACTCAGGGACTGCCAGGAACTGTACAGGTACCCACGTGCTCAGCAAAGACAAGC
AGGGCCTCCTGCTGGGCTGCCAGTTCCACCATGAAGCCAGAGTTGTTAGCGAAGGACCAG
ATATCCCCCTCATTCCCTGTGTAGAAAAAGATGGGCCCTTCGCCCATCTTCCAGAACTTA
TCTGTTGGAAGTAAATGAGTTTCCATAAGGCCAGGGAAACGCAGGTAGGAACCCATGCGG
TCGAGCCAGCACTCACCTGACACTAGGAACCGCTGGCCAAAGGTTTTGTTGCCGAAACTC
TCAAAGTTGAAATGGTCCATGTATTGCTCAAAATAATTCTCATGAAAGTCAGGGTCTAGA
ACTCTGTCGGCTGAGGGCAGGTGCAGAGACTCAGGAGCTGGTTGGGATCATCAGGGATCT
AGGCGGGTCAGGAGGAAGGGCAGCCAGTCTGTACTCACCTCTGGCCTGGAGGTTGCACAG
TCCCAGTGACAGCAGCAGGACCAGGATCCAGGAGGGGACACCATGGTCCACAGGGTAACA
AGGATGGAAGTTCATGCTTGATTCTGAGCCGGGCGCTGACTGTCATGTGATTTGGTCACA
TGACCGACACAACGGGCGGGGCAGCATCACGTGATAGTCTGGCGGGGGCTGTCCTACTGT
GGCTGGATTCTAGTTGGAGGATCAGCCTACTCTTCTTCAGTTTCCCGGTTCCTCCAAATT
TCTGGGCTCCTACTTGTTTCCACAGAGATG

>94 Get primers
GGGGGCATCATGGACCCCAAGGTCCTGCCGCAGGGCCTGGAGCAGCACAGCAAAGTCGGC
CAGCGCCTGCTCCACAGTCAGCAGCTGTGTATATCCCCGCTGTGTGGACTGGACACCGAA
CGGAAGCGATTTCCCATAGTACCGCTGCAGAAAGCAGGAAGGGATGGCTAATCCACTCCT
CGGTGCTCCCCACCTCCTTCAACTCAGGGACTGCCAGGAACTGTACAGGTACCCACGTGC
TCAGCAAAGACAAGCAGGGCCTCCTGCTGGGCTGCCAGTTCCACCATGAAGCCAGAGTTG
TTAGCGAAGGACCAGATATCCCCCTCATTCCCTGTGTAGAAAAAGATGGGCCCTTCGCCC
ATCTTCCAGAACTTATCTGTTGGAAGTAAATGAGTTTCCATAAGGCCAGGGAAACGCAGG
TAGGAACCCATGCGGTCGAGCCAGCACTCACCTGACACTAGGAACCGCTGGCCAAAGGTT
TTGTTGCCGAAACTCTCAAAGTTGAAATGGTCCATGTATTGCTCAAAATAATTCTCATGA
AAGTCAGGGTCTAGAACTCTGTCGGCTGAGGGCAGGTGCAGAGACTCAGGAGCTGGTTGG
GATCATCAGGGATCTAGGCGGGTCAGGAGGAAGGGCAGCCAGTCTGTACTCACCTCTGGC
CTGGAGGTTGCACAGTCCCAGTGACAGCAGCAGGACCAGGATCCAGGAGGGGACACCATG
GTCCACAGGGTAACAAGGATGGAAGTTCATGCTTGATTCTGAGCCGGGCGCTGACTGTCA
TGTGATTTGGTCACATGACCGACACAACGGGCGGGGCAGCATCACGTGATAGTCTGGCGG
GGGCTGTCCTACTGTGGCTGGATTCTAGTTGGAGGATCAGCCTACTCTTCTTCAGTTTCC
CGGTTCCTCCAAATTTCTGGGCTCCTACTTGTTTCCACAGAGATGGATACTGTGGAGGTC
CAGGAAGCAGAGAGATGGCTAAGGCTCATCAGGACCGTATGATCTCCCAAGTGTCCAGCT
ACTGAGTACCACAAGGTGATGGGTGGGAGG

>105 Get primers
GGCAGCAGGTGAGGAAAGAGTCCCTCACCTCCCTGGAGGAACAAGTCTTTGATTTGCTGA
AAGGCATCCCGCACAGCCTGGGCGCACTTGGGACTCTGGCCATAAAAGTCCTGGAGAAGA
GACCAAGGTTGCTGCTGCCATTCTTGCACTGGCCTGGGGTACCCAAGTCCCCTCACTCAC
CGCTGTGACATCTCGGAAGAATTGGTAGGAGTCCCCAAGGCCTGCAACAGCTACAACAGG
AGCGCTGGCTGCCAGTGCCCCAGCCACCAGGTGGGGGTACTTCATCCTCATGTAGGCACT
CAGCATCCCCCCATAACTGGGAGTACAGAGCACAGATCATGGTTGTGGGAAGCTGCCCAC
AACTCAGGCGAGCAGCCTCACTGTCCTCCAGGCTGAGGTGCTAGGCTGCTCTTTCCCTGC
TCAGAACGCCCAAGGGTGGGAAAGAAGGACCTGAAACTGTCAGGCCCACACACCCTGATC
CCAGGGCCAAGGCAGATACAGCCTTCACTGGGAGAAGGCACCTGTGGGTGCCCTGCCCTG
ACCCAGCAATGAAGACATTGCAGAGACAAAGTCAGAAGGAATTGTCCCACTAGTGGGAAC
AACATAGCATACACTGCCTATGAGGTCCACTCAAGGAGGGCTTCCAGAAGGAGGTAAAGC
TAGACCCCGCCCTTCCACATGTGGGGTAGGCATAGGATGTTGAGACTGTAAGAGACATCT
CTTTGGCCCTCCTTGTATAGGGTGTCAATCGGCACAACAGGGTGGAGCCTTAGAGTAGGG
TAAGATTAGGACTCTAGGTTCTCTCATGGGTCCAGATCTGTCATGAAGGGAGGTCAAGGA
CCCACCTCCCTCCAAAGGCTATGGTGGGGGCATCATGGACCCCAAGGTCCTGCCGCAGGG
CCTGGAGCAGCACAGCAAAGTCGGCCAGCGCCTGCTCCACAGTCAGCAGCTGTGTATATC
CCCGCTGTGTGGACTGGACACCGAACGGAAGCGATTTCCC

>106 Get primers
CATAAAAGTCCTGGAGAAGAGACCAAGGTTGCTGCTGCCATTCTTGCACTGGCCTGGGGT
ACCCAAGTCCCCTCACTCACCGCTGTGACATCTCGGAAGAATTGGTAGGAGTCCCCAAGG
CCTGCAACAGCTACAACAGGAGCGCTGGCTGCCAGTGCCCCAGCCACCAGGTGGGGGTAC
TTCATCCTCATGTAGGCACTCAGCATCCCCCCATAACTGGGAGTACAGAGCACAGATCAT
GGTTGTGGGAAGCTGCCCACAACTCAGGCGAGCAGCCTCACTGTCCTCCAGGCTGAGGTG
CTAGGCTGCTCTTTCCCTGCTCAGAACGCCCAAGGGTGGGAAAGAAGGACCTGAAACTGT
CAGGCCCACACACCCTGATCCCAGGGCCAAGGCAGATACAGCCTTCACTGGGAGAAGGCA
CCTGTGGGTGCCCTGCCCTGACCCAGCAATGAAGACATTGCAGAGACAAAGTCAGAAGGA
ATTGTCCCACTAGTGGGAACAACATAGCATACACTGCCTATGAGGTCCACTCAAGGAGGG
CTTCCAGAAGGAGGTAAAGCTAGACCCCGCCCTTCCACATGTGGGGTAGGCATAGGATGT
TGAGACTGTAAGAGACATCTCTTTGGCCCTCCTTGTATAGGGTGTCAATCGGCACAACAG
GGTGGAGCCTTAGAGTAGGGTAAGATTAGGACTCTAGGTTCTCTCATGGGTCCAGATCTG
TCATGAAGGGAGGTCAAGGACCCACCTCCCTCCAAAGGCTATGGTGGGGGCATCATGGAC
CCCAAGGTCCTGCCGCAGGGCCTGGAGCAGCACAGCAAAGTCGGCCAGCGCCTGCTCCAC
AGTCAGCAGCTGTGTATATCCCCGCTGTGTGGACTGGACACCGAACGGAAGCGATTTCCC
ATAGTACCGCTGCAGAAAGCAGGAAGGGATGGCTAATCCACTCCTCGGTGCTCCCCACCT
CCTTCAACTCAGGGACTGCCAGGAACTGTACAGGTACCCA

>107 Get primers
ATTGGTAGGAGTCCCCAAGGCCTGCAACAGCTACAACAGGAGCGCTGGCTGCCAGTGCCC
CAGCCACCAGGTGGGGGTACTTCATCCTCATGTAGGCACTCAGCATCCCCCCATAACTGG
GAGTACAGAGCACAGATCATGGTTGTGGGAAGCTGCCCACAACTCAGGCGAGCAGCCTCA
CTGTCCTCCAGGCTGAGGTGCTAGGCTGCTCTTTCCCTGCTCAGAACGCCCAAGGGTGGG
AAAGAAGGACCTGAAACTGTCAGGCCCACACACCCTGATCCCAGGGCCAAGGCAGATACA
GCCTTCACTGGGAGAAGGCACCTGTGGGTGCCCTGCCCTGACCCAGCAATGAAGACATTG
CAGAGACAAAGTCAGAAGGAATTGTCCCACTAGTGGGAACAACATAGCATACACTGCCTA
TGAGGTCCACTCAAGGAGGGCTTCCAGAAGGAGGTAAAGCTAGACCCCGCCCTTCCACAT
GTGGGGTAGGCATAGGATGTTGAGACTGTAAGAGACATCTCTTTGGCCCTCCTTGTATAG
GGTGTCAATCGGCACAACAGGGTGGAGCCTTAGAGTAGGGTAAGATTAGGACTCTAGGTT
CTCTCATGGGTCCAGATCTGTCATGAAGGGAGGTCAAGGACCCACCTCCCTCCAAAGGCT
ATGGTGGGGGCATCATGGACCCCAAGGTCCTGCCGCAGGGCCTGGAGCAGCACAGCAAAG
TCGGCCAGCGCCTGCTCCACAGTCAGCAGCTGTGTATATCCCCGCTGTGTGGACTGGACA
CCGAACGGAAGCGATTTCCCATAGTACCGCTGCAGAAAGCAGGAAGGGATGGCTAATCCA
CTCCTCGGTGCTCCCCACCTCCTTCAACTCAGGGACTGCCAGGAACTGTACAGGTACCCA
CGTGCTCAGCAAAGACAAGCAGGGCCTCCTGCTGGGCTGCCAGTTCCACCATGAAGCCAG
AGTTGTTAGCGAAGGACCAGATATCCCCCTCATTCCCTGT

>108 Get primers
CAGCATCCCCCCATAACTGGGAGTACAGAGCACAGATCATGGTTGTGGGAAGCTGCCCAC
AACTCAGGCGAGCAGCCTCACTGTCCTCCAGGCTGAGGTGCTAGGCTGCTCTTTCCCTGC
TCAGAACGCCCAAGGGTGGGAAAGAAGGACCTGAAACTGTCAGGCCCACACACCCTGATC
CCAGGGCCAAGGCAGATACAGCCTTCACTGGGAGAAGGCACCTGTGGGTGCCCTGCCCTG
ACCCAGCAATGAAGACATTGCAGAGACAAAGTCAGAAGGAATTGTCCCACTAGTGGGAAC
AACATAGCATACACTGCCTATGAGGTCCACTCAAGGAGGGCTTCCAGAAGGAGGTAAAGC
TAGACCCCGCCCTTCCACATGTGGGGTAGGCATAGGATGTTGAGACTGTAAGAGACATCT
CTTTGGCCCTCCTTGTATAGGGTGTCAATCGGCACAACAGGGTGGAGCCTTAGAGTAGGG
TAAGATTAGGACTCTAGGTTCTCTCATGGGTCCAGATCTGTCATGAAGGGAGGTCAAGGA
CCCACCTCCCTCCAAAGGCTATGGTGGGGGCATCATGGACCCCAAGGTCCTGCCGCAGGG
CCTGGAGCAGCACAGCAAAGTCGGCCAGCGCCTGCTCCACAGTCAGCAGCTGTGTATATC
CCCGCTGTGTGGACTGGACACCGAACGGAAGCGATTTCCCATAGTACCGCTGCAGAAAGC
AGGAAGGGATGGCTAATCCACTCCTCGGTGCTCCCCACCTCCTTCAACTCAGGGACTGCC
AGGAACTGTACAGGTACCCACGTGCTCAGCAAAGACAAGCAGGGCCTCCTGCTGGGCTGC
CAGTTCCACCATGAAGCCAGAGTTGTTAGCGAAGGACCAGATATCCCCCTCATTCCCTGT
GTAGAAAAAGATGGGCCCTTCGCCCATCTTCCAGAACTTATCTGTTGGAAGTAAATGAGT
TTCCATAAGGCCAGGGAAACGCAGGTAGGAACCCATGCGG

>109 Get primers
CTAGGCTGCTCTTTCCCTGCTCAGAACGCCCAAGGGTGGGAAAGAAGGACCTGAAACTGT
CAGGCCCACACACCCTGATCCCAGGGCCAAGGCAGATACAGCCTTCACTGGGAGAAGGCA
CCTGTGGGTGCCCTGCCCTGACCCAGCAATGAAGACATTGCAGAGACAAAGTCAGAAGGA
ATTGTCCCACTAGTGGGAACAACATAGCATACACTGCCTATGAGGTCCACTCAAGGAGGG
CTTCCAGAAGGAGGTAAAGCTAGACCCCGCCCTTCCACATGTGGGGTAGGCATAGGATGT
TGAGACTGTAAGAGACATCTCTTTGGCCCTCCTTGTATAGGGTGTCAATCGGCACAACAG
GGTGGAGCCTTAGAGTAGGGTAAGATTAGGACTCTAGGTTCTCTCATGGGTCCAGATCTG
TCATGAAGGGAGGTCAAGGACCCACCTCCCTCCAAAGGCTATGGTGGGGGCATCATGGAC
CCCAAGGTCCTGCCGCAGGGCCTGGAGCAGCACAGCAAAGTCGGCCAGCGCCTGCTCCAC
AGTCAGCAGCTGTGTATATCCCCGCTGTGTGGACTGGACACCGAACGGAAGCGATTTCCC
ATAGTACCGCTGCAGAAAGCAGGAAGGGATGGCTAATCCACTCCTCGGTGCTCCCCACCT
CCTTCAACTCAGGGACTGCCAGGAACTGTACAGGTACCCACGTGCTCAGCAAAGACAAGC
AGGGCCTCCTGCTGGGCTGCCAGTTCCACCATGAAGCCAGAGTTGTTAGCGAAGGACCAG
ATATCCCCCTCATTCCCTGTGTAGAAAAAGATGGGCCCTTCGCCCATCTTCCAGAACTTA
TCTGTTGGAAGTAAATGAGTTTCCATAAGGCCAGGGAAACGCAGGTAGGAACCCATGCGG
TCGAGCCAGCACTCACCTGACACTAGGAACCGCTGGCCAAAGGTTTTGTTGCCGAAACTC
TCAAAGTTGAAATGGTCCATGTATTGCTCAAAATAATTCT

>110 Get primers
GCCTTCACTGGGAGAAGGCACCTGTGGGTGCCCTGCCCTGACCCAGCAATGAAGACATTG
CAGAGACAAAGTCAGAAGGAATTGTCCCACTAGTGGGAACAACATAGCATACACTGCCTA
TGAGGTCCACTCAAGGAGGGCTTCCAGAAGGAGGTAAAGCTAGACCCCGCCCTTCCACAT
GTGGGGTAGGCATAGGATGTTGAGACTGTAAGAGACATCTCTTTGGCCCTCCTTGTATAG
GGTGTCAATCGGCACAACAGGGTGGAGCCTTAGAGTAGGGTAAGATTAGGACTCTAGGTT
CTCTCATGGGTCCAGATCTGTCATGAAGGGAGGTCAAGGACCCACCTCCCTCCAAAGGCT
ATGGTGGGGGCATCATGGACCCCAAGGTCCTGCCGCAGGGCCTGGAGCAGCACAGCAAAG
TCGGCCAGCGCCTGCTCCACAGTCAGCAGCTGTGTATATCCCCGCTGTGTGGACTGGACA
CCGAACGGAAGCGATTTCCCATAGTACCGCTGCAGAAAGCAGGAAGGGATGGCTAATCCA
CTCCTCGGTGCTCCCCACCTCCTTCAACTCAGGGACTGCCAGGAACTGTACAGGTACCCA
CGTGCTCAGCAAAGACAAGCAGGGCCTCCTGCTGGGCTGCCAGTTCCACCATGAAGCCAG
AGTTGTTAGCGAAGGACCAGATATCCCCCTCATTCCCTGTGTAGAAAAAGATGGGCCCTT
CGCCCATCTTCCAGAACTTATCTGTTGGAAGTAAATGAGTTTCCATAAGGCCAGGGAAAC
GCAGGTAGGAACCCATGCGGTCGAGCCAGCACTCACCTGACACTAGGAACCGCTGGCCAA
AGGTTTTGTTGCCGAAACTCTCAAAGTTGAAATGGTCCATGTATTGCTCAAAATAATTCT
CATGAAAGTCAGGGTCTAGAACTCTGTCGGCTGAGGGCAGGTGCAGAGACTCAGGAGCTG
GTTGGGATCATCAGGGATCTAGGCGGGTCAGGAGGAAGGG

>111 Get primers
AACATAGCATACACTGCCTATGAGGTCCACTCAAGGAGGGCTTCCAGAAGGAGGTAAAGC
TAGACCCCGCCCTTCCACATGTGGGGTAGGCATAGGATGTTGAGACTGTAAGAGACATCT
CTTTGGCCCTCCTTGTATAGGGTGTCAATCGGCACAACAGGGTGGAGCCTTAGAGTAGGG
TAAGATTAGGACTCTAGGTTCTCTCATGGGTCCAGATCTGTCATGAAGGGAGGTCAAGGA
CCCACCTCCCTCCAAAGGCTATGGTGGGGGCATCATGGACCCCAAGGTCCTGCCGCAGGG
CCTGGAGCAGCACAGCAAAGTCGGCCAGCGCCTGCTCCACAGTCAGCAGCTGTGTATATC
CCCGCTGTGTGGACTGGACACCGAACGGAAGCGATTTCCCATAGTACCGCTGCAGAAAGC
AGGAAGGGATGGCTAATCCACTCCTCGGTGCTCCCCACCTCCTTCAACTCAGGGACTGCC
AGGAACTGTACAGGTACCCACGTGCTCAGCAAAGACAAGCAGGGCCTCCTGCTGGGCTGC
CAGTTCCACCATGAAGCCAGAGTTGTTAGCGAAGGACCAGATATCCCCCTCATTCCCTGT
GTAGAAAAAGATGGGCCCTTCGCCCATCTTCCAGAACTTATCTGTTGGAAGTAAATGAGT
TTCCATAAGGCCAGGGAAACGCAGGTAGGAACCCATGCGGTCGAGCCAGCACTCACCTGA
CACTAGGAACCGCTGGCCAAAGGTTTTGTTGCCGAAACTCTCAAAGTTGAAATGGTCCAT
GTATTGCTCAAAATAATTCTCATGAAAGTCAGGGTCTAGAACTCTGTCGGCTGAGGGCAG
GTGCAGAGACTCAGGAGCTGGTTGGGATCATCAGGGATCTAGGCGGGTCAGGAGGAAGGG
CAGCCAGTCTGTACTCACCTCTGGCCTGGAGGTTGCACAGTCCCAGTGACAGCAGCAGGA
CCAGGATCCAGGAGGGGACACCATGGTCCACAGGGTAACA

>112 Get primers
TGAGACTGTAAGAGACATCTCTTTGGCCCTCCTTGTATAGGGTGTCAATCGGCACAACAG
GGTGGAGCCTTAGAGTAGGGTAAGATTAGGACTCTAGGTTCTCTCATGGGTCCAGATCTG
TCATGAAGGGAGGTCAAGGACCCACCTCCCTCCAAAGGCTATGGTGGGGGCATCATGGAC
CCCAAGGTCCTGCCGCAGGGCCTGGAGCAGCACAGCAAAGTCGGCCAGCGCCTGCTCCAC
AGTCAGCAGCTGTGTATATCCCCGCTGTGTGGACTGGACACCGAACGGAAGCGATTTCCC
ATAGTACCGCTGCAGAAAGCAGGAAGGGATGGCTAATCCACTCCTCGGTGCTCCCCACCT
CCTTCAACTCAGGGACTGCCAGGAACTGTACAGGTACCCACGTGCTCAGCAAAGACAAGC
AGGGCCTCCTGCTGGGCTGCCAGTTCCACCATGAAGCCAGAGTTGTTAGCGAAGGACCAG
ATATCCCCCTCATTCCCTGTGTAGAAAAAGATGGGCCCTTCGCCCATCTTCCAGAACTTA
TCTGTTGGAAGTAAATGAGTTTCCATAAGGCCAGGGAAACGCAGGTAGGAACCCATGCGG
TCGAGCCAGCACTCACCTGACACTAGGAACCGCTGGCCAAAGGTTTTGTTGCCGAAACTC
TCAAAGTTGAAATGGTCCATGTATTGCTCAAAATAATTCTCATGAAAGTCAGGGTCTAGA
ACTCTGTCGGCTGAGGGCAGGTGCAGAGACTCAGGAGCTGGTTGGGATCATCAGGGATCT
AGGCGGGTCAGGAGGAAGGGCAGCCAGTCTGTACTCACCTCTGGCCTGGAGGTTGCACAG
TCCCAGTGACAGCAGCAGGACCAGGATCCAGGAGGGGACACCATGGTCCACAGGGTAACA
AGGATGGAAGTTCATGCTTGATTCTGAGCCGGGCGCTGACTGTCATGTGATTTGGTCACA
TGACCGACACAACGGGCGGGGCAGCATCACGTGATAGTCT

>113 Get primers
CTCTCATGGGTCCAGATCTGTCATGAAGGGAGGTCAAGGACCCACCTCCCTCCAAAGGCT
ATGGTGGGGGCATCATGGACCCCAAGGTCCTGCCGCAGGGCCTGGAGCAGCACAGCAAAG
TCGGCCAGCGCCTGCTCCACAGTCAGCAGCTGTGTATATCCCCGCTGTGTGGACTGGACA
CCGAACGGAAGCGATTTCCCATAGTACCGCTGCAGAAAGCAGGAAGGGATGGCTAATCCA
CTCCTCGGTGCTCCCCACCTCCTTCAACTCAGGGACTGCCAGGAACTGTACAGGTACCCA
CGTGCTCAGCAAAGACAAGCAGGGCCTCCTGCTGGGCTGCCAGTTCCACCATGAAGCCAG
AGTTGTTAGCGAAGGACCAGATATCCCCCTCATTCCCTGTGTAGAAAAAGATGGGCCCTT
CGCCCATCTTCCAGAACTTATCTGTTGGAAGTAAATGAGTTTCCATAAGGCCAGGGAAAC
GCAGGTAGGAACCCATGCGGTCGAGCCAGCACTCACCTGACACTAGGAACCGCTGGCCAA
AGGTTTTGTTGCCGAAACTCTCAAAGTTGAAATGGTCCATGTATTGCTCAAAATAATTCT
CATGAAAGTCAGGGTCTAGAACTCTGTCGGCTGAGGGCAGGTGCAGAGACTCAGGAGCTG
GTTGGGATCATCAGGGATCTAGGCGGGTCAGGAGGAAGGGCAGCCAGTCTGTACTCACCT
CTGGCCTGGAGGTTGCACAGTCCCAGTGACAGCAGCAGGACCAGGATCCAGGAGGGGACA
CCATGGTCCACAGGGTAACAAGGATGGAAGTTCATGCTTGATTCTGAGCCGGGCGCTGAC
TGTCATGTGATTTGGTCACATGACCGACACAACGGGCGGGGCAGCATCACGTGATAGTCT
GGCGGGGGCTGTCCTACTGTGGCTGGATTCTAGTTGGAGGATCAGCCTACTCTTCTTCAG
TTTCCCGGTTCCTCCAAATTTCTGGGCTCCTACTTGTTTC

>127 Get primers
GCACAGCCTGGGCGCACTTGGGACTCTGGCCATAAAAGTCCTGGAGAAGAGACCAAGGTT
GCTGCTGCCATTCTTGCACTGGCCTGGGGTACCCAAGTCCCCTCACTCACCGCTGTGACA
TCTCGGAAGAATTGGTAGGAGTCCCCAAGGCCTGCAACAGCTACAACAGGAGCGCTGGCT
GCCAGTGCCCCAGCCACCAGGTGGGGGTACTTCATCCTCATGTAGGCACTCAGCATCCCC
CCATAACTGGGAGTACAGAGCACAGATCATGGTTGTGGGAAGCTGCCCACAACTCAGGCG
AGCAGCCTCACTGTCCTCCAGGCTGAGGTGCTAGGCTGCTCTTTCCCTGCTCAGAACGCC
CAAGGGTGGGAAAGAAGGACCTGAAACTGTCAGGCCCACACACCCTGATCCCAGGGCCAA
GGCAGATACAGCCTTCACTGGGAGAAGGCACCTGTGGGTGCCCTGCCCTGACCCAGCAAT
GAAGACATTGCAGAGACAAAGTCAGAAGGAATTGTCCCACTAGTGGGAACAACATAGCAT
ACACTGCCTATGAGGTCCACTCAAGGAGGGCTTCCAGAAGGAGGTAAAGCTAGACCCCGC
CCTTCCACATGTGGGGTAGGCATAGGATGTTGAGACTGTAAGAGACATCTCTTTGGCCCT
CCTTGTATAGGGTGTCAATCGGCACAACAGGGTGGAGCCTTAGAGTAGGGTAAGATTAGG
ACTCTAGGTTCTCTCATGGGTCCAGATCTGTCATGAAGGGAGGTCAAGGACCCACCTCCC
TCCAAAGGCTATGGTGGGGGCATCATGGACCCCAAGGTCCTGCCGCAGGGCCTGGAGCAG
CACAGCAAAGTCGGCCAGCGCCTGCTCCACAGTCAGCAGCTGTGTATATCCCCGCTGTGT
GGACTGGACACCGAACGGAAGCGATTTCCCATAGTACCGCTGCAGAAAGC

>128 Get primers
AGTCCCCTCACTCACCGCTGTGACATCTCGGAAGAATTGGTAGGAGTCCCCAAGGCCTGC
AACAGCTACAACAGGAGCGCTGGCTGCCAGTGCCCCAGCCACCAGGTGGGGGTACTTCAT
CCTCATGTAGGCACTCAGCATCCCCCCATAACTGGGAGTACAGAGCACAGATCATGGTTG
TGGGAAGCTGCCCACAACTCAGGCGAGCAGCCTCACTGTCCTCCAGGCTGAGGTGCTAGG
CTGCTCTTTCCCTGCTCAGAACGCCCAAGGGTGGGAAAGAAGGACCTGAAACTGTCAGGC
CCACACACCCTGATCCCAGGGCCAAGGCAGATACAGCCTTCACTGGGAGAAGGCACCTGT
GGGTGCCCTGCCCTGACCCAGCAATGAAGACATTGCAGAGACAAAGTCAGAAGGAATTGT
CCCACTAGTGGGAACAACATAGCATACACTGCCTATGAGGTCCACTCAAGGAGGGCTTCC
AGAAGGAGGTAAAGCTAGACCCCGCCCTTCCACATGTGGGGTAGGCATAGGATGTTGAGA
CTGTAAGAGACATCTCTTTGGCCCTCCTTGTATAGGGTGTCAATCGGCACAACAGGGTGG
AGCCTTAGAGTAGGGTAAGATTAGGACTCTAGGTTCTCTCATGGGTCCAGATCTGTCATG
AAGGGAGGTCAAGGACCCACCTCCCTCCAAAGGCTATGGTGGGGGCATCATGGACCCCAA
GGTCCTGCCGCAGGGCCTGGAGCAGCACAGCAAAGTCGGCCAGCGCCTGCTCCACAGTCA
GCAGCTGTGTATATCCCCGCTGTGTGGACTGGACACCGAACGGAAGCGATTTCCCATAGT
ACCGCTGCAGAAAGCAGGAAGGGATGGCTAATCCACTCCTCGGTGCTCCCCACCTCCTTC
AACTCAGGGACTGCCAGGAACTGTACAGGTACCCACGTGCTCAGCAAAGA

>129 Get primers
CAGCCACCAGGTGGGGGTACTTCATCCTCATGTAGGCACTCAGCATCCCCCCATAACTGG
GAGTACAGAGCACAGATCATGGTTGTGGGAAGCTGCCCACAACTCAGGCGAGCAGCCTCA
CTGTCCTCCAGGCTGAGGTGCTAGGCTGCTCTTTCCCTGCTCAGAACGCCCAAGGGTGGG
AAAGAAGGACCTGAAACTGTCAGGCCCACACACCCTGATCCCAGGGCCAAGGCAGATACA
GCCTTCACTGGGAGAAGGCACCTGTGGGTGCCCTGCCCTGACCCAGCAATGAAGACATTG
CAGAGACAAAGTCAGAAGGAATTGTCCCACTAGTGGGAACAACATAGCATACACTGCCTA
TGAGGTCCACTCAAGGAGGGCTTCCAGAAGGAGGTAAAGCTAGACCCCGCCCTTCCACAT
GTGGGGTAGGCATAGGATGTTGAGACTGTAAGAGACATCTCTTTGGCCCTCCTTGTATAG
GGTGTCAATCGGCACAACAGGGTGGAGCCTTAGAGTAGGGTAAGATTAGGACTCTAGGTT
CTCTCATGGGTCCAGATCTGTCATGAAGGGAGGTCAAGGACCCACCTCCCTCCAAAGGCT
ATGGTGGGGGCATCATGGACCCCAAGGTCCTGCCGCAGGGCCTGGAGCAGCACAGCAAAG
TCGGCCAGCGCCTGCTCCACAGTCAGCAGCTGTGTATATCCCCGCTGTGTGGACTGGACA
CCGAACGGAAGCGATTTCCCATAGTACCGCTGCAGAAAGCAGGAAGGGATGGCTAATCCA
CTCCTCGGTGCTCCCCACCTCCTTCAACTCAGGGACTGCCAGGAACTGTACAGGTACCCA
CGTGCTCAGCAAAGACAAGCAGGGCCTCCTGCTGGGCTGCCAGTTCCACCATGAAGCCAG
AGTTGTTAGCGAAGGACCAGATATCCCCCTCATTCCCTGTGTAGAAAAAG

>130 Get primers
CCCACAACTCAGGCGAGCAGCCTCACTGTCCTCCAGGCTGAGGTGCTAGGCTGCTCTTTC
CCTGCTCAGAACGCCCAAGGGTGGGAAAGAAGGACCTGAAACTGTCAGGCCCACACACCC
TGATCCCAGGGCCAAGGCAGATACAGCCTTCACTGGGAGAAGGCACCTGTGGGTGCCCTG
CCCTGACCCAGCAATGAAGACATTGCAGAGACAAAGTCAGAAGGAATTGTCCCACTAGTG
GGAACAACATAGCATACACTGCCTATGAGGTCCACTCAAGGAGGGCTTCCAGAAGGAGGT
AAAGCTAGACCCCGCCCTTCCACATGTGGGGTAGGCATAGGATGTTGAGACTGTAAGAGA
CATCTCTTTGGCCCTCCTTGTATAGGGTGTCAATCGGCACAACAGGGTGGAGCCTTAGAG
TAGGGTAAGATTAGGACTCTAGGTTCTCTCATGGGTCCAGATCTGTCATGAAGGGAGGTC
AAGGACCCACCTCCCTCCAAAGGCTATGGTGGGGGCATCATGGACCCCAAGGTCCTGCCG
CAGGGCCTGGAGCAGCACAGCAAAGTCGGCCAGCGCCTGCTCCACAGTCAGCAGCTGTGT
ATATCCCCGCTGTGTGGACTGGACACCGAACGGAAGCGATTTCCCATAGTACCGCTGCAG
AAAGCAGGAAGGGATGGCTAATCCACTCCTCGGTGCTCCCCACCTCCTTCAACTCAGGGA
CTGCCAGGAACTGTACAGGTACCCACGTGCTCAGCAAAGACAAGCAGGGCCTCCTGCTGG
GCTGCCAGTTCCACCATGAAGCCAGAGTTGTTAGCGAAGGACCAGATATCCCCCTCATTC
CCTGTGTAGAAAAAGATGGGCCCTTCGCCCATCTTCCAGAACTTATCTGTTGGAAGTAAA
TGAGTTTCCATAAGGCCAGGGAAACGCAGGTAGGAACCCATGCGGTCGAG

>131 Get primers
CTGAAACTGTCAGGCCCACACACCCTGATCCCAGGGCCAAGGCAGATACAGCCTTCACTG
GGAGAAGGCACCTGTGGGTGCCCTGCCCTGACCCAGCAATGAAGACATTGCAGAGACAAA
GTCAGAAGGAATTGTCCCACTAGTGGGAACAACATAGCATACACTGCCTATGAGGTCCAC
TCAAGGAGGGCTTCCAGAAGGAGGTAAAGCTAGACCCCGCCCTTCCACATGTGGGGTAGG
CATAGGATGTTGAGACTGTAAGAGACATCTCTTTGGCCCTCCTTGTATAGGGTGTCAATC
GGCACAACAGGGTGGAGCCTTAGAGTAGGGTAAGATTAGGACTCTAGGTTCTCTCATGGG
TCCAGATCTGTCATGAAGGGAGGTCAAGGACCCACCTCCCTCCAAAGGCTATGGTGGGGG
CATCATGGACCCCAAGGTCCTGCCGCAGGGCCTGGAGCAGCACAGCAAAGTCGGCCAGCG
CCTGCTCCACAGTCAGCAGCTGTGTATATCCCCGCTGTGTGGACTGGACACCGAACGGAA
GCGATTTCCCATAGTACCGCTGCAGAAAGCAGGAAGGGATGGCTAATCCACTCCTCGGTG
CTCCCCACCTCCTTCAACTCAGGGACTGCCAGGAACTGTACAGGTACCCACGTGCTCAGC
AAAGACAAGCAGGGCCTCCTGCTGGGCTGCCAGTTCCACCATGAAGCCAGAGTTGTTAGC
GAAGGACCAGATATCCCCCTCATTCCCTGTGTAGAAAAAGATGGGCCCTTCGCCCATCTT
CCAGAACTTATCTGTTGGAAGTAAATGAGTTTCCATAAGGCCAGGGAAACGCAGGTAGGA
ACCCATGCGGTCGAGCCAGCACTCACCTGACACTAGGAACCGCTGGCCAAAGGTTTTGTT
GCCGAAACTCTCAAAGTTGAAATGGTCCATGTATTGCTCAAAATAATTCT

>132 Get primers
GCAATGAAGACATTGCAGAGACAAAGTCAGAAGGAATTGTCCCACTAGTGGGAACAACAT
AGCATACACTGCCTATGAGGTCCACTCAAGGAGGGCTTCCAGAAGGAGGTAAAGCTAGAC
CCCGCCCTTCCACATGTGGGGTAGGCATAGGATGTTGAGACTGTAAGAGACATCTCTTTG
GCCCTCCTTGTATAGGGTGTCAATCGGCACAACAGGGTGGAGCCTTAGAGTAGGGTAAGA
TTAGGACTCTAGGTTCTCTCATGGGTCCAGATCTGTCATGAAGGGAGGTCAAGGACCCAC
CTCCCTCCAAAGGCTATGGTGGGGGCATCATGGACCCCAAGGTCCTGCCGCAGGGCCTGG
AGCAGCACAGCAAAGTCGGCCAGCGCCTGCTCCACAGTCAGCAGCTGTGTATATCCCCGC
TGTGTGGACTGGACACCGAACGGAAGCGATTTCCCATAGTACCGCTGCAGAAAGCAGGAA
GGGATGGCTAATCCACTCCTCGGTGCTCCCCACCTCCTTCAACTCAGGGACTGCCAGGAA
CTGTACAGGTACCCACGTGCTCAGCAAAGACAAGCAGGGCCTCCTGCTGGGCTGCCAGTT
CCACCATGAAGCCAGAGTTGTTAGCGAAGGACCAGATATCCCCCTCATTCCCTGTGTAGA
AAAAGATGGGCCCTTCGCCCATCTTCCAGAACTTATCTGTTGGAAGTAAATGAGTTTCCA
TAAGGCCAGGGAAACGCAGGTAGGAACCCATGCGGTCGAGCCAGCACTCACCTGACACTA
GGAACCGCTGGCCAAAGGTTTTGTTGCCGAAACTCTCAAAGTTGAAATGGTCCATGTATT
GCTCAAAATAATTCTCATGAAAGTCAGGGTCTAGAACTCTGTCGGCTGAGGGCAGGTGCA
GAGACTCAGGAGCTGGTTGGGATCATCAGGGATCTAGGCGGGTCAGGAGG

>133 Get primers
CTTCCAGAAGGAGGTAAAGCTAGACCCCGCCCTTCCACATGTGGGGTAGGCATAGGATGT
TGAGACTGTAAGAGACATCTCTTTGGCCCTCCTTGTATAGGGTGTCAATCGGCACAACAG
GGTGGAGCCTTAGAGTAGGGTAAGATTAGGACTCTAGGTTCTCTCATGGGTCCAGATCTG
TCATGAAGGGAGGTCAAGGACCCACCTCCCTCCAAAGGCTATGGTGGGGGCATCATGGAC
CCCAAGGTCCTGCCGCAGGGCCTGGAGCAGCACAGCAAAGTCGGCCAGCGCCTGCTCCAC
AGTCAGCAGCTGTGTATATCCCCGCTGTGTGGACTGGACACCGAACGGAAGCGATTTCCC
ATAGTACCGCTGCAGAAAGCAGGAAGGGATGGCTAATCCACTCCTCGGTGCTCCCCACCT
CCTTCAACTCAGGGACTGCCAGGAACTGTACAGGTACCCACGTGCTCAGCAAAGACAAGC
AGGGCCTCCTGCTGGGCTGCCAGTTCCACCATGAAGCCAGAGTTGTTAGCGAAGGACCAG
ATATCCCCCTCATTCCCTGTGTAGAAAAAGATGGGCCCTTCGCCCATCTTCCAGAACTTA
TCTGTTGGAAGTAAATGAGTTTCCATAAGGCCAGGGAAACGCAGGTAGGAACCCATGCGG
TCGAGCCAGCACTCACCTGACACTAGGAACCGCTGGCCAAAGGTTTTGTTGCCGAAACTC
TCAAAGTTGAAATGGTCCATGTATTGCTCAAAATAATTCTCATGAAAGTCAGGGTCTAGA
ACTCTGTCGGCTGAGGGCAGGTGCAGAGACTCAGGAGCTGGTTGGGATCATCAGGGATCT
AGGCGGGTCAGGAGGAAGGGCAGCCAGTCTGTACTCACCTCTGGCCTGGAGGTTGCACAG
TCCCAGTGACAGCAGCAGGACCAGGATCCAGGAGGGGACACCATGGTCCA

>134 Get primers
TATAGGGTGTCAATCGGCACAACAGGGTGGAGCCTTAGAGTAGGGTAAGATTAGGACTCT
AGGTTCTCTCATGGGTCCAGATCTGTCATGAAGGGAGGTCAAGGACCCACCTCCCTCCAA
AGGCTATGGTGGGGGCATCATGGACCCCAAGGTCCTGCCGCAGGGCCTGGAGCAGCACAG
CAAAGTCGGCCAGCGCCTGCTCCACAGTCAGCAGCTGTGTATATCCCCGCTGTGTGGACT
GGACACCGAACGGAAGCGATTTCCCATAGTACCGCTGCAGAAAGCAGGAAGGGATGGCTA
ATCCACTCCTCGGTGCTCCCCACCTCCTTCAACTCAGGGACTGCCAGGAACTGTACAGGT
ACCCACGTGCTCAGCAAAGACAAGCAGGGCCTCCTGCTGGGCTGCCAGTTCCACCATGAA
GCCAGAGTTGTTAGCGAAGGACCAGATATCCCCCTCATTCCCTGTGTAGAAAAAGATGGG
CCCTTCGCCCATCTTCCAGAACTTATCTGTTGGAAGTAAATGAGTTTCCATAAGGCCAGG
GAAACGCAGGTAGGAACCCATGCGGTCGAGCCAGCACTCACCTGACACTAGGAACCGCTG
GCCAAAGGTTTTGTTGCCGAAACTCTCAAAGTTGAAATGGTCCATGTATTGCTCAAAATA
ATTCTCATGAAAGTCAGGGTCTAGAACTCTGTCGGCTGAGGGCAGGTGCAGAGACTCAGG
AGCTGGTTGGGATCATCAGGGATCTAGGCGGGTCAGGAGGAAGGGCAGCCAGTCTGTACT
CACCTCTGGCCTGGAGGTTGCACAGTCCCAGTGACAGCAGCAGGACCAGGATCCAGGAGG
GGACACCATGGTCCACAGGGTAACAAGGATGGAAGTTCATGCTTGATTCTGAGCCGGGCG
CTGACTGTCATGTGATTTGGTCACATGACCGACACAACGGGCGGGGCAGC

>135 Get primers
AGGTCAAGGACCCACCTCCCTCCAAAGGCTATGGTGGGGGCATCATGGACCCCAAGGTCC
TGCCGCAGGGCCTGGAGCAGCACAGCAAAGTCGGCCAGCGCCTGCTCCACAGTCAGCAGC
TGTGTATATCCCCGCTGTGTGGACTGGACACCGAACGGAAGCGATTTCCCATAGTACCGC
TGCAGAAAGCAGGAAGGGATGGCTAATCCACTCCTCGGTGCTCCCCACCTCCTTCAACTC
AGGGACTGCCAGGAACTGTACAGGTACCCACGTGCTCAGCAAAGACAAGCAGGGCCTCCT
GCTGGGCTGCCAGTTCCACCATGAAGCCAGAGTTGTTAGCGAAGGACCAGATATCCCCCT
CATTCCCTGTGTAGAAAAAGATGGGCCCTTCGCCCATCTTCCAGAACTTATCTGTTGGAA
GTAAATGAGTTTCCATAAGGCCAGGGAAACGCAGGTAGGAACCCATGCGGTCGAGCCAGC
ACTCACCTGACACTAGGAACCGCTGGCCAAAGGTTTTGTTGCCGAAACTCTCAAAGTTGA
AATGGTCCATGTATTGCTCAAAATAATTCTCATGAAAGTCAGGGTCTAGAACTCTGTCGG
CTGAGGGCAGGTGCAGAGACTCAGGAGCTGGTTGGGATCATCAGGGATCTAGGCGGGTCA
GGAGGAAGGGCAGCCAGTCTGTACTCACCTCTGGCCTGGAGGTTGCACAGTCCCAGTGAC
AGCAGCAGGACCAGGATCCAGGAGGGGACACCATGGTCCACAGGGTAACAAGGATGGAAG
TTCATGCTTGATTCTGAGCCGGGCGCTGACTGTCATGTGATTTGGTCACATGACCGACAC
AACGGGCGGGGCAGCATCACGTGATAGTCTGGCGGGGGCTGTCCTACTGTGGCTGGATTC
TAGTTGGAGGATCAGCCTACTCTTCTTCAGTTTCCCGGTTCCTCCAAATT

>150 Get primers
GCTGCTGCCATTCTTGCACTGGCCTGGGGTACCCAAGTCCCCTCACTCACCGCTGTGACA
TCTCGGAAGAATTGGTAGGAGTCCCCAAGGCCTGCAACAGCTACAACAGGAGCGCTGGCT
GCCAGTGCCCCAGCCACCAGGTGGGGGTACTTCATCCTCATGTAGGCACTCAGCATCCCC
CCATAACTGGGAGTACAGAGCACAGATCATGGTTGTGGGAAGCTGCCCACAACTCAGGCG
AGCAGCCTCACTGTCCTCCAGGCTGAGGTGCTAGGCTGCTCTTTCCCTGCTCAGAACGCC
CAAGGGTGGGAAAGAAGGACCTGAAACTGTCAGGCCCACACACCCTGATCCCAGGGCCAA
GGCAGATACAGCCTTCACTGGGAGAAGGCACCTGTGGGTGCCCTGCCCTGACCCAGCAAT
GAAGACATTGCAGAGACAAAGTCAGAAGGAATTGTCCCACTAGTGGGAACAACATAGCAT
ACACTGCCTATGAGGTCCACTCAAGGAGGGCTTCCAGAAGGAGGTAAAGCTAGACCCCGC
CCTTCCACATGTGGGGTAGGCATAGGATGTTGAGACTGTAAGAGACATCTCTTTGGCCCT
CCTTGTATAGGGTGTCAATCGGCACAACAGGGTGGAGCCTTAGAGTAGGGTAAGATTAGG
ACTCTAGGTTCTCTCATGGGTCCAGATCTGTCATGAAGGGAGGTCAAGGACCCACCTCCC
TCCAAAGGCTATGGTGGGGGCATCATGGACCCCAAGGTCCTGCCGCAGGGCCTGGAGCAG
CACAGCAAAGTCGGCCAGCGCCTGCTCCACAGTCAGCAGCTGTGTATATCCCCGCTGTGT
GGACTGGACACCGAACGGAAGCGATTTCCCATAGTACCGCTGCAGAAAGCAGGAAGGGAT

>151 Get primers
CCTGCAACAGCTACAACAGGAGCGCTGGCTGCCAGTGCCCCAGCCACCAGGTGGGGGTAC
TTCATCCTCATGTAGGCACTCAGCATCCCCCCATAACTGGGAGTACAGAGCACAGATCAT
GGTTGTGGGAAGCTGCCCACAACTCAGGCGAGCAGCCTCACTGTCCTCCAGGCTGAGGTG
CTAGGCTGCTCTTTCCCTGCTCAGAACGCCCAAGGGTGGGAAAGAAGGACCTGAAACTGT
CAGGCCCACACACCCTGATCCCAGGGCCAAGGCAGATACAGCCTTCACTGGGAGAAGGCA
CCTGTGGGTGCCCTGCCCTGACCCAGCAATGAAGACATTGCAGAGACAAAGTCAGAAGGA
ATTGTCCCACTAGTGGGAACAACATAGCATACACTGCCTATGAGGTCCACTCAAGGAGGG
CTTCCAGAAGGAGGTAAAGCTAGACCCCGCCCTTCCACATGTGGGGTAGGCATAGGATGT
TGAGACTGTAAGAGACATCTCTTTGGCCCTCCTTGTATAGGGTGTCAATCGGCACAACAG
GGTGGAGCCTTAGAGTAGGGTAAGATTAGGACTCTAGGTTCTCTCATGGGTCCAGATCTG
TCATGAAGGGAGGTCAAGGACCCACCTCCCTCCAAAGGCTATGGTGGGGGCATCATGGAC
CCCAAGGTCCTGCCGCAGGGCCTGGAGCAGCACAGCAAAGTCGGCCAGCGCCTGCTCCAC
AGTCAGCAGCTGTGTATATCCCCGCTGTGTGGACTGGACACCGAACGGAAGCGATTTCCC
ATAGTACCGCTGCAGAAAGCAGGAAGGGATGGCTAATCCACTCCTCGGTGCTCCCCACCT
CCTTCAACTCAGGGACTGCCAGGAACTGTACAGGTACCCACGTGCTCAGCAAAGACAAGC

>152 Get primers
CCATAACTGGGAGTACAGAGCACAGATCATGGTTGTGGGAAGCTGCCCACAACTCAGGCG
AGCAGCCTCACTGTCCTCCAGGCTGAGGTGCTAGGCTGCTCTTTCCCTGCTCAGAACGCC
CAAGGGTGGGAAAGAAGGACCTGAAACTGTCAGGCCCACACACCCTGATCCCAGGGCCAA
GGCAGATACAGCCTTCACTGGGAGAAGGCACCTGTGGGTGCCCTGCCCTGACCCAGCAAT
GAAGACATTGCAGAGACAAAGTCAGAAGGAATTGTCCCACTAGTGGGAACAACATAGCAT
ACACTGCCTATGAGGTCCACTCAAGGAGGGCTTCCAGAAGGAGGTAAAGCTAGACCCCGC
CCTTCCACATGTGGGGTAGGCATAGGATGTTGAGACTGTAAGAGACATCTCTTTGGCCCT
CCTTGTATAGGGTGTCAATCGGCACAACAGGGTGGAGCCTTAGAGTAGGGTAAGATTAGG
ACTCTAGGTTCTCTCATGGGTCCAGATCTGTCATGAAGGGAGGTCAAGGACCCACCTCCC
TCCAAAGGCTATGGTGGGGGCATCATGGACCCCAAGGTCCTGCCGCAGGGCCTGGAGCAG
CACAGCAAAGTCGGCCAGCGCCTGCTCCACAGTCAGCAGCTGTGTATATCCCCGCTGTGT
GGACTGGACACCGAACGGAAGCGATTTCCCATAGTACCGCTGCAGAAAGCAGGAAGGGAT
GGCTAATCCACTCCTCGGTGCTCCCCACCTCCTTCAACTCAGGGACTGCCAGGAACTGTA
CAGGTACCCACGTGCTCAGCAAAGACAAGCAGGGCCTCCTGCTGGGCTGCCAGTTCCACC
ATGAAGCCAGAGTTGTTAGCGAAGGACCAGATATCCCCCTCATTCCCTGTGTAGAAAAAG

>153 Get primers
CTAGGCTGCTCTTTCCCTGCTCAGAACGCCCAAGGGTGGGAAAGAAGGACCTGAAACTGT
CAGGCCCACACACCCTGATCCCAGGGCCAAGGCAGATACAGCCTTCACTGGGAGAAGGCA
CCTGTGGGTGCCCTGCCCTGACCCAGCAATGAAGACATTGCAGAGACAAAGTCAGAAGGA
ATTGTCCCACTAGTGGGAACAACATAGCATACACTGCCTATGAGGTCCACTCAAGGAGGG
CTTCCAGAAGGAGGTAAAGCTAGACCCCGCCCTTCCACATGTGGGGTAGGCATAGGATGT
TGAGACTGTAAGAGACATCTCTTTGGCCCTCCTTGTATAGGGTGTCAATCGGCACAACAG
GGTGGAGCCTTAGAGTAGGGTAAGATTAGGACTCTAGGTTCTCTCATGGGTCCAGATCTG
TCATGAAGGGAGGTCAAGGACCCACCTCCCTCCAAAGGCTATGGTGGGGGCATCATGGAC
CCCAAGGTCCTGCCGCAGGGCCTGGAGCAGCACAGCAAAGTCGGCCAGCGCCTGCTCCAC
AGTCAGCAGCTGTGTATATCCCCGCTGTGTGGACTGGACACCGAACGGAAGCGATTTCCC
ATAGTACCGCTGCAGAAAGCAGGAAGGGATGGCTAATCCACTCCTCGGTGCTCCCCACCT
CCTTCAACTCAGGGACTGCCAGGAACTGTACAGGTACCCACGTGCTCAGCAAAGACAAGC
AGGGCCTCCTGCTGGGCTGCCAGTTCCACCATGAAGCCAGAGTTGTTAGCGAAGGACCAG
ATATCCCCCTCATTCCCTGTGTAGAAAAAGATGGGCCCTTCGCCCATCTTCCAGAACTTA
TCTGTTGGAAGTAAATGAGTTTCCATAAGGCCAGGGAAACGCAGGTAGGAACCCATGCGG

>154 Get primers
GGCAGATACAGCCTTCACTGGGAGAAGGCACCTGTGGGTGCCCTGCCCTGACCCAGCAAT
GAAGACATTGCAGAGACAAAGTCAGAAGGAATTGTCCCACTAGTGGGAACAACATAGCAT
ACACTGCCTATGAGGTCCACTCAAGGAGGGCTTCCAGAAGGAGGTAAAGCTAGACCCCGC
CCTTCCACATGTGGGGTAGGCATAGGATGTTGAGACTGTAAGAGACATCTCTTTGGCCCT
CCTTGTATAGGGTGTCAATCGGCACAACAGGGTGGAGCCTTAGAGTAGGGTAAGATTAGG
ACTCTAGGTTCTCTCATGGGTCCAGATCTGTCATGAAGGGAGGTCAAGGACCCACCTCCC
TCCAAAGGCTATGGTGGGGGCATCATGGACCCCAAGGTCCTGCCGCAGGGCCTGGAGCAG
CACAGCAAAGTCGGCCAGCGCCTGCTCCACAGTCAGCAGCTGTGTATATCCCCGCTGTGT
GGACTGGACACCGAACGGAAGCGATTTCCCATAGTACCGCTGCAGAAAGCAGGAAGGGAT
GGCTAATCCACTCCTCGGTGCTCCCCACCTCCTTCAACTCAGGGACTGCCAGGAACTGTA
CAGGTACCCACGTGCTCAGCAAAGACAAGCAGGGCCTCCTGCTGGGCTGCCAGTTCCACC
ATGAAGCCAGAGTTGTTAGCGAAGGACCAGATATCCCCCTCATTCCCTGTGTAGAAAAAG
ATGGGCCCTTCGCCCATCTTCCAGAACTTATCTGTTGGAAGTAAATGAGTTTCCATAAGG
CCAGGGAAACGCAGGTAGGAACCCATGCGGTCGAGCCAGCACTCACCTGACACTAGGAAC
CGCTGGCCAAAGGTTTTGTTGCCGAAACTCTCAAAGTTGAAATGGTCCATGTATTGCTCA

>155 Get primers
ATTGTCCCACTAGTGGGAACAACATAGCATACACTGCCTATGAGGTCCACTCAAGGAGGG
CTTCCAGAAGGAGGTAAAGCTAGACCCCGCCCTTCCACATGTGGGGTAGGCATAGGATGT
TGAGACTGTAAGAGACATCTCTTTGGCCCTCCTTGTATAGGGTGTCAATCGGCACAACAG
GGTGGAGCCTTAGAGTAGGGTAAGATTAGGACTCTAGGTTCTCTCATGGGTCCAGATCTG
TCATGAAGGGAGGTCAAGGACCCACCTCCCTCCAAAGGCTATGGTGGGGGCATCATGGAC
CCCAAGGTCCTGCCGCAGGGCCTGGAGCAGCACAGCAAAGTCGGCCAGCGCCTGCTCCAC
AGTCAGCAGCTGTGTATATCCCCGCTGTGTGGACTGGACACCGAACGGAAGCGATTTCCC
ATAGTACCGCTGCAGAAAGCAGGAAGGGATGGCTAATCCACTCCTCGGTGCTCCCCACCT
CCTTCAACTCAGGGACTGCCAGGAACTGTACAGGTACCCACGTGCTCAGCAAAGACAAGC
AGGGCCTCCTGCTGGGCTGCCAGTTCCACCATGAAGCCAGAGTTGTTAGCGAAGGACCAG
ATATCCCCCTCATTCCCTGTGTAGAAAAAGATGGGCCCTTCGCCCATCTTCCAGAACTTA
TCTGTTGGAAGTAAATGAGTTTCCATAAGGCCAGGGAAACGCAGGTAGGAACCCATGCGG
TCGAGCCAGCACTCACCTGACACTAGGAACCGCTGGCCAAAGGTTTTGTTGCCGAAACTC
TCAAAGTTGAAATGGTCCATGTATTGCTCAAAATAATTCTCATGAAAGTCAGGGTCTAGA
ACTCTGTCGGCTGAGGGCAGGTGCAGAGACTCAGGAGCTGGTTGGGATCATCAGGGATCT

>156 Get primers
CCTTCCACATGTGGGGTAGGCATAGGATGTTGAGACTGTAAGAGACATCTCTTTGGCCCT
CCTTGTATAGGGTGTCAATCGGCACAACAGGGTGGAGCCTTAGAGTAGGGTAAGATTAGG
ACTCTAGGTTCTCTCATGGGTCCAGATCTGTCATGAAGGGAGGTCAAGGACCCACCTCCC
TCCAAAGGCTATGGTGGGGGCATCATGGACCCCAAGGTCCTGCCGCAGGGCCTGGAGCAG
CACAGCAAAGTCGGCCAGCGCCTGCTCCACAGTCAGCAGCTGTGTATATCCCCGCTGTGT
GGACTGGACACCGAACGGAAGCGATTTCCCATAGTACCGCTGCAGAAAGCAGGAAGGGAT
GGCTAATCCACTCCTCGGTGCTCCCCACCTCCTTCAACTCAGGGACTGCCAGGAACTGTA
CAGGTACCCACGTGCTCAGCAAAGACAAGCAGGGCCTCCTGCTGGGCTGCCAGTTCCACC
ATGAAGCCAGAGTTGTTAGCGAAGGACCAGATATCCCCCTCATTCCCTGTGTAGAAAAAG
ATGGGCCCTTCGCCCATCTTCCAGAACTTATCTGTTGGAAGTAAATGAGTTTCCATAAGG
CCAGGGAAACGCAGGTAGGAACCCATGCGGTCGAGCCAGCACTCACCTGACACTAGGAAC
CGCTGGCCAAAGGTTTTGTTGCCGAAACTCTCAAAGTTGAAATGGTCCATGTATTGCTCA
AAATAATTCTCATGAAAGTCAGGGTCTAGAACTCTGTCGGCTGAGGGCAGGTGCAGAGAC
TCAGGAGCTGGTTGGGATCATCAGGGATCTAGGCGGGTCAGGAGGAAGGGCAGCCAGTCT
GTACTCACCTCTGGCCTGGAGGTTGCACAGTCCCAGTGACAGCAGCAGGACCAGGATCCA

>157 Get primers
GGTGGAGCCTTAGAGTAGGGTAAGATTAGGACTCTAGGTTCTCTCATGGGTCCAGATCTG
TCATGAAGGGAGGTCAAGGACCCACCTCCCTCCAAAGGCTATGGTGGGGGCATCATGGAC
CCCAAGGTCCTGCCGCAGGGCCTGGAGCAGCACAGCAAAGTCGGCCAGCGCCTGCTCCAC
AGTCAGCAGCTGTGTATATCCCCGCTGTGTGGACTGGACACCGAACGGAAGCGATTTCCC
ATAGTACCGCTGCAGAAAGCAGGAAGGGATGGCTAATCCACTCCTCGGTGCTCCCCACCT
CCTTCAACTCAGGGACTGCCAGGAACTGTACAGGTACCCACGTGCTCAGCAAAGACAAGC
AGGGCCTCCTGCTGGGCTGCCAGTTCCACCATGAAGCCAGAGTTGTTAGCGAAGGACCAG
ATATCCCCCTCATTCCCTGTGTAGAAAAAGATGGGCCCTTCGCCCATCTTCCAGAACTTA
TCTGTTGGAAGTAAATGAGTTTCCATAAGGCCAGGGAAACGCAGGTAGGAACCCATGCGG
TCGAGCCAGCACTCACCTGACACTAGGAACCGCTGGCCAAAGGTTTTGTTGCCGAAACTC
TCAAAGTTGAAATGGTCCATGTATTGCTCAAAATAATTCTCATGAAAGTCAGGGTCTAGA
ACTCTGTCGGCTGAGGGCAGGTGCAGAGACTCAGGAGCTGGTTGGGATCATCAGGGATCT
AGGCGGGTCAGGAGGAAGGGCAGCCAGTCTGTACTCACCTCTGGCCTGGAGGTTGCACAG
TCCCAGTGACAGCAGCAGGACCAGGATCCAGGAGGGGACACCATGGTCCACAGGGTAACA
AGGATGGAAGTTCATGCTTGATTCTGAGCCGGGCGCTGACTGTCATGTGATTTGGTCACA

>158 Get primers
TCCAAAGGCTATGGTGGGGGCATCATGGACCCCAAGGTCCTGCCGCAGGGCCTGGAGCAG
CACAGCAAAGTCGGCCAGCGCCTGCTCCACAGTCAGCAGCTGTGTATATCCCCGCTGTGT
GGACTGGACACCGAACGGAAGCGATTTCCCATAGTACCGCTGCAGAAAGCAGGAAGGGAT
GGCTAATCCACTCCTCGGTGCTCCCCACCTCCTTCAACTCAGGGACTGCCAGGAACTGTA
CAGGTACCCACGTGCTCAGCAAAGACAAGCAGGGCCTCCTGCTGGGCTGCCAGTTCCACC
ATGAAGCCAGAGTTGTTAGCGAAGGACCAGATATCCCCCTCATTCCCTGTGTAGAAAAAG
ATGGGCCCTTCGCCCATCTTCCAGAACTTATCTGTTGGAAGTAAATGAGTTTCCATAAGG
CCAGGGAAACGCAGGTAGGAACCCATGCGGTCGAGCCAGCACTCACCTGACACTAGGAAC
CGCTGGCCAAAGGTTTTGTTGCCGAAACTCTCAAAGTTGAAATGGTCCATGTATTGCTCA
AAATAATTCTCATGAAAGTCAGGGTCTAGAACTCTGTCGGCTGAGGGCAGGTGCAGAGAC
TCAGGAGCTGGTTGGGATCATCAGGGATCTAGGCGGGTCAGGAGGAAGGGCAGCCAGTCT
GTACTCACCTCTGGCCTGGAGGTTGCACAGTCCCAGTGACAGCAGCAGGACCAGGATCCA
GGAGGGGACACCATGGTCCACAGGGTAACAAGGATGGAAGTTCATGCTTGATTCTGAGCC
GGGCGCTGACTGTCATGTGATTTGGTCACATGACCGACACAACGGGCGGGGCAGCATCAC
GTGATAGTCTGGCGGGGGCTGTCCTACTGTGGCTGGATTCTAGTTGGAGGATCAGCCTAC

>174 Get primers
CTGGCCATAAAAGTCCTGGAGAAGAGACCAAGGTTGCTGCTGCCATTCTTGCACTGGCCT
GGGGTACCCAAGTCCCCTCACTCACCGCTGTGACATCTCGGAAGAATTGGTAGGAGTCCC
CAAGGCCTGCAACAGCTACAACAGGAGCGCTGGCTGCCAGTGCCCCAGCCACCAGGTGGG
GGTACTTCATCCTCATGTAGGCACTCAGCATCCCCCCATAACTGGGAGTACAGAGCACAG
ATCATGGTTGTGGGAAGCTGCCCACAACTCAGGCGAGCAGCCTCACTGTCCTCCAGGCTG
AGGTGCTAGGCTGCTCTTTCCCTGCTCAGAACGCCCAAGGGTGGGAAAGAAGGACCTGAA
ACTGTCAGGCCCACACACCCTGATCCCAGGGCCAAGGCAGATACAGCCTTCACTGGGAGA
AGGCACCTGTGGGTGCCCTGCCCTGACCCAGCAATGAAGACATTGCAGAGACAAAGTCAG
AAGGAATTGTCCCACTAGTGGGAACAACATAGCATACACTGCCTATGAGGTCCACTCAAG
GAGGGCTTCCAGAAGGAGGTAAAGCTAGACCCCGCCCTTCCACATGTGGGGTAGGCATAG
GATGTTGAGACTGTAAGAGACATCTCTTTGGCCCTCCTTGTATAGGGTGTCAATCGGCAC
AACAGGGTGGAGCCTTAGAGTAGGGTAAGATTAGGACTCTAGGTTCTCTCATGGGTCCAG
ATCTGTCATGAAGGGAGGTCAAGGACCCACCTCCCTCCAAAGGCTATGGTGGGGGCATCA
TGGACCCCAAGGTCCTGCCGCAGGGCCTGGAGCAGCACAGCAAAGTCGGCCAGCGCCTGC
TCCACAGTCA

>175 Get primers
CGCTGTGACATCTCGGAAGAATTGGTAGGAGTCCCCAAGGCCTGCAACAGCTACAACAGG
AGCGCTGGCTGCCAGTGCCCCAGCCACCAGGTGGGGGTACTTCATCCTCATGTAGGCACT
CAGCATCCCCCCATAACTGGGAGTACAGAGCACAGATCATGGTTGTGGGAAGCTGCCCAC
AACTCAGGCGAGCAGCCTCACTGTCCTCCAGGCTGAGGTGCTAGGCTGCTCTTTCCCTGC
TCAGAACGCCCAAGGGTGGGAAAGAAGGACCTGAAACTGTCAGGCCCACACACCCTGATC
CCAGGGCCAAGGCAGATACAGCCTTCACTGGGAGAAGGCACCTGTGGGTGCCCTGCCCTG
ACCCAGCAATGAAGACATTGCAGAGACAAAGTCAGAAGGAATTGTCCCACTAGTGGGAAC
AACATAGCATACACTGCCTATGAGGTCCACTCAAGGAGGGCTTCCAGAAGGAGGTAAAGC
TAGACCCCGCCCTTCCACATGTGGGGTAGGCATAGGATGTTGAGACTGTAAGAGACATCT
CTTTGGCCCTCCTTGTATAGGGTGTCAATCGGCACAACAGGGTGGAGCCTTAGAGTAGGG
TAAGATTAGGACTCTAGGTTCTCTCATGGGTCCAGATCTGTCATGAAGGGAGGTCAAGGA
CCCACCTCCCTCCAAAGGCTATGGTGGGGGCATCATGGACCCCAAGGTCCTGCCGCAGGG
CCTGGAGCAGCACAGCAAAGTCGGCCAGCGCCTGCTCCACAGTCAGCAGCTGTGTATATC
CCCGCTGTGTGGACTGGACACCGAACGGAAGCGATTTCCCATAGTACCGCTGCAGAAAGC
AGGAAGGGAT

>176 Get primers
ACCAGGTGGGGGTACTTCATCCTCATGTAGGCACTCAGCATCCCCCCATAACTGGGAGTA
CAGAGCACAGATCATGGTTGTGGGAAGCTGCCCACAACTCAGGCGAGCAGCCTCACTGTC
CTCCAGGCTGAGGTGCTAGGCTGCTCTTTCCCTGCTCAGAACGCCCAAGGGTGGGAAAGA
AGGACCTGAAACTGTCAGGCCCACACACCCTGATCCCAGGGCCAAGGCAGATACAGCCTT
CACTGGGAGAAGGCACCTGTGGGTGCCCTGCCCTGACCCAGCAATGAAGACATTGCAGAG
ACAAAGTCAGAAGGAATTGTCCCACTAGTGGGAACAACATAGCATACACTGCCTATGAGG
TCCACTCAAGGAGGGCTTCCAGAAGGAGGTAAAGCTAGACCCCGCCCTTCCACATGTGGG
GTAGGCATAGGATGTTGAGACTGTAAGAGACATCTCTTTGGCCCTCCTTGTATAGGGTGT
CAATCGGCACAACAGGGTGGAGCCTTAGAGTAGGGTAAGATTAGGACTCTAGGTTCTCTC
ATGGGTCCAGATCTGTCATGAAGGGAGGTCAAGGACCCACCTCCCTCCAAAGGCTATGGT
GGGGGCATCATGGACCCCAAGGTCCTGCCGCAGGGCCTGGAGCAGCACAGCAAAGTCGGC
CAGCGCCTGCTCCACAGTCAGCAGCTGTGTATATCCCCGCTGTGTGGACTGGACACCGAA
CGGAAGCGATTTCCCATAGTACCGCTGCAGAAAGCAGGAAGGGATGGCTAATCCACTCCT
CGGTGCTCCCCACCTCCTTCAACTCAGGGACTGCCAGGAACTGTACAGGTACCCACGTGC
TCAGCAAAGA

>177 Get primers
AGCTGCCCACAACTCAGGCGAGCAGCCTCACTGTCCTCCAGGCTGAGGTGCTAGGCTGCT
CTTTCCCTGCTCAGAACGCCCAAGGGTGGGAAAGAAGGACCTGAAACTGTCAGGCCCACA
CACCCTGATCCCAGGGCCAAGGCAGATACAGCCTTCACTGGGAGAAGGCACCTGTGGGTG
CCCTGCCCTGACCCAGCAATGAAGACATTGCAGAGACAAAGTCAGAAGGAATTGTCCCAC
TAGTGGGAACAACATAGCATACACTGCCTATGAGGTCCACTCAAGGAGGGCTTCCAGAAG
GAGGTAAAGCTAGACCCCGCCCTTCCACATGTGGGGTAGGCATAGGATGTTGAGACTGTA
AGAGACATCTCTTTGGCCCTCCTTGTATAGGGTGTCAATCGGCACAACAGGGTGGAGCCT
TAGAGTAGGGTAAGATTAGGACTCTAGGTTCTCTCATGGGTCCAGATCTGTCATGAAGGG
AGGTCAAGGACCCACCTCCCTCCAAAGGCTATGGTGGGGGCATCATGGACCCCAAGGTCC
TGCCGCAGGGCCTGGAGCAGCACAGCAAAGTCGGCCAGCGCCTGCTCCACAGTCAGCAGC
TGTGTATATCCCCGCTGTGTGGACTGGACACCGAACGGAAGCGATTTCCCATAGTACCGC
TGCAGAAAGCAGGAAGGGATGGCTAATCCACTCCTCGGTGCTCCCCACCTCCTTCAACTC
AGGGACTGCCAGGAACTGTACAGGTACCCACGTGCTCAGCAAAGACAAGCAGGGCCTCCT
GCTGGGCTGCCAGTTCCACCATGAAGCCAGAGTTGTTAGCGAAGGACCAGATATCCCCCT
CATTCCCTGT

>178 Get primers
GTGGGAAAGAAGGACCTGAAACTGTCAGGCCCACACACCCTGATCCCAGGGCCAAGGCAG
ATACAGCCTTCACTGGGAGAAGGCACCTGTGGGTGCCCTGCCCTGACCCAGCAATGAAGA
CATTGCAGAGACAAAGTCAGAAGGAATTGTCCCACTAGTGGGAACAACATAGCATACACT
GCCTATGAGGTCCACTCAAGGAGGGCTTCCAGAAGGAGGTAAAGCTAGACCCCGCCCTTC
CACATGTGGGGTAGGCATAGGATGTTGAGACTGTAAGAGACATCTCTTTGGCCCTCCTTG
TATAGGGTGTCAATCGGCACAACAGGGTGGAGCCTTAGAGTAGGGTAAGATTAGGACTCT
AGGTTCTCTCATGGGTCCAGATCTGTCATGAAGGGAGGTCAAGGACCCACCTCCCTCCAA
AGGCTATGGTGGGGGCATCATGGACCCCAAGGTCCTGCCGCAGGGCCTGGAGCAGCACAG
CAAAGTCGGCCAGCGCCTGCTCCACAGTCAGCAGCTGTGTATATCCCCGCTGTGTGGACT
GGACACCGAACGGAAGCGATTTCCCATAGTACCGCTGCAGAAAGCAGGAAGGGATGGCTA
ATCCACTCCTCGGTGCTCCCCACCTCCTTCAACTCAGGGACTGCCAGGAACTGTACAGGT
ACCCACGTGCTCAGCAAAGACAAGCAGGGCCTCCTGCTGGGCTGCCAGTTCCACCATGAA
GCCAGAGTTGTTAGCGAAGGACCAGATATCCCCCTCATTCCCTGTGTAGAAAAAGATGGG
CCCTTCGCCCATCTTCCAGAACTTATCTGTTGGAAGTAAATGAGTTTCCATAAGGCCAGG
GAAACGCAGG

>179 Get primers
CCTGTGGGTGCCCTGCCCTGACCCAGCAATGAAGACATTGCAGAGACAAAGTCAGAAGGA
ATTGTCCCACTAGTGGGAACAACATAGCATACACTGCCTATGAGGTCCACTCAAGGAGGG
CTTCCAGAAGGAGGTAAAGCTAGACCCCGCCCTTCCACATGTGGGGTAGGCATAGGATGT
TGAGACTGTAAGAGACATCTCTTTGGCCCTCCTTGTATAGGGTGTCAATCGGCACAACAG
GGTGGAGCCTTAGAGTAGGGTAAGATTAGGACTCTAGGTTCTCTCATGGGTCCAGATCTG
TCATGAAGGGAGGTCAAGGACCCACCTCCCTCCAAAGGCTATGGTGGGGGCATCATGGAC
CCCAAGGTCCTGCCGCAGGGCCTGGAGCAGCACAGCAAAGTCGGCCAGCGCCTGCTCCAC
AGTCAGCAGCTGTGTATATCCCCGCTGTGTGGACTGGACACCGAACGGAAGCGATTTCCC
ATAGTACCGCTGCAGAAAGCAGGAAGGGATGGCTAATCCACTCCTCGGTGCTCCCCACCT
CCTTCAACTCAGGGACTGCCAGGAACTGTACAGGTACCCACGTGCTCAGCAAAGACAAGC
AGGGCCTCCTGCTGGGCTGCCAGTTCCACCATGAAGCCAGAGTTGTTAGCGAAGGACCAG
ATATCCCCCTCATTCCCTGTGTAGAAAAAGATGGGCCCTTCGCCCATCTTCCAGAACTTA
TCTGTTGGAAGTAAATGAGTTTCCATAAGGCCAGGGAAACGCAGGTAGGAACCCATGCGG
TCGAGCCAGCACTCACCTGACACTAGGAACCGCTGGCCAAAGGTTTTGTTGCCGAAACTC
TCAAAGTTGA

>180 Get primers
AGCATACACTGCCTATGAGGTCCACTCAAGGAGGGCTTCCAGAAGGAGGTAAAGCTAGAC
CCCGCCCTTCCACATGTGGGGTAGGCATAGGATGTTGAGACTGTAAGAGACATCTCTTTG
GCCCTCCTTGTATAGGGTGTCAATCGGCACAACAGGGTGGAGCCTTAGAGTAGGGTAAGA
TTAGGACTCTAGGTTCTCTCATGGGTCCAGATCTGTCATGAAGGGAGGTCAAGGACCCAC
CTCCCTCCAAAGGCTATGGTGGGGGCATCATGGACCCCAAGGTCCTGCCGCAGGGCCTGG
AGCAGCACAGCAAAGTCGGCCAGCGCCTGCTCCACAGTCAGCAGCTGTGTATATCCCCGC
TGTGTGGACTGGACACCGAACGGAAGCGATTTCCCATAGTACCGCTGCAGAAAGCAGGAA
GGGATGGCTAATCCACTCCTCGGTGCTCCCCACCTCCTTCAACTCAGGGACTGCCAGGAA
CTGTACAGGTACCCACGTGCTCAGCAAAGACAAGCAGGGCCTCCTGCTGGGCTGCCAGTT
CCACCATGAAGCCAGAGTTGTTAGCGAAGGACCAGATATCCCCCTCATTCCCTGTGTAGA
AAAAGATGGGCCCTTCGCCCATCTTCCAGAACTTATCTGTTGGAAGTAAATGAGTTTCCA
TAAGGCCAGGGAAACGCAGGTAGGAACCCATGCGGTCGAGCCAGCACTCACCTGACACTA
GGAACCGCTGGCCAAAGGTTTTGTTGCCGAAACTCTCAAAGTTGAAATGGTCCATGTATT
GCTCAAAATAATTCTCATGAAAGTCAGGGTCTAGAACTCTGTCGGCTGAGGGCAGGTGCA
GAGACTCAGG

>181 Get primers
CATAGGATGTTGAGACTGTAAGAGACATCTCTTTGGCCCTCCTTGTATAGGGTGTCAATC
GGCACAACAGGGTGGAGCCTTAGAGTAGGGTAAGATTAGGACTCTAGGTTCTCTCATGGG
TCCAGATCTGTCATGAAGGGAGGTCAAGGACCCACCTCCCTCCAAAGGCTATGGTGGGGG
CATCATGGACCCCAAGGTCCTGCCGCAGGGCCTGGAGCAGCACAGCAAAGTCGGCCAGCG
CCTGCTCCACAGTCAGCAGCTGTGTATATCCCCGCTGTGTGGACTGGACACCGAACGGAA
GCGATTTCCCATAGTACCGCTGCAGAAAGCAGGAAGGGATGGCTAATCCACTCCTCGGTG
CTCCCCACCTCCTTCAACTCAGGGACTGCCAGGAACTGTACAGGTACCCACGTGCTCAGC
AAAGACAAGCAGGGCCTCCTGCTGGGCTGCCAGTTCCACCATGAAGCCAGAGTTGTTAGC
GAAGGACCAGATATCCCCCTCATTCCCTGTGTAGAAAAAGATGGGCCCTTCGCCCATCTT
CCAGAACTTATCTGTTGGAAGTAAATGAGTTTCCATAAGGCCAGGGAAACGCAGGTAGGA
ACCCATGCGGTCGAGCCAGCACTCACCTGACACTAGGAACCGCTGGCCAAAGGTTTTGTT
GCCGAAACTCTCAAAGTTGAAATGGTCCATGTATTGCTCAAAATAATTCTCATGAAAGTC
AGGGTCTAGAACTCTGTCGGCTGAGGGCAGGTGCAGAGACTCAGGAGCTGGTTGGGATCA
TCAGGGATCTAGGCGGGTCAGGAGGAAGGGCAGCCAGTCTGTACTCACCTCTGGCCTGGA
GGTTGCACAG

>182 Get primers
TAGGGTAAGATTAGGACTCTAGGTTCTCTCATGGGTCCAGATCTGTCATGAAGGGAGGTC
AAGGACCCACCTCCCTCCAAAGGCTATGGTGGGGGCATCATGGACCCCAAGGTCCTGCCG
CAGGGCCTGGAGCAGCACAGCAAAGTCGGCCAGCGCCTGCTCCACAGTCAGCAGCTGTGT
ATATCCCCGCTGTGTGGACTGGACACCGAACGGAAGCGATTTCCCATAGTACCGCTGCAG
AAAGCAGGAAGGGATGGCTAATCCACTCCTCGGTGCTCCCCACCTCCTTCAACTCAGGGA
CTGCCAGGAACTGTACAGGTACCCACGTGCTCAGCAAAGACAAGCAGGGCCTCCTGCTGG
GCTGCCAGTTCCACCATGAAGCCAGAGTTGTTAGCGAAGGACCAGATATCCCCCTCATTC
CCTGTGTAGAAAAAGATGGGCCCTTCGCCCATCTTCCAGAACTTATCTGTTGGAAGTAAA
TGAGTTTCCATAAGGCCAGGGAAACGCAGGTAGGAACCCATGCGGTCGAGCCAGCACTCA
CCTGACACTAGGAACCGCTGGCCAAAGGTTTTGTTGCCGAAACTCTCAAAGTTGAAATGG
TCCATGTATTGCTCAAAATAATTCTCATGAAAGTCAGGGTCTAGAACTCTGTCGGCTGAG
GGCAGGTGCAGAGACTCAGGAGCTGGTTGGGATCATCAGGGATCTAGGCGGGTCAGGAGG
AAGGGCAGCCAGTCTGTACTCACCTCTGGCCTGGAGGTTGCACAGTCCCAGTGACAGCAG
CAGGACCAGGATCCAGGAGGGGACACCATGGTCCACAGGGTAACAAGGATGGAAGTTCAT
GCTTGATTCT

>183 Get primers
ATGGTGGGGGCATCATGGACCCCAAGGTCCTGCCGCAGGGCCTGGAGCAGCACAGCAAAG
TCGGCCAGCGCCTGCTCCACAGTCAGCAGCTGTGTATATCCCCGCTGTGTGGACTGGACA
CCGAACGGAAGCGATTTCCCATAGTACCGCTGCAGAAAGCAGGAAGGGATGGCTAATCCA
CTCCTCGGTGCTCCCCACCTCCTTCAACTCAGGGACTGCCAGGAACTGTACAGGTACCCA
CGTGCTCAGCAAAGACAAGCAGGGCCTCCTGCTGGGCTGCCAGTTCCACCATGAAGCCAG
AGTTGTTAGCGAAGGACCAGATATCCCCCTCATTCCCTGTGTAGAAAAAGATGGGCCCTT
CGCCCATCTTCCAGAACTTATCTGTTGGAAGTAAATGAGTTTCCATAAGGCCAGGGAAAC
GCAGGTAGGAACCCATGCGGTCGAGCCAGCACTCACCTGACACTAGGAACCGCTGGCCAA
AGGTTTTGTTGCCGAAACTCTCAAAGTTGAAATGGTCCATGTATTGCTCAAAATAATTCT
CATGAAAGTCAGGGTCTAGAACTCTGTCGGCTGAGGGCAGGTGCAGAGACTCAGGAGCTG
GTTGGGATCATCAGGGATCTAGGCGGGTCAGGAGGAAGGGCAGCCAGTCTGTACTCACCT
CTGGCCTGGAGGTTGCACAGTCCCAGTGACAGCAGCAGGACCAGGATCCAGGAGGGGACA
CCATGGTCCACAGGGTAACAAGGATGGAAGTTCATGCTTGATTCTGAGCCGGGCGCTGAC
TGTCATGTGATTTGGTCACATGACCGACACAACGGGCGGGGCAGCATCACGTGATAGTCT
GGCGGGGGCT

>202 Get primers
CCTGCAACAGCTACAACAGGAGCGCTGGCTGCCAGTGCCCCAGCCACCAGGTGGGGGTAC
TTCATCCTCATGTAGGCACTCAGCATCCCCCCATAACTGGGAGTACAGAGCACAGATCAT
GGTTGTGGGAAGCTGCCCACAACTCAGGCGAGCAGCCTCACTGTCCTCCAGGCTGAGGTG
[truncated: 42,662 more chars]
