# Supplementary figures and images for: Automated design of genomic Southern blot probes
Source: BMC Genomics. 2010 Jan 29;11:74. doi: 10.1186/1471-2164-11-74 (PMC2830989; doi:10.1186/1471-2164-11-74)

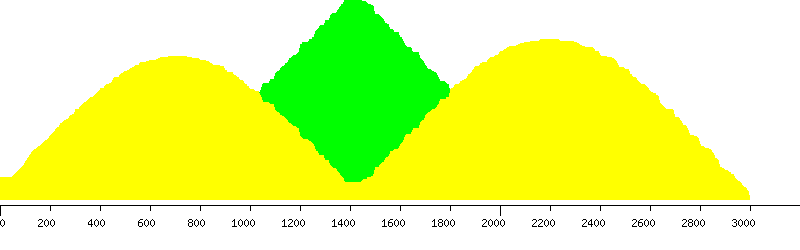

Supplement: Additional file 1 — Software package for automated design of genomic Southern blot probes. Archive of all the components of the pipeline packaged using "tar", and subsequently compressed with "gzip". Includes source code, example configuration files, example output, and a user's guide for installation. [file 1471-2164-11-74-S1.GZ › southern_blot_design/docs/example_run_output/test_diag.png]
